# An Ancestry Perspective of the Evolution of PBS1 Proteins in Plants

**DOI:** 10.3390/ijms22136819

**Published:** 2021-06-25

**Authors:** Edgar Yebrán Villegas-Vázquez, Beatriz Xoconostle-Cázares, Roberto Ruiz-Medrano

**Affiliations:** Departamento de Biotecnología y Bioingeniería, Centro de Investigación y de Estudios Avanzados del Instituto Politécnico Nacional. Av. IPN 2508 San Pedro Zacatenco, México City 07360, Mexico; eyebran.villegas@cinvestav.mx

**Keywords:** PBS1, RPS5, evolution, cleavage site motif, resistance recognition motif

## Abstract

The AVRPPHB SUSCEPTIBLE1 (PBS1) and RESISTANCE TO PSEUDOMONAS SYRINGAE 5 (RPS5) proteins are involved in signal transduction to evoke innate plant immune response. In Arabidopsis, PBS1 is cleaved by the AvrPphB (*Pseudomonas phaseolicola* Avirulence protein B) protease, activating RPS5 and turning in a hypersensitive response (HR). We searched for PBS1 orthologs to trace their origin and evolution. PBS1 orthologs were found in embryophytes and in other plant taxa but with lower similarity. PBS1 phylogenetic analysis indicates high divergence, suggesting that the decoy function described for Arabidopsis PBS1 might be associated with a small fraction of orthologs. Ancestral reconstruction analysis suggests an elevated diversity in the amino acid sequence within the described motifs. All the orthologs contain the conserved PBS1 kinase subdomains, whereas the cleavage motif is present in several embryophyte orthologs but absent in most other taxa. The putative resistance recognition motifs in PBS1 orthologs are highly diverse. PBS1 cleavage site motif is exposed in some 3D structure predictions, whereas it is not in others, suggesting different modes of regulation and functions in PBS1 orthologs. Our findings suggest that PBS1 originated in the lineage that gave rise to embryophytes, with the angiosperm sequences forming a separate clade from pteridophyte proteins.

## 1. Introduction

Plants, in general, and particularly terrestrial plants, have coexisted with a variety of microorganisms for hundreds of millions of years through different types of interactions [1]. Indeed, the colonization of terrestrial by plants was possible due to their association with symbiotic fungi [2]. The coexistence of plants and environmental microbes led to the evolution of plant systems involved in the interaction with the latter, including pathogens [3]. Such continuous interaction resulted in the increased genome complexity of both plants and their interacting microorganisms [2].

Plants possess diverse defense strategies against pathogens; the first includes pattern recognition receptor proteins (PRRs) [3,4], which recognize microbial- or pathogen-associated molecular patterns (MAMPs or PAMPs), such as bacterial flagellin or fungal cell wall fragments. Such recognition activates PAMP-triggered immunity (PTI) [3,5,6]. Plants harbor different receptor-like kinases (RLKs) that function as PRRs [1,7]. RLKs consist mainly of an N-terminal ligand-binding domain, a transmembrane domain and a C-terminal kinase domain [8,9]. These play roles in abiotic stress, plant–microbe interactions, and plant development [10,11]. One subset is the family of receptor-like cytoplasmic kinases (RLCK) consisting of a family of VII RLKs that participate in PTI [9,12]. Certain pathogens can suppress the immune response by delivering effectors that inactivate it, thus contributing to pathogen virulence. As a counter defense mechanism, plants harbor resistance proteins that recognize such effectors directly or indirectly. These belong to the nucleotide binding-site leucine-rich repeat protein family (NBS–LRR or NLR) involved in effector-triggered immunity (ETI) characterized by the hypersensitive response (HR) leading to host programmed cell death [2,3,13].

A remarkable example of PRR, which has been recruited in some species for ETI, is the PBS1 kinase and a number of PBS1-like (PBL) proteins. PBS1 is one of the most conserved serine/threonine protein kinases in flowering plants, which is anchored to the plasma membrane by N-terminal *S*-acylation [14]. The Arabidopsis PBS1 (AthPBS1) is cleaved by AvrPphB, a cysteine protease effector from *Pseudomonas syringae*, internalized by a type-three secretion system (TTSS) [15]. The cleavage occurs in the GDK motif, resulting in a conformational change; this exposes another motif (the SEMPH sequence, localized in the C-terminal loop) that is recognized and bound by the NLR protein family member RPS5. This interaction activates RPS5, which in turn leads to the subsequent induction of HR [16,17]. RPS5 localizes to the plasma membrane and associates to PBS1 through its amino terminal coiled coil (CC) domain [16]. Therefore, PBS1 may serve as a decoy, guarded by RPS5 to detect effector activity [18]. AvrPphB cleaves other PBL proteins, such as BIK1 and PBL1, that physically associate with the flagellin-detecting receptor (FLS2); these cleaved proteins are unable to activate PTI through FLS2, interfering with the host immune response [19,20].

The function of PBS1 has been studied in a few species; while highly conserved, it remains to be determined if its function, and by extension, the whole PBS1-RPS5 system, is similarly conserved across different taxa. Recent evidence suggests that this is the case in some related species, such as *Triticum aestivum* and *Hordeum vulgare* PBS1 [9,21,22]. However, the presence of potential orthologs in species including bryophytes and chlorophytes has been seldom studied.

The vast amount of genomic data can now allow the analysis of PBS1 orthologs in different plant species, which may provide insights into the origin and evolution of resistance proteins. Additionally, the analysis of the diversity and/or conservation of both the cleavage site (GDK) and resistance recognition (SEMPH) motifs in plants can yield information in this regard.

In this study, an evolutionary analysis of PBS1 was carried out, using as a model the Arabidopsis protein AthPBS1. The aim was to analyze PBS1 orthologs in Viridiplantae through the characterization of kinase subdomains and motifs in PBS1 in both chlorophytes and terrestrial plant. Furthermore, an ancestral reconstruction of PBS1 motifs as well as predictive structural analysis of PBS1 orthologs proteins in different linages of terrestrial plants was carried out. The results presented here suggest that a PBS1-RPS5 system arose after the divergence of chlorophytes from terrestrial plants. Furthermore, such system emerged independently in certain lineages, according to phylogenetic and structural considerations.

## 2. Results

**Figure 1:**
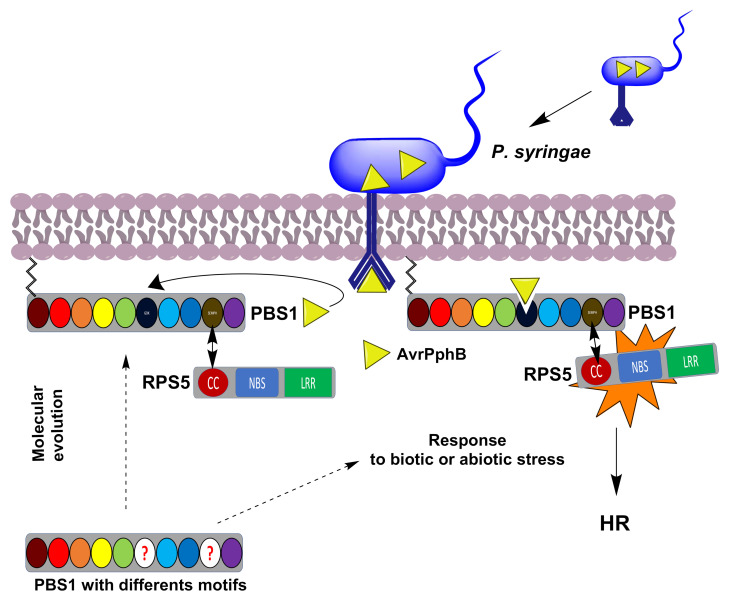
Schematic representation of the *P. syringae* AvrPphB effector activation of RPS5 mediated by PBS1 leading to the defense response. PBS1 is normally associated, through its SEMPH motif, to the RPS5 CC domain, forming a complex in which the latter is inactive. Afterwards, the AvrPphB effector (yellow triangle), injected by *P. syringae*, binds and cleaves PBS1 in its GDK motif. This induces a conformational change in RPS5, thus activating the HR. The evolutionary origin of the GDK and SEMPH motifs in Arabidopsis and other plants is not clear. A role for the PBS1-RPS5 system in activation of the defense response in other plant lineages is unknown. The circles correspond to the PBS1 motifs and kinase subdomains (GxGxxG, burgundy; K, red; E, orange; DxxxSN, yellow; DFG, green; GDK motif, dark blue; APE, light blue; DxxxG, blue; SEMPH motif; dark yellow; and R, purple). The circle with red questions indicates different motifs. Dotted arrows indicates a possible to the classic PBS1-RPS5 system or a possible response to abiotic or biotic stress. An arrow indicates interaction between the SEMPH and CC domains.

The origin of the PBS1-RPS5 system in Arabidopsis and other plants is not clear. Thus, their homologs were searched for in the annotated genomes and other databases from embryophytes, bryophytes, pteridophytes, streptophyte algae, and chlorophytes in order to gain insight into the evolution of the genes underlying this defense mechanism, and shed light on the evolutionary origins of defense mechanisms that involve interaction between a pathogen effector and plant resistance proteins (Figure 1).

### 2.1. Identification of PBS1 Proteins, Conserved Kinase Subdomains and Motifs Involved in the Indirect Immune Response in PBS1 in the Plant Kingdom

Considering that AthPBS1 contains conserved kinase subdomains, the GDK and SEMPH motifs were used as queries to search for these motifs and subdomains in PBS1 homologs from diverse plant taxa (see Materials and Methods, Section 4). After removing redundant sequences, 881 sequences were identified. To ensure that ortholog sequences were obtained, an analysis was performed with OrthoMCL (Supplementary File S2), which yielded 881 ortholog sequences (Supplementary File S1) corresponding to angiosperms, gymnosperms, bryophytes, pteridophytes, lycophytes, liverworts, charophytes and chlorophytes. To gain insight into the evolution of the aforementioned motifs and domains, three analyses were carried out with the MEME program. First, with 116 PBS1 ortholog sequences corresponding to chlorophytes; second, with 765 PBS1 orthologs of terrestrial plants; and finally, with 182 PBS1 orthologs, which showed the highest similarity from each species and genus of the 765 PBS1 sequences analyzed.

**Figure 2:**
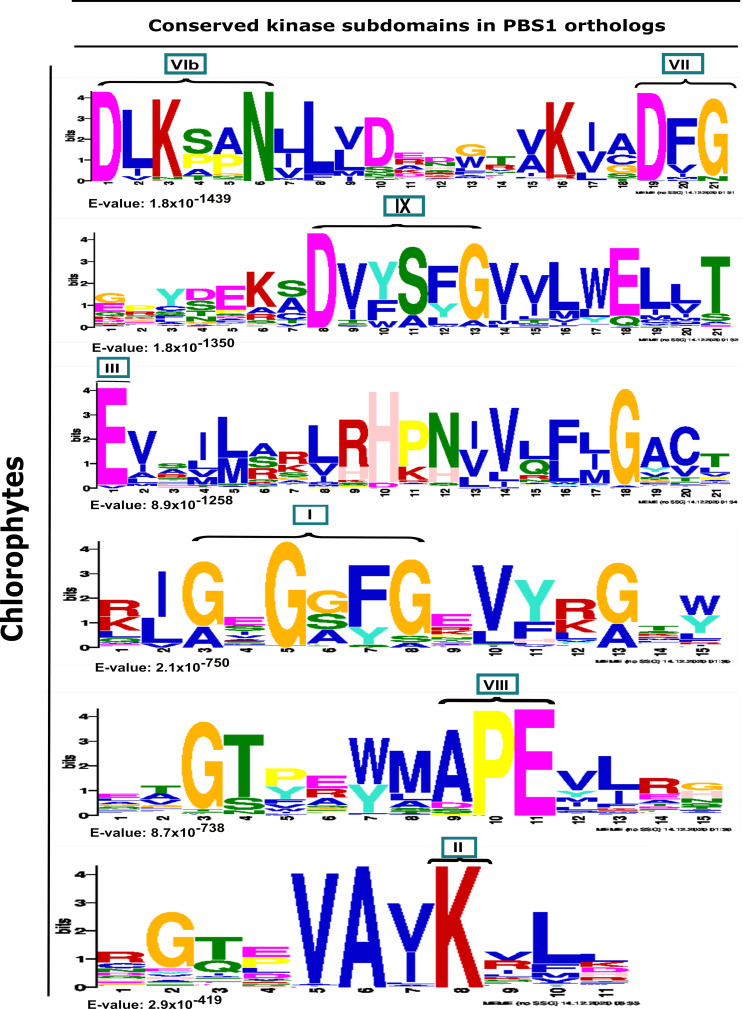
Analysis of conserved kinase subdomains in PBS1 orthologs in chlorophytes. Sequence logo representation of multiple sequence alignment of 116 sequences from chlorophytes with high similarity to Arabidopsis PBS1 were identified by MEME analysis. The domains are ordered from lowest to the highest expectation (E) values. The conserved kinase subdomains are labeled with Roman numerals. Subdomain I: GxGxxG; II: K; III: E; VIb: DxxxxN; VII: DFG; VIII: APE; IX:DxxxxG; XI: R. PBS1 orthologs in chlorophytes do not contain the GDK cleavage site and the recognition motif required for RPS5-mediated plant resistance.

**Figure 3:**
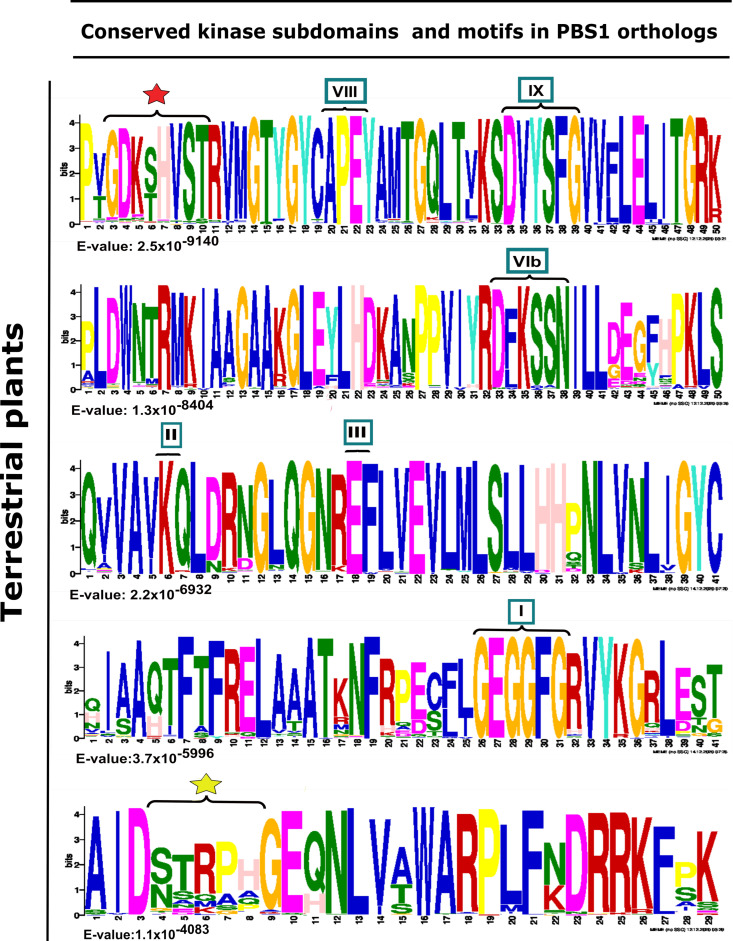
Analysis of conserved kinase subdomains and important motifs in PBS1 orthologs in terrestrial plants. Sequence logo representation is shown in multiple sequence alignment of 182 sequences with high similarity to Arabidopsis PBS1 from monocots (37/182), eudicots (117/182), bryophytes (7/182), charophytes (2/182), gymnosperms (7/182), lycophytes (7/182), liverwort (1/182), and pteridophyte (4/182). The domains are ordered from lowest to highest expectation (E) values. The conserved kinase subdomains are labeled with Roman numerals. Subdomain I: GxGxxG; II: K; III: E; VIb: DxxxxN; VII: DFG; VIII: APE; IX:DxxxxG; XI: R. The AvrPphB cleavage site GDK is labeled with a red star, and the recognition motif required for RPS5-mediated resistance is labeled with a yellow star.

Analysis from chlorophytes showed that these sequences contain the conserved kinase subdomains (Figure 2). Interestingly, neither the GDK nor SEMPH motifs are present in the PBS1 orthologs of the chlorophytes studied herein (Figure 2). A more detailed analysis revealed the consensus sequence of kinase subdomains in these proteins (Figure 3). The conserved kinase subdomains (labeled with Roman numerals) corresponded to the previously reported subdomains, namely, I: GxGxxG; II: K; III: E; VIb: DxxxxN; VII: DFG; VIII: APE; IX: DxxxxG; XI: R, some of which are conserved in several PBS1 proteins (Figure 2, Figure 3 and Figure S1).

**Figure 4:**
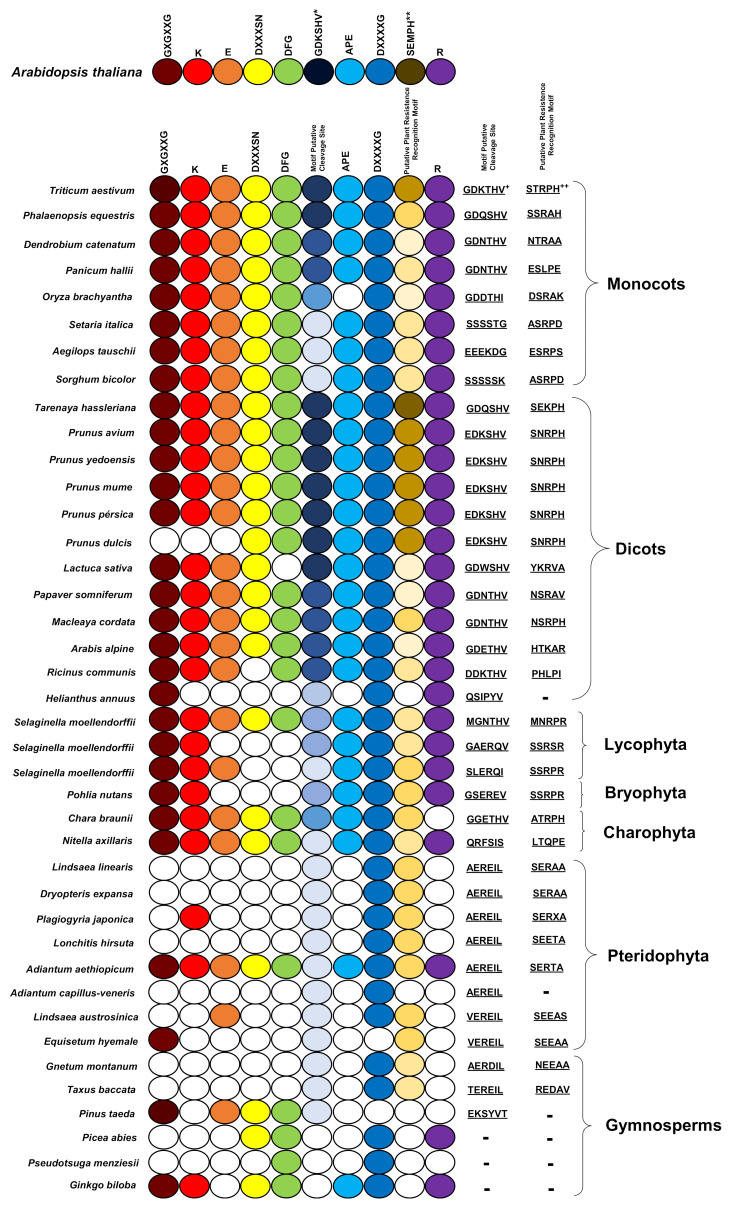
Comparison of conserved kinase subdomains and motifs in PBS1 orthologs in terrestrial plants. Forty sequences from PBS1 orthologs in terrestrial plants were analyzed. Sequences were compared with Arabidopsis PBS1. Selected monocot, dicot, bryophyte, lycophyte, and chlorophyte species harbor most of the kinase subdomains, but these are more variable; in gymnosperms and pteridophytes, the kinase and motif subdomains are less conserved. Color keys are as in Figure 1. The putative motif cleavage site is indicated with a dark blue circle, the blue hue decreasing if there is one or more amino acid changes relative to the motif indicated in Arabidopsis. The same applies to the putative plant resistance recognition motif but in dark yellow. White circles indicate the absence of a subdomain or motif. All motif putative cleavage sites and putative plant resistance recognition motifs identified are listed in Supplementary Table S1. *, ** Motifs are taken as a reference for the analysis. ^+^, ^++^ Motifs with greater abundance in different species; see Supplementary Table S1.

Furthermore, the kinase subdomain, as well as the GDKmotif, is highly conserved in PBS1 orthologs in terrestrial plants. However, pteridophytes and gymnosperms lack these (Supplementary Table S1). Different motifs were identified in the analyzed species, which differ from one to six amino acids in the putative cleavage site relative to the AthPBS1 canonical motif (Figure 4). On one hand, sequences from certain monocot, dicot, bryophyte, lycophytes, liverwort, and charophyte species show little variation; however, pteridophytes and gymnosperms contain completely different motifs (Figure 4, Supplementary Table S1). Interestingly, logo sequences in terrestrial plants showed higher similarity between GDKSHV and GDKTHV motifs (Figure 2 and Figure S1 and Supplementary Table S1). The SEMPH motif was found in nine dicot species. Interestingly, different putative resistance recognition motifs were identified in the PBS1 orthologs analyzed (Supplementary Table S1). The putative resistance recognition motifs were defined as those motifs similar in position and amino acid composition to the SEMPH motif but with an unknown function Among the resistance recognition motifs found in these sequences, the most conserved was the STRPH motif (Figure 3, Supplementary Table S1). The GDK motif is present in lycophytes, liverworts, charophytes (*Klebsormidium nitens*), and angiosperms (Supplementary Table S1). However, it is absent in chlorophytes. Interestingly, some species within gymnosperms, lycophytes, bryophytes, and charophytes also lack such motif (Figure 4 and Supplementary Table S1). The resistance recognition motifs are the most variable ones in different species (Figure 3 and Supplementary Table S1). This may allow the interaction of PBS1 with a wide range of downstream NBS-LRR proteins (including RPS5), in turn giving rise to a wide range of responses depending on the nature of the effector.

### 2.2. Phylogeny of PBS1 Orthologs in Chlorophytes

**Figure 5:**
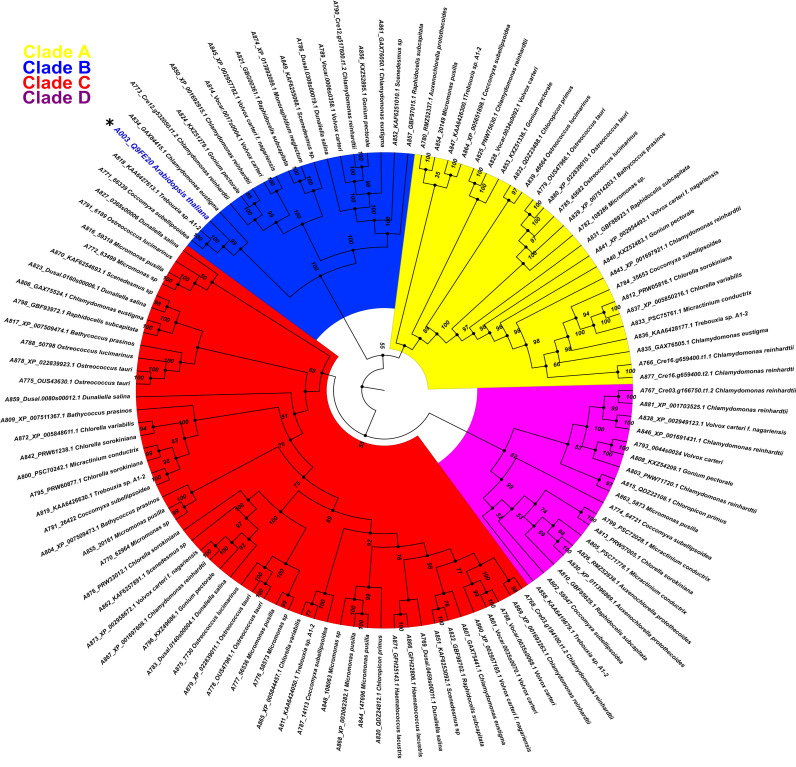
Phylogenetic relationship between PBS1 of Arabidopsis and orthologs protein kinases from chlorophytes. A total of 116 amino acid sequences with high similarity to PBS1 were retrieved from different databases and aligned using ClustalW. Maximum likelihood phylogeny was constructed based on the deduced amino acid sequences of PBS1 orthologs from different chlorophyte species. Different colors indicate clades for different kinases orthologs. Position of Arabidopsis in the tree in clade B, is indicated with an asterisk (*) and blue color.

One hundred and sixteen amino acid sequences from chlorophyte species with high similarity to AthPBS1 (Arabidopsis ID Q9FE20) were retrieved from different databases (see Materials and Methods, Section 4). Thus, the chlorophytes *Dunaliella salina*, *Chlamydomonas reinhardtii*, *Volvox carteri*, *Micromonas sp.*, *Micromonas pusillis*, *Ostreococcus lucimarinus*, *Auxenochlorella protothecoides*, *Chlorella sorokiniana*, *Gonium pectorale*, *Raphidocelis subcapitata*, *Micractinium conductrix*, *Bathycoccus prasinos*, *Chlamydomonas eustigma*, *Trebouxia sp.*, *Chloropicon primus*, *Scenedesmus sp.*, and *C. subellipsoidea* harbor in their genome’s gene coding for protein kinases with orthology to AthPBS1. Interestingly, a tyrosine kinase from *Coccomyxa subellipsoidea* and two hypothetical proteins from *C. eustigma* and *Trebouxia sp.*, respectively, grouped with AthPBS1 (A003) in the same clade (B) with considerable similarity to AthPBS1 (Figure 5).

The phylogenetic relationship between AthPBS1 and chlorophyte orthologs was analyzed using the maximum likelihood method, executed in IQ-TREE (Figure 5). The resulting phylogeny shows four major clades (A, B, C and D) clustered with related species from chlorophyte and protein kinases from this or closely related groups with high bootstrap values in branches (Figure 5). A preliminary analysis identified kinase subdomains GxGxxG, K, E, DxxxSN, DFG, APE, DxxxG, and R in the PBS1 orthologs from chlorophytes (Figure 2). Additionally, AthPBS1 was clustered in clade B (Figure 5) with different protein kinases that contain kinase subdomains (Hanks et al., 1995). Furthermore, The *C. subellipsoidea* PBS1 (which, as mentioned earlier, is a protein tyrosine kinase with high similarity to AthPBS1) harbors the classical kinase domains and is clustered in the same clade as AthPBS1 (Figure 5, clade B). These results indicate that different orthologous kinases from chlorophytes are phylogenetically related based on their high similarity. This finding lends credence to the hypothesis that PBS1 has evolutionary counterparts in chlorophytes. However, the SEMPH and the GDK motifs were absent in chlorophyte PBS1 orthologs (Figure 5). Therefore, the subdomains of these protein kinases present synapomorphy, since these characters are intrinsic to protein kinases; however, the motifs present in AthPBS1 may belong to a homoplastic character since it could have been acquired independently. Given this perspective, AthPBS1 orthologs were analyzed in terrestrial plants.

### 2.3. Phylogenetic Analysis of PBS1 Orthologs from Different Plant Lineages

**Figure 6:**
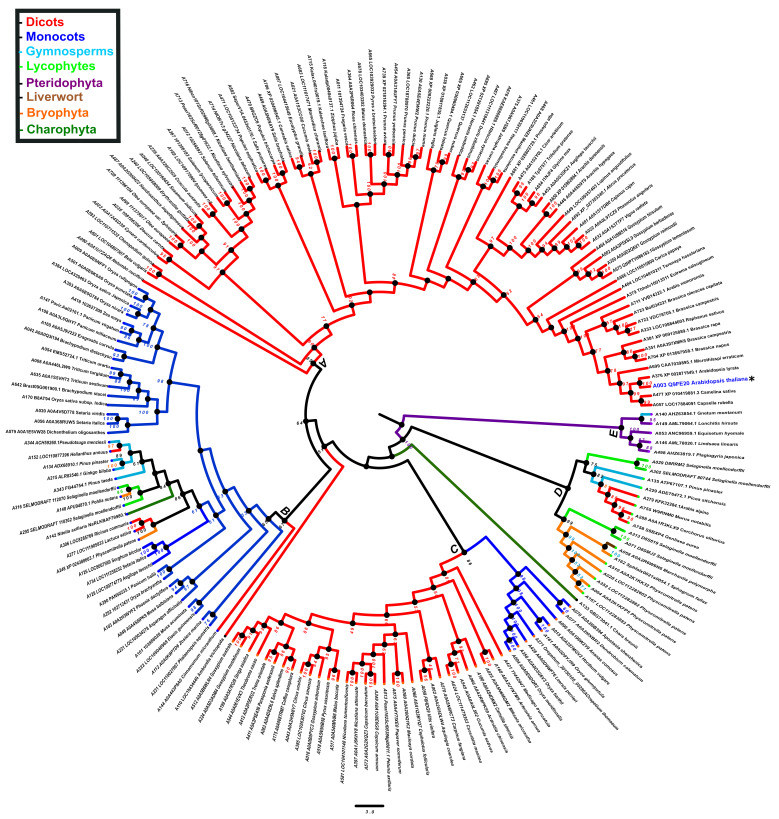
Phylogenetic analysis of PBS1 orthologs in terrestrial plants. A total of 182 amino acid sequences from Arabidopsis PBS1 orthologs were obtained from different databases and aligned using ClustalW. A maximum likelihood phylogenetic tree was constructed based on the deduced amino acid sequences PBS1 proteins from several terrestrial plants. Red (clade A) indicates eudicots; blue (clade B and C), monocots; purple (clade E), pteridophytes; orange (clades B and D), bryophytes; dark green (clade B), charophytes; light blue (clades B, D and E), gymnosperms; light green (clades B and D) lycophytes and brown (clade D) liverwort. Position of Arabidopsis in the tree is indicated with asterisk (*) and blue color.

To explore the evolutionary relationship of PBS1 orthologs in major plant domains, a phylogeny was constructed with 881 PBS1 orthologous sequences with considerable similarity to AthPBS1 (Supplementary Table S2). However, the results obtained were not conclusive, because the amount of polytomies observed made it difficult to resolve their phylogenetic relationships (Supplementary Figure S2). Thus, fewer sequences were used to construct another phylogeny until an optimized tree was obtained. Finally, the phylogenetic analysis was performed with 182 PBS1 orthologous sequences using maximum likelihood. The results showed five major clades (A, B, C, D, and E); clade A is subdivided into different subclades; only PBS1 orthologs of dicots were grouped into clade A, which contained AthPBS1 (A003) (Figure 6). Interestingly, clade B was made up of PBS1 orthologs of monocots, dicots, lycophytes (A295, A316), bryophytes (A148, A248), gymnosperms (A344, A134, A215, and A343), and charophytes (A142) (Figure 6). Clade C is subdivided into two clades including monocots and dicots only. Curiously, a branch of *Chara brauni* (A133) emerges from the three clades analyzed, suggesting a possible diversification from a basal ancestor of this charophyte. On the other hand, clade D is also subdivided into two subclades, one clustering PBS1 orthologs of *Physcomitrella patens* (A164, A064, A352, A028, and A510), *Selaginella moellendorffii* (A071 and A313), *Sphagnum fallax* (A162), and *Marchantia polymorpha* (A058), while the other included dicots (A758, A288, and 755), lycophytes (A029 and A302), and gymnosperms (A229 and A135). Finally, pteridophytes (A498, A146, A053, and A149) and one gymnosperm (A140) were grouped in clade E (Figure 6). These results indicate that different PBS1 orthologous proteins are phylogenetically related. However, clades B and D contain proteins from diverse taxa, suggesting convergent evolution within these groups.

### 2.4. Ancestral State Reconstruction of PBS1 Orthologs in Terrestrial Plants

**Figure 7:**
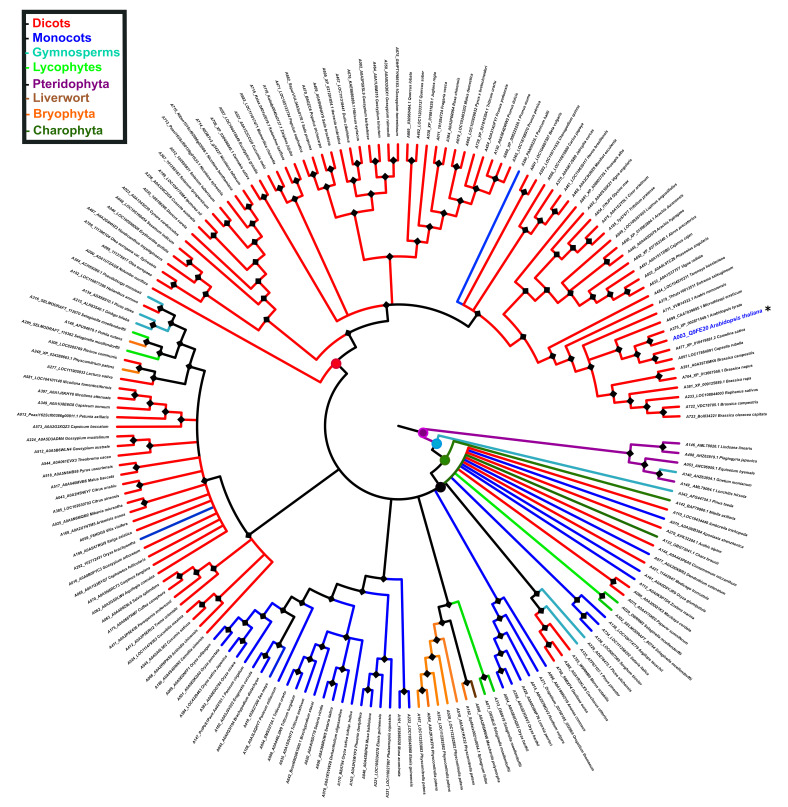
Reconstruction of PBS1 ancestral ortholog sequences in terrestrial plants. The ancestral tree was produced by Bayesian statistics using 182 sequences from monocots (blue), dicots (red), bryophytes (orange), liverwort (brown), lycophyte (light green), gymnosperm (light blue), charophytes (dark green), and pteridophytes (purple). Circles in nodes indicate mainly ancestors. Position of Arabidopsis in the tree is indicated with asterisk (*) and blue color. The analysis of PBS1 ancestral sequences was obtained with the Mesquite software (http://www.mesquiteproject.org, accessed on 6 February 2021).

To gain insight into the evolution of PBS1 in terrestrial plants, its ancestral reconstruction was carried out. In this regard, 182 PBS1 orthologs were used to infer the phylogenetic tree with the Bayesian method. Ancestral state reconstruction was obtained by maximum parsimony; based on these results, we investigated the phylogeny of species from terrestrial plants. The phylogeny shows that PBS1 orthologs from pteridophytes form an independent branch originating from the first ancestral node (purple circle). Next, a series of ancestors originated from different branches, which clustered with different species of streptophytes (charophytes, bryophytes, liverworts, lycophytes, gymnosperms, monocots, and dicots). The second ancestral node (light blue circle) gave rise to a branch that contains solely a gymnosperm (A343), and another one that includes a third ancestral node (dark green circle). This includes a branch containing a charophyte (A142) as well as a fourth ancestral node (black circle), which diverged into different PBS1 orthologs from terrestrial plants, except pteridophytes (Figure 7). Finally, a fifth ancestral node (red circle) gave rise to different clades exclusively containing dicot as well as one monocot species (A290) (Figure 7).

**Figure 8:**
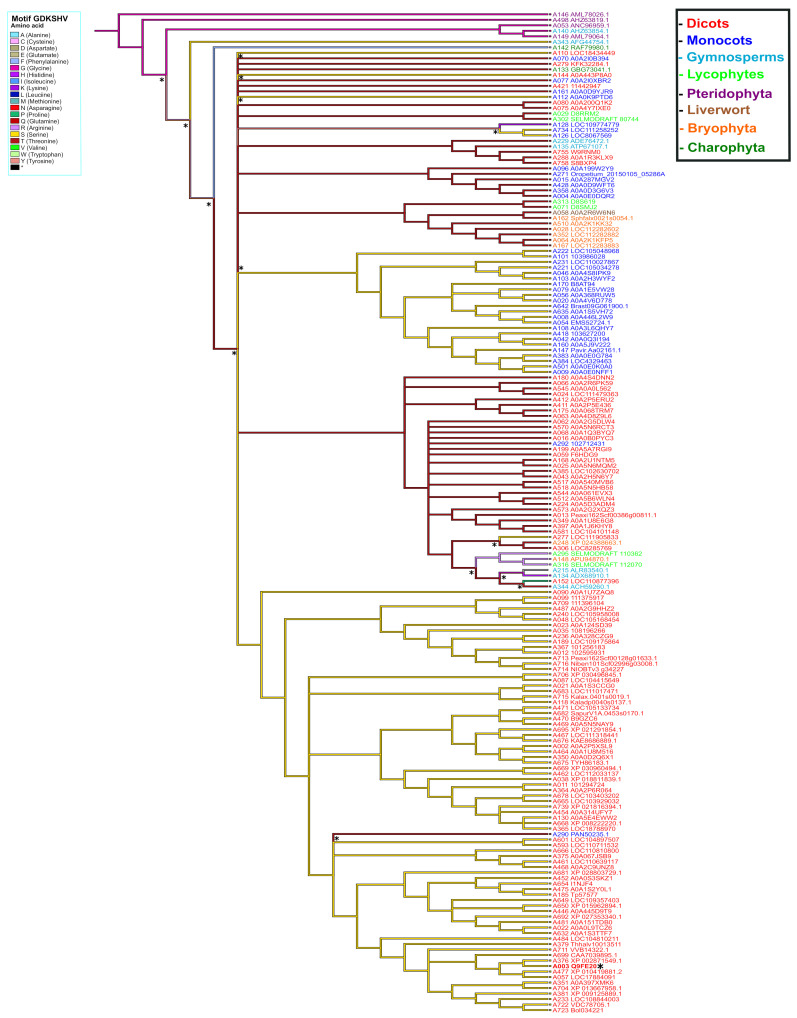
Ancestral state reconstruction in PBS1 orthologs based on the GDKSHV motif. Ancestor reconstruction was carried out with the maximum parsimony algorithm using as ancestral character the PBS1 amino acid sequence. Reconstruction was carried out on amino acid 248 of PBS1. The colors in the branches indicate the evolutionary divergence of the ancestral amino acid. The color in the names indicates terrestrial plants species. Transitions and reversion are indicated with asterisks (*) above nodes. This figure shows the ancestral state of serine (S). Position of Arabidopsis in tree is indicated with asterisk (*) and bold red to observe the ancestry of other amino acids; see Supplementary Figures S2 and S3.

To elucidate the possible origin of the GDKSHV and SEMPH motifs, the reconstruction of ancestral character was performed for each amino acid position. We analyzed positions 245 to 250 for the GDKSHV motif and positions 296 to 300 for the SEMPH motif. The reconstruction of character evolution revealed a single change in the character states of the amino acids in positions 245 (ancestral character glycine), 246 (ancestral character aspartate), 247 (ancestral character arginine, diverging to lysine) (Supplementary Figure S3), 249 (ancestral character leucine, diverging to histidine), and 250 (ancestral character lysine, diverging to valine) (Supplementary Figure S4). However, the most relevant change occurred in position 248, the ancestral amino acid being glycine (magenta) that later diverged into threonine (deep red) and serine (yellow) in different species (Figure 8). These results are in agreement with the analysis carried out with MEME (Figure 3).

**Figure 9:**
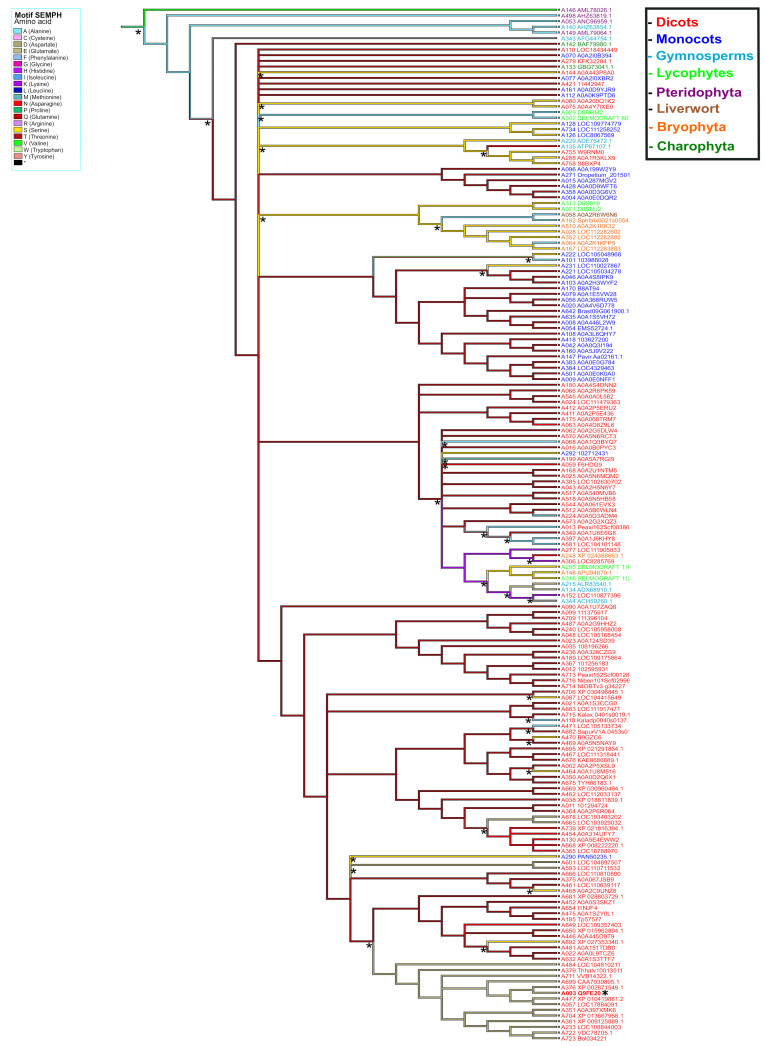
Ancestral state reconstruction in PBS1 orthologs based on the SEMPH motif. Ancestor reconstruction was performed with maximum parsimony using as ancestral character the PBS1 amino acid sequence. Reconstruction of ancestral state in amino acid 297 position of PBS1. Branch color indicates the evolutionary divergence of the ancestral amino acid. Accession numbers are shown in red. Transitions and reversions are indicated with asterisks above nodes. This figure shows the ancestral state of glutamic acid (E). Arabidopsis is indicated with asterisk (*) and bold red. For ancestry of other amino acids, see Supplementary Figures S4 and S5.

The ancestral distributions of the SEMPH motif were the most diverse. The ancestral character at position 296 was arginine (light purple); this amino acid diverged mostly to serine (yellow) and asparagine (red) (Supplementary Figure S5). In position 297, the ancestral character has two origins, alanine and valine; however, alanine was more conserved. The evolution of this character diverged mainly to threonine (deep red). Moreover, a clade containing Arabidopsis diverged to the ancestral character glutamate (gray) (Figure 9). The ancestral character in position 298 has two origins, isoleucine (blue) and arginine (light purple), conserved in most species. Additionally, a clade diverged to the ancestral character methionine (light blue). Arginine is the principal ancestral character in position 299 and primarily diverged to proline (light green). Finally, the ancestral character in position 300 was glutamate and mainly diverged to histidine (purple) (Supplementary Figure S6). This analysis suggests that substitutions in certain positions in the SEMPH motif have been favored throughout evolution. This was likely a result of the selection of those PBS1 variants that showed enhanced ability to elicit a defense response through RPS5 or functionally analogous proteins.

### 2.5. Structure Modeling of PBS1 Orthologs

**Figure 10:**
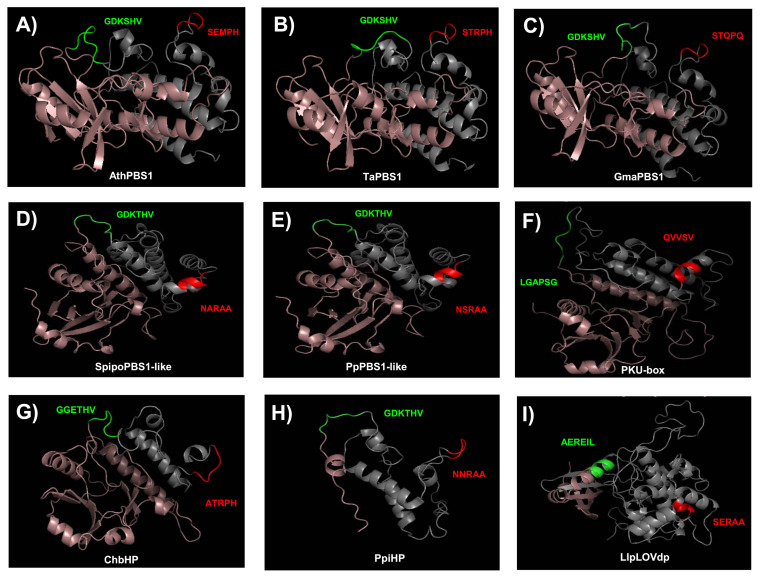
Predicted structures of PBS1 from several plant species. Swiss Predictive structures for PBS1 orthologs were generated with Swissmodel. (**A**) AthPBS1 from Arabidopsis (amino acid residues 71 to 366); (**B**) TaPBS1 from *T. aestivum* (residues 89 to 386); (**C**) GmaPBS1 from *G. max* (residues 84 to 381); (**D**) SpipoPBS1-like from *S. polyrhiza* (residues 72 to 356); (**E**) PpPBS1-like from *P. patens* (residues 71 to 361); (**F**) CrePKU from *C. reinhardtii* (residues 474 to 786); (**G**) ChbHP from *Ch. braunii* (residues 16 to 249); (**H**) PpiHP from *P. pinaster* (residues 2 to 123); (**I**) LlpLOVdp from *L. linearis* (residues 346 to 689). Models for AthPBS1, TaPBS1, GmaPBS1, and ChbHP were based on the BIK1 structural model (SMTl ID: 5tos.1). Models for CrePKU and PpiHP were based on the PK (SMTI ID: 6j5t.1). Models for SpipoPBS1, PpPBS1-like, and LlpLOVdp were based on BAK1 (SMTI ID: 3tl8.1), BR1 (SMTI ID: 5lpz.1), and BAC (SMTI ID:6npz.1. A). Cleavage site (GDKSHV) and putative cleavage sites are represented in green. The RPS5 recognition motif (SEMPH) and putative RPS5 recognition motifs are represented in red. Structure from the N-end to the putative cleavage site motif is shown in pink. Structure from putative RPS5 recognition motifs to the C-end is represented in gray. BIK1, Botrytis-induced kinase 1; BAK1, BRASSINOSTEROID INSENSITIVE 1-associated receptor kinase 1; BR1, Protein BRASSINOSTEROID INSENSITIVE 1; PK, Protein kinase superfamily protein; BAC, RAC-alpha serine/threonine-protein kinase. SMTI ID: Swiss-Model accession number.

To gain further insight into the conservation of the GDK and SEMPH motifs, we obtained models for the structure of PBS1 orthologs from Arabidopsis, *T. aestivum*, *Glycine max*, *Spirodela polyrhiza*, *P. patens*, *C. reinhardtii*, *Chara braunii*, *Pinus pinaster*, and *Lindsaea linearis*. These were selected because their function is known (AthPBS1 and TaPBS1), or harbor motifs with more similar to the former. Additionally, orthologs from diverse taxa were selected. According to these models, its structure consists mostly of alpha helices, and, less abundantly, beta sheet structures (Figure 10).

The residues corresponding to the GDK motif in AthPBS1, TaPBS1, GmaPBS1, SpipoPBS1-like, PpPBS1-like, and PpiHP were located within a loop (Figure 10A–E,H, green loop). Interestingly, ChbHP contains the motif GGETHV within the loop (Figure 10G, green loop). Moreover, no cleavage site motif was found in the *C. reinhardtii* and *L. linearis* PBS1 (Figure 10F,I). The residues LGAPSG (*C. reinhardtii*) and AERIL (*L. linearis*) were in the same position as the predicted cleavage site motif in other PBS1 orthologs.

Additional resistance recognition motifs, such as SEMPH, STRPH, STQPQ, ATRPH and NNRAA in AthPBS1, TaPBS1, GmaPBS1, ChbHP, and PpiHP, respectively, were identified. According to the predicted structures, these sequences are exposed in a loop (Figure 10A–C,G,H). Interestingly, in Arabidopsis, *T. aestivum*, *G. max*, and *Ch. braunii*, the analyzed motifs are close to one another. Furthermore, in SpipoPBS1-like, PpPBS1-like, and CrePKU-box from *S. polyrhiza*, *P. patens*, and *C. reinhardtii*, respectively, resistance recognition motifs were found, which is in agreement with previous sequence analysis. In contrast, in the same position where the resistance recognition motif is present in other PBS1 proteins, the residues NARAA, NSRAA, and QVVSV (which form an alpha helix) were found instead (Figure 10D–F). However, in *P. pinaster*, the NNRAA motif was located within a loop (Figure 10H).

## 3. Discussion

**Figure 11:**
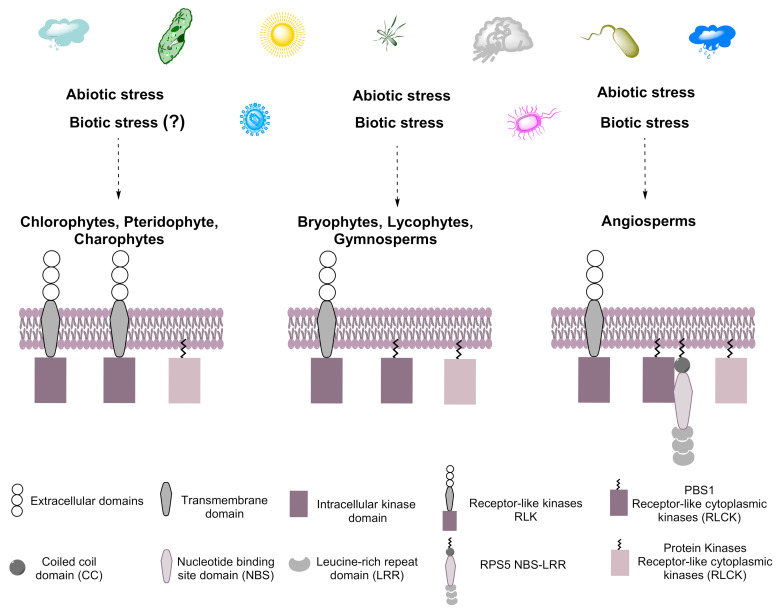
Hypothetical model for the evolution of PBS1. Plants are exposed constantly to biotic and abiotic stress, resulting in the evolution of systems that cope with such stress in streptophytes and chlorophytes. We suggest that PBS1 evolved from RLKs containing transmembrane domains in ancestral embryophytes and are related to those present in chlorophytes that would later become part of the plant immune system. The arsenal of microbial effectors is recognized by various response proteins, such as receptor-type kinases, among them PBS1, which participates in the defense response by activating RPS5 in angiosperms. Dotted arrows indicate towards which species the biotic and abiotic stresses will be caused. Question mark (?) indicates it is not known whether that biotic stress induces this response.

RLKs are present in all Viridiplantae (Streptophytes and Chlorophytes), share several typical kinase subdomains, and are induced by abiotic and biotic stress [23,24]. The proteins involved in the defense response tend to be more diverse than those involved in growth and development [25]. RLCKs, a subgroup of RLKs, belong to the RLK/Pelle family and are clustered in the superfamily of eukaryotic serine/threonine/tyrosine kinases [8]. RLCKs, and PBS1 in particular, evolved from RLKs by the deletion of an extracellular domain [8]. The divergence of these genes could have resulted in the division of labor, with both types of proteins responding to different and partially overlapping sets of stimuli. Indeed, RLKs have a lower biotic stress responsiveness compared to RLCKs [26]. The expansion of the RLCK genes was followed by divergence from ancestral terrestrial plants [26] (Figure 11). Indeed, PBS1 and PBS1-like proteins, such as BIK1 and PBL1, play important roles in signaling from the cell surface immune receptors, but may not play an essential function in PTI signaling, becoming a decoy by mimicking true virulence targets to trigger ETI [19,27]. According to previous reports, PBS1 has a kinase activity (autophosphorylation) that activates HR mediated by RPS5 in Arabidopsis, but also PBS1 from *N. benthamiana*, and can phosphorylate NbREM4 (remorin protein) in vitro, allowing the interaction with HopZ1a (host cell plasma membrane localized effector protein) triggering the ZAR1-dependent HR in *N. benthamiana* [28,29]. Arabidopsis PBS1 is grouped within a so-called universal stress response transcriptome because it includes genes that are induced by diverse conditions, such as osmotic shock, cold, salinity, wounding, and biotic stress, indicating that PBS1 responds to both abiotic and biotic stress [30]. Less is known regarding the phylogeny and possible origin of PBS1 and other RLCKs.

PBS1 proteins are characterized by the presence of GDK and SEMPH motifs in Arabidopsis [31]; however, these are absent in chlorophytes and pteridophytes as well as in most terrestrial plants. Notably, the GDK motif (and the GDKSHV and GDKTHV extended motifs) is present mostly in dicots and monocots, as well as in some gymnosperms, lycophytes, and bryophytes, suggesting that this motif (and thus, potentially the decoy mechanism) arose in the common ancestor of Streptophyta. However, the resistance recognition motif (RRM) is highly diverse (Supplementary Table S1). The sequence of this motif is SEMPH in Arabidopsis PBS1 and other closely related orthologs; however, the present study showed that the STRPH motif is more widespread in embryophytes. Extant evidence indicates that a negatively charged amino acid residue (glutamate in the SEMPH motif) is required for the recognition of PBS1 by RPS5 [9]. However, when PBS1 harbors the STRPH motif, RPS5 binding is weakened but is still functional [11]. The predicted tridimensional structure of PBS1 suggests the importance of this residue for PBS1–RPS5 interaction [9]. However, the “decoy” function of PBS1 has been reported only for Arabidopsis RPS5 and the *Hordeum vulgare* PBR1 [18]. Nevertheless, in the latter case, phylogenetic analysis of PBR1 indicates that it is not closely related to RPS5. It is thus possible that PBS1 proteins that harbor a non-canonical RRM motif interact with a wide array of NLR proteins. Given that RLCKs can also mediate responses to abiotic stress, other factors could interact with PBS1-like proteins from diverse taxa.

It is conceivable that during evolution, there were no constraints to the diversification of the RRM; indeed, the canonical SEMPH motif is present only in Arabidopsis, *Capsella rubella*, *Arabidopsis lyrata*, *Eutrema salsugineum*, *Camelina sativa*, *Microthlaspi erraticum*, *Arabis nemorensis*, *Brassica campestris*, and *Brassica oleracea capitata* clustered in one clade. It must be noted that all these are closely related species. PBS1 orthologs in terrestrial plants harbor similar conserved kinase subdomains and GDK motif, but the RRM motif may allow for the interaction with different NLRs. This is supported by the phylogenetic analysis presented herein.

RLCKs evolved to recognize microbial effectors only in some taxa. Accordingly, some species harbor PBS1-like sequences. Furthermore, some of these are found in clades that include evolutionary divergent taxa, supporting the notion of uneven substitution rates in these, even though PBS1 is one of the most highly conserved proteins in angiosperms [21]. In chlorophytes, AthPBS1 is more closely related to a tyrosine kinase from *C. subellipsoidea* and two hypothetical proteins from *C. eustigma* and *Trebouxia sp.*, respectively. Interestingly, the GDK motif was absent in these PBS1 orthologs. Furthermore, PBS1 orthologs from the analyzed chlorophytes and streptophyte share conserved kinase subdomains, suggesting a common ancestor for these sequences. Green algae (i.e., Charales, Zygnematales and Coleochaetales) harbor serine/threonine-protein kinases as well as mitogen activated protein kinase kinase kinase 3 (MAPKKK3), and interleukin-1 receptor-associated kinase 4 (IRAK4), all belonging to the RLK family. This suggests that the expansion of these genes has allowed an accelerated evolution in the domains involved in signal reception in these kinases [15,32].

The phylogeny of PBS1-like sequences identified two large clusters that contain the GDK motif. One cluster contains diverse kinases and hypothetical proteins closely related to PBS1 from various streptophytes and charophytes (angiosperms, bryophytes, gymnosperms, lycophytes, and the charophyte alga *N. axillaris*). The second cluster consists of diverse streptophytes, but harboring kinases more distantly related to PBS1, such as CDL1-like serine/threonine-protein kinases, LRR receptor-like protein kinases, STRUBBELIG-RECEPTOR FAMILY 8, protein kinase domain-containing, hypothetical proteins, and brassinosteroid-like receptor proteins. The STRUBBELIG protein is involved in signaling during tissue morphogenesis [25], and the CDL1-like serine/threonine-protein kinase positively regulates brassinosteroid signaling and growth. This suggests in some cases the convergent evolution of this protein. Another, smaller clade that includes kinases lacking a GDK, such as phototropin, a putative LOV domain-containing protein, and neochrome proteins, was identified in pteridophytes. These are related to blue-light photoreceptors (which, unlike RLCKs, display a transmembrane domain), important in the regulation of the circadian clock and photoperiodic flowering [33,34]. Therefore, PBS1 and its related proteins have been co-opted during evolution to serve diverse functions, ranging from immune signaling to developmental and growth responses to light [35]. Moreover, members of the same lineage according to extant taxonomic evidence lie in different clades, suggesting that the presence of unrelated species in common clades might be attributed to the characteristics of the analyzed sequences due to the selective pressure generated by the environment.

According to the present analysis, PBS1-like proteins arose prior to the divergence of embryophytes from streptophyte algae. The fact that sequences similar to PBS1 in the latter also lack these signature motifs supports this notion. Thus, PBS1 evolved from preexisting kinases in ancestral lineages. To learn more about the evolution of PBS1 proteins, an ancestral analysis of PBS1 orthologs in terrestrial plants was carried out.

Ancestral reconstruction is a powerful tool to gain knowledge on the evolutionary relationships of modern taxa to their potential ancestors, as well as the evolutionary process involved [36,37], based on a comparison of extant homologous proteins to infer the sequence of precursor proteins in ancestral species [38]. The ancestral reconstruction of PBS1 orthologs was analyzed with its amino acid sequences (ancestral character states). First, the ancestral tree showed five main ancestral nodes, indicating a possible origin from pteridophytes. The next ancestral node originates a gymnosperm (*Pinus taeda*) corresponding to seed-producing plants, such as pteridophytes. Interestingly, the next ancestral node included a charophyte (*N. axillaris*). Finally, the ancestral black node diverged into different terrestrial plant species, including charophytes, bryophytes, lycophytes, gymnosperms, and angiosperms; dicots diverged from the last ancestral node. Therefore, we propose that the PBS1 ancestor harbored both GDK and SEMPH motifs. However, these motifs are not conserved in pterydophytes, *P. taeda*, or *N. axillaris*. An important species that protrudes from the fourth ancestral node is *Amborella trichopoda*. This plant is considered a single sister species to flowering plants [39]. Interestingly, another branch from the same ancestral node corresponds to *Ch. braunii*. The PBS1 ortholog of this chlorophyte corresponds to a hypothetical protein that contains putative modified motifs. During evolution, the GDK motif showed minimal changes in amino acid residues, likely a reflection of selective pressure that maintained its function. The GDKSHV and GDKTHV extended motifs are the most conserved in PBS1, in which serine and threonine are interchangeable. As mentioned earlier, the SEMPH motif showed a low degree of conservation. The amino acids that are likely more relevant for interaction with RPS5 are glutamate (in position 297) and methionine (in position 298). According to the ancestral state, threonine is the most conserved ancestral character in position 297, originating from *N. axillaris*. In the case of position 298, the most conserved ancestral character is arginine. Its divergence in some species to a hydrophobic amino acid suggests a different function of these motifs [9,40]. PBS1 sequences containing TDMPH, SDRPH, and GDRPH motifs were identified in dicots; in these, an aspartate residue substitutes the similarly negatively charged glutamate residue, suggesting similar functions of these motifs [11]. In summary, although the more conserved motifs are GDKSHV and STRPH, some PBS1 proteins have amino acid residues with different or similar biochemical properties, which may have implications in the specificity of the decoy function of PBS1.

Structure modeling of PBS1 proteins from nine different species allowed a closer analysis of the motifs relevant for its function. The GDK motif is located on an exposed loop, which would be accessible for its cleavage. None of the *C. reinhardtii* and *L. linearis* PBS1 orthologs harbor a GDK motif; rather, the LGA and AER sequence are found in its position, respectively. The function of these proteins is unknown; however, based on their similarity to described kinases, they are likely involved in signal transduction in response to developmental and environmental signals. This also illustrates the diversity of functions of this protein family, the decoy function in ETI being a late evolutionary addition. In the PBS1 models analyzed in this study, STQPQ motifs are separate from the GDK motif and are exposed in a loop, suggesting that there is no steric hindrance for their recognition by RPS5. It is not known whether the STQPQ motif serves a similar function to SEMPH and STRPH, i.e., RPS5 binding. In the *S. polyrhiza*, *P. patens*, *L. linearis*, and *C. reinhardtii* PBS1 orthologs, a different sequence is found instead of the SEMPH motif (NARAA, NSRAA, SERAA, and QVVSV, respectively), which forms an alpha helix, and would thus be inaccessible to binding to a putative RPS5 homolog. Interestingly, the hypothetical *P. pinaster* protein contains the GDK motif and the motif NNRAA exposed in a loop. On the other hand, *Ch. braunii* presents GGETHV (potentially equivalent to the GDK and SEMPH motifs). The function of these motifs is unknown, but their mechanism of action could be different from PBS1, given the sequence dissimilarity. A study in which the AthPBS1 SEMPH motif was replaced by the *P. patens* PBS1 NSRAA motif results in a loss of HR in Arabidopsis, which shows that their functions are different [17]. The barley HvPBS1-1 and HvPBS1-2 are AthPBS1 orthologs and are cleaved by AvrPphB [21]. Indeed, a protein only distantly related to RPS5, PBR1, recognizes AvrPphB, lending support to the notion, first, that proteins that recognize microbial effectors have in several cases undergone convergent evolution; second, that different host proteins may recognize the same effector (and are under strong balancing selection in different genotypes); and finally, the interaction mechanism of PBR1 to PBS1 homologues differs from RPS5 [21].

Nucleotide-binding domain leucine-rich repeat (NBS-LRR) proteins, such as RPS5, are an important category of immune receptors [41]. Comparative genomic analysis of plant species suggests that NBS-LRRs are present in charophytes [42], terrestrial plants, and 2 out of 24 chlorophyte species [43]. The origin of NBS-LRRs can be traced to a common ancestor before the divergence of green algae and terrestrial plants [42,43]. 

These data suggest that PBS1 and RPS5 have evolved from ancestral species, acquiring motifs useful for defense response. In NBS-LRR proteins, including RPS5, gain and loss of domains, as well as gene duplication and translocation events have occurred frequently during evolution, resulting in high variability required for the recognition of rapidly evolving pathogens. RPS5 is thus a highly polymorphic protein, suggesting that balancing selection has an important role in its evolution [1,42,44,45].

In conclusion, we identified PBS1 ortholog protein sets in angiosperms, bryophytes, gymnosperms, liverwort, lycophytes, pteridophytes, charophytes, and chlorophytes. The bioinformatics analyses on PBS1 indicate the presence of GDK motif in ancestral species, such as bryophytes, charophytes, liverwort, and lycophytes, but not in chlorophytes and pteridophytes, suggesting that PBS1 might have arisen in evolution from a sister group of these ancestral species, but this ancestral PBS1 possibly had a function unrelated to the defense response. It may have acquired the SEMPH motif subsequently in dicots, and given rise to PBS1 involved in the response to *P. syringae*; RPS5 is within a group of resistance proteins that, as PBS1, have evolved from ancestral species through confrontation with various pathogens to trigger ETI.

## 4. Materials and Methods

### 4.1. Bioinformatic Identification of PBS1 Proteins in Plant Species

The sequences of Arabidopsis PBS1 (ID: Q9FE20) were used to identify homologous proteins in the UNIPROT (https://www.uniprot.org/, accessed on 10 October 2020), OrthoDB (https://www.orthodb.org/, accessed on 10 October 2020), and Phytozome 12 (https://phytozome.jgi.doe.gov/pz/portal.html#!search?show=BLAST, accessed on 10 October 2020) databases by the BLASTP tool. The amino acid sequences were filtered. First, redundant sequences were removed by the BlastClust tool using Biolinux (used the following command line: blastclust -i infile -o outfile -p T -L 1 -b T -S 100). Second, OrthoMCL was used for identification of ortholog proteins using homologous amino acid sequences of PBS1 at E-value Exponent Cutoff = 1 × 10^−5^ and percent Match Cutoff = 50 [46]. A total of 881 ortholog amino acids sequences of PBS1 were in a wide range of species, including monocot, eudicot, bryophyte, pteridophyte, gymnosperm, lycophytes, liverworts, charophytes, and chlorophyte, which were identified and used in the following analyses. The resulting 881 PBS1 sequences can be found in Supplementary Table S1 (to facilitate the search for sequences and species, a four-digit code from A001 to A881 was implemented).

### 4.2. Identification of Subdomains in PBS1 Orthologs

The sequences of PBS1 from different species were subjected to multiple sequence alignments (MSA), which were performed using ClustalW (https://www.genome.jp/tools-bin/clustalw, accessed on 12 December 2020) with default settings (pairwise alignment: Fast/Approximate: K-tuple (word) size: 1, windows size: 5, gap penalty: 3) to identify the conserved kinase subdomains, the GDK and SEMPH motifs are required for RPS5 recognition. Conserved regions obtained through multiple alignment analysis were analyzed using the MEME program (https://meme-suite.org/meme/index.html, accessed on 12 December 2020, ref. [47]) with the following settings: discriminative mode, primary sequence was PBS1 (ID: Q9FE20) of Arabidopsis, any number of repetitions, and 10 motifs.

### 4.3. Phylogenetic Analysis of PBS1

The evolutionary relationships among PBS1 proteins were investigated using a total of 881 PBS1 ortholog amino acidic sequences from PBS1 corresponding to terrestrial plants and chlorophyte species. Out of a total of 881 sequences, 182 PBS1 ortholog sequences were selected on the basis of higher homology scores within each genus and species of terrestrial plants. In the case of chlorophytes, 116 PBS1 orthologs sequences were used to construct the phylogeny. Multiple alignments were performed using the muscle algorithm, implemented in Seaview, and the analysis was edited using Aliview; the regions showing gaps were eliminated.

Phylogenetic trees were produced using maximum likelihood from multiple alignments using the IQ-TREE program (http://www.iqtree.org/, accessed on 5 January 2020) with parameters -m TESTNEWONLY -b 1000 and with bootstrapping resampled 1000 times with the replacement of the best model evolution according the Corrected Akaike Information Criterion VT + F + R9 (transversion model, AG = CT and unequal base frequency, and empirical base frequencies; this is the default if the model has unequal base frequency). This method results in the tree (s) most likely to fit the data. In the IQ-TREE case, the model section performs it automatically. The phylogenies were visualized using the Figtree program and were rooted by the midpoint.

### 4.4. Ancestral State Reconstruction of PBS1 Orthologs

For the ancestral PBS1 reconstructions, 182 PBS1 ortholog amino acid sequences from terrestrial plant species were used. Phylogenetic trees were produced using Bayesian statistics (https://nbisweden.github.io/MrBayes/download.html, accessed on 6 February 2021). For this tree, 5 million generations were used to build the phylogeny, with parameters chains = 4, printfreq = 100, samplefreq = 100, and burnin = 200. The tree was visualized in Mesquite (https://www.mesquiteproject.org/, accessed on 6 February 2021), and the reconstruction of ancestral characters was carried out using the maximum parsimony algorithm.

### 4.5. PBS1 Structure Modeling

To generate a model for the PBS1 structure, the following protein orthologs were selected: A003—Arabidopsis, A635—*T. aestivum*, A654—*G. max*, A179—*S. polyrhiza,* A352—*P. patens*, and A773—*C. reinhardtii*. Structural modeling was performed using Swiss Model (https://swissmodel.expasy.org/interactive, accessed on 15 February 2021) visualized and manipulated using Pymol software (http://pymol.sourceforge.net/newman/user/S0200start.html, accessed on 15 March 2021). The 3D structures of PBS1 homologs from the species mentioned above were analyzed using Swiss Model (https://swissmodel.expasy.org/, accessed on 15 February 2021). These known structures were the serine/threonine-protein kinase named Botrytis-induced kinase 1 (BIK1) for A003, A635, and A654 species; BRASSINOSTEROID INSENSITIVE 1-associated receptor kinase 1 (BAK1) for A179; protein BRASSINOSTEROID INSENSITIVE 1 (BR1) for A352; and protein kinase (PK) for A773. Swiss Model accession numbers for the known structures were as follows: 5tos.1 (A003, a635 and A654), 3tl8.1 (A179), 5lpz.1. A (A352), and 6j5t.1 (A773).

## Data Availability

Data is contained within the article or supplementary material.

## Supplementary Materials

The following are available online at https://www.mdpi.com/article/10.3390/ijms22136819/s1.
