# Supplementary material for: An Ancestry Perspective of the Evolution of PBS1 Proteins in Plants
Source: Int J Mol Sci. 2021 Jun 25;22(13):6819. doi: 10.3390/ijms22136819 (PMC8269361; doi:10.3390/ijms22136819)
Supplement: Supplementary file 1 [file ijms-22-06819-s001.zip › Supplementary File 1.pdf]

Supplementary File 1. Orthologs amino acid sequences of PBS1. 881 PBS1 orthologs sequences used in this study.

>A001|LOC103943624

MGGCFPCFGSSKNKEGSGGGGGGGGGGVKEVTRKDSSIKEGSAAQSHRVTRVSSDKKSRNGSDPKKEPPIPKD
GSTAHIAAQTFTFRELAAATKNFRPECLLGEGGFGHVYKGRLESTGQVVAVKQLDRNGLQGNREFLVEVLMLSL
LHHPNLVNLIGYCADGDQRLLVYEFMPLGSLEDHLHDLPSDKEPLDWNTRMKIAAGAAKGLEYLHDKANPPVIY
RDLKSSNILLDEGFHPKLSDFGLAKLGPVGDKTHVSTRVMGTYGYCAPEYAMTGQLTLKSDVYSFGVVFLELITGR
KAIDNDRGPGEHNLVAWARPLFKDRRKFPKMADPLLQGRYPMRGLYQALAVAAMCLQEQAATRPLIGDVVTA
LTYLASQTYDPNAATTPSNRGGSSTPRHRDERRNMGDGLDSPDEYVRGGRHGSPATHKNSPDFRKRDPNRDL
NTGVELGRIETGTGSGRRWGLDGLERQESQRDSPQSAGRARETPRNRDLDRERAVAEAKVWGENWREKKRA
NAMGSFDGTNE

>A002|A0A2P5XSL9

MGCFSCFDSKEEEKLNTVNESNDPKQAQPTVPSNISRLSSGGDRLRSRSNGGSKRELPCPRDGPGVQIAAHIFSF
RELAAATKNFRPESFLGEGGFGHVYKGQLESTGQVVAVKQLDRNGLQGNREFLVEVLMLSLLHHHNLVNLIGYC
ADGDQRLLVYEFMPLGSLEDHLHEIKIAGSGKLDYSMIFVNLFADLPPGKEPLDWNTRMKIAAGAAKGLEYLHD
KANPPVIYRDFKSSNILLDEGFHPKLSDFGLAKLGPVGDKSHVSTRVMGTYGYCAPEYAMTGQLTVKSDVYSFGV
VFLELITGRKAIDSTRPHGEQNLITWARPLFTNRRKLSKLADPLLQGRFPMRGLYQALAVASMCIQEEAAARPHI
GDVVTALSYLANQAYEPNASGHGHSGSGETDEKRYRDDRGGRVSKNDEGGVSGCRWDLEGSEKDDSPKESAR
MLNRDLDRERAVAEAKMWGENWREKRRQSAQGSSDGSNG

>A003|Q9FE20

MGCFSCFDSSDDEKLNPVDESNHGQKKQSQPTVSNNISGLPSGGEKLSSKTNGGSKRELLLPRDGLGQIAAHTF
AFRELAAATMNFHPDTFLGEGGFGRVYKGRLDSTGQVVAVKQLDRNGLQGNREFLVEVLMLSLLHHPNLVNLI
GYCADGDQRLLVYEFMPLGSLEDHLHDLPPDKEALDWNMRMKIAAGAAKGLEFLHDKANPPVIYRDFKSSNILL
DEGFHPKLSDFGLAKLGPTGDKSHVSTRVMGTYGYCAPEYAMTGQLTVKSDVYSFGVVFLELITGRKAIDSEMP
HGEQNLVAWARPLFNDRRKFIKLADPRLKGRFPTRALYQALAVASMCIQEQAATRPLIADVVTALSYLANQAYD
PSKDDSRRNRDERGARLITRNDDGGGSGSKFDLEGSEKEDSPRETARILNRDINRERAVAEAKMWGESLREKRR
QSEQGTSESNSTG

>A004|A0A0E0DQR2

MGCFPCFGSGGKGEAKKGGGGGRKDGGSADRRVARVGSGGCIQWLPKSKLEGGKAFLAVGTSSVAAEFLINGI
SLGLGEGGLESRKDAFIPRDANGQPIAAHTFTFRELAAATKNFRQDCLLGEGGFGRVYKGHLENGQAVAVKQLD
RNGLQGNREFLVEVLMLSLLHHDNLVNLIGYCADGDQRLLVYEFMPLGSLEDHLHDIPPDKEPLDWNTRMKIA
AGAAKGLEFLHDKANPPVIYRDFKSSNILLGEGYHPKLSDFGLAKLGPVGDKTHVSTRVMGTYGYCAPEYAMTG
QLTVKSDVYSFGVVFLELITGRKAIDNTKPLGEQNLVAWARPLFKDRRKFPKMADPLLAGRFPMRGLYQALAVA
AMCLQEQAATRPFIGDVVTALSYLASQTYDPNAPVQHSRSNASTPRARNRVGGNFDQRRLHSPTHQQSPDLRK
EGTTTSKYEAEVSRTNSGSGSGRRSGLDSMDVTGSQMGSPAHAGRKKESSRSTDRQRAVAEAKTWGENSRER
KWPNARGSFDSTNE

>A005|A0A1U7ZZU5

MGWFPCSGKSNKKSKKKKPDDQQIQSTSDLGRNDLGIFFEQKSWFDGFYTSKKLKVNPSLDVKKESSKDGSDRI
AAQTFTFRELAAATKNFRADCLLGEGGFGRVYKGRLESINQIVAIKQLDRNGLQGNREFLVEVLMLSLLHHPNLV
NLIGYCADGDQRLLVYEYMPLGSLEDHLHDLPPDKKRLDWNTRMKIAAGAAKGLEYLHDKANPPVIYRDLKCSN
ILLGEGYHPKLSDFGLAKLGPVGDKTHVSTRVMGTYGYCAPEYAMTGQLTLKSDVYSFGVVLLEIITGRKAIDNSK
AAGEHNLVAWARPLFKDRRKFSQMADPMLQGQYPVRGLYQALAVAAMCVQEQPTMRPLIADVVTALTYLAS
QTYDPDTQPVQSSRMYPSTPPRTKRDGDKRLNGGNGYERDQGRGLK

>A006|A0A0Q3PRH2

LQKPRKPSPPPPPPPPPFVPWWRVFFPGGGARMSCFVCFGSAQDGEAKKQVADAKDPRKDGPPDRGVARVGS
DKSRSQGGSDSKDIIIHRDGNSQNIAAQTFTFRELAAATKNFRQDCLLGEGGFGRVYKGRLENGQAVAVKQLDR
NGLQGNREFLVEVLMLSLLHHTNLVNLIGYCADGDQRLLVYEFMPLGSLEDHLHDLPKDKEALDWNTRMKIAA
GAAKGLEYLHDKASPPVIYRDFKSSNILLGEGFHPKLSDFGLAKLGPVGDKTHVSTRVMGTYGYCAPEYAMTGQL
TVKSDVYSFGVVFLELITGRKAIDNSKPQGEQNLVAWARPLFKDRRKFPKMADPKLQGRFPMRGLYQALAVAA
MCLQEQAATRPHIGDVVTALSYLASQAYDPNAPVQHSRSNSSTPRARNPAGWNDDQRSVRSPNHHSPDLRRR
DAARASKYGAEVSRTSSTGGSGRRSGLDEMDMTGSQMGSPAQTGRRRETPRAAADRQRAIAEAKMWGEYSR
ERSNGHGSFDSTNE

>A007|M4CD91

MSGCLPCFGSSAKDAASKDSVKKEASAKAKDASLTQSHHVSLDKSKSKGGSEQKKELTAPKEGPTAHIAAQTFTF
RELAAATKNFRPDCLLGEGGFGRVYKGRLETTGQIVAVKQLDRNGLQGNREFLVEVLMLSLLHHTNLVNLIGYCA
DGDQRLLVYEYMPLGSLEDHLHDLPPDKEPLDWNTRMTIAAGAAKGLEYLHDKANPPVIYRDLKSSNILLGDGY
HPKLSDFGLAKLGPVGDKTHVSTRVMGTYGYCAPEYAMTGQLTLKSDVYSFGVVFLELITGRKAIDNARAHGEH
NLVAWARPLFKDRRKFPKMADPSLQGRYPMRGLYQALAVAAMCLQEQAATRPLIGDVVTALTYLASQTFDPN
AASSQNSRSGGGGPPFIRTRDERRSMGDGSSLDSPAETRSRLGSPATHKNSPDYRRRDMVREVNAGSEAGSEN
GGGSGRKWGLSDVEGTESQRGSPASVGRGTRGTPRNRDLDRERAVAEAKVWGENWRERKRGEGREGSYETF
VESGRVGQLSDLMSESEERLFNIVVEAQEHDANDERLGN

>A008|A0A446L2W9

MQRGSKAMANRWGLIQSACNKWHGIVEKIAARPESGASVEDQGGGGGGNGTGGRTVSAASSSGVGAREER
PMVPPRVEKLPAGAEKARAKGNAGMKELSDLRDANGNVLSAQTFTFRQLTAATRNFREECFIGEGGFGRVYKG
RLDGGQVVAIKQLNRDGNQGNKEFLVEVLMLSLLHHQNLVNLVGYCADGEQRLLVYEYMPLGSLEDHLHDLPP
DKEPLDWNTRMKIAAGAAKGLEYLHDKAQPPVIYRDFKSSNILLGDDFHPKLSDFGLAKLGPVGDKSHVSTRVM
GTYGYCAPEYAMTGQLTVKSDVYSFGVVLLELITGRKAIDSTRPHGEQNLVSWARPLFNDRRKLPKMADPGLQG
RYPMRGLYQALAVASMCIQSEAASRPLIADVVTALSYLASQIYDPNAIHASKKAGGDQRSRVSDSGRTLLKNDEA
GSSGHKSDRDDSPREPPPGILNDRERMVAEAKMWGANLREKTRAAANAQGSLDSPTEIG

>A009|A0A0E0NFF1

MGCFSCFDSPAEEQLNPKVGGPYGGGSSSSAAAAARAAPHRPRLRSRRCGGSSAGRHGERGGGYPDLHHHHQ
QQQLPMAAPRVEKLSAGAEKTRVKSNAILREPSAPKDANGNVISAQTFTFRELATATRNFRPECFLGEGGFGRV
YKGRLESTGQVVAIKQLNRDGLQGNREFLVEVLMLSLLHHQNLVNLIGYCADGDQRLLVYEYMHFGSLEDHLHD
LPPDKEALDWNTRMKIAAGAAKGLEYLHDKANPPVIYRDFKSSNILLDESFHPKLSDFGLAKLGPVGDKSHVSTR
VMGTYGYCAPEYAMTGQLTVKSDVYSFGVVLLELITGRRAIDSTRPHGEQNLVSWARPLFNDRRKLPKMADPR
LEGRYPMRGLYQALAVASMCIQSEAASRPLIADVVTALSYLASQSYDPNAAHASRKPGGDQRSKVGENGRVVS

RNDEASSSGHKSPNKDREDSPKEPPGILNKDFDRERMVAEAKMWGDRERMVAEAKMWGDRERMVAEAKM
WGENWRDKRRAIENGQGSLDSPTENG

>A010|100814575

MGGCFPCFGSSNKEGSGGVRVKEVPNRDSSFKEAAASVVPQSHHPSRVNSDKSKSRSGADTKKETPVPKDGPT
AHIAAQTFTFRELAAATKNFRPECLLGEGGFGRVYKGRLETTGQVVAVKQLDRNGLQGNREFLVEVLMLSLLHH
PNLVNLIGYCADGDQRLLVYEFMPLGSLEDHLHDLPPDKEPLDWNTRMKIAAGAAKGLEYLHDKANPPVIYRDL
KSSNILLDEGYHPKLSDFGLAKLGPVGDKTHVSTRVMGTYGYCAPEYAMTGQLTLKSDVYSFGVVFLELITGRKAI
DNTRAHGEHNLVAWARPLFKDRRKFPKMADPLLQGRYPMRGLYQALAVAAMCLQEQAATRPLIGDVVTALTY
LASQTYDPNAANQSNRVGPSTPRSRDDRRSMADSVDSPDHGRLGSPSTHRNSPDFRKRDSRDPSTATELGRID
TGGGSGRKWGLDDYERQDSQRDSPVNTARARETPWNRDLDRERAVAEAKVWGENWREKKKANAVGSFDAT
ND

>A011|101294724

MGCFPCFDSKEEEKLNPEKESDDRKQGQPTVSSNISRLPSGVDRLRSRSNGGSRRVDVGSKMPDPKDVVPGGQ
IAAQTFTFRELATATKNFRPESFIGEGGFGRVYKGRLESTGQIVAVKQLDRNGLQGNREFLVEVLMLSLLHHTNLV
NLIGYCADGDQRLLVYEFMPLGSLEDHLHDVPLDKEVLDWNTRMKIAAGAAKGLEYLHDKANPPVIYRDFKSSN
ILLEEGFHPKLSDFGLAKLGPTGDKSHVSTRVMGTYGYCAPEYAMTGQLTVKSDVYSFGVVFLELITGRKAIDSTR
SHGEQNLVTWARPLFNDRRKFSKLADPRLQGRYPMRGLYQALAVASMCIQEQAATRPLIGDVVTALSYLANQA
YDPTAASGHGHRSSGEKDERRRDERGGRILKNEEGGGSGRKWPDLDGGSEKDDSPKETARMLNRDLDRERAV
AEAKMWGENWREKRRQSAQGSFDGTNL

>A012|102595931

MGCFSCFDSKEDEKLNPQKDRDDSNRKQPHLTAPSNISRLSSGADRLKTRSTNCSKREFLGIKDAPDVQIAAHTF
TFRELAAATNNFKPESFIGEGGFGRVYKGQLPSGQVVAVKQLDRNGLQGNREFLVEVLMLSLLHHPNLVSLIGYC
ADGDQRLLVYEFMPLGSLEDHLHDLPPDKEPLDWNTRMTIASGAAKGLEHLHDKANPPVIYRDFKSSNILLKDN
FFPKLSDFGLAKLGPTGDKSHVSTRVMGTYGYCAPEYAMTGQLTVKSDVYSFGVVFLELITGRKAIDSTKPQGEQ
NLVAWARPLFNDRRKFAKLADPSLQGQFPMRGLYQALAVASMCIQEQAAGRPLIGDVVTALSYLANQSYDPGT
VPGQIHRFGADSVDRRNKDDRVGRIFRNEDGAGGGSGQKWDVDGGSEKEDSPRETARMLNRDLDRERAVAE
AKMWGENWRDKRRQNGQGSFDGGNE

>A013|Peaxi162Scf00386g00811.1

MGGCFPCFGSSNKETGNGVKEVVKKESFKDGSVAAQSHHLSKVSSDKSKSRGTHDPKKDPPLPKDGPTAHIAA
QTFTFRELAAATKNFRPECLLGEGGFGRVYKGRLESTGQVVAVKQLDRNGLQGNREFLVEVLMLSLLHHPNLVN
LIGYCADGDQRLLVYEFMPLGSLEDHLHDLPPDKEPLDWNTRMKIAAGAAKGLEYLHDKANPPVIYRDLKSSNIL
LDEGYHPKLSDFGLAKLGPVGDKTHVSTRVMGTYGYCAPEYAMTGQLTLKSDVYSFGVVFLELITGRKAIDNARA
HGEHNLVAWARPLFKDRRKFPKMADPLLQGRYPMRGLYQALAVAAMCLQEQASTRPLIGDVVTALSYLASQT
YDPNAAAAQSNRVGSSTPRSREDRLHSVDGVDSPDEHGGSAHHGSPSFQRNSPDSRKRDSARDFNTGIELRKIA
TSGGSGRKWGLDESERPDSQRDSPASAGRARETPRNRDLDRERAVAEAKVWGENWRDKKKTNARGSSFDGM
ND

>A014|PHAVU007G000800g

MGCFSCFDSREDEKLNPNPHQENHQHHLHHQHHHHDHDLNPPIPSRISRLPSAAAGADKLLSSTNGNANNGD
SKRELAILKDGNIAAQTFTFRELAAATKNFRPQSFLGEGGFGRVYKGRLETTGQAVAVKQLDRNGLQGNREFLVE
VLMLSLLHHPNLVSLIGYCADGDQRLLVYEFMPLGSLEDHLHDLPPDKEPLDWNTRMKIAAGAAKGLEYLHDKA
NPPVIYRDFKSSNILLDEGYHPKLSDFGLAKLGPVGDKSHVSTRVMGTYGYCAPEYAMTGQLTVKSDVYSFGVVF
LELITGRKAIDSTRPHGEQNLVTWARPLFNDRRKFPKLADPELHGRYPMRGLYQALAVASMCIQEQAAARPLIG
DVVTALSFLANQAYEHKGGGDDKRNRDDQGGRILKNEQGGGSGRRWDLEGSEKDESPRETARILNNRDLDRE
RAVAEAKMWGENWREKRRQSAQGSFDASNA

>A015|A0A287MGV2

MSCFSCFGPALEAEAGKPGPDAKDARGKDGAAPDRAGSDKLKLQGGSDPKNTHLTIPRDGNSQNIAAQIFTFRE
LAAATKNFRQDCMLGEGGFGRVYKGRLENGQAVAVKQLDRNGLQGNREFLVEVLMLSLLHHTNLVNLIGYCA
DGDQRLLVYEFMPLGSLEDHLHDVPPEKEPLDWNTRMKIAAGAAKGLEHLHDKASPPVIYRDFKSSNILLGEGF
HPKLSDFGLAKLGPVGDKTHVSTRVMGTYGYCAPEYAMTGQLTVKSDVYSFGVVFLELITGRKAIDNTKPQGEQ
NLVAWARPLFKDRRKFPKMADPMLQGRFPMRGLYQALAVAAMCLQEQATTRPHIGDVVTALSYLASQTYDP
NAPTQHTRSNSSTPRARNMGGRNSEQRNGRSPNHHSPRTSKHGGEVSRTSSNGGDSGRRSGLDDMDMAGS
QAGSPAQTGRKRDTPRTADRHRAVVDAKMWGENSRERKRPNGHGSFDSTNE

>A016|A0A0B0PYC3

MGGCFPCFGSSNKEANNNNNNGGTTVKELSKKDSTKDSSVPQSQHVNRDKSKSRNGSDSKRELPVVPKDGPM
ANIAAQTFTFRELAAATKNFRPECLLGEGGFGRVYKGRLESTGQVVAVKQLDRNGLQGNREFLVEVLMLSLLHH
PNLVNLIGYCADGDQRLLVYEFMPLGSLEDHLHDLPPDKEPLDWNTRMKIAAGAAKGLEYLHDKANPPVIYRDL
KSSNILLHEGFHPKLSDFGLAKLGPVGDKTHVSTRVMGTYGYCAPEYAMTGQLTLKSDVYSFGVVFLELITGRKAI
DNTRAHGEHNLVAWARPLFKDRRKFPKMADPLLQGHYPMRGLYQALAVAAMCLQEQAATRPLIGDVVTALT
YLASQTYDPNAPGNQSNRVGPSIPRPKEDGLDSPKEHSHRNSPDYRKRNHARESSTGEELGRNETSGGSGRKW
GLDDSERHESHRDNPLNTSRPRERNRDLDRERAVAEAKVWGENLREKKRANAMGSFDVKGRNTTKTGPRAVD
QFRGLTGDIEIHVGFVWDVSQLAFASFVMRQTS

>A017|A0A0R0IXX0

MSCFWYWVSLLLVEFGGEESNLEFHCKATFLCLGINKMGCFSCFDSSSKEDHNLRPQHQPNQPLPSQISRLPSG
ADKLRSRSNGGSKRELQQPPPTVQIAAQTFTFRELAAATKNFRPESFVGEGGFGRVYKGRLETTAQIVAVKQLDK
NGLQGNREFLVEVLMLSLLHHPNLVNLIGYCADGDQRLLVYEFMPLGSLEDHLHDLPPDKEPLDWNTRMKIAV
GAAKGLEYLHDKANPPVIYRDFKSSNILLDEGYHPKLSDFGLAKLGPVGDKSHVSTRVMGTYGYCAPEYAMTGQ
LTVKSDVYSFGVVFLELITGRKAIDSTQPQGEQNLVTWARPLFNDRRKFSKLADPRLQGRFPMRGLYQALAVAS
MCIQESAATRPLIGDVVTALSYLANQAYDPNGYRGSSDDKRNRDDKGGRISKNDEAGGSGRRWDLEGSEKDDS
PRETARILNRDLDRERAVAEAKMWGENLRQKRKQSLQQGSLDA

>A018|25485762

MGCFSCFDSKEDEKLNPNQHQHQETHHHHHDHNNISRLPSSGPSGGVEKLRSTSNGSSKRELQLQLQLPAALK
DGPPGQIAAQTFTFRELAAATKNFRPQSFLGEGGFGRVYKGRLETTGQAVAVKQLDRNGLQGNREFLVEVLML
SLLHSPNLVSLIGYCADGDQRLLVYEFMPLGSLEDHLHDLPADKEPLDWNTRMKIAAGAAKGLEYLHDKANPPVI
YRDFKSSNILLDEGYHPKLSDFGLAKLGPVGDKSHVSTRVMGTYGYCAPEYAMTGQLTVKSDVYSFGVVFLELIT
GRKAIDSTRPHGEQNLVTWARPLFNDRRKFSKLADPRLQGRYPMRGLYQALAVASMCIQEQAAARPLIGDVVT
ALSYLANQAHDTNNASIGNNKGTGDEKRNRDDKGGRILKNDEGGGSGRRWDLEGSEKDDSPRETARMLNRDL
DRERAVAEAKMWGENLREKRRQSVQGSFDASNA

>A019|A0A0E0D145

MGCFPCFDSRMGEEEEELYYGKRGADGGGNGGGALSAAAAAAASSSGVGGGGGGGWEGTSTAAPRVEKISA
GARGRGNATVKKELSALKDANSNVISAQTFTFRQLAAATRNFREECFIGEGGFGRVYKGRLDGTGQIVAIKQLNR
DGTQGNKEFLVEVLMLSLLHHQNLVNLVGYCADGDQRLLVYEYMPLGSLEDHLHDLPPDKVPLDWNTRMKIA
AGAAKGLEYLHDKAQPPVIYRDFKSSNILLGEDFHPKLSDFGLAKLGPVGDKSHVSTRVMGTYGYCAPEYAMTG
QLTVKSDVYSFGVVLLELITGRKAIDSTRPHVEQNLVSWARPLFNDRRKLPKMADPGLEGRYPMRGLYQALAVA
SMCIQSEAASRPLIADVVTALSYLASQKYDPNTTPSSKKAGGGGEAGRALSRNDEAGSSGHKSPSSKDSPREQLP
GILNDRERMVAEAKMWGENWREKRRAAATSSNAQGSLDSPTETG

>A020|A0A4V6D778

MGCFPCFGSGREDDELGYYGGNGGAAGWAAAAASSSAAAAAAAAVGGGAEAAVAAPPRAERSPTGSNKSKA
KGNGSKKELAVLKDANGNVISAQTFTFRQLAAATKNFRDECFIGEGGFGRVYKGRLDGTGQVVAIKQLNRDGN
QGNKEFLVEVLMLSLLHHQNLVNLVGYCADGDQRLLVYEYMPLGSLEDHLHDLPPGKEALDWNTRMKIAAGA
AKGLEYLHDKADPPVIYRDFKSSNILLGEGFHAKLSDFGLAKLGPVGDKSHVSTRVMGTYGYCAPEYAMTGQLTV
KSDVYSFGVVLLELITGRKAIDSTRPAAEQNLVSWARPLFNDRRKLPKMADPGLEGRFPTRGLYQALAVASMCIQ
SEAASRPLIADVVTALSYLANQIYDPNTASTSKKGGGDQRSRVSDSGRALSRNDDTGSSGHRSPSKDLDDSPRER
HATGTAHKGERERMVAEAKMWGENWREKQRAAQGSLDSPTGGG

>A021|A0A1S3CCG0

MGCFPCFDSREEEKLNPEKESDDGKQDHPMVPPNIAKLPSGIDKLRSKSNGGSKREQQIPTPLVNISAQTFTFRE
LATATKNFRPECFIGEGGFGRVYKGRLESTGQIVAVKQLDRNGLQGNREFLVEVLMLSLLHHPNLVNLIGYCADG
DQRLLVYEFMPLGSLEDHLHEIPPEKEPLDWNTRMKIAAGAARGLEYLHDKANPPVIYRDFKSSNILLDEGYHPKL
SDFGLAKLGPVGDKSHVSTRVMGTYGYCAPEYAMTGQLTVKSDVYSFGVVFLELITGRRAIDSTRPQGEQNLVT
WARPFFNDRRRFSKLADPQLQGRYPMRGLYQALAVASMCTQEQAAARPLIGDVVTALSYLANQSYDPSSPSGT
AAAAGTAAQRGSGDRDERRNRDEKGGGGMVKNEEGGASGRRWPLDGPEKDDSPRETARILNRDLDRERAVA
EAKMWGENWREKRRQSTQGSFDGSNG

>A022|A0A0L9TCZ6

MGCFSCFDSSSSSRENHNLRSHHHPQPNSNSNSNPNPNINLSLPSQISKLPSGADKLRSRSNGGSKRELPSSNTK
EGHGPAVQIAAQTFTFRELAAATKNFRPESFVGEGGFGRVYKGRLETTGQIVAVKQLDKNGLQGNREFLVEVLM
LSLLHHPNLVNLIGYCADGEQRLLVYEFLPLGSLEDHLHDVPPDKEPLDWNTRMKIAAGAAKGLEYLHDKANPP
VIYRDFKSSNILLDEAYHPKLSDFGLAKLGPVGDKSHVSTRVMGTYGYCAPEYAMTGQLTVKSDVYSFGVVFLELI
TGRKAIDSTQPHGEQNLVTWARPLFNDRRKFSKLADPRLQGRFPMRGLYQALAVASMCIQESAATRPLIGDVV
TALSYLANQGYDPNNAGHGYRGSSDDKRNRDDKGGRILKNDEAGGSGRRWDLEGSEKDDSPRETARMLNRD
LDRERAVAEAKMWGENLRQKRQQSLQEGSYGST

>A023|A0A124SD39

MGCFSCFDSRVEEKSNPQKVGADRPEVHPSAPSNISRLPSGVDRLKSRNNVSLRRESSGPKDGPYSQIAAHTFTF
RELAAATNNFSPDCFLGEGGFGHVYRGRLQGSGQAIAVKQLDRNGLQGNREFLVEVLMLSLLHHPNLVNLIGYC
ADGDQRLLVYEFMPLGSLEDHLHGNHFDLPPDREPLDWNTRMKIAAGAARGLEFLHDKANPPVIYRDFKSSNIL
LGEGFQPKLSDFGLAKLGPTGDKSHVSTRVMGTYGYCAPEYAMTGQLTVKSDVYSFGVVFLELITGRKAIDSTAP
QGQQNLVTWARPLFNDRRKFASLADPRLEGQYPMRGLYQALAVASMCIQEQAAARPLIGDVVTALSYLANHA

YDPSAAAATGHSNRHNNGEKNGRISKNEEGGGSGRSRWDLEGLEKGDSPRETPKMLNRDLDRERAVAEAKM
WVEKRRQSAQGSFDGNNNG

>A024|LOC111479363

MLVWLCNWVCSSGVSFFVVVFGCFHLIMGGCFPCFGSSDEDGNGVKDATKKDSAKDGSAAQSHHVTRVSSDK
SKSKSVSDAKKEPTVQKDGTTAHIAAQTFTFRELATATKNFRSECLLGEGGFGRVYKGRLESTGQVVAVKQLDRN
GLQGNREFLVEVLMLSLLHHPNLVNLIGYCADGDQRLLVYEFMPLGSLEDHLHDLPPDKEPLDWNIRMKIAAGA
AKGLEYLHDKANPPVIYRDLKSSNILLDEGYHPKLSDFGLAKLGPVGDKTHVSTRVMGTYGYCAPEYAMTGQLTL
KSDVYSFGVVFLELITGRKAIDNTRGPGEHNLVAWARPLFKDRRKFPKMADPLLQGRYPMRGLYQALAVAAMC
LQEQAATRPLIGDVVTALTYLASQAYDPNAAASQSNRMGGSTPRARDDRRGLSDGLDSPDERGRGRGSPSNYR
NSPDYRRKDLHRELSCGGTELNKIDTGGGSGRKWGLDELERQESLRDSPVYTGRGRETPRNRDLNRERAVAEAK
VWGENWRERKRANAQGSFDGSQE

>A025|A0A5N6MQM2

MGGCLSCFSSSNEQRNGVKEVSKKDNGKEGSVNHQSHHVSRVSSGRSKTHASLDHKKDQMATKDGQTANIA
AQTFTFRELAAATKNFRPESLLGEGGFGRVYKGRLESTGQVVAVKQLDRNGLQGNREFLVEVLMLSLLHHSNLV
NLIGYCADGDQRLLVYEFMPLGSLEDHLHDLPPDKEPLDWNTRMKIAAGAAKGLEYLHDKANPPVIYRDLKSSNI
LLDQGFHPKLSDFGLAKLGPVGDKTHVSTRVMGTYGYCAPEYAMTGQLTLKSDVYSFGVVFLELITGRKAIDNTR
APGEHNLVAWARPLFKDRRKFPKMADPLLQGRYPVRGLYQALAVAAMCLQEQAATRPLIGDVVTALTYLASQA
YDPEAARTERASRGSGSPTPRNRADRRKPSDAGLDNLDDSIREPRPHGSPSTYKNSPDHRRINSRSDAGDGESS
NGGGGSGRKWGLVDDLDSQKNSPANTGRARNRDLDRERAVAEAKVWGENWRDRKKTNTMGGGSSFDATK
V

>A026|A0A0K9Q987

MGGCFPCWESSNHAGNSDKEAANKDGGVKDSSIIQSHQLAKVNSDKSKFRNGSDPKKEPAVSKDGSTAHIAA
QTFTFRELAAATKNFRPESLLGEGGFGRVYKGRLESTGQVVAVKQLDRNGLQGNREFLVEVLMLSLLHHPNLVN
LIGYCADGDQRLLVYEYMPLGSLEDHLHDIPPGKEPLDWNTRMKIAAGAAKGLEYLHDKANPPVIYRDLKSSNIL
LDEGFHPKLSDFGLAKLGPVGDKTHVSTRVMGTYGYCAPEYAMTGQLTLKSDVYSFGVVFLELITGRKAIDNTRA
PGEHNLVAWARPLFKDRRKFPKMADPLLQGRYPMRGLYQALAVAAMCLQEQAATRPLIGDVVTALTYLASQT
YDPNAPSNQSSRGGSSTPRSRDDRRSYGDSLDSPDRRAARLSSPSTLRNSPDFRKRDHVRELSTGAELGRSEIGG
GSGRRWGLDDLERQESQRGSPASAGRTPRNRDIDRERAVAEAKVWGENWREKKRANAMGSFDSSNE

>A027|A0A2H3YBX8

MGCFPCFGSPNKEKEKESDVKGGGDFKKEAPAAPSTHHAASVSSDKLKSRSGSESKKEASMPKEGNSAHIAAQI
FTFRELAAATKNFRADCLVGEGGFGRVYKGRLENGQVVAVKQLDRNGLQGNREFLVEVLMLSLLHHPNLVNLIG
YCADGDQRLLVYEFMPLGSLEDHLHDIPPDKEPLDWNTRMKIAAGAAKGLEYLHDKANPPVIYRDFKSSNILLGE
GYHPKLSDFGLAKLGPVGDKTHVSTRVMGTYGYCAPEYAMTGQLTVKSDVYSFGVVFLELITGRKAIDNTRPDG
EQNLVAWARPLFRDRRKFPKMADPLLQGRYPMRGLYQALAVAAMCLQEQAATRPLIGDVVTALSYLASQTYD
PNAPAAQSNKVGPSTPRNRGDRKSLGCGTINQHALQSPQRNSPDFRQRDLIRGASYGADVGRGGVGGGSGRK
WSLEELETQESQRDSPVHVGKARESPKNVNRDLDRERAVAEAKVWGENWRERKQSYVQGGCDSTNE

>A028|LOC112282602

MGWFSCFTSAPKERKPQKNDDNNSSRDGQSAATTPVAQISNLPSGLAGNSKKYDAKGSFDKNEPQREGSTHIA
AQTFTFRELAAATKNFRPECLLGEGGFGRVYKGRLENTGQVVAVKQLDRNGLQGNREFLVEVLMLSLLHHPNLV

SLIGYCADGDQRLLVYEFMPLGCLEDHLHDLPQDKECLDWNTRMKIAAGAARGLEYLHDTAKPPVIYRDFKSSNI
LLDEGFHPKLSDFGLAKLGPVGDKTHVSTRVMGTYGYCAPEYAMTGQLTLKSDVYSFGVVLLELITGRKAIDNSR
SAGEHNLVAWARPLFKDRRKFPSMADPMLQGRYPMRGLYQALAVAAMCLQEQAATRPLIGDVVTALSYLASQ
IYDPGMHPLGSSRFAPATPSREKREKEKKMPPGPNTIEERMVKEQRAGKGGPRSPSVRSQAASPDLWVKEGGG
RARVTNGVLEESRGSESGSGRKRDSEEWERHEGGGRDSPGYAGRASRDGLKSQAPTRERERAVAEARVWGEN
WRERKRGAG

>A029|D8RRM2

MGCFACFGSSKKQASGRKGLRREDSAGSFNKTASGEYKLKSKGFFDAKKEGSRGDESKIAAQTFTFRELASATKN
FRPECMLGEGGFGRVYKGRLDSGQVVAVKQLDRNGLQGNREFLVEVLMLSLLHHPNLVNLIGYCADGDQRLLV
YEFMQLGSLEDHLHDVPADKEPLGWNTRMKIAAGAARGLEYLHDKANPPVIYRDFKSSNILLGEGHHPKLSDFG
LAKLGPVGDKTHVSTRVMGTYGYCAPEYAMTGQLTLKSDVYSFGVVLLELITGRKAIDNARPAGEHNLVAWAR
PLFKDRRKFPSMADPLLQGHYPMRGLYQALAVAAMCLQEQANTRPLIGDVVTALNYLASQTYDPGLYPANNSR
FAPSTPSRDRREREKKSSGTEERRLRDRGARSSLPSAQGTPELRSKDPRFRAANGGGGNSSDDLEGGHRDSPTH
AATRHAKAVATREQRDRAWGESNNNSRDRKKGSFGYDG

>A030|A0A1U7YUT8

MGGCLPCFGSPGKEKSGVKEGGKKDSVKEGSAAQSHHVSRVSSDKSKSRISSDSKRETPAAKDGQTTNIAAQTF
TFRELAAATKNFRQECLLGEGGFGRVYKGRLESTGQIVAVKQLDRNGLQGNREFLVEVLMLSLLHHPNLVNLIGY
CADGDQRLLVYEYMPLGSLEDHLHDLPPDKEPLDWNTRMKIAAGAAKGLEYLHDKANPPVIYRDFKSSNILLDE
GFRPKLSDFGLAKLGPVGDKTHVSTRVMGTYGYCAPEYAMTGQLTLKSDVYSFGVVFLELITGRKAIDNTRAPGE
HNLVAWARPLFKDRRKFPKMADPLLQGGYPMRGLYQALAVAAMCLQEQAATRPLIGDVVTALSYLASQSYDP
NTASTQSNRVGSSVLRDGDDKRNLNSGADGLDEVGCAGWHGLQSPHQNSPDFRQRDPARGMSFGAEVGRG
ETSGGSGRKWGLDELEKQESQRDSPVHAGRARETPRNYNRDFDRERAVAEAKVWGENWRERKRANTAAGSF
DGTNEPQ

>A031|LOC110785742
MGCFSCFESKEEERLNPSSGEKDDRKDAPPSSNSNISKLSSGADRVKARNNVGLRRESSISKDEPVANINSSAHTF
TFRELAAATKNFRDECFIGEGGFGRVYKGQLETNQVVAVKQLDRNGLQGNREFLVEVLMLSLLHHVNLVNLIGY
CADGDQRLLVYEYMALGSLEDHLFDFPPDKEPLDWNTRMKIAAGAAKGLEYLHDKANPPVIYRDFKSSNILLDE
GFHPKLSDFGLAKLGPTGDKSHVSTRVMGTYGYCAPEYAMTGQLTVKSDVYSFGVVFLEMITGRRSIDSNRPHG
EQNLVAWARPLFNDRRKFLKLADPQLQSRFPTRGLYQALAVASMCIQENAAARPLIGDVVTALSYLANQAYDPS
MKDRTSSRDENGGRITRGSEDGANSGRKWDLDGGSEKDDSPRETVRMLNKDSDRERAVAEAKMWGENWRE
KRRQTLDLEGGGGSSERDDSPKETARMLNRDSERERAVAEAKMWGENLREKRRQSAQGTNENSDG

>A032|100281460

MGCFPCFGSTREEELKYYGSKGPGGGNGGGGRATASSSSSAAAGGGGGRVEEAVVAPPRAQRGPAGADKTRA
KGNAGSKKELSVLRDASGNVISAQTFTFRQLAAATKNFRDECFIGEGGFGRVYKGRLDMGQVVAIKQLNRDGN
QGNKEFLVEVLMLSLLHHQNLVNLVGYCADGDQRLLVYEYMPLGSLEDHLHDLPPDKEPLDWNTRMKIAAGA
AKGLEYLHDKAQPPVIYRDFKSSNILLGEGFHPKLSDFGLAKLGPVGDKSHVSTRVMGTYGYCAPEYAMTGQLTV
KSDVYSFGVVLLELITGRKAIDSTRPASEQNLVSWARPLFNDRRKLPKMADPGLEGQFPTRGLYQALAVASMCIQ
SEAASRPLIADVVTALSYLANQIYDPSLAHASKKAGGSDQRNRVGDSGRALSKNDDAGSSGHRSPSKDRADSPR
EQFPGAANRGQDRERMVAEAKMWGENWREKRRAAQGSLDSPTGGG

>A033|LOC107486844

MGCFSCFDSREDQKLNPHGKHDHDHDHLRKQHQQQQHPHPSNQALPSHISRLPSGADKLRSRSNGGSKREL
PAPKDGPGVQIAAQTFTFRELAAATKNFRPESFIGEGGFGRVYKGKLESTGQVVAVKQLDRNGLQGNREFLVEV
LMLSLLHHPNLVNLIGYCADGEQRLLVYEFMPLGSLEDHLHVLVIGFHADLPPDKEPLDWNTRMKIAAGAAKGL
EYLHDKANPPVIYRDFKSSNILLDEGYHPKLSDFGLAKLGPVGDKSHVSTRVMGTYGYCAPEYAMTGQLTVKSDV
YSFGVVFLELITGRKAIDSTQPHGEQNLVTWARPLFNDRRKFSKLADPRLQGRFPMRGLYQALAVASMCIQESA
ATRPLIGDVVTALSYLANQAYDPNNTGHGNRGSGDDKRNRDDKGARLLKNDEAGGSGHRWDLEGSEKDESPR
ETARMLNRDLDRERAVAEAKMWGENWREKRRQSLEPGSFDATNV

>A034|LOC101754541

MSCLPCSGSSGKEAKSLDALSPSPRPAAKSAPVRSKSRASGSRKEDSVPVRRGGNTAHGPTQIFTFRELAIATKNF
RKDCLLGEGGFGRVYKGRMENGQVIAVKQLDRNGFQGNREFLVEVLMLSLLHHPNLVRLIGYCADGDQRLLVY
EYMVLGSLEDHLYDCPPDKEPLDWNTRMKIAAGAARGLEYLHDKANPPVIYRDFKSSNILLGEDYYPKLSDFGLA
KLGPVGDKTHVSTRVMGTYGYCAPEYAMTGQLTVKSDVYSFGVVFLELITGRKAIDHTQPSGEQNLVAWARPL
FRDRRKFCQLADPLLQGHYPKRGLYQALAVAAMCLQEQAASRPLIGDVVTALSYLASHPYDPNAPTTKDSRTCP
STPRAKTHRRTTSVPDAQHAAESLMLNFPDLRKDTTRGGEFEQDHTEGSGSSSSSGRNDGLDVPQLLAVPNGK
AYSEADSIQKSTVKVGGREK

>A035|108196266

MGCFPCFDSKEEETLNPQKQQANGLKDGHPTQPTNISRLSSGADRLKSRSNVGAKREQNGIKELADAQIAAQTF
TFRELAAATNNFRPESFIGEGGFGRVYKGRLQSTGQVVAVKQLDRNGLQGNREFLVEVLMLSLLHHPNLVNLIG
YCADGDQRLLVYEFMPLGSLEDHLHDLPPEKESLDWNTRMKIAAGAARGLEFLHDKASPPVIYRDFKSSNILLEEE
FHPKLSDFGLAKLGPTGDKSHVSTRVMGTYGYCAPEYAMTGQLTVKSDVYSFGVVFLELITGRKAIDSTQPQGQ
QNLVTWARPLFNDRRKFAKLADPRLQGQYPMRGLYQALAVASMCIQEQAAARPLIGDVVTALSYLANQAYDP
SSGPGNGYRLNGDKDEKNIRGSGRFLRNDVGGGSGRKWDLEGSEKDDSPRETARMLNRDLERERAVAEAKM
WGENLRDKRRQSAQGSFDANNG

>A036|LOC104807389

MGCFSCFDSKEEEKLNPRKESEDRKQPQPTVPNNVSSLSSGGEKLRSRSNRASKGELPIPKDGPGGVQIAAQTFT
FRELAAATRNFHPDSFLGEGGFGRVYKGRLESTGQVVAVKQLDRNGLQGNREFLVEVLMLSLLHHPNLVNLIGY
CADGDQRLLVYEFMPLGSLEDHLHDLPPDKEPLDWNTRMKIAAGAAKGLEFLHDKANPPVIYRDFKSSNILLYEG
FCPKLSDFGLAKLGPTGDKSHVSTRVMGTYGYCAPEYAMTGQLTVKSDVYSFGVVFLELITGRQAIDSTKPHGEQ
NLVAWARPLFNDRRKFIKLADPRLQGRFPMRGLYQALAVASMCIQEQAATRPLIGDVVTALSYLANQGYDPNR
DERRSRDERGGKLLSKNDEGGGSGRRFDLSGSEKDDSPRETARILNRDVDRERAVAEAKMWGENLREKRRQSA
QGTSDSSNG

>A037|M0YAZ8

MLGEGGFGRVYKGRLENGQAVAVKQLDRNGLQGNREFLVEVLMLSLLHHTNLVNLIGYCADGDQRLLVYEFM
PLGSLEDHLHDVPPEKEPLDWNTRMKIAAGAAKGLEHLHDKASPPVIYRDFKSSNILLGEGFHPKLSDFGLAKLG
PVGDKTHVSTRVMGTYGYCAPEYAMTGQLTVKSDVYSFGVVFLELITGRKAIDNTKPQGEQNLVAWARPLFKD
RRKFPKMADPMLQGRFPMRGLYQALAVAAMCLQEQATTRPHIGDVVTALSYLASQTYDPNAPTQHTRSNSST
PRARNMGGRNSEQRNGRSPNHHSPRTSKHGGEVSRTSSNGGDSGRRSGLDDMDMAGSQAGSPAQTGRKR
DTPRTADRHRAVVDAKMWGENSRERKRPNGHGSFDSTNE

>A038|XP_018811839.1

MGCFPCFDTKEEGKLNPVKESDGRKQGQPMVPSAISGLPSGADRLKSRSNAASKGELPGPKDGPGIHPGVHIAA
QTFTFRELAAATMNFRPESFIGEGGFGRVYKGRLETTGQIVAVKQLDRNGLQGNREFLVEVLMLSLLHHPNLVN
LIGYCADGDQRLLVYEFMPLGSLEDHLHDLPPDKEPLDWNTRMKIAAGAAKGLEYLHDKANPPVIYRDFKSSNIL
LDEGYQPKLSDFGLAKLGPVGDKSHVSTRVMGTYGYCAPEYAMTGQLTVKSDVYSFGVVFLELITGRKAIDGTRP
HGEQNLVAWARPLFNDRRKFSKLADPWLQGRYPIRGLYQALAVASMCIQEQAATRPLIGDVVTALSYLANQAY
DPKTASGHSHKGSGDRDERRNRDDRGGRISKNEESGGSGRRWDFEGSEKDDSPKETAKMLNRDLIRERAVAEA
KMWGENWREKRRQSAQGSFDGNN

>A039| ACY92439.1

MSAKTKHSVRVAQTSADARLHAAFEGSGSEGGAGDGGPGVSDYSKSEQPSASSRNEVAPEAITAYLQRMQRG
GLTQAFGCMLAVAGNSILALSENAPEMLEMAVGLSTDLRMLFTSGSTAALEQAAKEEDLSRVNPVVVQSCGGS
GKRFYAVLHRIEDVAGLVMDLEPIEGGLGVLVEKKSSAEMAVKSIERIQLLPEGDIGQLCQAVVEEVQEMTGYDR
VMAYKFHEDEHGEVVAEMRRPEQEPYLGLHYPSTDVPQASRMMFMKNGVRMIGDCTLPPVRVVQAKELAQ
PISLAGSTLRAPHGCHAQYMCNMGSVASLTMAVVIDDYDDDSSLSGSRKLWGLVVCHHTSPRKVSYPLRCACQ
KLMEAFGVQLNMELEFAAQLREHHILTTQTLLCDMLRRIRGAPIGIVSRSPSIMDLVKCDGAALYYGGKLWPLGT
TPSEFQVQDLAEWILGSSEEIASSGVTCTDRLAEAGYPGAAALGDAVCGMAAARITPNSDFLFWFRSHTAKEVF
WGGAEHDPQARDDDSRLLPRSSFKAFLEIVKRRSLPWEEVEVDAIRSLQLILRENLEEFCAAVAAGADGEDVDSL
VPLSRKLSLKESEEMGGAENSKRLERATSAAAGGAGGGWEKMSLPFNLAQEWMEAIRGTGDGGASGGVGGV
PFDWDAISVFQQTSFVVVDALKPDLPIIFASTGFFNLTGYTSREVIGGNCRFLQGPDTNPEDVASIREALVPQGTG
TFCGRLLNYRKDGSNFWNLLTIAPIKDDSGTIVKFIGVQLEVSKYTEGSRANRLRPNGLPQSLIKYDVRHQDKVSA
LVAQLVAALTKPDKVQPPRPSYAMRVSLTGQTIEPLSPGRAAAARPYSVSDVPQTAAISREGGGRRRHRSSTFLS
LLGMEEKDSEEDQFPEPELIMVDDASVARPGTSDDRERTRRGIDLATTLERIGHSFVITDPRLPDNPIIFASDQFLE
LTEYSREEVLGENCRFLQGRDTDRKAVQLIRDAVKEGRDVTVQLLNYTRGGRPFWNLFHLQAMTDTKGNLQYFI
GVQQETDMPDRVEQEKARVMRATAQNVDLAARELPDANVTPDHLWERHSKAVTPLPHSKINSPCWYAIRRV
QRRLRRGERLGLKHFRPIKPLGSGDTGSVHLVELRGTGQVFALKAMDKSMMLQRNKVHRARAEREILGMMDH
PFLPTLYASFQTKTHVCLIMDFCPRGDLFLLQDKQPNKTLSEEGARFYAAEVVVALEYLHCMGVIYRDLKPENVLL
QKNGHILLTDFDLSFLTSCRPQLITSGRSGRRRIKKRRVRVIFCAEPNVSSNSFVGTEEYIAPEIISGHPHSSAVDW
WALGILLYEMLYGRTPFCGRNRHKTFINVLNEELTFPTSIPVSIAGRQLIAGLLQRDPARRLGALRGASDVKKHPFF
QGIDWPLI

>A040|LOC107468111

MGGCFPCFGSSNKEESGGGGLKKEVAKKESFKESSAPQVHHPSRVSSDKSKSRCSSDPKKETPVPKDGPTAHIAA
QTFTFRELAAATKNFRPECLLGEGGFGRVYKGRLENTGQVVAVKQLDRNGLQGNREFLVEVLMLSLLHHPNLVN
LIGYCADGDQRLLVYEFMPLGSLEDHLHAENIMTYRLARCHNSANRFESALFNTMLFHLTNLPPDKEPLDWNTR
MKIAAGAAKGLEYLHDKANPPVIYRDLKSSNILLDEGYHPKLSDFGLAKLGPVGDKTHVSTRVMGTYGYCAPEYA
MTGQLTLKSDVYSFGVVFLELITGRKAIDNTRAHGEHNLVAWARPLFKDRRKFPKMADPLLQGRYPMRGLYQA
LAVAAMCLQEQAATRPLIGDVVTALTYLASQTYEPNVANQSNRLGPSTPRSRDDRRSMIDGVDSPDRGRLGSPS
THRNSPDYRRRDNRELSVGTELGRSVSGSGGSGRKWGLDDLERQDSRRDSPVNTARARETPRNRDLDRERAVA
EAKVWGENWREKKRANAMGSFDATNE

>A041|LOC105032131

MSCFSCFGSPDKEEEEEEEKNEEKEGGGDVMRDTSGASSSNHVTRASSDKSKMRNGSDSRNPTLVPRDATSSHI
AAQTFTFRELAAATQNFRQDCLLGEGGFGRVYKGRLENTGQVVAVKQLDRNGLQGNREFLVEVLMLSLLHHPN
LVNLIGYCADGDQRLLVYEFMPLGSLEDHLHDIPPDRKPLDWNTRMKIAAGAAKGLEYLHDEANPPVIYRDFKSS

NILLAEGYFPKLSDFGLAKLGPVGDKTHVSTRVMGTYGYCAPEYAMTGQLTLKSDVYSFGVVFLELITGRKAIDNT
RAAGEHNLVAWARPLFRDRRKFPKMADPKLQGRYPMRGLYQALAVAAMCLQEQAATRPLIGDVVTALTYLAS
QTYDPNAASSQSNRAGPSTPRAREDWKNPSVGFDNQHAIQSPHRHSPDFRHRDFFRGPNIGDGGRGDVVGQ
SGRNLDPDELQIQESQMNSAIHVGKAREAPGTSNRDLDRERAVAEAKVWGENWRERKRMKEPISFDSTSLVPG
TRCGKKASIEGRVAFQIE

>A042|A0A0Q3I194

MRRLLGWGGGAPLDMSCFSCFDSPADEQLNPKVGGSRYGGSSVAAASYGGGVGGGRQGEMSYPELHQPMA
APRIEKLSSGAGHARVKGNAIAREASVPKDANGNVISAQTFTFRELATATRNFRQECFLGEGGFGRVYKGRMES
TGQVVAIKQLNRDGLQGNREFLVEVLMLSLLHHQNLVSLIGYCADGDQRLLVYEYMPFGSLEDHLHDLPIDKEAL
DWSSRMKIAAGAAKGLEYLHDKANPPVIYRDFKSSNILLDESFHPKLSDFGLAKLGPVGDKSHVSTRVMGTYGYC
APEYAMTGQLTVKSDVYSFGVVLLELITGRRAIDSTRPHGEQNLVSWARPLFNDRRKLPKMADPRLEGRYPMR
GLYQALAVASMCIQSEAASRPLIADVVTALSYLASQSYDPNAPHASRKPGGDQRSKTGENGRVVSRNDEAGSSG
HKSPGKDREDSPNGLPGIVNKDLERERMVAEAKRWGDRERMVAEAKMWGDRERMVAEAKMWGENWREK
KHADTNGQGSLDSPTRNG

>A043|A0A2H5N6Y7

MGGCFPCFGSSNNKETGGGGGGGAVGAVKELNRKDSAKDGSIAQSHHVNRVSSDKSKSRNGSDPKKESAIPKE
PTAHIAAQTFTFRELAAATKNFRPECLLGEGGFGRVYKGRLESTGQVVAVKQLDRNGLQGNREFLVEVLMLSLL
HHSNLVNLIGYCADGDQRLLVYEFMPLGSLEDHLHDLPPDKEPLDWNTRMKIAAGAAKGLEYLHDKANPPVIYR
DLKSSNILLDEGFHPKLSDFGLAKLGPVGDKTHVSTRVMGTYGYCAPEYAMTGQLTLKSDVYSFGVVFLELITGRK
AIDNTRPPGEHNLVAWARPLFKDRRKFPKMADPLLQGRYPMRGLYQALAVAAMCLQEQAATRPLIGDVVTAL
TYLASQTYDPNAASNLSNRVGPSTPRNRDDRRSMADGQDSPDEHGRVGRHGSPSTHKNSPDYRKRSHPRDGS
TGAELSRNETGGGSGRKWLGDDLERQESQRDSPVNTGRARQSPRNRDLDRERAVAAAKVWGENWREKKRA
NAMGSFDGTDE

>A044|A0A2I0VJV6

MGCFPCFGSLEEEEEEKKREVKTGGADFSKEASAAPSSNQMTRVSSVFDSNGMNSSVDCAFVSSRSVSDSKKET
LVPKEGNATHIAAQTFTFRELATATKNFKPECLLGEGGFGRVYKGRLESGQVVAVKQLDRNGLQGNREFLVEVL
MLSLLHHPNLVNLIGYCADGDQRLLVYEYMPLGSLEDHLHDLPSDKEPLDWNTRMKIAAGAAKGLEYLHDKAN
PPVIYRDFKSSNILLGEEYHPKLSDFGLAKLGPVGDKTHVSTRVMGTYGYCAPEYAMTGQLTLKSDVYSFGVVFLE
LITGRKAIDNTRPAGEQNLVAWARPLFKDRRKFPKMADPLLQGHYPMRGLYQALAVAAMCLQEQAATRPLIG
DVVTALSYLASQTYDPNAPATQSSRVGPSTPRSRDERKNHGSGSDSKSATDSPHRDSPKLRLRDSIKGMNLGTD
SDGTPGRKWGLEELERQESHRDSPNHLAKPRDSPKALNRDADRERAIAEAKVWGENWRERKRATMPKSFDST
NE

>A045|A0A199VK74

MGCFPCFGSSHEGEEGKGGAKGSGDVKKDASAAPSSRHMSRISSDKLKSRNGSDSKKEGSVQKEESTAHIAAQT
FTFRELAAATKNFRQDCLLGEGGFGRVYKGRLEGGQVVAVKQLDRNGLQGNREFLVEVLMLSLLHHANLVNLIG
YCADGDQRLLVYEFMPLGSLEDHLHDLPPDKEPLDWNTRMKIAAGAAKGLEHLHDKANPPVIYRDFKSSNILLG
EGFHPKLSDFGLAKLGPVGDKTHVSTRVMGTYGYCAPEYAMTGQLTVKSDVYSFGVVFLELITGRKAIDNTRPA
GEQNLVAWARPLFKDRRKFPKMADPLLQNRFPSRGLYQALAIAAMCLQEQAATRPLIGDVVTALSYLASQTHN
PNAASVQSTRSGPSTPRVREDRRSIRLPDAQNSVQSPNPNSPDLRRKESLKGANSGADSGRKSGLDESEMQEFQ
YGSPVHARKNRETQRNSDRQRAIAEAKLWGENWRERKQANTRGSFDSTNE

>A046|A0A4S8IPK9

MGCFPCFESEEAAQLSHVNDCDRKREEQPMVAPRVEKLSSGDDRIRARNDVNGKTESLGPKEGSDFAISAHTFT
FRELSSATGNFRAQSLLGEGGFGRVYRGRLETTGQVVAVKQLDRNGLQGNREFLVEVLMLSMLHHPNLVNLIG
YCADGDQRLLVYEYMSMGSLEDHLHASYLTGRDIVINQPIQLSSDLPPDKEPLDWNTRMKIAAGAAKGLEYLHN
KASPPVIYRDLKSSNILLDKGFHPKLSDFGLAKLGPVGDKSHVSTRVMGTYGYCAPEYAMTGQLTVKSDIYSFGVV
LLELITGRKAVETTKSHAEQNLVSWARPMFNDRRKLPSLADPKLQGRYPMRGVYQALAVASMCIQEEAASRPAI
ADVVTALSYLASQAYDPGACPTTNNRPGGERRNRSADEGSGRTPALNSDAETAHNWQMNGENTSKETSANV
RQGFDRERALAEAKIWGENWREKTQAKANAEENFHVASAIG

>A047|A0A2H3XZG9

MGCFPCFDSKEAAQLNPGEGRDDRREELPMVAPRVEKLSSGADRSKTRSNLDLKRESLRPNEGSGFNISAQTFT
FRELAVATKNFRPECFLGEGGFGRVYKGCLESTGQVVAVKQLDRNGLQGNREFLVEVLMLSLLHHPNLVNLIGY
CADGDQRLLVYEYMPLGSLEDHLHDLPPEKEPLDWNRRMKIAAGAAKGLEYLHDKANPPVIYRDFKSSNILLDE
GFHPKLSDFGLAKLGPVGDKSHVSTRVMGTYGYCAPEYAMTGQLTVKSDVYSFGVVLLELITGRKAIDSTRAHGE
QNLVSWARPMFNDRRKLPKLADPKLQGRYPMRGLYQALAVASMCIQEEAASRPLIADVVTALAYLASQAYDPN
AVPNSNNRSGGERRSRTSDEKGGRMQAKNNDGGSGCKWELDVEKEDSPRETVGILNRDFDRERAVAEAKM
WGENWREKRRANANAQGSVDAASGNG

>A048|LOC105168454

MGCFPCFDSREEEKLNPHKERDDHREVHPSIPSSISKLSSGGDRLKSRSNVGPRKEASGLKDLPDAQIAAQTFTFR
ELANATNNFRPECFLGEGGFGRVYKGRLSNGQIVAVKQLDRNGLQGNREFLVEVLMLSLLHHPNLVNLLGYCAD
GDQRLLVYEFMPLGSLEDHLHDLPPDKEPLDWNTRMKIAAGAAKGLEYLHDKANPPVIYRDFKSSNILLGEGYSP
KLSDFGLAKLGPTGDKSHVSTRVMGTYGYCAPEYAMTGQLTVKSDVYSFGVVFLELITGRKAIDNTLPQGEQNL
VAWARPLFNDRRKFAKLADPRLQGKFPMRGLYQALAVASMCIQEQAAARPLIGDVVTALSYLANQAYDPTLAP
GHSSRTAGDKDDRSKDERGGKILRNEEGGASGRKWDLEGSERDDSPRETAKMLNRDLERERAVAEAKMWGE
NWREKRRQNAVGSFDGTNG

>A049|102723023

MAAPRVEKLSAGAEKGRVKSNAIAREPSAPKDANGNVISAQTFTFRELATATRNFRPECFLGEGGFGRVYKGRLE
SSGQVVAIKQLNRDGLQGNREFLVEVLMLSLLHHQNLVNLIGYCADGDQRLLVYEYMHFGSLEDHLHDLPPDKE
ALDWNTRMKIAAGAAKGLEYLHDKANPPVIYRDFKSSNILLDESFHPKLSDFGLAKLGPVGDKSHVSTRVMGTY
GYCAPEYAMTGQLTVKSDVYSFGVVLLELITGRRAIDSTRPHGEQNLVSWARPLFNDRRKLPKMADPRLEGRYP
MRGLYQALAVASMCIQSEAASRPLIADVVTALSYLASQSYDPNAPHASRKPGGDQRSKVGENGRIVSRNDEASS
SGHKSPNKDREDSPKEPPGILNKDFDRERMVAEAKMWGDRERMVAEAKMWGDRERMVAEAKMWGENW
RDKRRAIENGQGSLDSPTENG

>A050|LOC104700667

MGCFGRTVKSNKRSEPKTTKKNDVTSPKKLTNDPNIVHKNINRDNQTQPSSDCLKVTLCGNVNEEVDTKEEDQL
ALDAKDTLVEDEVSGKKAQTFTFGQLAVATGNFKSDCFLGEGGFGKVYRGFIEKINQVVAIKQLDRTGSQGIREF
VVEVLTLSLADHPNLVKLIGFCAEGVQRLLVYEYMPLGSLDNHLHDLPHSKKPLAWNTRMKIAAGAARGLEYLH
DTIKPPVIYRDLKCSNILLDEEYHAKLSDFGLAKVGPRGSETHVSTRVMGTYGYCAPDYALTGQLTFKSDVYSFGV
VLLELITGRKAIDNTRPRNKQSLVEWARPLFKNRNNFKKMVDPLMEGEYPIRGVYQALAIAAMCVQEQPSMRP

VIADVVMALDHLASSKYDHAHHHRRKHSNVTETRGDDEEKKTLPESNVTEKRGNEEEKIVVPESNVCVEEEKQEI
KICSDQAT

>A051|A0A446JZQ8

MGCLPCFGSSGKGEPAKKGGARKDVPSDRRATGVGSDKPKPQGLLDSKKDTVIPREGNNQHIAAHTFTFRELA
AATKNFRQDCLLGEGGFGRVYRGRLDNGQAVAVKQLDRNGLQGNREFLVEVLMLSLLHHDNLVNLIGYCADG
DQRLLVYEYMPLGSLEDHLHDIPPEKEPLDWNTRMKIAAGAAKGLEYLHDKASPPVIYRDFKSSNILLGEEFHPKL
SDFGLAKLGPVGDKTHVSTRVMGTYGYCAPEYAMTGQLTVKSDVYSFGVVFLELITGRKAIDNTKPHGEQNLVA
WARPLFKDRRKFPKMADPALQGRFPMRGLYQALAVAAMCLQEQAATRPFIGDVVTALSYLASQAYDPNAPTQ
HSRSNASTPRARDRSS

>A052|4338592

MGVTSWYQSLPRLRSPLLDRIVGVAELGGAYVRYLIGRSVKKFDYINRVVKRFRFRSTRHRGVVSWLVIPGGGAV
TVVVFGAIAPGTLHRKPTTPLAGSTIPVLPGGTVVGGGSCAAVPQRRRTCFRGGRLWDEEGRGKGGWGRVCCL
GRPGRRREGDKPKPQGLLDSKDTVIPREGNNQHIAAHTFTFRELAAATKNFRQDCLLGEGGFGRVYRGRLDNG
QAVAVKQLDRNGLQGNREFLVEVLMLSLLHHDNLVNLIGYCADGDQRLLVYEYMPLGSLEDHLHVSELICILKAK
FCCISWEDIPPEKEPLDWNTRMKIAAGAAKGLEYLHDKASPPVIYRDFKSSNILLGEEFHPKLSDFGLAKLGPVGD
KTHVSTRVMGTYGYCAPEYAMTGQLTVKSDVYSFGVVFLELITGRKAIDNTKPHGEQNLVAWARPLFKDRRKFP
KMADPALQGRFPMRGLYQALAVAAMCLQEQAATRPFIGDVVTALSYLASQAYDPNAPTQHSRSNASTPRARD
RGSVNGDQRRIRSPNHHSPDLRRKEVTTTSKYEAEVSRNSSGGGSGRQSGLDDRDVTGSQQGSPARAGKRRET
SRTSERQRAIAEAKTWGENSRERKWPNARGSFDSTNE

>A053|ANC96959.1

QPPAHSNEQPVLRKKSPDIGSHGAVLPGEGGLKKNRSSGLRPFVGSRKSLMNEQEPSEFIEPEILMTRDEPLDEM
DERGKEIRKGFDLATTLERIEKNFVVTDPRLPDNPIIYASDNFLELTEYSREEVLGRNCRFLQGPETDEETVKRIRKAI
REQREITVQLLNYTKSGRRFWNLFHLLPMRDEKGELQYFIGVQLDGTEHIDPAQKRLSERTEIEGAKIVNETATNV
DVAVRELPDANMTPDALWERYSKKVLPKPHKMNNKSWEAIQKIWERGEKLGLKHFSLLKPLGFGDSGSVYLVE
LRGSGEIFAMKAMDKSVMLHRNKVHRAFVEREILARMDHPFLPTLYASFQTPTHVCLITEFCPGGDLFLLLDRQP
TKIVSEEASRFYLAEVVIALEYLHCQGVLYRDLKPENVLLQRDGHILLSDFDLSFIASCDPQLVQPSQPSGKRKKPKS
VKPPFIMAEPMASCNSFVGTEEYIAPEVITGAGHSGAVDWWALGILLYEMIYGRTPFRGKNRQKTFANVLNKEL
VFPSSIPVSLAARQLMNGLLHRDPAKRIGSLKGVHEIKQHAFFHGIKWPLIRSMAPPKLETPQMNDTQTDTKVEL
EWDEYESRIPPFISEVF

>A054|EMS52724.1

MDDIITAGSAAAAASPGFTTQDEMIGLDGDMDSELEYDEEEPDEQEDKDEEPAPIPPKRRKKKGAVRTGCLAKA
WKVVCLDPITGRNQSIDTYWKRINAEFDKRKLVDPDFKGVFMQRGSKAMANRWGLIQSACNKWHGIVEKIAA
RPESGASVEDQGGGGGGNGTGGRTVSAASSSGVGAREERPMVPPRVEKLPAGAEKARAKGNAGMKELSDLR
DANGNVLSAQTFTFRQLTAATRNFREECFIGEGGFGRVYKGRLDGGQVVAIKQLNRDGNQGNKEFLVEVLMLS
LLHHQNLVNLVGYCADGEQRLLVYEYMPLGSLEDHLHDLPPDKEPLDWNTRMKIAAGAAKGLEYLHDKAQPPV
IYRDFKSSNILLGDDFHPKLSDFGLAKLGPVGDKSHVSTRVMGTYGYCAPEYAMTGQLTVKSDVYSFGVVLLELIT
GRKAIDSTRPHGEQNLVSWARPLFNDRRKLPKMADPGLQGRYPMRGLYQALAVASMCIQSEAASRPLIADVVT
ALSYLASQIYDPNAIHASKKAGGDQRSRVSDSGRTLLKNDEAGSSGHKSDRDDSPREPPPGILNDRERMVAEAK
MWGANLREKTRAAANAQGSLDSPTETG

>A055|A0A2K3P4K4

MGGCFPCFGSSNKEDTNAVKEVSKKETFKEASLPQSHHPTRVSSDKSKSKSVSDSKKEAPVQKDGPTAHIAAQTF
TFRELAAATKNFRPECLLGEGGFGRVYKGRLESTGQVVAVKQLDRNGLQGNREFLVEVLMLSLLHHPNLVNLIGY
CADGDQRLLVYEFMPLGSLEDHLHDLPPDKEPLDWNTRMKIAAGAAKGLEYLHDKANPPVIYRDLKSSNILLDE
GFHPKLSDFGLAKLGPVGDKTHVSTRVMGTYGYCAPEYAMTGQLTLKSDVYSFGVVFLELITGRKAIDNTRGHG
EHNLVAWARPLFKDRRKFPKMADPLLQGRYPMRGLYQALAVAAMCLQEQAATRPLIGDVVTALTYLASQTFD
PNAGNQSSRGGSSTPRMRDDRRSSMADSVDSPDRARLGSPSTHRNSPDFRKREGRDPSELGRIDTGGGGSGR
KWGLDDLERRDSQRDSPVNTGKGRETPRNRDLDRERAVAEARVWGENWREKKRANAMGSFDGQRTVERVN
LATKVPDRYWLFTPALKYSARPPVSIYVPQQNAASIDILNMSCLYSLIDGYCLVMGRSMLCTLFGLLEIALSVMVL
RQVGD

>A056|A0A368RUW5

MGCFPCFGSGQEDDELGYYGGNGGAAGWAAAAASSSAAAAAAAAVGGGAEAAVAAPPRAERSPTGSNKSK
AKGNGSKKELAVLKDANGNVISAQTFTFRQLAAATKNFRDECFIGEGGFGRVYKGRLDGTGQVVAIKQLNRDG
NQGNKEFLVEVLMLSLLHHQNLVNLVGYCADGDQRLLVYEYMPLGSLEDHLHDLPPGKEALDWNTRMKIAAG
AAKGLEYLHDKADPPVIYRDFKSSNILLGEGFHAKLSDFGLAKLGPVGDKSHVSTRVMGTYGYCAPEYAMTGQLT
VKSDVYSFGVVLLELITGRKAIDSTRPAAEQNLVSWARPLFNDRRKLPKMADPGLEGRFPTRGLYQALAVASMCI
QSEAASRPLIADVVTALSYLANQIYDPNTASTSKKGGGDQRSRVSDSGRALSRNDDTGSSGHRSPSKDLDDSPRE
RHATGTAHKGERERMVAEAKMWGENWREKQRAAQGSLDSPTGAAPLKCRRTNLYSFYACSMPWWYCGRLR
NTVFSKECPRSHIPIRLSEAGAGEVYWVWWSFAISCAYRDQQELGCLATKKSRCEFTEYCD

>A057|Carubv10000618m

ASSSLSLSLSLSLSLSLSLFSFLFLFQLQFVFTSSNLNKVCFFIGTQNLNYPSFLLGVCKVSLFLIYPIVPVHLFHNRIEGF
CWIIDIFFILGKLSGEEMGCFSCFDSSDDEKLNPVEESNGQKKQLQPTVSNSISGLPSGGEKLSSKSNGGSKRELLL
PRDGLGQIAAHTFAFRELAAATMNFHPDTFLGEGGFGRVYKGRLDSTGQVVAVKQLDRNGLQGNREFLVEVL
MLSLLHHPNLVNLIGYCADGDQRLLVYEFMPLGSLEDHLHDLPPDKEALDWNMRMKIAAGAAKGLEFLHDKA
NPPVIYRDFKSSNILLDEGFHPKLSDFGLAKLGPTGDKSHVSTRVMGTYGYCAPEYAMTGQLTVKSDVYSFGVVF
LELITGRKAIDSEMPHGEQNLVAWARPLFNDRRKFIKLADPKLKGRFPTRALYQALAVASMCIQEQAATRPLIAD
VVTALSYLANQAYDPSKDESRRNRDERGARLITRNDEGGGSGSKFDLDGSEKEDSPRETARILNRDINRERAVAE
AKMWGESLREKRRQSEQGTSESNSTG

>A058|A0A2R6W6N6

MRPGPLAFRKQTQEPEMGCFSCFGTRKQDNRKPLKKDDSGREGQSTGGSANNNLTKVPSENKTKGKGSFDAK
KEVPTADGATHIAAQTFTFRELAAATKNFRPECLLGEGGFGRVYKGRLENTGQVVAVKQLDRNGLQGNREFLVE
VLMLSLLHHPNLVNLIGYCADGDQRLLVYEYMPLGCLEDHLHDLPPDKEALDWNTRMKIAAGAARGLEYLHDK
ANPPVIYRDFKSSNILLDEGFHPKLSDFGLAKLGPVGDKTHVSTRVMGTYGYCAPEYAMTGQLTLKSDVYSFGVV
LLELITGRKAIDNARAPGEHNLVAWARPLFKDRRKFPSMADPMLQGRYPMRGLYQALAVAAMCLQEQAATRP
LIGDVVTALSYLASQTYDPGLHPVGSSRFAPPTPSREKKEKDKKLGSGGSERHGNSPMLPSTDERVQKERGGRQ
AGSRSPSNLPSTASPDLRKDSGRGRNNGTFDGRGQDTGAARKRDSDEWDRYDSHRESPGHTGRTPKDARSLA
PSRERERAVAEARVWGENWRERKRSNNQGGGDGFDGGFG

>A059|F6HDG9

MGGCFPCFGSSNKEGNGVKKDVAKKDSVKDGSSAQSHHVTRVSSDKSKSRSGSDSKKEPAVPKDGPTAHIAAQ
TFTFRELAAATKNFRPESLLGEGGFGRVYKGRLESTGQVVAVKQLDRNGLQGNREFLVEVLMLSLLHHPNLVNLI
GYCADGDQRLLVYEFMPLGSLEDHLHDLPPDKEPLDWNTRMKIAAGAAKGLEYLHDKASPPVIYRDLKSSNILLD
EGYHPKLSDFGLAKLGPVGDKTHVSTRVMGTYGYCAPEYAMTGQLTLKSDVYSFGVVFLELITGRKAIDNNRAA
GEHNLVAWARPLFKDRRKFPKMADPLLQGRYPMRGLYQALAVAAMCLQEQAATRPLIGDVVTALTYLASQTY
DPNAASAQSNRVGPSTPRSKDDRRSLPDGLDSPDNPGRGGRLGSPSTHKNSPDFRRRDPLRDLSTATDLGRSEA
DGGSGRKWGLDELERQESQRDIPISAGRARDTERDIPISSGRARETPRNRDLDRERAVAEAKVWGENWREKKR
ANVVGSFDGTNG

>A060|A0A2J6L327

MGCFPCFDSSNKEGNGVKEVSKKDIGKEGSAPHQSHHVSRVSSGKSKSRVSLDPKKDQMVTKDGQTANIAAQT
FTFRELAAATKNFRPESMLGEGGFGRVYKGRLDSTGQVVAVKQLDRNGLQGNREFLVEVLMLSLLHHPNLVNLI
GYCADGDQRLLVYEFMPLGSLEDHLHDLPPDKEPLDWNTRMKIAAGAAKGLEYLHDKANPPVIYRDLKSSNILL
DEGYHPKLSDFGLAKLGPVGDKTHVSTRVMGTYGYCAPEYAMTGQLTLKSDVYSFGVVFLELITGRKAIDNTRAP
GEHNLVAWARPLFKDRRKFPKMADPLLQGRYPVRGLYQALAVAAMCLQEQAATRPLIGDVVTALTYLASQPYD
PEAARAERSARGSGSGTPRSRADRRNPSDGGLDDSMPRAHGSPSTYKNSPDYRKRESLREINTRGADLADGESS
NGGGGSGRKWGVVEDSDSQRNSPANTSSRGRNRDLERERAVAEAKVWGENWRDRKRTNTTSGGGSGSGG
GGGGGGSFDATNE

>A061|111375815
MGCLPCFGSSEKEQNNVNAVKEVAKKESFKEGSGAQSHNHVNRTSSDKSKSRSGNDPKKEAASPKEPLAYIAAQ
TFTFRELASATQNFRPECLLGEGGFGHVYKGQLESTGQVVAVKQLDRNGLQGNREFLVEVLMLSLLHHPNLVNL
IGYCADGDQRLLVYEYMPLGSLEDHLHDLLPEKKPLDWNTRMKIAAGAAKGLEYLHDKANPPVIYRDLKSSNILL
DEGYFPKLSDFGLAKLGPVGDKTHVSTRVMGTYGYCAPEYAMTGQLTLKSDVYSFGVVFLEIITGRKAIDNRQAP
GEQNLVAWARPLFRDRMKFPKMADPRLQGHYPIRGLYQALAVAAMCLQEQAATRPLIGDVVTALTYLASQTY
DPNDPSSQSNRIDASTLRHRDARRNMSIGTDTLDESGRRSHHGSPSVNKNSPDFRKRDSFKEFNNGELRRIETG
GGSGRKWGLDEPERPDSERDTPVSAGRGRETPRNRDLDKERAVADAKVWGENWRGRKRANGGVSFDCTND
SLPNVSLLC

>A062|A0A2G5DLW4

MGKCFPCFGSSRKENKQGVKDSSGKKDFSKDTSFAQAHGISRVSSDKSKSRNASDSKKELAGPKDGSTTHIAAQ
TFTFRELAAATKNFRPECLLGEGGFGRVYKGRLESTGQVVAVKQLDRNGLQGNREFLVEVLMLSLLHHSNLVNLI
GYCADGDQRLLVYEFMPLGSLEDHLHDLPPNKEPLDWNTRMKIAAGAAKGLEYLHDKANPPVIYRDFKSSNILL
DEEYHPKLSDFGLAKLGPVGDKTHVSTRVMGTYGYCAPEYAMTGQLTLKSDVYSFGVVFLELITGRKAIDNTRGP
GENNLVAWARPLFKDRRKFPKMADPLLQGRYPMRGLYQALAVAAMCLQEQAATRPLIGDVVTALTYLASQTY
DPNAAAAQNNRVGPSTPRSKDGQKSSQGSGASNQGDVGSGGRHGSQSPHQDSPNYRHKDLIRGVSFGAEVG
RGETGSGSGRKFGLNETERQDSQKNSPAGRPRDTPRQFNRDIDRERMVAEAKVWGENWRERKRTSTMGSFD
STNEL

>A063|A0A4D8Z9L6

MGGCVPCFGSSNKDGNGNDFDNGVKEMGKKEPFKDGSAAHSNSQVNRVNSDKSKARNSHDSKKEPGIPKEP
TAHIAAQTFTFRDLAAATKNFRPECLLGEGGFGRVYKGKLESGQVVAVKQLDRNGLQGNREFLVEVLMLSLLHH
QNLVNLIGYCADGDQRLLVYEYMPLGSLEDHLHDLSPDKQPLDWNTRMKIAAGAAKGLEYLHDKANPPVIYRD
LKSSNILLDEDYFPKLSDFGLAKLGPVGDKTHVSTRVMGTYGYCAPEYAMTGQLTLKSDVYSFGVVFLELITGRKAI
DNNRGAGEHNLVAWARPLFKDRRKFPKMADPDLQGQYPIRGLYQALAVAAMCLQEQAATRPLIGDVVTALTY

LASQTYDPNSPSARSNKTGSSTPRHRHERSASNGMDGLDDAGRGGHHRSSSAHKNSPDLRKRESPREFSVGGE
LRRIETSGGSGSKWGSDEADRADSQRDSPASAGRVRESSRNRDLERERAVALAKVWGENWRERKRTSAMGSF
DGNE

>A064|A0A2K1KFP5

MGCFPCLDSKPKEGKSLKKEDNNSSRNGQSPADNPVAQIPKLSSGNSKRGVTKESVDKKEPVREGNTHINAQTF
TFRELVAATKNFRADCLLGEGGFGRVYKGRLESTGQVVAVKQLDRSGLQGNREFLVEVLMLSLLHHTNLVNLIGY
CAEGDQRLLVYEFMPLGCLEDHLHDLPQDKECLDWNTRMKIAAGAARGLEYLHDKAQPSVIYRDFKSSNILLDE
KFHPKLSDFGLAKLGPVGDKTHVSTRVMGTYGYCAPEYAMTGQLTLKSDVYSFGVVLLELITGRKAIDNARAVGE
QNLVAWARPLFKDRRKFPFMADPLLQGRYPMRGLYQALAVAAMCLQEQAATRPLIGDVVTALSYLASQTYDP
GVQPQGSSRFAPATPSGEKREKEKKVPLGPNGAEERMVKEQKVGKVAMQSPDLRVKRGGARARVANGVLEES
QGSELGKMRESEESVRREEGGRDSPRAVRRTGTARDGVKPHIPTRERERAVAEARVWGENWREKQRGPTT

>A065|A0A022S2R6

MGGCFPCFRSSSTEGISNSNNGVKEVSRKESFKEGSAANSNSNVSRLSSEKSRARNSSDSKKEIGIPKEPTAHIAA
QTFTFRELAAATKNFRPESLLGEGGFGRVYKGRLESTGQVVAVKQLDRNGLQGNREFLVEVLMLSLLHHPNLVN
LIGYCADGDQRLLVYEYMPLGSLEDHLHDRPPDKEPLDWNTRMKIAAGAAKGLEYLHDKANPPVIYRDLKSSNIL
LDEEYSPKLSDFGLAKLGPVGDKTHVSTRVMGTYGYCAPEYAMTGQLTLKSDVYSFGVVFLELITGRKAIDNTRE
AGEHNLVAWARPLFKDRRKFPKMADPLLKGHYPMRGLYQALAVAAMCLQEQAATRPLIGDVVTALSYLASQIY
DPNAPQSNKAGGPPTPRHRDGRRNTSEGTDGLDDDSQRGSPSIHKNSPDSRKRGSGREFGELRKVETGGGSRS
KWGSEESERPDSQRDSPASAGRGREALRNRDLERERAVALAKVWGENWRERKRTNGAGSFDGTNE

>A066|A0A2R6PK59

MGGCFPCFRSSNKDGNGVKEVAKKEFVKEPSAAQSVNRVISDKSKTRNGSDTKKDPKKEPAVQKDGPPAHIAA
QTFTFRELATATKNFRPECLLGEGGFGRVYKGRLESTGQVVAVKQLDRNGLQGNREFLVEVLMLSLLHHPNLVN
LIGYCADGDQRLLVYEFMPLGSLEDHLHDLPPNKEPLDWNTRMKIAAGAAKGLEYLHDKANPPVIYRDLKSSNIL
LGEGYHPKLSDFGLAKLGPVGDKTHVSTRVMGTYGYCAPEYAMTGQLTLKSDVYSFGVVFLEIITGRKAIDNTRV
AGEHNLVAWARPLFKDRRKFPKMADPLLQGRYPMRGLYQALAVAAMCLQEQAATRPLIGDVVTALTYLASQT
YDPNAPTAQNRAGPSTPRHKDDRRNMAEGQNSPDDSRRGGSHGSPSNHRNSPDFRKRDAMRELSELGRIETG
CGSGRKWGVDDVERQDSQRDSPVSAGRMKETPRNRDLDRERAVAEAKVWGENWRERKRANAMGSFDGTN
E

>A067|A0A2Z7D519

MGCLRCFGSSNEKEGIDNNSGVKEVAKKESFKDCSAAQSNNHVNRLSSDKSKSWGTNDSKREASIPKEPTANIA
AQTFTFRELAAATKNFRPECLLGEGGFGRVYKGRLESTGQVVAVKQLDRNGLQGNREFLVEVLMLSLLHHPNLV
NLIGYCADGDQRLLVYEYMPLGSLEDHLHDLHQDKEPLDWNTRMKIAAGAAKGLEYLHDKANPPVIYRDLKSSN
ILLDNGYFPKLSDFGLAKLGPVGDKTHVSTRVMGTYGYCAPEYAMTGQLTLKSDVYSFGVVFLEIITGRKAIDNTK
GAGEHNLVAWARPLFKDRRKFPKMADPLLQGQYPIRGLYQALAVAAMCLQEQAATRPLIGDVVTALTYLASQT
YDPNAPVSQTNRVGPSTPRHRDERRHTSDGNDSLYDPSHQGSPSTYKNSPDYRRRDPARAGADLRRVETGSGS
GRKWGLDDLDRPGSQKDSPVSGGRARETPRNRDLDRERAVAEAKVWGENWRERKRANGMDSFDGTNE

>A068|A0A1Q3BYQ7

MGGCFPCFGSSTKEGSGGIVKEVIKKDSVKDVSVAQSHHVSRVSSDKSKSRSGSDLKKESAVPKDGPTQHIAAQT
FTFRELAAATKNFRPECLLGEGGFGRVYKGRLESTGQVVAVKQLDRNGLQGNREFLVEVLMLSLLHHPNLVNLIG

YCADGDQRLLVYEFMPLGSLEDHLHDLPPDREPLDWNTRMKIAAGAAKGLEYLHDKANPPVIYRDLKSSNILLDE
GYHPKLSDFGLAKLGPVGDKTHVSTRVMGTYGYCAPEYAMTGQLTLKSDVYSFGVVFLELITGRKAIDNARTHG
EHNLVAWARPLFKDRRKFPRMADPLLQGRYPMRGLYQALAVAAMCLQEQAATRPLIGDVVTALTYLASQTYD
PNASPAQGNRVGPSTPRRRDDRRNTADGLDGLDERGRLGSPSAQSPDYWRREHVRDSSTGSELGRSETGGGS
CRKWGLDDVERPDSLRDSPVNIGRARETPRNRGLDRERAVAEAKVWGENWRDKKRANAMGSFDGTNE

>A069|A0A4D8YTN4

MGCFSCFGSSKTEVNSSSNGVKEVSKKDSVKEGSAAQTNSNVNRVNSDKPRLRTGNDSKKEMTIPKEPNIAAQI
FTFRELAAATKNFRPESLLGEGGFGRVYKGRLESTGQAVAVKQLDRNGLQGNREFLVEVLMLSLLHHPNFVNLIG
YCADGDQRLLVYEYMPLGSLEDHLHDVPPDKEPLDWNTRMKIAAGAAKGLEYLHDKANPPVIYRDLKSSNILLD
EGYFPKLSDFGLAKLGPVGDKTHVSTRVMGTYGYCAPEYAMTGQLTLKSDVYSFGVVFLEIITGRKAIDNMRSAG
EQNLVAWARPLFKDRRKFPKMADPLLQGRYPMRGLYQALAVAAMCLQEQAATRPLIGDVVTALTYLASQSYD
PNAPGSQSRGGSGTPRHRDVRRNISDADSLEDVGRGGHYGSPSAHKNSPNFRMKDSVGDLNAGAELGRIDTG
GGSGRRWGVDEPDRSDSQWGSPASGVRTRETPKNRDLDRERAVAAAKVWGENWREKKRTNGPGDDVV

>A070|A0A2I0B394

MGCFPCFGSSEEGEKTKEEVKAGGGNLKKEPAVVPSSKQVTGVSSDKLRSNDSESKKDALVPKEGSATRIAAQIF
TFRELAVATKNFRPECLLGEGGFGRVYKGRLENGQVVAVKQLDRNGLQGNREFLVEVLMLSLLDHPNLVNLIGY
CADGDQRLLVYEFLPLGSLEDHLHDLPPNKEPLDWNTRMKIAAGAAKGLEYLHDRANPPVIYRDFKSSNILLGEG
YHPKLSDFGLAKLGPVGDKTHVSTRVMGTYGYCAPEYAMTGQLTLKSDVYSFGVVFLELITGRKAIDNTRPTGEQ
NLVAWARPLFKDRRKFPKMADPLLQGHYPTRGLYQALAVAAMCLQEQAATRPLIGDVVTALSYLASQTYDPNA
PSTPRSREERKSLGGSSESKQARDSPRRDSPKFRQRDSIKGMNSVSDCIRVDADVGSGRKWGLEDLEKEEPQRD
SPGHLGRSRDSPRTMNGDSNRERAIAEAKLWGENWRERKRGNDPECIDSTDKQEKTR

>A071|D8SMJ2

MPNFCSSFGCQISCLRFWLFFRFFATIDKVKPAASLDRKKENAPGDSTPAIAAQTFTFRELAAATKNFKAECLLGE
GGFGRVYKGRLENSGQVVAVKQLDRNGLQGNREFLVEVLMLSLLHHPNLVNLIGYCADGDQRLLVYEFMPLGC
LEDHLHDIPPEKAPLDWNTRMKIAAGAAMGLEYLHDKANPPVIYRDFKSSNILLDNNFHPKLSDFGLAKLGPVG
DKTHVSTRVMGTYGYCAPEYAMTGQLTLKSDVYSFGVVLLELITGRKAIDNSRPAGEHNLVAWARPLFKDRRKF
PSMSDPLLQGRYPMRGLYQALAVAAMCLQEQAGTRPLIADVVTALNYLASQTYDPGDKEKERKLSLATPAAAA
AAGDDRGKNNGVHSPSVTAKSSAKSSPNVRVKDEDSPRNLLEEAKLHDSSKQREADDWEMYDRNSMSPSRTN
KIFKEGFKILDHDRERAVAEARIWGESYRERKRGNTQGGGGGGGGSGGGSGGGGGGGTDC

>A072|A0A4V4H8L3

MGCFPCFGSASQREEEEVKKKNEGKAGGAYQKDSSAATASNHGSIDKSKSQMGSDTSKKASTPKECNADHIAA
KTFTFRELAAATQNFRQDCLLGEGGFGRVYKGRLENGQVVAVKQLDRNGLQGNREFLVEVLMLSLLHHLHLVN
LIGYCADGDQRLLVYEFMPLGSLEDHLHDIPADKEPLDWNTRMKIAAGAAKGLEYLHDKANPPVIYRDFKSSNIL
LGEGYHPKLSDFGLAKLGPVGDKTHVSTRVMGTYGYCAPEYAMTGQLTVKSDVYSFGVVFLELITGRKAIDNTRP
AGEQNLVAWARPLFKDRRKFPQMADPLLQGHYPARGLYQALAVAAMCLQEQAATRPLIGDVVTALSYLASQT
YDPNSVTGQSTRFGPSTPRSRSGKDHQHAVHSPQRNSPDLRQRDPVKGLSKGAKVGRGGSGGGSGQKWGLE
ESETQESQMDSPVHTEGARDSPKNIHRDLVREHAVAEAKLWGEKWRERQRRNAPDSFESTHE

>A073|J3LXC0

MGCFPCFDSRLEEEEEVCYRKGAGGGGNGAAASSSGVGGGGGGGGGGGGGGGGEGPECAXXXAPRVERISA
AAGACRSGKGNATVKELSALKDANGNVISAQTFTFRQLAAATRNFREECFIGEGGFGRVYKGRLDGTGQVVAIK
QLNRDGTQGNKEFLVEVLMLSLLHHQNLVNLVGYCADGDQRLLVYEYMPLGSLEDHLHDLPPDKVPLDWNTR
MKIAAGAAKGLEYLHDKAQPPVIYRDFKSSNILLGEGFHPKLSDFGLAKLGPVGDKSHVSTRVMGTYGYCAPEYA
MTGQLTVKSDVYSFGVVLLELITGRKAIDSTRPNVEQNLVSWARPLFNDRRKLPKMADPGLEGRYPMRGLYQA
LAVASMCIQSEAASRPLIADVVTALSYLASQKYDPNATPSSKKVGGGGGGCEGGRAALSRNDEAGSSGHKSPSS
KDSPRGPGPGPGPLPGILNDRERMVAEARMWGENWREKRRAATTGNSSSAQGSLDSPTETG

>A074|I1HV01

MSCFSCFGSAQAAAAAGEAEKPGHARKDGAAADRGAAPHKGGSDKVRPQGGSDSKKDHLTIPRDANSQNIA
AQIFTFRELATATKNFRQDSLLGEGGFGRVYKGRLDNGQAVAVKQLDRNGLQGNREFLVEVLMLSLLHHTNLV
NLIGYCADGDQRLLVYEFMPLGSLEDHLHDVPPDKEPLDWNTRMKIAAGAAKGLEHLHDKASPPVIYRDFKSSN
ILLGEGFHPKLSDFGLAKLGPVGDKTHVSTRVMGTYGYCAPEYAMTGQLTVKSDVYSFGVVFLELITGRKAIDNT
KPQGEQNLVAWARPLFKDRRKFPKMADPMLQGRFPMRGLYQALAVAAMCLQEQATTRPHIGDVVTALSYLA
SQTYDPNTPVQHNRSNSSTPRARNVVRRNEDPRSARSPNHHSPDLRREAARASKYGAEVSRTSSAGDSGRRSG
LDDMDMAGTQVGSPAQTGRKRETPRTADRQRAIADAKSWGQNSRERKLSNGHGSFDSSTNE

>A075|A0A4Y7IXE0

MVGCFSCLGPSDKGKNKGVKESSKKDGSGPQSRNTSKVSSEQHESQGGGVAKKEKLLPVDGLTPNIAAHTFKY
QELAAATNNFKEECVLGEGGFGRVYKGYLESTDQVVAVKQLDRDGLQGNREFLVEVLMLSLLDHSNLVKLIGYC
ADGDQRLLVYELMPLGSLDDHLYDLPPNKEPLNWITRMKIAEGAAKGLEYLHDKANPSVIYRDLKASNILLGEGF
YPKLSDFGLAKLGPTGDNTHVSTRVMGTYGYCAPEYAMTGQLTLKSDVYSFGVVLLELITGRKAIDNSRAVKERN
LVAWARPLFRDRRMFPEMADPLLQGRYPRRGLYQALAVAAMCLQEQAVSRPQIAEVVTALSYLTSQTNAAWD
KNKAGPSSNPSTMHIDKESGNEVGNGRENNVFESPRRNSPNLRHNREAGYEQGMKLRLNKSEEQSSHQDSSS
NLPKARETVKSPNNRDLDREHAVAEAKLWGENLRGRKRGNSVGNSFDSTNGTIM

>A076|A0A4Y7JEV9

MGCFPCFDSKEEEKLNPGNKEESSDNVRKEVHHNHNNQPVVNPNVAKLSSGADRLRSRSTLGGSRRESSGLKE
GPYDHIAAQTFTFRELAAATKNFRPESLLGEGGFGRVYRGFLESTGQAVAVKQLDKDGLQGNREFLVEVLMLSLL
HHQNLVSLIGYCADGDQRLLVYEFMCLGSLEDHLHVFLREIRIDLRVDLPPGKEALDWNTRMKIAAGAAKGLEYL
HDKANPPVIYRDFKSSNILLGEGFHPKLSDFGLAKLGPTGDKSHVSTRVMGTYGYCAPEYAMTGQLTVKSDVYSF
GVVFLELITGRKAIDSTQGHGEQNLVQWARPMFNDRRKFPKLADPRLEGRFPMRGLYQALAVASMCIQESAAT
RPLIGDVVTALSYLASQSYDPSAPPSNKANGDKEDRRTSNERGGRIGKNEEGGGSGRKWDLEGSEKDDSPRETQ
RMLNRDLDRERAVAEAKMWGENWREKKRQSAQGSFDGVNG

>A077|A0A2I0XBR2

MGCFPCFGSSENRRDKKVDVEGGKGGFRNEAASALSTSQVMRVNSDKSKSRNGSESKKEALVPKEGNTAHIAA
QTFSFRELAAATKHFRPECLLGEGGFGRVYKGRLENGQIVAVKQLDRNGLQGNREFLVEVLMLSLLHHQNLVNLI
GYCADGDQRLLVYEYMPLGSLEDHLHDLPPNKVPLDWNARMKIAAGAAKGLEYLHDKASPPVIYRDFKSSNILL
GEGYHPKLSDFGLAKLGPVGDNTHVSTRVMGTYGYCAPEYAMTGQLTLKSDVYSFGVVFLELITGRKAIDNTRA
AGEQNLVAWARPLFKDRRKFPKMADPLLQGHYPMRGLYQALAVAAMCLQEQAATRPLIGDVVTALSYLASQT
YDPNAPLPHTSRIGPSTPRLREDGKNLLIGLESKHSTDSPKLRQRDSVKVEAVGELESRQDSPSYSPVLLGKPRDSD
PDRERAIAEAKVWGENWRERKKGNAGNFDSTYEEEEEDY

>A078|A0A2I0B7M0

MRCSFPCFDWNDGVVDFDSGDRRQDEKREEKPMMPPTIDRLPLGAESLNNRVSLSSKKEPPNGFNKELYDELE
KLQLDDLNGELLSQNHSSSSDFSARTFTFRELAAATKNFRQECFLGEGGFGSVYKGCVDTGEVVAVKRLDRNGL
QGNKEFLVEVLMLSLLHHSNLVNMIGYCADGDQRLLVYEFMPLGSLEDHLHDLPPDREPLDWNTRMKIAASAA
KGLDYLHNEASPPVIYRDLKSANILLDEEFHPKLSDFGLAKLGPTGDKSHVSTRVMGTYGYCAPEYAMTGQLTAK
SDVFSFGVVLLELITGRRAFDNNRAHGEQNLVSWARPLFSDRRRLSKLADPKLEGRYPMRGLYQALAVASMCIQ
EHPAARPLVSDIVIALSFLASHPYNPSTHPSSTPRNGEADKDRPREGERIEEVSVDVQKLSPTEREDSPRETAAFLN
RDIDRERAVAEAKMWGANWREKRRAGNVDPAPASSNE

>A079|A0A1E5VW28

MGCFPCFGSGREEEELKYYGGKGGGGGNGGHAGWAAAAASSSAAAAAAGGGAEAAVAAAPRAERFPRGAD
KVRGKGNAGWKKELAGIKDANGNAISAQTFTFRQLAAVTKNFRDECFIGEGGFGRVYKGRLDTGQVVAIKQLN
RDGNQGNKEFLVEVLMLSLLHHQNLVSLVGYCADGDQRLLVYEYMPLGSLEDHLHDLPPGKKPLDWNTRMKI
AAGAAKGLEYLHDKAQPPVIYRDFKSSNILLGEGFHAKLSDFGLAKLGPVGDKSHVSTRVMGTYGYCAPEYAMT
GQLTVKSDVYSFGVVLLELITGRKAIDSTRPATEQNLVSWARPLFNDRRKLPKMADPGLEGRFPMRGLYQALAV
ASMCIQSEAASRPLIADVVTALSYLANQIYDPNAANTSKKGGGGIGDQRSRVSDSGRALSKYDDTGSSGHRSPSR
DDSPRDANKGLERERMVAEAKTWGENWREKQRAAQGSLDSPTGGG

>A080|A0A200Q1K2

MGGCFPCLGPSKKEKRQGVKESVKKDFVKNGSGPQSRTTSRVSSDELISQSGAGSKKETSIPEGGPTPHIAAQTF
KFQELAVATNNFTEECLLGEGGFGRVYKGFLESTGQVVAVKQLDRNGLQGNREFLVEVLMLSLLDHSNLVNMIG
YCADGDQRLLVYELMPLGSLEDHLHDLPPNKEPLDWNTRMKIAEGAAKGLEYLHDKANPPVIYRDLKSSNILLDE
GFHPKLSDFGLAKLGPVGDNTHVSTRVMGTYGYCAPEYAMTGQLTVKSDVYSFGVVFLELITGRKAIDNSRPHK
ERNLVAWARPLFKDRRNFAEMADPLLQGHYPKRGLYQALAIAAMCLQEQAVSRPLIGEVVTALSYLVSQTYVQK
NEVGPSTTTTKDSKEIQDEVGNSGENGSRPPHQNSPNFRDRCETGRESGMKLGLDELEGQNSQRDSPGNLLKA
RETLGNSNRNLDREQAVAEAKLWGENWRGRKRGNSMG

>A081|A0A1J7GYH1

MGCFSCFESLEDEKLNTHSEQQYQHQQQQQQQQQTNHNLRKHQPPTPPHISRMPSDKSQSRTNRDSKREE
PAPNAIHIPGVQIAAQTFTYRELAAATKNFRPESFIGEGGFGCVYKGWLESTNQIVAVKQLDRNGLQGNREFLVE
VLMLSLLHHTNLVNLIGYCADGDQRLLVYEFMPLGSLEDHLHESFFMLVNELFLQDLPPDKEPLDWNTRMKIAA
GAAKGLEYLHDKANPPVIYRDLKSANILLDEGFHPKLSDFGLAKLGPVGDKSHVSTRVMGTYGYCAPEYAMTGQ
LTVKSDVYSFGVVLLELITGRKAIDSNQPHGEQNLVSWACPLINDRRKLSKLADPKLHGRLPMRGLYQALAVASM
CIQESAAARPLIGDVVTALSYLASQAYDPKGSSGDDKRNKDDKGGRILKNDEAGGSGLKWDLEGSEKDDSPRET
ARMIDRERAVAEAKLWGENLREKRRQSAPPGNFNDSNT

>A082|P0625E02.115

MSCFPCSGSSGKGGVDAKSVAALSPGPRPAASAAPDRSNSSRGSGIKKDDSVRRGGSSANDGPAKIFTFRELAV
ATKNFRKDCLLGEGGFGRVYKGQMENGQQVIAVKQLDRNGLQGNREFLVEVLMLSLLHHPNLVRLIGYCADG
DQRLLVYEYMLLGSLENHLHDRPPGKKPLDWNARMKIAVGAAKGLEYLHDKANPPVIYRDFKSSNILLGEDYYPK
LSDFGLAKLGPVGDKTHVSTRVMGTYGYCAPEYAMTGQLTVKSDVYSFGVVFLELITGRKAIDHTQPAGEQNLV
AWARPLFRDRRKFCQMADPSLQGCYPKRGLYQALAVASMCLQENATSRPLIADIVTALSYLASNHYDPNAPSAK

SSRTCPSTPKAKAHRRTTSVPDAQHAADSLNWNFPDLGRKETTRGEFEQDHSEGYGSGSSSGRNDGLDVPELLA
LHNGQNNSEADIYHKSSVKLDAHEKQRSGSGKGSRQF

>A083|LOC109726516

MGCFPCFDSREEEQLNPKSGGCGVGGVEEEAKSGERPMIPPQIEKLSSGTDILKIRSNLGLKREPSGQKDGSGVN
ISAQTFTFRELTAATKNFRQECFLGEGGFGRVYKGRLESTGQIVAIKQLDRNGLQGNREFLVEVLMLSLLHHPNLV
NLIGYCADGDQRLLVYEYMPLGSLEDHLHDLPLDKEPLDWNTRMKIAAGAARGLEYLHDKANPPVIYRDFKSSNI
LLDEGFHPKLSDFGLAKLGPVGDKSHVSTRVMGTYGYCAPEYAMTGQLTVKSDVYSFGVVLLELITGRKAIDSTR
PHGEQNLVSWALQARPLFNDRRKLPKMADPRLDGRYPMRGLYQALAVASMCIQSEAASRPLIADVVTALSYLA
SQTYDPSTAHHSSSNRSSGERRGGRFLNDDKVGKLPSRTDSPKDSNGDREDSPKEAPGILNKDFDRERAVAEAK
MWGENWREKRRAIANNEKQGANAADSPTKSG

>A084|A0A5P1ECE0

MMGCFPCFGSTKGSKIEVNSSQQGGGDLKKEASVAPSSDKSKSQSGSESKNEALVTKEGNATRIAARTFTFRELA
AATQNFRQECLIGEGGFGRVYKGRLEDGQVVAVKQLDRNGLQGNREFLVEVLMLSLLHHPNLVNLIGYCADGD
QRLLVYEFMPLRSLEDHLHDLPSDKEPLDWNTRMKIAAGAAKGLEYLHDKANPPVIYRDLKSSNILLGEGYHPKL
SDFGLAKLGPVGDKTHVSTRVMGTYGYCAPEYAMTGQLTLKSDVYSFGVVLLEIITGRKAIDSTRPTGEQNLVA
WARPLFKDRRKFPKMADPLLQGRYPMRGLYQALAVAAMCLQEQAATRPLIGDVVTALTYLASQSYDPNAAPP
QRNRASLHALRTKEESRGLGGGFDSPRANDSPRQNSPGFRYNRKETLEEPEAHESHNDSPKNFGKAVESRRSLK
DIDRERAVAEAKLWGENSRERRRSNAAGSYEGTNE

>A085|A0A0K9PFH2

MGCFHFPCFNSKMTTELKNPANGSVDRRHQPMVPPEVHRLTSGADRVKNRSNGGVGGSKRMSDAQISGVKI
SAQIFTFRELATATKNFRPECFIGEGGFGHVYKGQLESTGQVVAVKRLDRDGLQGNREFLVEVLMLSLLHHSNLV
NLIGYCADGDQRLLVYEFMPLGSLEDHLHDLPPDKEPLEWNVRMKIAAGAAKGLEYLHDEANPPVIYRDFKSSNI
LLDNGFHPKLSDFGLAKLGPVGDKSHVSTRVMGTYGYCAPEYAMTGQLTVKSDVYSFGVVFLELITGRKAIDSTR
PHSEQNLVAWARPMFNDRRRLGKLADPMLQGRFPMRGLYQALAVASMCIQEEAAARPLIADVVTALSYLASQ
ANDQNAADNDAQRGRARREERNNNNVLSRVMVRNGSEEGSQRGGSGRSRFDLEGSEKEDSPRDTIRMNLN
NKDFDRERAVAEAKMWGENLRRANGQGATTTATAGVANT

>A086|A0A2I0X7U8

MPCFFPCFDWNDEAVEFVGGSRQQDETREEKPMMPPSVDRLPAGNGGLNSRNSFDTKRELVNGFKREQVNV
SEKRQLGGLKLEALNQNKSSGSDFSARTFTFRQLAAATKNFRQECFLGEGGFGSVHKGRLETGEIVAVKQLDRN
GLQGNKEFLVEVLMLSLLHHPNLVNMIGYCADGDQRLLVYEYLPLGSLENHLHDLPPDKEPLDWNTRMKIAAG
AANGLDYLHNVASPPVIYRDLKSANILLDADFQPKLSDFGLAKLGPTDDKSHVSTRVMGTYGYCAPEYAMTGQL
TVKSDVFSFGVVLLELITGRRAFDSTRAHGEQNLILWVKPLFNDRRRISKLADPTLQGRYPMRGLYQALAVASMC
IQEHPAARPSVTDIVTALSYLASHPYDPSLQGGEVENGRRQEAERIKTYLDDQKVPPNERDDSPKDKGTFLNQNF
DRERAVAEAKMWGANWREKRLATVDPGLTNSNS

>A087|LOC104415649

MGCFPCFDSREEETLNPGKGSDDPKHGVPNVSSKISSLPSGVERLRSRSNLGLRRDLIGPKDVPGVHIAAQTFTFR
ELAAATKNFKPECFLGEGGFGGVYKGKLETTGQDKSCNHIEDSDAVRQVVAVKQLDRNGLQGNREFLVEVLML
SLLHHPNLMNLIGYCADGDQRLLVYEFMAFGSLEDHLFDLPLGKEPLDWNTRMTIAAGAAKGLEYLHDKANPP
VIYRDFKSSNILLDEKFHPKLSDFGLAKLGPVGDKSHVSTRVMGTYGYCAPEYAMTGQLTVKSDVYSFGVVFLELI

TGRKAIDSSRPHGEQNLVTWARPLFNERRKFSKLVDPSLQGRYPMRGLYQALAVASMCIQEQAAARPLIGDVV
TALSYLANQAYDPNVASNFGNKGSSVDERGSKILRNEDAGGSGRSRWEFEGSEKDNSPKDGKLLNRDLNRERA
VAEAKMWGENWREKRRQSAQGSFDGSNGRLC

>A088|A0A200QGH7

MGCFPCFDSKEEEKLNPGKEREDRKEIQPAVNPNVARLSSGADRLKSRSTHGGSRRESSGPKDGPDAHIAAQTF
TFRELAAATKNFRPESLLGEGGFGRVYRGHLESTGQQAVAVKQLDKDGLQGNREFLVEVLMLSLLHHPNLVNLI
GYCADGDQRLLVYEFMPLGSLEDHLHDLPPDKEALDWNTRMKIAAGAAKGLEYLHDKANPPVIYRDFKSSNILL
DEGFHPKLSDFGLAKLGPVGDKSHVSTRVMGTYGYCAPEYAMTGQLTVKSDVYSFGVVFLELITGRKAIDSTQA
HGEQNLVAWARPMFNDRRKFPKLADPRLQGRFPMRGLYQALAVASMCIQEQAATRPLIGDVVTALSYLASQS
YDPNAPQGHSSYRGNGDKEDRRNRVGGSTDDRGGRIGKNEEGGGSGRKWDLDGSEKEDSPRETARMLNRDL
DRERAVAEAKMWGENWREKRRQSAQGSFDAANG

>A089|A0A1J7FPP8

MGCFSCFDSMEDEKLNQNHHQQQQQQQQTHQQHHHHHHNNNIRNPNPSHISTMPSGADKLRSRSNG
GSKREQPSPNGIHIPGVQIAAQTFTFRELAAATKNFRPESFIGEGGFGRVYIGRLESTRQTVAVKQLDRNGLQGN
REFLVEVLMLSLLHHPNLVSLIGYCADGDQRLLVYEFMPLGSLEDHLHDLPPDKEPLDWNTRMKIAAGAAKGLE
YLHDKANPPVIYRDFKSSNILLDEGFQPKLSDFGLAKLGPVGDKSHVSTRVMGTYGYCAPEYAMTGQLTVKSDVY
SFGVVFLELITGRKAIDSTQPHGEQNLVTWARPLFNDRRKFSKLADPKLQGRFPMRGLYQALAVASMCIQESAA
TRPLIGDVVTALSYLANNAYDPKGSGGDDKRNKDDKGGRILKNDEAGGSGRRWDLEGSEKEDSPRETARMLDR
ERAVAEAKLWGENLREKRRQSALPGSFDGSNP

>A090|A0A1U7ZAQ8

MGCFSCFDSREEEEMNLEKGRDDKMERQQTENSNVSRLSSGADRLKSRGNTGSKRESSGSKDGPDVNIAAQTF
TFRELATATRNFRQECLLGEGGFGRVYKGRLESTGQVVAVKQLDRDGLQGNREFLVEVLMLSLLHHPNLVNLIG
YCADGDQRLLVYEYMPLGSLEDHLHDLPPDKEPLDWNTRMKIAAGAAKGLEYLHDKANPPVIYRDFKSSNILLD
EGFHPKLSDFGLAKLGPVGDKSHVSTRVMGTYGYCAPEYAMTGQLTVKSDVYSFGVVFLELITGRKAIDSTRAHG
EQNLVTWARPMFNDRRKFSKLADPRLQGRYPMRGLYQALAVASMCTQEQAATRPLIADVVTALSYLASQTYD
PNAPPGHSYRGSGDERRNRGAGAERDDKGGKIANNEDGGGFGRRWDQEGSERDDSPRETARMLNRNLDRE
RAVAEAKMWGENWREKRRQSAQGSFDSANG

>A091|A0A2I4DXF9

MGCFPCFDTKEEGKLNPVKESDGRKQGQPMVPSAISGLPSGADRLKSRSNAASKGELPGPKDGPGIHPGVHIAA
QTFTFRELAAATMNFRPESFIGEGGFGRVYKGRLETTGQIVAVKQLDRNGLQGNREFLVEVLMLSLLHHPNLVN
LIGYCADGDQRLLVYEFMPLGSLEDHLHDLPPDKEPLDWNTRMKIAAGAAKGLEYLHDKANPPVIYRDFKSSNIL
LDEGYQPKLSDFGLAKLGPVGDKSHVSTRVMGTYGYCAPEYAMTGQLTVKSDVYSFGVVFLELITGRKAIDGTRP
HGEQNLVAWARPLFNDRRKFSKLADPWLQGRYPIRGLYQALAVASMCIQEQAATRPLIGDVVTALSYLANQAY
DPKTASGHSHKGSGDRDERRNRDDRGGRISKNEESGGSGRRWDFEGSEKDDSPKETAKMLNRDLIRERAVAEA
KMWGENWREKRRQSAQGSFDGNNG

>A092|A0A2I4G517

MGCFPCFDSKEEEKLNPEKVSDDRKQVRPMVPSAFSGLPSGADRFKSRSNVGYKKESLGPKDGPGVHPGVNIA
AQTFTFRELAAATKNFRPESFLGEGGFGRVYKGRLETTGQIVAVKQLDRNGLQGNREFLVEVLMLSLLHHPNLV
NLIGYCADGDQRLLVYEFMSMGSLEDHLHDLPPDKEPLDWNTRMRIAAGAAKGLEYLHDKANPQIIYRDFKSSN

ILLDEGYQPKLSDFGLAKLGPVGDKSHVSTRVMGTYGYCAPEYAMTGQLTVKSDVYSFGVVFLELITGRKAIDSTR
PHGEQNLIAWARPLFNDRRKFSKFADPLLQGRYPMRGLYQALAVASMCIQEQAAARPLIGDVVTALSYLANQA
YDPNTTYAHGHRGSGDKDERRDRDERGGRILKNEEGGGSGHRWDLEGSEKEDSPRETARMLNRDLDRERAVA
EAKMWGENWREKRRQIAQGSFDGKNA

>A093|A0A2G5E5E5

MGCFPCFDSNSREEEELNPVKVRDDRKEPPPILNTNISRLSSGADRLKTRSALGGSKRESPTAKDGPGSIAAQTFT
FRELAAATKNFRPECLLGEGGFGRVYKGRLESTGQAVAVKQLDRDGLQGNREFLVEVLMLSLLHHPNLVNLIGY
CADGDQRLLVYEFMPLGSLEDHLHDLPPDKEPLDWNTRMKIAAGAAKGLEYLHDKANPPVIYRDFKSSNILLGD
GFHPKLSDFGLAKLGPTGDKSHVSTRVMGTYGYCAPEYAMTGQLTVKSDVYSFGVVFLELITGRKAIDSTQPHGE
QNLVVWARPMFNDRRKFPKLADPRLQGRYPMRGLYQALAVASMCIQEQSATRPLIGDVVTALSYLANQSYDP
STAPGYRGLGDKDDRRRSGGVDEKGIRIVKNEEGGGSGRKWDLEGSEKDDSPKETARMLNRALDREKAVAEAK
MWGENWRAQRRQSAQGSFDGTNG

>A094|103999349

MGCFPCFESKEGAQLGRGNESDRKREEQPMVAPRVEKLSLGNDDRKRTPNSLNTQKESLGEKRGAEFSISAQTF
TFRELAVATGNFRSECFLGEGGFGRVYRGRLESTGQVVAVKQLDRDGLQGNREFLVEVLMLSLLHHPNLVNLIG
YCADGEQRLLVYEFMPLGSLEDHLHDLPPDKSPLDWNTRMKIAAGAAKGLEYLHDKANPPVIYRDLKASNILLDK
GFHPKLSDFGLAKLGPVGDKSHVSTRVMGTYGYCAPEYALTGQLTVKSDVYSFGVVLLELITGRKAIDSTKTHAE
QNLVSWVRPMFKERRKLPGLADPKLQGRFPMRGLYQALAVASMCIQEEAASRPVIADVVTALSYLASQAYDPG
AASAVNTRPSGERTSRSGGEEGAKLTVRVNNIDPGHMQQMISEDSSKETVAILRHNFDRERALAEAKMWGQN
WREKTQAKANVEGSFDVANAIG

>A095|A0A2Z7CGC2

MVKEDNGDIKGVTDQSKSRSGNESKKEAIIPKEPMAHIAAQTFTFRELAAATKNFRPECLLGEGGFGRVYKGQLE
STGQLVAVKQLDRNGLQGNREFLVEVLMLSLLHHQNLVNLIGYCADGDQRLLVYEYMPLGSLEDHLHDLRPDKE
PLDWNTRMKIAAGAAKGLEYLHDKANPPVIYRDLKSSNILLDEGYFPKLSDFGLAKLGPVGDKTHVSTRVMGTY
GYCAPEYAMTGQLTLKSDVYSFGVVFLEIITGRKAIDNNKGAGEHNLVAWARPLFKDRRKFPKMADPLLQGQYP
IRGLYQALAVAAMCLQEQAATRPLIGDVVTALTYLASQTYDHNALVSQGNKIGPSTPTSRLRPDQRNISDGTNNL
DESDTHGSPSSHIQSPNFRREFNNGAELRRFETGCGSGRKWGLEESEQPDSQRDSPGGKARGTPRNRDLDRER
AVAEAKVWGENWRERKRMNE

>A096|A0A199W2Y9

MSCLPCCGSREEEEEETKGGSGGGGKGGGDLKKEASGDASSRRVSRIGSDKSKSRGGSDSNKEGSVRKDGSNTH
IAAQTFTFRELAAATKNFREDCLLGEGGFGRVYKGHLENGQVVAVKQLDRNGLQGNREFLVEVLMLSLLHHPNL
VNLIGYCADGDQRLLVYEFMPLGSLEGHLHDVPPDKEPLDWNTRMKIAAGAAKGLEYLHDKANPPVIYRDFKSS
NILLGEGYHPKLSDFGLAKLGPVGDKTHVSTRVMGTYGYCAPEYAMTGQLTVKSDVYSFGVVFLELITGRKAIDN
TRPTGEQNLVAWARPLFKDRRKFAKMADPLLQGHFPMRGLYQALAVAAMCLQEQAATRPLIGDVVTALSYLA
SQTYDPNAHNNRTGPSTPRARSHHRTISGQSDNSHYTVNSPSQNSPDVRGSPLRMERARDATRNVNRQRTLA
ETKENWRERKRGSAPGSFDSTDE

>A097|A0A5J5AKL4

MGCFPCFDSREEEKLNPVKQRDDRNDGQPAVASNISRLSSGADRLKSRSNVGSRKGSLGPKDAPDVPIAAQTFT
FRELAAATKNFRPESFLGEGGFGRVYKGQLDSSGQVVAVKQLDRNGLQGNREFLVEVLMLSLLHHPNLVNLIGY
CADGDQRLLVYEFMPMGSLEDHLHDLPPDKEPLDWNTRMKIAAGAAKGLEYLHDKANPPVIYRDFKSSNILLDE
GYHPKLSDFGLAKLGPTGDKSHVSTRVMGTYGYCAPEYAMTGQLTVKSDVYSFGVVFLELITGRKAIDSARSQGE
QNLVTWARPLFNDRRKFAKLADPRLQGRYPMRGLYQALAVASMCIQEQAAARPLIADVVTALSYLANQAYEPN
TGLGHSYRFAGDKDDKRNKDERVGRISKNEEGGGSGCKWDLEGSEKDDSPRETARMLNRDLDRERAVAEAKM
WGENWREKRRQNAQGSFDGSNG

>A098|F6I1N7

MGCFPCFDSREEEKLNPDKGRDDRRESPQMVASNISRLSSGADRLKARNSTGLKKESSGPKDVPDGHIAAQTFT
FRELAVATKNFQPESFIGEGGFGRVYKGRLESTGQVVAVKQLDREGLQGNREFLVEVLMLSLLHHPNLVNLIGYC
ADGEQRLLVYEFMPLGSLEDHLLDLPPDKEPLDWNTRMKIAAGAAKGLEYLHDKASPPVIYRDFKSSNILLEEGF
HPKLSDFGLAKLGPVGDKSHVSTRVMGTYGYCAPEYAMTGQLTVKSDVYSFGVVFLELITGRKAIDSTLPHGEQ
NLVTWARPLFNDRRKFAKLADPRLHGRYPMRGLYQALAVASMCIQEQAATRPLIGDVVTALSYLANQSYDPNA
AHGHIYRGSGDRDERRNREERSARVTRNEDGGGSGRRWDLEGSEREDSPRETARMLNRDLDRERAVAEAKM
WGENWREKRRQSAQGSFDGTNG

>A099|111375917

MGCFPCFDSRDEEKLNPQKERDDHREVQPNISSNISRLSSGADRAKLRSNVGPRKEPSGPKDAPVAQIAAQTFTF
RELAAATTNFRPECFLGEGGFGCVYKGHLPSGQVVAVKQLDRNGLQGNREFLVEVLMLSLLHHPNLMNLIGYC
ADGDQRLLVYEFMPLGSLEDHLLDLPPDKEPLDWNTRMKIAAGAAKGLEYLHDKANPPVIYRDFKSSNILLGEGY
FPKLSDFGLAKLGPTGDKSHVSTRVMGTYGYCAPEYAMTGQLTVKSDVYSFGVVFLELITGRKAIDGTRPHGEQ
NLVAWARPLFNDRRKFAKLADPRLQDKYPMRGLYQALAVASMCIQEQAASRPLIGDVVTALSYLANQKYDNSI
APGHICRFSGDKDDNRNKDERGGKILRNEEGGGSGRKWDLEGSEKDDSPKETTKMLNRDLHRERAVAEAKM
WGENWREKRRQNAQGSFDSSNG

>A100|103976152

MGCFPCFESPEEEDKKKRSEVGGGGDAKAETSAGVRFDKSKPRNVSDSKKEASAPKEENAGLIASRTFTFHELAA
ATRNFRQDCLLGEGGFGRVYKGMLDDGQVVAVKQLDLNGLQGNREFLVEVLMLCLLHHSNLVNLVGYCADG
DQRLLVYEFMPLGSLQDHLHDIPPYKEPLDWNTRMKIAAGAAKGLEYLHDKADPPVIYRDFKSSNILLGEGYHPK
LSDFGLAKLGPVGDKTHVSTRVMGTYGYCAPEYAMTGQLTVKSDVYSFGVVFLELITGRKAIDVTRPAGEQNVV
LWARPLFKDRRKFPKMVDPLLQGCYPIRGLYQALAVAAMCLQEQAATRPLIGEVVTALSYLASQSYDPNATAVQ
NAGICSSTQRVREDKRLLGGRFVDEHVVHSPHHDSSDFRHRHQEEEVCTRVDVGRARGSPKNLSNGLVREGVV
TEAKVWGKNLTEKEDRSAWY

>A101|103986028

MGCFPCFESRRGGVLNPGNGRDDSREEHPMVPPHIDRSSSVADSVNHRSNSGSKREAQSEKDVSSVNISAHTF
TFRELATATRNFRDECFLGEGGFGRVYKGRLESTGQIVAVKQLDREGLQGNREFLVEVLMLSLLHHPNLVNLIGY
CADGDQRLLVYEFLPLGSLEDHLHDLPPDKEPLDWNTRMKIAAGAAKGLEYLHDKASPPVIYRDFKSSNILLGEGF
HPKLSDFGLAKLGPTGDKSHVSTRVMGTYGYCAPEYAMTGQLTVKSDVYSFGVVLLELITGRKAIDSALPHGEQN
LISWARPMFNDRKKLPKLADPRLQGRFPMRGLYQALAVASMCIQEQAASRPGIADVVTALSYLANKAYDPNAS

PASSNRSGGDHDERRIRGGSGMITKNEEVGDSGRKRELEGSEKEDSPREMAGIFKKDFDRERAVAEAKMWGE
NWRGKTRASSRNASSANS

>A102|A0A059D9V6

MGCFSCFDSREDVKLNPEKESDDRNQGPLSSDISRLASGVDRPRLRGNAGVKRDLPGLKDTPGIAAQTFTFRELA
AATKNFRSECFLGEGGFGRVYKGRLESTGQVVAVKQLDRNGLQGNREFLVEVLMLSLLHHPNLVNLIGYCADGD
QRLLVYEYMPLGSLEDHLHDLPPGKEPLNWNMRMKIAAGAAKGLEYLHDKANPPVIYRDFKSSNILLEEGFHPK
LSDFGLAKLGPTGDKSHVSTRVMGTYGYCAPEYAMTGQLTVKSDVYSFGVVLLELITGRKAIDSNLPHGEQNLVA
WARPLINDRRKFSRMVDPSLQGQYPMRGLYQALAVASMCIQEQAVARPLTRDVVTALSYLANQAYDPNTAAG
HAYKGSGGVKEGKSSGDDRGIKILRNEEGEGSGRRWDFEGSSEKDDSPRDTVRMLNKDLDRERAVAEAKMWG
ENWREKRRQSAQGSFDGSNS

>A103|A0A2H3WYF2

MGCFPCFDSKEAAQLNPGNRAGDRREEQPMVPPRVDKLSSGADRMETRSNLDLKRESLGPREESGFNISAQTF
TFRELAAATKNFRPECFLGEGGFGRVYKGRLESTGQVVAIKQLDRNGLQGNREFLVEVLMLSLLHHPNLVNLIGC
CADGDQRLLVYEYMPLGSLEDHLHDLPPGKEPLDWNRRMKIASGAAKGLEYLHDKANPPVIYRDFKSSNILLDE
GFHPKLSDFGLAKLGPVGDKSHVSTRVMGTYGYCAPEYAMTGQLTVKSDVYSFGVVLLELITGRKVIDSTRAHAE
QNLVSWARPMFNDRRKLPKLADPKLQGRYPMRGLCQALAVASMCIQEEAASRPLIADVVTALSYLSSQAYDPS
AGPNYNSRYGGERSGGNEKGGRMLVKNEGSGHERGLDMEKEDSPREVVGILNRDFDRERAVAEAKMWGEN
WREKRRANANLQESYDAASGNG

>A104|A0A2H3YUK1

MGCFPCFDSSDGGVLDTGDGRDDKREEQPMVPPRVDRVSWVADSLRSKNNSESERETQNRKDGSDVTIEAQT
FTFRELAAATENFRPECLIGEGGFGRVYKGYIESIGKVVAVKQLDKNGLQGNREFLVEVLMLSLLHHPNLVNLIGY
CADGDQRLLVYEFMPLGSLEDHLFDLPPDKEPLDWNTRMRIATGAAKGLEYLHDKANPPVIYRDFKSSNILLDEG
FHPKLSDFGLAKLGPVGDKSHVSTRVMGTYGYCAPEYAMTGQLTVKSDVYSFGVVLLELITGRRAIDSTRPHGEQ
NLVSWARLMFNDRRKLPKLADPKLQGRYPMRGIYQALAVASMCIQEQAASRPLIADVVTALSYLASQAYDPNA
LHVSNNRSGREHHERSHQGGCGKVPAKNEEGGGSGQKFVEEREDAPRVVVGTLNKDFDRERAVAEAKLWGE
NWREKIRANGNQQGASNAN

>A105|A0A2Z7CE00

MGCLPCFDSKEEETLSPHKEGSDDRRAIYPSIPPNPPNLSSGADRLKTRSNIGVPEDSPHVQIFTFRELAIATKSFM
PECLLGEGGFGRVYKGRLLNGQVVAVKQLDRNGLQGNREFLVEVLILCLLHDRNLVNLIGYCAEGEHRLLVYEFM
PLGSLEDHLFDVPLDKEPLDWNTRMKIAAGAAEGLQYLHDKANPPVIYRDFKSSNILLGEGYFPKLSDFGLAKLGP
TGDKSHVSTRVMGTHGYCAPEYAMTGQLTVKSDVYSFGVVFLELISGRKAIDSNRPPGEENLVSWARPMLNDR
RKISNLADPRLKGKFPTRGLYQALTVVSKCIQEQAAGRPWIREVVTALLYLANLPYDASLASGPNRSCVAEKDDN
GSKDGRGGALMRKEEGGSGSGCKWNLEGFEIEREDSPRENKKMLNRLERERAVAEAKTWGDSWRLQNSRLN
FDGSNRRPASESMF

>A106|LOC103649959

MGFLACLCRCPQDGDDDEKEGEQFRINHQVASVDYPKSSESSPLKAERVVHMDGAQLIGRHDEATIFTLRQLAE
ATNNFRQDSLLGRGGFGCVYKATLSNGQVVAVKQLDLNGLQGNREFLVEVLMLNLLHHPNLVNLHGYCVDGD
QRLLVYEYMPLGSLEDHLHDLAPNQQPLDWKTRMKIAAGAAAGLEYLHDKANPPVIYRDIKPSNILLGEGYHAK
LSDFGLAKLGPVGDKTHVTTRVMGTHGYCAPEYALTGQLTVKSDIYSFGVVFLELITGRRPQDSDRPPEEQDLVA
WARPLFKDQKKFRKMADPSLCGRFPKRGLFQALAIAAMCLQEKAKSRPPMREVAAALSYLASQAYDRNNNAA
AHRNRAGPSTPRVLDDQIGQDTCTLANQQGAQMSMHVGTDHAMPEVKEASCSGSHRGGRGRVTPNGADR
ERALADANVWAEAWRRQEKASKMR

>A107|A0A1J7IXN4

MGWFSCFDPRDDEKLNSHPHQKQQQIHHHHLRKHHPPIPSHISKLPSGADKLRWRSNGGSKKEAPNTIHIPGV
QIAAQTFTFRELAAATKNFRPESFLGEGGFGSVYKGRLESSSQTVAVKQLDRNGLQGNREFLVEVLMLSLLHHPN
LVNLIGYCADGDQRLLVYEFMPMGSLEDHLHDLLPDKEPLDWNTRMKIAAGAAKGLEYLHDKANPPVIYRDFKS
SNILLDEGFHPKLSDFGLAKLGPVGDKSHVSTRVMGTYGYCAPEYAMTGQLTVKSDVYSFGVVLLELITGRKAIDC
TQSHGEQNLVTWARPLFNDRRKFSKLADPKLQGRFPMRGLYQALAVASMCIQESAATRPLIGDVVTALSYLAN
QAYDTKGSGGDDKRNKDDKGGRILKNDEAGGSGHRLDLEGSEKDESPRETARMSDRERAVAEAKLWGEKLRE
KRRQNALPGSFDGSNA

>A108|A0A3L6QHY7

MGCFSCFDSPADEQLNPKLGGAGGYGGASSAAAAYGGGSGGGRHGDRGYPDLQQAPMVAPRVEKLSAAAEK
ARVKSNALAREASAPKDANGNVISAQTFTFRELATATRNFRPECFLGEGGFGRVYRGRLESTGQVVAIKQLNRD
GLQGNREFLVEVLMLSLLHHQNLVNLIGYCADGDQRLLVYEYMPFGSLEDHLHDLPLDKEALDWNTRMKIAAG
AAKGLEYLHDKANPPVIYRDFKSSNILLDESFHPKLSDFGLAKLGPVGDKSHVSTRVMGTYGYCAPEYAMTGQLT
VKSDVYSFGVVLLELITGRRAIDSTRPHGEQNLVSWARPLFNDRRKLPKMADPRLEGRYPMRGLYQALAVASMC
IQSEAASRPLIADVVTALSYLASQSYDPNAALASRKPGGDQRSKPGENGRLVSRNDETSSSGHKSPGKDREDSPR
DLPGRFASRWRREGREG

>A109|A0A1Q3BTP5

MGCFPCFDSREEEQLNPDKEVDDRKQGQPMVSSNVSRLASGGDRLKTRSNGESRRELPGPKDVPGGAIAAQTF
TFRDLTAATKNFRQESFLGEGGFGRVYKGRLESTGQVVAVKQLDRNGLQGNREFLVEVLMLSLLHHPNLVSLIGY
CADGDQRLLVEYMPFGSLEDHLHDLPPDKETLDWNKRMKIAAGAARGLEYLHDKANPPVIYRDFKSSNILLDEG
FHPKLSDFGLAKLGPTGDKSHVSTRVMGTYGYCAPEYAMTGQLTVKSDVYSFGVVFLELITGRKAIDSTQPHGE
QNLVAWARPLFNDRRKFSRLADPQLRGRYPMRGLYQALAVASMCIQEQAATRPLIGDVVTALSYLANQAYDP
NSPGHAHRSSGDKQDKRNRDDRGGKLLKNDGGGGSGLRWDLESSEKDDSPKETARMLNRERAVAEAKMWG
ENWREKRRQTAQESFDVTNG

>A110|LOC18434449

MGCFPCFESKKEFDPANSRDGKKEGQPSAAPHVSRIPSGSDKLKARTGSGSRKEAAPPKDGPFAHIAAQTFTFKE
LAAATKNFRQECFLGEGGFGRVYKGRLESTGQVVAVKQLDRNGLQGNREFLVEVLMLSLLHHPNLVNLIGYCAD
GDQRLLVYEFMPLGSLDDHLHDLPPDKEPLDWNTRMKIAAGAAKGLEYLHDKANPPVIYRDFKSSNILLDEGYH
PKLSDFGLAKLGPVGDKSHVSTRVMGTYGYCAPEYAMTGQLTVKSDVYSFGVVFLELITGRKAIDSTRAPGEQNL
VAWARPLFNDRRKFPKMADPLLQGRYPMRGLYQALAVAAMCLQEQAATRPLIGDVVTALSYLASQSYDPNTA
SVQSSRVGREKDDRKGTRSEKGDERGGGIMKSEDEGGSGRKWEGADGFERDDSPRNSNRVSDRERAVAEAKV
WGENWRDKRRVSV

>A111|A0A2I0WGV2

MGCFSCFVSEEGGDSHLRGEEDGRKEARTMEAPRMKMLSSASSKIRSRNSMGSRKGSLSQKDATGVTISAQTF
TFRELSAATKNFRRDCFIGEGGFGRVYKGRLENSGQIVAIKRLDKDGLQGNREFLVEVLMFSLLHHQNLVNLIGYC
ADGDQRLLVYEFMPLGSLEDHLHYLPPDKEPLDWNTRMRIAAGAARGLEYLHDKANPPVIYRDFKSSNILLDEGF
HSKLSDFGLAKLGPVGDQSHVSTRVMGTYGYCAPEYAMTGQLTVKSDVYSFGVVFLELITGRKSIDNSKPHGEQ
NLIRWARPMLNDQRKLSKLADPTLQGRYPMRSLYQAVAVASMCIQEEAAARPLIADVVTALSYLESQTYDPKAV
AAYSHRLDRLSRGSREENPSRGNQEDGSKWGLCGIERDDTPKEIAGVINKDVERERAVAEAMMWGANWREK
QRASRHEALTL

>A112|A0A0K9PTD6

MGCCPCFDSKDAEELNPSKSLNEDTSPRGDEHPMVPPQVHRLVSGADRLKTRNNGEAKGRTPGSQLSSVKISA
QTFTFKELALATKNFTPECFLGEGGFGHVYRGCLSMTNQDVAIKQLNRNGLQGNREFLVEVLMLSLLHHPNLVN
LIGYCADGDQRLLVYEFMPLGSLEDHLHDLASDKEGLDWNTRMKIAAGAAKGLEYLHDIAKPPVIYRDFKSANIL
LDKEFQPKLSDFGLAKLGPVGDKSHVSTRVMGTHGYCAPEYAMTGQLTVKSDVYSFGVVFLELITGRKAIDSTKP
HGEQNLIAWARPMFSNRRRLARLADPRLHGQFPVRGLYQALAVASMCIQEQAVTRPLIADVVTALSYLSSHTYE
SNAAQNSTVEGIGRDFDALGGRVRRNERNGRRSSRNETSSIKKHLNQKDLDREKAVAEAKLWGENLRRANAQI
DSNHSDPV

>A113|LOC101756364

MGFLACLCRCPQNDEDDEEKEGEQFRINHQVTSESCPLNTGGILHMEGSQPIGRHDEAIIFPLHQLADATKNFS
QDCLLGRGGFGCVYKATLSDGQVVAVKQLDLNGLQGNREFLVEVLMLNLLHHPNLVNLFGYCVDGDQRLLVYE
YMPLGSLEDHLHDLAPDQEPLDWKTRMKIAASAAAGLEYLHDEAHPPVIYRDIKPSNILLGEGYHAKLSDFGLAK
LGPVGDKTHVTTRVMGTHGYCAPEYFLTGQLTIKSDIYSFGVVFLELITGRRPNDSNRPPEEQDLVAWARPLFKD
QRKFSRMADPLLHGRFPRRGLYQALAIAAMCLQEKAKHRPPIREVATALSYLASQTYDRNNTTARRNRAGPSTS
RVLDDQMNQDTTLASQQEVQMSIHCQTNQVVPEVKETSWSGSHRPGRGRVAPNGIDRERALADANVWAEA
WRRQEKASKMW

>A114|A0A2J6JXZ2

MGCFPCFDSREEEKLNPQKVRADRHEVHPSAPTNISRLSSGVDRLKSRNNNVSLRRESSGPKDSPDAQIAAQTFT
FRELAAATNNFRPDCFLGEGGFGCVYRGHLQGSGQVVAVKQLDRNGVQGNREFLVEVLMLSLLHHPNLVNLIG
YCADGEQRLLVYEFMALGSLEDHLHDVPADREALDWNTRMKIAAGAAKGLEFLHDKANPPVIYRDFKSSNILLD
QGFQPKLSDFGLAKLGPTGDKSHVSTRVMGTYGYCAPEYAMTGQLTVKSDVYSFGVVFLELITGRKAIDSTQPH
GQQNLVTWARPLFNDRRKFTSLVDPRLEGRYPMRGLYQALAVASMCIQEQAAARPLIGDVVTALSYLANQHQ
GYDQPRPKPETNARILSTNTEEQQAGASGRSRWDLALEVGSEKEESPKESAKMLNRERAVAEAKMWVEKRRQ
TAQAAFDANN

>A115|A0A2Z7B8S2

MGCFPCFDSREEETLNPRERRDDHKEVYPSIPSNISKLSSGADRLKTRSNVGLRKEPSVLKDLPNEQIAAQIFTFRE
LAAATNNFRPECFLGEGGFGRVYKGRLPNGQVVAVKQLDRNGLQGNREFLVEVLMLSLLHHSNLVNLIGYCAE
GEQRLLVYEFMPLGSLEDHLHDLPPGKDPLDWNTRMKIAAGAAKGLEHLHDKANPPVIYRDFKSSNILLGEGYF
PKLSDFGLAKLGPTGDKSHVSTRVMGTYGYCAPEYAMTGQLTVKSDVYSFGVVFLELITGRKAIDSSLPQGEQNL
VAWARPLFNDRRKFAKLADPRLQGKFPIRGLYQALAVASMCTQEQAAGRPLIGDVVTALSYLANQTYDPSIAVG

SGNRIALEKDDSRSNGRVLGTEEGGGSGRKWNLDGFDRDDSPRENTRNLNRDLDRERAVAEAKMWGENLRK
NNAQKF

>A116|A0A5P1E7Y4

MGMGCFSCFDSKEEEQLNTDHNNEKVVYKREEQPMVPPRIDMLPSGLDRVRSSGNLSSRRESVSPRDGSGVNI
SAQTFTFRELAAATKNFRPECLIGEGGFGMVYKGRLESTGQVVAVKQLDRNGLQGNREFLVEVLMLSLLHHPNL
VNLIGYCADGDQRLLVYEFMPLGSLEDHLHDLPPDKEPLDWNTRMKIAAGAAKGLEYLHDKANPPVIYRDFKSS
NILLDEGFHPKLSDFGLAKLGPVGDKSHVSTRVMGTYGYCAPEYAMTGQLTVKSDVYSFGVVFLELITGRKAIDSS
KAHGEQNLISWARPMFNDRRKLSKLADSNLQGRYPTRGLYQALAVASMCIQEEAATRPLIADVVTALSYLATQS
YDSSTAPASGHPSTRAGRPPAEKAGNDDGQRLEAYGSERDDSPREGVPLNNKDFDREKAIAEAKMWGANWR
EKRRAND

>A117|A0A5P1F0Z8

MGCFPCLDSEEDGSELRITKNKDDDDGDDEKRMEKPMVPPGIQSFSLRTESPKTKKSPQSKKESMKQEDVLGIN
ISAQTFTLKELAAATKNFRPECFLGEGGFGRVYKGQLQSTGQVVAVKQLDTSGLQGTREFLVEVIMLSVLHHPNL
VNLIGYCYDGDQRLLVYEFMPLGALDDHLHDLPPEKEALDWNTRMKIAAGAAKGLEHLHDKADPPVIYRDFKSS
NILLDEGFHPKLSDFGLAKLGPTGDKSHVSTRVMGTYGYCAPEYAMTGQLTKKSDVYSFGVVLLELITGRKAYDS
TKSHGQQSLVAWARPMFNDRKKLAKLADPRLEGRYPMRGLYQALAIASMCIQEDSTARPLIGDVVTALSYLSTQ
AYDPSSATRHRSSNQEREERRKNESEEPESSERDESPKEAAVITNKDLDRERAIAEAKLWGENLREKRRAAESSN
G

>A118|Kaladp0040s0137.1

MGCFPCFDSKEEEQLKPEPVKYGRRDNHLSVPSNISRLASGADRKPRNNTGSGRDLISPRDGPEGQISAHVFTFR
ELAAATKNFRPESFLGEGGFGRVYKGQLETTGQVVAVKQLDRNGLQGNREFLVEVLMLSLLHHPNLVNLIGYCA
DGDQRLLVYEFMPLGSLEDHLFDLPPEKEPLDWNTRMKIAAGAAKGLEHLHDNAKPPVIYRDFKSSNILLDEGFT
PKLSDFGLAKLGPTGDKSHVSTRVMGTYGYCAPEYAMTGQLTVKSDVYSFGVVFLELITGRRAIDGARPHGEQN
LVTWARPIFNDRRKFGKLADPQLEGRYPVRGLYQALAVASMCIQEQAATRPLIAEVVTALSYLANQAYDPASTS
GQDRRFKEPQASKVSPRGDSRWDLDGSEKDDSPKETARMLNKDLERERAVAEAKMWGENWREKRRQHGQG
SFDANG

>A119|A0A251ULT6

MGCFSCFDSRVEEKKIPQKVGADQPQLHSPASSNISRLPSGVDRLKNKNNVSLRRESSGPKDGPYSQIPAHTFTF
RELAAATRNFSPDCFLGEGGFGSVYRGRLQGSGQIVAVKQLDRNGLQGNREFLVEVLMLSLLHHPNLVNLIGYC
ADGDQRLLVYEFMPLGSLEDHLLDLPPDREALDWNTRMKIAAGAAKGLEFLHDKANPPVIYRDFKSSNILLGEGF
QPKLSDFGLAKLGPTGDKSHVSTRVMGTYGYCAPEYAMTGQLTVKSDVYSFGVVFLELITGRKAIDSAAPQGQQ
NLVTWARPLFNDRRKFATLVDPRLEGRYPMRGLYQALAVASMCIQEQAAARPLIGDVVTALSYLANHTYDPSER
NGRVSKNEEGGGSGRSRWGDLEGSDKGDSPRETQPPKIVNRDLDRERAVAEAKMWVEKRRQSAQGSFDGNH
NG

>A120|A0A251RUB6

MGCFPCFESSQEDNNNFNHQKVGHEVHPSAPSNISRLSSGVDRMKTRNNVNNAASLRRESSGPPDAQIAAQT
FTFRELAAATNNFRPDCFLGEGGFGCVYRGRLQSSGQVVAVKQLDRNGLQGNREFLVEVLMLSLLHHPNLVNLI
GYCADGDQRLLVYEFMALGSLEDHLHDVPPDREPLDWNTRMKIAAGAAKGLEFLHDKANPPVIYRDFKSSNILL
DEGFQPKLSDFGLAKLGPTGDKSHVSTRVMGTYGYCAPEYAMTGQLTVKSDVYSFGVVFLELITGRKAIDSTQP
HGQQNLVTWARPLFNDRRKFTSLVDPRLEGRYPMRGLYQALAVASMCIQEQAAARPLIADVVTALSYLANQGY
DPTTAPAHNIISSSSKERPKTARSSKNDEGGCSRWDLEGSDSPKETVKMLERERAVAEAKMWVEKRRQSG

>A121|PNY12509.1

MISNFITVLTLNLFSDLPPDKEPLDWNTRMKIAAGAAKGLEYLHDKANPPVIYRDLKSSNILLDEGFHPKLSDFGLA
KLGPVGDKTHVSTRVMGTYGYCAPEYAMTGQLTLKSDVYSFGVVFLELITGRKAIDNTRGHGEHNLVAWARPL
FKDRRKFPKMADPLLQGRYPMRGLYQALAVAAMCLQEQAATRPLIGDVVTALTYLASQTFDPNAGNQSSRGG
SSTPRMRDDRRSSMADSVDSPDRARLGSPSTHRNSPDFRKREGRDPSELGRIDTGGGGSGRKWGLDDLERRDS
QRDSPVNTGKGRETPRNRDLDRERAVAEARVWGENWREKKRANAMGSFDGQRTVERVNLATKVPDRYWLF
TPALKYSARPPVSIYVPQQNAASIDILNMSCLYSLIDGYCLVMGRSMLCTLFGLLEIALSVMVLRQVGD

>A122|LOC108846258

MGCFGRTGKSSKRSETKKKNNINDFAKHNKLEIDANCVRKSADRDSQTEPSSEGSPSGDDVNKEVTTDEDQFSL
DAKDSNVEDDVSGKKAETFAFEELSVSTGNFRSDCFLGEGGFGKVYKGLIERINQVVAIKQLDRNGAQGIREFVV
EVMTLSLADHPNLVKLIGFCAQGVQRLLVYEYMPLGSLEDHLHDLPYGKTPLSWKTRMKIAAGAARGLEYLHDT
MKPPVIYRDLKCSNILLGEDYHPKLSDFGLAKVGPSGDATHVSTRVMGTYGYCAPEYAMTGQLTFKSDIFSFGVV
LLELITGMKAIYKTKARKDQTLVGWARPLFKDRKNFKKMVDPLMEGDYPVRGLYQALAIAAMCVQDQPGMRP
VISDVVMALDHLASCNYDHNHKEKQSNFTETRVDVEDKRVIESNVCMEEGKQEIKICSAQAS

>A123|EPS66443.1

GCFPCFGSSGEKVSNSKHGDRESPKESSKEGSATQSNNRSSGVGSDKSRTPSSNESKKESTIPKDANISAQTFTYN
ELATATQNFKPECLLGEGGFGRVYKGRIQSTGQVVAVKKLDRNGLQGNREFLVEVLMLSLLHHPNLVNLIGYCA
DGDQRLLVYEFMPLGSLEDHLYDIAANKEPLDWNTRMKIAAGAAKGLEYLHDKANPPVIYRDLKSSNILLDDGYF
PKLSDFGLAKLGPVGDKTHVSTRVMGTYGYCAPEYAMTGQLTLKSDVYSFGVVLLEIITGRRAIDSTRGPGEEQN
LVAWARPLFKDRRKFPKMADPLLQGQYPMRGLYQALAVAAMCLQEHAATRPLIGDVVTALNFLASQTYDPAT
AAAATSSAQSSRVSTPRHHHRGRNPSDGGFFNVPDHGSPSSTHKNSPETGSGSGRK

>A124|LOC9315234

MGCFGRTTKSNKRSDIKTTKNNDFTPKKLTINSNIVHKNVNLTQPSSDCLKVSLCGDVSKEVVTKEDQLALDAKD
TNVEDEVIGKKAQTFTFEELAVSTGNFKSDCFLGEGGFGKVYKGFIEKINQVVAIKQLDRNGAQGIREFVVEVLTL
SLADHPNLVKLIGFCAEGVQRLLVYEYMPLGSLENHLHDLPHGRKPLVWSTRMKIAAGAARGLEYLHDTMKPPV
IYRDLKCSNILLDEGYHAKLSDFGLAKVGPRGSETHVSTRVMGTYGYCAPDYALTGQLTFKSDVYSFGVVLLELITG
RKAFDNTRTRNHQSLVEWARPLFKDRKNFKKMVDPLLEGDYPVRALYQALAIAAMCVQEQPSMRPVIADVVM
ALDHLASSKYDHSHRQKQPNVTETKVEEEKTLVESNVCVEEKQEEIKICSDQAT

>A125|PAN15826.1

MLSWLRRFPHDVVHRKENAGSGGRRTSSTTTWRNRSSSFTARIIRCASSVVDATAKHDDDDDCPLPSSPPPAPP
PPPEDDRVTVVSAQAFSFRELAEAAGNFRQDKLIGEGGFGRVYKGRLPAGEQEQQQRVVAVKQLDRNGRQGN
SEFVVEVLMLSMLHHPNLVRLVGYCAEGEQRLLVYEYMALGSLEDHLLLPMRRDDALLPWRTRMRVAHGAAR
GLEYLHDRAVIYRDLKSSNILLADDYSPRLSDFGLARLLPLPSSSSSSSSSSSSSSSSGTGRSRVMGTYGYCAPEYLRT

GKLSAKSDVYSFGVLLLELITGRRAIDASRPDGEQSLVGWAATMFGDPRRFQELVDPRLVMAMQGPPASELKQAVGVAAMCLQENHALRPVMTDVVISLSFLAASTTHNHHHHHPPPLRPSPDPSYAS

>A126|LOC8067569 GDK

MLSWLRRFPNDVIHRKQGNGTTGRRTSSSTSWRNKSNSFTARIIRCASSVVDTAGRRHDDDDDEDDDCRLPSSPPPPAPPPNDHYNNNDNNRNTSVILAAKAFSFRELADAAGNFRQANFLGEGGFGRVYKGRLHHIIAGGEELPVAIKQLDRNGFQGNNEFMVEVLMLSMLHHPNLVSLVGYCAEGDQRLLVYEYMALGSLEDHLLLLRGDADADDGSHQRQHGPLPWRTRMKIALGAAQGLEYLHENTVIYRDLKSSNILLDQDYSPKLSDFGLAKLLPAPRTDSSSSRSSSSSKVMGTYGYCAPEYLRTGKLSAKSDVYSFGVLLLELITGRRAIDASRPDGEQSLVGWAARIFGDPKRFHELVDPRLVMAMQSPTASELKQAVGVASMCLQEHYALRPVMTDVVMALSFLATNDSAPC

>A127|EMS68686.1

MGCCTSSQKKGAAAGDRRKEPAEKPSQIAPAASPSPTPSPSAEMSRLEASQGAVRTKFISSPGLHTNRLSFTYEELNAATLGFPRDHFLGEGGFGKVYKGVLDGNEVAIKILNPNGLQGNREFCTEVMVLSRMHHPNLVKLVGFCTDDDQRLLVYEYMPLGSLETHIFDLPPNKKPLDWNTRINILAGAAQGLKHLHVNCNPPIINRDVKCANILLGEEYHPKLADFGLAKLGPTGDDTHVSTRVMGTPGYCAPEYLESGQLTIKSDVYSFGVVILEVITGRKALDQSRIKAERSLAEWNYSLLPPNAIMAVPRTILCLPGISLATPLINRRDFAMLADPALGNQYSMTSLYQVLSVARMCLNTTASQRPQITDVAAALAHISKSRRTRRPAHQQSAAQVHQPGEDI

>A128|LOC109774779

MLSWLRCFPHDGTVMDEERKPRPGRSATFRKKHSPDAAPSRKRFIRSGTSLTASSSARASFGRHSVDVPNYNHSIVSARSFTFPELAAATDSFSHANLIGEGGFGRVYRGLIGSSAVAVKQLDRTGFQGDHEFLVEVLVLSSLLTHPNLVGLLGYCADGNQRLLVYQLMPLGSLENHLFLPRVPADGEEKPPPPVLPWRTRMRIAHDAAQGLEFLHETANPPVIYRDLKSSNILLDEGYNAKLSDFGLAKLATPITRNGKGGDAEEEKDGSSRVMGTYGYCAPEYVRKGHLTVKSDVYSFGVVLLELITGRRVIDESRPSGEQNLVAWAAPMFSEQRRMHELVDPLLVEGPSGRELKQAVAVAAMCLQEEDTVRPIMSDVVMALSFAADDLPSAPRYTSL

>A129|EPS68489.1

QRLGSSEKGMGCFPCFGSRDEEKLNPNGGRRREDDLIEALPTIPSSISKLSSGGDRPKSRTGVAPQKQSLGVKDSPPDAQIAAQTFTFRELANATDNFRPECFLGEGGFGRVYKGKLQTCQDVAVKQLDRNGLQGNREFLVEVLMLSLLHHPNLVNLLGYCADGDQRLLVYEFMPLGSLEDHLLDLPPEKQALDWNTRMKIAAGAAKGLEYLHDKAKPPVIYRDFKSSNILLGEGFFPKLSDFGLAKLGPTGDKSHVSTRVMGTYGYCAPEYAMTGQLTVKSDVYSFGVVFLELITGRKAIDSTQPQGEQNLVSWARPFFNDRRKFAKLADPRLNGKFPIRGLYQALAIASMCTQEQAAARPLIGDVVTALSYLANQSYDPN

>A130|A0A5E4EWW2

MLSLLHHPNLVNLIGYCADGDQRLLVYEFMPLGSLEDHLHDLPPDREPLDWNTRMKIASGAAKGLEYLHDKANPPVIYRDFKSSNILLEEGFHPKLSDFGLAKLGPTEDKSHVSTRVMGTYGYCAPEYAMTGQLTVKSDVYSFGVVFLELITGRKSIDSNRPHGEQNLITWARPLFNDRRKFSKLADPRLQGRYPMRGLYQALAVASMCIQEQAATRPLIGDVVTALSYLANQSYDPNMASGHGHRGSGEKDERRHRDERGGRILKNEEGGGSGRRWDLDGSEKDDSPKETARMLNRDLDRERAVAEAKMWGENWREKRRQSAQGSFDGTNL

>A131|A0A2I0L8D1

MLSLLHHPNLVNLIGYCADGDQRLLVYEFMPLGSLEYHLFDLPPDKEPLDWNTRMKIAAGAAKGLEYLHDKANP
PVIYRDFKSSNILLDEGYFPKLSDFGLAKLGPVGDKSHVSTRVMGTYGYCAPEYAMTGQLTVKSDVYSFGVVFLEL
ITGRRAIDSSRPHGEQNLVTWARPLFNDRKKFSKLADPRLQGRYPMRGLYQALAVASMCIQEQAAARPLIGDVV
TALSYLANRLYDSTAPSGHRGGLGSNDGRGSGRILRNEELGGSGRRWDFEGSEKEDSPRETGKILNSNRDAERER
VVAEAKMWGENWREKRRQSAQGSLDRNITNI

>A132|A0A2I0JNM1

MLSLLHHPNLVNLIGYCADGDQRLLVYEFMPLGSLEDHLHDLPPDKEPLDWNTRMRIAAGAAKGLEYLHDKAN
PPVIYRDFKSSNILLEEGFNPKLSDFGLAKLGPVGDKSHVSTRVMGTYGYCAPEYAMTGQLTVKSDVYSFGVVFL
ELITGRKAIDSTLPHGEQNLVTWARPYFHDRRKFVKLADPRLQGRFPMRGLYQALAVASMCIQEQGAARPLIGD
VVTALSYLSNQAYDNSSRGSGNKEGKANGEERGGGRILRNEEGEGSGRRWDLEGGSEKEDSPRDTARILNKDLE
RERAVAEAKMWGENWREKRKQDAQGSFDGSNG

>A133|ChHPGBG73041.1

MRGRLGYDGPPIHTGAQPFTFKELMIATRNFRSDCLIGEGGFGRVYKARLETGLVVAVKQLDRNGLQGNREFRV
EVAMLSRLCHPNLVNLIGYCADGDQRLLVYEYMPLGSLEDHLFDLPPEREALDWIMRMKIAAGAAIGLEYLHDK
ANPPVIFRDFKSSNILLDEKYHPKLSDFGLARMGPEGGETHVSTRVMGTYGYCAPEYAMTGQLTLKSDVYSFGV
VLLELITGRKVIDATRPHGEHNLVAWVS

>A134|ADX68910.1

MAEMETLGKIKHRNLVPLLGYCKVGEERLLVYEYMEFGSLEDMLHGEKGGFKTRLTWETRKQIAKGAARGLAFL
HHNCIPHIIHRDMKSSNVLLDRNMEARVSDFGMARLISALDTHLSVSTLAGTPGYVPPEYYQSFRCTAKGDVYSF
GVLLLEILTGKRPTDKEEFGDSNLVGWVKLHLNQGKAMEVIDPDLKDMGADYEVMRYLQITMQCLEDFPSRRP
SMLQVVSSQR

>A135|PpiHPATP67107.1

EDFHPKLSDFGLAKLGPVGDKTHVSTRVMGTYGYCAPEYAMTGQLTLKSDVYSFGVVLLEIITGRKAIDNNRAAG
EHNLVTWARPLFKDRRKFSQMADPLLQGRYPMRGLYQALAVAAMCLQEE

>A136|AcvPHY3

MATPGGPKTKHSVSVAQTRADARLHAAFEGSGDAGAGGSRAPFDYSKSGMDASSVTSVAPEAITAYLQRMQR
GGLTQAFGCMLVVAGQKIVAFSENAPEMLEVAAGLGTDLRMLFTQGSTAALDQAVKEEDLSSVNPLVLQSCGG
SAKQFYAMLHRIEDVAGVVIDLEPIENGVVEKKSSAEMAVKPIARVQSLPGGEIGRLCQVVVEEVQEMTGYDRV
MAYKFHEDGHGEVVAEVRRPDLEPYLGLHYPSTDVPQASRMMFMKNGVRMIGDCTLPPVRVVQAKELAQPIS
LAGSTLRAPHGCHAQYMSNMGSAASLTMAVAIDDYDDDSSLSCGSRKLWGLVVCHHPSPRTVSYPLRCACEKL
MVAFGVQLNIELDLAAQLRENHILTTQALLCDMLRRIRGAPIGIVSRSPSIMDLVKCDGAALYYGGRFWPLGTTP
SEFQIRDLAEWLLGASEEIASTGVTCTDSLAEMGYPGAALLGDAVCGMAAAMITPNSDFLFWFRSHTAKEVYW
GGAEHDPQSRDDDSWMQLPRSSFKAFLEIVKRRSLPWEEVEVDAIRSLQLILREDLEQFCAAVGAVKASDGDDE
DSLVPSAKKLSLKETEENGGADNSKKLERMHSAAAGGGGGGGRWEKMRLPSSLAQEWMEAIRGTGDGGASG
GGGGGPFDWDLISAFQHNSFIVVDALKPDFPIIYASTGFFNLTGYTSREVIGGNCRFLQGPDTNPADVASIREALA
QGTGTFCGRLLNYRKDGSSFWNLLTIAPIKDDLGSIVKLIGVQLEVSKYTEGIRANNRRPNGMPQSLIRYDVRHQ
DKVSAFIAQLVAALTKPDKVETPRLSSAMRFSLTGQTIESLPQPTAIPREGGGRTRRPRSSSFLSLLGMEKEKDIPEE
DELQELEVIMLEDASVGRPGSLDDPERTRRGIDLATTLERIGKSFVITDPRLPDNPIIFASDRFLELTEYTREEVLGN
NCRFLQGRGTDRKAVQLIRDAVKEQRDVTVQVLNYTKGGRAFWNLFHLQVMRDENGDVQYFIGVQQEMVA

PRPVHQPPELPDILPDRVEQEKAEVVRATAQRVDAAARELPDANLVPDHLFAPHSKVVTPLPHSKTNSSSWFAIR
RVQRRLRRGERLGLKHFRPIKPLGSGDTGSVHLVELRGTGQVFALKAMDKSMMLQRNKVHRARAEREILAIMD
HPFLPTLYASFQTKTHVCLITDYCPGGDLFLLQDKQPTQTLSERTASFYAAEVVVALEYLHCMGVIYRDLKPENVLL
QKNGHILLTDFDLSFLTSCRPQLILQGGKGRSRRSKRRRRVTFCAEPRVSSNSFVGTEEYIAPEIISGEPHSSAVDW
WALGILLYEMLYGRTPFVGRNRQKTFYNVLNKELIFPTSIPVSLAGRQLIAGLLQRDPTIRLGTLRGASELKKHPFFR
EINWPLIRWRKFSANQAHNANNVSSLDEGESDSGNAWEANGGSTQSFQDTF

>A137|LauNeo ACY92450.1

MSAKTKHSVRVAQTSADARLHAAFEGSDSGRGAGDGRSDVSQSGQPSASGHTEVAREAITAYLHRMQRGGLT
QSFGCMLVVAGNSILAFSENATEMLEMGVGLSTDLRILFTSASTAALDQAAKAEDLSSVNPLVVQSCGGSGKQF
YAVLHRIEDVEGLVVDLEPIESRPGFPVEKKSNAAVRPIARIQSLPRGNIGRLCQGVVEEVQEMTGYDRVMAYKF
HEDEHGEVVAEVRHPELEPYLGLHYPSTDVPQASRMMFMKNGVRMIGDCTLPPVLVVQTKELAQPISLAGSTL
RAPHGCHAQYMRNMGSAASLTMAVVIDDYDDSSLSGTRKLWGLVVCHHTSPRKVSYPLRCACQKLMEVFGV
QLNMELEFAAQLREQHILTTQTLLCDMLRRIRGAPIGIVSRSPSIMDLVKCDGAALYYRGRLWPLGTTPSEFQVQ
DLAEWLLGSSEEIASNGLICTDSLAEAGYPGAAVLGDAVCGMAAARITPYSDFLFWFRSHTAKEVFWGGAEHDP
QARDDDSRLLPRSSFKAFLEIVKRRSLPWEEVEVDAICSLQLILREDLEEFCAAAAPGEEYVDSLVPLSKKLSLKESRE
MGSVENSKKLERATSAGDGDGGGGTGGRRGWEKMSLPANLAQEWMEAIRGTAGDGGASAGGGGVPIPFD
WDSISVFQQTSFVVVDALKPDLPIIFASTGFYNLTGYTYREVVGANCRFLQGPDTNPADIASIREAVVPQGTGTFC
GRLLNYRKDGSSFWNLLTIAPIKNDSDTTVMLVGVQLEVSKYTEGFRANKLRPNGLPQSLIKYDVRHQDKVSALV
AQLVAALTKPHSVQEPPRPSFTMPVSLTGQTIEPLSPKVPAARPYSSSNVPQTASIAREGRGRRRHRSSTFLSLLG
MEEKDDLEEDPFPEPELIMVDDALVGRLRSLDDPDRTRRGIDLATTLERIGHSFVITDPRLPDNPIIFASDQFLELTE
YSREEVLGENCRFLQGRDTDRKAVQLIRDAVKEGRDMTVQLLNYKRSGKPFWNLFHLQAMKDKKGNLQYFIGV
QEETDMPDRVEQETAKVMRATAQNVDVAARELPDANLTPDHLWERHSKGVTPLPHSKINSPCWYAIRRVQRR
LRRGERLGLKHFRPIKPLGSGDTGSVHMVELRGTGRVFALKAMDKSMMLHRNKVHRARVEREILAMMDHPFL
PTLYASFQTKTHICLIMDFCPRGDLFLLQDKQPNKTLSEEAARFYAAEVVVALEYLHCMGVIYRDLKPENLLLQKN
GHILLTDFDLSFHTSCRPQLITSGRNSRRRIKKRQVQFIFCAEPHVSSNSFVGTEEYIAPEIIAGHSHSSAVDWWAL
GILLYEMLYGHTPFCGNNRHKTFMNVLGEELTFPSSIPVSIAGRQLIAGLLQRDPARRLGTFRGASDVKKHPFFEG
IDWPLI

>A138|AML79240.1

MEPSNPGNTNNSFPSGNVVRKVLDDSPAIISPLPRDSRGSLEVFNPVGYSAPASASQTTSNFSRWAPHNVLRNS
DASPNKSRADKDNGKHGNKDGLGSQSGRNTESVHTWMGFSEIDHLKGSIDDLGKGTSMVPRDIPSELGKQPQ
VSQERQLDSHKEPGIKQAVSGTWPLQRETGDTEEKLDKYVVPFLNEKSKKKKDCGSKFSESGMAERVAEWGLIL
KTDNKTGKPQGVGVRTSGESTKSGTSRRTSGNSMRTSEESDCGNEMGVTRISKELKDALSAFQQTFVVSDATKP
DYPILYASAGFFKMTGYAAKEVIGRNCRFLQGAGTDPEDVSKIREALAEARSYCGRLLNYKKDGTPFWNLLTIAPI
KDETGKVLKFIGMLVEVSKHTEGEKEKMLRPNGLPESLIRYDARQKEVAVSSVTELVLAVKNPHSFPESRNRPAM
RKSESAVDQMHAERTENMNNLNLKSSAPERRKSSSRLSGIFEVGKKRRKSSARSFMSFSGKSADTENQEPGQAP
ESDSESSDEERPDSLDNLERKREIRRGLDLATTLERIEKNFVITDPRLPDNPIIFASDSFLELTEYTREEILGKNCRFLQ
GPETDRATVKEIREAIDNQREVTVQLINYTKNGKKFWNIFHLQPMRDQKGEVQYFIGVQLDGSQHVKPLMNKI
PEKTEQESAILVKKTAANVDEAVRDLPDANSKPEDLWINHSKEVFPKPHMKSNSSWKAIQKILEAGEQISLKHFK
PIKSLGYGDTGSVHLVELHNGGDLFAMKTMDKTMVLNRNKVHRVCTEREILDMVDHPFIPTLYASFQTKTHICLI
TDYCPGGELFMLIDRQPTKVLREDAVRFYAAEVVVALEYLHCQGIIYRDLKPENVLIQRDGHVMLTDFDLSCLTSC
KPQVMMPICVSEKKKTKRNQPMFMAEPIRASNSFVGTEEYIAPEIINGAGHSSAVDWWALGILLYEMLYGYTPF

RGKTRQKTFANILHKDIKFPKKSHVSMAAIQLIHGLLHRDTTTRLGSYAGANEIKQHPFFRGVNWALVRNMKPP
ELDVPVQTTGENVDNTKVVSSDWSEREALLAMQSDVF

>A139|66339

MSTDIDLAIALSLSEGRTSNFVDTRRDSSIARSIQDEDIAQWQSTLGPGPLPEMHADDRQLRLERTLSERLAESGA
LTQHIPRTNSIEEGPGIADVGEFGAERLRMQERLGRYSLCEREVKGDGNCQFRALSDQLYRAPGYYDQLRRVAV
DELRSHADRYSPYVAEDWGDYLRQMAKSGTWGDHLTLQAIADHFGVKMYIITSYREGEIIQINPIGRLRSERVLY
LSFWAEVHYNSVFPRAEPPPVLPKDKTLGSRKLSQQVLPVGCKYFTYRELEEATGDWAQSNVIGDGGFGRVYKG
RLRNGLLIAAKRLDRHGLQGDKEFNVEVSILSRLHHPGIIKLLGVCVDGDQRIAVFELLSRGSLSSALGNSNDSESA
RSAGGRDPSKQLSHVLTWQQRMQIALGLAQGLSYMHQERLVHRDVTSGNVLLTEGAHARIADLGLAQRLTMP
DSIVIAAPVEADSILMGTYGYVAPEYAMSGELSQKTDVYAFGVVLLEILTAKPAVDGSRPPGCQMLSEWLLPSLSS
VDRIWEHLDPALDIGTVAAPQLATLADVALASLSKNPSDRPRMTDVVRVLETAAAFKPDPSAASPSAANRPALN
LIDAAAAPVGQLSAFGDPFASNPFAVGAPPESNLHAPLCTGMPGATDPGRGHAGGSSAAAAVTPQRGSPVSP
VRHLGHLSSSRAAHALPKAQPEDFASPLQSGLRFGVMSQVSPDVGSAFGRSSATDQAAGSGRSNDREVSSVAS
SPLFDAMASRQSSPAASSGAPQQRDEASQSPSQLGSNTISLLNYPADQFWPHRGGSTAAPEEPSSSDTGPGPP
VGSSFAAAVRRKEWRVAGPPSAPSFGDEPAGFSGGSGTQNRQRQRAGGGGLGGPLADSVASLTMSADRPPG
RQTYSHAPGEIFGALQPIVDEEAAGPPEAAKKGVEGAGLSPSVARRRSSGNPFKDEECSTPPNRSSAERSGSGNP
FAVAGGGDVQGRPGPSDGAQLHLDNSREAEVHWPCAQLDPAFLQLSPKPLAA

>A140|AHZ63854.1

MDSLKNELSDENFSSGSTSKKASSSSYPLVIPLPRDSRGSLEVFNPTEYSSLSTEHLSKPRAIAHTDQSQGTEGWEF
NKQREESFKKTIGWLSEMKKGNSTRSEILHVDSESGSLEKDENKEMEERAAAWGLILKTDNYTGKPQGVSVRTS
AEGGRAVTSQRTSANSMRNSEDSDHINQKEMPTVAENVKEALSSFQQTFVVADATKPDYPILYASAGFYKMTG
YTVKEVIGQNCRFLQGAETDPSEITKIREALSEGKGYCGRILNYKKDGTPFWNLLTISPIKDDTGNILKFIGMQVEV
SKFTEGRKDQTVRPNGLPESLIRYDARQKDMAASSVSELVLAMKELHSDFKHSELPAYQKSECTIEHLSTDPSEKKI
SKTKPFRKLCKSHKTMRKTLSNFKEDCKNTMKRRKSALRILLSCRGKTMHSGQDEPKQKIEFNDGGNFDDGRPE
SLDTLERQREIRHGIDLATTLERIEKNFVITDPRLPDNPIIFASDSFLELTEYSREEVLGRNCRFLQGPETDRETVKQIR
EAIDNEHEITVQLLNYTKNGKKFWNLFHLQPMRDQKGEVQYFIGVQLDGTQYLEPSLKRIPDSKAEINTKLVKGT
AASVDEAVRELPDANMKPDDLWKNHSKVVFPKPHGRNSSSWKAIEKILDRDERIGLKHFKPIKVLGHGDTGSVY
LVELDDTGERFAMKTMNKTVLLNRNKVHRACAERDILDILDHPFLPTLYASFQTKMHICLITDYCSQGELFLLLDK
QPEKVINEEAARFYAAEVIIALEYLHCQGIIYRDLKPENLLIQKDGHLQLTDFDLSCLTCCKPQIVAPECCNEKKNLK
QRDRSVMFIAEPIRASNSFVGTEEYIAPEIIKGTGHSSAVDWWALGILLYEMLYGFTPFRGKTRQKTFSNILHKELK
FPLNHYVSLSALQLIHELLQRDPSKRLGSARGANEIKLHPFFKDINWALIRSQRPPQLAADFSTLGEDDENKGILPE
WNHGELSLPFSSSMF

>A141|GAQ85442.1

MANCFACFARSDKEEAPEDDRSAPGRQHGAPARKVLPEHEMLVPSNGPIIGTPAHPNARLGRLPSKEPEEHFAS
HPEKSPAQIFTFRELQAATRDFRPQNLLGEGGFGRVYKGRLERTGQIVAVKQLDRTGLQGNAEFRVEVSMLSLL
HHENLVNLIGYCADGDQRLLVYEFMPLGCLEDHLHDVPPEREPLDWYTRMRVAYGAAKGLEYLHDKADPPVIY
RDFKSSNILLDVGFHPKLSDFGLAKLGPTGDKTHVSTRVMGTYGYCAPEYAMTGQLTTKSDVYSFGVVLLELITG
RRAIDSSRPRQSNLVAWARPLFKDRRTFPSMADPLLQGRYPMRALYQAMAVAAMCLQETATLRPQIGDVVM
ALNHLANQRQEPGEPGRRTSREIPRAGPRGMTTPERRGGEERNGDRLRGGYENVRQAGGRRREDENCGGRN
WEREKGIYFRGGQEGRQETPRRRDDEFGNGGERSESSHSRKPSGDGSESYRSEDTFEAERGGVKAAEQDPLGD
GDSARASPQVERFVPAPLQQEYAYPPSTREHPAFAGTAKSAILERMSSWEARRGRGGPAGTLRHRVLDRTQDR

EAAARQARVVEIPERGAGGEGSIAAESPREMPLGWDLQSEATTLSAEDRGQAVFDLEVPEASLQETFGVAAPG
RIEMGYGFGQDEPGRGPGAAAGQQDAFGSVVYPEVPDRGYHDFGRLSIDERASALEPPLVGAGAATELPLSNG
GANAFPSETAAEPFEPAVAQQKVADLGGGEKEPTDQAGGSSEAPAEKPVGFLAIPSFKARQEQALDAAGRVAFL
WQQQQQQSPPPVRPTPANPFDLSEESPQPSRARSPVTVESPRIVHMVESPRIVHMVESPRIVHTVESPRIVQSV
RSRPRTPERLMRLDMPRDSLERVDFVAEPGASGEPSRLEEYAWAQPQDPSNGRRPGSFGVGGMG

>A142|NaRLKBAF79980.1

SSSSSSSSSAAAAAAAAPSSLSSRPFVWTPPADSRPYCRPCPTNQVLRPFEGCDCVWPMMVILIIQSNSTSFPMH
QDEFRREIANGIKLDVEQVMIRNVTDVAPGDDSSLEVTLWLLPRTGERLSQDQATTIESALRGRSIKLNSSLFGSY
AIESILCPGEIESAAPERPSPFLPLSATSVKSRNEGEKPMPIWFVPVIVLSSVAAIAILLGSAWVLLICFDKREKKKRA
ATVATKAARREALHAKTSIKQSRAGPFALAQIPTAGGVGSFLGSARAYTLAEMKAATNDFKAANILGVGGFGKV
YKGVLENGTPVAVKVLIRNDCQGGREFVAEVTMLSRVHHRNLVKLLGVCHEDGVRMLIYELVPNGSVESHLHSA
HKAIKPLGWDKRMKIALGSAHALAYLHEDSNPSVIHRDFKASNILLEDDYTPKVSDFGLAKSAVEGQRFSISSRVM
GTFGYVAPECSMTGRIELKSDVYSYGVVLLELLSGRKPVDLTQPEGQQNLVTWARPLLEDTGEDGCGIERLIDPSL
RDGPMIEDIGHVAFIARMCVEPEASNRPSMGEVVQALKLVYTEDEPFSESSVKPIRKQPTTLYSGLLVGYDASASP
SVERPRSHAPAASNNPHSKSIEMSSTDAEIGCDNEGPSVGDALINVNPPLTTGETACHVDATNRPPSHAVKIGKS
KSISWDESMIARGQGVDWQKRPAPSRPAPNSRSKLKFLSMPFPNTLKRRSDDDSSARKEVRASRLGRIGMLDR
AAGPGRAGKPQAPNAAMTVSGIPQSTQSGPIVAMGLGASYGYGYGYGYGYGHEDGGSEKNSTKLSSLDYDTS
DDFGSRVWKKRPIGAAAVANGFPLDESPQWHCQVIEH

>A143|mrna06753.1v1.0

MDRVAKLASQKAVVIFSKSSCCMSHAIKRLFYEQGVSPAIYELDEESRGKEMEWALMRLGCQPSVPAVFIGGKF
VGSANTNVHQGYLVETFGRFLRAREWNVVKAHKMLIDCLNWRVQNEIDNILAKPILPIELYRGVRESQLIGLSGY
SREGMPVFAIGVGLSTFDKASVHYYVQSHIQINEYRDHVILKYGKPITTCVKVLDMTGLKLSALSHIKLLTIISTVDD
LNYPEKTIFDMGGCFPCFGSSNKEGSSGGGGVKEVSKKELGKEGSATQSHHVTRVSSDRSKSRTGSDPKKEPPIP
KDGPTAHIAAQTFTFRELAAATKNFRPECLLGEGGFGRVYKGHLESTGQVVAVKQLDRNGLQGNREFLVEVLML
SLLHHPNLVNLIGYCADGDQRLLVYEFMPLGSLEDHLHDLPTEKEPLDWNTRMKIAAGAAKGLEYLHDKANPPV
IYRDLKSSNILLDEGFHPKLSDFGLAKLGPVGDKTHVSTRVMGTYGYCAPEYAMTGQLTLKSDVYSFGVVFLELIT
GRKAIDNTRGPGEHNLVAWARPLFKDRRKFPKMADPLLQGHYPMRGLYQALAVAAMCLQEQAATRPLIGDV
VTALTYLASQTYDPNAASGQSNRVGPSTPRHKDDRRNMGDGLDSPDEPGRGGRHGSHSYRNSPDFRRKDPNR
DLGAGVELGRIETGSGSGRKWGLDGLERQESQRDSPVSAGRARETPRNRDLDRERAVAEAKVWGENWREKKR
ANVMGGSFDGTNE

>A144|A0A443P8A0

MGCFSCFDSKVEEELHAKNVRDDRREGKPVVESHISRLSSGADRQRMRSVIGSRKELSDRKDVPDLSIAAQTFTF
RELAAATRNFRPECLLGEGGFGRVYKGRLESTGQVVAVKQLDRDGLQGNREFLVEVLMLSLLHHPNLVNLIGYC
ADGDQRLLVYEFLPLGSLEDHLHDLPPDKEPLDWSTRMKIAAGAAKGLEYLHDKANPPVIYRDFKSSNILLAEGF
HPKLSDFGLAKLGPVGDKSHVSTRVMGTYGYCAPEYAMTGQLTVKSDVYSFGVVFLELITGRKAIDSSRPHGEQ
NLITWARPMFNDRRKLPRLADPQLQGQYPMRGLYQALAIASMCIQEQAAARPLIGDVVTALSYLASQSYNTNA
APAHNIKSLERKMIKEAEVAELMDKRREKVRFIPTEEKAIMRGMKSTKPLKQLKLSVPAQETPITNFLMVVAIHKE
GTSQPADAKEVDHQFSLEDLETIKVIGKGSGGVVQLVRHKWLGTLFALKVIQMNIQETVRKQIVQEVKINQASQ
CPHVVVCYHSFYHNGVISLVLEYMDRGSLVDIIKQVKTILEPYLAVVCKQVLMGLVYLHHERHVIHRDIKPSNLLV
NHKGEVKITDFGVSAVLQSSMGQRDTFVGTYNYMSPERISGSSYDYSSDIWSLGMVILECAIGRFPYISSEQEEG

WLSFYELLEAIVDQPPPSAPPDQFSPEFCSFISACIQKDPQQRMSSLDLLSHPFIKKFEDKDLDLGILMNSLEPPVN
FSR

>A145|K3YQ98

MNSHIASQFRSAGSHISGAEAVDRKGVDLGANTPRSTREESEGENSHMLAEEEEDLVETDRDGGAAPGQGRG
RGGGRQRLDRDAEGRQRLVAGRGGVGVDREIRHGEHRRRRTPCCLLAPSPLRFPSKNPIKNAPFLPLPSFASLSP
HLPPSIPSPGRSDPIHIHVHLPAQSSESPLGAGVGGAAERGGGGRNVAPLIDAAATRWWFSWPAAMGCFSCFD
SPADEQLNPKLGGAGGYGGSSSAAAAYGAGSGGGRHGDRGYPDLQQAPMVAPRVEKLCTAAEKARVKSNAL
AREASAPKDANGNIISAQTFTFRELATATRNFRPECFLGEGGFGRVYRGRLESTGQVVAIKQLNRDGLQGNREFL
VEVLMLSLLHHQNLVSLIGYCADGDQRLLVYEYMPFGSLEDHLHDLPLDKEALDWNTRMKIAAGAAKGLEYLHD
KANPPVIYRDFKSSNILLDEGFHPKLSDFGLAKLGPVGDKSHVSTRVMGTYGYCAPEYAMTGQLTVKSDVYSFGV
VLLELITGRRAIDSTRPHGEQNLVSWARPLFNDRRKLPKMADPRLEGRYPMRGLYQALAVASMCIQSEAASRPLI
ADVVTALSYLASQSYDPNAALASRKPGGDPRSKPSENGRVVSRNDETSSSGHKSPGKDREDSPRDLPGILNKDFD
RERMVAEAKMWGDRERMVAEAKMWGDRERMVAEAKMWGDRERMVAEAKMWGENWRDKRRTENGQ
GSLDSPTGNI

>A146|AML78026.1

MIAPIKDDTGSIVNFVGVQLEVSKYTEGARANSLRPNGLPHSLIKYDTRHQDKVSAFVAQLVAALTKPEKAETPRP
SSTMRLPLTGQTIEPLPQPAAMPREGGGKIRKHRSSSFLSLVGIPEKEKDIPEEDELPEPEVIMVDDASVGRPESLD
DPERIRRGIDLATTLERIRKSFVITDPRLPDNPIVFASDRFLELTEYSREEVLGNNCRFLQGRGTDRKAVQLIRDAVK
EQRDVTVQLLNYTKSGRPFWNLFHLQAMRDEKGDLQYFIGVQQETVAPRPVHQPLGVPDVLPDRVEQEKAM
VVRATAQNVDAAARELPDANLVPDHLWAPHSKVVIPLPHSKMNNSSWYAIRRVQRRLRRFERLGLKHFRPIKPL
GSGDTGSVHLVELRGTGQVFALKAMDKSMMLQRNKVHRVIAEREILAMMDHPFLPTLYASFQTNTHVCLITDF
CPGGDLFLLQDRQPTKTLSERAARFYAAEVVVALEYLHCMGVIYRDLKPENVLLQKNGHILLTDFDLSFLTSCSAQ
LILHGGKGRSRRSKRRRRVTFCAEPHVSSNSFVGTEEYIAPEIITGEPHSSGVDWWALGILLYEMLYGRTPFVGRN
RQKTFFNVLNKELIFPSSIPVSLAGRQLIAGLLQRDPTTRLGALRGASDVKKHPFFRGINWPLIRWRKSPATLARNL
SNANSVDEGDSDSGNKDGRSGNPWKGRNSKLGTTHKEARYSGSTETFQDTF

>A147|Pavir.Aa02161.1

MVVRASWPPRNGGHGRVKWGRSEALGAEALGCLRRQGCRRGCRWCDHGGVDDAVAVDEDAGGPDVADA
GAVDDGDEGASAAGVGRPSSADGGQEGVSEEAVGLKNGDSRRKTASRVLHHHHCCCGDSPCCSLPSLPLQKP
NKKRTFPSPPLLRLAFPPTSLPRSPRREDPIRSHPIHLSRLNQSPPLGGGEDQGGGGRNVAPLIDAASRWWFSWP
AAMGCFSCFDSPADEQLNPKLGGASSAAVAYGAGSGGGRHGDRGYPDLQQAPMAAPRVEKLSAAAEKARVK
SNALAREASVPKDANGNVISAQTFTFRELATATRNFRPECFLGEGGFGRVYKGRLESTGQVVAIKQLNRDGLQG
NREFLVEVLMLSLLHHQNLVNLIGYCADGDQRLLVYEYMPFGSLEDHLHDLPLDKEALDWNTRMKIAAGAAKG
LEYLHDKANPPVIYRDFKSSNILLDEGFHPKLSDFGLAKLGPVGDKSHVSTRVMGTYGYCAPEYAMTGQLTVKSD
VYSFGVVLLELITGRRAIDSTRPHGEQNLVSWARPLFNDRRKLPKMADPRLEGRYPMRGLYQALAVASMCIQSE
AASRPLIADVVTALSYLASQSYDPNAALASRKPGGDQRSKPGENGRVVSRNDETSSSGHKSPGKDREDSPRDLP
GILNKDFDRERMVAEAKMWGDRERMVAEAKMWGDRERMVAEAKMWGENWRDKRRAENGQGSLDSPTR
SS

>A148|KAPU94870.1

MGTPKAAGAVLVFYAALVSVVSSVYGISDPSEVNALSTMFNDFNQNPRLTGWTQNNGDPCGAAWYGISCLPD
NFITSIKIPGLMLNGKLEGWVLQNLEHLQVVDVSNNNITGEIPQQFPNNITEITMNNNLFTGSLPQLDQLSLVQII
NLSNNELTGNINPQIFTALTNLITLDLSFNQLQGTLPDTVKNMVALQTLNLQNNQLSGQLPPTLSQLNNLQTFNI
ENNQFTGSLPAGFNPRTYKYGGNQLQAPSSSPGTPSTPQSTPATTKASGGGSSIKAWFTSTTHIIEVAAGGAALV
LVLFSAFLFFCVIRKRRHPAKALDDQEAHHSSRRMQFPQSPVKEPVTSKRIFEAAPVDKGTIDEPKTETMKTLKAP
PSFKGVMGVMNPKASTTSKAVNKSSIAVTAFSVADLQAATNSFSQENLIGEGSMGRVYRAEFANGQVLAVKKI
DSFNASQVENEDDFLSVVQGLARLQHANAVELVGYCVEHDQRLLVYEYIGRGNLHELLHYSGDNFKGLTWNVR
IKIALGSARALEYMHEVCAPPVVHRSFKSANILLDDELNPHVSDCGLAALSPSGSEREVSAQMLGSFGYSAPEYA
MSGTYTVKSDVYSFGVVMLELLTGRKPLDSSRPRSEQSLVRWATPQLHDIDALARMVDPALKGKYPAKSLSRLA
DIVALCVQPEPEFRPPMSEVVQSLVRLMQRATLSKRSSDSGGAGMESIEPSENSI

>A149|AML79064.1

MQVEVSKHTEGYKVKALRPNGLPESLIKYDARQKDRAVMEVTELVQAMKHPHPSVPSSAYPPSSVKSSIGEIAAS
VAMPLVSEQPQVMLGERRGSQTASRTDQMAEGVSRNHRSSGLRSLIGLGKSDHPEPTEFIEPEILMTKDEEGSE
SLDELDDNKRLQEIRRGIDLATTLERIEKNFVITDPRLPDNPIIFASDSFLELTEYTREEIIGRNCRFLQGQDTDPKTVE
KIRDAIREQREITVQLLNYTKTGKRFWNLFHLQPMRDQKGELQYFIGVQLDGSEQLEPTQKRLSEKTEKEGAKIVR
ATARNVGEAVQELPDANLTPDDLWANHSKSVSAKPHKMHSDLWKAIQKVCERGEKIGLKHFRPIKPLGFGDTG
SVHLVELRGSGELFAIKAMEKSAMLNRNKVHRACAEREILAVLDHPFLPTLYASFQTKTHVCLVTDFCPGGELFLV
LDRQPSKVFSEETAKFYLAEVVIALEYLHCQGIIYRDLKPENVLLQKNGHVVLTDFDLSXCKPQLIRRDKPPGRCQR
HRPFPPPFFVAEPVNPSNSFVGTEEYIAPEVISGAGHSSAVDWWAVGILLYEMIYGRTPFRGKNRQKTFANILHK
DLTFPSSIPASLTARQLINGLLHRDPVNRLGTTTGAHEIKEHAFFQGIDWPLIRHLPPPSLEAPLELLGKDPDLDTKE
ALEWDELEASTPFISDVF

>A150|A0A5P1FHP5

MSNSSSESATIKPEIKHDIRICCNIRGYCQFMDGIVPQMRSEHWRAYGSSNFKNYVSFGMIPSDKATITLLLDNV
DLGTGKFFFNDSQTGTKQAYTFDLNWVRKHTRFNDEGEKPEGNRDMDNRIWTTYFSNKQQSQYKSGRGYLEA
LLEKLVRSKKKSDVSDFVMLQILYQLTMIWTPTARMNAPRHMFKYVARVDAVDHFPWATIIYKELWDSFKAVK
KATKGLTYLSGCAPLLSGAPEVKRDGGGGDNWQGVTARTGKMVVDRREGQPMAPPRIHRLPSGLDRTRSRGN
LSGRRESVSPRDGSGVKISSQTFTFRELAAATKNFRPECFIGEGGFGRVYKGRLENTGQVVAIKQLDRNGLQGNR
EFLVEVLMLSLLHHPNLVNLIGYCADGDQRLLVYEFMPFGCLEDHLLDLPDDKEPLDWNTRMKIAAGAAKGLEY
LHDKANPPVIYRDFKSSNILLDEGFHPKLSDFGLAKLGPVGDKSHVSTRVMGTYGYCAPEYAMTGQLTVKSDVYS
FGVVLLELITGRRAIDSTKAHGEQNLISWARPMFNDRRKLAKLADPKLQGRYPMRGLYQALAVASMCIQEDATT
RPLIADVVTALSYLASQAYDPSMGPASIHRSRSGRSATEKGEDGGGSSGRKLEVDGLEKDESPREVTSVNKDFDR
ERAIAEAKMWGANWREKRRASENGSFDASSGNG

>A151|OMO72628.1

MEKFSAITPITIFFTLLPLAFVAIADQQRPYVPIENITLNCGASSDLNDPDGRFWAGDNKGSKFGPNIESSSISYKAD
TRGSVVDTVPYMTARVSSSEFTYRFNVSQGKKFVRLYFYPVSYGKFDQAKAYFSVKTENQVLDLIFTPTDPSASN

DTYHAFINGIEIVSMPPQLYNLSSPTVETGLRFIGQRSTALETVYRLNVGGRTIPPVKDSGMLFRLWSDDFSYLLTD
NSYFSANFSLPIKYTKIPSYTAPEEVYMTARSMGPNHTYNKLHNLTWGLPVDSGFTYMDIEQQNEQSTITISLHPL
VDKYFDSILNGIEVFKLSDAGGNLAAPNPQVSTISPHDPSNSDMRPPIRKSGNKRTRIVAIVGPISSFIAISLLVFFIY
RILSNKVGTRNTPQLPSDVCRHFSLREIKVATNNFDPNFIVGRGGFGDVYKGFINDGSTIVAIKRLNPSSKQGVHE
FKTEIEMLSQLRHQNLVSLIGYCEDGKEMLLVYDYMAHGTLRDHLYNTNNPPLLWNQRLKICIGAAYGLNYLHR
GPNHTIIHRDVKTANILLSEKWVAKVSDFGLSKMNDMSNTHISTVVKGSFGYLDPEYYRLQQLSEKSDVYSFGVV
LSEVLCARAPIDRTRDHMEISLVEWVKHCYKNEILNRIVDSHLQGR

>A152|LOC110877396

MTPKYTSESSSLSFDVPSCLKNKRLKGINVTFKYSVSGDEWAWFCKVSTTNGVVDLMYNPKVFGKPEFGEVGIW
LSYWPIGNTLDFGNTVHVSIDVMSGLEVHECGVSLVYSDKETMENNMGGVEILGGNLSGFELSTGAYYLCRHDF
LRLMEVGRLTPDWFRILVGDTIDYTEVRGWRQTGRPKQLNDPSFTELKTVRCIIYGPQVEDTYKIAEMSKSSLGD
DTVAFTSSLLKGETIDEAVEEINVKQYKSGDETSDRYSHARIMKPATGEGEIVSSSTSAYPCRRFSLAKIQSATNNFS
DELVIGWGGFGKVYKGQIFSEEAGHIVAIKRRDSVSNFGEPEFKAEIDTLCTFHHVHLVSLVGYCDDNGEKILVYK
FMPNGSLYRKLHDVHTPLSWVTRLKIAIGAARGLEYLHIGVRTQHGVIHRNVKSSNILLDENWVAVISGLGLCIAG
PTDQSIPYVEDTVKGTFGYLDPEYALTGKLTYKTDVYAFGVVLFELLSGRRAIDMHNGEDDSKLVVWARKCVKER
KLDQMVDSNIKGTISTNCLRQFAQIADRCVHPVPKERPTMSELVASLEALLELQEKPDTSAKSSSIMGFPWKIKKY
FVPAIKPNFEESGTSSQKSHDKNKKRDDE

>A153|AcRKAIA99455.1

MRATSFLLLLQLLLQLFPRILGNSEGDALHNLRLALNDPSNVLQSWDPTLVNPCTWFHVTCNPSNSVIRVDLGN
AQLSGSLVPDLGELSELQYLELYSNNITGEIPKELGKLSQLVSLDLYQNGFSGSIPDELGELSHLRFLRLNNNTLQGN
IPYSLTKVNALQVLDLSNNNLSGRVPTNGSFSLFTPISFSGNILLCGPVVGKPCDGEPPFPPPPPFQPPPSPATTGN
TGGRSPSSTGAIAGGVAAGAALIFAVPAIIFAWYKSRRPQEHFFDVPAEEDPEVHLGQLKRFSLRELQVATDNFN
NRNILGRGGFGKVYKGRLADGSLVAVKRLKEERSPGGEMQFQTEVEMISMAVHRNLLRLRGFCMTPTERLLVY
PYMPNGSVSSRLRDRSPGEQPLEWPQRKRIALGSARGLSYLHDHCDPKIIHRDVKAANILLDEEFEAVVGDFGLA
KLMDYKDTHVTTAVRGTIGHIAPEYLSTGKSSEKTDVFGFGIMLLELITGQRAFDLARLANDDDVMLLDWVKGLL
RERKIEMLVDPDLKSNYETHEVEQLIQVALLCTQSAPADRPKMSEVVRMLEGDGLAERWEEWQRVEVNRSQD
VELVPHRSDWIVDSVDNLHAVELSGPR

>A154|M4CQI0

MLKETRVTSSEVVFKRSERRKKISSLSLILVSLHSDSALYSIYVVQSIPNCLFKLPKKKISTCVELYHVGILRLIDSVGKA
PFVLIEFRYLRGLKLSEMSWCCLPCFGSSAKDADSKDSLKKEVSAKDGSVTQQSHRASLDKSKSRGGSEHKKELTA
PKEGPTANIAAQTFTFRELAAATKNFRPECLLGEGGFGRVYKGRLETTGQIVAVKQLDRNGLQGNREFLVEVLML
SLLHHTNLVNLIGYCADGDQRLLVYEYMALGSLEDHLHDLPPGKEPLDWNTRMTIAAGAAKGLEYLHDKANPP
VIYRDLKSSNILLGDGYHPKLSDFGLAKLGPVGDKTHVSTRVMGTYGYCAPEYAMTGQLTLKSDVYSFGVVFLELI
TGRKAIDNARAPGEHNLVAWARPLFKDRRKFPKMADPSLQGRYPMRGLYQALAVAAMCLQEQAATRPLIGDV
VTALTYLASQTFDPNAPNGQNSRSSGGGGPPFIRTRDERRSLGDGSSLDSPAETRSRLGSPGAHKNSPDYRRRD
MVREVNAGSEAGSEAGGGSGRKWGLSDVEGTESQRESPASVGRGTRGTPRNRDLDRERAVAEAKVWGENW
RERKRGANEPGSFDSFND

>A155|B8AXG7

MGCFPCFGSGGKGEAKKGGGGRKDGGSADRRVARVGSGPWWLTGHDLEGSGCVWEWGLHPWLPKSKLEG
GKAFLAVGTSSVAAEFVINGTSLGLGEEREVQQLSRLEYLVDHGSVPSGMVAPNTRSFSLDKSKSQGGLDSRKDA

FIPRDANGQPIAAHTFTFRELAAATKNFRQDCLLGEGGFGRVYKGHLENGQAVAVKQLDRNGLQGNREFLVEV
LMLSLLHHDNLVNLIGYCADGDQRLLVYEFMPLGSLEDHLHDIPPDKEPLDWNTRMKIAAGAAKGLEFLHDKAN
PPVIYRDFKSSNILLGEGYHPKLSDFGLAKLGPVGDKTHVSTRVMGTYGYCAPEYAMTGQLTVKSDVYSFGVVFL
ELITGRKAIDNTKPLGEQNLVAWARPLFKDRRKFPKMADPLLAGRFPMRGLYQALAVAAMCLQEQAATRPFIG
DVVTALSYLASQTYDPNAPVQHSRSNASTPRARNRVGANFDQRRLHSPNHQQSPDLRKEGTTTSKYEAEVSRT
NSGSGSGRRAGLDSMDVTGSQMGSPAHAGRKRESSRSTDRQRAVAEAKTWGENSRERKWPNARGSFDSTNE

>A156|LOC107416413

MNPDPHVSLKQNLTWRFPVEVGFLYLVRLHFCETQLEVTESNQRVFFIFLNEMIAEEEMDVIFLSGGSKIPLYRDY
LVSVQDQTHGRLANLTLQLHPNMKNKPKYANAILNGVEIFKLNKSDGSLAGPNPEAPVVNTNPKPWLPEKKNN
KGSWPIKAVITAGAVVTTFLLALSVIWFLVKLRRKQTVPLTISTVTSLPSDICRRFSLAEIRAATNNFDHQRIIGLGGF
GNVYKGYIDDQTTKVAIKRLKSMSKQGAQEFRTEIEMLSKLRHVNLVSLIGYCDDESEMILVYEYMARGTLRDHL
YQTENPSLPWKQRLQICIGSARGLHYLHTGAKHMIIHRDVKSTNLLLDEKWTAKVSDFGLCRTGPTDSLNSHVST
GVKGSFGYLDPEYCRRQQLTEKSDVYSFGVVLLEVLCSRPPLGRSLPKEQVGLANWALTCYQNGAVDQIVDPKL
MEQIAPVCLKKFVGIAVSCVDNEGIKRPSMEDVVGGLEFALQLQETAEKCINAEDDQVQASPYEERVSELFGDSS
KSKSDQGWSIGTSTDTSNVDGGSSMSAGNLMSGSESVFSVVETQEGRLMF

>A157|101314641

MNKINGIARTTLNGSELQFLLWSDGDRQWKQSYAEPKLQCDWYGHCGANSKCYLDNISLFECDCLPGFEPKSIN
EWNQNDGSDGCVSKRKEVSKCGNGDGFVQVTSVKFPDTSIAARLEARISNEVCRQECLANCSCKAYMSLENEE
GVVDCWTWYDDLMDIIVYTEAGRDLYVRVDQMELAESAGKSKGFFKRSMLAISIVCALLALALIIMCACWWCK
KRRNTTEIVEADELEETKRHPELQFFDLNTLMAATDNFSPVNELGQGGFGSVYKGQLPNEQKVAVKRLSKTSGQ
GTEEFKNEVALIARLQHRNLVKLLGCCIKGEERMLVLEYMPNKSLDSFLFDHTRRSFLDWEKRFEIINGIARGILYL
HQDSRLRIIHRDLKTSNVLLDAEMNPKISDFGMARIFDGDQVQDKTNRVVGTYGYMSPEYAVFGRFSTKSDVFS
FGIVLLEVVSGKRNNSFSQEDPSMNLIRHVWNLWREDRVLEIVDSILESYNSDEVMRCIQVALLCVQEESEDRPA
MSTIVFMLSGETSPASPNQPAYVFRKNSITDADPSVVPTPLYSMNDLTITAEAAR

>A158|LOC108818503

MVKQLCERTNTRGRDKNKLLCFHCPLYSLSLSLSKQQLFLFFLILQFESEQKSESQFPSFLSGVQLKKFLRFIVFFYSC
SFDSSIGSKGGGFCKFCDYYSFPKLSGEEEEEMGCFSCFDSSDDDEKLNPVDESKSQKTTQPTLSNNNISGLPSGG
EKLSSKSNGGSRRDLLLPRDGLSQISAHTFAFREVAAATMNFHPDTFLGEGGFGRVYKGRLDSTGQVVAVKQLD
RNGLQGNREFLVEVLMLSLLHHPNLVNLIGYCADGDQRLLVYEFMPLGSLEDHLHDLPPDKEALDWNMRMKIA
AGAAKGLEFLHDKANPPVIYRDFKSSNILLDEGFHPKLSDFGLAKLGPTGDKSHVSTRVMGTYGYCAPEYAMTG
QLTVKSDVYSFGVVFLELITGRKAIDTDMPHGEQNLVAWARPLFNDRRKFIKLADPRLKGRFPTRALYQALAVAS
MCIQEQAATRPLIADVVTALSYLANQAYDPNKDESKRSRDERGGRLITRNDEGGGSGSKFDLEGSEKEDSPRETT
RILNRDINRERAVAEAKMWGESLREKRRQSEQGTSESNSTG

>A159|A0A453GIA6

HVLLPLPVASIPLPLPQFHPPLPPPRLKIQSCLGAVPPRKRRSSPGGDPASSAPARRPLLSASSTRFPGSGEGAEERR
ERAWMSCFSCFGPALEAEGRKPVPDAKDPRAKDGAVSDRAGSDKLRLQGGSDPKNNHLTIPRDGSSQNIAAQI
FTFRELAAATKNFRQDCMLGEGGFGRVYKGRLESGQAVAVKQLDRNGLQGNREFLVEVLMLSLLHHTNLVNLI
GYCADGDQRLLVYEFMPLGSLEDHLHDVPPEKEPLDWNTRMKIAAGAAKGLEHLHDKASPPVIYRDFKSSNILL
GEGFHPKLSDFGLAKLGPVGDNTHVSTRVMGTYGYCAPEYAMTGQLTVKSDVYSFGVVFLELITGRKAIDNTKP
QGEQNLVAWARPLFKDRRKFPKMADPMLQGRFPMRGLYQALAVAAMCLQEQATTRPHIGDVVTALSYLASQ

TYDPNAPTQHTRSNSSTPRARNVGGRNSEQRNGRSPNHHSPRTSKHGGEVSRTSSTGGDSGRRSGLDEMDM
AGSQAGSPAQTGRKRETPRTADRQRAIADAKMWGENSRERKRPNDSFDSTNE

>A160|A0A5J9V222

MGCFSCFDSPADEQLNPKLGGGAGAGGGYGGSSSAAAAYGAGAGGRHGDRGYPDLQQAAMVAPRVEKLSA
GAFLGLAGWVDPGARSLVGGIGSGGAAEKARLKSNALAREASAPKDANGNVISAQTFTFRELATATRNFRPECF
LGEGGFGRVYKGRLESTGQVVAIKQLNRDGLQGNREFLVEVLMLSLLHHDNLVNLIGYCADGDQRLLVYEYMPF
GSLEDHLHGTLINDGEQHTMSFAFEFPCHLPADKEALDWNTRMKIAAGAAKGLEYLHDKASPPVIYRDFKSSNIL
LAEGFHPKLSDFGLAKLGPVGDKSHVSTRVMGTYGYCAPEYAMTGQLTVKSDVYSFGVVLLELITGRRAIDSTRP
HGEQNLVSWARPLFNDRRKLPKMADPRLEGRYPMRGLYQALAVASMCIQSEAASRPLIADVVTALSYLASQSY
DPNAARKPVGDQRNNKAGENGRVVSRNDETGSSGHKSPGKEREDSPRGLPGILNKDFDRERMVAEAKMWG
DRERMVAEAKMWGDRERMVAEAKMWGENWRDKRRAVENGQGSLDSPTETG

>A161|A0A0D9YJR9

MSCFLCFGSAQEGEAKKPGADSKDARKDGSADRGVSRVGSDVWVKAQDHFEVVLGWIFYVSADKSVNAQRIA
HYHYSAPQKEKRTDYQLYNIFFLDKSRSHGGLDSKKDVVIQRDGNNQNIAAQTFTFRELAAATKNFRQDCLLGE
GGFGRVYKGRLETGQAVAVKQLDRNGLQGNREFLVEVLMLSLLHHTNLVNLIGYCADGDQRLLVYEFMPLGSL
EDHLHDLPPDKEPLDWNTRMKIAAGAAKGLEYLHDKASPPVIYRDFKSSNILLGEGFHPKLSDFGLAKLGPVGDK
THVSTRVMGTYGYCAPEYAMTGQLTVKSDVYSFGVVFLELITGRKAIDNTKPQGEQNLVAWARPLFKDRRKFPK
MADPMLQGRFPMRGLYQALAVAAMCLQEQATTRPHIGDVVTALSYLASQTYDPNAPVQHSRSNSSTPRARNL
AGWNEDRRSVRSPNHHSPDLRREAARSSRAEVSRTSSTGDSGRRSGLDDLDMTGSQMGSPAQTGRKRETPRT
ADRQRAIAEAKTWGENSRERKHPNGHGSFDSTNE

>A162|Sphfalx0021s0054.1

MGCFPCFGSQKKQDKKPLKKEDSNRETTTTTTGQSPVGSAVHLARLPSEKLKSSSSFDSKKEGSRDGAATTHIAA
QTFTFRELAAATKNFRPESLLGEGGFGRVYKGRLENTGQVVAVKQLDRNGLQGNREFLVEVLMLSLLHHPNLVN
LIGYCADGDQRLLVYEFMPLGCLEDHLHDLPPDKDALDWNTRMKIAAGAARGLEYLHDKANPPVIYRDFKSSNI
LLDEGFHPKLSDFGLAKLGPVGDKTHVSTRVMGTYGYCAPEYAMTGQLTLKSDVYSFGVVLLELITGRKAIDNAR
AAGEHNLVAWARPLFKDRRKFPSMADPMLQGRYPMRGLYQALAVAAMCLQEQAATRPLIGDVVTALSYLAS
QQYDPSAHPVGSSRFAPSTPSREKREKKVTTGSGRGGAADERVRDRGSKGGSRSPALHPLASPDVRPKDGLPRG
RTVNSGFESGRGQDSASARKRDSDEWDRYDGSRDSPSNSERAPKEVPKPVAPTRERERAVAEARVWGENWRE
RKRGTNNQGTGDGYDAGFG

>A163|LOC109713813

MGCFCSIDQSSDTPSSSSPPPPPPPTPPQPPQWNSPPALLIPPQPHPPPLQSSPAEQMPRCPQSSPLLIPTVQSA
SPRLAPIYWGSAVSCRVLIEPRGGPPPSPHRVPIEQSTSPPPSSRCVPTEWRSSPLPSDRRVLIERRGRPLPSPLRVP
IERIASPPSSSRHVPTKRSTSLLSSPRRVPIARNTSPPPGGSSLFEPFFKGPSVAASSSSAPSAEMKKHSQNFTFHDL
AIATENFSNANFLGEGGFGNVYKGKLNSGEVVAVKVLRKMGVQGNKEFLTEVLMLTMLKHPNLIGLVGFCSEG
DQRLLVYEFMPNGTLLDHLFDLPPNREPLDWTTRIKIILGVANGLHYLHDLADPPVIYRDMKAENILLDDDFNPKL
SDFGLTKLAPAGDEAYVFTRILENQGYCDPEYVATGKLSKKSDVYSFGILMLELISGRIVYDETAPRLLAWASACLS
NRKLYVQLVDPLLLGCFRRRSLNILAPVIGFCTNYEPCLRPPIKDVVKAVANAASFSYDSESSTPSTTSSESSASL

>A164|LOC110790751

MIFLGCCRAEILDEEGRRSSNSNNSLVSTSRKSRGWRRRFSKGDTMDRDLTNNDGKMSIATVVKTLSLRRTTKG
KKKQHTVEQSERSIPGQKISAAQTFTFRQMANATNNFSIDNLVGEGGFGRVYKGFLQGVDKIVAVKQLDRNGL
QGNREFLSEVFMLSLVDHPNLVNLIGYCADGDQRILVYEYMAFGSLEDHLLDLPPNTEPLSWHTRMKIAEGAAK
GLEYLHETSKPPVIYRDFKASNILLDEEFHPKLSDFGLARIGPTGDKEHVSTRVMGTYGYCAPEYAMTGKLTTKSD
VYSFGVVFLELISGRRAVDTTKPTSEQNLISWAQPLFKDRKMFTKMADPLLQDRYPVKGLYQALAVAAMCLQEE
ASTRPLISDVVTALAFLSKPEDNNDNINIIDSPRHSVNTVIIHSVEEDDDDYDNEQRFYKCKNSEKEEEEEEEDDDE
QRYVYVGYLCSQKSTYTHVFPMATPPPEISPLILADDATVDAPPAPPSGTTAAIPPGPPAVVSPVGVSSVSSLPTS
GMSKDTSTA

>A165|A0A443NDJ9

MGGCFPCFESSNKEEEEEEDVEGRNGVKGGDEGNKKEFGKEGSAAVNRVNSDKSKSLNGTDSKKERKEASITKE
VPNVHIVAAQTFTFRELAAATSNFKQECLLGEGGFGRVYKGQLESTGQVCYNRYVVAVKQLDRNGLQGNREFL
VEVLMLSLLHHSNLVNLIGYCADGDQRLLVYEFMPLGSLEDHLHDIPPDKEPLDWITRMKIAAGAAKGLEYLHDK
ANPPVIYRDFKSSNILLGEGYHPKLSDFGLAKLGPVGDKTHVSTRVMGTYGYCAPEYAMTGQLTLKSDVYSFGVV
FLELITGRKAIDNTRVAGEHNLVAWARPLFKDRRNFAKMADPLLQGRCPTRGLYQALAVAAMCLQEQAATRPLI
GDVVTALTYLVSQPFDPNAAAAQNNRVGSSFLGHNDEQKSFNAMSDTQNAVRRGGRHGEESPQNSLDLRKR
DHVRGVSFGAEDGRGESGGGSGRKLGFDDLERLESQRDSPIHARKTKDSLKNINRDVDRERAVAEAKVWGDIW
RERKRANTRSSSDGTNE

>A166|101295698

MFPYSNGNYTTARLSPLSLTYYAHCLANGNYTVKLHFAEIVLRDNRSYYGVGRRMFDVYIQDKLVLKDFDIRKEA
MGVNKEVIKVFNAVVNVKTLLIRFQWAGRGTTNVPRRGIYGSLISAISVQSDFKPPDDSKRTIYIVVAVVSALCLLF
LIIGILWLRGCFGHKTTREEELRGLDLQTGYFKFKQIKAATNNFDPANKLGEGGFGAVYKGELLDGSFIAVKQLSSK
SKQGNHEFINEIGMMSALQHPNLVKLYGCCTEGNQLLLVYEYMENNSLSRTLFGSEEGLRKLNWPTRQKICVGI
ARGLAFLHDGTLKIVHRDIKTTNILLDRDLNAKIADFGLAKLDEEHTHISTRVAGTIGYMASEYALWGYLTDKADV
YSFGVVALELVSGKNNIKYRPNENFVCLLDWALVLQQKGNLMELVDPKLGSGFNKEEALRMIKVALLCANSSPAL
RPTMSQVVSMLEGRTDVHEVTINPSIYGDEMRFKAFTEDSDPDMQESFEETRSFIRSDSKWTASSSLNIDNSETR

>A167|LOC112283883

MGCFPCFDSKPKERTSLKKNAYNSSQRDGQSPADNPTPQISKLTSGNSKKFETNESFDKKEPVREESTPIDAHTFT
FRELAVATKNFRPECLLGEGGFGRVYKGRLENTGQEVAVKQLDRRGLQGNREFLVEVLMLSLLHHTNLVNLIGYC
ADGQQRLLVYEFMPLGCLEDHLHGLPQNKECLDWNTRMKIAVGAARGLEYLHDKAEPPVIYRDLKSSNILLGEG
FHPKLSDFGLAKLGPVGDKTHVSTRVMGTYGYCAPEYAMTGQLTLKSDVYSFGVVLLELITGRKAIDESRGPGEH
NLVAWARPMFKDKRKFQSMADPMLQGRYPIRGLNQALAVAAMCLQEQAATRPLIADVVTALSYLASQIYNPG
VHPLGSSRFAPATPSHEKPHKEKKAPPGPNALEERMLKEQRAGKVAPWSPSQRSQTASLDLRVKGSMGARARV
VNGMMLESQESQGSQGSQALEEWERRSSVGRRDSPKHHAAPAARTGRDGGMQPHAPTRERERAVAEARV
WGENWRDRQRGPT

>A168|A0A2U1NTM5

MGGCFPCFGSSNTNNEGNNNNNNGVKEVVKKDVVIGKEGSVGHQSHHVSRVSSGKSKSRVSLDPKKEQMVT
KDGQTANIAAQTFTFRELAAATKNFRPESLLGEGGFGRVYKGRLESTGQVVAVKQLDRNGLQGNREFLVEVLML
SLLHHPNLVNLIGYCADGDQRLLVYEFMPLGSLEDHLHDLAPDKEPLDWNTRMKIAAGAAKGLEYLHDKANPP
VIYRDLKSSNILLDEGFHPKLSDFGLAKLGPVGDKTHVSTRVMGTYGYCAPEYAMTGQLTLKSDVYSFGVVFLELI
TGRKAIDNTRAPGEHNLVAWARPLFKDRRKFPKMADPLLQGRYPVRGLYQALAVAAMCLQEQAATRPLIGDV

VTALTYLASQTYDPEAARAERAARGSGSGTPRSRTDRRNPSDGGLDGLDDPSRALGSPSTYKNSPDYRKRESLRE
INTRSDIGDGESSNGGNGSGRKWGLVDDPDSQRNSPANTSRARETARNRDLDRERAVAEAKVWGENWRDRK
RTNTMGGSFDATNE

>A169|KMZ56879.1

MRRFFICCGTSVGDGDDYEEEEEEKQKQKHKGLKKEKENFRRERRSGDGFKENGSTHVPSNHGARVIGVRSNSR
IGSESKKDNIPAPRDSNSEPHIAAQTFSFQQLAIATKFFKQESLLGEGGFGRVYKGNLDSTGQTVAVKQLNRNGL
QGNREFLVEVLMLSLLHHPNLVNLIGYCADGDQRLLVYEFMSMGCLEDHLHDVPLGQEPLNWNTRMKIAAGA
AKGLEYLHDKANPPVIYRDLKSSNILLDSAFNPKLSDFGLAKLGPVGDNTHVSTRVMGTYGYCAPEYAMTGQLTL
KSDVYSFGVVFLELITGRKAIDSTRRSSEENLVAWARPLFKDRRKFPKMADPLLHGRYPMRGLYQALAVAAMCL
QEQAATRPLIADVVTALSYLASQTYDQNATIGSNRVGSSTPRRSTGHRNAGGDSNHSPYWNSPDSVPSEPAGR
VVSFGAEVGRSEFSTPGSGSRLGLDDELDTQSSGLGSRIDSPVRTLKARDSPKNIDRERAIAEAKVWGENWRDRK
RPNQKTL

>A170|B8AT94

MICVGLCAGARGRGNATVKKELSALKDANGNVISAQTFTFRQLAAATRNFREECFIGEGGFGRVYKGRLDGTGQ
IVAIKQLNRDGTQGNKEFLVEVLMLSLLHHQNLVNLVGYCADGDQRLLVYEYMPLGSLEDHLHDLPPDKVPLD
WNTRMKIAAGAAKGLEYLHDKAQPPVIYRDFKSSNILLGEDFHPKLSDFGLAKLGPVGDKSHVSTRVMGTYGYC
APEYAMTGQLTVKSDVYSFGVVLLELITGRKAIDSTRPHVEQNLVSWARPLFNDRRKLPKMSDPGLEGRYPMRG
LYQALAVASMCIQSEAASRPLIADVVTALSYLASQKYDPNTTPSSKKAGGGEAGRALSRNDEAGSSGHKSPSSKD
SPREQQLPGILNDRERMVAEAKMWGENWREKRRAAATTSSNAQHIIGVARAKGVSGDDGEADGGECEAARR
ASVLGEDTAGGKQVEESLDDGGVQVARVGASDQSLVGTPRRMMAVSAMGTEIMFASSIITRRASMGRIYRLG
RLLRPFSKVSP

>A171|A0A443PX47

MGSCLPCFGSSNTDKEERNGVKGGETGSKNEFGNGKEGSSVSRFSSDKSKSRSGSDSKKEKKEISISKDGSTAHIA
AQTFTFRELAAATKNFRQESLLGEGGFGRVYKGRLESTGKVVAVKQLDRNGLQGNREFLVEVLMLSLLHHPNLV
NLIGYCADGDQRLLVYEFMPLGSLEDHLHDLPPDKEPLDWNTRMKIAAGAAKGLEYLHDKANPPVIYRDFKSSNI
LLGEGYHPKLSDFGLAKLGPVGDKTHVSTRVMGTYGYCAPEYAMTGQLTLKSDVYSFGVVFLELITGRKAIDNTR
AAGEHNLVAWARPLFKDRRKFHRMADPLLQGRYPTRGLYQALAVAAMCLQEQAATRPLIGDVVTALTYLASQT
YDPNAAAAQNNKVGPSTPRHKDERKSLGFVPNTQDGYDGRHGAQSPHQNSPDFRQRDLVRGVSFGAEAGRG
ESGSGSGRKWGLDELERSDSHLDSPIHVGKTREGPKNVNRDFGRERAVAEARVWGENWREKKRTNTYGGND
GMNE

>A172|XP_022842474.1

MGGCFPCFRSSKTEGNNSDNGVKQVVKKESFKEGSAAQAHGNRVNSDKSKSRSSNDSKKEATIPKEPTPNIAA
QTFTFRELATATKNFRSECLLGEGGFGRVYKGRLESTGQVVAVKQLDRNGLQGNREFLVEVLMLSLLHHQNLVN
LIGYCADGDQRLLVYEYMPLGSLEDHLHDLPQDKEPLDWNTRMKIAAGAAKGLEYLHDKANPPVIYRDLKSSNIL
LDEGYFPKLSDFGLAKLGPVGDKTHVSTRVMGTYGYCAPEYAMTGQLTLKSDVYSFGVVFLELITGRKAIDNNRP
PGEQNLVAWARPLFKDRRKFPKMADPLLQGCYPMRGLYQALAVAAMCLQEQAATRPLIGDVVTALTYLASQT
YDPSTPTSHSNRVGPSTLRHRDDRRNTSDRMDGLDEPGRGGQNGSPSTNKNSPDIRKRDSFPEFNAGAELRRIE
TGCGSGRKWGLDDTEHPDSRKDSPVNVGRARETTRNRDLDRERAVAEAKVWGENWRERKRGNAAGSFDAT
NDSLR

>A173|LOC105156909

MGGCFPCFGSSKKEGNSSSSNNGVKEVAKKESFKEGSAAHSNSHVNRVNSDKSRARNSSDSKKETGIPKEPTAHI
AAQTFTFRELAAATKNFRPECLLGEGGFGRVYKGRLESTGQVVAVKQLDRNGLQGNREFLVEVLMLSLLHHPNL
VNLIGYCADGDQRLLVYEYMPLGSLEDHLHDLPPDRKPLDWNTRMKIAAGAAKGLEYLHDKANPPVIYRDLKSS
NILLDEGYFPKLSDFGLAKLGPVGDKTHVSTRVMGTYGYCAPEYAMTGQLTLKSDVYSFGVVFLELITGRKAIDNT
RGAGEHNLVAWARPLFKDRRKFPKMADPLLQGQYPMRGLYQALAVAAMCLQEQAATRPLIGDVVTALTYLAS
QTYDPNSPSAQSNKIGPSTPRHRDERRNPSDGMEGLDDSGRGAHHGSPSTYKNSPDSRKRDSAFVSGADLRRIE
TGGASGRRWGLDESDRPASQKDSPVSAGRGGETPRNRDLDRERAVAEAKVWGENWRERKRGNTIGSFDGSN
E

>A174|LOC110819901

MGGCFPCFGSSDKEGSSSGGAVKELSKKDSSKDCSVAQSHHVSRVSSDKSKSRSGSDHKKEPVVPKDGTTAHIA
AQTFTFRELAAATKNFRPECLLGEGGFGRVYKGRLESTGQVVAVKQLDRNGLQGNREFLVEVLMLSLLHHPNLV
NLIGYCADGDQRLLVYEFMPLGSLEDHLHDLPQDKDPLDWNTRMKIAAGAAKGLEYLHDKANPPVIYRDLKSSN
ILLDEGYHPKLSDFGLAKLGPVGDKTHVSTRVMGTYGYCAPEYAMTGQLTLKSDVYSFGVVFLELITGRKAIDNTR
GPGEHNLVAWARPLFKDRRKFPKMADPLLQGRYPMRGLYQALAVAAMCLQEQAATRPLIGDVVTALTYLASQ
TYDPNASSAQNNRVGPSTPRSKDDRRNMADGLDSPDERGRHGSPSTHKNSPDYRRRDTVRELSTASDLVCSET
GGVSGRKWDLDDLERQEFQTDSPVNTGREGRXPRNRDLDRERAVAEAKVWGENWREKKRAHAVGSFDGTN
E

>A175|A0A068TRM7

MGGCFPCFGSSDKGGNGVKEVVKKESFKDGSAAVSHHVDRVSSDKSKSRGGNDPKKEPSIAKDGPPAHIAAQT
FTFRELAAATKNFRPECLLGEGGFGRVYKGRLESGQAVAVKQLDRNGLQGNREFLVEVLMLSLLHHQNLVNLIG
YCADGDQRLLVYEFMPLGSLEDHLHELPPDKEPLDWNTRMKIAAGAAKGLEYLHDKANPPVIYRDLKSSNILLDE
GYHPKLSDFGLAKLGPVGDKTHVSTRVMGTYGYCAPEYAMTGQLTLKSDVYSFGVVFLELITGRKAIDNTRGPG
EHNLVAWARPLFKDRRKFPKMADPLLQGRYPMRGLYQALAVAAMCLQEQAATRPLIGDVVTALTYLASQTYD
PNATSTQSNRVGPSTPRSREDRRSMADGVDSPDEPGRGFHGSPSAHKNSPDFRRRDSARELLNNGAELRKIET
GGGSGRKWGLDESERPESQRDSPLSAGRARETPRNRDLDRERAVAEAKVWGENWRERKKANNAAGSFDGTN
D

>A176|LOC105171481

MGCLPCFGSSKKEVNGSDNGVKELSKKESFKEGAAAQSNSRVNRVNSDKSRSRSNDSKKEPTIPKEPNIAAQTFT
FRELAAATKNFRPECLLGEGGFGRVYKGRLESTGQVVAVKQLDRNGLQGNREFLVEVLMLSLLHHPNLVNLIGY
CADGDQRLLVYEYMPLGSLEDHLHDLPPDKEPLDWNTRMKIAAGAAKGLEYLHDKANPPVIYRDLKSSNILLDE
GYFPKLSDFGLAKLGPVGDKTHVSTRVMGTYGYCAPEYAMTGQLTLKSDVYSFGVVFLEIITGRKAIDNTRGAGE
HNLVAWARPLFKDRRKFPKMADPLLQGRYPMRGLYQALAVAAMCLQEQAATRPLIGDVVTALTYLASQTYDP
NAPAAQGNKVGPSTPRHRHERRDTSDRLDSMDEPGYGGHYGSPSTHKNSPDFRKRESGRDFNTGGELRRIETG
TGSGRKWGLDELERPESQRGSPGSAGRARETPRNRDLDRERAVAEAKVWGENWRERKRTIAVDSFDSSYE

>A177|LOC109175002

MGGCFPCFGSSNKDGNGVKEVGKKESFKDGSAAQPNHVNKLSAEKSKSRNSNDSKKETAIAKDGSTANIAAQT
FTFRELAVATKNFRPECLLGEGGFGRVYKGRLESTGQVVAVKQLDRNGLQGNREFLVEVLMLSLLHHPNLVNLIG
YCADGDQRLLVYEFMPLGSLEDHLHDLPPDKEPLDWNTRMKIAAGAAKGLEYLHDKANPPVIYRDLKSSNILLDE

GYHPKLSDFGLAKLGPVGDKTHVSTRVMGTYGYCAPEYAMTGQLTLKSDVYSFGVVFLELITGRKAIDNTRAPGE
HNLVAWARPLFKDRRKFPKMADPLLQGRYPMRGLYQALAVAAMCLQEQAATRPLIGDVVTALTYLASQTYDP
NAGSGQSNRVGPSTPRSREDRRTMSDGVHSLDDPGHGGYHGSPSVHKNSPDYRRRDSGRDYNNTGAELRRIE
TGGGSARKWGLDESERPDSQRDSPLSAGRGKDTPRNRDLERERAVALAKVWGENWRDRKKANNASFDGTND

>A178|A0A3S4PTI0

MGGCFPCFGSSDEEKKVKNGVKGGEEGRKDARKEGSVSVSKVSLDKSRSGGGSDSKKEASVPKDGSNAHIAAQ
TFSFKELAATTKNFSQECLLGEGGFGRVYKGRLESTGQVVAVKQLDRNGLQGNREFLVEVLMLSLLHHPNLVNLI
GYCADGDQRLLVYEFMPLGSLEDHLHDIPPDKEPLDWNTRMKIAAGAAKGLEYLHDKANPPVIYRDLKSSNILLG
EGFFPKLSDFGLAKLGPVGDKTHVSTRVMGTYGYCAPEYAMTGQLTLKSDIYSFGVVFLELITGRKAIDNTRPGG
EQNLVAWARPLFRDRKKFPKMADPLLQGRYPMRGLYQALAVAAMCLQEQAATRPLIGDVVTALSYLASQTYD
PNAASAQSNRVGPSTPRAKDERKSLGGVPSSQDEMGHGGHRGVPSPKHSPDFRNRDSMPRVSFGADIGRGDS
GGGSGRKWGLDEHGSQRDSPVHAGKVKEGLKNFNRDLERERAVAEAKMWGENWRERKRSNASGSSEVANK

>A179|Spipo3G0040700

MGCFPCFGSSEQVKERRGEVKAGGDSKKDPSSTAPSSHHAARVGSEKLKSRSGTETRKETTAPKDGPTHIAAQIF
TFRELAAATKNFRQECLLGEGGFGRVYKGRLESTGQVVAVKQLDRNGLQGNREFLVEVLMLSLLHHPNLVNLIG
YCADGDQRLLVYEFMPLGSLEDHLHDIPPDKEPLDWNTRMKIAAGAAKGLEYLHDKANPPVIYRDFKSSNILLDE
GFHPKLSDFGLAKLGPVGDKTHVSTRVMGTYGYCAPEYAMTGQLTLKSDVYSFGVVLLELITGRKAIDNARAAG
EHNLVAWARPLFKDRRKFPKMADPLLQGRYPMRGLYQALAVAAMCLQEQAATRPLIGDVVTALSYLASQSYD
PNAAGAHNSRAGGGPSTPRGREDWKSLSVGGDSQLAAFSPARNSPDFRKRDLARGVSFGAEVGRGELGGGSG
RKTALVEDLEGEESQRGSPMNAPKARNGPRNANRDADRERAIAEAKVWGENWRDRRHANAQGSFDSTHE

>A180|A0A4S4DNN2

MGGCFPCFGSSNKDGNGVKEVGKKDSVKESSAAQSHHVTRVGSDKSKSRSNSDPKKDPKKEPAVQKDGPTAHI
AAQTFTFRELAAATKNFRPECLLGEGGFGRVYKGRLESTGQVVAVKQLDRNGLQGNREFLVEVLMLSLLHHQNL
VNLIGYCADGDQRLLVYEFMPLGSLEDHLHDLPPDKEPLDWNTRMKIAAGAAKGLEYLHDKANPPVIYRDLKSS
NILLGEGYLPKLSDFGLAKLGPVGDKTHVSTRVMGTYGYCAPEYAMTGQLTLKSDVYSFGVVFLELITGRKAIDNT
RAAGEQNLVAWARPLFKDRRKFPKMADPLLQGCYPMRGLYQALAVAAMCLQEQAATRPLIGDVVTALTYLAS
QTYDPSAPTGHSNRVGPSTPRSRDDRRSMANGLDSPDESRRGGRYGSPSNHKNSPDFRKRDSARELNAGAELG
GSETGGGSGRKWGMDEVERQDSQRDSPISAGRARETPRNRDLDRERAVAEAKVWGENWREKKTSKRNG

>A181|104000072

MMKCLPCLASPGQGDAADEVKEEEKKKKKNDEGGDGAVRKESSAAPTSRLGSLDKSKLRADSKKEPSVPKEGN
PEHIAAQTFSFRELSAVTKNFRRDCFLGEGGFGRVYKGRLETGQVVAVKQLDRNGLQGNREFLVEVLMLSLLHH
PNLVNLIGYCADGDQRLLVYEFMPLGSLEDHLHYIPANKEPLDWNSRMKIAAGAAKGLEYLHDKANPPVIYRDF
KSSNILLGEGYHPKLSDFGLAKLGPVGDNTHVSTRVMGTYGYCAPEYAMTGQLTVKSDVYSFGVVFLELITGRKA
IDNARPTGEQNLVAWARPLFKDRRKFPKMADPLLQGRYPMRGLYQALAVAAMCLQEQAATRPLIGDVVTALS
YLASQNHDPIAASIQSTKVGPSIPSSRSGSGDQHAVQSPHQNSSELKQRGPLKGASKGAKIGRDGSSGGSGRKW
SMEESETRESQMDSPVQVERARDSPKNIDRDFVRERAVAEAKEWGENWRERQRRNAQGSSDRTHE

>A182|LOC104827116

MSGCLPCFGSSTKDAAAKESAKKDASAKDGSVTQSHYVSSDKSKSKGGSEHKKDATTAPKDGPPANIAAQTFTF
RELASATQNFRPECLLGEGGFGRVYKGRLDSTGQIVAVKQLDRNGLQGNREFLVEVLMLSLLHHPNLVNLIGYCA
DGDQRLLVYEYMPLGSLEDHLHDLPPDREPLDWNTRMKIAAGAAKGLEYLHDKANPPVIYRDLKSSNILLGEGY
HPKLSDFGLAKLGPVGDKTHVSTRVMGTYGYCAPEYAMTGQLTLKSDVYSFGVVFLELITGRKAIDNTRAPGEH
NLVAWARPLFKDRRKFPKMADPLLQGRYPMRGLYQALAVAAMCLQEQAATRPLIGDVVTALTYLASQTYDPN
SPSAQSSRGGQGGQPSIRTARDDRRSTGDGSSLDSPASTRSRLGSPATHRNSPDYRRRDASMGSELGRSETGGG
SGRKWGLGDMEGPESQRDSPVSVGRGRNRDLDRERAVAEAKVWGENWRERKRASNAASFDSTNE

>A183|102717901

MERNQHGGSCVKTISALTFVGCPRRLLPPRSDISCTLSSLYRPSLPLSLQIADRPPPNSTPDPLHPSKIGFWKGPPP
TPDQGEIPVSSDKSNSQGGFDSRKDSIMPRDGNGQHIAAHTFTFRDLAAATKNFRQDCLLGEGGFGRVYKGHL
ENGQDVAVKQLDRNGLQGNREFLVEVLMLSLLHHDNLVNLIGYCADGDQRLLVYEFMPLGSLEDHLHDIPPDK
EPLDWNTRMKIVAGVAKGLEFLHDKANPPVIYRDFKSSNILLGQGYHPKLSDFGLAKLGPVGDKTHVSTRVMGT
YGYCAPEYAMTGQLTVKSDVYSFGVVFLELITGRKAIDNTKPLGEQNLVAWARPLFKDRKKFPKMADPLLAGRF
PMRGLYQALAVAAMCLQEQAATRPFIGDVVIALSYLASQTYDPNAPVQHSRSNASTPRARPRGGGNGDQRRL
HSPDSGCGSGRRLGLDNMDTNSSQMGSPAYTGKKRESSRGTVRPRAIAEAKTSGENSRERK

>A184|A0A2R6P4D9

MGGCFPCFGSSNKDSNGVKEVAKKDSVKEGSANQSHHVTRVSSDKSKSRSGSDPKKDPKKEPAVQKDGPTAHI
AAQTFTFRELAAATKNFRPECLLGEGGFGHVYRGQLESTGQAVAVKQLDRNGLQGNREFLVEVLMLSLLHHPNL
VNLIGYCADGDQRLLVYEFMPLGSLEDHLHDIPPNKGPLDWDTRMKIAAGAAKGLEYLHDKANPPVIYRDLKSS
NILLDEGYHPKLSDFGLAKLGPVGDKTHVSTRVMGTYGYCAPEYAMTGQLTLKSDVYSFGVVFLEIITGRKAIDN
NRAAGENNLVAWARPLFKDRRKFPKMADPLLQGRYPIRGLYQALAVAAMCLQEQAATRPLIGDVVTALTYLAS
QTYDPNAPPAQSNRIGPSTLRNKDDRRNIPDGLASHHGSPSNHRNSPDFRKRESSAGPELGKSESGGGSGRKW
GVEVENQEYQRDSPGSAGRARETPRNRDLERERAVAEAKVWGENFREKKRVNAMGSFDGTNE

>A185|Tp57577

MGCFSCFDSKEDEKLNPNPQQQQQQTHHHDHILPSHVSRLPSAPSAGPDKLRSTSNGGASKREFTAALKDGPP
AQIAAQTFTFRELAAATKNFRPQSFLGEGGFGRVYKGRLESTGQVFYYYNLFNSSLIQIIWIGDHSLLMFVPIPMQ
AVAVKQLDRNGLQGNREFLVEVLMLSLLHHPNLVSLIGYCADGDQRLLVYEFMPLGSLEDHLHDLPPDKEPLDW
NTRMKIAAGAAKGLEYLHDKANPPVIYRDFKSSNILLDEGYHPKLSDFGLAKLGPVGDKSHVSTRVMGTYGYCAP
EYAMTGQLTVKSDVYSFGVVFLELITGRKAIDSTRPHGEQNLVTWARPLFNDRRKFPKLADPRLQGRYPMRGLY
QALAVASMCIQEQAAARPLIGDVVTALSYLANQAHDTNNAGIGNNKGTGDDKRSRDDKGGRILQNDEGGGSG
RRWDLEGSEKDESPRETARMLNRDLDRERAVAEAKMWGENWREKRRQSVQGSFDASNA

>A186|ALR83541.1

RGCPPLLTLDVSENLLTGSISSGLGLCQRLTLLNLSGNKLNGSIPIEFASLPVLATLGLSHNNLSGSIPSDFQNSTALE
VFDVSYNNLSGIVPSSGLFKIINPGMLVGNADLCGGTLGACATLEDEIPQPHHVSNKKSLGLMAWVITAIFAIALV
VLMASFRCFYKQYKWYICSKLKQDGDEDGWKLTAFQRLSFTAEDIVQCLTTNNIIGMGAAGTVYRADVPSGEVI
AVKKLWRSQKEVDVKDEQILGEVDVLGSVRHRNIVRLLGCCANSETTLLLYEYMPNGSLGDLLHGNKEVNILAD
WMTRYKIAMGVAQGLCYLHHDCFPVVVHRDVKSNNILLDSNMEARVADFGVAKLIEETESMSVIAGSYGYIAP
EYAYTMQVDEKSDIYSFGVVLLELLTGKRPVEAEYGDAVNIVEWVRVKVQGGEDAAAVEVLGGEGAALDWVRE
EMMLVLRVALLCTSKAPIDRPSMRDVVTMLSEAKPRRKHSQDSTSLTTKRAL

>A187|103995349

MVCFPCFRSPGQGKEKEVKKKIEGIGGADCKQEFSAAPSSHHGSSDKSKLRTDSVSKTGASTPKEGNAGNIAAQ
TFTFRELATATKNFRRDCLLGEGGFGRVYKGRLETGQVVAVKQLDRNGLQGNREFLVEVLMLSLLHHPNLVNLI
GYCADGDQRLLVYEFMPLGSLEDHLHDIPADVEPLDWNTRMKIAAGAAKGLEYLHDTANPPVIYRDFKSSNILL
GKGYHPKLSDFGLAKLGPVGDKTHVSTRVMGTYGYCAPEYAMTGQLTVKSDVYSFGVVLLELITGRKAIDNSRP
VGEQYLVAWARPLFKDRRKFAKMADPLLQGHYPTRGIYQALAVAAMCLQEQAATRPLIGDVVTAVSYLASQNY
DPIAASTRSTRNGPSTPRSTSRIDNQHAVHSPHQNSPDSRQRDPIMIACEDAKIGIGSSGGCSGHTWVLEELETR
ESQMDSPVQVGSVRDSPKDICHNLDRERAVAEAKEWGETWRERQRKNTPGSFHSTT

>A188|103998241

MMGCFPCFGSASQREEEEEEVKKRNEGKGGGGFKGASLSHHGGSDKLRSRTGSDSKKEASTPKESNAGHIAAQ
TFTFRELAAATKNFRQECLLGEGGFGRVYKGRLENGQVVAVKQLDRNGLQGNREFLVEVLMLSLLHHPNLVNLI
GYCADGDQRLLVYEFMPLGSLEDHLHDIPADKEPLDWNIRMKIAAGAAKGLEYLHDKANPPVIYRDFKSSNILLG
EGYHPKLSDFGLAKLGPVGDKTHVSTRVMGTYGYCAPEYAMTGQLTVKSDVYSFGVVFLELITGRKAIDNTRPSG
EQNLVAWARPLFKDRRKFPNMADPLLQGRYPMRGLYQALAVAAMCLQEQAATRPFIGDVVTALSYLASQTYD
PNAASVQRTRFGSSTPRSRSGSDNQQAVHSPHQNSPDLRQRDPFKAGGKGAKVGRGGSAGGSGRKWGLDDL
ETQESQMDSPVHVGVAEGSPKTVDPNLVREHAVAEAKLWGENWRERQPRNTPGNYDSTHE

>A189|LOC109175864

MGCFPCFDSREEETLNPQKERDDRKEVHPVVPSHMSRLSSGADRLKSRGNTGSKRETSGLKDLPGVQIAAHTFT
FRELAAATSNFRPESFIGEGGFGRVYKGRLANGQAVAVKQLDRNGLQGNREFLVEVLMLSLLHHPNLVNLIGYC
ADGDQRLLVYEFMPLGSLEDHLHDLPPDKEPLDWNTRMRIAAGAAKGLEHLHDKANPPVIYRDFKSSNILLDEG
FSPKLSDFGLAKLGPTGDKSHVSTRVMGTYGYCAPEYAMTGQLTVKSDVYSFGVVFLELITGRKAIDSTQPPGEQ
NLVAWARPLFNDRRKFAKLADPRLQGQFPMRGLYQALAVASMCIQEQAAARPLIGDVVTALSYLANQAYDPS
AAPGFKDDKRGPRISRNEDGSGGGSGRKWDLDGSEKEDSPRETARMLNRDAERERAVAEAKMWGENFREKR
RQNGELEGGSEKEDSPRETVKMLNRDLDRERAVAEAKMWGENFREKRRQNTHAGFEGNNG

>A191|03977711

MGCFTCFGSPSQREEEEEVKKKRREGKGGGDRKREPSSRRGSSDKLTARTGSDSKKEASASKEGNAGHIAAQTF
TFRELAAATKNFRQDCLLGEGGFGRVYRGRLENGQVVAVKQLDRNGLQGNREFLVEVLMLSLLHHPNLVNLIGY
CADGDQRLLVYEFMRLGSLEDHLHDIPADKEPLDWNTRMKIAAGAAKGLEYLHDKANPPVIYRDFKSSNILLSEG
YHPKLSDFGLAKLGPVGDKTHVSTRVMGTYGYCAPEYAMTGQLTVKSDVYSFGVVFLELITGRKAIDNTRPAGE
QNLVAWARPLFKERRKFPKMADPLLQGHYPMRGLYQALAVAAMCLQEQAATRPLIGDVVTALSYLASQTYDQ
NAATAQNTRVGPSTPRSRSGFDNQQAVHSPHRNSPDLRQRDLVKGVSKGAKHGRGGSGGGSGRKWGLDELE
TQESQMDSPVHVGRARDSPKNIDRDVVREHAVAEAKVWGENERERQQKNALGIFNGTHE

>A191|108196251

MGCFPCFGSSEDDGKNGEQEVVKKDSFKEGSAPQSYHMTRVGSDNTKSRGGFDPKKELPLPKDVPTPHIAAQT
FTFRELAAATKNFMPECLLGEGGFGHVYKGRLESTGQVVAVKQLDRNGLQGNREFLVEVLMLSLLHHPNLVNLI
GYCADGDQRLLVYEFMPLGSLEDHLHDLPPNKEPLDWNTRMKIAAGAAKGLEYLHDKANPPVIYRDLKSSNILL
GEGYHPKLSDFGLAKLGPVGDKTHVSTRVMGTYGYCAPEYAMTGQLTLKSDVYSFGVVFLELITGRKAIDNART
HGEHNLVAWARPLFKDRRKFPKMADPLLQGRYPMRGLYQALAVAAMCLQEQAATRPLIGDVVTALTYLASQA

YDPNASVSQSSRVGSSTPRSRDRRSTHDAVDSPGHNGSPTTHKNSPDFRKRDLMTNSRNGETSGGSGRRWAV
DDLEYTESQRDSPVSAGRTREPRNRDLDRERAVAEAKVWGENLREKKQGGAMDSFDATNE

>A192|A0A4Y7J4Z4

MGRCLPCFGSSDEKNHHQSGETKKEFVKDGSTTGQSNHINRVSSEKKSRGGSDSKKETPVPKDGPSAPIAAQTF
TFRELAAATKNFRPESLLGEGGFGRVYKGRLESTGQVVAVKQLDRNGLQGNREFLVEVLMLSLLHHTNLVNLIGY
CADGDQRLLVYEFMPLGSLEDHLHDVPPNKEALDWNTRMKIAAGAAKGLEYLHDKANPPVIYRDFKSSNILLDE
GFHPKLSDFGLAKLGPVGDKTHVSTRVMGTYGYCAPEYAMTGQLTLKSDVYSFGVVFLELITGRKAIDQTRLQGE
QNLVAWARPLFKDRRKFPKMADPLLQGRYPMRGLYQALAVAAMCLQEQATTRPLIGDVVTALSYLASQTYDP
NAVQNNRGGSSRDKEDRKSHADLSESQKNSPRYQDSPGSRRRDPVQQGMNFGPEVGRGESGSSGRKWGLEE
FDRQESQKNSPAHGGKTPRSSSRGPDRERAVQEAKVWGENWRERKRTDPNGSFDRTNE

>A193|A0A103XRM2

MGGCFPCFGSSNKEGNGVKEVSKKDIGKEGSATHQSHHVSRVSSGKSKSRVNLDPKKDQMVTKDGQTANIAA
QIFTFRELAAATKNFRPESLLGEGGFGRVHKGRLESTGQVVAVKQLDRNGLQGNREFLVEVLMLSLLHHPNLVN
LIGYCADGDQRLLVYEFMPLGSLEDHLHDLPPDKEPLDWNTRMKIAAGAAKGLEYLHDKANPPVIYRDLKSSNIL
LDEGYHPKLSDFGLAKLGPVGDKTHVSTRVMGTYGYCAPEYAMTGQLTLKSDVYSFGVVFLELITGRKAIDNTRA
PGEHNLARPLFKDRRKFAKMADPLLQGRYPVRGLYQALAVAAMCLQEQAATRPLIGDVVTALTYLASQTYDPE
AARTERSNRGSGSGTPRSRRNPSEGSGGGGLDSLDPQGSPSAFKNSPDYRKIGGDGESSNGGGGSGRKWAVA
EDLDGSQRNSPANTSRGRNRDRDLERERAVAEAKVWGENWRERKRTNAMGSFDATNE

>A194|A0A2R6P373

MGCFSCFDSREVEKLNLDKQSDHRKEIRPSVSSNISRLSSGEDRLKSRSNVGLRKESSGPKDAPDVNIAAQTFTFR
ELATATKNFRPESFIGEGGFGSVYKGRLESTGQVVAVKKLDRNGLQGNREFLVEVLMLSLLHHPNLVNLIGYCAD
GDQRLLVYEFMPLGSLEDHLHDLPLNKDPLDWSTRMKIAAGAAKGLEYLHDKASPPVIYRDFKSSNILLDEGFHP
KLSDFGLAKLGPMGDKSHVSTRVMGTYGYCAPEYAMTGQLTVKSDVYSFGVVFLELITGRKAIDSTRPQGEQNL
VAWARPLINDRRKFGKLADPGLEGQFPIRGLYQALAVASMCTQEQAAARPLIGDVVTALSYLANQSYEPNSAPG
HISRFNGDKDERRTGGRISKNEEGGGSGRKGDVEGSERDDSPRETAKMLNKDLDRERAVAEAKMWGENWRE
KRPERDDSPRETAKMLNKDLDRERAVAEAKMWGENWREKRRQSAQGSFDGTNR

>A195|LOC111472284

MGGCFPCFGSSDRNGAKEATKKDSAKDGATAQSHRVSRVSSDKSKSWSVSDAKNEPTVQKDGTTAHIAAQTF
TFRELATATKNFRPECLLGEGGFGRVYKGRLESTGQAVAVKQLDRNGLQGNREFLVEVLMLSLLHHPNLVNLIGY
CADGDQRLLVYEFMPLGSLEDHLHDFPPDKEPLNWNTRMKIAAGAAKGLEYLHDKANPPVIYRDLKSSNILLDE
GYHPKLSDFGLAKLGPVGDKTHVSTRVMGTYGYCAPEYAMTGQLTLKSDVYSFGVVFLELITGRKAIDNTRGPG
EHNLVAWARPLFKDRRKFPKMADPLLQGRYPTRGLYQALAVAAMCLQEQAASRPLIGDVVTALTYLASQTYDP
NTAASQSNRMSVSTPQSRDERRGLPDGLDSPDERGHGCCSPTNYRNSGGTELSRIDTGGGSGRKWELDELEQQ
ESLRDSPAYTGRARETQKNRDVNRERAVAEAKMWGENWKERNRANVQGSFDSS

>A196|A0A078IUE7 Brassica napus

MVDKEKKKTFLLRMESQFPSFLSGYSQSFWLFRSCSKGLCKFLITILHPLCQTLSGRRWVVSRVFIRVKESKPQKQS
QTIVSNNFSTLPSGGEKKLSSKSNVRSKRELLLPRDGLEQIAAHTFAFRELVAATMNFHPDTFLGEGGFGCVYKGR
LESTGQVVAVKQLDRNGLQGNREFLVEVLMLSLLHHPNLVNLIGYCADGDQRLLVYEFMPLGSLEDHLHDLPPD
KEALDWNTRMKIAAGAAKGLEFLHDKANPPVIYRDFKSSNILLGEGFHPKLSDFGLAKLGPTGDKSHVSTRVMG

TYGYCAPEYAMTGQLTVKSDVYSFGVVFLELITGRKAIDSEMPHGEQNLVAWARPMFNDRRKFIKLADPKLKGR
FPTRALYQALSVASMCIQEEAATRPLIADVVTALSYLANQAYDPNKNERGGGKLITRDDEGGGSEKEDSPRETAR
ILNRDIDRERAVAEAKMWGESLREKRRQSEQGTSESNSTGSK

>A197|108199240

MGGCFPCFRSSEEAVKEVVKKDSLKEGAAAQPHHVSLDKSKSRGGLDPKKEPTLPKDGPTAHIAAQTFTFRELAA
ATKNFRPECLLGEGGFGRVYKGRLESTGQVVAVKQLDRNGLQGNREFLVEVLMLSLLHHPNLVNLIGYCADGD
QRLLVYEFMPLGSLEDHLHDFPPNKEPLDWNTRMKIAAGAAKGLEYLHDKANPPVIYRDLKSSNILLDEAYHPKL
SDFGLAKLGPVGDKTHVSTRVMGTYGYCAPEYAMTGQLTLKSDVYSFGVVFLELITGRKAIDNTRTHGEHNLVA
WARPLFKDRRKFPKMADPLLQGRYPMRGLYQALAVAAMCLQEQAATRPLIGDVVTALTYLASQAYDPNAPVS
QSSRGGTPRSRDRRSLADGVDSPGHYGSPSTHRNSPDFRKRDSVTESRNGETSGGSGRRWALEELERSDSLRDS
PVSAGRARETRNRDLDRERAVAEAKVWGENLREKKRGGAMDSFDATNE

>A198|1088818_0:0025a6 PKA57651.1

MSCFPCFGSSEKGEGKKVVVKTGAEDFKLTPAAAPTSQVTRVTSDRSTTRSASEPRKEESLVTNGGNTTHIAAHT
FSFGELAAATKNFRPECLLGEGGFGHVYKGRLEDGQVVAVKQLDRNGLQGNREFLVEVLMLSLLHHSNLVNLIG
NCIGSSCSFITCRHLIKFSPFVLGLPVLSFRVDLPPNKDPLDWNTRMRIAAGAAKGLEYLHDEANPPVIYRDLKSSN
ILLGEGYHPKLSDFGLAKLGPVGDKTHVSTRVMGTYGYCAPEYAMTGQLTLKSDVYSFGVVFLEIITGRKAIDSTK
PAGEQNLVAWARPLFKDRRKFPKMVDPMLHGHYPMRGLYQALAVAAMCLQEQAATRPLIGDVVTALSYLAS
QTYDPSTPSAALSSKVGPSTLRVREERKSHGGNFTHSPNMYSPKFRQRDAVKELSSEDENGGSGQQEQPMDSL
KALDGKTDRQQAVAEAKVWGENLREREQSNSPRSFDASEMDGSC

>A199|A0A5A7RGI9

MGGCFPCFGSSKKAVNGSNSSNGVKEVAKKASFKENSSVHSSSHVKRVNSDKSKGQKSTDTKKELLLPKEPTANI
AAQTFTFRELAAATNNFRPECLLGEGGFGRVYKGRLESTGQLFQCCILTLELEKIVAWLLVTMVVAVKQLDRNGL
QGNREFLVEVLMLSLLHHPNLVNLIGYCADGDQRLLVYEYMPLGSLEDHLHDLPPDKEPLDWNTRMKIAAGAA
KGLEYLHDKANPPVIYRDLKSSNILLDEGYFPKLSDFGLAKLGPVGDKTHVSTRVMGTYGYCAPEYAMTGQLTM
KSDVYSFGVVFLELITGRRAIDNMRSAGEHNLVAWARPLFKDRRKFPKMADPLLQGRYPMRGLYQALAVAAM
CLQEQAATRPLIGDVVTALSYLASQTYRHQRRDTSDGGPHLGSPSSSVQKNSPEDSSRRMIEETVGRAHSQVGS
PGSAGRAHEMPKNPERERAVALAKVWGENFREKRKSDAGSFDSTNE

>A200|OAE29636.1

MRPGPLAFRKQTQEPEMGCFSCFGTRKQDNRKPLKKDDSGREGQSTGGSANNNLTKVPSENKTKGKGSFDAK
KEVPTADGATHIAAQTFTFRELAAATKNFRPECLLGEGGFGRVYKGRLENTGQANLPPDKEALDWNTRMKIAA
GAARGLEYLHDKANPPVIYRDFKSSNILLDEGFHPKLSDFGLAKLGPVGDKTHVSTRVMGTYGYCAPEYAMTGQ
LTLKSDVYSFGVVLLELITGRKAIDNARAPGEHNLVAWARPLFKDRRKFPSMADPMLQGRYPMRGLYQALAVA
AMCLQEQAATRPLIGDVVTALSYLASQTYDPGLHPVGSSRFAPPTPSREKKEKDKKLGSGGSERHGNSPMLPST
DERVQKERGGRQAGSRSPSNLPSTASPDLRKDSGRGRNNGTFDGRGQDTGAARKRDSDEWDRYDSHRESPG
HTGRTPKDARSLAPSRERERAVAEARVWGENWRERKRSNNQGGGDGFDGGFG

>A201|LOC109851463

MGCFPCFGSSSDKEKEEGKKGDLKDLKKDNSSTHHVTRVSSDKSKSRSGSDSKKDAAVIKDGNSAHIAAQTFTFR
ELAAATKNFRPECLLGEGGFGRVYKGRLENTGQVVAVKQLDRNGLQGNREFLVEVLMLSLLHHPNLVNLIGYCA
DGDQRLLVYEFMPLGSLEDHLHDIPPEKKPLDWNTRMKIAAGAAKGLEYLHDKANPPVIYRDFKSSNILLDEEFH

PKLSDFGLAKLGPVGDKTHVSTRVMGTYGYCAPEYAMTGQLTLKSDVYSFGVVFLELITGRKAIDNTRAAGEQN
LVAWARPLFKDRRKFAKMADPLLRGHYPMRGLYQALAVAAMCLQEQATTRPLIGDVVTALSYLASQTYDPNAV
NSSRSRPSTPRLREDRKAHLPHTNSPDIRQKDPFKGMNLGAEVGRGDSGGDSGRKWDFDESDAISSYQDSPVH
GGKSKRSMGQDHARARAVAEARVWGENWRERKRSEFGSFDGANE

>A202|LOC107471145

MASFKELSMGGVVLNNSRRNSSKERKKKGDEDDLGLKRKLKLWFGCMTLILRSKAKDSATDHKYKADGIEEKSI
GPISSTPSNSPNFHGPGGSSITTPIVIIDEESVVNPKSFTYKQLKLATKNFKREHFLGEGGFGSVFKGWLSRDDAMT
PTKPGMGIPVAIKSLNQFGLQGHQEWLAEVNYLSQLKHPNLVKLIGFCNEDDKMLLVYEFMPRGSLESHLFRRG
VVFPWSIRVRIMLDAAKGLAYLHEKTQNKNAVIFRDFKTSNILLDKEFHAKLSDFGFARDGPEGDKTHISTRVMG
THGYAAPEYVMNGHLSAKSDVYSFGVVMLEMVTGRKVIDRQRAHSEMNLVRWALPLIRDNGRNSHILLIDPRL
HGHYSPKASLKALKLASRCLRFNPSMRPTMDSVVQSIINFPQEASPLPIPPPPHLNTDSNKYGLVSSASASRTDVP
TRFQASPCHLNHHHQAHVASTSSTPARPNVTIDQRR

>A203|A0A078FLA4

MSGCLPCFGSSAKDAASKDSVKKEASAKAKDASVTQSHHVSLDKSKSKGGSEQKKELTAPKEGPTAHIAAQTFTF
RELAAATKNFRPDCLLGEGGFGRVYKGRLETTGQIVAVKQLDRNGLQGNREFLVEVLMLSLLHHTNLVNLIGYCA
DGDQRLLVYEYMPLGSLEDHLHDLPPDKEPLDWNTRMTIAAGAAKGLEYLHDKANPPVIYRDLKSSNILLGDGY
HPKLSDFGLAKLGPVGDKTHVSTRVMGTYGYCAPEYAMTGQLTLKSDVYSFGVVFLELITGRKAIDNARAHGEH
NLVAWARPLFKDRRKFPKMADPSLQGRYPMRGLYQALAVAAMCLQEQAATRPLIGDVVTALTYLASQTFDPN
AASSQNSRSGSGGGGPPSLDSPAETRSRLGNRDLDRERAVAEAKVWGENWRERKRGINGPGSFDSEGREGSYE
TFVESGRVGQLSDLMSESEERLFNIVVEAQEHDANDKRLGN

>A204|LOC108344550

MGRISGVRVGSSIGGKSKGGRKNKDVVQSSSGEGSRFWKKLRTFLGCMFWSSKGEDSSINKINQVDHGRHVTN
DVDRQQPIVPRSSSNRRISSHTRLSVPDQELFEASLLLRRFAFQDLMLATRTFKVENFHDEKGFGVLLKGWINPY
GNYAARPGKGIPIAVKALNLNECQDKNEWLDEIIYLSKLQHLNLVRLVGFCLEDDKRLLVYEHICEGSLEKHLFKSA
VHLTWPTRMKIAIGAANGMSFLHEEASRPLIFGDFKTSSILLDKDYNTKLCDFGLAKDASMGHEMRTTKMVGSK
GYEAPEYIMTGLLTPKSNVYSFGLVLLELLTGRRVIDKTMPIEEQNLIEWLGPRLGNKANFHYLMDPRLEGQYPTK
FAHKTMKLAIHCLRLDPKTRPLMSEVLHELKSLHDDMLGSSTSHGRRHKGPSNHVSPNKYGVGICPSSNFPRCF
QAMPECQDYPLPLPPSPSNPRVRSSSNPSLEIYST

>A205|A0A5A7PJW0

MGGCIPCFGSSKKAVSSGNSGNVVKEFAKKESFKDKSAVSSNSHVNGVNSDKSKGRRSTDTKKELVLPKEPTANI
AAQTFTFRELAAATKNFRPECLVGEGGFGRVYKGRLESTGQVVAVKQLDRNGLQGNREFLVEVLMLSLLHHPNL
VNLIGYCADGDQRLLVYEYMPLGSLEDHLHDVPPDKEPLDWNTRMKIAAGAAKGLEYLHDKANPPVIYRDLKSS
NILLDEGFFPKLSDFGLAKLGPVGDKTHVSTRVMGTYGYCAPEYAMTGQLTLKSDVYSFGVVFLELITGRRAIDNT
RAAGEHNLVAWARPLFRDRRKFPKMADPLLHGHYPMRGLYQALAVAAMCLQEQAATRPLIADVVTALSYLAS
QTYDPNSPTLKHSRHSRKNTSDSLDGPHMGSPSSAHKNSPDLRRIETRVGSGSRLGPVECQLDSPGSSGRAGET
PKNPERARAVALAKVWGENFREKKKGNATDSFDSTNE

>A206|LOC110033478

MPCFFPCFDWNDEAAEFVGGGRQEDEKREEKPMMPPSVDRLPAGNGELNARRCFDSKREHVNAFKKEPANA
LHKRQLDSSKLEALNQNKCSGSDFSARTFTFRELAAATKNFRQECFLGEGGFGSVHKGRLETGETVAVKLLDRNG

LQGNKEFLVEVLMLSLLHHPNLVNMIGYCADGDQRLLVYEYLPLGSLENHLHDLPPDKDSLDWNTRMKIAAGA
ANGLDYLHNEASPPVIYRDLKSANILLDGDFNPKLSDFGLAKLGPTDDKSHVSTRVMGTYGYCAPEYAMTGQLT
VKSDVFSFGVVLLELITGRRAFDSTRPHGEQNLILWVKPLLNDRRRLSKVADAKLQGRYPMRGLYQALAVASMCI
QEHPAARPSVKDIVTALSYLASHPYDPSTSPNSSLQRGEVEKGRREAAERIKTYLDDQKLPTAERDDSPKETATFL
NHQNFDRERAVAEAKMWGANWREKRMANVDPGPANSDS

>A207|PIA38762.1

MGWFPCSGKSIEKTRKKRSDVDPIKPTVSDKMKIYAPLSLKKEEPKVVASDKLINPPLTAKKEEPKDEHQTAQTFT
FHELESATKNFREESLLGEGGFGRVYKGRLESTNQVAAIKQLDRNGMQGNKEFLVEVLMLSLLHHPNLVNLIGYC
ADGDQRLLVYEYMPLGSLEDHLHDLPPNKKPLDWNARMKIAAGAAKGLEYLHDTANPPVIYRDLKCSNILLGEG
YYPKLSDFGLAKLGPVGDKTHVSTRVMGTYGYCAPEYAMTGQLTLKSDIYSFGVVLLEIITGRKAIDNSKSAAEQN
LVAWARPLFRDRKKFSQMADPKLQGVYPMRGLYQALAVAAMCVQEQASLRPVIADVVTALGYLASQLYKPET
ETVHSPRMAPSASPRPKKVNTETESAQSSRMTPSTPPRPKKVSTETESVQSSRVAPSTPPRPKKVNTETESVQSS
RMAPSTPPRPKKFNAKQLNGGSLSVINQTRGMN

>A208|A0A5J9TXY7

MGCFSCFGSGREEGRHGAKGGGNGAAASSSAVGGGGRRQEEAAAVTAAPRAERIPAGADKARAKGNASSRK
ELSVLKDANGNTISAQTFTFRQLAAATRNFREECFIGEGGFGRVYKGRLDGSGQVVAIKQLNRDGTQGNKEFLV
EVLMLSLLHHQNLVNLVGYCADGDQRLLVYEYMPLGSLEDHLHDLPPDKEPLDWNTRMKIAAGAAKGLEYLHD
KAQPPVIYRDFKSSNILLGNGFHPKLSDFGLAKLGPVGDKSHVSTRVMGTYGYCAPEYAMTGQLTVKSDVYSFG
VVLLELITGRKAIDSTRPPGEQNLVSWARPLFNDRRKLPKMADPGLAGRYPTRGLYQALAVASMCIQSEAASRPL
IADVVTALSYLANQTYDPNAANTSKKAGGGDQLSRVVDSGRALSKNDDSGSSGHKSPNKDREDSPRERLRIANK
DLDRERMVAEAKMWGENWREKRRAAASTQGSQDSPTSGG

>A209|LOC100845438

MSCFPCSGSSGKAGEEAAAAALSPSPRPAAKAPPDQSNSRRSGSKKEDSVRRGGSIAHGPAKIFTFRELAVSTKN
FRRDCLLGEGGFGRVYKGHMENGQVIAVKQLDRSGFQGNREFLVEVLMLSLLHHANLVRLIGYCADGDQRLLV
YEYMLLGSLENHLHDRPPNKEPLDWNTRIRIAFGAAKGLEYLHDKANPPVIYRDFKPSNILLGEDYYPKLSDFGLA
KLGPVGDKTHVSTRVMGTYGYCAPEYAMTGQLTVKSDVYSFGVVFLELISGRRAIDHTQPDGEANLVAWARP
MFRDRTRFCQIVDPLLQGRYPQRGLYQALAVTAMCLLEHAASRPLIKDVVSALGFLDKHPYDPNAPRRKDSKTC
PSTPRAKAHRRTTSVPDAQHAADSLNWNAVDLRRKKIIGGGEFEQDHSEGCGSGSSSGRNECLDVPALLALDN
GKLFGEADITRKSTVKVDAREKLRADSGKYRRQV

>A210|102721589

MGFLACLFPCPQEEEDDDGEEPRSGQRVSSVEYSKSSESCPLKTERSIDMIGIRRNRGHGEATIFTLRELADATKN
FSEECLLGRGGFGSVYKAYLNDRQVVAVKQLDLNGLQGNREFLVEVLMLSLLHHPNLVHLFGYCVDGDQRLLIYE
YMPLGSLEDHLHDLPPGQGPLDWTTRMKIAADAAAGLEYLHDEARPAVIYRDIKPSNILLGEGYQAKLSDFGLAK
LGPVGDKTHVTTRVMGTHGYCAPEYLSTGKLTIKSDIYSFGVVFLELITGRRALDSNRPPEEQDLVAWARPLFKDK
RKFPKMADPLLHGRFPKRGLFQALAIAAMCLQEKAKNRPSIREVATALSYLASQTYERHSTPAPRHNPAGPSVPR
ALLDDQIDQDTSFPNQNGAHMSVHTGTSHMVQEEVKENCWSSSHRSGRGRVAPNGVDRERALADANIWVE
AWRRQEKTSKMRLPTYILKNGSDCIKSC

>A211|A0A443NNF5

MGCFSCFDSKEEGELNPGNRRDGTNEGKAMVESHISRLSSGSDRLKARSATGLRRESLGPKDGPTGSIAAQTFTF
RELAAATKNFRPECLLGEGGFGRVYKGRLESTGQVVAVKQLDRNGLQGNREFLVEVLMLSLLHHPNLVNLIGYC
ADGDQRLLVYEYMPLGSLEDHLHDLPPDKEPLDWSMRMKIAAGAAKGLEYLHDKANPPVIYRDLKSSNILLDEG
YLPKLSDFGLAKLGPTGDKSHVSTRVMGTYGYCAPEYAMTGQLTVKSDVYSFGVVFLELITGRKAIDSTRAHGEQ
NLVSWARPMFNDRRKFPKLADPLLQGHYPMRGLYQALAVASMCIQEQAATRPLIADVVTALSYLASHAYDPNT
APAHSLRASGEKEERRSRGTTEKQDERGGRTGRKEEGVSGHKWDLDSFEREDSPKETAQNLNRDLDRERAVAE
AKLWGENWREKRRANVKGNFDAPNGSIRFGP

>A212|A0A5J5C3J9

MGGCFQCFGSSNKEGNSVKEAAKKDSVKEGSAAQSHRVNRVSSDKSKSRSGSDPKKEPAVPKDGTTAHIAAQT
FTFRELATATKNFKPECLLGEGGFGRVYKGHLESTGQVVAVKQLDRNGLQGNREFLVEVLMLSLLHHPNLVNLIG
YCADGDQRLLVYEFMPLGSLEDHLHDLPPDKEPLDWNTRMKIAAGAAKGLEYLHDKANPPVIYRDLKSSNILLDE
GYHPKLSDFGLAKLGPVGDKTHVSTRVMGTYGYCAPEYAMTGQLTLKSDVYSFGVVFLELITGRKAIDNTRAPGE
HNLVAWARPLFKDRRKFPKMADPLLQGRYPMRGLYQALAVAAMCLQEQAATRPLIGDVVTALTYLASQTYDP
NAATTQSNRVGPSTPRNKDDRRNMVDGLDSPGGRHGSPSTHRNSPDFRKRDSARELSAGAELGRSESSGGSG
RKWGVDDLEQSRISERQPFKHRKSKRKESRSG

>A213|A0A4S4E5G8

MGCFPCFDSKEEKQLNPDKQRDHRNEIHPTLPSNISKLSSGGDRLKSRNNVGMRKESLGGPKEAPDVNIAAQTF
TFRELAAATKNFTPECFLGEGGFGCVYKGRLESTGQAVAVKQLDKNGLQGNREFLVEVLMLSLLHHPNLVSLIGY
CADGEQRLLVYEFMPFGSLEDHLHDLPPDKEPLDWNTRMTIAAGAAKGLEYLHDKANPPVIYRDFKSSNILLDER
YHPKLSDFGLAKLGPTGDKSHVSTRVMGTYGYCAPEYAMTGQLTVKSDVYSFGVVFLELITGRKAIDSTLPHGAQ
NLVSWARPLFNDRRKFAKLADPRLKGQYPMRALYQALAVASMCIQEQAAARPLIRDVVTAISYLANQAYDPNA
TAPPGHSYRFPGDKDEKRTKDERGGRILRNDKGCGSGLKWDFEGFEKEDSPKETARMLNNRDLDRERAVAEAK
MWGENWLVKRRENAQGGFDGTDGQCSNV

>A214|M0TSJ6

MVCFPCFRSPGQGKEKEVKKKIEGIGGADCKQEFSAAPSSHHGSSVSKTGASTPKEGNAGNIAAQTFTFRELATA
TKNFRRDCLLGEGGFGRVYKGRLETGQVVAVKQLDRNGLQGNREFLVEVLMLSLLHHPNLVNLIGYCADGDQR
LLVYEFMPLGSLEDHLHDIPADVEPLDWNTRMKIAAGAAKGLEYLHDTANPPVIYRDFKSSNILLGKGYHPKLSDF
GLAKLGPVGDKTHVSTRVMGTYGYCAPEYAMTGQLTVKSDVYSFGVVLLELITGRKAIDNSRPVGEQYLVAWA
RPLFKDRRKFAKMADPLLQGHYPTRGIYQALAVAAMCLQEQAATRPLIGDVVTAVSYLASQNYDPIAASTRSTR
NGPSTPRSTSRIDNQHADAKIGIGSSGGDSPKDICHNLDRERAVAEAKEWGETWRERQRKNTPGSFHTVCIRHL
IYVSICLMGLCVADVVDVIVGFFCSL

>A215|ALR83540.1

INEIDLSHNELTGMIPERFDNCSTLEIFNVSYNRLTGPIPSYGPIFRNMSISALAGNQGLCGGVLSPCFDFPPPSEYT
PQNKKPGPLVWVMGAVFALALFILIVGTRCFYKQYKWYISGNKYRTEHDEGPWKMTAFQRLNFTVEAILECLKS
SNIIGMGAAGTVYKAEMPSGEVIAVKKLWRNQKEASRRRRGVLAEVDVLGNVRHRNIVRLLGCCSNNENTLLLY
EYMPNGSLADLLHGNKEANMLADWMTRYKIAVGVAQGLMYLHHDCYPVVVHRDVKPSNILLDADMEARVA
DFGVAKLIQTDESMSVIAGSYGYIAPEYAYTLQVDEKSDIYSFGVVLLELLTGRRSVDSEFGDAINIVDWVRGKIQT
KEGILQVLDQNVGASCSSVQEEMILVLRVALLCTTRCPADRPSMRDVVTMLSEAKPRRKTILNLNCLQQLQQQT
PNLRSNPSSQGSSPATSQVLV

>A216|LOC109778264

MSCFPCSGSSGKAGEDAAALSPSPRPSAAKPAPDRSNSRSSSSVRRGGSIPHGPAKIFTFRELAIATKNFRKDCLL
GEGGFGRVYKGHMENGQQVIAVKQLDRNGFQGNREFLVEVLMLSLLHHPNLVRLIGYCADGDQRLLVYEYML
LGSLENHLHDRPPDREPLDWNARMRIAVGAAKGLEYLHDKANPPVIYRDFKPSNILLSEDYYPKLSDFGLAKLGP
VGENTHVSTRVMGTYGYCAPEYAMTGQLTVKSDVYSFGVVFLELVTGRRAIDHTKPDGESNLVAWARPMFRD
RRKFCQMADPLLQGRYPKRGLYQALAVAAMCLQEKAASRPLIGDIVTALSYLASQHYDPKSCRTCPSTPRAKAH
RRTTSCVPDAQRAADTLNWDAVDLRRKDSRGGEFEEDLSEGCCSGSSSGRNDGLDVPVLLALHNGKSYGEADR
DRKSAAKVDAHEKPRADPGKYSRQV

>A217|LOC110092571

MGCFPCFGSEEVVTFHPRDEGDVKKEERPMVAPRIKTLSSGSDRFRSRSNLGAKKEALSLKDGSGVTISAQTFTF
RELAAATKNFRPACFLGEGGFGRVYKGHLESTGQIVAVKQLDKDGLQGNREFLVEVLMLSLLHHPNLVNLIGYCA
DGDQRLLVYEFMPLGSLEDHLHDLQPDKEPLDWNTRMKIAAGAAKGLEYLHDKANPPVIYRDFKSSNILLDEGF
HPKLSDFGLAKLGPVGDKSHVSTRVMGTYGYCAPEYAMTGQLTVKSDVYSFGVVLLELITGRKSIDSTRAHGEQ
NLIPWVIHVIGNFNKLMQPKARPIFNDPKNLSKLADPRLQGRYPMRGLYQAVAVAFMCIQEEAAARPLIADVVT
ALSFLVNKPYDPHTSSHHSEERQSQGPSDEKMERENHENGCKWDGYVLEKDDSPSQIASILKKEFDRDRAVSEA
MMWGANWREKQRATNQANFD

>A218|LOC110731365

MIFLGCCRAEILDEEGRSSSSSSLVSTPRKSNRWRRRRSGKRAIMNNNGNYHAKMSIATVVKTFSFRTTTKGKKK
QQTVEYSDRSISGQKISAAQTFTFRQMANTTNNFSIDNLVGEGGFGRVYKGYLQGVDKVVAVKQLDRNGLQG
NREFLSEVFMLSLVDHPNLVNLIGYCADGDQRILVYEYMAFGSLEDHLLDLPPNTEPLSWQTRMKIAEGAAKGLE
YLHETSKPPVIYRDFKASNILLDEEFNPKLSDFGLARIGPTGDKEHVSTRVMGTYGYCAPEYAMTGKLTTKSDVYS
FGVVFLELISGRRAVDTTKPTAEQNLISWAQPLFKDRKMFTKMADPLLKDKYPVKSLYQALAVAAMCLQEEAST
RPLISDVVTALAFLSKPEDDYESPRNSVIIHAVEEEDMENDHEEDYHKDDENEDEDEDDDKKVHQHGYVVDIDE
SSPNRHFETSHPLPMPDSNA

>A219|LOC109756959

MGFLSCLFRCPEEEERVVKEHDDDEDSGGIDHGVASESSESVPLKTESTHMEGIQRNGTHNEAMIFTLRELVDAT
KNFSKDFQLGRGGFGCVYKAYLNDGQVVAVKQLDLNGLQGNREFLVEVLMLNLLHHPNLVNLIGYCVDGDQR
LLVYEYMPLGSLEDHLHDLPPNKEPLDWTTRMKIAAGAAAGLEYLHDKANPPVIYRDIKPSNILLSEGYHAKLSDF
GLAKLGPVGDKTHVTTRVMGTYGYCAPEYAATGQLTVKSDIYSFGVVFLELITGRRALDSNRPREEQDLVSWAR
PLFKDQRKFPKMADPLLRGRFPKRGLYQALAIAAMCLQEKSRNRPLIREVAAALSYLSSQTYDGNDAAVCRYLDG
PSASKVSDDQVNQDDALASQHGAQTSMHDRMNDLVPEGKEHCRSGSNRGVRGRVVPNGVDRDRALADAN
VWAEAWRRHEKASKVRVTDEILG

>A220|LOC109830615

MGCFHSREEEQLNNPDNNDSNNNNNNGKVVDRREGQPMAPPRIHRLPSGLDRTRSRGNLSGRRESVSPRDG
SGVKISSQTFTFRELAAATKNFRPECFIGEGGFGRVYKGRLENTGQVVAIKQLDRNGLQGNREFLVEVLMLSLLH
HPNLVNLIGYCADGDQRLLVYEFMPFGCLEDHLLDLPDDKEPLDWNTRMKIAAGAAKGLEYLHDKANPPVIYRD
FKSSNILLDEGFHPKLSDFGLAKLGPVGDKSHVSTRVMGTYGYCAPEYAMTGQLTVKSDVYSFGVVLLELITGRR
AIDSTKAHGEQNLISWARPMFNDRRKLAKLADPKLQGRYPMRGLYQALAVASMCIQEDATTRPLIADVVTALSY

LASQAYDPSMGPASIHRSRSGRSATEKGEDGGGSSGRKLEVDGLEKDESPREVTSVNKDFDRERAIAEAKMWG
ANWREKRRASENGSFDASSGNG

>A221|LOC105034278

MGCFSCFDSKEAAQLNPGNGGDDRREEQPMVPPRVLKLSSGADRIKNRSNLDLKRDSMGPKEEPGSNISAHTF
TFRELAAATKNFRPECFLGEGGFGRVYKGHLESTGQVVAVKQLDRNGLQGNREFLVEVLMLSLLHHPNLVNLIG
YCADGDQRLLVYEYMPLGSLEDHLHDLPPGKEPLDWNTRMKIAAGAAKGLEYLHDKANPPVIYRDFKSSNILLD
EGFHPKLSDFGLAKLGPVGDKSHVSTRVMGTYGYCAPEYAMTGQLTVKSDVYSFGVVLLELITGRKAIDSTRAHG
EQNLVSWARPMFNDRRKLPKVADPKLQGRYPTRGLYQALAVASMCIQEEAASRPLIADVVTALAYLASQAYDSS
AGPNSNNRSAGERRCGDEKGRRMPAKNEGGFGHRWELDVEKEDNPREMVGILNRDFDRERAVAEAKMWGE
NWREKKQADANLQDSNDTVSGNG

>A222|LOC105048968

MGCFPCFDSSDEGVFDTGSEREDKREEQPMVPPRVDLVSSVADSMRSKSNSRSERETQYQKDGSGVTISAQTF
TFRELAAATGNFRPKCLIGEGGFGRVYKGYLESTGKVVAVKQLDKNGLQGNREFLVEVLMLSLLHHPNLVNLIGY
CADGDQRLLVYEFMPLGSLEDHLLDLPPDKEPLDWNTRMRIATGAAKGLEYLHHKANPPVIYRDFKASNILLDEG
FDPKLSDFGLAKLGPVGDKSHVSTRVMGTYGYCAPEYAMTGQLTVKSDVYSFGVVLLELITGRRAVDNSRPQGE
QNLVSWVRPILNDRRKLPKLADPKLQGRFPMRGLYQALAVASMCVQDQAASRPLIADVVTALSYLASQAYDPG
ALHAFSNRSGRDHDERSHQGGVRVLANNEEGGGSGRKFVEEREESPRVVVGALNKDFDRERAVAEAKLWGEN
WREKIRANANQRDASNANG

>A223|A0A443N7L4

MGCFSCFDSKEEQQLNPGKKKDYASEGKPMAESHFAKLSSGADRLKARNTAVSRKESSGPKGEAAADIKAQTFT
FRELAAATNNFRPECLLGEGGFGPVYKGRLESTGQVVAVKRLDKDGLQGNREFLVEVLMLSLLHHPNLVNLIGYC
ASGDQRLLVYEYMALGSLEDHLLDLPPHKKPLDWNTRMKIAAGAAKGLEYLHNEANPPVIYRDFKSSNILLDEEY
NPKLSDFGLAKLGPTGENTHVSTRVMGTYGYCAPEYAMTGQLTVKSDVYSFGVVFLELITGRRAFDSSRVHGQQ
SLVAWARPMFNNRKKLPDLVDPRLEGRYPMRGLYQALAVASMCIQEEAAARPQIADVMTALSYLTSHAFDPN
ASSAQSIREKEERRTSNSNGSSNRPAERGGSIGRNGEGGSGSKLDLDGSGREDSPKETVRMLNRDLDRERAVAE
AKLWGENWREKRQANG

>A224|A0A5D3ADM4

MGGGGCFPCFGSSNKEKSNNGGKSIKELNNKDSIKDGSVGQSHHVNRASLDKSKSRSGSDSKKEPAVSKNGSTE
NIPAQTFTFRELAAATKNFRPECLLGEGGFGRVYKGRLESTGQVVAVKQLDRNGLQGNREFLVEVLMLSLLHHP
NLVNLIGYCADGDQRLLVYEFMPLGSLEDHLHDLPPDKEPLDWNTRMKIAAGAAKGLEYLHDEASPPVIYRDLK
SSNILLDEGYHPKLSDFGLAKLGPVGDKTHVSTRVRGTYGYCAPEYAMTGQLTLKSDVYSFGVVFLELITGRKAID
NMRAPGEQNLVAWARPLFKDRRKFPKMADPLLQGRYPMRGLYQALAVAAMCLQEQAATRPLMRDVVTALT
YLASQTYDPNAPGNQSNRVGPSNPRLKDDRNMADGLDSPEGRGWHHGSPSTHRNSPDYRKRNQVRESSTGS
ELIRNDAGGGSGRKCGSDDSE

>A225|J3M6I1

MGCFPCFSSTGKGEAKKEGGAGGGSGGSDRKDDSSDRRVTRVGSDKSNSQGGFDSRKDSIMPRDGNGQHIAA
HTFTFRDLAAATKNFRQDCLLGEGGFGRVYKGHLENGQDVAVKQLDRNGLQGNREFLVEVLMLSLLHHDNLV
NLIGYCADGDQRLLVYEFMPLGSLEDHLHDIPPDKEPLDWNTRMKIVAGVAKGLEFLHDKANPPVIYRDFKSSNI
LLGQGYHPKLSDFGLAKLGPVGDKTHVSTRVMGTYGYCAPEYAMTGQLTVKSDVYSFGVVFLELITGRKAIDNT

KPLGEQNLVAWARPLFKDRKKFPKMADPLLAGRFPMRGLYQALAVAAMCLQEQAATRPFIGDVVIALSYLASQ
TYDPNAPVQHSRSNASTPRARPRGGGNGDQRRLHSPDSGCGSGRRLGLDNMDTNSSQMGSPAYTGKKRESS
RGTVRPRAIAEAKTSGENSRERK

>A226|LOC109710403

MGCLPCFESRRKRDELGDDGDRREEKRKEEQPMVPPSLDRVSSDLKNRSSSESKKGIDAPNDSSSVNISAQTFTF
RELAAATRNFRSECFLGEGGFGRVYKGRLESTGQIVAVKQLDQNGLQGNREFLVEVLMLSLLHHPNLVNLIGYCA
DGDQRLLVYEFMPFGSLEDHLHDLPPEKEGLDWNTRMKIAAGAAKGLEYLHNKANPPVIYRDFKSSNILLDKDF
HPKLSDFGLAKLGPTGDKSHVSTRVMGTYGYCAPEYAMTGQLTVKSDVYSFGVVFLELITGRRAIDSTRPHGEQ
NLVSWARPLFNDRKKLPKLADPRLQGRFPMRGLYQALAVASMCIQEQAASRPNIADVVTALSYLASSYDPNASS
PTSSNKSGGGEHDDRRSRGTGGRLVQKNEEGGGGLEREDSPREGVGSVMNKDFDRERAVAEAKLWGENWRE
KIRANAVAREAANNSS

>A227|A0A2R6QRF8

MGCFSCFDSRDVENLNLDKQRDHRKEIHPPVPSNISRLSSGEDRLKSRSNVGLRKESPNVNIAAQTFTFRELAAAT
KNFRPESFIGEGGFGRVYKGRLESTDQVIAVKQLDRNGLQGNREFLVEVLMLSLLHHPNLVNLIGYCADGDQRLL
VYEFMPLGSLEDHLHDLPLDKEPLDWSTRMKIAAGAAKGLEYLHDKASPPVIYRDFKSSNILLHEGFHPKLSDFGL
AKLGPTGDKSHVSTRVMGTYGYCAPEYAMTGQLTVKSDVYSFGVVFLELITGRKAIDSTRPQGEKNLVAWARPL
INDRRKFAKLADPGLEGQFPIRGLYQALAVASMCTQEQAAVRPLIGDVVTALSYLANQSYDPNSAPGHCSRFNG
DKDERRTRDERGGRILKNEEGGGSGRKWDLEGSERDDSPRETAKMLNRDLDRERAVAEAKMWGENWREKRR
QSAQGSFDGTTG

>A228|A0A2R6REL7

MGCFPCFESREDEKLNSNKQIDHRKQSCPTLPSNMSRLSSGADRLKSRSNVGSRKEPPSQKDAPDANIAAQTFT
FRELAAATKNFRPESFLGEGGFGSVYKGRLESTGQAVAVKQLDRNGLQGNREFLVEVLMLSLLHHSNLVSLIGYC
ADGDQRLLVYEFMALGSLEDHLHDLPPGKGPLDWNTRMKIAAGAAKGLEYLHDKANPPVIYRDFKSSNILLDEG
YQPKLSDFGLAKLGPTGDKSHVSTRVMGTYGYCAPEYAMTGQLTVKSDVYSFGVVFLELITGRKAVDSTRPSGE
HNLVAWARPLFNDRRKFVKLADPRLQGQYPMRGLYQALAVASMCIQEQAAARPLIGDVVTALSYLVNQTYDP
NTKSSGDKDDKRNKDERIGRILKNEEGGGSGRKWDFEGSERDDSPKETARMLNRDLDRERAVAEAKMWGEN
WREKRRQNAHGSFDGTNG

>A229|ADE76472.1

MGCFPCFRSSKKKKKPSKPSKPLKPSKSLKPSKDDSKTGQSAASSTHQPSKDPPDSLKSKPSFDSRKEVSRDGSQH
IAAQTFTFRELAAATKNFRPECLLGEGGFGRVYRGRLESTGQAVAVKQLDRNGVQGNREFLVEVLMLSLLHHDN
LVNLIGYCADGDQRLLVYEYMPLGSLEDHLHDLPPDKEPLDWKTRMKIAAGAAKGLEYLHDKANPPVIYRDLKC
SNILLDEGYHSKLSDFGLAKLGPVGDKTHVSTRVMGTYGYCAPEYAMTGQLTIKSDVYSFGVVLLELITGRKAIDN
SRSAGENNLVAWARPLFKDRRKFSQMADPLLQCRYPMRGLYQALAVAAMCVQEQATMRPLIADVVTALTYLA
SQTYDPETHPVINSRFGPPTPSRAKRDNEKKLTSSARTNRHLGVHSPSIHAHGSPDQNQAAAETKIWIERGEKKR
VNTRGGMNEFG

>A230|LOC100830409

MGFLSCLFRCPEDGLAAAEEEECSRVAHQDASVEFSESSESCPLKTESMDMEGIQRNGAHDEALIFTMRELADA
TNNFSPDFLLGRGGFGCVYKAYMNGGQVVAVKQLDLNGLQGNREFLVEVLMLNLLHHPNLVNLLGYCVHGD
QRLLVYEYMPLGSLEDHLHDLSPNQQPLDWTTRMKIAAGAAAGLEYLHDTANPPVIYRDIKPSNILLGEGYHAKL

SDFGLAKLGPVGDKTHVTTRVMGTYGYCAPEYASTGQLTIKSDIYSFGVVFLELITGRRALDSNRPREEQDLVSW
ARPLFKEQKKFPKMADPLLQGHFPRRGLYQAMAIAAMCLQEKARNRPLIREVAAALSYLSSQTYDRHDAASRRH
LVGPSAPRAQDDLVNQDLAMPSQHGAETSMHDQSNHFVPEGKEHCWSGSNRTSRGRVVPNGVDRERALAD
ANVWAEAWRRHEKENKIR

>A231|LOC110027867

MGCFPCFGSEVVDFHARDEGDAKKEEKPMVAPRVRRLSSGSDRFRTRSNLGSKRETLTVKDGSGVAISAQTFTF
RELAAATKNFKPECFLGEGGFGRVYKGYLESTGQTVAVKQLDKDGLQGNREFLVEVLMLSLLHHPNLVSLIGYCA
DGDQRLLVYEFMPLGSLEDHLHDLPPDKEPLDWNTRMKVAAGAAKGLEYLHDKANPPVIYRDFKSSNILLDEGF
HPKLSDFGLAKLGPVGDQSHVSTRVMGTYGYCAPEYAMTGQLTVKSDVYSFGVVFLELITGRKSIDSSRAHGEQ
NLIPWARPMFSDRRKLSKLADPRLQGRYPMRGLYQALAVASMCIQEEAAARPLIADVVTALSYLASQTSSHHPE
DQRSRGSRDRESQEDGGKWDAYALEKGDSPREIANIMNKEVDRDRAVSEAMMWGANWREKQRAATSSGA
AAAAANQVTFDTLNVNE

>A232|A0A4S8IEH3

MGCFPCFESEEEAQLSHGNECDRTREERPMVAPRVEKLSSGDDRMKTARDLSTKRESLGAIEGSDFSISAQTFTF
SELLAATSNFRTESLLGEGGFGRVYRGRLETTGQVVAVKQLDRNGVQGNREFLVEVLMLSLLHHPNLVNLIGYCA
DGDQRLLVYEYMPLGSLEDHLHDFPPDKEPLDWNTRMKIAAGAAKGLEYLHDKANPPVIYRDLKSSNILLDKGF
HPKLSDFGLAKLGPVGDKSHVSTRVMGTYGYCAPEYAMTGQLTVKSDVYSFGVVLLELITGRKAVESTKSHSEQ
NLVSWASPMFKDRRKMASLADPRLQGRYPMRGLYQALAVASMCIQEEAASRPAIADVVTALSYLASQAYDPG
ASPTNNNRPGGERRNKNADEGSGRKPSNNNGESDHNSQMNCENTLKEKAATLRRDFDRERALAEAKMWGR
NWREKTQAKDNAGGN

>A233|LOC108844003

MGCFSCFDSSEGEKLNRVEESSKAQKHSQPTVSNNISKLPSGGEKQLTSKSKVGLKTELLLPSDGQQISAHTFAFR
DLVAATMNFHPDTFLGEGGFGSVYKGRLETTGQVVAVKQLDRDGLQGNREFLVEVLMLSLLHHPNLVNLIGYC
ADGDQRLLVYEFMPLGSLEDHLHDLPPDKEGLDWNTRMKIAAGAARGLEFLHDKANPPVIYRDFKSSNILLGEG
FHPKLSDFGLAKLGPTGDKSHVSTRVMGTYGYCAPEYAMTGQLTVKSDVYSFGVVFLELITGRKAIDTELPHGEQ
KLVAWARPLFNDRRKFMKLADPKLKGRFPTRALYQALSVASMCIQEEAATRPLIGDVVTALSYLANQAYDPNKN
ERGGGGRLITTRNDEGGGSGSGSGRKFDVEGSEKDDSPRETARMLSRDIDRERAVAEAKMWGESLREKRRQSE
QGTSESSGTG

>A234|LOC4326237

MGFLACLFPCPQEVRDEDEEPRSGQRVSSVEYSKSSESCPLKTEGSIDMVGIRRNKGHGEATIFTLRELADATNNF
STECLLGRGGFGSVYKAFLNDRQVVAVKQLDLNGLQGNREFLVEVLMLSLLHHPNLVKLFGYCVDGDQRLLIYEY
MPLGSLEDRLHDLRPGQEPLDWTTRMKIAADAAAGLEYLHDEAIPAVIYRDIKPSNILLGEGYNAKLSDFGLAKLG
PVGDKTHVTTRVMGTHGYCAPEYLSTGKLTIKSDIYSFGVVFLELITGRRALDSNRPPDEQDLVAWARPLFKDQR
KFPKMADPSLHGHFPKRGLFQALAIAAMCLQEKAKNRPSIREVAVALSYLASQTHESQNTAARHTLPGPSVPRV
LDNQINQDTSLPSQHGVHMPPLAGTDHMVQEVKENCRSSSHRPGRGRVTPNGADRERALAEANVWVEAWR
RQEKTSKMR

>A235|A0A5A7PVX7

MGCFPCFDSREEEKLNSYQESNDHKEVHPTVPPNVAKLSSGGDRLKTSNVGPNKEAPLSKDLPDAQIAAQTFTF
RELANATNNFRPECFLGEGGFGRVYKGRLPSGQAVAVKQLDRNGLQGNREFLVEVLMLSLLHHPNLVSLMGYC

ADGDQRLLVYEFMPLGSLEDHLHDLPIEKEALDWNTRMKIAAGAARGLEYLHDKAKPPVIYRDFKSSNILLGEGF
SPKLSDFGLAKLGPTGDKSHVSTRVMGTYGYCAPEYAMTGQLTVKSDVYSFGVVFLELITGRKAIDSTLPQGEQN
LVAWARPLFNDRRKFAKLADPRLQGKFPMRGLYQALAVASMCTQEQAAARPLIGDVVTALSYLANQAYDPTQ
GNCKDRDEKGGKLLRNEESGGSGRKWDLEGSERDDSPRENPTKSTKDLERERAVAEAKMWGENWREKRRQV
QDNALGTFDGHNG

>A236|A0A328CZG9

MGCFPCFDSKEAETLNKGSDDHIEVHPTVPSNMSRLSSGADRLKSRSSASLKREPSVLKDLPGGQIAAHTFTFREL
AAATSNFRPESFIGEGGFGRVYKGKLANGQAVAVKQLDRNGLQGNREFLVEVLMLSLLHHPNLVNLIGYCADG
DQRLLVYEFMPLGSLEDHLHDLPPDKEPLDWNKRMKIAAGAAKGLEHLHDKANPPVIYRDFKSSNILLDEGFTPK
LSDFGLAKLGPTGDKSHVSTRVMGTYGYCAPEYAMTGQLTVKSDVYSFGVVFLELITGRKAIDSTQPPGQQNLV
TWARPLFNDRRKFAKLADPKLQGQFPMRGLYQALAVASMCIQEQAAARPLIGDVVTALSYLANQAYDPCTANK
SSGDDKRALRLSKNEDGSGCGSGRKWDLDGSEKEDSPRETVRKLNRDIERERAVAEAKMWGEKFREKRQQGS
DGSCDGSNG

>A237|A0A4S4E5M2

MGCFSCFDSREDEKPNLHNERDHPNESLPTAPSNISRLSSGGDRLKSRSNLGSRKESSSPKGLPDVNIAAHIFSFRE
LATATKNFRPESFLGEGGFGRVYKGRLESTGQAIAVKQLDRNGLQGNREFLVEVLMLSLLHHTNLVNLIGYCADG
DQRLLVYEFMPLGSLEDHLHDLPLDKEPLDWNTRMTIAAGAAKGLEYLHDKANPPVIYRDFKSSNILLDEQHHPK
LSDFGLAKLGPTGDKSHVSTRVMGTYGYCAPEYAMTGQLTVKSDVYSFGVVFLELITGRKAIDSTRPPGEQNLVA
WARPLFNDRRKFAKLADPRLQGKYPMRGLYQALAVASMCIQEQAAARPLIGDVVTALSYLANQAYDHKDERRT
KEERGGRILKHEEGGGSGCKWDFEGSERDESPRETAKMLNRDLDRERAVAEAKMWGENWREKRRQNAQGSF
DGTNR

>A238|OEL37153.1

LMLNGSLTVGYSKSSESCPLNTGSIVHVEGTQLISRHDEAIIFPLYQLADATKNFSQDCLLGRGGFGCVYKATLSDG
KVVAVKQLDLNGLQGNREFLVEVLMLNLLHHPNLVNLFGYCIDGDQRLLVYEYMPLGSLEDHLHDLAPDQEPLD
WKTRMKIAADAAAGLEYLHDKAHPPVIYRDIKPSNILLGEGYHAKLSDFGLAKLGPVDDKTHVTTRVMGTYGYC
APEYALTGQLTIKSDIYSFGVVFLEIITGRRPHDSYRPPEEQDLVAWARPLFKDQRKFPKMADPLLHGRFPRRGLY
QALAIAAMCLQEKAKNRPPIREVAAALSYLASQTHDMNNTAARRNRAGPSTSGVLVGQMNQDATLPSQQEV
QMSIHGRTNHAAPEVKETSWSGAHRAGRGRVVPNGIDRERALADANVVAVKQLDLNGLQGNKEFLMEVLML
NLLRVTLI

>A239|A0A2I0ASL5

MLNFNPCENLKDRSAICLPVATSACIFMRFHYTTGDKSKSWSASESKKDALVPQEGSASHIAAQTFTFRELAAAT
KNFRPECLLGEGGFGRVYKSRLENGQVVAVKQLDRNGLQGNREFLVEVLMLSLLHHPNLVKLIGYCADGDQRLL
VYEYMPLGSLKDHLHDLLPNKEPLDWTTRMKIAAGAAKGLEYLHDRANPPVIYRDFKSSNILLGEGYHPKLSDFG
LAKLGPVGDKTHVSTRVMGTYGYCAPEYAMTGQLTVKSDVYSFGVVFLEVITGRKAIDNSRSTGEQNLVAWAR
PMFKDRRKFPKMADPLLQGRYPMRGLYQALAVAAMCLQEQAATRPLIGDVVTALSYLASQTFDPNSPANQSC
LVSPTLKAREERKSVGGGFESNLATGLPRKDSPKFRQKDSVKGINFGTDFGIVVEDSGGGSEAKWGLEEFEQREC
AIVGG

>A240|LOC105958008

MGCFSCFDSREEEKLNPRKERDDHKEIHPSIPSNISKLSSGGDRLKSRNNVGPKKEISLPKDLPDSQIAAQTFTFREL
ANATNNFRPDCFLGEGGFGRVYKGRLSTGQAVAVKQLDRNGLQGNREFLVEVLMLSLLHHPNLVSLMGYCAD
GDQRLLVYEFMPLGSLEDHLHDLPPDKEALDWSTRMKIAAGAARGLEYLHDKANPPVIYRDFKSSNILLGEEFAP
KLSDFGLAKLGPTGDKSHVSTRVMGTYGYCAPEYAMTGQLTVKSDVYSFGVVFLELITGRKAIDSTRPQGEQNLV
AWARPLFNDRRKFAKLADPILQGKFPMRGLYQALAVASMCIQEQAAARPLIGDVVTALSYLANQSYDPTNGGR
GEERGGKILRNEEGGGSGRKWEFEGSEKDDSPKETAKMLNRDLERERAVAEAKMWGENWREKRRQNAQGSF
DANNG

>A241|LOC109770260

MGILCCFQSGADKLLDHGHGGGGGGGPAAVPASTTKKPPPRDAPTVTVRPPNLLRRDDDHQEEGGARSSSNN
NNLATLVDEIVAESATHQHNRRVADEILGMNKEEAAVTERAFTFAELSEASGGFRVESMLGEGGFGPVYRGRLR
DGTEVAVKQLDRNGLQGTREFLVEVLMLSLLKHPHLVTLIGYCADASHRMLVYEFMPQGSLEDHLLDLPPSSPGL
DWAMRMRIAQGAARGLEYLHDASRRPGPPVIYRDFKASNILLDGCFRAHLSDFGLAKVGPVGDKTHVSTRVM
GTYGYCAPEYALTGKLTTMSDVYSFGVVFLEIITGRRVIDTSRPRDEHNLVQWAAPRFKSKKRFREMADPLLRGA
YPTKGLYQALAISAMCLQEDATMRPSIADVVMALDYLTGVAKPSPTPTPQQQSPSPSPSPTPTPQQQSPPKEED
DATD

>A242|LOC110028637

MGCFSCFAFEAERFDSRGEGDAKEEGETMEVPRMRILSSGSSKIRSRSSLGSRRLSLAQKDETGVTISAQTFTLKQ
LSAATKNFRRDCFIGEGGFGRVYKGRLDSSGQTVAIKRLDQDGLQGNREFLVEVLMFSLLHHPNLVNLIGYCAEG
DQRLLVYEFMPLGSLEDHLHDLPPGKEALDWNTRMRIAAGAARGLEYLHDKANPPVIYRDFKSSNILLDEGFHA
KLSDFGLAKLGPVGDKSHVSTRVMGTYGYCAPEYAMTGQLTAKSDVYSFGVVFLELITGRKSIDSTRAHGEQNLI
RWAKPMLTDHRKLSKLADPLLQGRYPMRSLYQAVAVASMCIQEEATTRPLIADVVTALSYLESQTDEPAGVPVY
SQQQSRGSREENLGRGNHEDRSKRNLCGIEKGDVPKEIGSITNKEIDRQQEVAKAMMWGANWREKQRATM

>A243|A0A068UYJ8

MGCFPCFDSREEEKLNPQNDRDGPKEVHPAIPSNFSRLSSGADRLKTRSNVGSRKESSGLKDMPDVQIAAQTFT
FRELAAATNNFRQESFLGEGGFGRVYKGQLLSGQVVAVKQLDRNGLQGNREFLVEVLMLSLLHHSNLVNLIGYC
ADGDQRLLVYEFMPLGSLEDHLHDLPPDKEPLDWNTRMKIAAGAAKGLEYLHDKANPPVIYRDFKSSNILLDEG
FVPKVSDFGLAKLGPTGDKSHVSTRVMGTYGYCAPEYAMTGQLTVKSDVYSFGVVFLELITGRKAIDSTLPHGEQ
NLVTWARPLFNDRRKFARLADPRLQGQYPMRGLYQALAVASMCIQEQAAARPLIGDVVTALSYLANQAYDPSA
APGLGYRFTNDKDEKRNKDERGSEKEDSPRETARMLNRDLDRERAVAEAKMWGENWREKRRQNAQGSFDG
NNG

>A244|LOC104882836

MTFLACCRADFLEEEGRSSSTSTSSAISSPRKSRRRNRRRFIRRTTMKANFTDNKMSMATVVKTLSLRTAARGKK
KQQQMLESVAGHKSSAQTFTFRQMANATNNFSIENLVGEGGFGRVYKGYIPGVEKVVAVKQLDRNGQQGNR
EFLSEVFMLSLVDHPNLVNLIGYCADGDQRILVYEFMANGSVEDHLLDLPPNKEPLDWHTRMKIAEGAAKGLEY
LHETNKPQVIYRDFKASNILLDEEFNSKLSDFGLARIGPTGDKEHVSTRVMGTYGYCAPEYAMTGQLTTKSDVYSF
GVVFLELISGRRAVDTTKPTAEQNLVSWAEPLFKDRKMFTKMADPLMADKYPVKGLYQALAVAAMCLQEEAST
RPLISDVVTALEFLAKPEDDLGSPRSDSIIIRAVEAEDIEGEQEEHHEEEAEDKKEEHQHGYVVQLGTSPDHMI

>A245|LOC110876806

MKVKAWTYNEEVALAKAFIHCFKDPVHGIQLFGKRFYGVYRSKLIISGGLKDIYRGSCNKLVPYPLPLPPQHSSTD
TQNLPTPSPGNGNEMIFATHLKSFTILEVLVATRNFSPDYRLGEGGFGLIYKGWLNKETLIPAQPGPRMDVVVKA
LKPTCFQGHREWLSEITYLGRLDHPNLVKLLGFCSEAGNRLLVCEFMPQGSLENHLFRRREQSLSWALRIKVAVE
AARGLAFLHASESKIIYGDFKSSNILLDMDYNAKLSDFGLAKAGPMGDLTHETTQVMGTQGYTAPEYMATGRLT
KKCDAYSFGIVLLELITGRRAIDNKRCCEERNLLEWVRSQLREAKKLCRIMDIRLEGRYSRKDAFVVATLALQCCHP
EAKHRPHMSEVLSILENIPSIRANRQPELSSSKSKIIGDGPSSWSHGCHYGSQVPMDCKIHP

>A246|OEL36186.1

MGCLPCFGSAGEGAAKKGSARKDGSSDRRVTRVGSAPGIILSLYLKKDMAIALEILSQRAYGVVAVKQLDRNGLQ
GNREFLVEVLMLSLLHHDNLVNLIGYCADGDQRLLVYEFMPLGSLEDHLHDIPPDKEPLDWNTRMKIAAGAAK
GLEYLHDKASPPVIYRDFKSSNILLGEGFHPKLSDFGLAKLGPVGDKTHVSTRVMGTYGYCAPEYAMTGQLTVKS
DVYSFGVVFLELITGRKAIDNTKPHGEQNLVAWARPLFKDRRKFPKMADPLLQGRFPMRGLYQALAVAAMCLQ
EQAATRPFIGDVVTALSYLASQTYDPNAPAQHSRSNSSTPRASRGGGSNDQRRLRSPNHHSPDLRRREATTASK
YEAEVSRTNSGSGSGRRSGLDDLDMTGSQLGSPGHGGRKRESPRTERQLAVASAKTWGENSRERN

>A247|LOC17889262

MGCFGRTAKSNKRSEIKNTTKNDVSPKKLTIDSNIVDKNVNRDNQTQPSSDCLKVSLCGDVNKEVVIKEDQLALD
AKDTLVEDEVSGKKAQTFTFEQLAVSTGNFKSDSFLGEGGFGKVYKGFIEKINQVVAIKQLDRSGSQGIREFVVEV
MTLSLADHPNLVKLIGFCAEGVQRLLVYEYMPLGSLENHLHDLPHCKKPLSWYTRMKIAAGAARGLEYLHDTIKP
PVIYRDLKCSNILLDEGYHAKLSDFGLAKVGPRGAETHVSTRVMGTYGYCAPDYALTGQLTFKSDVYSFGVVLLEL
ITGRKAVDNTRARNDQCLVEWARPLFKDRKNFKKMVDPLMEGEYPIRGLYQALAIAAMCVQEQPSMRPVIAD
VVMALDHLASSKYDHGHRRKQTNVTETRADDEEKILPESNVCVEEKQEEIKICSDQADD

>A248|XP_024388663.1

MCTCWNALDGAWFGSCWWSKSSSTAAASHSMPHTESKKDPLHGQAPSEQVIVSIREVGIKSSEEIRQGGNLRI
FTYQDLKSATRNFRPDSLLGEGGFGSVYKGWIDEHGTTAAKAGTGLTVAVKQLNQEGLQGHREWLAEVNFLG
QLHHPNLVKLIGYCSEDDQRLLVYEFMPRGSLENHLFRKGVMPLPWLTRMKIALGAASGLAFLHEAVKPVIYRDF
KTSNILLDSDYTAKLSDFGLAKDGPEGDKTHVSTRVMGTYGYAAPEYVMTGHLTSRSDVYSFGVVLLEMLTGRR
SVDKNRPSGEQNLVEWARPYLNDKRKFYRLIDPRLDGQYSVKGAQKAAILSHHCLSRDPKSRPLMGDVVDTLKP
LQDMRDMFSSSSNQASTNYRSTGMNGHGGGRPYHNGHGHGQKKYEQLRNIPVRASGVSPLPTY

>A249|M0SD10

MGCFTCFGSPSQREEEEESCFGIRISNFLVYYRGLLVVLDKLTARTGSDSKKEASASKEGNAGHIAAQTFTFRELAA
ATKNFRQDCLLGEGGFGRVYRGRLENGQVVAVKQLDRNGLQGNREFLVEVLMLSLLHHPNLVNLIGYCADGD
QRLLVYEFMRLGSLEDHLHDIPADKEPLDWNTRMKIAAGAAKGLEYLHDKANPPVIYRDFKSSNILLSEGYHPKLS
DFGLAKLGPVGDKTHVSTRVMGTYGYCAPEYAMTGQLTVKSDVYSFGVVFLELITGRKAIDNTRPAGEQNLVA
WARPLFKERRKFPKMADPLLQGHYPMRGLYQALAVAAMCLQEQAATRPLIGDVVTALSYLASQTYDQNAATA
QNTRVVKGVSKGAKHGRVHVGRARDSPKNIDRDVVREHAVAEAKVWGENSRERDSRKMH

>A250|LOC100839243

MGSGRQPAGKDKMLSWLRCFPHDGTAAMDSDDDHHPHIRQGPPPPAARPAAARTITSSFSFRKKPAAAVDP
DSAAERRKRFTRTNTTLPDRRRHSSVSASASDGGASDDYNHSVVSARSFSFAELAAATGGFSDDNLLGSGGFGR
VYRGRLAATGAGGEGTAVAVKRLDRTGHQGDREFLVEVLFLSSLLLRHPNLVGLLGYCADGSHRLLVYRLMPLGS
LHDHLFVPSSGAALPWRVRIQIARGAARGLEFLHEKASPAVIYRDLKPSNILLDSGFRARLSDFGLAKLAGNNGDG

DDGDRRMGTHGYCAPEYVRSGRLTVKSDVYSFGVVLLELITGRHAVDEESSLVGWAAPLLAGERHDELLDPRLQ
QGEAVNGRELKQAVAVAAMCLQEEDALRPNMSDVVMALSFLTGAGEEDDDEQQVASRR

>A251|LOC18028063

MGCFGRTGKSNKRSETKNNDFTKNNKLAIDGNCIRKNVDQTQPSSDCLKFSQCGDVNDDIAAKEDQLALDAKD
LNVEDEVSGKKAQTFTFEELSVSTGNFRSDCFLGEGGFGKVYKGFIDKINQVVAIKQLDRNGAQGIREFVVEVMT
LSLADHPNLVKLIGFCAEGVQRLLVYEYMPLGSLENHLHDLPCGKKPLDWNARMKIAAGAARGLEYLHDIMKPP
VIYRDIKCSNILLGEDYHPKLSDFGLAKVGPSGDETHVSTRVMGTYGYCAPEYAMTGQLTFKSDIYSFGVVLLELIT
GRKAIDNTKARKDQNLVGWAAPLFKDRKNFKKMVDPLLEGDYPVRGLYQALAIAAMCVQEQPSMRPVISDVV
MALDHLASSNYDPNHRRKQCNETETKVDGEEKRVNETKGCVEEEKQEIKMCSDEAS

>A252|LOC8062581

MGILCCFQSHTGDGTAHHDHFVASSPATSSSSVPSSYRRAPPERQSAGVEKSSRRNDSIDNSNLVALVNDIVTES
VSYHHRRVAEEILKMGKAGKVTARAFTYAELCEATGGFRPESLLGEGGFGPVYRGRLGSSSGPEVAVKQLDRNG
MQGTREFLVEALMLSLLKHPNLVTLLGFCTDADHRMLIYEYMPLGSLEDHLLDLPPGRAPLDWATRMGVAQDA
ARGLEYLHDAAQPPVIYRDFKASNILLDTGFRARLSDFGLAKVGPVGDKTHVSTRVMGTYGYCAPEYALTGKLTT
MSDVYSFGVVFLEIITGSRAIDTTRPPDKHNLVLWAGPRFKDKRRFAEMADPLLQGAYPTKGLYQALAIAAMCL
QEDATMRPVISDVVTALEYLTVAGGGAADEEPAPDPKQQQQQQQIDVIGHADDQA

>A253|M0RFL4

MKCLPCLASPGQGDAADEVKEEEKKKKKNDEGGDGAVRKEADSKKEPSVPKEGNPEHIAAQTFSFRELSAVTKN
FRRDCFLGEGGFGRVYKGRLETGQVVAVKQLDRNGLQGNREFLVEVLMLSLLHHPNLVNLIGYCADGDQRLLV
YEFMPLGSLEDHLHYIPANKEPLDWNSRMKIAAGAAKGLEYLHDKANPPVIYRDFKSSNILLGEGYHPKLSDFGL
AKLGPVGDNTHVSTRVMGTYGYCAPEYAMTGQLTVKSDVYSFGVVFLELITGRKAIDNARPTGEQNLVAWARP
LFKDRRKFPKMADPLLQGRYPMRGLYQALAVAAMCLQEQAATRPLIGDVVTALSYLASQNHDPIAASIQSTKVA
SKGAKIGRDGSSGGSGRKWSMEESETRESQMDSPVQVERARDSPKNIDRDFVRERAL

>A254|100191239

MLSWLRRFPNDVIHRKGGSSGRRTSSSTSWRNKSSSSFTARIIRCASSVVDTHHQHRGDDDDDDGDEDDEYDD
CRLPSSPPNNHARRNTTTTAVISARAFSFRELADAAGNFRQDNLIGEGGFGRVYKARLPTRVQHAADDADLQGL
PVAIKQLDRNGFQGNNEFMVEVLMLSMLHHPNLVSLVGYCAEGEQRLLVYEYMALGSLEDHLLLLRDDGHGSP
LPWRTRMKIALGAARGLEYLHESAVIYRDLKSSNILLDQDYAPKLSDFGLAKLLPAPRTTDSSSSSSSSSSGGGKV
MGTYGYCAPEYLRTGKLSVKSDVYSFGVLLLELITGRRAIDASRPDGEQSLVGWAAGMFGDSTRFHELLDPRLV
MAIRGRPTASQLKQAVGVASMCLQEHHALRPVMADVVVALSFIANDSPASPPC

>A255|LOC100832072

MGIWCCFQKKQHQDCGGGGAPAVAPAEPPPCHAPATVSPTKRQQPREQDGGGLATLVNEMVAESVSYRHN
KGVADEFLGMNKEAVTARAFTYGELREATGGFRPESMLGEGGFGPVYRGRLRIPHGGGETKPVTGEVAVKQLD
RNGLQGTREFLVEVLMLSLLKHPHLVTLIGYCTDADHRMLVYEYMPHGCLEDHLLDLPPSSPGLDWKTRMRIAQ
GAARGLEYLHCAADRPGPPVIYRDFKASNILLDGAFQARLSDFGLAKVGPVGDKTHVSTRVMGTYGYCAPEYAL
TGKLTTMSDVYSFGVVFLEIITGRRVIDCARPRDEQNLVQWAGPRFKNKRRFREMADPLLRDAYPTKGLYQALAI
AAMCLQEDATMRPAISDVVTALEYLTGASQAPAPPPPPAGEDDGAAAGAGAAEVTSGN

>A256|LOC104818845

MSGCLPCCFGSSAKEGAAKESVKKEASAKDVSLAHSHHVSSEKSKSKGGSEHKKEAAAGKDGATAHIAAQTFTF
RELAAATNNFRPECLLGEGGFGRVYKGRLESPGRIVAVKQLDRNGLQGNREFLVEVLMLSLLHHPNLVSLIGYCA
DGDQRLLVYEFMPLGSLEDHLHDLPPDREPLDWNTRMKIAAGAAKGLEYLHDKANPPVIYRDLKSSNILLGEGY
HPKLSDFGLAKLGPVGDKTHVSTRVMGTYGYCAPEYAMTGQLTLKSDVYSFGVVFLELITGRKAIDNARDPGEH
NLVAWARPLFKDRRKFPKMADPLLQGRYPMRGLYQALAVAAMCLQEQAATRPLIGDVVTALTYLASQSYDPN
GRGGRHPVRTRDELRRSSHGSPAETRSRLGSPASATHRNSPDCRRRDLMNSGSDL

>A257|LOC103632376

MGILCCFQSHTSDHAVASSPATSSSSAPSSCRNNDRRAPPERQAPGEEKSRRRNDSVDNSNLVDLVNDIVAESV
SYRHRRVAEDILKMEKAGKVTARAFTYAELSEATGGFRPESLLGEGGFGPVYRGRLGSSSGPEVAVKQLDRNGM
QGTREFLVEALMLSLLKHSNLVTLLGFCTDADHRMLIYEYMPLGSLEDHLLDLPPGRAPLDWATRMRVAQGAA
RGLEYLHDTARPPVIYRDFKASNILLDTGFRARLSDFGLAKVGPVGDKSHVSTRVMGTYGYCAPEYALTGKLTTM
SDVYSFGVVFLEIITGSRAIDTTRPPEKHNLVLWAAPRFKDKRRFAEMADPLLKGAYPTKGLYQALAIAAMCLQE
DATMRPAISDVVTALDYLTVAGGGAAANEPPAPNPNQQQQQTDIIGHAGEQA

>A258|LOC110024789

MGCFPCCGSSENGREKKVEEEGGKGGFRKEPASALPANQVMRVSSEKSKSRNSSEAKQEALVPKEGNATHIAA
QTFTFRELAAATKNFRLECLLGEGGFGRVYKGRLENGQVVAVKQLDRNGLQGNREFLVEVLMLSLLHHQNLVNL
IGYCADGDQRLLVYEYMPLGSLEDHLHEKKHHSSSKCGLTVIWRKANGGQFIPMKGIQLLFRFSMDLTISVNCDL
SHGPFLNDLGLLKHLAEYSLYVAHGKVMHASKPLNLMQARPLFKDRRKFPKMADPLLQGCYPMRGLYQALAVA
AMCLQEQAATRPLIGDVVTALSYLASQTCDPNATLPQTSRTCLSSPRGREEAKNLIVGLETKHSTDLPQSDSGRKL
GLEELERQDSASYSLGHSGKTPKALDRDPDRERAIAEAKVWGGNWRERKK

>A259|OEL14892.1

MLSWLRRFPHDIIRDRKSNASSGTSTGRRASSSTSWRNKSNSFTARIIRCASSVVDGAGRGHQHHDDDDADDA
DDDDCRPSSPPPAPPPEDNRDADGNTVVVSAQAFSFRELAEAAGNFRQEHLIGEGGFGRVYKARLRNNNNNG
QQQQQQVVAVKQLDRNGLQGNSEFVVEVLMLSMLHHPNLVNLVGYCADGDQRLLVYEYMALGSLEDHLLQ
QVPQLEQQQVLPWRTRMRIAHGAARGLEYLHDRGVIYRDLKSSNILLDADYSPRLSDFGLAKLLPAASSSSSSSS
GSSSSGKVMGTYGYCAPEYLRTGKLSVKADVYSFGVLLLELITGRRAIDASRPDGEQILLGWAVTMFGDPSRFQE
LVDPRLVMAMQGPAASELKQAVGVAAMCLQEHHALRPVMTDVVMALSFLATDDRH

>A260|OEL24530.1

MGILCCFQSHACGGSSHHDPGIPSSAASSSSATSSCRNKDWPLPQRRPGEDKSSRKNNGVDGNLVALVNEIVAE
SVSYRHKRVAEEILKMGKAGKVTARAFTYAELSEATGGFRPESLLGEGGFGPVYRGHLPPRSTGPEVAVKQLDRN
GMQGTREFLVEALMLSLLKHPNLVTLLGFCTDADHRMLVYEYMPLGSLEDHLLDLPPGRAPLEWSTRMRVAQD
AARGLEYLHDAVRPPVIYRDFKASNILLDTGFRARLSDFGLAKVGPVGDKTHVSTRVMGTYGYCAPEYALTGKLT
TMSDVYSFGVVFLEIITGRRAIDTTRPPDQHNLVLWAGPRFKDKRRFAEMADPLLQGAYPTKGLYQALAIAAMC
LQEDATMRPAISDVVTALEYLTLAGGGAADDEPAPDPNEQQQTDDDDAQA

>A261|LOC101783701

MGILCCFQSHHAGGESSDHAVPSSSTAASSSVTSNKDRPLPERRPGDYKSIRSNNSVDYSNLVTLVNEIVADSVSY
RHKRVAEEILKMGKAGKVTARAFTYAELSEATGGFQPESVLGEGGFGPVYRGRLPPRSSGPEVAVKQLDRNGVQ
GTREFLVEALMLSLLKHPHLVTLLGFCTDSDHRMLVYEYMPLGSLEDHLLDLPPGRPPLDWATRMRVAQGAAR
GLEYLHDTARPPVIYRDFKASNILLDTGFRARLSDFGLAKVGPSGDKTHVSTRVMGTYGYCAPEYALTGKLTTMS
DVYSFGVVFLEIVTGRRAIDTAREPDQHNLVLWAGPRFKDKRRFAEMADPLLQGEYPTKGLHQALAIAAMCLQE
DATMRPAISDVVSALEYLVVAGGGAGDDEEAPDPNEQQQTDDDDAQA

>A262|LOC4347926

MGIFCCFQSEDRGGDGDGDGDGAPPSTSSSGCSNSSSSSKKKNLASERSLGGSSRDNNSNLVNLVNEIVAESVT
YRHKRVADEILKIGKGKVTARAFTYGELSEATGGFRAESLLGEGGFGPVYRGRLSIKGTVTEAAVKQLDRNGMQG
NREFLVEVLMLSLLAEHPNLVTLLGYCTDGDHRILVYEYMARGSLEDHLLDLPPGAAALDWTTRMRIAQGAARG
LEHLHDAARPPVIYRDFKASNILLDSSFQARLSDFGLAKVGPVGDKTHVSTRVMGTYGYCAPEYALTGKLTTCSD
VYSFGVVFLEIITGRRAIDMARPHDEQNLVQWAAPRFKDKKLFADMADPLLRGAYPTKGLYQALAIAAMCLQE
DATMRPAISDVVTALEYLTVAGASSEPAPRPQKLQPPEDDDDDQRPAA

>A263|LOC104802722

MSGCYPCFGSSAKDGAAQETVRKDASAKDGSVAQSHHASSDKSKCNGGSEHKKKEVTVPKDGSTAHIAAQTFT
FRELAAATKCFRPECLLGEGGFGRVYKGRLESTGQTVAVKQLDRNGLQGNREFLVEVLMLSLLHHPNLVNLIGYC
ADGDQRLLVYEYMPLGSLEDHLHDLPPDTEPLDWNTRMKIAAGAAKGLEYLHDKANPPVIYRDFKSSNILLGEG
YHPKLSDFGLAKLGPVGDKTHVSTRVMGTYGYCAPEYAMTGQLTLKSDVYSFGVVFLELITGRKAIDNTRAPGEH
NLVAWARPLFKDRKKFPKMADPLLQGRYPMRGLYQALAVAAMCLQEQASTRPLIGDVVTALTYLATQTYDPN
AYSRGGGPPLIRTRYEYGGSGLDSPGTDTRSRLSSPSTHRNSPNFRRRD

>A264|M0TLB8

MGCFPCFGSASQREEEEVKKKNEDKSKSRMGSDTSKKASTPKECNADHIAAKTFTFRELAAATQNFRQDCLLGE
GGFGRVYKGRLENGQVVAVKQLDRNGLQGNREFLVEVLMLSLLHHPHLVNLIGYCADGDQRLLVYEFMPLGSL
EDHLHDIPADKEPLDWNTRMKIAAGAAKGLEYLHDKANPPVIYRDFKSSNILLGEGYHPKLSDFGLAKLGPVGDK
THVSTRVMGTYGYCAPEYAMTGQLTVKSDVYSFGVVFLELITGRKAIDNTRPAGEQNLVAWARPLFKDRRKFP
QMADPLLQGHYPARGLYQALAVAAMCLQEQAATRPLIGDVVTALSYLASQTYDPNSATGQNLRQRDPVKGLS
KGAKVGRGARDSPKNRDLVREHAVAEAKLWGEKWRERQRRNAPDSFESTHE

>A265|A0A199WAZ5

MGCLPCFESRRKRDELGGDGDRREEKRKEEQPMVPPSLDRVSSDLKNRSSSESKKGIDAPNDSSGIVAVKQLDQ
NGLQGNREFLVEVLMLSLLHHPNLVNLIGYCADGDQRLLVYEFMPFGSLEDHLHDLPPEKEGLDWNTRMKIAA
GAAKGLEYLHNKANPPVIYRDFKSSNILLDKDFHPKLSDFGLAKLGPTGDKSHVSTRVMGTYGYCAPEYAMTGQ
LTVKSDVYSFGVVFLELITGRRAIDSTRPHGEQNLVSWARPLFNDRKKLPKLADPRLQGRFPMRGLYQALAVAS
MCIQEQAASRPNIADVVTALSYLASSYDPNASSPTSSNKSGGGEHDDRRSRGTGGRLVQKNEEGGGGLEREDSP
REGVGSVMNKDFDRERAVAEAKLWGENWREKIRANAVAREAANNSS

>A266|PAN11089.1

MGILCCFQSHAGGGGGHDQAVPSSSAASPSSATSSSGNKDRPLSERNNSVDYSNLVALVNEIVGDSVSYRHKRV
ADEILKMGKAGKVTARAFTYAELSEATGGFRPESLLGEGGFGPVYRGRLPPKSTGPEVAVKQLDRNGMQGTREF
LVEALMLSLLKHPHLVTLLGFCTDANHRMLVYEYMPLGSLEDHLLDLPPGRAPLEWATRMRVAQGAARGLEYL
HDTAQPPVIYRDFKASNILLDTGFRARLSDFGLAKVGPSGDKTHVSTRVMGTYGYCAPEYALTGKLTTMSDVYSF

GVVFLEIITGRRAIDTTRPPDQHNLVLWAGPRFKNKRRFAEMADPMLQGDYPTKGLHQALAIAAMCLQEDAT MRPGISDVVTALDYLTVAGGGTTDIDDDDQGPDPDEQQQTDDDAQA

>A267|OEL19995.1

MGCCFSSEGGGSDRNERVVTSQIPRAEQAESTVAVPTEPVIPEIVESVVPVVTVEAPHDEFITNQGPEGYGEDFT YRELYFATCGFRVDRLLGQGGFGQVYKGFLESTNQEVAIKSLDLQGQQGDREFLTEVVMLSKLHHPNLVKLVGY CTNHGQRILVYEYMPLGSLHSHIHDLPPGRQPLDWSTRIKILLGAAKGLQYLHDKVKPSVINRDVKCANILLGEGY HPKLSDFGLAKLGPTGDNTHVSTRVMGTPGYCAPEYLMTGRLSVSTDIYSFGVVMLEVLTGRVARDERLPESER NLARWAVNRILRPDTPNMLDPALQGQRSAYHFYHAFLLAARCASELPCVRPPIGEVVSTLDMLSKPRRRRLERG GPSTPTRSSSDGNQAQVQEQGEGSSSDGNQAQVQDQGEGT

>A268|LOC101755770

MLSWLRRFPHDVMHRKDNGGAAGRRAASSTTWRNKNSNSFTARIIRCASSVARRRRFDDDDDDDDRQQLPS SPPPPGRSPPPAESKLDRNNKVGVVSARAFSFRELAEAAGNFRQEHLIGEGGFGRVYKARIVKEQQQQEEEEEQ VVVAVKQLDRNGPQGNGEFVVEVLMLSMLHHPNLVSLLGYCADGEQRLLVYEYMALGSLEDHLLLVGDHHRP LLPWRTRMRIACGAGRGLEYLHERGVIFRDLKPSNILLDDHHNPKLSDFGLARLLPPSSSSTSSTGSSSSRVMGTY GYCAPEYLRTGKLSAKSDVYSFGVLLLELITGRRALDASRPDGEQSLVGWAAPMFGDPARIHELVDPRLVMAMQ APPAPELKQAVGLAAMCLQEHHALRPVMTDVVFALDFLATDRP

>A269|A0A5N6PS69

MGLGCFPCFDSSEEEVEEIFNHHTLSQADRQPSGFDRLKTRSNVVSLSLRREASQTFTFRQLAAATNNFRPDCFL GEGGFGCVYRGRLQTTGQVVAVKQLDRNGVQGNREFVVEVLMLSLLHHPNLVNLIGYCADGHQRLLVYEFMP LGSLEDHLHDVPVDKEALDWNTRMKIAAGAAKGLEFLHDKANPPVIYRDFKSSNILLDEGFQPKLSDFGLAKLGP TGDKSHVSTRVMGTYGYCAPEYAMTGQLTVKSDVYSFGVVFLELITGRKAIDSTQPHGQQNLVTWARPLLNDR RKFTSLVDPRLEGQYPMRGLYQALAVASMCVQEQAAARPLIADVVTALSYLANQEYDPAHNIMKPKVERNEP MVCSRWDHLDGSEKQETVNMLNRERAVAEAKMWVEKRRQTAS

>A270|LOC111258252

MLSWLRRFPHDVLHRKDNGGAGRRAASSTTWRNKNNNSFTARIIRCASSAARRRFDDDDDDRQLPSSPPPPG PPPAEEEEDDDDDDYSRSKDRNNKVGVVSARAFSFRELAEAAGNFRQEHLIGEGGFGRVYKARIKEQQEQVVV AVKQLDRNGPQGNGEFVVEVLMLSMLHHPNLVSLLGYCAEGEQRLLVYEYMALGSLEDHLLLISNHPLLPWRTR MRIACGAGRGLEYLHERGVIFRDLKPSNILLDDHHNPRLSDFGLARLLPPSNSSSSSNSSSSSTGSSRVMGTYGYC APEYLRTGKLSAKSDVYSFGVLLLELITGRRALDASRPDGEQSLVGWAAPMFGDPTRIHELVDPRLVMAMQGPP APELKQAVGLAAMCLQEHHALRPVMTDVVFALDFLSTDRP

>A271|Oropetium_20150105_05286ª

MGCLPCFGSGGEGADKKGSVRKDASSDRRVSRDGSDKSKPQGGSDSKKDAVILREGSNQHIAAHTFTFRELAA ATKNFRQDCLLGEGGFGRVYKGRLENGQPVAVKQLDRNGLQGNREFLVEVLMLSLLHHDNLVNLIGYCADGD QRLLVYEFMPLGSLEDHLHDIPPDKEPLDWNTRMKIAAGAAKGLEYLHDKASPPVIYRDFKSSNILLGEGYHPKLS DFGLAKLGPVGDKTHVSTRVMGTYGYCAPEYAMTGQLTVKSDVYSFGVVFLELITGRKAIDNTKPHGEQNLVA WARPLFKDRRKFPKMADPLLQGGGSSDQRRLRSPNHHSPDLRRRDSTTTSKYEAEVSRTNSGSGSGRRSGLDD VEGLLVGSPAHGRKRESPRTSDRQRAVAEAKTWGEISRERK

>A272|GAV91719.1

MGFICIMAENVTSESGPPTGAIAGGVAAGAALLFAAPAIGFAYWRRRKPRELFFDLPAEEDPEVHLGQLKRFSLR
ELQVATDTFSNKNILGRGGFGKVYKGRLADGSLVAVKRLKEERTPGGELQFQTEVEMISMAMHRNLLRLRGFC
MTPTERLLVYPYMANGSVASCLRERSPSSPPLDWPTRKRIALGSSRGLSYLHDHCDPKIIHRDVKAANILLDEEFE
AVVGDFGLAKLMDYKDTHVTTAVRGTIGHIAPEYLSTGKSSAKTDVFGYGIMLLELITGQRAFDLARLANDDDV
MLPDWVKGLLKEKKLEMLVDPDLQNNYVEAEVARLIQVALLCTQSSPMERPKMSEVVRMLEGDGLTEKWEE
WQKAEEVRHQLELMPPICSEWIVDSTKNIHAIELSGPR

>A273|A0A287MGW8

SPCRVAKFPSYKLKLQGGSDPKNTHLTIPRDGNSQNIAAQIFTFRELAAATKNFRQDCMLGEGGFGRVYKGRLE
NGQAVAVKQLDRNGLQGNREFLVEVLMLSLLHHTNLVNLIGYCADGDQRLLVYEFMPLGSLEDHLHDVPPEKE
PLDWNTRMKIAAGAAKGLEHLHDKASPPVIYRDFKSSNILLGEGFHPKLSDFGLAKLGPVGDKTHVSTRVMGTY
GYCAPEYAMTGQLTVKSDVYSFGVVFLELITGRKAIDNTKPQGEQNLVAWARPLFKDRRKFPKMADPMLQGRF
PMRGLYQALAVAAMCLQEQATTRPHIGDVVTALSYLASQTYDPNAPTQHTRSNSSTPRARNMGGRNSEQRN
GRSPNHHSPRTSKHGGEVSRTSSNGGDSGRRSGLDDMDMAG

>A274|LOC102721957

PAPAVAPSSSSSNKEKSSNANAAGAGASTSERSHGGGSSRSNNNNLVDLVNEIVAESVTYRHKRVADEILKIGKG
KVTARAFSYAELSEATGGFRPESLLGEGGFGPVYRGRLKGKGGSVTEAAVKQLDRNGMQGTREFLVEVLMLSLL
EHPNLVTLLGYCTDADHRILVYEYMPRGSLEDHLLDLPPGSASLDWTTRMRVAQGAARGLEHLHDAARPPVIYR
DFKASNILLDRSFQARLSDFGLAKVGPVGDKTHVSTRVMGTYGYCAPEYALTGKLTTCSDVYSFGVVFLEIITGRR
AIDMGRPADEQNLVQWAAPRFKDKKRFAEMADPLLRGAYPTKGLYQALAIAAMCLQEDATMRPAISDVVTAL
EYLTVAGASSEGAPRPQKLLQQPQEDDDDDDDDRP

>A275|LOC103435759

MSGGIRTSVKGYTKVKRGDLAMDTTHENTERDNASETTFINSQSSNAPQLAAAADCIDKKPANEDSPSNPPRD
GLNSNVRVSSVSVPSTRQIEGEILQPFNLKSFRIDELKTATRNFRIDPTVNGVRMRRVFKGWIDENSLTATVPGTG
MAIAVSYVLFKHEMPLQSRNEWLTEVNRVRQLHNQNLVRLIGYCLEDDCRLLVYEFMAKGSLDNHLFLRGYFEP
LSWNVRMKIALGSAKGLAFLHGDDANILYRDFKACNILLHSAYNAKLYNFCVSKDGSASYRNHVSTDVMATHGY
AAPEYIATGDCTAKCDVYSFGVVLLEMLTGRRAVDKNRPSEEQNLVEWARRYLASKRKVVXIFDXRLEGQYSXG
VALGAANLASQCLSIEPRCRPNMDEVVKALEKLQG

>A276|LOC109758699

MRNCFVSLTGTATTVPSTSQCEGKILQSEDFRRFAFKELKRATMNFWPDGLLSDGHFGSVHKGWVNEATFAPA
SWGTGMPITVKKLNQGSVQGHMEWLVGASQYQSIPIHSSLQFCIYAEVKYLGQLSHPNLVKLLGYCNEDRSQCL
LVYEFMPQGSLENHLVRRGLHFQPLSWHLRMKVALGAAKGLAFLHSGNVPVIQHEFNTSNVLLDSSYNAKLSDF
GLARMEPMDDLSTDVHAAPEYVDTGHLTMKSDVYSFGVVLLEILAGRPALDKNRPSIEHNLVEWARQYLTSERR
ISHIMDAQLGGQYSLACAHKAATLVLQCISVHPRDRPGMEQVVAALEQLQDANETGASGQSDVRLGMEQVVA
VLGQQLQDDKETVASGQQKVSGGGGGACGFFRMCGGGRQ

>A277|LOC111905833

MGNCCRTRRNGDNHYRYVQPHASEVVTAGGAKEPYRSDSKKLTQNLPSPNPRNGDEMILSPHLKVFAFSELIN
ATKYFSPDYLLGEGAFGYVYEGWLNKETLSPVEPESGMPVAIKKLKRLGFQGHMEWLSDVSYLGGLHHRNLVNL

IGFCYEGENRLLVSEFMPGGSLEHHLFRRGGEPFSWALRLKVAVEVAQGLAFLHASQSKIIYCDFKSSNVLLDMD
YNVKLSVFGLAKADPSGDWSHVTSQITGTEGYTAPEYFAAGRLTTKCDVYSFGIVLLELITGRRAIDYKRVAEEKRL
LEWVRTQLRDTKKVFKIMDSRLEGKYSRKAASVVANLALQCCYPEATYRPHMSDVLSILEKIPSPRAFRNKDMSN
SKSKINAHSSNGQSNGHDHQHESAADMVHH

>A278|A0A200R663

MGWFPCSGNSSKKARRKKPDDQIKPTSSEKLKVNPAVDVKEGSKDGSKDGGSNNIAAQTFTFRELAAATKNFR
ADCLLGEGGFGRVYKGRLESTNQIVAIKQLDRDGLQGNREFLVEVLMLSLLHHPNLVNLIGYCADGDQRLLVYEF
MPLGSLEDHLHDLPPDKKRLDWNTRMKIAAGAAKGLEYLHDKANPPVIYRDLKCSNILLGEGYSPKLSDFGLAKL
GPVGDNTHVSTRVMGTYGYCAPEYAMTGQLTLKSDVYSFGVVLLEIITGRKAIDNSKAAKEQNLVAWARPLFKD
RRKFPQMADPALQGQYPVRGLYQALAVAAMCVQEQPTMRPLIADVVTALTYLASQAHNPQTESVQNSRMAP
GTPPRTRRGDSDKRFNGGNSGSGSERDQSSRGMIN

>A279|KFK32284.1

MGCFGRTGKSSKRSETKKKDFTDGICVRKNVNRDTQIQSSSDCFKVSPCGDVTKEEEDQLSLDAKDLNVAGKKA
QTFTFEELTLSDCFLGEGGFGKVYKGFIHNISQVVAIKQLDRNGSQGIREFVVEVLTLSLADHPNLVKLIGFCADGV
QRLLVYEYMPLGSLEHHLHDLPCGRKPLVWNTRMKIAAGAARGLEYLHDRMKPPVIYRDLKCSNILLGEEYHAKL
SDFGLAKVGPRGDETHVSTRVMGTYGYCPPEYAMTGQLTFKSDLFSFGVVLLELITGRKAIDHTKARKDQNLVG
WALPLFKDRKNFKKMVDPSMEGDYPVKGLYQALAIAAMCVQEKPCMRPVISDVVMALDHLASCSYDSNYKRK
TETRVDDEEKRRAESKVCMEEQQEIKQHDQ

>A280|LOC110883144

MVRNVNLCLLMGACFSSDLDEQHSFQPEPDEIQPSEQRFDDSSNLESPSSVEVISRDIEDLKQNLGYTNLDSFTY
DEMRMATELFNSNLVIGEGGFGVVYKGIIDESVRSGYTRVEVAIKKLNPMGLQGEKEWLTELNYLARLRHPNLM
KLIGYCCEGTHRLLVYEFMSAGSLEDIMFAPLKKGSIHPLSWGKRMKIALDAAKGLQYLHTTERPVIYRDFKSSNIL
LDENFNAKLSDFGIAKDAPMGKTTHISTRVVGTEGYTAPDYAQTGHLRVNSDTYALGVVLLELALGLRAIDDSRA
SDMRSLPDFANQYMTHKRVMVDLIDPRMERNYSRKDAVKVINLALQCLHDSPVMRPPMSKILKILESVQTPEA
SFQSESESVTTADRKKTRKSRYKLKLK

>A281|LOC103871132

MVCTIENAAVHGAAVMVYIIMIVLIFILIYIVCKRRSLRSKTHVEGEFKSLDPMINSFSLRQIKAATNNFDTANRIGE
GGFGPVHKGKLPDGTIIAVKQLSTGSKQGNREFLNEIGMITALHHPNLVKLYGCCVEGDQLLLVYEYVENNCLAR
ALFGPQETQLRLDWPTRRKICIGVARGLAYLHEESRIKIVHRDIKATNVLLDKEMNSKISDFGLAKLNEDDNTHIST
RVAGTFGYMAPEYAMRGHLTDKVDVYSFGIVALEIVHGRSNKINQSASNYNTPYVIDWVTILREQNNLLELVDP
RLGSDYNREEALTMLHVVILCTSPDPSDRPLMSEVVKMLEGKKMVELERLEEASEYRETKRLENMNTMKKYYE
MIGSETSMTMTMTLTDQTTSSKH

>A282|A0A1U7ZXV7

MGWFPCSGNPNKAKKKKPIDRIESNPEKLQISPPLDEKKEGPKDGGSDHIAAQTFTFRELAAATKNFRADCFLGE
GGFGRVYKGRLESTNQIVAIKQLDRNGLQGNREFLVEVLMLSLLHHRNLVNLIGYCADGDQRLLVYEYMPLGSLE
DHLHDLPPDKKRLDWNTRMKIAAGAAKGLEYLHDKANPPVIYRDLKCSNILLGEGYYPKLSDFGLAKLGPVGDKT
HVSTRVMGTYGYCAPEYAMTGQLTLKSDVYSFGVVLLEIITGRKAIDNSKAAGEQNLVAWARPLFKDRRKFAQ

MADPMLQGQYPVRGLYQALAVAAMCVQEQPTMRPLIADVVTALSYLASQKYDPESQPLQISRMLPPTPPRTK
KDSDKRINGGSESERDQTGVGERKFLFIS

>A283|LOC109733467

MSCFSCFKPPQADEEEEEVDKAPAGSIKRLGSRRRSLRSKSGGSFAASRQPSTRPSNITSCSARAFTYDQLAAATG
DFRADCLLGEGGFGRVYRGRLDDGQLVAVKQLDLEGLQGDREFVVEVLMLSLLHHDNLVSLVGYCSHGHQRLL
VYEYMALGSLADHLLLLDDRLATPRATARAALSWETRMRVALGAARGLEYLHETANPAVIYRDLKSSNVLLDDAF
CPKLSDFGLARLCSTSSSAAGPGPGERSPRVMGTYGYCAPEYIRTGRLSVKSDVYSFGVLLLELITGRRAVDSARPA
PEQVLVTWAAPMFKDSKRYRELADPLLRGDFPERDLNQAVAVAAMCLQDQASARPCMSDAAVTLSFLAEAAA
ASAAQPLPLPPPQPQADETLREEEA

>A284|103634484
MGSCFSLEGGRSESIVPAQRSQIPEDEQVEPTVTSELAIPVPEVAESVVMLEALRTGFITDQRPERHIRVRSFTYDE
VCAATHGFEVDRFLGQGGFGQVYRGFLESTNQEVAIKRLDLQGQQGHREFVTEVLILSNVHHPNLVKLVGHCTS
HDQRILVYEYMPLGSLNSHIHDLPPGQQPLDWSTRIKILLGAAKGLEHLHHNLNPPVINRDVKCANILLGAGYHP
KLSDFGLAKLGPTGDNTHVSTRVMGTPGYCAPEYLMTGKLTVKTDIYSFGVVMLEVLTGRMARDERLPESERNL
VAWALNFLRRRELDNLLDPALRGQCPQACLEHAFFVVSRCISESPNTRPSMRDVVASLTVISEFRNRNTRRLERG
GRSTPTRTPDRNHRGDDQGEGS

>A285|LOC103965951

SFNNEGENSRKVKIATVVVGIAIVFSGVLLVGYVIRRRKRKNKEIRERNEDNEGAPRGDMELPLFDLMTVASATD
NFSSNKKLGEGGFGPVYKGTLADGQEIAVKRLSRSSGQGLNEFMNEVRLIAKLQHRNLVRLLGCCVQGEEKLLLY
EYMPNGSLDSSIFDETRREPFDWPKRFNIICGIARGLVYLHQDSRLRIIHRNLKASNVLLDNEMNPKISDFGLARLL
TEGDSTVANTERLVGTYGYMAPEYVMGGQFSVKSDVFSFGILVLEIVTGRKNKGFSDPSNSRNLSEQLWRLWN
EGKPLELIDTCLASSGTLSEVLRCINIGLLCAQHDPDDRPSMAAVVIMLGSETALAQPKQPGFFMEKGTPEAGSN
SGNQTCSTNELSISLLEAR

>A286|OEL25261.1

MGRARSPPPPTPRMRAVAVKQLDRNGLQGNREFLVESLYPWSLYFISSFHFLAVATLWSFNWVVSFLYIDLPPD
KEALDWNTRMKIAAGAAKGLEYLHDKASPPVIYRDFKSSNILLGEGFHPKLSDFGLAKLGPVGDKTHVSTRVMG
TYGYCAPEYAMTGQLTVKSDVYSFGVVFLELITGRKAIDNTKPQGEQNLVAWARPLFKDRRKFPKMADPMLQG
RFPMRGLYQALAVAAMCLQEQAATRPHIGDVVTALSYLASQAYEPNAPVQHSRSNSSTPRARNPAGWNDDQ
RSVRSPNHHSPDLRRRDAARASKYGAEVSRTSSTGDSGRRSGLDDMDMTGSQVGSPAQTGRRRENPGAAAD
RQRAIAEAKMWGEYSRERSNGHGSFDSTNE

>A287|LOC101785262

MGSCFSSEGGGGSRREVRAMTSQIPRAEQAESTEAVIREMSMVTVDASHAGFITNLDLECCIDGKAFRYADLYV
TTGGFSVDRLLGHGGFGQVYKGFHNGTNEEVAIKRLDLQGQQGDREFITEAVMLSSLHHPNLVKLVGYCADLG
QRILVYEYMPLGSLYSHIHDLPPGRQPLDWSTRIQILLGAAKGLEYLHNKVKPPIINRDVKCANILLGEGYHPKLSD
FGLAKLGPTGDNTHVSTRVMGTPGYCAPEYLMTGKLTVSTDIYSFGVVMLEVLTGRIARDESLPESERNLASWA
VNPTLRPDITNLVDPALQGQCSASRLYHAFLLAARCVSELPAMRPAITEVVRTLDKISKPATKKRQLERGGPSTTA
RTSSGGNQAQVKDQGQGS

>A288|A0A1R3KLX9

MLVWLASVSIFTMGMLNEPVFGFYTCGSKPIFSIVDMKFGFHIMVEKLKGNSVSDAKKETAKDGGSDHIAAHTF
TFRELAAATKNFRADCLLGEGGFGRVYKGRLESTNQVVAIKQLDRNGLQGNREFLVEVLMLSLLHHPNLVNLIGY
CADGDQRLLVYEYMPLGSLEDHLHDLPPDRRRLDWNTRMKIAAGAAKGLEYLHDKASPPVIYRDLKCSNILLNE
GYHPKLSDFGLAKLGPVGDKTHVSTRVMGTYGYCAPEYAMTGQLTLKSDVYSFGVVLLEIITGRKAIDNSKTGGE
QNLVAWARPLFKDRRKFPQMADPLLQGQYPVRGLYQALAVAAMCVQEQPNMRPLIADVVTALTYLASQKYD
PETQSVQGSRTGSSTPRMRRE

>A289|LOC112034189

MEGISQSISKLLRWSGKVRSDEIKQYYSSVLPEGLCRRFSLAEIKKATNYFADDLVIGEGGFGKVYEGFIDDGGIRV
AIKRLKIGSMQGLPELKNEVLLLCQLRHPNLIPLIGYCIDEGEGCIVYEFMVNGNLRDHIVGTNYDPLPWRQRLAIC
IGVARGLQFLHTGIKHTIIHRDVKMSNILLDEKLEAKLGDFGLSKMGPPSLSETLIRIQSLVKGVYGYLDPEYHRRQ
VLTDKSDVYSFGVVLLELLCGRKVVDDKFGAERWHLVYWARQCKRDGTINETIDPYLMGKIAPECFKIYVSIASSC
VREEGKDRPAMGEVEVGLEHALQLQESADAAREEGEYDYFINELTCNDSPIELNRYGRGASPSTVENPFPDSDST
EFSYND

>A290|PAN50235.1

MGSCFSSEGGGSRRKARTATSRIPRAEQSESIVPTELVIPEMVESVDTVEAPHAEFITNHGLEGYKNFTYNELYVA
TDGFRVDRLLGQGGFGQVYMGFLDSTNQEVAIKRLDLQGQQGDREFANEVVMLSSLHHPNLVKLVGYCANN
GQRILVYEYMPLGSLYSHIHDLPPGQQPLDWSTRINILLGAAKGLEYLHSKAPPVINRDVKSANILLGEGYHPKLSD
FGLARLGPTGDNTHVSTRVMGTHGYCAPEYLMTGKLTVSTDVYSFGVVMLEVLTGRIARDESLPEPNRDLALW
ADEQGIANLVDPALQGQCSLNRLSLAFAVARRCVRELPDRRPTMPEIVTALTRISAPEPRRRRFERGGPSTPARTS
SDGNQAQGQDQGEGS

>A291|Os03g0283900

MGSCFSSEGGNESRKESAERPQITPDETAASEMDVNLNTTNADSISNHGMERLIHGQSFTYGELYAATGGFSDD
RFLGEGGFGQVYRGVLDNSQEVAIKILNLQGNQGDREFITEASVLSKLHHTNLVKLIGCCQDGDQRLLVYEYMPL
GSLKSHLHDLSPDKKPLDWNTRIKILVGAAKGLQHLHVNVDPPVINRDVKSENILLGDGYHPKLSDFGLAKMGPT
GDDTHISTRVMGTLGYCAPDYLESGKLTVQSDIYSFGVVMLEVITGQKVIDDSRAKPERNIVEWAIPKINKKDFPK
LADPVLNGQYHMRSLFRALTVAALCVDRTANRRPDITAVVDALTQISESQSSRKRWSSRLQSSVGSSASTEPRIE
DWNQAKDQGEGS

>A292|102712431

MGCCFSSEGGDESVKELAERSQITPNETAASASEMVVNLDPRHVEFISNHGLERLVQGKLFTYGELYAATGGFSD
HLFLGEGGFGQVYKGMLDATGQEVAIKILNLQGNQGNKEFFTEITVLSKVHHANLVKLIGHCVDGDQRLLVYEY
MPLGSLKSHLHDLSPDKKPLDWDTRIKILVGAAKGLQHLHVNADPPIINRDVKSENILLGEGYHPKLSDFGLAKLG
PTGDDTHISTRVMGTLGYCAPDYLESGKLTVQSDIYSFGVVMLEVITGQKVIDDSRAKTERSIVEWAMPMINQK
DFAKLADPVLNGQYHMRSLFRALIVAAMCIDRTANRRPDITLVVDALTQISESQSRRKRWSSRLQSTVASNASSA
EPREDQGESS

>A293|A0A3S4NUF4

MSWFSCIGKSHKKSKEKPEKRKEESSLSEKLKLKPLLDMKKEILKDGADHISAQTFAFRELAVATKNFRNDCLLGE
GGFGRVYKGRLESTNQIVAIKQLDRNGLQGNREFLVEVLMLSLLHHPNLVNLIGYCADGDQRLLVYEYMPLGSLE
DHLHDLPPDKKRLDWNARMKIAAGAAKGLEYLHDKANPPVIYRDLKCSNILLGEGYHPKLSDFGLAKLGPVGDK
THVSTRVMGTYGYCAPEYAMTGQLTLKSDVYSFGVVLLEIITGRKAIDNSKGVGEHNLVAWARPLFKDRRKFQQ

MADPMLQGQYPTRGLYQALAVASMCVQEQPTMRPLVADVVTALTYLAAQKYDPDSQPVQSFHLAPATPPRT
RNRTAGLRKAKVED

>A294|LOC108330065

MEESEERTLIALGSTTDRRARSESPIAIKRAKAFSFRELAAATKNFRSRLLLGRGGFGSVYKGQLKNGQDVAVKVLE
KISQQEDQEFVTGVEMLSLLYHPNLVTLIGYCCERDQRLIVYEYMPLGSLENHLFDRPLGREPLDWNTRMKIASG
VAKGLEYLEIWRDLPVMFGELRSSNILLDEGYHPKLSYFGIPKFGNSSHVSTGLVGTYGYCAPEYAVTGNLTPECK
VYGLGVVLLELISGRKAIDKSRSPETCHLVKWAQPILKDISEYHRLVDPLLHGSYPVEWLNQAIVIAAMCLHEKAD
RRPTIREVVSALTYIESQQKQCNRRGRHPCSFFYPSLVRKESGLEKERKKGEFERGSTGYGADSGKQVVGYIEMLE
I

>A295|SELMODRAFT_110362

MEKYHPPIQKTSGNLVLSTKAKELDHHIAAASFSCVEIQAATRNFGKHNLIGEGLLGSVYRSEFPSGQVFAIKKLDS
TSSYFKDEETLRRIISSISSLRHGNIVELCGFCVEKNQCFLVYPYFSTGTLHDHLHSSPEKTLSWNQRMKVSLGAAR
ALEYLHEVASPVTVHRNFKSANILLDQEFNPFVSDTGLAALIPLSLERQISSQMLGSFGYSAPEYALAGIYTVKSDV
YSFGVLMLELLTGREPLDSSRPRSKELSLVAWAVPKLNDLDTLASIVDPKLEGMYAAKALSRYAEIITQCVQAEAV
DRPAMSEVVQSLAGLMQRAGTNKKTVSREMPLQQSPGRAPLRNSGNEVASTSSPSFEKSFEAQNLSDSIKFSM

>A296|LOC103650676

MVGCFACFKPAGDQEADEEAPLPSTSRRRGRSLRLPSCSSSNKGNQQADNPIAAASSSNVDSRARAFTYDELAA
ATENFRAECLLGEGGFGRVYRGRLESGQVVAVKQLDREGVQGNREFVVEVLMLSLLHHPNLVNLVGYCADGEQ
RLLVYEYMALGSLADHLLLDTSSRDKGNAAPEQEQRALSWETRMRVALGAARGLEYLHETANPPVIYRDLKSSN
VLLDDALCPKLSDFGLAKLGPIGDRSPRVMGTYGYCAPEYVRAGTITVKADVYSFGVLLLELITGRRAVDSTRPTAE
QLLVAWAMPMLRDSKRYRELADPLLRGGFPERDLKQAVAVAAMCLQEEASARPLMSDAAMTLAYLAEAAASA
AASSSTS

>A297|PKI39317.1

MLSLLHHPNLVNLIGYCADGDQRLLVYEFMPLGSLEDHLHDLPPDKEPLDWNTRMKIAAGAAKGLEYLHDKAN
PPVIYRDLKSSNILLDEGYHPKLSDFGLAKLGPVGDKTHVSTRVMGTYGYCAPEYAMTGQLTLKSDVYSFGVVFLE
LITGRKAIDNTRAPGEHNLVAWARPLFKDRRKFPKMADPLLQGRYPMRGLYQALAVAAMCLQEQAATRPLIGD
VVTALTYLASQTYDPVAAAAQNNRVGPSTPRLRDDRRSLADGGLDSPEGGTRHGSPSAHRNSPDFRRRDRGRE
LSSAGTEAAKTESGGGSGRKWGLDESELQDSQRDSPVSVGRARERNRDLDRERAVAEAKVWGENWREKKRA
GAVGSFDSTNE

>A298|LOC109781043

MANICGGREKARVAADYRPSSPDEESGLQEPNSGTMNSKASSSATTGFMDSVDSDGTNGATKQGCFLEGQILE
APNLRKFTFLEIQTATKNFRPDRLIGFGGFGWVYRAWVDEKTMNPTTSGPHMAVAIMHGKEEWQKFLTVNLR
SDINLLGRFSHPNLVKLLGYCWEGKEVFLVNEFMAQRSLEYHLFTVSGRCPPLSWEQRLKIAIGAARGLAFLHASE
KKAMYRDFKASKILLDADYNAKLSDFGLAKLGSPGNSRMTIMPTRTHGYAAPEYVISGLLYEQSDVYSFGVMILE

MLSGQLAKDPNRPKGQTNIIDWAKSLADRRKLSHFMDPRLKGQYNSKQALQALHVALSCLAGELRSRPSMKVV
LKALEQI

>A299|EMS66854.1

MANICGDPGKAHVAADYRPSSPDDESVLQEHNSGTMNSKASSSAATSVMASVGSDGSNGGTKQGSFLEGQIL
ETPNLRKFTFLEIQTATRKFRPDGLIGFGGFGWVYKGWVDEKTMNPTTSGPRMAVAIMHGKEEWQKFLTVHL
QSDIKLLGRFSHPNLVKLLGYCWEGMELFLVYEFITQGSLERHLFFRGGCAPLSWEIRLKIAIGAARGLAFLHASEK
KAMYRDFKASRILLDADYNAKLSDYGLAKLGSTGNSRMTIMPTRTHGYAAPEYVITGLLYEMSDVYSFGVMMLE
MLSGQRARDPNRPKGQMSIIDWAKPLVDRRKVARLMDPRLKGQFNSKQALQVVHVALSCLDRDPSCRPSIKV
VLEALEQI

>A300|PAN26288.1

MVGCFACFKPAGEEEEAPSPSPSRRRGRSLRLSCSSGRNQQAGGDTAAASIIADRRSSSSRARAFTYGELAAATD
NFRAECLLGEGGFGRVYRGRLESGQVVAVKQLDREGAQGNREFVVEVLMLSLLHHPNLVNLVGYCADGEQRLL
VYEYMALGSLADHLLLPAGGEQDEPQRALSWETRMRVALGAARGLEYLHETANPPVIYRDLKSSNVLLDDALCP
KLSDFGLAKLGPVGDRSPRVMGTYGYCAPEYVRAGNLTVKTDVYSFGVLLLELVTGRRAVDSSRPPAEQLLVAW
ARPMLRDGKRYRELADPLLRGDFPERDLKQAVAVAAMCLQDEASARPLMSDAAVTLEYLAEAAASLAAPAASS
SSS

>A301|LOC104900857

MQGRIWMDKEKEEDLNELSGPDSVQFNFSEINAATNSFAADNKLGEGGFGSVYKGRLGNGQEIAVKRLSIHSR
QGFREFKTEVLIIIKLQHKNLVRLLGFCIEKEEYILVYELLPNLSLDKFLFDTKKRANLDWRTRYNIIIGIARGLLYLHED
SRLTIVHRDLKSSNILLDEAMNPKIADFGMARLFGAEQIYASTKLIVGTYGYMAPEYAMHGHFSVKSDVYSFGMII
LEILSGQSCTLNNPSEYENSLLYHTWMCWNVKQPLSVVDMSLSGNFSVEEVIKCIHIGLLCIQDEASRRPTMPWI
VNQLNNNSTTDLPEPAPPLMRHPVDVQVISSETIPSETTHTNVGSSSQDLGKFSGTREISELLPR

>A302|SELMODRAFT_80744

MGCFQVHQSDGERRKRKRRRILWWSFSFVFSRNSGRSSSSGGSSDSSGELNCQRLQELRQFTLAELKSATRNFS
AAEKLGEGGFGCVFRGHIKSKKTDERIDVAVKQLNVKGQQGQKEWLNEVTYLRMVDHPNLVKLLGYCLEHDDR
GPQCLLVYELMPNKSLDDHIFQSRRPVIPWGQRLQIALGTARGLAYLHEEMKPPIIYRDLKSANILLDNEFRPKLS
DFGLARDGPAMGNTHVTTAVVGTAGYAAPEYVQTGHINAKSDVWTFGMVLLELLTGRRALDMNRPRSERSLA
DWVKPYSSDSKKFRKIIDPRLKTNFSSSEARTLLWVAQKCIAKNPKLRPKMSEVVKQLEGILVVTAPVEKPAEVP

>A303|PIN14366.1

MLSLLHHPNLVNLIGYCADGDQRLLVYEYMPLGSLEDHLHDLLPDKQPLDWNTRMKIAAGAAKGLEYLHDKAN
PPVIYRDLKSSNILLDEGFFPKLSDFGLAKLGPIGDKTHVSTRVMGTYGYCAPEYAMTGQLTLKSDVYSFGVVLLEL
ITGRRAIDNTRGAGEQNLVAWARPLFKDRRKFPKMADPLLQGRYPMRGLYQALAVAAMCLQEQAATRPLIGD
VVTALTYLASQAYDPNAPSTHSNKVGPSTPRHRDDRRNTSDVTDSPNYGSPSTHKNSPDYRKINIGSDLRRIETG
GGSGRKWGQDESDRPDSQRDSPASARARETLRNRDLDRERAVAEAKVWGENWRERKRANALGSFDGTNE

>A304|LOC111886728

MGGCFSSNQDANDAKQLPKKDYWEKGSYPHHPSILISDEFKFGDNLDHKGITFTKDVETAHGVVQFSFRELATA
TKNFCIESFMGEGDFGCVYKGRLERSGQVVSVQHRHRTCDQGIIEFFKEVIKLSLFHHANLVNLIGYCAYGDHLLL
VHEFVPLGSLKDHLHDLLPDQKPLDWNTRMKIAVGVARGLEYLHKTSNPPVVYRDLKPSNILLDEGYHPKLSNFK
LGKHTPFDNITSFPSFRVDGTYTHCAPEGPIIFDSDVYSFGLIFLELITGRKVIDYTRPYEEQCLVYWARPLLKDRKKY
AEMADPLLEGRYPPERLKEALVVAAMCIQEGIAARPDIRSVVAALTYIASSTYDPNA

>A305|PAN26317.1

MGNCWGTNQQQTSSSSRRVSAAASIIAAVAPRSEGDILQSANVRSFTFNELRTSTRNFRPDSVLGEGGFGFVFK
GWIDEKTFAPARPGTGMVIAVKKLNQEGFQGHREWLAEVNYLGQLSHPNLVRLVGYCLEDEQRLLVYEFMPR
GSLENHLFRRGSHFQPLSWNLRMKVALGAAKGLAFLHGEKAKVIYRDFKTSNVLLDSKYNAKLSDFGLAKDGPT
GDKSHVSTRVMGTYGYAAPEYLATASVWCSWSCCPGGAPWTRTGRPASTTWWSGRGRSCATSAASSASWT
RGWAGSTRCPAPRRRRRWRCSASPATRRTGPPWTRWWRRCSSSSNMMMRMTMTMPPPPPRTPWNSKED
D

>A306|LOC8285769

MIIGRIWYIELSSVNSKFSDKGGKSAKTSPKVLTATLPSTPQSKGEILQSSNLKSFSFVELQKATRYFHPNNLLGEGD
FGNVYRGFVNQDSLEAASPKTGISIAVKKMYQNGCQGQQEWLTEIKYLGQLCHPNLVRLIGYCTQEDHRVLVYE
FMPNGSLDKHLYRKDAREKPLSWDLRMKVALGVAKGVAFLHNEAAQVIYRNLTTSNILLDSDFNVKISDFGLAK
DLPVDDKTHVTTRVLGTIGYTAPEYNQTGHLTIKSDVYSFGVLLLELISGRPAVNPHLPITEQYLVLWAMPFLSNK
RKVFGIFDVCLEGKYVLSGALKAADLALRCLSKTPHTRPTMDDVVKVLEQIISEKY

>A307|LOC108841721

MERDFKSLDPMINSFSLRQIKAATNNFDTANRIGEGGFGPVHKGKLPDGTIIAVKQLSTGSKQGNREFLNEIGMI
SALHHPNLVKLYGCCVEGDQLLLVYEYVENNCLARALFGPQETQLRLDWPTRRKICIGVARGLAYLHEESRIKIVH
RDIKATNVLLDKDMNSKISDFGLAKLNEDDNTHISTRVAGTFGYMAPEYAMRGHLTDKVDVYSFGIVALEIVHG
RSNKINQSLSNYNTPYVIDWVTILREQNNLLELVDPRLGSDYNREEAMTMLQVVILCTSPDPSDRPLMSEVVKM
LEGEKMVELEKLEEASVYRETKRLENMNTMKKYYEMIGSETSMTMTMTITDQTTSSKH

>A308|LOC101503660

MGCFPFFGSSNKNADFVYSTRIFPDRYKFAPNDKSLQNIKAPKFTLEELEAATKNFGPECLLGEGTLGRVFETHLD
SINKVVAVKQLDPNNFKGNREFLVKVLRLSLLHDKNLVNLIGYCADNNQRLLVQEFMPFGSLKSHINKMPLDWN
KRIKIAFEVAMGLDYLHNKTSPPLVHGDLKASNVLLDEDYSVKLSDFGLAKLGPEDENGPNRFTGTIGYCAPEFVIT
GETTMMSDVYSFGVIFLQLISGREAFDLTRPIHERNIVVWAQSLLGNRKNFSKIVDPLLKGYPLRGFHRALAIIGM
CLVKEPSKRPVIGDIVVALTLEFNMKYNDQIDHSDPHYEKTRIVAPHRKMYYD

>A309|LOC100844145

MGCCFSSEKGAMGESKEPAEKTSQIAPAALPSSSGESEMAILEVSKGPHCPLLTYEELNVATEGFRPDHFLGEGG
FGRVYKGVVNGTNQVAIKILNPKGKQGNREFCMEVLILSRLDHPNLVKLVGYCIDGDQRLLVYEYMPLGSLGSHL
HDLSPDQKPLDWNTRMKILAGAAQGLQHLHVKADPPVINRDVKCENILLGEGYHPKLSDFGLAKLGPTGDDTH
VSTRVMGTPGYCAPEYLASGQLTVKSDIYSFGVVMLEVITGRKAIDYCRSRAERNLVEWATPLINRKDFQKLADP
ALGDQYSMKSLFRALTVAQLCVNRTASQRPQITEVAEALAQISQSRSKSVPRSIHLK

>A310|LOC103961642

MPLGFVEDHLLDLPEGQKPLDWFKRMRIALGAAKGLEYLQDEANPPVIYRDLKSSNILLDDDFNAKLSDFGLAKL
GPIGDKTHVSSRVMGTYGYCAPEYQRIGQLTVKSDVYSFGVVLLELITARKAIDTTLSTREQNLVAWAQLVFKDP
NRYPELADPLLQGNFPVRALNQAMAVAAMCLHEEAPVRPLISDIVSALSVLGTGPDTATSPVSSLPSPSPDQTM
VINEDSQLEDSVTERQRAVAEAMEWGIIPSLLPSILPPPSLPPPHTIPSQTQSYSDSQPPSSFPRELRSPTATSTNCS
SPAPTLCNSCFARKEVLKLTKLGGRKSIPCRETKPSTKVFGFVRSVSGGPGRE

>A311|LOC8081452

MTESMVMMEILNNNFITCQCPDRRILLRSFTYDEVCAATHDFEMDCFLGQGGFGQVYKGFLDSTNQVAIKRLD
LQGQQGHREFVTEVLILGNVHHPNLVKLIGYCTSQGQRILVYEYMPLGSLNSHIHDLHPGQQPLDWSTRIKILLG
AAKGLEHLHHNFTPPVINRDVKCANILLGAGYHPKLSDFGLAKLGPTGDDTHVSTRVMGTPGYCAPEYLMTGKL
TVKTDIYSFGVVMLEVLTGRMARDERLPESERNLVAWALNFLRRRELDVLVDPALRGQCSQPCLEHAFFVVSRCI
SETPSMRPNMRDVVASLTVISEVRYRRRLDRGGRSTPTRTSSDRNHQGEDQGEGN

>A312|LOC110904744

MGSCLSAQLKLQTSCHDGKVASATTPPRPLSQDDILRSTNLKSFSFNVLKTATRSFRPDSVLGEGGFGMVFKGWI
DEQNVTAAKPGTRTVIAVKILNQNGLQGHREWLTEINYLGQLKHPNLVQLIGYCIEGDKRLLVYEFVSRGSLENH
LFKRGCRPLSWSQRLKVALGAAKGLAYLHSPEANVIYRDFKCSNILIDYEYNAKLSDFGLAKDGPVDGKSHVTTRV
MGTYGYAAPEYMASGHLSERNDIYSFGVVLLEILTGRPCIDKNRPSNEKKLVDFAKPYLTSRRILQIMDRRIDGQY
ASDVAMRVAALAKKCLAVRPKDRPTANELVEALEQLQEPQKGSCIRRK

>A313|D8S619

ATIDKVKPAASLDRKKENAPGDSTPAIAAQTFTFRELAAATKNFKAECLLGEGGFGRVYKGRLENSGQVVAVKQL
DRNGLQGNREFLVEVLMLSLLHHPNLVNLIGYCADGDQRLLVYEFMPLGCLEDHLHDIPPEKAPLDWNTRMKIA
AGAAMGLEYLHDKANPPVIYRDFKSSNILLDNNFHPKLSDFGLAKLGPVGDKTHVSTRVMGTYGYCAPEYAMT
GQLTLKSDVYSFGVVLLELITGRKAIDNSRPAGEHNLVAWARPLFKDRRKFPSMSDPLLQGRYPMRGLYQALAV
AAMCLQEQAATRPLIADVVTALNYLASQTYDPGVHPLSNSRLTPPSSREKRE

>A314|LOC102662273

MRTATNNFDEVFVGGFGNVYKGHIDNGSTTVAIKRLKQGSRQGIREFKNEIEMLSQLRHPNIVSLIGYCYESNEM
ILVYEFMDCGNLRDHLYDTDNPSLSWKHRLQTCIGVARGLDYLHTGVKQVIIHRDVKSANILLDEKWEAKVSDFG
LARIGGPMGISMMTTRVNTEVKGSIGYLDPEYYKRNILTEKSDVYSFGVMLLEVLSGRHPLLHWEEKQRMSLAN
WAKHCYEKGTLSEIVDSELKGQIKPQCLNKFSEVALSCLLEDGTQRPSMKDIVGVLEFVLQFQDSAVNYEDSSSHS
TVPLSDCSENTGSSMTSDGDRSNGRMESFVLIPDDVFSETKNPKGR

>A315|PAN36407.1

MKRVRRFFNLKDLGRGKEKDTAAGTAVSTGSDDEDTRFTLNVETFSFKELSAATDNFNDYLLIGSGGSAKVYEGR
LPGIGKVAVKRLSFGGVSRHTAAHLQRGFLREVFVLNSINHPNIVRLIGCCSEESERLLVYEYIYWGSMRKCLSELD
WQKRMNTALGAAKGLERLHLQVNPSIIHRDIKSDNILLCMDFEPKVSDFGSAKIAPAGGAGSGQVGTFGYMAP
EVAFCKSISIRSDIYSFGVVLLELITGRKAIDSKRQDEEQHLASWARSKLQGQNNIEELLDPRLPGCAPKFDDLNKA
LAVAWMCTMTDDVDRPEIGEIVQDLRCLADSFSDNKGSLSSAA

>A316|SELMODRAFT_112070

MKSITPTPGKASKLQIAVPAISIAELQAATNSFSQENLVGEGALGRVYRAEIDDKIVAVKKLDTSAPMVQNEDEFI
KVVSNLARLRHSNITELVGYCTEHSQRLLVYDFVEYGTLFEVLHCSDESSRRLSWNQRVKIALGAARALEYLHEVY

HPAIVHRNFKSVNILLDEELNPRVSDCGLAALAPYGAERQVSSQMLVSFGYSAPEFAMSGVYTVKSDVYSFGVV
MLELLTGRKSLDSSRSRAEQSLVRWAVPQLHDIDALSRMVDPALKGIYPAKSLSRFADIISSCVQPEPEFRPPMSE
VVQALVRLMQRASLSKRRLTADDLGSSQRSLDRYEPSDSSA

>A317|PAN06184.1

MGCFSCFDSPADEQLNPKLGGAGGYGGASSAPKDANGNVISAQTFTFRELATATRNFRPECMLGKGGFGRVYR
GHLESTGQVVAIKRLNRYGLQGNREFLVEVFMLRVLHHQNLVNLIGYCADGDQRLLVYEYMPFGSLEDHLHDLP
LDKEALDWNTRMKIAAGAAKGLEYLHDKAKPPVIHRDFKSSNILLDERFHPKLSDFGLAKVGPVGDRSHVSTRV
MGTYGYCAPEYAMTGQLTVKSDVYSFGVVLLELITGRRAIDSTRPHGEQNLVLWAGPLFNDRRKLPKMADPRL
EGRYPMRGLYKALEVASMCLQSEAASRPLIADVVTALSYFTPK

>A318|LOC110115010

MGGNCSGSVYQQNTPKIFTFTEVCSATKKFRPDLLVDEQCFAKIYKGTLHLNNQLVAVKQFKPTEDKEQNHKGF
LVEVLMLSKLRHENIINLLGYCQVENQRLLVLEKAALHSLKNYLFSRSSSAKSKSYFKPLDWTARIKIALGVAKGLEY
LHNKVSPPVIHRDLQTSTIMLGEDLTPKISNFELAKLGPMGDRKHIMTSVVGTAKFYAPEYVKQGLVSVKLDVYN
FGIVLLELITGRRVSELICIDTGHQLASWVEAVAKDTKKSIDMADPELNGEFQIQEVSKMLQLAAMCLREERDHR
PLTTEVVAVLSSLLDSSAVEIQIQPQVAKKLLK

>A319|M1AFW9

MLSLLHHPNLVSLIGYCADGDQRLLVYEFMPLGSLEDHLHDLPPDKEPLDWNTRMTIASGAAKGLEHLHDKANP
PVIYRDFKSSNILLKDNFFPKLSDFGLAKLGPTGDKSHVSTRVMGTYGYCAPEYAMTGQLTVKSDVYSFGVVFLEL
ITGRKAIDSTKPQGEQNLVAWARPLFNDRRKFAKLADPSLQGQFPMRGLYQALAVASMCIQEQAAGRPLIGDV
VTALSYLANQSYDPGTVPGQIHRFGADSVDRRNKDDRVGRIFRNEDGAGGGSGQKWDVDGGSEKEDSPRETA
RMLNRDLDRERAVAEAKMWGENWRDKRRQNGQGSFDGGNE

>A320|A0A2G5E5H6

MLSLLHHPNLVNLIGYCADGDQRLLVYEFMPLGSLEDHLHDLPPDKEPLDWNTRMKIAAGAAKGLEYLHDKAN
PPVIYRDFKSSNILLGDGFHPKLSDFGLAKLGPTGDKSHVSTRVMGTYGYCAPEYAMTGQLTVKSDVYSFGVVFL
ELITGRKAIDSTQPHGEQNLVVWARPMFNDRRKFPKLADPRLQGRYPMRGLYQALAVASMCIQEQSATRPLIG
DVVTALSYLANQSYDPSTAPGYRGLGDKDDRRRSGGVDEKGIRIVKNEEGGGSGRKWDLEGSEKDDSPKETAR
MLNRALDREKAVAEAKMWGENWRAQRRQSAQGSFDGTNG

>A321|LOC106804320

MAGDAAAAPASGNIAGSSSRARAFTYGELAAATDNFQAECLLGEGGFGRVYRGRLESGQVVAVKQLDREGAQ
GNREFVVEVMMLSLLHHPNLVNLVGYCADGEQRLLVYEYMALGSLADHLLLMPGGGSPGGDERRALSWETR
MRVALGAARGLEYLHETANPPVIYRDLKSSNVLLDDALCPKLSDFGLAKLGPVGDRSPRVMGTYGYCAPEYVRA
GNLTVKTDVYSFGVLLLELVTGRRAIDSSRPPAEQLLVAWARPMLRDGKRYRELADPLLRGAFPERDLKQAVAVA
AMCLQDEASARPLMSDAAVTLEYLAEAAAAADAAPPSTS

>A322|100193149

MERILRCFNLGPTGSEDDDISFTLTVETFSFEELSAATDNFSSTLRIGSGGQATVYEGHIPHIGQVAVKRLRTNDFP
SKQRAFLMEVYVLNSTNHPHIVKLLGCCSEGTERLLVYEYVKGGTLRHRLKELNWYTRMRIARDTATVLEKLHLQ

SDPPIIHRDFKSDNILLSTNLSPKVSDFGIAKIAPAGDELTAATSETLGTYGYASPGIFRPLNATVMTDIYSFGVVLLE
IITGKPAIDHSKEGDEHYLVHWAASKSKDGNFEEVLDPRLRGSVSRMVLSEALAVVDRCTGTEPVGRPRIREIIRA
LRHVVSISQDDTPGASSSAAS

>A323|Niben101Scf05202g00005.1

MKIAAGAAKGLEYLHDKANPPVIYRDLKSSNILLDEGYHPKLSDFGLAKLGPVGDKTHVSTRVMGTYGYCAPEYA
MTGQLTLKSDVYSFGVVFLELITGRKAIDNARSHGEHNLVAWARPLFKDRRKFPKMADPLLQGRYPMRGLYQA
LAVAAMCLQEQASTRPLIGDVVTALSYLASQTYDPNAVGAQSNRVGSSTPRSREDRLHSADGVDSPDEHSSAH
HGSPSIQRNSPDSRKRDYARDFNTGIELRKIATSGGSGRKWGLDESERPDSQKNSPVSAGRSRETPRNRDLDRER
AVAEAKVWGENWRDKKKTSARGNSFDGMNE

>A324|OEL36841.1

MAGEGGDAAVASSSSRARAFTYGELAAATDNFRAECLLGEGGFGRVYRGRLESGQVVAVKQLDRDGAQGNRE
FVVEVLMLSLLHHPNLVNLVGYCADGEQRLLVYEYMALGSLADHLLLDCSKAPGPGAEQEQRALSWETRMRVA
LGAARGLEYLHETANPPVIYRDLKSSNVLLDDALCPKLSDFGLAKLGPVGDRSPRVMGTYGYCAPEYVRAGTLTV
KTDVYSFGVLLLELVTGRRAVDSSRPPAEQLLVAWARPMLRDGKRYRELADPLLRGDFPERDLKQAVAVAAMCL
QDEASARPLMSDAAMTLAYLAEAAALAAPASS

>A325|A0A4D9ADT7

MLSLLHNPHLVNLLGYCADGDQRLLVYEFMPLGSLEDHLHDLPPDKEPLDWSTRMKIAAGAAKGLEHLHDKAN
PPVIYRDFKSSNILLGEGFVPKLSDFGLAKLGPTGDKSHVSTRVMGTYGYCAPEYAMTGQLTVKSDVYSFGVVFLE
IITGRKAIDSTRPQGEQNLVAWVRPFFNDRKRYVKLADPRLQGKFPIRGLYQALAVASMCTQEQAAARPLIGDV
VTALSYLANHSYDPSHSNREERGGKLLRNEEGAGSGRKWDVEGGSERDDSPRETAKMLNRDLERERAVAEAK
MWGENWREKRRQSAQGGSFDANNS

>A326|EMS58714.1

MGFLSCLFRCPEEEERVVKEHDDDEDSGGIDHGVASESSESAPLKTESAHMEGIQRNGTHNEATIFTLRELVDAT
KNFSKDFQLGRGGFGCVYKAYLNDGQVVAVKQLDLNGLQGNREFLVEVLMLNLLHHPNLVNLIGYCVDGDQR
LLVYEYMPLGSLEDHLHDLPPNKEPLDWTTRMKIAAGAAAGLEYLHDKANPPVIYRDIKPSNILLSEGYHAKLSDF
GLAKLGPVGDKTHVTTRVMGTYGYCAPEYAATGQLTVKSDIYSFGVVFLELITGRRALDSNRPREEQDLVSWVG
FDNLTVQLTGPIIMIL

>A327|103996684

MGCFPCFESEEEAQLSHGNECDRTREERPMVAPRVEKLSSGDDRMKTARDLSTKRESLGAIEGSDFSISAQTFTF
SELSAATSNFRTESLLGEGGFGRVYKGRLETTGQVVAVKQLDRNGLQGNREFLVEVLMLSLLHHPNLVNLIGYCA
DGDQRLLVYEYMPLGSLEDHLHDFPPDKEPLDWNTRMKIAAGAAKGLEYLHDKANPPVIYRDLKSSNILLDKGF
HPKLSDFGLAKLGPVGDKSHVSTRVMGTYGYCAPEYAMTGQLTVKSDVYSFGVVLLELITGRKAVESTKSHSEQ
NLVSWQSGI

>A328|101307507

MLFCEAQPEGHIFETSNSRVFSYEELKTATRNFRQEGLLGEESFGKVFKGWLDEKTLVPSNAGTRMIIAVKKWNP
DSFQGLKEWQAEVDFLGRLSHPNIVKLLGHCSEDKQLPLVYEFMPKGSLVYEFMPKRSLADHLFRTQLSFPGAVP

SWDNRLKIAIGVARALSFLHSLQKQIILRDFKASNILLDENYNAKLSGFGLAMLGPSDAELYVETEVMGTYGYAAP
EYVATGCLYVKSDVYSFGVVLLEMLTCLKALDMNRSSNKHNLVDWAKPLLSHKRTLETLIDAKIKGQYSSRAWPD
LT

>A329|LOC110731912

MDERSFGPVYQGKLNDTGEIVAVELWDRKIQHESQEHKEFLRQLKILSFLNHPNVITLIGYCIEGDQRILVCEYLSL
TSSLYQHLHGRLSKQKPPLDWNTRMKIALGIARGLECIHAASPPIIYVDLKPCNIFIDDDFNAKLSDFWLSKLEPYD
DDEYTCTTLIGIASGGYHAPEYIATGHLTVKADVYSFGVVLLELVTGRKAKDISRPTKEQDLISWAQQKIKNPNKFP
ELADPLLQGNFPANGFSQALVLAVKCLRLKESTRPMMSNVVADIMCLTSDELNDKRLV

>A330|OEL29886.1

MKIAAGAAKGLEYLHDKANPPVIYRDFKSSNILLGEDYYPKLSDFGLAKLGPVGDKTHVSTRVMGTYGYCAPEYA
MTGQLTVKSDVYSFGVVFLELITGRKAIDHTQPSGEQNLVAWARPLFRDRRKFCQLADPLLQGRYPKRGLYQAL
AVAAMCLQEQAASRPLIGDVVTALSYLASHPYDPNAPTIKDSRTCPSTPRAKTHRRTTSVPDAQHAAESLMLNF
PDLRKDTIRGGEFEQDRTEGSGSSSSSGRNDSLDVPQLLAVPNGKAYSEADSIQKSTVKVGAREKK

>A331|GAV92667.1

MKIAAGAARGLEYLHDKANPPVIYRDFKSSNILLDEGFLPKLSDFGLAKLGPTGDKSHVSTRVMGTYGYCAPEYA
MTGQLTVKSDVYSFGVVFLELITGRKAIDSTQPHGEQNLVAWARPLFNDRRKFSRLADPQLRGRYPMRGLYQA
LAVASMCIQEQAATRPLIGDVVTALSYLANQAYDPNSPGHAHRSSGDKQDKRNRDDRGGKLLKNDGGGGSGL
RWDLESSEKDDSPKETARMLNRERAVAEAKMWGENWREKRRQTAQESFDGTNG

>A332|LOC109807025

MGLCFSSGSSLRRPQTQYSGSATTSSAGKSQFSEIASGSIDSQGSLPLALPLPSPDGQILERPNLKVFTFGDLRSAT
KSFKSDTLLGEGGFGRVYKGWLDEKTLSPAKAGFGMIVAIKKLNPQSTQGFQEWQSEVNFLGRLSHPNLVKLLG
YCWDDDELLLVYEFMSKGSLENHLFRRNPNIEPLSWSTRFKIAIGAARGLAFLHASEKQVIYRDFKASNILLDGNY
NAKISDFGLAKLGPSGGQSHVTTRVMGTYVYAAPEYIVTGK

>A333|LOC104737970

MGCFSCFDSSDDEKLNPVEESNGQKKQLQPTVSNSISGLPSGGEKLSSKSNGGSKRELLLPRDGLNQIAAHTFAF
RELAAATMNFHPDTFLGEGGFGRVYKGRLDSTGQVVAVKQLDRNGLQGNREFLVEVLMLSLLHHPNLVNLIGY
CADGDQRLLVYEFMPLGSLEDHLHDLPPDKEALDWNMRMKIAAGAAKGLEFLHDKANPPVIYRDFKSSNILLDE
GFHPKLSDFGLAKLGPTGDKSHVSTRVMGTYGYCAPEYAMTG

>A334|LOC107484697

MIVAVKRLNLDGLQGHKEWLAEINYLGQLQHPNLVKLIGYCLEDDHRLLVYEFMPKGSVENHLFRRGSYFQPFS
WNLRMKIALGAARGLAFLHSPEVQVIYRDFKTANILLDTNYNAKLSDFGLARDGPTGDRSHVSTRVMGTRGYA
APEYLATGHLTSKSDVYSFGVVLLEMISGRKAIDKNQPTGEHNLVEWAKPYLSNKRRVFRVMDPRLEGQYSQSR
AQAAAALAMQCLAVEPKARPNMDEVVKXIGAASGIKRLAQKKH

>A335|PKI74639.1

MLSLLHHPNLVNLIGYCADGDQRLLVYEYMPLGSLEDHLHDLPPDKKRLDWNTRMKIAAGAAKGLEYLHDKAN
PPVIYRDLKCSNILLDEDYHPKLSDFGLAKLGPVGDKTHVSTRVMGTYGYCAPEYAMTGQLTLKSDVYSFGVVLLE
IITGRKAIDNLKSAGEHNLVAWARPLFKDRRKFSQMADPTLQGQYPVRGLYQALAIAAMCVQEQPNMRPVIAD
VVTALSYLASQKYEPSSRSASSTPRARREQWPAN

>A336|LOC103956522

MIVAVKGLNQEHLPGHKEWLAEINYLGQLHHENLVRLIGYCLEDDHRLLVYEYMPHGSLDRHLFSKSSTSQPLP
WSLRMRIALGAAKGLAFLNSDLQMICYDFKTSNILLDSMNNAKLTNFDLARTRVMGRRYGYAAPEYIATGQLTA
KSNVYSFGVVLLEMVSGRPVVDMNRPSGERNLVEWAKPYLASKRKVFKIFDARFEGQYPSSDAREVVKLAIQCL
ADNPKSRPNMNDVVKTLEQLQKSEPSLSAG

>A337|PNX94680.1

MSCFPCCKSEVESPSFRGCSKKVSKGRRTFKSLAAAMSLKTAVKTLNREGNQGTREFFAEVLMLSMVNHPNLVK
LLGYCVEDDHRVLVYEYMANGSLENHLLDLDKDKEPLDWETRMKIADGAARGLEYLHNSADPPVIFRDFKSSNIL
LDENFNAKLSDFGLAKIAPKEGENLVTTRVMGTYGYCSPEYAATGKLTSKTDIYSFGVVFLEIITGRRVIDTAREPEE
RNLIDW

>A338|LOC110824639

MLSLLHHPNLVNLIGYCADGDQRLLVYEYMRLGSLEDHLHDLPPDKKQLDWNTRMKIAAGAAKGLEYLHDKAN
PPVIYRDLKCSNILLAEGYHPKLSDFGLAKLGPVGDKTHVSTRVMGTYGYCAPEYAMTGQLTLKSDVYSFGVVLLE
IITGRKAIENSRGAGEHNLVAWVRHYLLQFDVSFAFVKIIARDFRHNTRRANLIDFLSVMSYQNQRAECQISFLL

>A339|LOC111298251

MQNHGFHPKLSDFGLAKLGPVGDKSHVSTRVMGTYGYCAPEYAMTGQLTVKSDVYSFGVVFLELITGRKAFDS
TQPHGEQNLVTWARPLFNDRRKFSKLADPRLQGRYPMRGLYQALDVASLCIQEQAAARPLIGDVVTALSYLAN
QAYDPNAAGHGYRGSGDKDDKRCRDDRGGRASRNDGASGCRWDLEGSEKDDSPRETARILNRDLE

>A340|LOC110896597

MPLGSLENHLFDLELDQEPLDWHTRLKIAVGAARGLEYLHCKANPPVIYRDLKSSNILLDNDFNPKLSDFGMAKL
GPVGDNTHVSTRVIGTYGYCAPDYAMSGKLTIKSDIYSFGVVLLELVTGRKAIDKTKKPGEQNLVSWARPFLRDR
KKYVQLVDPLLPGRFSMRTVHHMVAITAMCLQDEANFRPLISDIVVGLEYLASQAEMCK

>A341|GAV92589.1

MLSLLHHPNLVNLIGYCADGDQRLLVYEFMPLGSLEDHLHDLPPDREPLDWNTRMKIAAGAAKGLEYLHDKAN
PPVIYRDLKSSNILLDEGYHPKLSNFGLAKLGPVGDKTHVSTRVMGTYGYCAPEYAMTGQLTLKSDVYSFGVVFL
ELITGRKAIDNARTHGEHNLVAWMTFSCSQRLTFPP

>A342|LOC110034372

MPLGSLEDHLHGLAPNKEPLDWNARMKIAAGAAKGLEYLHDKANPPVIYRDFKSSNILLGEGFHPKLSDFGLAKL
GPVGDKTHVSTRVMGTYGYCAPEYAMTGQLTLKSDVYSFGVVFLELITGRKAIDNTRASGEQNLVAWVGPLLH
NCFHLRHLHTYHQILICLHI

>A343|AFG44754.1

EFRVEVEAIGRVRHKNLVRLLGYCAEGAGRMLVYEFVDNGNLEQWLHGDVGPVSPLTWEIRMKIILGTAKGLAY
LHEGLEPKVVHRDVKSSNILLDKQWNAKVSDFGLAKLLGSEKSYVTTRVMGTFG

>A344|ACH59260.1

HLHHRNLVPLLGYCVAKNEKLLVYRHMANGSLYERLHAHEIEDDNYLDWTRRLKIAIGAARGLAWLHHSCNPRII
HRNVSSNCILLDENHEAKITDFGLARLMNPVDTHLSTFINGDFGDLGY

>A345|A0A067GTS8

MGCFPCFDSREDEKLNPEKEPDDQKQGQPTVSNNISRLPSGRDRLRSRSNGVSKRELHLPKDGAGVNIAAQTFT
FRELAAATKNFRPESFIGEGGFGRVYKGWLESTGQVVAVKQLDRNGLQGNREFLVEVLMLSLLHHSNLVNLIGY
CADGDQRLLVYEFMPLGSLEDHLHDLPPDKEALDWNTRMKIAAGAAKGLEYLHDKANPPVIYRDFKSSNILLEE
GFHPKLSDFGLAKLGPVGDKSHVSTRVMGTYGYCAPEYAMTGQLTVKSDVYSFGVVFLELITGRKAIDSTRPHGE
QNLVTWARPLFNDRRKFSKLADPRLEGRYPIRGLYQALAVASMCIQEQAATRPLIGDVVTALSYLANQTYDPNS
HRGAGDKDDRRNRDGARIFKNDEGGGSGRRWDLEGSEKEDSPRETARILNRDLERERAVAEAKMWGENLREK
RRQSAQGSFDGTNG

>A346|A0A368PL81

MGCFSCFDSPADEQLNPKLGGAGGYGGSSSAAAAYGAGSGGGRHGDRGYPDLQQAPMVAPRVEKLCTAAEK
ARVKSNALAREASAPKDANGNIISAQTFTFRELATATRNFRPECFLGEGGFGRVYRGRLESTGQVVAIKQLNRDG
LQGNREFLVEVLMLSLLHHQNLVSLIGYCADGDQRLLVYEYMPFGSLEDHLHDLPLDKEALDWNTRMKIAAGA
AKGLEYLHDKANPPVIYRDFKSSNILLDEGFHPKLSDFGLAKLGPVGDKSHVSTRVMGTYGYCAPEYAMTGQLTV
KSDVYSFGVVLLELITGRRAIDSTRPHGEQNLVSWARPLFNDRRKLPKMADPRLEGRYPMRGLYQALAVASMCI
QSEAASRPLIADVVTALSYLASQSYDPNAALASRKPGGDPRSKPSENGRVVSRNDETSSSGHKSPGKDREDSPRD
LPGILNKDFDRERMVAEAKMWGDRERMVAEAKMWGDRERMVAEAKMWGDRERMVAEAKMWGENWR
DKRRTENGQGSLDSPTGNI

>A347|A0A3B5ZVP1

MGCLPCFGSSGKGEPAKKGGARKDVPSDRRATGVGSDKPKPQGLLDSKKDTVIPREGNNQHIAAHTFTFRELA
AATKNFRQDCLLGEGGFGRVYRGRLDNGQAVAVKQLDRNGLQGNREFLVEVLMLSLLHHDNLVNLIGYCADG
DQRLLVYEYMPLGSLEDHLHDIPPEKEPLDWNTRMKIAAGAAKGLEYLHDKASPPVIYRDFKSSNILLGEEFHPKL
SDFGLAKLGPVGDKTHVSTRVMGTYGYCAPEYAMTGQLTVKSDVYSFGVVFLELITGRKAIDNTKPHGEQNLVA
WARPLFKDRRKFPKMADPALQGRFPMRGLYQALAVAAMCLQEQAATRPFIGDVVTALSYLASQAYDPNAPTQ
HSRSNASTPRARDRGSVNGDQRRIRSPNHHSPDLRRKEVTTTSKYEAEVSRNSSGGGSGRRSGLDDRDVTGSQ
QGSPAQAGKRRETSRTSERQRAIAEAKTWGENSRERKWPNARGSFDSTNE

>A348|A0A3B6C6Q2

MGLREKTGIRCGAEKARAKGNAGMKELSDLRDANGNVLSAQTFTFRQLTAATRNFREECFIGEGGFGRVYKGR
LDGGQVVAIKQLNRDGNQGNKEFLVEVLMLSLLHHQNLVNLVGYCADGEQRLLVYEYMPLGSLEDHLHDLPPD
KEPLDWNTRMKIAAGAAKGLEYLHDKAQPPVIYRDFKSSNILLGDDFHPKLSDFGLAKLGPVGDKSHVSTRVMG
TYGYCAPEYAMTGQLTVKSDVYSFGVVLLELITGRKAIDSTRPHGEQNLVSWARPLFNDRRKLPKMADPGLQGR
YPMRGLYQALAVASMCIQSEAASRPLIADVVTALSYLASQIYDPNAIHASKKAGGDQRSRVSDSGRTLLKNDEAG
SSGHKSDRDDSPREPPPGILNDRERMVAEAKMWGANLREKTRAAANAQGSLDSPTETG

>A349|A0A1U8E6G8

MGGCFPCFGSSNKETGKQVVKKESFKDASAPQSIHLSKVNSDKSKSRGSHDPKKDPAIPKDGPTAHIAAQTFTFR
ELAAATKNFRPECLLGEGGFGRVYKGRLESTGQVVAVKQLDRNGLQGNREFLVEVLMLSLLHHPNLVNLIGYCA
DGDQRLLVYEFMPLGSLEDHLHDLPPDKGPLDWNTRMKIAAGAAKGLEYLHDKANPPVIYRDLKSSNILLDEGY
HPKLSDFGLAKLGPVGDKTHVSTRVMGTYGYCAPEYAMTGQLTLKSDVYSFGVVFLELITGRKAIDNTRSHGEH
NLVAWARPLFKDRRKFPKMADPLLQGRYPMRGLYQALAVAAMCLQEQASTRPLIGDVVTALTYLASQTYDPN
AAGAQSNRVGSSTPRSREDRLHSVDGVDSPEHCSAHHGSPSIQRNSPDSRKRDYARDFNTGIELRKIATSGGSSR
KWGLDESERPDSQRNSPVSAGRTRETPRNRDLDRERAVAEAKVWGENWRDKKKTNGRGSSFDGIND

>A350|A0A0D2Q6X1

MGCFPCFDSKEEEKLNTVNESNDPKQAQPTVSSNISSLSSGGDRLRSRSNGGSKRELPCPRDGPGVQIAAHIFSF
RELAAATKNFRPESFLGEGGFGHVYKGQLESTGQVVAVKQLDRNGLQGNREFLVEVLMLSLLHHPNLVNLIGYC
ADGDQRLLVYEFMPLGSLEDHLHDLPPGKEPLDWNTRMKIAAGAAKGLEYLHDKANPPVIYRDFKSSNILLEEGF
HPKLSDFGLAKLGPVGDKSHVSTRVMGTYGYCAPEYAMTGQLTVKSDVYSFGVVFLELITGRKAIDSTRPHGEQ
NLITWARPLFTNRRKLSKLADPLLQGRFPMRGLYQALAVASMCIQEEAAARPHIGDVVTALSYLANQAYDPNAS
GHGHSGSGETDEKRYRDDRGGRVSKNDEGGVSGCRWDLEGSEKDDSPKETARMLNRDLDRERAVAEAKMW
GENWREKRRQSAQGSSDGSNG

>A351|A0A397XMK6

MGCFSCFDSSDDEKLNPVDESKSQKQSQPTLSNNNISGLPSGGEKLSSKSNGGSRRELLLPRDGLSQISAHTFSFH
EVAAATMNFHPDTFLGEGGFGRVYKGRLDSTGQVVAVKQLDRNGLQGNREFLVEVLMLSLLHHPNLVNLIGYC
ADGDQRLLVYEFMPLGSLEDHLHDLPPDKEALDWNMRMKIAAGAAKGLEFLHDKANPPVIYRDFKSSNILLDEG
FHPKLSDFGLAKLGPTGDKSHVSTRVMGTYGYCAPEYAMTGQLTVKSDVYSFGVVFLELITGRKAIDTDMPHGE
QNLVAWARPLFNDRRKFIKLADPRLKGRFPTRALYQALAVASMCIQEQAATRPLIADVVTALSYLANQAYDPNK
DDSRRNRDEKGGRLITRNDEGGGSGSKFDLEGSEKEDSPRETTRILNRDINRERAVAEAKMWGESLREKRRQSE
QGTSESNSTG

>A352|LOC112282882

MGCFPCFDSKPKERKPLKRDDNNSSRDGQSAAANPVAQISKLPSGNSKKYDAKGSFDKKEPQREGSTHIAAQTF
TFRELAAATKNFRPECLLGEGGFGRVYKGRLENTGQVVAVKQLDRNGLQGNREFLVEVLMLSLLHHPNLVSLIGY
CADGDQRLLVYEFMPLGCLEDHLHDLPQDKECLDWNTRMKIAAGAARGLEYLHDKANPPVIYRDFKSSNILLDE
GFHPKLSDFGLAKLGPVGDKTHVSTRVMGTYGYCAPEYAMTGQLTLKSDVYSFGVVLLELITGRKAIDNSRAAGE
HNLVAWARPLFKDRRKFPSMADPMLQGRYPMRGLYQALAVAAMCLQEQAATRPLIGDVVTALSYLASQIYDP
GVHPLGSSRFAPATPSREKREKEKKMPPGPNAIEERMVKEQRAGKAGPRSPSVRSQAASPDLRVKEGGARARV
TNGVLEESRGSESGSGRKRDSEEWERHEGGGRDSPGYAGRTGRDGLKPQAPTRERERAVAEARVWGENWRE
RKRGTA

>A353|100283091

MGCFPCFDSPADEQLNPKFGGAGGYGGTSSAVAAYGNGTGAGVSIGRHGDRGYPDLQQAPMAAPRVEKLSA
AAEKARVKSNGLTKEALVPKDANGNAISAQTFTFRELATATRNFRPECFLGEGGFGRVYKGRLESTGQVVAIKQL
NRDGLQGNREFLVEVLMLSLLHHQNLVNLIGYCADGDQRLLVYEYMPSGSLEDHLHDLPLDKEALDWNTRMKI
AAGAAKGLEYLHDKANPPVIYRDFKSSNILLDESFHPKLSDFGLAKLGPVGDKSHVSTRVMGTYGYCAPEYAMTG
QLTVKSDVYSFGVVLLELITGRRAIDSTRPHGEQNLVSWARPLFNDRRKLPKMADPRLEGRYPMRGLYQALAVA
SMCIQSEAASRPLIADVVTALSYLASQPYDPNAALASRKPGGDQRSKPGENGRAVSRNDETGSSGHKTPAKDRE

DSPRDLPAILNKDLDRERMVAEAKMWGDRERMVAEAKMWGDRERMVAEAKMWGENWRDKRRAEIGQGS
LDSVTGNS

>A354|A0A0D2UVL2

MGGCFPCFGSSNKAGSNGGGSVKELSKKDSTKDSSVGQPHHVNRVNSDKAKSRSVSDPKKEPAVPKDGPTANI
AAQTFTFRELAAATKNFRPECLLGEGGFGRVYKGHLESTGQVVAVKQLDRNGLQGNREFLVEVLMLSLLHHPNL
VNLIGYCADGDQRLLVYEFMPLGSLEDHLHDLPSDKEPLDWNTRMRIAAGAAKGLEYLHDKANPPVIYRDLKSS
NILLDEGFHPKLSDFGLAKLGPVGDKTHVSTRVMGTYGYCAPEYAMTGQLTLKSDVYSFGVVFLELITGRKAIDN
TRAPGEQNLVAWARPLFRDRRKFPKMADPLLQGRYPIRGLYQALAVAAMCLQEQAATRPLIGDVVTALTYLAS
QTYDPNAPGNQSNRVGPSTPRVKDDRRSMADGLDSPDARGRVGSPSTHRNSPDYRKKNHVREMSSGAELSR
NEPGEGSGRKWGSLDESEQQESHTDSPMNSARARETSRNRDLDRERAVAEAKVWGETWREKKRANAMGGS
NGRND

>A355|A0A0L9VER5

MGGCFPCFGSSNKEDSGGVRVKEVPNKDSSFKEAASLVPQSHHPSRANTDKSKSSRSGADAKKEAPVPKDGPT
AHIAAQTFTFRELAAATKNFRPECLLGEGGFGRVYKGRLESTGQVVAVKQLDRNGLQGNREFLVEVLMLSLLHH
PNLVNLIGYCADGDQRLLVYEFMPLGSLEDHLHDLPPDKEPLDWNTRMKIAAGAAKGLEYLHDKANPPVIYRDL
KSSNILLDEGYHPKLSDFGLAKLGPVGDKTHVSTRVMGTYGYCAPEYAMTGQLTLKSDVYSFGVVFLELITGRKAI
DNTRAHGEHNLVAWARPLFKDRRKFPKMADPLLQGRYPMRGLYQALAVAAMCLQEQAATRPLIGDVVTALTY
LASQTYDPNAANQSNRVGPSTPRMRDDRRSLADGVDSPDRRLGSPSTHRNSPDFRKRDSRDASIGTELGSRMD
TGGGSGRKWGLDDYERQESQRDSPVNTARARETPWNRDLDRERAVAEAKVWGENWREKKKANAMGSFDA
TND

>A356|A0A1S3XBK3

MGGCFPCFRSSHKESGNGVKEVVKKESFKDGSAAQSIHLSKVNSDKSKSRGSHDSKKDPAIPKDGPTAHIAAQTF
TFRELAAATKNFRPECLLGEGGFGRVYKGRLESTGQVVAVKQLDRNGLQGNREFLVEVLMLSLLHHPNLVNLIGY
CADGDQRLLVYEFMPLGSLEDHLHDLPPDKEPLDWNTRMKIAAGAAKGLEYLHDKANPPVIYRDLKSSNILLDE
GYHPKLSDFGLAKLGPVGDKTHVSTRVMGTYGYCAPEYAMTGQLTLKSDVYSFGVVFLELITGRKAIDNARSHG
EHNLVAWARPLFKDRRKFPKMADPLLQGRYPMRGLYQALAVAAMCLQEQASTRPLIGDVVTALSYLASQTYDP
NAVGAQSNRVGSSTPRSREDRLHSADGVESPDEHSSAHHGSPSIQRNSPDSRKRDSARDFNTGIELRKIATSGGS
GRKWGLDESERPDSQKNSPVSAGRTRETPRNRDLDRERAVAEAKVWGENWRDKKKTSARGSSFDGMND

>A357|C5XSN6

MGCFSCFDSPADEQLNPKFGGAGGYGGGTSAAAAAYGAGAGAGVGRHGGRGGYPDLQQAPMAAPRVEKFS
AAAEKARVKSNVLTKEASVPKDANGNAISAQTFTFRELATATRNFRPECFLGEGGFGRVYKGRLESTGQVVAIKQ
LNRDGLQGNREFLVEVLMLSLLHHQNLVNLIGYCADGDQRLLVYEYMPSGSLEDHLHDLPLDKEALDWNTRMK
IAAGAAKGLEYLHDKANPPVIYRDFKSSNILLDESFHPKLSDFGLAKLGPVGDKSHVSTRVMGTYGYCAPEYAMT
GQLTVKSDVYSFGVVLLELITGRRAIDSTRPHGEQNLVSWARPLFNDRRKLPKMADPRLEGRYPMRGLYQALAV
ASMCIQSEAASRPLIADVVTALSYLASQQYDPNTALASRKPGGDQRSRPGENGRVVSRNDETGSSGHKSPGKDR
EDSPRDLPAILNKDLERERMVAEAKMWGDRERMVAEAKMWGDRERMVAEAKMWGENWRDKRRAENGQ
GSLD

>A358|A0A0D3G6V3

MGCFPCFGSGGKGEAKKGGGGRKDGGSADRRVARVGSDKSKSQGGLDSRKDAFIPRDANGQPIAAHTFTFRE
LAAATKNFRQDCLLGEGGFGRVYKGHLENGQAVAVKQLDRNGLQGNREFLVEVLMLSLLHHDNLVNLIGYCAD
GDQRLLVYEFMPLGSLEDHLHDIPPDKEPLDWNTRMKIAAGAAKGLEFLHDKANPPVIYRDFKSSNILLGEGYHP
KLSDFGLAKLGPVGDKTHVSTRVMGTYGYCAPEYAMTGQLTVKSDVYSFGVVFLELITGRKAIDNTKPLGEQNLV
AWARPLFKDRRKFPKMADPLLAGRFPMRGLYQALAVAAMCLQEQAATRPFIGDVVTALSYLASQTYDPNAPV
QHSRSNASTPRARNRVGANFDQRRLHSPNHQQSPDLRKEGTTTSKYEAEVSRTNSGSGSGRRAGLDSMDVTG
SQMGSPAHAGRKRESSRSTDRQRAVAEAKTWGENSRERKWPNARGSFDSTNE

>A359|A0A0E0N7C7

MSCFLCFGSAQEGEAKKPGADSKDARKDGSADRGVSRVGSDKSRSHGGLDSKKDVVIQRDGNNQNIAAQTFT
FRELAAATKNFRQDCLLGEGGFGRVYKGRLETGQAVAVKQLDRNGLQGNREFLVEVLMLSLLHHTNLVNLIGYC
ADGDQRLLVYEFMPLGSLEDHLHDLPPDKEPLDWNTRMKIAAGAAKGLEYLHDKASPPVIYRDFKSSNILLGEGF
HPKLSDFGLAKLGPVGDKTHVSTRVMGTYGYCAPEYAMTGQLTVKSDVYSFGVVFLELITGRKAIDNTKPQGEQ
NLVAWARPLFKDRRKFPKMADPMLQGRFPMRGLYQALAVAAMCLQEQATTRPHIGDVVTALSYLASQTYDP
NAPVQHSRSNSSTPRARNLAGWNEDRRSVRSPNHHSPDLRREAARSSRAEVSRTSSTGDSGRRSGLDDLDMT
GSQMGSPAQTGRKRETPRTADRQRAIAEAKTWGENSRERKHPNGHGSFDSTNE

>A360|A0A0E0PLM1

MGCFPCFGSGGKGEAKKGGGGRKDGGSADRRVARVGSDKSKSQGGLDSRKDAFIPRDANGQPIAAHTFTFRE
LAAATKNFRQDCLLGEGGFGRVYKGHLENGQAVAVKQLDRNGLQGNREFLVEVLMLSLLHHDNLVNLIGYCAD
GDQRLLVYEFMPLGSLEDHLHDIPPDKEPLDWNTRMKIAAGAAKGLEFLHDKANPPVIYRDFKSSNILLGEGYHP
KLSDFGLAKLGPVGDKTHVSTRVMGTYGYCAPEYAMTGQLTVKSDVYSFGVVFLELITGRKAIDNTKPLGEQNLV
AWARPLFKDRRKFPKMADPLLAGRFPMRGLYQALAVAAMCLQEQAATRPFIGDVVTALSYLASQTYDPNTPV
QHSRSNASTPRARNRVGANFDQRRLHSPNHQQSPDLRKEGTTTSKYEAEVSRTNSGSGSGRRAGLDSMDVTG
SQMGSPAHAGRKRESSRSTDRQRAVAEAKTWGENSRERKWPNARGSFDSTNE

>A361|A0A3B6H4U8

MSCFSCFGPALEAEGRKPVPDAKDPRAKDGAVSDRAGSDKLRLQGGSDPKNNHLTIPRDGSSQNIAAQIFTFRE
LAAATKNFRQDCMLGEGGFGRVYKGRLESGQAVAVKQLDRNGLQGNREFLVEVLMLSLLHHTNLVNLIGYCAD
GDQRLLVYEFMPLGSLEDHLHDVPPEKEPLDWNTRMKIAAGAAKGLEHLHDKASPPVIYRDFKSSNILLGEGFHP
KLSDFGLAKLGPVGDNTHVSTRVMGTYGYCAPEYAMTGQLTVKSDVYSFGVVFLELITGRKAIDNTKPQGEQNL
VAWARPLFKDRRKFPKMADPMLQGRFPMRGLYQALAVAAMCLQEQATTRPHIGDVVTALSYLASQTYDPNA
PTQHTRSNSSTPRARNVGGRNSEQRNGRSPNHHSPRTSKHGGEVSRTSSTGGDSGRRSGLDEMDMAGSQAG
SPAQTGRKRETPRTADRQRAIADAKMWGENSRERKRPNDSFDSTNE

>A362|A0A3B6DCW2

MGCFPCFDSGSDGELLYPKQGGGGGGNGTGGRTAAAASSSGVGAREERPMVPPRVEKLPAGAEKARAKGNA
GMKELSDLRDANGNVLSAQTFTFRQLTAATRNFREECFIGEGGFGRVYKGRLDGGQVVAIKQLNRDGNQGNK
EFLVEVLMLSLLHHQNLVNLVGYCADGEQRLLVYEYMPLGSLEDHLHDLPPDKEPLDWNTRMKIAAGAAKGLE
YLHDKAQPPVIYRDFKSSNILLGDDFHPKLSDFGLAKLGPVGDKSHVSTRVMGTYGYCAPEYAMTGQLTVKSDV
YSFGVVLLELITGRKAIDSTRPHGEQNLVSWARPLFNDRRKLPKMADPGLQGRYPMRGLYQALAVASMCIQSEA
ASRPLIADVVTALSYLASQIYDPNAIHASKKAGGDQRSRVSDSGRTLLKNDEAGSSGHKSDRDDSPREPPPGILND
RERMVAEAKMWGANLREKTRAAANAQGSLDSPTETG

>A363|PHAVU008G001400g

MGCFSCFAPTSTSKEDHNHRHHHHHHRQPNPNPNLNPNPSLPSQISKLPSGADKLRSRSNGGSKRELAPPNTK
EGHGAAGQIAAQTFTFRELATATKNFRPDSFVGEGGFGRVYKGRLETTGQIVAVKQLDKNGLQGNREFLVEVL
MLSLLHHPNLVNLIGYCADGEQRLLVYEFMALGSLEDHLHDLPPDKEPLDWNTRMKIAAGAAKGLEYLHDKAN
PPVIYRDFKSSNILLDEGYQPKLSDFGLAKLGPVGDKSHVSTRVMGTYGYCAPEYAMTGQLTVKSDVYSFGVVFL
ELITGRKAIDSTQPHGEQNLVTWARPLFNDRRKFSKLADPRLEGRFPMRGLYQALAVASMCIQESAATRPLIGDV
VTALSYLANQGYDPNNAGHGYRGSGDDKRNRDDKGGRILKNDEAGGSGRRWDLEGSEKDDSPRETARMLNR
DLDRERAVAEAKLWGENLRQKRQQSLQEGTYGSK

>A364|A0A2P6R064

MGCFPCFDSKEEEKLNPAKESDDRKQGQPTVSSNISRLPSGVDRLRSRSNGGSRRVDVGSKMPDPKDVVPGGQ
IAAQTFTFRELAAATKNFRPESFIGEGGFGRVYKGRLESTGQIVAVKQLDRNGLQGNREFLVEVLMLSLLHHTNL
VNLIGYCADGDQRLLVYEFMPLGSLEDHLHDVPLDKEVLDWNTRMKIAAGAAKGLEYLHDKANPPVIYRDFKSS
NILLEEGFHPKLSDFGLAKLGPTGDKSHVSTRVMGTYGYCAPEYAMTGQLTVKSDVYSFGVVFLELITGRKAIDST
RPHGEQNLVTWARPLFNDRRKFSKLADPRLQGRYPMRGLYQALAVASMCIQEQAATRPLIGDVVTALSYLANQ
AYDPTAASGHGHRSSGEKDERRRDERGGRILRNEEGGGSGRKWPDLDGGSEKDDSPKETARMLNRDLDRERA
VAEAKMWGENWREKRRQSAQGSFDGTNL

>A365|LOC18788970

MGCFPCFDSKEEEKLNNPVNEIDDRKQGQPTVSSNISRLPSGADRMRSRSNGGSRRDLGSKLPDLKDVPGVQIA
AQIFTFRELVTATKNFRPESFIGEGGFGRVYKGRLESSGQVVAVKQLDRNGLQGNREFLVEVLMLSLLHHPNLVN
LIGYCADGDQRLLVYEFMPLGSLEDHLHDLPLDREPLDWNTRMKIASGAAKGLEYLHDKANPPVIYRDFKSSNILL
EEGFHPKLSDFGLAKLGPTEDKSHVSTRVMGTYGYCAPEYAMTGQLTVKSDVYSFGVVFLELITGRKSIDSNRPH
GEQNLITWARPLFNDRRKFSKLADPRLQGRYPMRGLYQALAVASMCIQEQAATRPLIGDVVTALSYLANQSYDP
NMASGHGHRGSGEKDERRHRDERGGRILKNEEGGGSGRRWDLDGSEKDDSPKETARMLNRDLDRERAVAEA
KMWGENWREKRRQSAQGSFDGTNL

>A366|100777987

MGCFSCFDSREDEKLNPNPQQENHQHEHEHEHDLKPPVPSRISRLPPSASASASASAVGADKLRSTTSNGNGES
TAVQIAAQTFSFRELAAATKNFRPQSFLGEGGFGRVYKGRLETTGQVVAVKQLDRNGLQGNREFLVEVLMLSLL
HHPNLVNLIGYCADGDQRLLVYEFMPFGSLEDHLHDLPPDKEPLDWNTRMKIAAGAAKGLEYLHDKANPPVIY
RDFKSSNILLDEGYHPKLSDFGLAKLGPVGDKSHVSTRVMGTYGYCAPEYAMTGQLTVKSDVYSFGVVFLELITG
RKAIDSTRPHGEQNLVTWARPLFSDRRKFPKLADPQLQGRYPMRGLYQALAVASMCIQEQAAARPLIGDVVTA
LSFLANQAYDHRGAGDDKKNRDDKGGRILKNDVGGGSGRRWDLEGSEKDDSPRETARMLNNRDLDRERAVA
EAKIWGENWREKRRQSAQGSFDGSNA

>A367|101256183

MGCFSCFDSKEDEKLNPQKDRDDSDRKQPPPSNISRLSSGADRLKIRSSNGSKREFLGLKDAPDVQIAAHTFTFRE
LAAATNNFRPESFIGEGGFGRVYKGQLPSGQVVAVKQLDRNGLQGNREFLVEVLMLSLLHHPNLVNLIGYCADG
DQRLLVYEFMPLGSLEDHLHDLPPDKEPLDWNTRMKIASGAAKGLEHLHDKANPPVIYRDFKSSNILLKENFFPK
LSDFGLAKLGPTGDKSHVSTRVMGTYGYCAPEYAMTGQLTVKSDVYSFGVVFLELITGRKAIDSTKPQGEQNLV
AWARPLFNDRRKFAKLADPSLQGQFPMRGLYQALAVASMCIQEQAAGRPLIGDVVTALSYLANQSYDPGTVP

GQIHRFGADSVDRRNKDDRVGRILRSEDGEGGGSGRKWDVDGGSEKEDSPRETARMLNRDLDRERAVAEAK
MWGENWRDKRRQNGQGSFDGGNE

>A368|A0A0B0P128

MGCFSCFDSKEEEKLNTVNESNDPKQAQPTVSSNISRLSSGGDRLRSRSNGGSKRELPCPRDGPGVQIAAHIFSF
RELAAATKNFRPESFLGEGGFGHVYKGQLESTGQVVAVKQLDRNGLQGNREFLVEVLMLSLLHHPNLVNLIGYC
ADGDQRLLVYEFMPLGSLEDHLHDLPPGKEPLDWNTRMKIAAGAAKGLEYLHDKANPPVIYRDFKSSNILLDEG
FHPKLSDFGLAKLGPVGDKSHVSTRVMGTYGYCAPEYAMTGQLTVKSDVYSFGVVFLELITGRKAIDSTRPHGEQ
NLITWARPLFTNRRKLSKLADPLLQGRFPMRGLYQALAVASMCIQEEAVARPHIGDVVTALSYLANQAYEPNAS
GHGHSGSGETDEKRYRDDRGGRVSKNDEGGVSGCRWDLEGSEKDDSPKESVRMLNRDLDRERAVAEAKMW
GENWREKRRQSAQGSSDGSNG

>A369|A0A0D2M5V1

MGCFSCFDSKEEEKLNTVNETNDPKRPQPIVSSNISRLSSGGDRLRSRSNGGSKREIPSLRDGPGVQIAAQTFSFR
ELAAATKNFRPESFLGEGGFGRVYKGRLESTGQVVAVKQLDRNGLQGNREFLVEVLMLSLLHHPNLVNLIGYCA
DGDQRLLVYEFMPLGSLEDHLHDLPPSKEPLDWNTRMKIAAGAAKGLEYLHDKANPPVIYRDFKSSNILLEEGFH
PKLSDFGLAKLGPVGDKSHVSTRVMGTYGYCAPEYAMTGQLTVKSDVYSFGVVFLELITGRKAIDSSQPHGEQN
LITWARPLFNNRRKLSKLADPLLQGRFPMRGLYQALAVASMCIQEEAATRPLIGDVVTALSYLANQAYDPNTTG
NGHRGSGETDEKRYRDDRGGRVSKNDEGGVSGRKWDLEGSEKEDSPRETARMLNRDLDRERAVAEAKMWG
ENWREKRRQSAQGSSDGSNG

>A370|A0A2C9UTI2

MGCFPCFDSREEETLNPQKESDDRKQSLPTESSNISKLSSGSDRLKSRNNGRSKRELPVPKDGLPAGNIAAQTFTF
RELAAATKNFRPESFIGEGGFGRVYKGRLESTGQVVAVKQLDRNGLQGNREFLVEVLMLSLLHHPNLVNLIGYCA
DGDQRLLVYEFMPLGSLEDHLHDLPPDKEPLDWNRRMRIAAGAAKGLEYLHDKANPPVIYRDFKSSNILLDEGF
HPKLSDFGLAKLGPTGDKSHVSTRVMGTYGYCAPEYAMTGQLTVKSDVYSFGVVFLELITGRKAIDSTRPHGEQ
NLVTWARPLFNDRRKFSKLADPQLQGRYPMRGLYQALAVASMCIQEQAAARPLIGDVVTALSYLANQAYEPSS
GGHGYRGSGDKDEKRREERGGQLPKNEEGGVSGHRWDLDGSEKEDSPRETARMLNRDLEREKAVAEAKMW
GENWREKRRQSAQGSFDGSNG

>A371|A0A498ICX1

MGCFPCFDSKEEEKLNNPAAEIDDRKQGQPTVSNNISRLPSGVDRLRSRSNGGSRRELPKLPDPKDVVPGGQIA
AQIFTFRELATATKNFRPESFIGEGGFGRVYKGRLESTGQVVAVKQLDRNGLQGNREFLVEVLMLSLLHHPNLVN
LIGYCADGDQRLLVYEFMPLGSLEDHLHDLPSDKEPLDWNTRMKIASGAAKGLEYLHDKANPPVIYRDFKSSNIL
LEEGFHPKLSDFGLAKLGPTGDKSHVSTRVMGTYGYCAPEYAMTGQLTVKSDVYSFGVVFLELITGRKAIDSDRP
HGEQNLITWARPLFNDRRKFAKLADPRLQGRYPMRGLYQALAVASMCIQEQAATRPLIGDVVTALSYLANQSY
DPNLASGHGHRGSGEKDEKRHRDGGRILKNEEGGGSGRRWDLDGSEKDDSPKETVRMLDRERAVAEAKMW
GENWREKRRLSAQGSFDGTNL

>A372|LOC21400483

MGCFSCFDSKEEEKLNPDKQSDDLKQSQQPTLTSNLARLPSGADRVRSRSNGGSKRELTLPKDVPGVHIAAQTF
TFRELAAATKNFRPECFLGEGGFGRVYKGQLESTGQVVAVKQLDRNGLQGNREFLVEVLMLSLLHHHNLVNLIG
YCADGDQRLLVYEFMPLGSLEDHLHDLPPEKEPLDWNTRMKIAAGAAKGLEYLHDKANPPVIYRDFKSSNILLEE
GFHPKLSDFGLAKLGPTGDKSHVSTRVMGTYGYCAPEYAMTGQLTVKSDVYSFGVVFLELITGRKAIDSTRPHGE

QNLVTWARPLFNDRRKFSKLADPRLQGRYPMRGLYQALAVASMCIQEQAATRPLIGDVVTALSYLANQAYDPT
TTSGHGYRGSGDKDDKRIRDGGRILRNEEGGGSGRRWDLEGSEKEDSPRETARILNRDLDRERAVAEAKMWGE
NLREKRRQSAQGSFDAANS

>A373|A0A287FS82

TSRTIAHDKSKPQGLLESKKDTVIPREGNNQHIAAHTFTFRELAAATKNFRQDCLLGEGGFGRVYRGRLDNGQA
VAVKQLDRNGLQGNREFLVEVLMLSLLHHDNLVNLIGYCADGDQRLLVYEYMPLGSLEDHLHDIPPEKEPLDW
NTRMKIAAGAAKGLEYLHDKASPPVIYRDFKSSNILLGEGFHPKLSDFGLAKLGPVGDNTHVSTRVMGTYGYCAP
EYAMTGQLTVKSDVYSFGVVFLELITGRKAIDNTKPHGEQNLVAWARPLFKDRRKFPKMADPSLQGRFPMRGL
YQALAVAAMCLQEQAATRPFIGDVVTALSYLASQAYDPNAPTQHSRSNASTPRTRDRGSVNGDQRRIRSPNHH
SPDLRRKEATTSSKYEAEVSRNSSGGGSGRRSGLDDRDVTGSQQGSPAQTGKRRETSRTSERQRAIAEAKTWGE
NSRERKWPNARGSFDSTNE

>A374|B9RM95

MGCFPCFDSREEETLNPQKESDDRKQSLPTESSNISKLSSGADRLRSRSNGRSKRELPSPKDGPVPGVNIAAQTFT
FRELAAATKNFRQESFIGEGGFGRVYKGLLETTGQVVAVKQLDRNGLQGNREFLVEVLMLSLLHHPNLVNLIGYC
ADGDQRLLVYEFMPLGSLEDHLHDLPPAKEPLDWNTRMRIAAGAAKGLEYLHDKANPPVIYRDFKSSNILLDEG
FHPKLSDFGLAKLGPTGDKSHVSTRVMGTYGYCAPEYAMTGQLTVKSDVYSFGVVFLELITGRKAIDSTRPHGEQ
NLVTWARPLFNDRRKFSKLADPQLQGRYPMRGLYQALAVASMCIQEQAAARPLIGDVVTALSYLANQAYEPNS
TGHGRERDDKRNRDERGGQLSKSEEGGGSGRRWDLEGSEKEDSPRETVRMLNRDLDRERAVAEAKMWGEN
WREKRRQSAQGSFDGSNG

>A375|A0A067JSB9

MGCFPCFDSREEETLNPQKESDDRKQSLPTEASNISKLSSGADRLRSRTNGRSKREFPIPKDAPGVNIAAQIFTFRE
LAAATKNFRPESLLGEGGFGRVYKGRLESTGQVVAVKQLDRNGLQGNREFLVEVLMLSLLHHPNLVNLIGYCAD
GDQRLLVYEFMPMGSLEDHLHDLPPEKEPLDWNTRMRIAAGAAKGLEYLHDKASPPVIYRDFKSSNILLDEGFH
PKLSDFGLAKLGPTGDKSHVSTRVMGTYGYCAPEYAMTGQLTVKSDVYSFGVVFLELITGRKAIDSTRPHGEQNL
VTWARPLFNDRRKFSKLADPLLQGRYPMRGLYQALAVASMCIQEQAAARPLIGDVVTALSYLANQAYETRGSG
DKDDKRHRDERGGQLSKSEEGGGSGCRWDLDGSEKEDSPRETARMLNRDLDRERAVAEAKMWGENWREKR
RQSAQGSFDGSNG

>A376|XP_002871549.1

MGCFSCFDSSDDDEKLNPVVESNGQKKQSQPTVSNNISGLPSGGEKLSSKTNGGSKRELLLPRDGLGQIAAHTF
AFRELAAATMNFHPDTFLGEGGFGRVYKGRLDSTGQVVAVKQLDRNGLQGNREFLVEVLMLSLLHHPNLVNLI
GYCADGDQRLLVYEFMPLGSLEDHLHDLPPDKEALDWNMRMKIAAGAAKGLEFLHDKANPPVIYRDFKSSNILL
DEGFHPKLSDFGLAKLGPTGDKSHVSTRVMGTYGYCAPEYAMTGQLTVKSDVYSFGVVFLELITGRKAIDSEMP
HGEQNLVAWARPLFNDRRKFIKLADPRLKGRFPTRALYQALAVASMCIQEQAATRPLIADVVTALSYLANQGYD
PSKDDSKRNRDERGARLITRNDDGGGSGSKFDLEGSEKEDSPRETGRILNRDINRERAVAEAKMWGESLREKRR
QSEQGTSESNSTG

>A377|Cagra.0596s0008.1

MGCFSCFDSSDDEKLNPVEESNGQKKQLQPTVSNSISGLPSGGEKLSSKSNGGSKRELLLPRDGLGQIAAHTFAF
RELAAATMNFHPDTFLGEGGFGRVYKGRLDSTGQVVAVKQLDRNGLQGNREFLVEVLMLSLLHHPNLVNLIGY
CADGDQRLLVYEFMPLGSLEDHLHDLPPDKEALDWNMRMKIAAGAAKGLEFLHDKANPPVIYRDFKSSNILLDE

GFHPKLSDFGLAKLGPTGDKSHVSTRVMGTYGYCAPEYAMTGQLTVKSDVYSFGVVFLELITGRKAIDSEMPHG
EQNLVAWARPLFNDRRKFIKLADPKLKGRFPTRALYQALAVASMCIQEQAATRPLIADVVTALSYLANQAYDPSK
DESRRNRDERGARLITRNDEGGGSGSKFDLDGSEKEDSPRETARILNRDINRERAVAEAKMWGESLREKRRQSE
QGTSESNSTG

>A378|A0A087G6P0

MGCFSCFDSSDDEKLNPVEESKGQKQSQPTISNNIAGLPSGGEKLTLKSNGGSKRELLLPRDGLSQIAAHTFAFRE
LAAATMNFHPDTFLGEGGFGRVYKGRLDSTGQVVAVKQLDRNGLQGNREFLVEVLMLSLLHHPNLVNLIGYCA
DGDQRLLVYEFMPLGSLEDHLHDLPPDKEALDWNMRMKIAAGAAKGLEFLHDKANPPVIYRDFKSSNILLDEGF
HPKLSDFGLAKLGPTGDKSHVSTRVMGTYGYCAPEYAMTGQLTVKSDVYSFGVVFLELITGRKAIDSEMPHGEQ
NLVAWARPLFNDRRKFIKLADPKLKGRFPTRALYQALAVASMCIQEQAATRPLIADVVTALSYLANQGYDPNKD
DNRRNRDERGGKLITRNDEGGGSGGKFDLDGSEKEDSPRETARILNRDINRERAVAEAKMWGESLREKRRQSE
QGTSESNSTG

>A379|Thhalv10013511m

MGCFSCFDSSDDETLNPVEESKGQKQSQPTVSNNISGLPSGGEKLSSKSNGGSKRELLLPRDGLGQIAAHTFAFR
ELAAATMNFHPDTFLGEGGFGRVYKGRLDSTGQVVAVKQLDRNGLQGNREFLVEVLMLSLLHHPNLVNLIGYC
ADGDQRLLVYEFMPLGSLEDHLHDLPPDKEALDWNMRMKIAAGAAKGLEFLHDKANPPVIYRDFKSSNILLDEG
FHPKLSDFGLAKLGPTGDKSHVSTRVMGTYGYCAPEYAMTGQLTVKSDVYSFGVVFLELITGRKAIDSEMPHGE
QNLVAWARPLFNDRRKFIKLADPKLKGRFPTRALYQALAVASMCIQEQAATRPLIADVVTALSYLANQAYDPNK
DDSRRNRDERGGRLITKNDEGGGSGSKFDLEGSEKEDSPRETARILNRDINRERAVAEAKMWGESLREKRRQSE
QGTSESNSTG

>A380|A0A1S4DLR6

MGCFSCFDSKEEEKLNPQKDRRDDRKEVHLTAPSNISRLSSGADRLKTRSINGSKREFLGLKDAPDVQIAAHTFTF
RELAAATSNFRPESFIGEGGFGRVYKGRLPSGQVVAVKQLDRNGLQGNREFLVEVLMLSLLHHPNLVNLIGYCA
DGDQRLLVYEFMPLGSLEDHLHDLPPDKEPLDWNTRMKIAAGAAKGLEYLHDKANPPVIYRDFKSSNILLEENFF
PKLSDFGLAKLGPTGDKSHVSTRVMGTYGYCAPEYAMTGQLTVKSDVYSFGVVFLELITGRKAIDSTMPQGEQN
LVAWARPLFNDRRKFAKLADPRLQGQFPMRGLYQALAVASMCIQEQAAARPLIGDVVTALSYLANQVDDKRN
KDDRGGRICRNEDGAGGGSGRKWPDLDGGSEKEDSPRETARMLNRDLDRERAVAEAKMWGENWREKRRQ
NAQGSFDGTNG

>A381|XP_009125889.1

MGCFSCFDSSDDETLNPAAEESSKTQKQSQPTVSNSLSALPSGGEKLNSNSKSNGGAKTELLLPRDGLGQIAAHT
FTFRELAAATMNFHPDTFLGEGGFGRVYKGRLDSTGQVVAVKQLDRNGLQGNREFLVEVLMLSLLHHPNLVNLI
GYCADGDQRLLVYEFMSLGSLEDHLHDLPPDKEALDWNMRMKIAAGAAKGLEFLHDKANPPVIYRDFKSSNILL
DEGFHPKLSDFGLAKLGPTGDKSHVSTRVMGTYGYCAPEYAMTGQLTVKSDVYSFGVVFLELITGRKAIDTDMP
HGEQNLVAWARPLFNDRRKFIKLADPKLKGRFPTRALYQALAVASMCIQGEAATRPLIADVVTALSYLANQGYD
PNKNERGARLITRNDEGGGSGSKFDLEGSEKEDSPRETARMLNRDINRERAVAEAKMWGESLREKRRQSEQGT
SESNSTG

>A382|A0A1S3ZDX8

MGCFSCFDSKEEEKLNPQRDDRKEVHLTAPSNISRLSSGADRLKTRSINGSKREFLGLKDAPDVQIAAHTFTFREL
AAATSNFRPESFIGEGGFGRVYKGRLPSGQVVAVKQLDRNGLQGNREFLVEVLMLSLLHHPNLVNLIGYCADGD

QRLLVYEFMPLGSLEDHLHDLPPDKEPLDWNTRMKIAAGAAKGLEYLHDKANPPVIYRDFKSSNILLEENFFPKLS
DFGLAKLGPTGDKSHVSTRVMGTYGYCAPEYAMTGQLTVKSDVYSFGVVFLELITGRKAIDSTMPQGEQNLVA
WARPLFNDRRKFAKLADPRLQGQFPMRGLYQALAVASMCIQEQAPARPLIGDVVTALSYLANQVDDKRNKDD
RGGRICRNEDGAGGGGSGRKWPDLDGGSEKEDSPRETARMLNRDLDRERAVAEAKMWGENWREKRRQNA
QGSFDGTNG

>A383|A0A0E0G784

MGCFSCFDSPAEEQLNPKVGGPYGGGSSSSAAAAAYGGGGGSGAGRHGERGGGYPDLHHHHQQQQLPMAA
PRVEKLSAGAEKTRVKSNAILREPSAPKDANGNVISAQTFTFRELATATRNFRPECFLGEGGFGRVYKGRLESTGQ
VVAIKQLNRDGLQGNREFLVEVLMLSLLHHQNLVNLIGYCADGDQRLLVYEYMHFGSLEDHLHDLPPDKEALD
WNTRMKIAAGAAKGLEYLHDKANPPVIYRDFKSSNILLDESFHPKLSDFGLAKLGPVGDKSHVSTRVMGTYGYC
APEYAMTGQLTVKSDVYSFGVVLLELITGRRAIDSTRPHGEQNLVSWARPLFNDRRKLPKMADPRLEGRYPMR
GLYQALAVASMCIQSEAASRPLIADVVTALSYLASQSYDPNAAHASRKPGGDQRSKVGENGRVVSRNDEASSSG
HKSPNKDREDSPKEPPGILNKDFDRERMVAEAKMWGDRERMVAEAKMWGDRERMVAEAKMWGENWRD
KRRAIENGQGSLDSPTENG

>A384|LOC4329463

MGCFSCFDSPAEEQLNPKVGGPYGGGSSSSAAAAAYGGGGGSSAGRHGERGGGYPDLHHHHQQQQLPMAA
PRVEKLSAGAEKTRVKSNAILREPSAPKDANGNVISAQTFTFRELATATRNFRPECFLGEGGFGRVYKGRLESTGQ
VVAIKQLNRDGLQGNREFLVEVLMLSLLHHQNLVNLIGYCADGDQRLLVYEYMHFGSLEDHLHDLPPDKEALD
WNTRMKIAAGAAKGLEYLHDKANPPVIYRDFKSSNILLDESFHPKLSDFGLAKLGPVGDKSHVSTRVMGTYGYC
APEYAMTGQLTVKSDVYSFGVVLLELITGRRAIDSTRPHGEQNLVSWARPLFNDRRKLPKMADPRLEGRYPMR
GLYQALAVASMCIQSEAASRPLIADVVTALSYLASQSYDPNAAHASRKPGGDQRSKVGENGRVVSRNDEASSSG
HKSPNKDREDSPKEPPGILNKDFDRERMVAEAKMWGDRERMVAEAKMWGDRERMVAEAKMWGENWRD
KRRAIENGQGSLDSPTENG

>A385|LOC102630702

MGGCFPCFGSSNNKETGGGGGGGAVGAVKELNRKDSAKDGSIAQSHHVNRVSSDKSKSRNGSDPKKEPAIPKE
PTAHIAAQTFTFRELAAATKNFRPECLLGEGGFGRVYKGRLESTGQVVAVKQLDRNGLQGNREFLVEVLMLSLL
HHSNLVNLIGYCADGDQRLLVYEFMPLGSLEDHLHDLPPDKEPLDWNTRMKIAAGAAKGLEYLHDKANPPVIYR
DLKSSNILLDEGFHPKLSDFGLAKLGPVGDKTHVSTRVMGTYGYCAPEYAMTGQLTLKSDVYSFGVVFLELITGRK
AIDNTRPPGEHNLVAWARPLFKDRRKFPKMADPLLQGRYPMRGLYQALAVAAMCLQEQAATRPLIGDVVTAL
TYLASQTYDPNAASNLSNRVGPSTPRNRDDRRSMADGQDSPDEHGRVGRHGSPSTHKNSPDYRKRSHPRDGS
TGAELSRNETGGGSGRKWLGDDLERQESQRDSPVNTGRARQSPRNRDLDRERAVAAAKVWGENWREKKRA
NAMGSFDGTDE

>A386|A0A1U7YW15

MGGCLPCFGSSDKEKNTVKESGKKDSAKEGSTAPQSHHVSRVSSDKSKSRNGSDSRKETPAAKDGQATHIAAQ
TFTFRELAAATKNFRQECLLGEGGFGRVYKGRLESTGQVVAVKQLDRNGLQGNREFLVEVLMLSLLHHPNLVNLI
GYCADGDQRLLVYEYMPLGSLEDHLHDLPPDKEPLDWNTRMKIAAGAAKGLEYLHDKANPPVIYRDFKSSNILL
DEGYHPKLSDFGLAKLGPVGDKTHVSTRVMGTYGYCAPEYAMTGQLTLKSDVYSFGVVFLELITGRKAIDNTRAP
GEHNLVAWARPLFKDRRKFPKMADPLLQGRYPMRGLYQALAVAAMCLQEQAATRPLIGDVVTALSYLASQTY
DPNAAIAQSNRIGSSATRDRDERRNLSGGPESQDEVGRIGRHGSQSPHQNSPDFRQRDPGRGMSFGADVGRG

ETSGGSGRKWGLDELEKQESQRDSPGHATRARETPRNYNRDFDRERAVAEAKVWGENWRERKRANAMGSF
DGTNEKP

>A387|A0A498IDV4

MGGCFPCFGSSKNKEGSGGGGGVKEVTKKDSSVKEGSAAQSHRVTRVSSDKKSRNGSDPKKEPPIPKDGSTAHI
AAQTFTFRELAAATKNFRPECLLGEGGFGHVYKGRLESTGQVVAVKQLDRNGLQGNREFLVEVLMLSLLHHPNL
VNLIGYCADGDQRLLVYEFMPLGSLEDHLHDLPSDKEPLDWNTRMKIAAGAAKGLEYLHDRANPPVIYRDLKSS
NILLDEGFHPKLSDFGLAKLGPVGDKTHVSTRVMGTYGYCAPEYAMTGQLTLKSDVYSFGVVFLELITGRKAIDN
TRGPGEHNLVAWARPLFKDRRKFPKMADPLLQGRYPMRGLYQALAVAAMCLQEQAATRPLIGDVVTALTYLA
SQTYDPNAATTPSNRGGSSTPRHRDERRNMGDGLDSPDEYVRGGRHGSPATHKNSPDFRRRDPNRDLNTGVE
LGRIETGTGSGRRWGLDGLERQESQRDSPQSAGRARETPRNRDLDRERAVAEAKVWGENWREKKRANAMGS
FDGTNE

>A388|A0A0B0MKC9

MGGGGCFPCFGSSNKEKSNNGGKSIKELNNKDSIKDGSVGQSHHVNRASLDKSKSRSGSDSKKEPAVSKNGSTE
NIPAQTFTFRELAAATKNFRPECLLGEGGFGRVYKGRLESTGQVVAVKQLDRNGLQGNREFLVEVLMLSLLHHP
NLVNLIGYCADGDQRLLVYEFMPLGSLEDHLHDLPPDKEPLDWNTRMKIAAGAAKGLEYLHDEASPPVIYRDLK
SSNILLDEGYHPKLSDFGLAKLGPVGDKTHVSTRVMGTYGYCAPEYAMTGQLTLKSDVYSFGVVFLELITGRKAID
NMRAPGEQNLVAWARPLFKDRRKFPKMADPLLQGRYPIRGLYQALAVAAMCLQEQAATRPLMRDVVTALTYL
ASQTYDPNAPGNQSNRVGPSNPRLKDDRNMADGLDSPEGRGWHHGSPSTHRNSPDYRKRNQVRESSTGSELI
RNDAGGGSGRKCGSDDSEGQESNRGSPLNASRARETPHNRVLDRERAVAEAKVWGENWRERKRANAMGSF
DGTNE

>A389|A0A0D2NHF1

MGGGGCFPCFGSSNKEKSNNGGKSIKELNNKDSTKDGSVGQSHHVNRASLDKSKSRSGSDSKKEPAVSKNGST
ENIPAQTFTFRELAAATKNFRPECLLGEGGFGHVYKGRLESTGQVVAVKQLDRNGLQGNREFLVEVLMLSLLHH
PNLVNLIGYCADGDQRLLVYEFMPLGSLEDHLHDLPPDKEPLDWNTRMKIAAGAAKGLEYLHDKASPPVIYRDL
KSSNILLDEGYHPKLSDFGLAKLGPVGDKTHVSTRVMGTYGYCAPEYAMTGQLTLKSDVYSFGVVFLELITGRKAI
DNMRAPGEQNLVAWARPLFKDRRKFPKMADPLLQGCYPMRGLYQALAVAAMCLQEQAATRPLIGDVVTALT
YLASQTYDPNAPGNQSNRVGPSNPRLKDDRNMADGLDSPEGRGWHHGSPSTHRNSPDYRKRNQMRESSTGS
ELIRNDAGGESGRKCGSDDSERQESNRGSPLNGSRARETPHNRVLDRERAVAEAKVWGENWRERKRANAMG
SFDGTNE

>A390|A0A2P6QYB5

MGGCFPCFGSSNKEGSGGGAGVKEVSKKESVKEGSATQSHHVTRVGSDKSKSRNGSDPKKEPPIPKDVPTAHIA
AQTFTFRELAAATKNFKPECLLGEGGFGRVYKGRLESTGQVVAVKQLDRNGLQGNREFLVEVLMLSLLHHPNLV
NLIGYCADGDQRLLVYEFMPLGSLEDHLHDIPTEKEPLDWNTRMKIAAGAAKGLEYLHDKANPPVIYRDLKSSNI
LLDEDFHPKLSDFGLAKLGPVGDKTHVSTRVMGTYGYCAPEYAMTGQLTLKSDVYSFGVVFLELITGRKAIDNTR
GPGEHNLVAWARPLFKDRRKFPKMADPLLQGRYPMRGLYQALAVAAMCLQEQAATRPLIGDVVTALTYLASQ
TYDPNAASGHSNRVGPSTPRHKDDRRNMGDGLDSPDEPGRGGRHGSHSYRNSPDFRRKDPNRDLGAGVELG
RIETGNGSGRKWGLDGLEQQDSQRDSPVSAGRARETLRNRDLDRERAVAEAKVWGENWREKKRANVMGGS
FDGTNE

>A391|A0A067KYE6 Jatropha curcas PROTEIN KINASE

MGGCFPCFGSSNKEGGSGGGSAVKEVAKKDSVKDGSVAQSHHVGRVSSDKSKLRNGTDPKKEPIIPKDGPTANI
AAQTFTFRELAAATKNFRPECLLGEGGFGRVYKGRLESTGQVVAVKQLDRNGLQGNREFLVEVLMLSLLHHPNL
VNLIGYCADGDQRLLVYEFMPLGSLEDHLHDLPADKEPLDWNTRMKIAAGAAKGLEYLHDKANPPVIYRDLKSS
NILLDEGYHPKLSDFGLAKLGPVGDKTHVSTRVMGTYGYCAPEYAMTGQLTLKSDVYSFGVVFLELITGRKAIDN
TRAPGEHNLVAWARPLFKDRRKFPKMADPLLQGRYPMRGLYQALAVAAMCLQEQAATRPLIGDVVTALTYLA
SQTYDPNAANQSNRIGPSTPRSRDDRRGMADGLDSPDEHGRGRHGSPSTYKNSPDYRRRDPVRELSSGADLGR
SETGGGSGRKWGSDDSERQDSQRDSPLNTSRARETPRNRDLDRERAVAEAKVWGENWREKKRANAMGSFD
ATNE

>A392|B9RT11

MGGCFPCFGSSNKEGTDGGGAIKEVAKKDSVKEGSVAQSHHVGRVSSDKSKSRNGSDPKKEPTIPKDGPTAHIA
AQTFTFRELAAATKNFRQECLLGEGGFGRVYKGRLESTGQVVAVKQLDRNGLQGNREFLVEVLMLSLLHHPNLV
NLIGYCADGDQRLLVYEFMPLGSLEDHLHDFPSDKEPLDWNTRMKIAAGAAKGLEYLHDKANPPVIYRDLKSSNI
LLDEGYHPKLSDFGLAKLGPVGDKTHVSTRVMGTYGYCAPEYAMTGQLTLKSDVYSFGVVFLELITGRKAIDNTR
APGEHNLVAWARPLFKDRRKFPKMADPLLQGRYPMRGLYQALAVAAMCLQEQAATRPLIGDVVTALTYLASQ
TYDPNSANQSNRVGPSTPRNRDDRKGMADGLDSPDEHGRGGWHGSPSTYKNSPDYRRRDPMRESSTASELG
RSETGGGSGRKWGLDDSERQDSQRGSPVNTSRVRETPRNRDLDRERAVAEAKVWGENWREKKRANAMGSF
DGTNE

>A393|A0A0B0MEM0

MGGCFPCFGSSNKAGSNGGGSVKELSKKDSTKDSSVGQPHHVNRVNSDKSKSRSVSDPKKEPAVPKDGPTANI
AAQTFTFRELAAATKNFRPECLLGEGGFGRVYKGRLESTGQVVAVKQLDRNGLQGNREFLVEVLMLSLLHHPNL
VNLIGYCADGDQRLLVYEFMPLGSLEDHLHDLPSDKEPLDWNTRMRIAAGAAKGLEYLHDKANPPVIYRDLKSS
NILLDEGFHPKLSDFGLAKLGPVGDKTHVSTRVMGTYGYCAPEYAMTGQLTLKSDVYSFGVVFLELITGRKAIDN
TRAPGEQNLVAWARPLFRDRRKFPKMADPLLQGRYPIRGLYQALAVAAMCLQEQAATRPLIGDVVTALTYLAS
QTYDPNAPNNQSNRVGPSTPRVKDDRRSMADGLDSPDARGRVGSPSTHRNSPDYRKKNHVREMSSGAELSR
NEPGEGSGRKWGSLDESEQQESHTDSPMNSARARETSRNRDLDRERAVAEAKVWGENWREKKRANAMGDS
DGRND

>A394|A0A2C9WAG1

MGGCFPCFGSSSNKEGSGGAVKEVAKKDSVKEGSVAQSHRVGRVSSDKSKSQNGSDPKKEAVVPKDGPTANIA
AQTFTFKELAAATKNFRPECLLGEGGFGRVYKGRLETTGQVVAVKQLDRNGLQGNREFLVEVLMLSLLHHPNLV
NLIGYCADGDQRLLVYEFMPLGSLEDHLHDLPPDKEPLDWNTRMKIAAGAAKGLEYLHDKANPPVIYRDLKSSNI
LLDEGYHPKLSDFGLAKLGPVGDKTHVSTRVMGTYGYCAPEYAMTGQLTLKSDVYSFGVVFLELITGRKAIDNTR
APGEHNLVAWARPLFKDRRKFPKMADPLLQGRYPMRGLYQALAVAAMCLQEQAATRPLIGDVVTALTYLASQ
TYEPSAANQSNRVGPSTPWNRDDRRGMADGLHSPDEHGSGRRHGSPSTRKNSPDYRKRDTVRELSSGTELRR
SDTGGGSGRKWGLDDSEQQDSIKGSPVNTSRARETSRNRDLDRERAVADAKVWGQNWREKKRANAMGSFD
GTKE

>A395|A0A0B0MRF4

MGGCFPCFGSSNKESNNGGTTVKELQNKDSTKDGSVGQFHHVNRVNSDKSKSQSGSDSKKEPAVPKDGPTAN
IAAQTFTFRELATATKNFRPECLLGEGGFGRVYKGRLESTGQVVAVKQLDRNGLQGNREFLVEVLMLSLLHHPNL
VNLIGYCADGDQRLLVYEFMPLGSLEDHLHDLPPGKEPLDWNTRMKIAAGAAKGLEYLHDKANPPVIYRDLKSA
NILLGEGYHPKLSDFGLAKLGPVGDKTHVSTRVMGTYGYCAPEYAMTGQLTLKSDVYSFGVVFLELITGRKAIDN

TRSHGEHNLVAWARPLFKDRRKFPQMVDPLLLGRYPMRGLYQALAVAAMCLQEQAATRPLIGDVVTALTYLAS
QTYDPNVASNQSNRVGPSTPRRKNDRRGMVDGLDSPDEHGQRGSPSSHRNSPDYLMRNHARKLSTGAELGR
HETGGGSGRKWGFEDSERHESHRGSPLNTTRTRETSRNRDFDRERAVAEAKVWGENWREKKRANAIGSFDST
NG

>A396|A0A0D2Q565

MGGCFPCLGSSNKESNNGGTTVKELQNKDSTKDGSVGQFHHVSRVNSDKSKYQSGSDSKKEPAVPKDGPMA
NIAAQTFTFRELATATKNFRPECLLGEGGFGHVYKGRLESTGQVVAVKQLDRNGLQGNREFLVEVLMLSLLHHP
NLVNLIGYCADGDQRLLVYEFMPLGSLEDHLHDLPPGKETLDWNTRMKIAAGAAKGLEYLHDKANPPVIYRDLK
SANILLGEGYHPKLSDFGLAKLGPVGDKTHVSTRVMGTYGYCAPEYAMTGQLTLKSDVYSFGVVFLELITGRKAID
NARSHGEHNLVAWARPLFKDRRKFPQMVDPLLQGHYPMRGLYQALAVAAMCLQEQAATRPLIGDVVTALTYL
ASQTYDPNAASNQSNRVGPSTPRRKNDRRGMLDGLDSPDEHGQRGSPSSHRNSPDYRMRNHARKLSTGAEL
GRNETGGGSGRKWGFEDSERHESHRGSPLNTTRTTETSRNRDFDRERAVAEAKVWGENWREKKRANAIGSFD
STNG

>A397|A0A1J6KHY8

MGGCFPCFRSSNKESGNGVKEVVKKESFKDGSAAQSIHLSKVNSDKSKSRGSHDPKKDPAIPKDGPTAHIAAQT
FTFRELAAATKNFRPECLLGEGGFGRVYKGRLESTGQVVAVKQLDRNGLQGNREFLVEVLMLSLLHHPNLVNLIG
YCADGDQRLLVYEFMPLGSLEDHLHDLPPDKEPLDWNTRMKIAAGAAKGLEYLHDKANPPVIYRDLKSSNILLDE
GYHPKLSDFGLAKLGPVGDKTHVSTRVMGTYGYCAPEYAMTGQLTLKSDVYSFGVVFLELITGRKAIDNARSHG
EHNLVAWARPLFKDRRKFPKMADPLLQGRYPMRGLYQALAVAAMCLQEQASTRPLIGDVVTALSYLASQTYDP
NAVGAQSNRVGSSTPRSREDRLHSSDGVNSPDEHCSAHHGSPSIQRNSPDSRKRDSARDFNTGIELRKIATSGGS
GRKWGLDESERPDSQKNSPVSAGRTRETPRNRDLDRERAVAEAKVWGENWRDKKKTSARGSSFDGMNE

>A398|A0A1R3JU90

MGGCFPCFGSSNKEGSNGGGTVKELSKKDSTKDASVGQAHHVNRVSSDKSKSRGASDSKKEPAVPKDGPTANI
AAQTFTFRELAAATKNFRPECLLGEGGFGRVYKGRLESTGQVVAVKQLDRNGLQGNREFLVEVLMLSLLHHPNL
VNLIGYCADGDQRLLVYEFMPLGSLEDHLHDLPPDKEPLDWNTRMKIAAGAAKGLEYLHDKANPPVIYRDLKSS
NILLDEGYHPKLSDFGLAKLGPVGDKTHVSTRVMGTYGYCAPEYAMTGQLTLKSDVYSFGVVFLELITGRKAIDN
TRAPGEHNLVAWARPLFKDRRKFPKMADPLLQGRYPMRGLYQALAVAAMCLQEQAATRPLIGDVVTALTYLA
SQTYDPNAPSNQSNRVGPSTPRLRDDRRGMADGLDSPDERGQHGSPSTHRNSPDYRKRNHAREMSTGSEFG
RSETGAGSGRKWGLDDSERPESQRDSPLNPLRARETPRNRDLDRERAVAEAKVWGENWREIKRANAMGSFD
GTND

>A399|A0A1S3XRX0

MGGCFPCFRSSNKESGNVVKEVVKKESFKDGSAAQSIHLSKVNSDKSKSCGSHDPKKDPAIPKDGPTAHIAAQTF
TFRELAAATKNFRPECLLGEGGFGRVYKGRLESTGQVVAVKQLDRNGLQGNREFLVEVLMLSLLHHPNLVNLIGY
CADGDQRLLVYEFMPLGSLEDHLHDLPPDKEPLDWNTRMKIAAGAAKGLEYLHDKANPPVIYRDLKSSNILLDE
GYHPKLSDFGLAKLGPVGDKTHVSTRVMGTYGYCAPEYAMTGQLTLKSDVYSFGVVFLELITGRKAIDNARSHG
EHNLVAWARPLFKDRRKFPKMADPLLQGRYPMRGLYQALAVAAMCLQEQASTRPLIGDVVTALSYLASQTYDP
NAVGAQSNRVGSSTPRSREDRLHSADGVDSPDEHSSAHHGSPSIQRNSPDSRKRDSARDFNTGIELRKIATSGG
SGRKWGLDESERPDSQKNSPVSAGRTRETPRNRDLDRERAVAEAKVWGENWRDKKKTSARGSSFDGMNE

>A400|100783535

MGGCFPCFGSSNKEGSGGGVRVKEVPNKDSSFKEAASVVPQSHLPSRVNSDKSKSRNGADIKKDTPVPKDGPT
AHIAAQTFTFRELAAATKNFRPECLLGEGGFGRVYKGRLESTGQVVAVKQLDRNGLQGNREFLVEVLMLSLLHH
PNLVNLIGYCADGDQRLLVYEFMPLGSLEDHLHDLPPDKEPLDWNTRMKIAAGAAKGLEYLHDKANPPVIYRDL
KSSNILLDEGYHPKLSDFGLAKLGPVGDKTHVSTRVMGTYGYCAPEYAMTGQLTLKSDVYSFGVVFLELITGRKAI
DNTRAHGEHNLVAWARPLFKDRRKFPKMADPLLQGRYPMRGLYQALAVAAMCLQEQAATRPLIGDVVTALTY
LASQTYEPNAANQSNRVGPSTPRIRDDRRSMADGVDSPDRRLGSPSTHRNSPDFRKRDSRDPSAATELGRIDIG
GGSGRKWGLDDNERQESQRDSPVNTARTRETPWNRDLDRERAVAEAKVWGENWREKKKANAMGSFDATN
D

>A401|A0A087G897

MSGCFPCFGSSGKDSAAKDSVKKEVSAKDSSVTQSHHVSLDKSKSRGGPEHKKELTAPKEGATAHIAAQTFTFRE
LAAATKNFRPECLLGEGGFGRVYKGRLESTGQIVAVKQLDRNGLQGNREFLVEVLMLSLLHHPNLVNLIGYCAD
GDQRLLVYEYMPLGSLEDHLHDLPPDKEPLDWNTRMTIAAGAAKGLEYLHDKANPPVIYRDLKSSNILLGDGYH
PKLSDFGLAKLGPVGDKTHVSTRVMGTYGYCAPEYAMTGQLTLKSDVYSFGVVFLELITGRKAIDNARAPGEHN
LVAWARPLFKDRRKFPKMADPSLQGRYPMRGLYQALAVAAMCLQEQAATRPLIGDVVTALTYLASQTFDPNAS
SAQNSRSSGGPPFIRTRDERRSMGDGSSLDSPAETRSRLGSPSTHKNSPDYRRRDMVREVNAGSEAGSETGGG
SGRKWGLSDLEGTESQRGSPASVGRSTRGTPRNRDLDRERAVAEAKVWGENWRERKRATNGPGSFDSTND

>A402|A0A151T743

MGGCFPCFGSSNKEDSGGVRVKVKVKEVPNKDSSFKEAASVLPQSHHSDKSKSSRSGSDTKKEPPVPKDGPTAH
IAAQTFTFRELAAATKNFRPECLLGEGGFGRVYKGRLESTGQVVAVKQLDRNGLQGNREFLVEVLMLSLLHHPN
LVNLIGYCADGDQRLLVYEFMPLGSLEDHLHDLPPDKEPLDWNTRMKIAAGAAKGLEYLHDKANPPVIYRDLKS
SNILLDEGYHPKLSDFGLAKLGPVGDKTHVSTRVMGTYGYCAPEYAMTGQLTLKSDVYSFGVVFLELITGRKAID
NTRAHGEHNLVAWARPLFKDRRKFPKMADPLLQGRYPMRGLYQALAVAAMCLQEQAATRPLIGDVVTALTYL
ASQTYDPNAANQSNRVGPSTPRVRDDRRSMADGVDSPDRGRLGSPSTHRNSPDFRKRDSRDPSALTDLARSET
GGGSGRKWGLDDSERQESQRDSPVNTARARESPWNRDLDRERAVAEAKVWGENWREKKKANAMGSFDAT
ND

>A403|A0A1S3AXF0

MGGCFPCFGSSDEDGSNGVKEATKKDTAKDGSTAQSHHVTRVSSDKSKSRSVSDAKKEATVQKDGTTAHIAAQ
TFTFRELATATKNFRSECLLGEGGFGRVYKGRLESTGQVVAVKQLDRNGLQGNREFLVEVLMLSLLHHPNLVNLI
GYCADGDQRLLVYEFMPLGSLEDHLHDLPPDKEPLDWNTRMKIAAGAAKGLEYLHDKANPPVIYRDLKSSNILL
DEGYHPKLSDFGLAKLGPVGDKTHVSTRVMGTYGYCAPEYAMTGQLTLKSDVYSFGVVFLELITGRKAIDNTRG
PGEHNLVAWARPLFKDRRKFPKMADPLLQGRYPMRGLYQALAVAAMCLQEQAATRPLIGDVVTALTYLASQT
YDPNAAASQSNRMGGSTPRARDERRGFPDGLDSPDERGRGRGSPSNYRNSPDYRKKDFHRELSCGGTELSKID
TGGGSGRKWGLDELERQESLRDSPVYAGRARETPRNRDLNRERAVAEAKVWGENWRERKRANAQGSFDGSQ
E

>A404|A0A2C9UBV6

MGGCFPCFGSSNKEGSGGGAVKEVAKKDSVKEGSVALSHHVGRVSSDKSKSQNGSDPKKEPAIPKDGPTANIA
AQTFTFKELAAATKNFRPECLLGEGGFGRVYKGRLESTGQIVAVKQLDRNGLQGNREFLVEVLMLSLLHHPNLV
NLIGYCADGDQRLLVYEFMPLGSLEDHLHDLPPDKEPLDWNTRMKIAAGAAKGLEYLHDKANPPVIYRDLKSSNI
LLDESYHPKLSDFGLAKLGPVGDKTHVSTRVMGTYGYCAPEYAMTGQLTLKSDVYSFGVVFLELITGRKAIDNNR
APGEHNLVAWARPLFKDRRKFPKMADPFLQGRYPMRGLYQALAVAAMCLQEQAATRPLIGDVVTALTYLASQ

TYDPNTANQSNRVGPSTPRNRDDPRGMADGLDSPGEHQHGGQHGSPHKNSSDYRKRDAARELSTGSELRRSE
TGGGSGPKWGLDDYERQDSQRDGSVNSSRARETPRNRDLDRERAVAAAKVWGENWRDKKRANAQGSFDGT
NE

>A405|A0A2H3YIJ5

MGCFSCFGSPDKEEEEEEEKEKNEEKEGGGDVMRDTSGASSSHHGTRASSDKSKMQNGSSSRNETSVPRDGTS
SRIAAQTFTFRELAAATQDFRLDCLLGEGGFGRVYKGHLESTGQVVAVKQLDRNGLQGNREFLVEVLMLSLLHH
PNLVNLIGYCADGDQRLLVYEFMPLGSLEDHLHDIPPDRKPLDWNMRMKIAAGAAKGLEYLHDKANPPVIYRD
FKSSNILLGEGYFPKLSDFGLAKLGPVGDKTHVSTRVMGTYGYCAPEYAMTGQLTLKSDVYSFGVVFLELITGRKA
IDNTRAAGEHNLVAWARPLFRDRRKFPKMADPKLQGHYPLRGLYQALAVAAMCLQEQAATRPLIGDVVTALTY
LASQTYDPNAAASQSNRAGPSVPRAREDWKSLGFGFDNQHAIQSPHRHSPDFRHRNLFRGPNLGDGGRGDV
VGQSGINLDLAELQIQESQTDSAIRLGKAREAPGISNRDLDRERAVAEARVWGENWRERKRMKEPSNYDSTSE

>A406|AT5G18610

MSGCLPCFGSSAKDAASKDSVKKELSAKDGSVTQSHHISLDKSKSRRGPEQKKELTAPKEGPTAHIAAQTFTFREL
AAATKNFRPECLLGEGGFGRVYKGRLETTGQIVAVKQLDRNGLQGNREFLVEVLMLSLLHHPNLVNLIGYCADG
DQRLLVYEYMPLGSLEDHLHDLPPDKEPLDWSTRMTIAAGAAKGLEYLHDKANPPVIYRDLKSSNILLGDGYHPK
LSDFGLAKLGPVGDKTHVSTRVMGTYGYCAPEYAMTGQLTLKSDVYSFGVVFLELITGRKAIDNARAPGEHNLV
AWARPLFKDRRKFPKMADPSLQGRYPMRGLYQALAVAAMCLQEQAATRPLIGDVVTALTYLASQTFDPNAPS
GQNSRSGSGPPFIRTRDDRRSLGDGSSLDSPAETRSRLGSPATHKNSPDYRRRDMVREVNAGSEGGSETGGGS
GRKWGLSDLEGQESQRGSPASVGRSSRGTPRNRDLDRERAVAEAKVWGENWRERKRATNGPGSFDSTND

>A407|D7LYF0

MSGCLPCFGSSAKDAASKDSVKKELSAKDGSVTQSHHISLDKSKSRRGPEQKKELAAPKEGPTAHIAAQTFTFREL
AAATKNFRPECLLGEGGFGRVYKGRLETTGQIVAVKQLDRNGLQGNREFLVEVLMLSLLHHPNLVNLIGYCADG
DQRLLVYEYMPLGSLEDHLHDLPPDKEPLDWSTRMTIAAGAAKGLEYLHDKANPPVIYRDLKSSNILLGDGYHPK
LSDFGLAKLGPVGDKTHVSTRVMGTYGYCAPEYAMTGQLTLKSDVYSFGVVFLELITGRKAIDNARAPGEHNLV
AWARPLFKDRRKFPKMADPSLQGRYPMRGLYQALAVAAMCLQEQAATRPLIGDVVTALTYLASQTFDPNAPS
GQNSRSGSGPPFIRTRDDRRSLGDGSSLDSPAETRSRLGSPATHKNSPDYRRRDMVREVNAGSEGGSETGGGS
GRKWGLSDLEGQDSQRGSPASVGRSSRGTPRNRDLDRERAVAEAKVWGENWRERKRATNGPGSFDSTND

>A408|LOC17881146

MSGCLPCFGSSAKDAASKDSVKKEASAKDGSVTQSHHASLDKSKSRRGLEQKKELTAPKEGPTAHIAAQTFTFRE
LAAATKNFRPECLLGEGGFGRVYKGRLETTGQIVAVKQLDRNGLQGNREFLVEVLMLSLLHHPNLVNLIGYCAD
GDQRLLVYEYMPLGSLEDHLHDLPPDKEPLDWNTRMTIAAGAAKGLEYLHDKANPPVIYRDLKSSNILLGDGYH
PKLSDFGLAKLGPVGDKTHVSTRVMGTYGYCAPEYAMTGQLTLKSDVYSFGVVFLELITGRKAIDNARAPGEHN
LVAWARPLFKDRRKFPKMADPSLQGRYPMRGLYQALAVAAMCLQEQAATRPLIGDVVTALTYLASQTFDPNA
PSGQNSRSSGGPPFIRTRDDRRSLGDGSSLDSPAETRSRLGSPATHKNSPDFRRRDMVREVNAGSEGGSETGGG
SGRKWGLSDLEGPESQRGSPASVGRTSRGTPRNRDLDRERAVAEAKVWGENWRERKRATNGPGSFDSTND

>A409|LOC18019044

MSGCLPCFGSSAKDAAAKDSVKKEVSAKDGSVTQSHHVILDKSKSRGGPEHKKEITAPKEGPTAHIAAQTFTFRE
LAAATKNFRPECLLGEGGFGRVYKGRLESTGQIVAVKQLDRNGLQGNREFLVEVLMLSLLHHPNLVNLIGYCAD
GDQRLLVYEYMPLGSLEDHLHDLPPDKEPLDWNTRMTIAAGAAKGLEYLHDKANPPVIYRDLKSSNILLGDGYH

PKLSDFGLAKLGPVGDKTHVSTRVMGTYGYCAPEYAMTGQLTLKSDVYSFGVVFLELITGRKAIDNARAPGEHN
LVAWARPLFKDRRKFPKMADPSLQGRYPMRGLYQALAVAAMCLQEQAATRPLIGDVVTALTYLASQTFDPNAS
SGQNSRSGSGPPFIRTRDERRSMGDGSSLDSPAETRSRLGSPATHKNSPDYRRRDMVREVNAGSEAGSETGGG
SGRKWGLSDVEGTESQRGSPASVGRATRGTPRNRDLDRERAVAEAKVWGENWRERKRATNGPGSFDSSND

>A410|PHAVU006G162400g

MGGCFPCFGSSNKEDSGGVRVKEVPNKDSSFKEAASLVPQSHHPSRANTDKSKSSRSGTDAKKETLVPKDGPTP
HIAAQTFTFRELAAATKNFRPECLLGEGGFGRVYRGRLESTGQAVAVKQLDRNGLQGNREFLVEVLMLSLLHHP
NLVNLIGYCADGDQRLLVYEFMPLGSLEDHLHDLPPDKEPLDWNTRMKIAAGAAKGLEYLHDKANPPVIYRDLK
SSNILLDEGYHPKLSDFGLAKLGPVGDKTHVSTRVMGTYGYCAPEYAMTGQLTLKSDVYSFGVVFLELITGRKAID
NTRAHGEHNLVAWARPLFKDRRKFPKMADPLLQGRYPMRGLYQALAVAAMCLQEQAATRPLIGDVVTALTYL
ASQTYDPNAANQSNRVGPSTPRMRDDRRSLADGVDSPDRRLGSPSTHRNSPDFRKRDSRDPSSGTELGRMDT
GGGSGRKWGLDDYERQDSQRDSPVNSARARETPWNRDLDRERAVAEAKVWGENVREKKKANAMGSFDAT
ND

>A411|A0A2P5E436

MGGCFPCFGSSNNEGSGGVKEVTKKDSVKEGSTAQSHHVTRVSSEKSKSRSGSDPKKEPVIPKDGTTAHIAAQT
FTFRELAAATKNFRAECLLGEGGFGRVYKGRLESTGQVVAVKQLDRNGLQGNREFLVEVLMLSLLHHQNLVNLI
GYCADGDQRLLVYEFMPLGSLEDHLHDLPPDKEPLDWNTRMKIAAGAAKGLEYLHDRANPPVIYRDFKSSNILL
EEGYHPKLSDFGLAKLGPVGDKTHVSTRVMGTYGYCAPEYAMTGQLTLKSDVYSFGVVFLELITGRKAIDNTRAQ
GEHNLVAWARPLFKDRRKFPKMADPLLQGRYPMRGLYQALAVAAMCLQEQAATRPLIGDVVTALTYLASQTY
DPNAGSNRVGPSTPWNRDDRRSMGEGLDSPDEHGRGRRNGSPSTHRNSPDFRKRDLARELSTGAELSRIETG
GGSGRKWGLDDSERPDSQRDSPASAGRARETPRNRDLDRERAVAEAKVWGENWREKKRVNAMGGSFDGTN
E

>A412|A0A2P5ERU2

MGGCFPCFGSSNKEGSGGVKEVTKKDSVKEGSTAQSHHVTGVSSDKSKSRSGSDPKKEPVIPKDGPTAHIAAQT
FAFRELAAATKNFRPECLLGEGGFGRVYKGRLESTGQVVAVKQLDRNGLQGNREFLVEVLMLSLLHHQNLVNLI
GYCADGDQRLLVYEFMPLGSLEDHLHDLPPDKEPLDWNTRMKIAAGAAKGLEYLHDKANPPVIYRDLKSSNILL
DEGYHPKLSDFGLAKLGPVGDKTHVSTRVMGTYGYCAPEYAMTGQLTLKSDVYSFGVVFLELITGRKAIDNTRA
QGEHNLVAWARPLFKDRRKFPKMADPLLQGRYPMRGLYQALAVAAMCLQEQAATRPLIGDVVTALTYLASQT
YDPNAASNRVGPSTPWNRDDRRSMGEGLDSPDEHGRGRRNGSPSTHRNSPDFRKRDLARELSTGAELSRIETG
GGSGRKWGLDDLERPDSQRDSPASAGRARETPRNRDLDRERAVAEAKVWGENWREKKRANAMGGSFDGTN
E

>A413|LOC18792881

MGGCFPCFGSSNKEGSGVKEVTKKDSVKEGSAAQSHHVTRVSSDKKSRNGSDHKKEPAIPKDGPTAHIAAQTFT
FRELAAATKNFRAECLLGEGGFGRVYKGRLESTGQVVAVKQLDRNGLQGNREFLVEVLMLSLLHHPNLVNLIGY
CADGDQRLLVYEFMPLGSLEDHLHDFPSDNEPLDWNTRMKIAAGAAKGLEYLHDKANPPVIYRDLKSSNILLDE
GFHPKLSDFGLAKLGPVGDKTHVSTRVMGTYGYCAPEYAMTGQLTLKSDVYSFGVVFLELITGRKAIDNTRGHG
EHNLVAWARPLFKDRRKFPKMADPLLQGRYPMRGLYQALAVAAMCLQEQAATRPLIGDVVTALTYLASQTYD
PNSASGHSNRVGPSTPRHKDERRNMADGLDSPDEPGRGGRHGSPSTHKNSPDYRRRESNRDLNTGVELGRIET
GTGSGRRWGLDGLERQESQRDSPVSAGRARETPRNRDLDRERAVAEAKVWGENWREKKRANAMGSFDGTN
E

>A414|101245581

MGGCFPCFGSSNKETGKDEVKKESFKDASSAAQSIHLTKVNSDKSKSRGSHDPKKDPAIAKDGPTAHIAAQTFTF
RELAAATKNFRPESLLGEGGFGRVYKGRLESTGQVVAVKQLDRNGLQGNREFLVEVLMLSLLHHPNLVNLIGYC
ADGDQRLLVYEFMPLGSLEDHLHDLPPDKEPLDWNTRMKIAAGAAKGLEYLHDKANPPVIYRDLKSSNILLDEG
YHPKLSDFGLAKLGPVGDKTHVSTRVMGTYGYCAPEYAMTGQLTLKSDVYSFGVVFLELITGRKAIDNTRSHGEH
NLVAWARPLFKDRRKFPKMADPLLQGRYPMRGLYQALAVAAMCLQEQASTRPLIGDVVTALTYLASQTYDPN
AVGAQSNRVGSSTPRSREDRLHSVDGVDSPEYTSAHHGSPSIQRNSPDSRKRDSARDFNTGIELRKIATSGGSGR
KWGVDESERPDSQRNSPVSAGRTRETPRNRDLDRERAVAEAKVWGENWREKKKTNARGSSFDGIND

>A415|A0A251RM65

MGGCFPCFGSSKKDGNGVKEVVKNDSVKEGSAAHQSHHVSRVSSGKSKSRVSLDPKKDQTVAKDGQPANIAA
QTFTFRELAAATNNFRPESLLGEGGFGRVYKGRLESTGQVVAVKQLDRNGLQGNREFLVEVLMLSLLHHPNLVN
LIGYCADGDQRLLVYEFMPLGSLEDHLHDLAPDKEPLDWNTRMKIAAGAAKGLEYLHDKANPPVIYRDLKSSNIL
LGEGYHPKLSDFGLAKLGPVGDKTHVSTRVMGTYGYCAPEYAMTGQLTLKSDVYSFGVVFLELITGRKAIDNTRA
PGEHNLVAWARPLFKDRRKFPKMADPLLQGRYPVRGLYQALAVAAMCLQEQAATRPLIGDVVTALTYLASQPY
DPEAARAERASRGSGSGTPRNRADRRNPSDGGLDSLEDSMREPRPHGSPSTYKNSPDYQRINTRSDVDGESSN
AGGGSGRKWGLVDDADSQKNSPANTSRARNRDLDRERAVAEAKVWGENWRDRKRTNTMGGGSFDAATNV

>A416|A0A2I4G9N7

MGGCFPCFGSSNEEGSGVKEVPKKDSVKEGSAHSHHVNRVNSEKSKSRSGSDPKKETAVPKDGPTANIAAQTFT
FRELAAATKNFRQECLLGEGGFGRVYKGRLESTGQVVAVKQLDRNGLQGNREFLVEVLMLSLLHHANLVNLIGY
CADGDQRLLVYEFMPLGSLEDHLHDLLPDKTPLDWNTRMKIAAGAAKGLEYLHDKANPPVIYRDLKSSNILLDEG
YHPKLSDFGLAKLGPVGDKTHVSTRVMGTYGYCAPEYAMTGQLTLKSDVYSFGVVFLELITGRKAIDNSRAPGEH
NLVAWARPLFKDRRKFPKMADPLLQGRYPMRGLYQALAVAAMCLQEQAATRPLIGDVVTALTYLASQSYDPD
AAAAQNNRVGPSTPRNKDERRTMADGLDSPGESGRGGRHGSPSAYKNSPDYRKRDLREGSAGAALARIDTGG
GSGRKAGLDESERRESQRDSPISTGRARETPRNRDLDRERAVAEAKVWGENWREKKRASRMGSFDGTNE

>A417|A0A4P1RMR5

MGGCFPCFGSSKNEDNNNGVKEVVAKKESFKDASIPQSQYPTRVSSDKSKSRSGSDPKKEIPVVKDGPTAHIAA
QTFTFRELAVATKNFRPECLLGEGGFGRVYKGRLESTGQVVAVKQLDRNGLQGNREFLVEVLMLSLLHHPNLVN
LIGYCADGDQRLLVYEFMPLGSLEDHLHDLPPDKEPLDWNTRMKIAAGAAKGLEYLHDKANPPVIYRDLKSSNIL
LDEGYHPKLSDFGLAKLGPVGDKTHVSTRVMGTYGYCAPEYAMTGQLTLKSDVYSFGVVFLELITGRKAIDNTRS
HGEHNLVAWARPLFKDRRKFPKMADPLLQGRYPIRGLYQALAVAAMCLQEQAATRPLIGDVVTALTYLASQTY
DPNASNLSNRLGSSSTPRSRDGRRNMGDSVDSPDRGRLGSPSSYRNSPDYRKSHSRDPSSGTELGRSVSNGGSG
RKWGLDDVERQESHRDSPVNTGRARETPRNRDLDRERAVAEARVWGENWREKKRANAMGSFDATNE

>A418|103627200

MGCFSCFDSPADEQLNPKFGADCGYGGTSAAAAYGAGFGRHGDRGYPDLQQAPMAAPRVEKLSAAAEKARA
KSNGLTKEASVPKDANGNAISAQTFTFRELATATRNFRPECFLGEGGFGRVYKGRLESTGQVVAIKQLNRDGLQ
GNREFLVEVLMLSLLHHQNLVNLIGYCADGDQRLLVYEYMPSGSLEDHLHDIPLDKEALDWNTRMKIAAGAAK
GLEYLHDKANPPVIYRDFKSSNILLDESFHPKLSDFGLAKLGPVGDKSHVSTRVMGTYGYCAPEYAMTGQLTVKS
DVYSFGVVLLELITGRRAIDSTRPHGEQNLVSWARPLFNDRRKLPKMADPRLEGRYPMRGLYQALAVASMCTQ

SEAASRPLIADVVTALSYLASQPYDPSTAFASRRLGGSKPGENGGRVVSRNDETGTSRSLGKDREDSPRDLPAILL
NKDLERERMVAEAKMWGDRERLVAEAKMWGDRERMVAEAKMWGENWRDKRRAENGQGSLDSPPTRNS

>A419|A0A059B5Z1

MGGCFPCFGSSKGNDGSGVKEVSKKDSAKEASTAHSHHVSRVSSDKTKSRNGSDPKKEVPMPKDGPTAHIAAQ
TFTFRELAAATKNFRPESLLGEGGFGRVYKGRLESTGQVVAVKQLDRNGLQGNREFLVEVLMLSLLHHPNLVNLI
GYCADGDQRLLVYEFMPLGSLEDHLHDLPPDKEPLDWNTRMKIAAGAAKGLEYLHDKANPPVIYRDLKSSNILL
DEGFHPKLSDFGLAKLGPVGDKTHVSTRVMGTYGYCAPEYAMTGQLTLKSDVYSFGVVFLELITGRKAIDNSRAP
GEHNLVAWARPLFKDRRKFPKMADPLLQGRYPMRGLYQALAVAAMCLQEQAATRPLIGDVVTALTYLASQTY
DPNSASAQSNRVGPSTPRMKDDRRGTAEGLDSPDGYRGSPSTHRNSPDFRKRDQVRQLTTGAELGRSETGGG
SGRKWGLDDFERVDSHRDSPGSAGRGRETPRNRDLDRERAVAEAKVWGENWRERKRAGAVGSFDSTNE

>A420|A0A0J8B4K8

MGGCFPCWGSSNQAGKSEKEAANKEGGKESSILQSHQLGKVNSDKSKSRNGPDPKKEPTLSKDGSTAHIAAQT
FTFRELAAATKNFRPESLLGEGGFGRVYKGRLESTGQVVAVKQLDRNGLQGNREFLVEVLMLSLLHHPNLVNLIG
YCADGDQRLLVYEYMPLGSLEDHLHDIPPGKEPLDWNTRMKIAAGAAKGLEYLHDKANPPVIYRDLKSSNILLDE
GYHPKLSDFGLAKLGPVGDKTHVSTRVMGTYGYCAPEYAMTGQLTLKSDVYSFGVVFLELITGRKAIDNTRAPGE
HNLVAWARPLFKDRRKFPKMADPLLQGRYPMRGLYQALAVAAMCLQEQAATRPLIGDVVTALTYLASQTYDP
NAPNSHSNRGGPSTPRSRDERRSYGDSLDSPDRRGARLSSPSTQRNSPDFRKRDHNRELSTGAELGRSEMGGG
SGRKWGLDDLERQESQRGSPASAGRTPRNRDLDRERAVAEAKVWGENWREKKRSNAMDSFDSSNE

>A421|11442947

MGGCFPCFGSSNKEDTNAVKEVSKKESFKEASLPQSHHPTRVSSDKSKSKSVSDSKKEKEAPVPKDGATAHIAAQ
TFTFRELAAATKNFRPECLLGEGGFGRVYKGCLESTKQVVAVKQLDRNGLQGNREFLVEVLMLSLLHHPNLVNLI
GYCADGDQRLLVYEFMPLGSLEDHLHDLPPEKEPLDWNTRMKIAAGAAKGLEYLHDKANPPVIYRDLKSSNILLD
EGFHPKLSDFGLAKLGPVGDKTHVSTRVMGTYGYCAPEYAMTGQLTLKSDVYSFGVVFLELITGRKAIDNTRGH
GEHNLVAWARPLFKDRRKFPKMADPLLQGRYPMRGLYQALAVAAMCLQEQAATRPLIGDVVTALTYLASQAF
DPNAANQSNRVGPSTPRLRDDRRSMADSVDSPDRARLGSPSTRRNSPDLRKRDGRDPSELSRIDTGGGSGRK
WGVDDLERHDSQRDSPVNTGRARETPRNRDLDRERAVAEARVWGENWREKKRGNAVGSFDGTNE

>A422|A0A251UIR3

MGGCFPCFGESDKGNGVKEVVKKDAGKEGSVGNQSHHVSRVSSGKSKSRVSLDPKKEQMVVKEGQTANIAA
QTFTFRELATATNNFRAESMLGEGGFGRVYKGRLDSTGQVVAVKQLDRNGLQGNREFLVEVLMLSLLHHPNLV
NLIGYCADGDQRLLVYEFMPLGSLEDHLHDLPPDKEPLDWNTRMKIAAGAAKGLEYLHDKANPPVIYRDLKSSNI
LLNEGFHPKLSDFGLAKLGPVGDKTHVSTRVMGTYGYCAPEYAMTGQLTLKSDVYSFGVVFLELITGRKAIDNTR
APGEHNLVAWARPLFKDRRKFPKMADPLLQGRYPVRGLYQALAVAAMCLQEQAATRPLIGDVVTALTYLASQP
YDPEAARTERASRGSGSGTPRSRADRRNPSEGGLDPFDGSMREPRPLGSPSTGKNSPDYRKINTRPDVGDGESS
NGSGRKWGLVDDPDSQRGSPANTGRARNRDLDRERAVAEAKVWGENWRDRKRTNTRGGGSFDATNE

>A423|LOC21406335

MGGCFPCFGSSNNDGSGVKEVNKKDSVKEGSTAQSHHVTRVSSEKSKSRSGSDPKKESTIPKDGPTAHIAAQTF
TFRELAAATKNFRPECLLGEGGFGRVYKGRLESTGQVVAVKQLDRNGLQGNREFLVEVLMLSLLHHPNLVNLIGY
CADGDQRLLVYEFMPLGSLEDHLHDLPPDKEPLDWNTRMKIAAGAAKGLEYLHDKANPPVIYRDLKSSNILLDE
GYHPKLSDFGLAKLGPVGDKTHVSTRVMGTYGYCAPEYAMTGQLTLKSDVYSFGVVFLELITGRKAIDNTRAPGE

HNLVAWARPLFKDRRKFPKMADPLLQGRYPMRGLYQALAVAAMCLQEQAATRPLIGDVVTALTYLASQTYDP
NAAPPYSNRVGPSTPRSRDDRRSMAEGLDSPDGRRNGSPSTHRNSPDFRKRDLVRELNTGGELGRIETGGGSG
RKWGLDDLDRPDSQRDSPASAGRARETPRTRDLDREREVAEAKVWGENWKKRATAMGSFDGTNE

>A424|A0A1S2XCY8

MGGCFPCFGSSNKEDTSVVKEVSKKETFKESSIPQSHHPTRVSSDKSKSRSVSDSKKETPVPKDGPTAHIAAQTFT
FRELAAATKNFRPECLLGEGGFGRVYKGRLESTGQVVAVKQLDRNGLQGNREFLVEVLMLSLLHHPNLVNLIGY
CADGDQRLLVYEFMPLGSLEDHLHDLPPDKEPLDWNTRMKIAAGAAKGLEYLHDKANPPVIYRDLKSSNILLDE
GYHPKLSDFGLAKLGPVGDKTHVSTRVMGTYGYCAPEYAMTGQLTLKSDVYSFGVVFLELITGRKAIDNTRGHG
EHNLVAWARPLFKDRRKFPKMADPLLQGRYPMRGLYQALAVAAMCLQEQAATRPLIGDVVTALTYLASQTYD
PNAANTSNRVGPSTPRMRDDRRSMTDGVDSPDRGRVGSPSTRRNSPDFRKRDSREPSELSRSDTGGGGSGRK
WGPDDLERQESQRDSPVSTGRGRETPRNRDLDRERAVAEARVWGENWREKKRASAMGSFDATNE

>A425|A0A2H3WZX1

MGCFPCFGSPDKEEEGKSEVKGGGDFKQEAPAAPSSHHVARVSSDKSKARNGSESKKEASIPKEGNAAHIAAQT
FTFRELATVTKNFRPDCLLGEGGFGRVYKGRLENGQVVAVKQLDRNGLQGNREFLVEVLMLSLLHHPNLVNLIG
YCADGDQRLLVYEFMPLGSLEDHLHDLPPDKEPLDWNTRMKIAAGAAKGLEYLHDKANPPVIYRDFKSSNILLDE
GYHPKLSDFGLAKLGPVGDKTHVSTRVMGTYGYCAPEYAMTGQLTVKSDVYSFGVVFLELITGRKAIDNTRPAG
EQNLVAWARPLFKDRRKFPKMADPLLQGCYPMRGLYQALAVAAMCLQEQAATRPLIGDVVTALSYLASQAYD
PNASSQNNKVGPSTSRARDDRKSLGGGNNQHGVQSPHRNSPDFRQRDLIRGATYGADIGRGGASGGSGRKW
GLDELETQESQKDSPVHVGKERETPKNANRDFDRERAVAEAKVWGENWRERKRTYAQGSFDSTNE

>A426|A0A2I4F320

MGGCFPCFGSSNKEGSGVKEVAKKDSVKEGSAAQSHHVNRVSSEKSKSRSGSDPKKEPVVPKDGPTAHIAAQT
FTFRELAAATKNFRPECLLGEGGFGRVYKGRLESTGQVVAVKQLDRNGLQGNREFLVEVLMLSLLHHPNLVNLIG
YCADGDQRLLVYEFMPLGSLEDHLHDLPPDKKSLDWNTRMKIAAGAAKGLEYLHDKANPPVIYRDLKSSNILLDE
GYHPKLSDFGLAKLGPVGDKTHVSTRVMGTYGYCAPEYAMTGQLTLKSDVYSFGVVFLELITGRKAIDNTRAPGE
HNLVAWARPLFKDRRKFPKMADPLLQGHYPIRGLYQALAVAAMCLQEQAATRPLIGDVVTALTYLASQTYDPD
ATAAQSNRVGPSTPRNKDERRIMVDGLDSPDEAGRGGRHGSPSAYKNSPDFRKRDSRESGAGAALARIDIGGG
SGRKAGLDELDRLESQRDSPIRIGRAREAPRNRDLDRERAVAEAKVWGENWRDKRAQCDG

>A427|A0A0D2S2S1

MGGCFPCFGSSNKEANNNNNNGGTTVKELSKKDSTKDSSVPQSQHVNRDKSKSRNGSDSKRELPVVPKDGPT
ANIAAQTFTFRELAAATKNFRPECLLGEGGFGRVYKGRLESTGQVVAVKQLDRNGLQGNREFLVEVLMLSLLHH
PNLVNLIGYCADGDQRLLVYEFMPLGSLEDHLHDLPPDKEPLDWNTRMKIAAGAAKGLEYLHDKANPPVIYRDL
KSSNILLHEGFHPKLSDFGLAKLGPVGDKTHVSTRVMGTYGYCAPEYAMTGQLTLKSDVYSFGVVFLELITGRKAI
DNTRAHGEHNLVAWARPLFKDRRKFPKMADPLLHGHYPMRGLYQALAVAAMCLQEQAATRPLIGDVVTALTY
LASQTYDPNAPGNQSNRVGPSIPRPKEDGLDSPKEHSHRNSPDYRKRNHARESSLGAELGRNETSGGSGRKWG
LDDSERHESHIDSPLNTSRPRERNRDLDRERAVAEAKVWGENLREKKRANAMGSFDGTND

>A428|A0A0D9WFT6

MGCFQCFGSGGKGVEAKKGGGGSSGSGRKDGSSDRRVARVGSDKSRSQGGLDSKKDSIIPRDGNGQHIAAHT
FTFRELAAATKNFRQDCLLGEGGFGRVYKGRLEDGQVVAVKQLDRNGLQGNREFLVEVLMLSLLHHDNLVNLIG
YCADGDQRLLVYEFMPLGSLEDHLHDIPPDKEPLDWNTRMKIAAGAAKGLEFLHDKANPPVIYRDFKSSNILLGE

GYHPKLSDFGLAKLGPVGDKTHVSTRVMGTYGYCAPEYAMTGQLTVKSDVYSFGVVFLELITGRKAIDNTKPLGE
QNLVAWARPLFKDRRKFPKMADPLLEGRFPMRGLYQALAVAAMCLQEQAATRPFIGDVVTALSYLASQTYDP
NAPVQHSRSNASTPRARNHGGVNFDQRRLQSPNHQQSPDLRREGTTTSKYEAEVSRTNSGSGSGRRSGFDSV
DVTGSQMGSPAYAGRKKESSRNTDRQRAIAEAKTWGENSRERKWPNARGSFDSTNE

>A429|A0A2T7DC37

MSCFVCFRSAQDGEAKKPAAADAKDARKDGPPDRGMARVGSDKSRSQGGSDSKKDIIIHRDGNSQNIAAQTF
TFRELAAATKNFRQDCLLGEGGFGRVYKGRLENGQAVAVKQLDRNGLQGNREFLVEVLMLSLLHHTNLVNLIGY
CADGDQRLLVYEFMPLGSLEDHLHDLPPDKEALDWNTRMKIAAGAAKGLEYLHDKASPPVIYRDFKSSNILLGE
GFHPKLSDFGLAKLGPVGDKTHVSTRVMGTYGYCAPEYAMTGQLTVKSDVYSFGVVFLELITGRKAIDNTRPQG
EQNLVAWARPLFKDRRKFPKMADPMLQGRFPMRGLYQALAVAAMCLQEQATTRPHIGDVVTALSYLASQAY
DPNAPVQHSRSNSSTPRARNPAGWNDDQRSMRSPNRHSPDLRRREAARASKYGAEVSRTSSTGDSGRRSGLD
DMDMTGSQVGSPAQTGRRRETPRAADRQRAIAEAKMWGEYSRERSNGHGSFDSTNE

>A430|100382129 K7V5T8_MAIZE

MSCFVCFGSAAHDGESRKPPAAGAGKDVPPDRAVVRVGSDKSRSQGGPESKKDLIIHRDGNSQNIAAHTFTFR
ELAAATKNFRQDCLLGEGGFGRVYKGRLENGQAVAVKQLDRNGLQGNREFLVEVLMLSLLHHTNLVNLIGYCA
DGDQRLLVYEFMPLGSLEDHLHDLPPDKEPLDWNTRMKIAAGAAKGLEYLHDKTSPPVIYRDFKSSNILLGEGFH
PKLSDFGLAKLGPVGDKTHVSTRVMGTYGYCAPEYAMTGQLTVKSDVYSFGVVFLELITGRKAIDNTKTQGEQN
LVAWARPLFKDRRKFPKMADPMLQGRFPMRGLYQALAVAAMCLQEQAATRPHIGDVVTALSYLASQAYDPN
APVQHSRSNASTPRSRNPAAWNGDQRSVRSPNHHHSPDPRRRDPARASKYGAEVSRTSSASDSGRRSGLDDM
DPTGSQVGSPAQTGRRREAPRATDRQRAVGEARMWGENSRERTNGHGSFDSTHE

>A431| **LOC100193908**

MSCFVCFGSAAQDEEPRKPAAAGAGKDAAPDRAVARVGSDKSRSQGGPDSKKDLIIHKDGNSQNIAAQTFTFR
ELAAATKNFRQDCLLGEGGFGRVYKGRLENGQAVAVKQLDRNGLQGNREFLVEVLMLSLLHHTNLVNLIGYCA
DGDQRLLVYEFMPLGSLEDHLHDLPPDKEPLDWNTRMKIAAGAAKGLEHLHDKASPPVIYRDFKSSNILLGEGF
HPKLSDFGLAKLGPVGDKTHVSTRVMGTYGYCAPEYAMTGQLTVKSDVYSFGVVFLELITGRKAIDNTKTQGEQ
NLVAWARPLFKDRRKFPKMADPMLQGRFPMRGLYQALAVAAMCLQEQAATRPHIGDVVTALSYLASQAYDP
NAPVQHVRSNSSTPRARNPAGWNGDQRSVRSPNHHSPDPRRRDAAGASKYGAEVSRTSSTSDSGRRSGLDD
MDLTGSQVGSPAQTGRRREAPRGTDRQRAVAEARTWGENSRERTNGHGSFDSTHE

>A432|102722444

MSCFLCFGSAQDGEAKKPVADAKDPRKDGSADRGVSRVGSDKSRSHGGLDSKKDVVIHREGNNQNIAAQTFT
FRELAAATKNFRQDCLLGEGGFGRVYKGRLETGQAVAVKQLDRNGLQGNREFLVEVLMLSLLHHTNLVNLIGYC
ADGDQRLLVYEFMPLGSLEDHLHDLPPDKEPLDWNTRMKIAAGAAKGLEYLHDKASPPVIYRDFKSSNILLGEGF
HPKLSDFGLAKLGPVGDKTHVSTRVMGTYGYCAPEYAMTGQLTVKSDVYSFGVVFLELITGRKAIDNTKPQGEQ
NLVAWARPLFKDRRKFPKMADPMLQGRFPMRGLYQALAVAAMCLQEQATTRPHIGDVVTALSYLASQTYDP
NAPVQHSRSNSSTPRARNLAGWNDDRRSVRSPNHHSPDLRREAARSSRAEVSRTSSTGDSGRRSGLDDLDMT
GSQMGSPAQTGRKRETSRTADRQRAIAEAKMWGENSRERKQPNGHGSFDSTNE

>A433|A0A0E0FYH4

MSCFLCFGSAQEGEAKKPGADSKDARKDGSADRGVSRVGSDKSRSHGGLDSKKDVVIQRDGNNQNIAAQTFT
FRELAAATKNFRQDCLLGEGGFGRVYKGRLETGQVVAVKQLDRNGLQGNREFLVEVLMLSLLHHTNLVNLIGYC

ADGDQRLLVYEFMPLGSLEDHLHDLPPDKEPLDWNTRMKIAAGAAKGLEYLHDKASPPVIYRDFKSSNILLGEGF
HPKLSDFGLAKLGPVGDKTHVSTRVMGTYGYCAPEYAMTGQLTVKSDVYSFGVVFLELITGRKAIDNTKPQGEQ
NLVAWARPLFKDRRKFPKMADPMLQGRFPMRGLYQALAVAAMCLQEQATTRPHIGDVVTALSYLASQTYDP
NAPVQHSRSNSSTPRARNLAGWNEDRRSVRSPNHHSPDLRREAARSSRAEVSRTSSTGDSGRRSGLDDLDMT
GSQMGSPAQTGRKRETPRTADRQRAIAEAKTWGENSRERKHPNGHGSFDSTNE

>A434|I1HK35

MGCLPCFGSGGKGEAKKGRAGARKDVPPDRRGTLAGSDKSKPQGGLDSKKDAVIPREGNNQHIAAHTFTFREL
AAATKNFRQDCLLGEGGFGRVYKGRLDNSQVVAVKQLDRNGLQGNREFLVEVLMLSLLHHDNLVNLIGYCADG
DQRLLVYEYMPLGSLEDHLHDIPPEKEPLDWNTRMKIAAGAAKGLEYLHDKASPPVIYRDFKSSNILLGEGFHPKL
SDFGLAKLGPVGDKTHVSTRVMGTYGYCAPEYAMTGQLTVKSDVYSFGVVFLELITGRKAIDNTKPHGEQNLVA
WARPLFKDRRKFPKMADPSLQGRFPMRGLYQALAVAAMCLQEQAATRPFIGDVVTALSYLASQAYDPNAPTQ
HNRSNASTPRARDRGSGNSDQRRINSPNHHSPDVRRREATTTSKYEAEVSRNSSGGGSGRRSALDDGDATGSQ
VGSPAHAGRRREASRTSDRQRAIAEAKTWGENSRERKWPNARGSFDSTNE

>A435|A0A446JZQ0

MGCLPCFGSSGKGEPAKKGGARKDVPSDRRATGVGSDKPKPQGLLDSKKDTVIPREGNNQHIAAHTFTFRELA
AATKNFRQDCLLGEGGFGRVYRGRLDNGQAVAVKQLDRNGLQGNREFLVEVLMLSLLHHDNLVNLIGYCADG
DQRLLVYEYMPLGSLEDHLHDIPPEKEPLDWNTRMKIAAGAAKGLEYLHDKASPPVIYRDFKSSNILLGEEFHPKL
SDFGLAKLGPVGDKTHVSTRVMGTYGYCAPEYAMTGQLTVKSDVYSFGVVFLELITGRKAIDNTKPHGEQNLVA
WARPLFKDRRKFPKMADPALQGRFPMRGLYQALAVAAMCLQEQAATRPFIGDVVTALSYLASQAYDPNAPTQ
HSRSNASTPRARDRGSVNGDQRRIRSPNHHSPDLRRKEATTTSKYEAEVSRNSSGGGSGRRSGLDDRDVTGSQ
QGSPAQAGKRRETSRTSERQRAIAEAKTWGENSRERKWPNARGSFDSTNE

>A436|A0A3B5Y202

MGCLPCFGSSGKGEPAKKGGARKDVPSDRRATGVGSDKPKPQGLLDSKDAVIPREGNNQHIAAHTFTFRELAA
ATKNFRQDCLLGEGGFGRVYRGRLDNGQAVAVKQLDRNGLQGNREFLVEVLMLSLLHHDNLVNLIGYCADGD
QRLLVYEYMPLGSLEDHLHDIPPEKEPLDWNTRMKIAAGAAKGLEYLHDKASPPVIYRDFKSSNILLGEEFHPKLS
DFGLAKLGPVGDKTHVSTRVMGTYGYCAPEYAMTGQLTVKSDVYSFGVVFLELITGRKAIDNTKPHGEQNLVA
WARPLFKDRRKFPKMADPALQGRFPMRGLYQALAVAAMCLQEQAATRPFIGDVVTALSYLASQAYDPNAPTQ
HSRSNASTPRARDRGSVNGDQRRIRSPNHHSPDMRRKEVTTTSKYEAEVSRNSSGGGSGRQSGLDDRDVTGS
QQGSPARAGKRRETSRTSERQRAIAEAKTWGENSRERKWPNARGSFDSTNE

>A437|A0A0E0H006

MGCFPCFDSRMGEEEEEELYYGKGGARGGGNGGGALSAAAAAASSSSGVGGGGWEGTSTAAPRVEKISAGAR
GRGNATVKKELSALKDANGNVISAQTFTFRQLAAATRNFREECFIGEGGFGRVYKGRLDGTGQIVAIKQLNRDG
TQGNKEFLVEVLMLSLLHHQNLVNLVGYCADGDQRLLVYEYMPLGSLEDHLHDLPPDKVPLDWNTRMKIAAG
AAKGLEYLHDKAQPPVIYRDFKSSNILLGEDFHPKLSDFGLAKLGPVGDKSHVSTRVMGTYGYCAPEYAMTGQLT
VKSDVYSFGVVLLELITGRKAIDSTRPHVEQNLVSWARPLFNDRRKLPKMADPGLEGRYPMRGLYQALAVASM
CIQSEAASRPLIADVVTALSYLASQKYDPNTTPSSKKAGGGEAGRALSRNDEAGSSGHKSPSSKDSPREQQLPGIL
NDRERMVAEAKMWGENWREKRRAAATTSSNAQVSLDSPTETG

>A438|A0A3B6EPA4

MSCFSCFGPALEAEGGKPGPDAKDPRAKDGAAPDRTGSDKLRLQGGSDPKNNHLTIPRDGSSQNIAAQIFTFRE
LAAATKNFRQDCMLGEGGFGRVYKGRLESGQAVAVKQLDRDGLQGNREFLVEVLMLSLLHHTNLVNLIGYCAD
GDQRLLVYEFMPLGSLEDHLHDVPPEKEPLDWNTRMKIAAGAAKGLEHLHDKASPPVIYRDFKSSNILLGEGFHP
KLSDFGLAKLGPVGDNTHVSTRVMGTYGYCAPEYAMTGQLTVKSDVYSFGVVFLELITGRKAIDNTKPQGEQNL
VAWARPLFKDRRKFPKMADPMLQGRFPMRGLYQALAVAAMCLQEQATTRPHIGDVVTALSYLASQTYDPNA
PTQHTRSNSSTPRARNVGGRNSEQRNGRSPNHLSPRTSKHGGEVSRTSSTGGDSGRRSGLDEIDMAGSQAGSP
AQTGRKRETPRIADRQRAIADAKIWGENSRERKRPNGSFDSTNE

>A439|C5YEV8

MGCFPCFGSTSDEELKYYGALGGNGGGVGRAAASSSSSSAAGGGGRAEEAVVAPPRVARDHAGADKARAKG
NAGSKKELSVLRDASGNVISAQTFTFRQLAAATKNFRDECFIGEGGFGRVYKGRLDMGQVVAIKQLNRDGNQG
NKEFLVEVLMLSLLHHQNLVNLVGYCADGDQRLLVYEYMPLGSLEDHLHDLPPDKEPLDWNTRMKIAAGAAKG
LEYLHDKAQPPVIYRDFKSSNILLGEGFHPKLSDFGLAKLGPVGDKSHVSTRVMGTYGYCAPEYAMTGQLTVKSD
VYSFGVVLLELITGRKAIDSTRPASEQNLVSWARPLFNDRRKLPKMADPGLEGRFPTRGLYQALAVASMCIQSEA
ASRPLIADVVTALSYLANQIYDPSLAHTSKKAGGSDQRNRVGDSGRVLSKNDDAGSSGHRSPSKDRADSPREQF
PGAANRGQDRERMVAEAKMWGENWREKRRAAQGSLDSPTGGG

>A440|EMS62361.1

MSCFSCFGPALEAEGGKPGPDAKDPRAKDGAAPDRAGSDKLRLQGGSDPKNNHLTIPRDGSSQNIAAQIFTFRE
LAAATKNFREDCMLGEGGFGRVYKGRLESGQAVAVKQLDRNGLQGNREFLVEVLMLSLLHHTNLVNLIGYCAD
GDQRLLVYEFMPLGSLEDHLHDVPPEKEPLDWNTRMKIAAGAAKGLEHLHDKASPPVIYRDFKSSNILLGEGFHP
KLSDFGLAKLGPVGDNTHVSTRVMGTYGYCAPEYAMTGQLTVKSDVYSFGVVFLELITGRKAIDNTKPQGEQNL
VAWARPLFKDRRKFPKMADPMLQGRFPMRGLYQALAVAAMCLQEQATTRPHIGDVVTALSYLASQTYDPNA
PTQHTRSNSSTPRARNVGGRNSEQRNGRSPNHLSPRTSKHGGEVSRTSSTGGDSGRRSGLDEMDMAGSQAGS
PAQTGRKRETPRIADRQRAIADAKIWGENSRERKRPNGSFDSTNE

>A441|K3Y6X9

MGCFPCFGSGQEDDELGYYGGNGGAAGWAAAAASSSAAAAAAAAVGGGAEAAVAAPPRAERSPTGSNKSK
AKGNGSKKELAVLKDANGNVISAQTFTFRQLAAATKNFRDECFIGEGGFGRVYKGRLDGTGQVVAIKQLNRDG
NQGNKEFLVEVLMLSLLHHQNLVNLVGYCADGDQRLLVYEYMPLGSLEDHLHDLPPGKEALDWNTRMKIAAG
AAKGLEYLHDKADPPVIYRDFKSSNILLGEGFHAKLSDFGLAKLGPVGDKSHVSTRVMGTYGYCAPEYAMTGQLT
VKSDVYSFGVVLLELITGRKAIDSTRPAAEQNLVSWARPLFNDRRKLPKMADPGLEGRFPTRGLYQALAVASMCI
QSEAASRPLIADVVTALSYLANQIYDPNTASTSKKGGGDQRSRVSDSGRALSRNDDTGSSGHRSPSKDLDDSPRE
RHATGTAHKGERERMVAEAKMWGENWREKQRAAQGSLDSPTGGG

>A442|A0A0D9ZK40

MGCFPCFDSRMGEEEEEELYYGKGGARGGGNGGGALSAAAAAAASSSGVGGGGWEGTSTAAPRVEKISAGA
RGRGNATVKKELSALKDANGNVISAQTFTFRQLAAATRNFREECFIGEGGFGRVYKGRLDGTGQIVAIKQLNRD
GTQGNKEFLVEVLMLSLLHHQNLVNLVGYCADGDQRLLVYEYMPLGSLEDHLHDLPPDKVPLDWNTRMKIAA
GAAKGLEYLHDKAQPPVIYRDFKSSNILLGEDFHPKLSDFGLAKLGPVGDKSHVSTRVMGTYGYCAPEYAMTGQ
LTVKSDVYSFGVVLLELITGRKAIDSTRPHVEQNLVSWARPLFNDRRKLPKMADPGLEGRYPMRGLYQALAVAS

MCIQSEAASRPLIADVVTALSYLASQKYDPNTTPSSKKAGGGEAGRALSRNDEAGSSGHKSPSSKDSPRELLLPGI
LNDRERMVAEAKMWGENWREKRRAAATTSSNAQLAGVS

>A443|A0A446L2R0

MGCFPCFDSGADGELLYPKQGGGGGGNGTGGRTVSAASSSGVGAREERPMVPPRVEKLPAGAEKARAKGNA
GMKELSDLRDANGNVLSAQTFTFRQLTAATRNFREECFIGEGGFGRVYKGRLDGGQVVAIKQLNRDGNQGNK
EFLVEVLMLSLLHHQNLVNLVGYCADGEQRLLVYEYMPLGSLEDHLHDLPPDKEPLDWNTRMKIAAGAAKGLE
YLHDKAQPPVIYRDFKSSNILLGDDFHPKLSDFGLAKLGPVGDKSHVSTRVMGTYGYCAPEYAMTGQLTVKSDV
YSFGVVLLELITGRKAIDSTRPHGEQNLVSWARPLFNDRRKLPKMADPGLQGRYPMRGLYQALAVASMCIQSEA
ASRPLIADVVTALSYLASQIYDPNAIHASKKAGGDQRSRVSDSGRTLLKNDEAGSSGHKSDRDDSPREPPPGILND
RERMVAEAKMWGANLREKTRAACFRLAGKDELGAFRWC

>A444|D8RXH9

MGCFACFGSSKKQASGRKGLRREDSAGSFNKTASDKLKSKGFFDAKKEGSRGDESKIAAQTFTFRELASATKNFR
PECMLGEGGFGRVYKGRLDSGQVVAVKQLDRNGLQGNREFLVEVLMLSLLHHPNLVNLIGYCADGDQRLLVYE
FMQLGSLEDHLHDVPADKEPLGWNTRMKIAAGAARGLEYLHDKANPPVIYRDFKSSNILLGEGHHPKLSDFGLA
KLGPVGDKTHVSTRVMGTYGYCAPEYAMTGQLTLKSDVYSFGVVLLELITGRKAIDNARPAGEHNLVAWARPLF
KDRRKFPSMADPLLQGHYPMRGLYQALAVAAMCLQEQATTRPHIGDVVTALNYLASQTYDPGLYPANNSRFA
PSTPSRDRREREKKSSGTEEKRLRDRGARSSLPSAQGTPELRSKDPRFRAANGGGGNSSDDLEGGHRDSPTHAA
TRHAKAVATREQRDRAWGESNNNSRDRKKGSFGYDG

>A445|PAN37847.1

MGCFPCFGPGREEELEYYGGNGGAAGWAAASSSAAAAGGGGAEAAVEVPPRAERIPAGVDKSRAKGNAGSK
KELAVLKDANGNVISAQTFTFRQLAAATKNFRDECFIGEGGFGRVYKGRLDTTGQVVAIKQLNRDGNQGNKEFL
VEVLMLSLLHHQNLVSLVGYCADGDQRLLVYEYMPLGSLEDHLHDLPPGKEPLDWNTRMKIAAGAAKGLEYLH
DKAQPPVIYRDFKSSNILLGEGFHAKLSDFGLAKLGPVGDKSHVSTRVMGTYGYCAPEYAMTGQLTVKSDVYSF
GVVLLELITGRKAIDSTRPASEQNLVSWARPLFNDRRKLPKMADPGLEGRFPTRGLYQALAVASMCIQSEAASRP
LIADVVTALSYLANQIYDPSPVNITKKGGGDQRSRVGDSERAVSRNDGTGSSGHRSPSKDRDDSPREHATGTAN
KGLERERMVAEAKMWGENWREKQRAAQGSLDSPKGGG

>A446|A0A445D9T9

MGCFSCFDSREDQKLNPHGKHDHDHDHIRKQHQQQQQHPHPSNPALPSHISRLPSGADKLRSRSNGGSKREL
PAPKDGPGVQIAAQTFTFRELAAATKNFRPESFIGEGGFGRVYKGKLESTGQIVAVKQLDRNGLQGNREFLVEVL
MLSLLHHPNLVNLIGYCADGEQRLLVYEFMPLGSLEDHLHDLPPDKEPLDWNTRMKIAAGAAKGLEYLHDKAN
PPVIYRDFKSSNILLDEGYHPKLSDFGLAKLGPVGDKSHVSTRVMGTYGYCAPEYAMTGQLTVKSDVYSFGVVFL
ELITGRKAIDSTQPHGEQNLVTWARPLFNDRRKFSKLADPRLQGRFPMRGLYQALAVASMCIQESAATRPLIGD
VVTALSYLANQAYDPNNTGHGYRGSGDDKRNRDDKGARLLKNDEAGGSGHRWDLEGSEKDESPRETARMLN
RDLDRERAVAEAKMWGENWREKRRQSLEPGSFDATNV

>A447|A0A446MC27

MGCFPCFDSGSDGELLYPKQGGGNGTGGRIAPAASSSGVGAREERPMVPPRVEKLPAGAEKARAKGNAGMKE
LSDLRDANGNVLSAQTFTFRQLTAATRNFREECFIGEGGFGRVYKGRLDGGQVVAIKQLNRDGNQGNKEFLVE
VLMLSLLHHQNLVNLVGYCADGEQRLLVYEYMPLGSLEDHLHDLPPDKEPLDWNTRMKIAAGAAKGLEYLHDK
AQPPVIYRDFKSSNILLGDNFHPKLSDFGLAKLGPVGDKSHVSTRVMGTYGYCAPEYAMTGQLTVKSDVYSFGV

VLLELITGRKAIDSTRPHGEQNLVSWARPLFNDRRKLPKMADPGLQGRYPMRGLYQALAVASMCIQSEAASRPL
IADVVTALSYLASQIYDPNAIHASKKAGGDLRSRVSDSGRTLLKNDEAGSSGHKSDRDDSPREPPPGILNDRERM
VAEAKMWGANLREKTRAACFRLAGKDELGAFRWC

>A448|K3Z5W4

MGCLPCFGSAGEGAAKKGGARKDGSSDRRVTRVGSDKSKPQGGSGSKKDAVILREGNNQHIAAHTFTFRELAA
ATKNFRQDCLLGEGGFGRVYKGRLENGQVVAVKQLDRNGLQGNREFLVEVLMLSLLHHDNLVNLIGYCADGD
QRLLVYEFMPLGSLEDHLHDIPPDKEPLDWNTRMKIAAGAAKGLEYLHDKASPPVIYRDFKSSNILLGEGFHPKLS
DFGLAKLGPVGDKTHVSTRVMGTYGYCAPEYAMTGQLTVKSDVYSFGVVFLELITGRKAIDNGKPHGEQNLVA
WARPLFKDRRKFPKMADPLLQGRFPMRGLYQALAVAAMCLQEQAATRPFIGDVVTALSYLASQTYDPNAPVQ
HNRSNSSTPRASRGGGSNDQRRLRSPNHHSPDLRRREATTGPKYEAEVSRTNSGSGSGRQSGLDDVDMTVSQL
GSPGHGGRKRESPRTAERQGAIAEAKTWGENSRGRN

>A449|100279542

MGCLPCFGSAGEGAAKEGGARKDGTSDLRVTRVESDKSKPQGGLDSKKDAVILREGNNQHIAAHTFTFRELAA
ATKNFRQDCLLGEGGFGRVYKGRLENGQVVAVKQLDRNGLQGNREFLVEVLMLSLLHHDNLVNLIGYCADGD
QRLLVYEFMPLGSLEDHLHDIPPDKEPLDWNTRMKIAAGAAKGLEYLHDKASPPVIYRDFKSSNILLGEGFHPKLS
DFGLAKLGPVGDKTHVSTRVMGTYGYCAPEYAMTGQLTAKSDVYSFGVVFLELITGRKAIDNTKPHGEQNLVA
WARPLFKDRRKFPKMADPSLQGCFPMRGLYQALAVAAMCLQEQAATRPFIGDVVTALSYLASHTYDPNAPAQ
HNRSNSSTPRVSRGGGSNDQRRLRSSNHHSPDLRREVTTASRYDDEVSRANSGTGSGRRSGLDDADMSGSQL
GSPAHTGRKRGSPRTSESQHAIAEAKTYGENSRGRK

>A450|C5YX81

MGCLPCFGSAGEGAAKKVGARKDGSSDRRVTRVESDKSKAHGGPDSKKDAVILRDGSNQHIAAHTFTFRELAA
ATKNFRQDCLLGEGGFGRVYKGRLENGQVVAVKQLDRNGLQGNREFLVEVLMLSLLHHDNLVNLIGYCADGD
QRLLVYEFMPLGSLEDHLHDIPPEKEPLDWNTRMKIAAGAAKGLEYLHDKASPPVIYRDFKSSNILLGEGFHPKLS
DFGLAKLGPVGDKTHVSTRVMGTYGYCAPEYAMTGQLTVKSDVYSFGVVFLELITGRKAIDNTKPHGEQNLVA
WARPLFKDRRKFPKMADPLLQGRFPMRGLYQALAVAAMCLQEQAATRPFIGDVVTALSYLASQTYDPNAPVQ
HNRSNSSTPRVSRGGGSNDQRRLRSPNHHSPDLRREATAASKYEAEVSRTNSGSGSGRRSGLDDVDMSGSQLG
SPAHAGRKRGSPRTAESQRAIAETKTCGEKSRGRK

>A451|C5YX82

MGCLPCFGSAGEGAAKKVGARKDGSSDRRVTRVESDKSKAQGGPDSKKDAVILRDENNQHIAAHTFTFRELAA
ATKNFRQDCLLGEGGFGRVYKGRLENGQVVAVKQLDRNGLQGNREFLVEVLMLSLLHHDNLVNLIGYCADGD
QRLLVYEFMPLGSLEDHLHDIPPEKEPLDWNTRMKIAAGAAKGLEYLHDKASPPVIYRDFKSSNILLGEGFHPKLS
DFGLAKLGPVGDKTHVSTRVMGTYGYCAPEYAMTGQLTVKSDVYSFGVVFLELITGRKAIDNTKPHGEQNLVA
WARPLFKDRRKFPKMADPLLQGRFPMRGLYQALAVAAMCLQEQAATRPFIGDVVTALSYLASQTYDPNAPVQ
HNRSNSSTPRVSRGGGSNDQRRLRSPNHHSPDLRREATAASKYEAEVSRTNSGSGSGRRSGLDDVDMSGSQLG
SPAHAGRKRGSPRTAESQRAIAETKTCGEKSRGRK

>A452|A0A0S3SKZ1

MGCFSCFDSREDEKLNPNPHQENHHHLHHHHDHDLNPPIPSRISRLPSASAGADKLRSSSNGNGNNGDSKREL
AALKDGPAVQIAAQTFTFRELAAATKNFRPQSFLGEGGFGRVYKGRLETTGQAVAVKQLDRNGLQGNREFLVE
VLMLSLLHHPNLVNLIGYCADGDQRLLVYEFMPLGSLEDHLHDLPPEKEPLDWNTRMKIAAGAAKGLEYLHDKA

NPPVIYRDFKSSNILLDEDYHPKLSDFGLAKLGPVGDKSHVSTRVMGTYGYCAPEYAMTGQLTVKSDVYSFGVVF
LELITGRKAIDSTRPHGEQNLVTWARPLFNDRRKFPKLADPELHGRYPMRGLYQALAVASMCIQEQAAARPLIG
DVVTALSFLANQAYDHKGGGDDKRNRDDQGGRILKNDQGGGSGRRWDLEGCEKDESPRETARMLNNRDLD
RERAVAEAKMWGENWREKRRQSAQGSFDGSNA

>A453|I1IXA5

MGCFSCFDSSSSEELGYPKHGGGGGSSTGGRAAAASSSGVGAREERPMVAPRVDKLPAGVEKARTKGNASMK
ELSVLRDANGNALSAQTFTFRQLTAATRNFREECFIGEGGFGRVYKGRLDGSQVVAIKQLNRDGNQGNKEFLVE
VLMLSLLHHQNLVNLVGYCADGEQRLLVYEYMALGSLEDHLHDLPPDKESLDWNTRMKIAAGAAKGLEYLHDK
AQPPVIYRDFKSSNILLGDDFHPKLSDFGLAKLGPVGDKSHVSTRVMGTYGYCAPEYAMTGQLTVKSDVYSFGV
VLLELITGRKAIDSTRPHGEQNLVSWARPLFSDRRKLPKMADPGLQGRYPSRGLYQALAVASMCIQSEAASRPLI
ADVVTALSYLAAQTYDPNAIHASKKAGSDQRSRVGDSGRVLLKNDEAGSFGDKSDRDDSPRERPLGILNDRERM
VAEAKMWGTNLREKTRAAANAQGNLDSPT

>A454|A0A314UFY7

MGCFPCFDSKEEEKLNNPVNEIDDQKQGQPTVSSNISRLPSGADRMRSRSNGVSRRDLGSKLPDLKDVPGVQIA
AQIFTFRELVTATKNFRPESFIGEGGFGRVYKGRLESSGQVVAVKQLDRNGLQGNREFLVEVLMLSLLHHPNLVN
LIGYCADGDQRLLVYEFMPLGSLEDHLHDLPADREPLDWNTRMKIASGAAKGLEYLHDKANPPVIYRDFKSSNIL
LEEGFHPKLSDFGLAKLGPTEDKSHVSTRVMGTYGYCAPEYAMTGQLTVKSDVYSFGVVFLELITGRKSIDSNRP
HGEQNLITWARPLFNDRRKFSKLADPRLQGRYPMRGLYQALAVASMCIQEQAATRPLIGDVVTALSYLANQSY
DPNTASGHGHRGSGEKDERRHRDERGGRILKNEEGGGSGRRWDLDGSEKDDSPKETARMLNNRDLDRERAV
AEAKMWGENWREKRRQNAQGSFDGTNL

>A455|A0A2K3NLH7

MGCFSCFDSKEDEKLNPNPQQQQQQTHHHDHNLPSHVSRLPSAPSGPDKLRSTSNGGASKREFTAALKDGPP
AQIAAQTFTFRELAAATKNFRPQSFLGEGGFGRVYKGRLESTGQAVAVKQLDRNGLQGNREFLVEVLMLSLLHH
PNLVSLIGYCADGDQRLLVYEFMPLGSLEDHLHDLPPDKEPLDWNTRMKIAAGAAKGLEYLHDKANPPVIYRDF
KSSNILLDEGYHPKLSDFGLAKLGPVGDKSHVSTRVMGTYGYCAPEYAMTGQLTVKSDVYSFGVVFLELITGRKAI
DSTRPHGEQNLVTWARPLFNDRRKFPKLADPRLQGRYPMRGLYQALAVASMCIQEQAAARPLIGDVVTALSYL
ANQAHDTNNAGIGNNKGTGDDKRSRDDKGGRILQNDEGGGSGRRWDLEGSEKDESPRETARMLNRDLDRER
AVAEAKMWGENWREKRRQSVQGSFDASNA

>A456|A0A5E4EW25

MGCFPCFDSKEEEKLNNPVNEIEDRKQGQPTVSSNISRLPSGADRMRSRSNGGSRRDLGSKLPDLKDVPGVQIA
AQIFTFRELVTATKNFRPESFIGEGGFGRVYKGQLESSGQVVAVKQLDRNGLQGNREFLVEVLMLSLLHHPNLVN
LIGYCADGDQRLLVYEFMPLGSLEDHLHDLPPDREPLDWNTRMKIASGAAKGLEYLHDKANPPVIYRDFKSSNIL
LEEGFHPKLSDFGLAKLGPTEDKSHVSTRVMGTYGYCAPEYAMTGQLTVKSDVYSFGVVFLELITGRKSIDSNRP
HGEQNLITWARPLFNDRRKFSKLADPRLQGRYPMRGLYQALAVASMCIQEQAATRPLIGDVVTALSYLANQSY
DPNMASGHGHRGSGEKDERRHRDERGGRILKNEEGGGSGRRWDLDGSEKDDSPKETARMLNRDLDRERAVA
EAKMWGENWREKRRQSAQGSFDGTNL

>A457|A0A5B6VG13

MIYNEEEKLNTVNESNDPKQAQPTVSSNISRLSSGGDRLRSRSNGGSKRELPCPRDGPGVQIAAHIFSFRELAAAT
KNFRPESFLGEGGFGHVYKGQLESTGQVVAVKQLDRNGLQGNREFLVEVLMLSLLHHPNLVNLIGYCADGDQR

LLVYEFMPLGSLEDHLHGNILMDAHKDCDLPPGKEPLDWNTRMKIAAGAAKGLEYLHDKANPPVIYRDFKSSNI
LLDEGFHPKLSDFGLAKLGPVGDKSHVSTRVMGTYGYCAPEYAMTGQLTVKSDVYSFGVVFLELITGRKAIDSTR
PHGEQNLITWARPLFTNRRKLSKLADPLLQGRFPMRGLYQALAVASMCIQEEAAARPHIGDVVTALSYLANQAY
EPNASGHGHSGSGETDEKRYRDDRGGRVSKNDEGGVSGCRWDLEGSEKDDSPKETARMLNRDLDRERAVAE
AKMWGENWREKRRQSAQGSSDGSNG

>A458|A0A2H3XZG9

MGCFPCFDSKEAAQLNPGEGRDDRREELPMVAPRVEKLSSGADRSKTRSNLDLKRESLRPNEGSGFNISAQTFT
FRELAVATKNFRPECFLGEGGFGRVYKGCLESTGQVVAVKQLDRNGLQGNREFLVEVLMLSLLHHPNLVNLIGY
CADGDQRLLVYEYMPLGSLEDHLHDLPPEKEPLDWNRRMKIAAGAAKGLEYLHDKANPPVIYRDFKSSNILLDE
GFHPKLSDFGLAKLGPVGDKSHVSTRVMGTYGYCAPEYAMTGQLTVKSDVYSFGVVLLELITGRKAIDSTRAHGE
QNLVSWARPMFNDRRKLPKLADPKLQGRYPMRGLYQALAVASMCIQEEAASRPLIADVVTALAYLASQAYDPN
AVPNSNNRSGGERRSRTSDEKGGRMQAKNNDGGSGCKWELDVEKEDSPRETVGILNRDFDRERAVAEAKM
WGENWREKRRANANAQGSVDAASGNG

>A459|A0A1U8EJL0

MGCFSCFDSKEEVKLNPQKDRDDRKEVHLTAPSNISRLSSGADRLKTRSTNGSKREFLGLKDAPDVQIAAHTFTF
RELAAATNNFRPESFIGEGGFGRVYKGQLPSGQVVAVKQLDRNGLQGNREFLVEVLMLSLLHHPNLVNLIGYCA
DGEQRLLVYEFMPLGSLEDHLHDLPPDKEPLDWNTRMKIASGAAKGLEYLHDKANPPVIYRDFKSSNILLEENFF
PKLSDFGLAKLGPTGDKSHVSTRVMGTYGYCAPEYAMTGQLTVKSDVYSFGVVFLELITGRKAIDSTRPQGEQNL
VTWARPLFNDRRKFAKLADPYLQGQFPMRGLYQALAVASMCIQEQAAARPLIGDVVTALSYLANQAYDPGAV
PGQSHRFGADSSDTRNKDDRGGRIFRNEDGAGGSGRKWDLDGGSEKEDSPRETARMLNRDLDRERAVAEAK
MWGENWREKRRQNAQGSFDRSNG

>A460|M0T8T3

MGCFPCFESEEAAQLSHVNDCDRKREEQPMVAPPVEKLSSGDDRIRTRNDVNEKKESLGPKEGSDFAISAHTFT
FRELSYATGNFRAESLLGEGGFGRVYRGRLETTGQVVAVKQLDRNGLQGNREFLVEVLMLSMLHHPNLVNLIGY
CADGDQRLLVYEYMSMGSLEDHLHDLPPDKEPLDWNTRMKIAAGAAKGLEYLHNKASPPVIYRDLKSSNILLDK
GFHPKLSDFGLAKLGPVGDKSHVSTRVMGTYGYCAPEYAMTGQLTVKSDIYSFGVVLLELITGRKAVETTKSHAE
QNLVSWARPMFNDRRKLPSLADPKLQGRYPMRGVYQALAVASMCIQEEAASRPAIADVVTALSYLASQAYDP
GTCPTSNNRPGGERRNRSADEGGGRTPALNSDAETAHNWQMNGENTSKETSANVRQGFDRERALAEAKIWG
ENWREKTQAKANAEENFHVASAIG

>A461|LOC110639117

MGCFPCFDSREEETLNPRKESDDRKQSLPTESSNISKLSSGADRLGSRSNGRSKRELPVAKDGLTGGNIAAQTFTF
RELAAATKNFRPESFLGEGGFGRVYKGLLESTGQVVAVKQLDRNGLQGNREFLVEVLMLSLLHHPNLVNLIGYCA
DGDQRLLVYEFMPLGSLEDHLHDLPPDKEPLDWNTRMRIAAGAAKGLEYLHDKANPPVIYRDFKSSNILLDEGF
HPKLSDFGLAKLGPTGDKSHVSTRVMGTYGYCAPEYAMTGQLTVKSDVYSFGVVFLELITGRKAIDSTRPHVEQ
NLVTWARPLFNDRRKFSKLADPRLQGRYPMRGLYQALAVASMCIQEQAAARPLIGDVVTALSYLANQAYEPNS
AAHGYRGSGDKDERRNKDERGGQLSKSEEGGGSGRRWDLDGSEKEDSPKETARMLNRDLDRERAVAEAKM
WGENWREKRRQSAQGSFDGSNG

>A462|LOC112033137

MGCFSCFDSKEEEKLNTQKPSDDRKQALPMVSSNISSMPSGADRLVSRANGGTKKELPSPKDGPVPGVHIAAQ
TFTFRELAAATKNFRPESFLGEGGFGRVYKGRLESTGQIVAVKQLDRNGLQGNREFLVEVLMLSLLHHPNLVNLI
GYCADGDQRLLVYDFMPLGSLEDHLHDLPPDKEPLDWNTRMKIAAGAAKGLEYLHDKANPPVIYRDFKSSNILL
DEGFHPMLSDFGLAKLGPVGDKSHVSTRVMGTYGYCAPEYAMTGQLTVKSDVYSFGVVFLELITGRKAIDSTRP
HGEQNLVTWARPLFNDRRKFSKLADPQLQGRYPMRGLYQALAVASMCIQEQAATRPLIGDVVTALSYLANQAY
DPNTGHGHRGSGDKDEKRNRDERVGRILKNEEGGGSGRRWDFEGSEKDDSPRETARMLNRDLDRERAVAEA
KMWGENWREKRRQSAQGSFDGNNG

>A463|A0A0B0P3A2

MGCFSCFDSKEEEKLNTVNETNDPKRPQPIVSSNISRLSSGGDRLRSRSNGGSKREIPSLRDGPGVQIAAQTFSFR
ELAAATKNFRPESFLGEGGFGRVYKGRLESTGQVVAVKQLDRNGLQGNREFLVEVLMLSLLHHPNLVNLIGYCA
DGDQRLLVYEFMPLGSLEDHLHDLPPGKEPLDWNTRMKIAAGAAKGLEYLHDKANPPVIYRDFKSSNILLEEGFH
PKLSDFGLAKLGPVGDKSHVSTRVMGTYGYCAPEYAMTGQLTVKSDVYSFGVVFLELITGRKAIDSSQPHGEQN
LIAWARPLFNNRRKLSKLADPLLQGRYPMRGLYQALAVASMCIQEEAATRPLIGDVVTALSYLANQAYDPNTTG
NGHRGSGETDEKRYRDDRGGRVSKNDEGGVSGRKGDLEGSEKEDSPRETARMLNRDLDRERAVAEAKMWGE
NWREKRRQSAQGSSDGSNG

>A464|A0A1U8M516

MGCFSCFDSKEEEKLNAVNETNDPKRPQPIVSSNISRLSSGGDRLRSRSNGGSKREIPSLRDGPGVQIAAQTFSFR
ELAAATKNFRPESFLGEGGFGRVYKGRLESTGQVVAVKQLDRNGLQGNREFLVEVLMLSLLHHPNLVNLIGYCA
DGDQRLLVYEFMPLGSLEDHLHDLPPSKEPLDWNTRMKIAAGAAKGLEYLHDKANPPVIYRDFKSSNILLEEGFH
PKLSDFGLAKLGPVGDKSHVSTRVMGTYGYCAPEYAMTGQLTVKSDVYSFGVVFLELITGRKAIDSSQPHGEQN
LITWARPLFNNRRKLSKLADPLLQGRFPMRGLYQALAVASMCIQEEAATRPLIGDVVTALSYLANQAYDPNTTG
NGHRGSGETDEKRYRDDRGGRVSKNDEGGVSGRKWDLEASEKEDSPRETARMLNRDLDRERAVAEAKMWG
ENWREKRRQSAQGSSDGSNG

>A465|A0A5D3ACQ9

MGCFSCFDSKEEEKLNTVNETNDPKRPQPIVSSNISRLSSGGDQLRSRSNGGSKREIPSLRDGPGVQIAAQTFSFR
ELAAATKNFRPESFLGEGGFGRVYKGRLESTGQVVAVKQLDRNGLQGNREFLVEVLMLSLLHHPNLVNLIGYCA
DGDQRLLVYEFMPLGSLEDHLHDLPPGKEPLDWNTRMKIAAGAAKGLEYLHDKANPPVIYRDFKSSNILLEEGFH
PKLSDFGLAKLGPVGDKSHVSTRVMGTYGYCAPEYAMTGQLTVKSDVYSFGVVFLELITGRKAIDSSQPHGEQN
LIAWARPLFNNRRKLSKLADPLLQGRYPMRGLYQALAVASMCIQEEAATRPLIGDVVTALSYLANQAYDPNTTG
NGHRGSGETDEKRYRDDRGGRVSKNDEGGVSGRKGDLEGSEKEDSPRETARMLNRDLDRERAVAEAKMWGE
NWREKRRQSAQGSSDGSNG

>A466|LOC107417665

MGCFPCFDSREEEKLNPEKQSDDRKQSQPAVPSGLSSMPSGADRFRSRSNANGGSKRELPVPKDVPTVNIAAQ
TFTFRELAVATKNFRPESFIGEGGFGRVYKGRLESTGQVVAVKQLDRNGLQGNREFLVEVLMLSLLHHPNLVNLI
GYCADGDQRLLVYEFMPMGSLEDHLHDLPPDKEPLDWNTRMKIAAGAAKGLEYLHDKANPPVIYRDFKSSNILL
DEGFHPKLSDFGLAKLGPTGDKSHVSTRVMGTYGYCAPEYAMTGQLTVKSDVYSFGVVFLELITGRKAIDSNRPH
GEQNLVTWARPLFNDRRKFSKLADPQLQGRYPMRGLYQALAVASMCIQEQAATRPLIGDVVTALSYLANQAYD

PKTSSGHGYRGSGDKDDKRNKDGRISKNEEGGGSGRRWDLEGSEKEDSPRETARMLNRDLDRERAVAEAKM
WGENWREKRRQSAQGSFDGANM

>A467|LOC111318441

MGCFPCFDSKEEEKLNPVKETDDRKQSRPTVSSNVSRLSSGGDRLRSRSNGESRRELPSPKDGPGVQIAAQTFTF
RELAAATKNFRPESFLGEGGFGHVYKGRLESTGQVVAVKQLDRNGLQGNREFLVEVLMLSLLHHPNLVNLIGYC
ADGDQRLLVYEFMPLGSLEDHLHDLPPEKEPLDWNTRMKIAAGAAKGLEYLHDKANPPVIYRDFKSSNILLEEGF
HPKLSDFGLAKLGPVGDKSHVSTRVMGTYGYCAPEYAMTGQLTVKSDVYSFGVVFLELITGRKAIDSTRPHGEQ
NLVTWASPLFNDRRKFSKLADPRLRGRYPMRGLYQALAVASMCIQEQAATRPLIGDVVTALSYLANQAYDPNTT
GHVYRGSGDKDDKRYKDDRGGRVSKNDEGGASGLRWDLEGSEKEDSPRETARMLNRDLDRERAVAEAKMW
GENWREKRRQSAQGSSDGSNG

>A468|A0A2C9UNZ8

MGCFPCFDSREEETLNPQKESDDRKRSLPAESSNISKLSSGADRLRSNGRSKRELALPKDGLPGGNIAAQIFTFREL
AAATKNFRPESFLGEGGFGRVYKGRLESTGQVVAVKQLDRNGLQGNREFLVEVLMLSLLHHPNLVNLIGYCADG
DQRLLVYEFMPLGSLEDHLHDLPPDKEPLDWNTRMRIAAGAAKGLEYLHDKANPPVIYRDFKSSNILLDEGFHPK
LSDFGLAKLGPTGDKSHVSTRVMGTYGYCAPEYAMTGQLTVKSDVYSFGVVFLELITGRKAIDSSQPHGEQNLVS
WARPLFNDRRKFSKLADPRLQGRYPMRGLYQALAVASMCIQEQAAARPLIGDVVTALSYLANQAYEPNSAGHG
HRGSADKDEKRNRDERGGQLSKSEEGGRSERRWDLDGSEKEDSPRETARMLNRDLDRERAVAEAKMWGEN
WREKRRQSAQGSFDGSNG

>A469|A0A5N5NAY9

MGCFSCFDSREEEKLNQEKQSVDLKQTLPTVSSNISKLSSGADRIRSRSNVGQFKRELPGLKDAPGVNIAAHIFTF
RELAAATKNFRPECFLGEGGFGRVYKGRLESTGQVVAVKQLDRNGLQGNREFLVEVLMLSLLHHPNLVNLIGYC
ADGDQRLLVYEFMPLGSLEDHLHDLPPEKEPLDWNTRMKIAAGAARGLEYLHDKASPPVIYRDFKSSNILLDEGF
HPKLSDFGLAKLGPTGDKSHVSTRVMGTYGYCAPEYAMTGQLTVKSDVYSFGVVFLELITGRKAIDSTRPHGEQ
NIVTWTRPLFNDRRKFSKLADPRLQGRYPMRGLYQALAVASMCIQEQAAARPLIGDVVTALSYLANQAYEPNG
HGHRGSGDRDEKRHRDERGGQLSRNEEGGGSGRKWELDGSEKEDSPRETARMLNRDLDRERAVAEAKMWG
ENWREKRRQSAQGSFDGSNG

>A470|B9GZC6

MGCFSCFDSREEEKLNQEKQSVDLKQTLPPVSSNISKLSSGADRFKSRSNGEQSKRELPSPKDAPGVNIAAHIFTF
RELAAATKNFRPECFLGEGGFGRVYKGRLESTGQVVAVKQLDRNGLQGNREFLVEVLMLSLLHHPNLVNLIGYC
ADGDQRLLVYEFMPLGSLEDHLHDLPPEKEPLDWNTRMKIAAGAARGLEYLHDKASPPVIYRDFKSSNILLEEGF
HPKLSDFGLAKLGPTGDKSHVSTRVMGTYGYCAPEYAMTGQLTVKSDVYSFGVVFLELITGRKAIDSSRPHGEQ
NLVTWTRPLFNDRRKFSKLADPRLQGRYPMRGLYQALAVASMCIQEQAAARPLIGDVVTALSYLANQAYEPNG
HGYRGLGDRDEKRHREERGGQLSRNEEGGGSGRKWDLDGSEKEDSPRETARMLNRDLDRERAVAEAKMWG
ENWREKRRQSAQGSFDGSNG

>A471|LOC105133734

MGCFPCFDSREEKELNRQKQSDALKQTLPIVPSNISKLSSGSDRLRPRSNGGQSKRQLPSPKDAPGVNIAAQIFAF
RELAAATKNFMPECFLGEGGFGRVYKGCLESTGQVVAVKRLDRNGLQGNREFLVEVLMLSLLHHPNLVNLIGYC
ADGDQRLLVYEFMPLGSLEDHLHDLPPEKEPLDWNTRMKIAAGAAKGLEYLHDKASPPVIYRDFKSSNILLEEGF
HPKLSDFGLAKLGPTGDKSHVSTRVMGTYGYCAPEYAMTGQLTVKSDVYSFGVVFLELITGRKAIDSAQPHGEQ

NLVAWARPLFSDRRKFSKLADPRLQGRYPMRGLYQALAVASMCIQEQAAARPLIGDVVTALSYLANQAYEPNG
HGHRGSGDRDEKRQRDERGGQLSRNEEGGGSGRRWDLDGSEKEDSPRETAKMVNRDLDRERAVAEAKMW
GENWREKQRQNAQGSFDGSNG

>A472|LOC111281877

MGCFPCFGSREEEKFNPVKETDDRKPGHATVSSNISRLSSGGDRLRSRSSGGSKRELPSPKDGPGVQIAAQIFTFR
ELAAATKNFRPESFLGEGGFGHVYKGQLESAGQVVAVKQLDRNGLQGNREFLVEVLMLSLLHHPNLVNLIGYCA
DGDQRLLVYEFMPLGSLEDHLHDLPPEKEPLDWNTRMKIAAGAAKGLEYLHDKANPPVIYRDFKSSNILLDEGFH
PKLSDFGLAKLGPVGDKSHVSTRVMGTYGYCAPEYAMTGQLTVKSDVYSFGVVFLELITGRKAIDSTRPHGEQNL
ITWARPLFNDRRKFSKLADPRLQGRYPMRGLYQALAVASMCIQEQAVTRPLIGDVVTALSYLASQAYDPNAAG
HGYRGSGEKDDRRHRDDRGRVPKNDEGGTSGRRWDLEGSEKDDSPRETARMLNRDLDRERAVAEAKMWGE
NWREKRRQSAQGSFDGSNG

>A473|100305406

MGCFSCFDSREDEMLNPNPQQENHHHEHEHDHDLKPPVPSRISRLPPSASAGDKLRSTTSNGESKRELAAAVQI
AAQIFTFRELAAATKNFMPQSFLGEGGFGRVYKGLLETTGQVVAVKQLDRDGLQGNREFLVEVLMLSLLHHPNL
VNLIGYCADGDQRLLVYEFMPLGSLEDHLHDLPPDKEPLDWNTRMKIAAGAAKGLEYLHDKANPPVIYRDFKSS
NILLDEGYHPKLSDFGLAKLGPVGDKSHVSTRVMGTYGYCAPEYAMTGQLTVKSDVYSFGVVFLELITGRKAIDST
RPHGEQNLVTWARPLFNDRRKFPKLADPQLQGRYPMRGLYQALAVASMCIQEQAAARPLIGDVVTALSFLAN
QAYDHRGGTGDDKRNRVLKNGEGGGGGSGGRWDLEGSEKDDSPRETARMLNSNNRDLDRERAVAEAKMW
GENWREKRRQSAQGSFDGSNA

>A474|A0A151U8B2

MGCFSCFDSREDEKLNPNPQQETNHHDHELKPPVPSRISRLPSASEKLRSTSNGDSNGELAALKDGPAVQIAAQ
TFTFRELAAATKNFRPQSFLGEGGFGRVYKGRLETTGQVVAVKQLDRNGLQGNREFLVEVLMLSLLHHPNLVNLI
GYCADGDQRLLVYEFMPMGSLEDHLHDLPPDKEPLDWNTRMKIAAGAAKGLEYLHDKANPPVIYRDFKSSNILL
DEGYHPKLSDFGLAKLGPVGDKSHVSTRVMGTYGYCAPEYAMTGQLTVKSDVYSFGVVFLELITGRKAIDSTRPH
GEQNLVTWARPLFNDRRKFPKLADPQLQGRYPMRGLYQALAVASMCIQEQAAARPLIGDVVTALSFLANQAF
DHRGAGEDKRNRDDKGGRILKNDEGGGSGRRWDLEGSEKDESPRETARMLNNMHLDRERAVAEAKMWGE
NWREKRRQSAQGSFDGSNA

>A475|A0A1S2Y0L1

MGCFSCFDSKEDEKLNPNPQQXXXXXHNLPSHLSRLPSASAAEKLRSTSNGASKRELPVALKDGPPGQIAAQTFT
FRELAAATKNFRPQSFLGEGGFGRVYKGRLETTGQAVAVKQLDRNGLQGNREFLVEVLMLSLLHHPNLVNLIGY
CADGDQRLLVYEFMPLGSLEDHLHDLPPDKEPLDWNTRMKIAAGAAKGLEYLHDKANPPVIYRDFKSSNILLDE
GYHPKLSDFGLAKLGPVGDKSHVSTRVMGTYGYCAPEYAMTGQLTVKSDVYSFGVVFLELITGRKAIDGTRPHG
EQNLVTWARPLFNDRRKFPKLADPRLQGRYPMRGLYQALAVASMCIQEQAAARPLIGDVVTALSYLANQAYDT
NNAGHGNNKGIGDDKGGKILKNDGGGGSGRRWDLEGSEKDDSPRETARMLNRDLDRERAVAEAKMWGENL
REKRRQSVQGSFDASNA

>A476|A0A1S2Y2H4

MGCFSCFDSKEDEKLNPNPQQXXXXXHNLPSHLSRLPSASAEKLRSTSNGASKRELPVALKDGPPGQIAAQTFTF
RELAAATKNFRPQSFLGEGGFGRVYKGRLETTGQAVAVKQLDRNGLQGNREFLVEVLMLSLLHHPNLVNLIGYC
ADGDQRLLVYEFMPLGSLEDHLHDLPPDKEPLDWNTRMKIAAGAAKGLEYLHDKANPPVIYRDFKSSNILLDEG

YHPKLSDFGLAKLGPVGDKSHVSTRVMGTYGYCAPEYAMTGQLTVKSDVYSFGVVFLELITGRKAIDGTRPHGE
QNLVTWARPLFNDRRKFPKLADPRLQGRYPMRGLYQALAVASMCIQEQAAARPLIGDVVTALSYLANQAYDT
NNAGHGNNKGIGDDKGGKILKNDGGGGSGRRWDLEGSEKDDSPRETARMLNRDLDRERAVAEAKMWGENL
REKRRQSVQGSFDASNA

>A477|XP_010419881.2

MGCFSCFDSSDDEKLNPVEESNGQKKKQLQPTVSNSISGLPSGGEKLSSKSNGGSKRELLLPRDGLNQIAAHTFA
FRELAAATMNFHPDTFLGEGGFGRVYKGRLDSTGQVVAVKQLDRNGLQGNREFLVEVLMLSLLHHPNLVNLIG
YCADGDQRLLVYEFMPLGSLEDHLHDLPPDKEALDWNMRMKIAAGAAKGLEFLHDKANPPVIYRDFKSSNILLD
EGFHPKLSDFGLAKLGPTGDKSHVSTRVMGTYGYCAPEYAMTGQLTVKSDVYSFGVVFLELITGRKAIDSEMPH
GEQNLVAWARPLFNDRRKFIKLADPKLKGRFPTRALYQALAVASMCIQEQAATRPLIADVVTALSYLANQAYDP
SKDESRRNRDERGARLITRNDEGGGSGSKFDLEGSEKEDSPRETVRILNRDINRERAVAEAKMWGESLREKRRQ
SEQGTSESNSTG

>A478|A0A0D3EFV1

MGCFSCFDSSDDEKLNPVDESKSQKQSQPTLSNNNISGLPSGGEKLGSKSNGGSRRELLLPRDGLSQISAHTFSF
HEVAAATMNFHPDTFLGEGGFGRVYKGRLDSTGQVVAVKQLDRNGLQGNREFLVEVLMLSLLHHPNLVNLIGY
CADGDQRLLVYEFMPLGSLEDHLHDLPPDKEALDWNMRMKIAAGAAKGLEFLHDKANPPVIYRDFKSSNILLDE
GFHPKLSDFGLAKLGPTGDKSHVSTRVMGTYGYCAPEYAMTGQLTVKSDVYSFGVVFLELITGRKAIDTDMPHG
EQNLVAWARPLFNDRRKFIKLADPRLKGRFPTRALYQALAVASMCIQEQAATRPLIADVVTALSYLANQAYDPN
KDDSKRNRDEKGGRLITRNDEGGGSGSKFDLEGSEKEDSPRETTRILNRDINRERAVAEAKMWGESLREKRRQS
EQGTSESNSTG

>A479|A0A2J6LE65

MGCFSCFDSRVEEKSNPQKVGADRPEVHPSAPSNISRLPSGVDRLKSRNNVSLRRESSGSKEGPYGQIAAQTFTF
RELAAATSNFSPDCFLGEGGFGHVYRGRLQGSGQVVAVKQLDRNGLQGNREFLVEVLMLSLLHHPNLVNLIGY
CADGDQRLLVYEFMPLGSLEDHLHDLPPDREALDWNTRMKIAAGAARGLEFLHDKANPPVIYRDFKSSNILLGE
GFQPKLSDFGLAKLGPTGDKSHVSTRVMGTYGYCAPEYAMTGQLTVKSDVYSFGVVFLELITGRKAIDSTAPQG
QQNLVTWARPLFNDRRKFASLADPRLEGHYPMRGLYQALAVASMCIQEQAAARPLIGDVVTALSYLANHAYDP
GAVNGQSNKHTNGRVLKNEEGGGSGRSRWDLEGSEKGESPRDTPRILNRDLDRERAVAEAKMWVEKRRQSA
QGSFDGNSNGSGQ

>A480|A0A0D3AIS7

MGCFSCFDSSDDETLNPAAEESSKAGQKQSQPTVSNSLSGLPSGGEKLNSNSKSNGGAKTELLLPRDGLGQIAA
HTFTFRELAAATMNFHPDTFLGEGGFGRVYKGRLDSTGQVVAVKQLDRNGLQGNREFLVEVLMLSLLHHPNLV
NLIGYCADGDQRLLVYEFMSLGSLEDHLHDLPPDKEALDWNMRMKIAAGAAKGLEFLHDKANPPVIYRDFKSS
NILLDEGFHPKLSDFGLAKLGPTGDKSHVSTRVMGTYGYCAPEYAMTGQLTVKSDVYSFGVVFLELITGRKAIDT
DMPHGEQNLVAWARPLFNDRRKFIKLADPKLKGRFPTRALYQALAVASMCIQGEAATRPLIADVVTALSYLANQ
GYDPNKSERGARLITRNDEGGGSGSKFDLEGSEKEDSPRETARMLNRDINRERAVAEAKMWGESLREKRRQSE
QGTSESNSTG

>A481|A0A151TDB0

MGCFSCFDSSSKEDHGVPQHQPNPSRISRLPSASGADKLRGGSSSRREVAPKDGHVQIAAQTFTFRELAAATKN
FRAESFVGEGGFGRVYKGRLETTAQIVAVKQLDKNGLQGNREFLVEVLMLSLLHHPNLVNLIGYCADGDQRLLV

YEYMPLGSLEDHLHDLPPDKEPLDWNTRMKIAVGAAKGLEYLHDKANPPVIYRDFKSSNILLDEGYNPKLSDFGL
AKLGPVGDKSHVSTRVMGTYGYCAPEYAMTGQLTVKSDVYSFGVVFLELITGRKAIDSTQPQGEQNLVTWARP
LFNDRRKFSKLADPRLQGRFPMRGLYQALAVASMCIQESAATRPLIGDVVTALSYLANQTYDPNNSGHGYRGSS
DDKRNRDDKGGRILKNDETGGSGRRWDLEGSEKDDSPRETARMLNRDLDRERAVAEAKMWGENLRQQRKQ
RGQQGSFDGSNA

>A482|A0A3N6PXS2

MGCFSCFDSSDDETLNPAAEESSKAGQKQSQPTVSNSLSGLPSGGEKLNSNSKSNGGAKTELLLPRDGLGQIAA
HTFTFRELAAATMNFHPDTFLGEGGFGRVYKGRLDSTGQVVAVKQLDRNGLQGNREFLVEVLMLSLLHHPNLV
NLIGYCADGDQRLLVYEFMSLGSLEDHLHDLPPDKEALDWNMRMKIAAGAAKGLEFLHDKANPPVIYRDFKSS
NILLDEGFHPKLSDFGLAKLGPTGDKSHVSTRVMGTYGYCAPEYAMTGQLTVKSDVYSFGVVFLELITGRKAIDT
DMPHGEQNLVAWARPLFNDRRKFIKLADPKLKGRFPTRALYQALAVASMCIQGEAATRPLIADVVTALSYLANQ
GYDPNKNERGARLITRNDEGGGSGSKFDLEGSEKEDSPRETARMLNRDINRERAVAEAKMWGESLREKRRQSE
QGTSESNSTG

>A483|A0A398A8N6

MGCFSCFDSSDDETLNPAAEESSKTQKQSQPTVSNTLSALPSGGEKLNSNSKSNGGAKTELLLPRDGLGQIAAHT
FTFRELAAATMNFHPDTFLGEGGFGRVYKGRLDSTGQVVAVKQLDRNGLQGNREFLVEVLMLSLLHHPNLVNLI
GYCADGDQRLLVYEFMSLGSLEDHLHDLPPDKEALDWNMRMKIAAGAAKGLEFLHDKANPPVIYRDFKSSNILL
DEGFHPKLSDFGLAKLGPTGDKSHVSTRVMGTYGYCAPEYAMTGQLTVKSDVYSFGVVFLELITGRKAIDTDMP
HGEQNLVAWARPLFNDRRKFIKLADPKLKGRFPTRALYQALAVASMCIQGEAATRPLIADVVTALSYLANQGYD
PNKNERGARLITRNDEGGGSGSKFDLEGSEKEDSPRETARMLNRDINRERAVAEAKMWGESLREKRRQSEQGT
SESNSTG

>A484|LOC104810211

MGCFSCFDSREEEKLNPQRESEDQKLSQPTVSNHFSSLSSGGEKLRARSNGARELPSPKDNGAGAQIAAQTFTFR
DLAAATMNFHPDSFLGEGGFGRVYKGRLESTGQVVAVKQLDRNGLQGNREFLVEVLMLSLLHHSNLVNLIGYC
ADGDQRLLVYEFMPFGSLEDHLHDIPPDKEPLDWNTRMKIAAGAAKGLEFLHDKANPPVIYRDFKSSNILLDEGF
HPKLSDFGLAKLGPTGDQSHVSTRVMGTYGYCAPEYAMTGQLTVKSDVYSFGVVFLELITGRKAIDSEKPHGEQ
NLVAWARPLFNDRRKFIKLADPRLKGRFPMRGLYQALAVASMCIQEQAATRPLIGDVVTALSYLANQGYDPNK
DERRSRDERGGKLLSRNDEGGSGRRLDLDGSEKDDSPRETARVLNRDIDRERAVAEAKMWGENLREKRRQSAQ
GTSESSNG

>A485|A0A314L0Y4

MGCFSCFDSKEEEKLNPQRDDRKEVHLTAPSNISRLSSGADRLKTRSINGSKRELLGLKDAPDVQIAAHTFTFREL
AAATSNFRPESFIGEGGFGRVYKGRLPSGQVVAVKQLDRNGLQGNREFLVEVLMLSLLHHPNLVNLIGYCADGD
QRLLVYEFMPLGSLEDHLHDLPPDKEPLDWNTRMKIAAGAAKGLEYLHDKANPPVIYRDFKSSNILLEENFFPKLS
DFGLAKLGPTGDKSHVSTRVMGTYGYCAPEYAMTGQLTVKSDVYSFGVVFLELITGRKAIDSTMPQGEQNLVA
WARPLFNDRRKFAKLADPRLQGQFPMRGLYQALAVASMCIQEQAAARPLIGDVVTALSYLANQVDDKRNKDD
RGGRICRNEDGAGGGSGRKWPDLDGGSEKEDSPRETARMLNRDLDRERAVAEAKMWGENWREKRRQNAQ
GSFDGTNG

>A486|A0A175YRL2

MGCFPCFDSKEEESLNPQKHSNHHKETHTHPALSNNISRLASGADRFKSRSNVGGKREQPGLKELADAQIAAQT
FTFRELAAATNNFRPESFIGEGGFGRVYKGRLQTTGQVVAVKQLDRDGLQGNREFLVEVLMLSLLHHSNLVNLIG
YCADGDQRLLVYEFMPLGSLEDHLHDLPPDKEPLDWNTRMKIAAGAAKGDFKSSNILLEEEFHPKLSDFGLAKLG
PTGDKSHVSTRVMGTYGYCAPEYAMTGQLTVKSDVYSFGVVFLELITGRKAIDSTQPQGQQNLVTWARPLFND
RRRFAKLADPRLQGQYPMRGLYQALAVASMCIQEQAAARPLIGDVVTALSYLANQAYDSSSVPGHGYRLNGDR
DEKNNRGSGRFLRNDMGGGSGLKWDLEGSEKDDSPKETARTLNRDLERERAVAEAKLWGENLREKRRQEDNF
DTNNE

>A487|A0A2G9HHZ2

MGCFPCFDSREEEKLNPNKDDHKEAHPNIPSNISKLSSGGEKLRSRSNVGLRKEGSGLKDLPDAQIAAQTFTFREL
ANATNNFRPECFIGEGGFGRVYKGRLPNGQVVAVKQLDRNGLQGNREFLVEVLMLSLLHHPNLVNLLGYCADG
DQRLLVYEFMPLGSLEDHLHDLPPDKEPLDWNTRMKIAAGAAKGLEYLHDKANPPVIYRDFKSSNILLGEGYAPK
LSDFGLAKLGPTGDKSHVSTRVMGTYGYCAPEYAMTGQLTVKSDVYSFGVVFLELITGRKAIDSTRPQGEQNLV
AWARPLFNDRRKFAKLADPRLQGKFPMRGLYQALAVASMCIQEQAAARPLIGDVVTALSYLANQAYDPSHCNR
TMGDKDDNRSKDERGGKILRNDEGGGSGRKWDMEMSERDDSPRETAKMLNRDLDRERAVAEAKMWGEN
WRKGRQNS

>A488|LOC108836105

MGCFSCFDSSDDETLNPAEESKAQKQSQPTVSNSLSGLPSGGEKLSSKSNGVSKTELLLPRDGLAQIAAHTFAFRE
LAAATMNFHPDTFLGEGGFGRVYKGRLDSTGQVVAVKQLDRNGLQGNREFLVEVLMLSLLHHPNLVNLIGYCA
DGDQRLLVYEFMSLGSLEDHLHDLPPDKEALDWNMRMKIAAGAAKGLEFLHDKANPPVIYRDFKSSNILLDEGF
HPKLSDFGLAKLGPTGDKSHVSTRVMGTYGYCAPEYAMTGQLTVKSDVYSFGVVFLELITGRKAIDTDMPHGEQ
NLVAWARPLFNDRRKFIKLADPRLKGRFPTRALYQALAVASMCIQEQAATRPLIADVVTALSYLANQGYDPNKN
ERGARLITRNDEGCVSGSKFDLEGSEKEDSPRETARMLNRDINRERAVAEAKMWGESLREKRRQSEQGTSESNS
TG

>A489|LOC103856003

MGCFSCFNSSEDEKLNPVEESSKPQKQSQTIVSNNFSTLPSGGEKLSSKSNVRSKRELLLPRDGFEQIAAHTFAFH
ELVAATMDFHPDTFLGEGGFGCVYKGRLETTGQVVAVKQLDRNGLQGNREFLVEVLMLSLLHHPNLVNLIGYC
ADGDQRLLVYEFMPLGSLEDHLHDLPPDKEALDWNMRMKIAAGAAKGLEFLHDKANPPVIYRDFKSSNILLGE
GFHPKLSDFGLAKLGPTGDKSHVSTRVMGTYGYCAPEYAMTGQLTVKSDVYSFGVVFLELITGRKAIDSEMPHG
EQNLVAWARPMFNDRRRFIKLADPKLKGRFPTRALYQALSVASMCIQEEAATRPPIADVVTAISYLANQAYDPN
KNDRGGRNDEGGGKFDLEGSEKEDSPRETARILNRDTDRKRAVAEAKMWGESSREKRRQSEKRTSESNSTTG

>A490|A0A251RUB6

MGCFPCFESSQEDNNNFNHQKVGHEVHPSAPSNISRLSSGVDRMKTRNNVNNAASLRRESSGPPDAQIAAQT
FTFRELAAATNNFRPDCFLGEGGFGCVYRGRLQSSGQVVAVKQLDRNGLQGNREFLVEVLMLSLLHHPNLVNLI
GYCADGDQRLLVYEFMALGSLEDHLHDVPPDREPLDWNTRMKIAAGAAKGLEFLHDKANPPVIYRDFKSSNILL
DEGFQPKLSDFGLAKLGPTGDKSHVSTRVMGTYGYCAPEYAMTGQLTVKSDVYSFGVVFLELITGRKAIDSTQP
HGQQNLVTWARPLFNDRRKFTSLVDPRLEGRYPMRGLYQALAVASMCIQEQAAARPLIADVVTALSYLANQGY
DPTTAPAHNIISSSSKERPKTARSSKNDEGGCSRWDLEGSDSPKETVKMLERERAVAEAKMWVEKRRQSG

>A491|A0A287ICY4

MAGAEKARAKGNAGMKELSDLRDANGNVLSAQTFTFRQLTAATRNFREECFIGEGGFGRVYKGRLDGGQVVA
IKQLNRDGNQGNKEFLVEVLMLSLLHHQNLVNLVGYCADGEQRLLVYEYMPLGSLEDHLHDLPPDKEPLDWNT
RMKIAAGAAKGLEYLHDKAQPPVIYRDFKSSNILLGDDFHPKLSDFGLAKLGPVGDKSHVSTRVMGTYGYCAPEY
AMTGQLTVKSDVYSFGVVLLELITGRKAIDSTRPHGEQNLVSWARPLFNDRRKLPKMADPGLQGRYPMRGLYQ
ALAVASMCIQSEAASRPLIADVVTALSYLASQIYDPNAIHASKKAGGDQRSRVSDSGRALLKNDEAGSSGHKSDR
DDSPREPPPGILNDRERMVAEAKMWGANLREKTRAAANAQGSLDSPTETG

>A492|A0A1U8J682

MGWFPCGGKPNKKGKKKLPNHNNTTTINSSDDQIPSTSEKLKVNSVPDAKKEANKDGGSDHIAAHTFTFRELA
AATKNFRADYLLGEGGFGRVYKGRLESTNQVVAIKQLDRNGLQGNREFLVEVLMLSLLHHPNLVNLIGYCADGD
QRLLVYEYMPLGSLEDHLHDLPSDKRQLDWNTRMKIAAGAAKGLEYLHDKASPPVIYRDLKCSNILLGEGYHPKL
SDFGLAKLGPVGDKTHVSTRVMGTYGYCAPEYAMTGQLTLKSDVYSFGVVLLEIITGRKAIDNSRAGGEQNLVA
WARPLFKDRRKFAQMADPLLQGQYPVRGLYQALAVAAMCVQEQPNMRPLIADVVTALTYLASQKYDPGTQT
VQGSRTGSSTPRMRRE

>A493|A0A2P5WD55

MGWFPCGGKPNKKGKKKLPNHNNTTTINSSDDQIPSTSEKLKVSSVPDAKKEANKDGGSDHIAAHTFTFRELAA
ATKNFRADYLLGEGGFGRVYKGRLESTNQVVAIKQLDRNGLQGNREFLVEVLMLSLLHHPNLVNLIGYCADGDQ
RLLVYEYMPLGSLEDHLHDLPPDKRQLDWNTRMKIAAGAAKGLEYLHDKASPPVIYRDLKCSNILLGEGYHPKLS
DFGLAKLGPVGDKTHVSTRVMGTYGYCAPEYAMTGQLTLKSDVYSFGVVLLEIITGRKAIDNSRAGGEQNLVAW
ARPLFKDRRKFAQMADPLLQGQYPVRGLYQALAVAAMCVQEQPNMRPLIADVVTALTYLASQKYDPGTQTVQ
GSRTGSSTPRMRRE

>A494|A0A5D2TAX2

MGWFLCGGNSNQDEKKKLPINNNTTTNNNFDHQIPSTSEKLKVNSAPNTKKEATKDGGSGHIAAHTFTFRELA
AATKNFRADCLLGEGGFGRVYKGRLESTNQVVAIKQLDPNGLQGNREFLVEVLMLSLLHHPNLVNLIGYCADGD
QRLLVYEYMPLGSLEDHLHDLPPDRRRLDWNTRMKIAAGAAKGLEYLHDKASPPVIYRDLKCSNILLGEGYHPKL
SDFGLAKLGPVGDKTHVSTRVMGTYGYCAPEYAMTGQLTLKSDVYSFGVVFLEIITGRKAIDNSRAGGEQNLVA
WARPLFKDRRKFAQMADPLLQGQYPARGLYQALAVAAMCVQEQPNMRPLIADVVTALTYLASQRYDPETQS
VQGPRTGSSTPRMRRE

>A495|A0A2H5NZ35

MGWFPCAGKSSKNAKKKHHNKPIDQIPSTSEKLKVNSTVDVKKEASKDGGSDHIAAHTFTFRELAAVTKNFRAD
CLLGEGGFGRVYKGRLESTNQVVAIKQLDRNGLQGNREFLVEVLMLSLLHHPNLVNLIGYCADGDQRLLVYEYM
PLGSLEDHLHDLPPDKKRLDWTTRMRIAAGAAKGLEYLHDKANPPVIYRDLKCSNILLGEGYHPKLSDFGLAKLG
PVGDKTHVSTRVMGTYGYCAPEYAMTGQLTLKSDVYSFGVVLLEIITGRKAIDNTRAAGEHNLVAWARPLFKDR
RKFSQMADPTLQGQYPVRGLYQALAVAAMCVQEQPNMRPLIADVVTALTYLACQKYDPESQPVQSSRTGSST
PRIRREQ

>A496|A0A2I0L8D1

MLSLLHHPNLVNLIGYCADGDQRLLVYEFMPLGSLEYHLFDLPPDKEPLDWNTRMKIAAGAAKGLEYLHDKANP
PVIYRDFKSSNILLDEGYFPKLSDFGLAKLGPVGDKSHVSTRVMGTYGYCAPEYAMTGQLTVKSDVYSFGVVFLEL
ITGRRAIDSSRPHGEQNLVTWARPLFNDRKKFSKLADPRLQGRYPMRGLYQALAVASMCIQEQAAARPLIGDVV
TALSYLANRLYDSTAPSGHRGGLGSNDGRGSGRILRNEELGGSGRRWDFEGSEKEDSPRETGKILNSNRDAERER
VVAEAKMWGENWREKRRQSAQGSLDRNITNI

>A497|AHZ63816.1

RVAQTSADARLHAAFEGSGSGGGGAGGDGGAGDGGSGVSDYSKLGQPSSSGRTEVAPEAITAYLQRMQRGGL
TQAFGCMLVVAGNTILALSENAPEMLEMGVGLGTDLRMLFTSGSTAALEQAAKEEDLNSVNPVVMQSCGGSG
KRFYAVLHRIEDVAGLVMDLEPIEGGVEKKSSAAMAVKPIARIKSLPGGDMGRLCQAVVEEVQEMTGYDRVMA
YKFHEDEHGEVVAEMRRPDLEPYLGLHYPSTDVPQASRMMFMKNGVRMIGDCTLPPVRVVQAKELAQPISLA
GSTLRAPHGCHAQYMCNMGSAASLTMAVVIDDFDDDSSLSGSRKLWGLVVCHHTSPRKVSYPLRCACQKLME
AFGVHLNMELEFAAQLREHHILTTQTLLCDMLRRIRGAPIGIVYRSPSIMDLVKCDGAALYYGGKLWPLGITPSEF
QVQDLAEWLLGSSEEIASSGVTCTDSLAEAGYPGATAFGDAVCGMAAARITPNSDFLFWFRSHTAKEVFWGGA
EHDPQARDDDSRLLPRSSFKAFLEIVKRRSLPWEEVEVDAIRSLQLILREDLEEFCAAAAAGADGMDVDSLVHLSK
KLSLKESEEMGGAENSKDAAAGGTGGGGWEKMSLPSSLAQEWMEAIRGTGDGGASGGVPFDWDAITVFQQ
TSFVVVDALKPDLPIIFASTGFFNLTGYTSREVIGGNCRFLQGPDTNPEDVDSIREALVPQGTGTFCGRLLNYRKD
GSNFWNLLTIAPIKDDTGTIVKLIGVQLEVSKYTEGSRANRLRPNGLPQSLIKYDVRHQDKVSALVAQIVAALTKPY
KVEPPRPSYAMRASLTGQTIEPLSPGQAAAARPYSTSDVRQTAAIPREGGGRRRHRSSTFLSLLGMEEKDSEEDQ
FLEPELIMVDDALVGRPGSLDDRERTRRGIDLATTLERIGHSFVITDPRLPDNPIIFASDQFLELTEYSREEVLGENC
RFLQGRDTDLKAVQLIRDAVNEGRDVTVQLLNYTRGGRPFWNLFHLQAMRDKKGNLQYFIGVQQETDTLDRV
EQEEAEVVRATAQNVDVAARELPDANLTPDHLWERHSKVVTPLPHSKINSPCWYAIRKVQRRLRRGERLGLKHF
RPIKPLGSGDTGSVHLVELRGTGQVFALKAMDKSMMLHRNKVHRARAEREILGMMDHPFLPTLYASFQTKTHV
CLVMDFCPRGDLFLLQDKQPDKTLSEEAARFYAAEVVVALEYLHCMGVIYRDLKPENLLLQKNGHILLTDFDLSFL
TSCHPQLITSGRGGRRRMKKRRARVVFCAEPHVSSNSFVGTEEYIAPEIISGHPHSSAVDWWALGILLYEMLYGR
TPFCGRNRHKTFINVLNDELTFPSSIPVSVAGRQLIAGLLQRDPARRLGAFRGASDVKKHPFF

>A498|AHZ63819.1

YLQRMQRGGLTQAFGCMLAVAGNSILALSENAPEMLEMAVGLSTDLRMLFTSASTAALEQAAKEEDLSXVNPV
VVQSCGGSGKRFYAVLHRIQDVGGLVMDLEPIEGGPGVXVXKXSSAEMAVKXIARIQXLPXGDIGRLCQAVVEE
VQEMTGYDRVMAYKFHEDEHGEVVAEMRRPEQEPYLGLHYPSTDVPQASRMMFMKNGVRMIGDCTLPPVR
VVQAKELAQPISLAGSTLRAPHGCHAQYMCNMGSAASLTMAVVIDDYDDDSSLSXGSRKLWGLVVCHHTSPR
KVSYPLRCACQKLMEAFGVQLNMELEFAAQLREHHILTTQTLLCDMLRRIRGAPIGIVSRSPSIMDLVKCDGAAL
YYGGKLWPLGTTPSEFQVQDLAEWILGSSEEIASSGVTCTDRLAEAGYPGAAALGDAVCGMAAARITPNSDFLF
WFRSHTAKEVFWGGAEHDPQARDDDSRLLPRSSFKSFLEIVKRRSLPWEEVEVDAIRSLQLILRENLEEFCAAAA
AGADGEDVDSLVPLSKKLSLKESGEMDGAENSKKLERATSTAAGAAGGTGGGWEKMSLPSNLAQEWMEAIRG
TGDGGASGGVGGVPFDWDAITVFQQTSFVVVDALKPDLPIIFASTGFFNLTGYTSREVIGGNCRFLQGPDTNPE
DVASIREALVPQGTGTFCGRLLNYRKDGSNFWNLLTIAPIKDDSGTIVKLIGVQLEVSKYTEGSRANRLRPNGLPQ
SLIKYDVRHQDKVSALVAQLVAALTKPDKVZPPRPSYAMRXSLTGQTIEXLSPGXAAAARXYSXSDVPQTAAIPRE
GGGRRRHRSSTFLSLLGMEEKDSEEDQFPEPELIMVDDASVGRPGTSDDRERTRRGIDLATTLERIGHSFVITDPR
LPDNPIIFASDQFLELTEYSREEVLGENCRFLQGRDTDRKAVQLIRDAVKEGRDVTVQLLNYTRGDRPFWNLFHL
QAMRDKKGNLQYFIGVQQETDMPDRVEQEKAKVMRATAQNVDLAARELPDANVTPDHLWERHSKAVTPLP
HSKINSPCWYAIRRVQRRLRRGERLGLKHFRPIKPLGSGDTGSVHLVELRGTGQVFALKAMDKSMMLQRNKVH
RARAEREILGMMDHPFLPTLYASFQTKTHVCLIMDFCPXGDLFLLQDKQPNKTLSEEXARFYAAEVVVVLEYLHC

MGVIYRDLKPENVLLQKNGHILLTDFDLSFLTSCRPQLITSGRGGRRRIKKRRVRVIFCAEPNVSSNSFVGTEEYIAP
EIISGHPHSSAVDWWALGILLYEMLYGRTPFCGSNRHKTFINVLNEELTFPTSIPVSLAGRQLIAGLLQRDPARRLG
ALRGASDVKKHPFFQGIEWPLIRWRSPPNNLHNLAPSFDEF

>A499|A0A4U6U1V7

MGCFPCFGSGREDDELGYYGGNGGAAGWAAAAASSSAAAAAAAAVGGGAEAAVAAPPRAERSPTGSNKSKA
KGNGSKKELAVLKDANGNVISAQTFTFRQLAAATKNFRDECFIGEGGFGRVYKGRLDGTGQVVAIKQLNRDGN
QGNKEFLVEVLMLSLLHHQNLVNLVGYCADGDQRLLVYEYMPLGSLEDHLHDLPPGKEALDWNTRMKIAAGA
AKGLEYLHDKADPPVIYRDFKSSNILLGEGFHAKLSDFGLAKLGPVGDKSHVSTRVMGTYGYCAPEYAMTGQLTV
KSDVYSFGVVLLELITGRKAIDSTRPAAEQNLVSWARPLFNDRRKLPKMADPGLEGRFPTRGLYQALAVASMCIQ
SEAASRPLIADVVTALSYLANQIYDPNTASTSKKGGGDQRSRVSDSGRALSRNDDTGSSGHRSPSKDLDDSPRER
HATGTAHKGERERMVAEAKMWGENWREKQRAAQGSLDSPTGAAPLKCRRTNLYSFYACSMPWWYCGRLR
NTVFSKECPRSHIPIRLSEAGAGEVYWVWWSFAISCAYRDQQELGCLATKKSRCEFTEYCD

>A500|A0A3N6SWR7

MSWCCLPCFGSSAKDADSKDSLKKEVSAKDGSVTQQSHRASLDKSKSRGGSEHKELTAPKEGPTANIAAQTFTF
RELAAATKNFRPECLLGEGGFGRVYKGRLESTGQIVAVKQLDRNGLQGNREFLVEVLMLSLLHHTNLVNLIGYCA
DGDQRLLVYEYMALGSLEDHLHDLPPGKEPLDWNTRMTIAAGAAKGLEYLHDKANPPVIYRDLKSSNILLGDGY
HPKLSDFGLAKLGPVGDKTHVSTRVMGTYGYCAPEYAMTGQLTLKSDVYSFGVVFLELITGRKAIDNARAPGEH
NLVAWARPLFKDRRKFPKMADPSLQGRYPMRGLYQALAVAAMCLQEQAATRPLIGDVVTALTYLASQTFDPN
APSGQNSRSGGGGGPPFIRTRDERRSLGDGSSLDSPAETRSRLGSPGTHKNSPDYRRRDMVREVNAGSEAGSE
AGGGSGRKWGLSDVEGTESQRGSPASVGRGGRGTPRNRDLDRERAVAEAKVLSFMARGFVVIWNICWVWS
GQLSDLMSESEGRLFNVSWLKLKNKKNGEGES

>A501|A0A0E0K0A0

MGCFSCFDSPAEEQLNPKVGGPYGGGSSSAAAAVYGGGGGSGAGRHGERGGGYPDLHHHHHHHQQQQPM
AAPRVEKLSAGAEKARVKSNAILREPSAPKDANGNVISAQTFTFRELATATRNFRPECFLGEGGFGRVYKGHLEST
GQVVAIKRLNRDGLQGNREFLVEVLMLSLLHHQNLVNLIGYCADGDQRLLVYEYMHFGSLEDHLHELPPDKEAL
DWNTRMKIAAGAAKGLEYLHDKANPPVIYRDFKSSNILLDESFHPKLSDFGLAKLGPVGDKSHVSTRVMGTYGY
CAPEYAMTGQLTVKSDVYSFGVVLLELITGRRAIDSTRPHGEQNLVSWARPLFNDRRKLPKMADPRLEGRYPMR
GLYQALAVASMCIQSEAASRPLIADVVTALSYLASQSYDPNAAHASRKPGSDQRSKVGENGRVVSRNDEASSSG
HKSPNKDREDSPKEPPGILNKDFDRERMVAEAKMWGDRERMVAEAKMWGDRERMVAEAKMWGENWRD
KRRAIENGQGSLDSPTENG

>A502|A0A0D9YSS0

MGCFSCFDSPAEEQLNPKVGGPYGGGSSSSAAAAAYGGGGGSGAGRHGERGGGYPDLHHHHHQQQLPMAA
PRVEKLSAGAEKTRVKSNAILREPSAPKDANGNVISAQTFTFRELATATRNFRPECFLGEGGFGRVYKGRLESTGQ
VVAIKQLNRDGLQGNREFLVEVLMLSLLHHQNLVNLIGYCADGDQRLLVYEYMHFGSLEDHLHDLPPDKEALD
WNTRMKIAAGAAKGLEYLHDKANPPVIYRDFKSSNILLDESFHPKLSDFGLAKLGPVGDKSHVSTRVMGTYGYC
APEYAMTGQLTVKSDVYSFGVVLLELITGRRAIDSTRPHGEQNLVSWARPLFNDRRKLPKMADPRLEGRYPMR
GLYQALAVASMCIQSEAASRPLIADVVTALSYLASQSYDPNAAHASRKPGGDQRSKVGENGRVISRNDEASSSG
HKSPNKDREDSPKEPPGILNKDFDRERMVAEAKMWGDRERMVAEAKMWGDRERMVAEAKMWGENWRD
KRRAIENGQGSLDSPTENG

>A503|A0A445BCI4

MGGCFPCFGSSNKEESGGGGLKKEVAKKESFKESSAPQVHHPSRVSSDKSKSRCSSDPKKETPVPKDGPTAHIAA
QTFTFRELAAATKNFRPECLLGEGGFGRVYKGRLENTGQVVAVKQLDRNGLQGNREFLVEVLMLSLLHHPNLVN
LIGYCADGDQRLLVYEFMPLGSLEDHLHGFSKKLLTLSLFPDLPPDKEPLDWNTRMKIAAGAAKGLEYLHDKANP
PVIYRDLKSSNILLDEGYHPKLSDFGLAKLGPVGDKTHVSTRVMGTYGYCAPEYAMTGQLTLKSDVYSFGVVFLEL
ITGRKAIDNTRAHGEHNLVAWARPLFKDRRKFPKMADPLLQGRYPMRGLYQALAVAAMCLQEQAATRPLIGD
VVTALTYLASQTYEPNVANQSNRLGPSTPRSRDDRRSMIDGVDSPDRGRLGSPSTHRNSPDYRRRDNRELSVGT
ELGRSVSGSGGSGRKWGLDDLERQDSRRDSPVNTARARETPRNRDLDRERAVAEAKVWGENWREKKRANA
MGSFDATNE

>A504|A0A445C2F8

MGGCFPCFGSSNKEESGGGGLKKEVAKKESFKESSAPQVHHPSRVSSDKSKSRCSSDPKKETPLPKDGPTAHIAA
QTFTFRELAAATKNFRPECLLGEGGFGRVYKGRLENTGQVVAVKQLDRNGLQGNREFLVEVLMLSLLHHPNLVN
LIGYCADGDQRLLVYEFMPLGSLEDHLHGFSKKFLTPSLFPDLPPDKEPLDWNTRMKIAAGAAKGLEYLHDKANP
PVIYRDLKSSNILLDEGYHPKLSDFGLAKLGPVGDKTHVSTRVMGTYGYCAPEYAMTGQLTLKSDVYSFGVVFLEL
ITGRKAIDNTRAHGEHNLVAWARPLFKDRRKFPKMADPLLQGRYPMRGLYQALAVAAMCLQEQAATRPLIGD
VVTALTYLASQTYEPNVANQSNRLGPSTPRSRDDRRSMIDGVDSPDRGRLGSPSTHRNSPDYRRRDNRELSVGT
ELGRSVSGSGGSGRKWGLDDLERQDSRRDSPVNTARARETPRNRDLDRERAVAEAKVWGENWREKKRANA
MGSFDATNE

>A505|A0A0E0CL51

MGCFSCFDSPAEEQLNPKVGGPYGGGSSSAAAAAYGGGGGSGAGRHGERGGGYPDLHHHHHQQLPMAAPR
VEKLSAGAEKARVKSNAILREPSAPKDANGNVISAQTFTFRELATATRNFRPECFLGEGGFGRVYKGRLESTGQV
VAIKQLNRDGLQGNREFLVEVLMLSLLHHQNLVNLIGYCADGDQRLLVYEYMHFGSLEDHLHDLPPDKEALDW
NTRMKIAAGAAKGLEYLHDKANPPVIYRDFKSSNILLDESFHPKLSDFGLAKLGPVGDKSHVSTRVMGTYGYCAP
EYAMTGQLTVKSDVYSFGVVLLELITGRRAIDSTRPHGEQNLVSWARPLFNDRRKLPKMADPRLEGRYPMRGLY
QALAVASMCIQSEAASRPLIADVVTALSYLASQSYDPNAAHASRKPGGDQRSKVGENGRVVSRNDEASSSGHKS
PNKDREDSPKEPPGILNKDFDRERMVAEAKMWGDRERMVAEAKMWGDRERMVAEAKMWGENWRDKRR
AIENGQGSLDSPTENG

>A506|B8AT94

MICVGLCAGARGRGNATVKKELSALKDANGNVISAQTFTFRQLAAATRNFREECFIGEGGFGRVYKGRLDGTGQ
IVAIKQLNRDGTQGNKEFLVEVLMLSLLHHQNLVNLVGYCADGDQRLLVYEYMPLGSLEDHLHDLPPDKVPLD
WNTRMKIAAGAAKGLEYLHDKAQPPVIYRDFKSSNILLGEDFHPKLSDFGLAKLGPVGDKSHVSTRVMGTYGYC
APEYAMTGQLTVKSDVYSFGVVLLELITGRKAIDSTRPHVEQNLVSWARPLFNDRRKLPKMSDPGLEGRYPMRG
LYQALAVASMCIQSEAASRPLIADVVTALSYLASQKYDPNTTPSSKKAGGGEAGRALSRNDEAGSSGHKSPSSKD
SPREQQLPGILNDRERMVAEAKMWGENWREKRRAAATTSSNAQHIIGVARAKGVSGDDGEADGGECEAARR
ASVLGEDTAGGKQVEESLDDGGVQVARVGASDQSLVGTPRRMMAVSAMGTEIMFASSIITRRASMGRIYRLG
RLLRPFSKVSP

>A507|LOC108817636

MSGCLPCFGSSAKDAASKDSVKKEASSAKAKDGSVTQSHHVSLDKSKSRGGAEHKKELTAPKEGPTAHIAAQTF
TFRELAAATKNFRPDCLLGEGGFGRVYKGRLETTGQIVAVKQLDRNGLQGNREFLVEVLMLSLLHHTNLVNLIGY

CADGDQRLLVYEYMPLGSLEDHLHDLPPDKEPLDWNTRMTIAAGAAKGLEYLHDKANPPVIYRDLKSSNILLGD
GYHPKLSDFGLAKLGPVGDKTHVSTRVMGTYGYCAPEYAMTGQLTLKSDVYSFGVVFLELITGRKAIDNARAHG
EHNLVAWARPLFKDRRKFPKMADPSLQGRYPMRGLYQALAVAAMCLQEQAATRPLIGDVVTALTYLASQTFD
PNAASSQNSRSGGGGGPPPFIRTRDERRSMGGDGSSLDSPAETRSRLGSPATHNKNSPDYRRRDMVREVVNA
AGSEAGSENGGGSGRKWGLSDVEGTESQRGSPASVGRGTRGTPRNRDLDRERAVAEAKVWGENWRERKRGI
NGPGSFDSSND

>A508|LOC108833676

MSGCLPCFGSSAKDASTKDSVKKEASSAKAKDGSVTQSHHVSLDKSKSRGGSEHKKELTAPKEGPTAHIAAQTFT
FRELAAATKNFRPDCLLGEGGFGRVYKGRLETTGQIVAVKQLDRNGLQGNREFLVEVLMLSLLHHTNLVNLIGYC
ADGDQRLLVYEYMPLGSLEDHLHDLPPDKEPLDWNTRMTIAAGAAKGLEYLHDKANPPVIYRDLKSSNILLGDG
YHPKLSDFGLAKLGPVGDKTHVSTRVMGTYGYCAPEYAMTGQLTLKSDVYSFGVVFLELITGRKAIDNARAHGE
HNLVAWARPLFKDRRKFPKMADPSLQGRYPMRGLYQALAVAAMCLQEQAATRPLIGDVVTALTYLASQTFDP
NAASSQNSRSGGGGGPPPFIRTRDERRSMGGDGSSLDSPAETRSRLGSPATHNKNSPDYRRRDMVREVVNAA
GSEAGSENGGGSGRKWGLSDVEGTESQRGSPASVGRGTRGTPRNRDLDRERAVAEAKVWGENWRERKRGIN
GPGSFDSSND

>A509|LOC108847302

MSGCCLPCFGSSTAKDAAESKDSLKKEVSVKDASVTHHSHHVSLDKSKSRGGSEHKKELTAPKEGPTAHIAAQTF
TFRELAAATKNFRPECLLGEGGFGRVYKGRLESTGQIVAVKQLDRNGLQGNREFLVEVLMLSLLHHTNLVNLIGY
CADGDQRLLVYEYMALGSLEDHLHDLPPDKEPLDWNTRMTIAAGAAKGLEYLHDKANPPVIYRDLKSSNILLGD
GYHPKLSDFGLAKLGPVGDKTHVSTRVMGTYGYCAPEYAMTGQLTLKSDVYSFGVVFLELITGRKAIDNARAPG
EHNLVAWARPLFKDRRKFPKMADPSLQGRYPMRGLYQALAVAAMCLQEQAATRPLIGDVVTALTYLASQTFD
PNAPSGQNSRSSGGGGGPPFIRTRDERRSLGDGSSLDSPAETRSRLGSPAAHKNSPDYRRRDMAREVVNAGSE
AGSEAGGGSGRKWGLSDVEGTESQRGSPASVGRGTRGTPRNRDLDRERAVAEAKVWGENWRERKRGTNGP
GSFDSSND

>A510|A0A2K1KK32

MGWFSCFTSAPKERKPQKNDDNNSSRDGQSAATTPVAQISNLPSGNSKKYDAKGSFDKNEPQREGSTHIAAQT
FTFRELAAATKNFRPECLLGEGGFGRVYKGRLENTGQVVAVKQLDRNGLQGNREFLVEVLMLSLLHHPNLVSLIG
YCADGDQRLLVYEFMPLGCLEDHLHDLPQDKECLDWNTRMKIAAGAARGLEYLHDTAKPPVIYRDFKSSNILLD
EGFHPKLSDFGLAKLGPVGDKTHVSTRVMGTYGYCAPEYAMTGQLTLKSDVYSFGVVLLELITGRKAIDNSRSAG
EHNLVAWARPLFKDRRKFPSMADPMLQGRYPMRGLYQALAVAAMCLQEQAATRPLIGDVVTALSYLASQIYD
PGMHPLGSSRFAPATPSREKREKEKKMPPGPNTIEERMVKEQRAGKGGPRSPSVRSQAASPDLWVKEGGGRA
RVTNGVLEESRGSESGSGRKRDSEEWERHEGGGRDSPGYAGRASRDGLKSQAPTRERERAVAEARVWGENW
RERKRGAG

>A511|A0A2P5WKR2

MGGCFPCFGSSNKAGSNGGGSVKELSKKDSTKDSSVGQPHHVNGVNSDKSKSRSVSDPKKEPAVPKDGPTANI
AAQTFTFRELAAATKNFRPECLLGEGGFGRVYKGRLESTGQVVAVKQLDRNGLQGNREFLVEVLMLSLLHHPNL
VNLIGYCADGDQRLLVYEFMPLGSLEDHLHGKGADLPSDKEPLDWNTRMRIAAGAAKGLEYLHDKANPPVIYR
DLKSSNILLDEGFHPKLSDFGLAKLGPVGDKTHVSTRVMGTYGYCAPEYAMTGQLTLKSDVYSFGVVFIELITGRK
AIDNTRAPGEQNLVAWARPLFRDRRKFPKMADPLLRGRYPIRGLYQALAVAAMCLQEQAATRPLIGDVVTALT
YLASQTYDPNAPNNQSNRVGPSTPRVKDDRRSMADGLDSPDARGRVGSPSTHRNSPDYRKKNHVREMSSGA

ELSRNEPGEGSGRKWGSLDESEQQESHTDSPMNSARARETSRNRDLDRERAVAEAKVWGENWREKKRANA
MGDSDGRND

>A512|A0A5B6WLN4

MGGCFPCFGSSNKEGSNGGGSVKELSKKDSTKDSSVGQPHHVNRVNSDKSKSRSVSDPKKEPAVSKDGPTANI
AAQTFTFRELAAATKNFRPECLLGEGGFGRVYKGRLESTGQVVAVKQLDRNGLQGNREFLVEVLMLSLLHHPNL
VNLIGYCADGDQRLLVYEFMPLGSLEDHLHGKGADLPSDKEPLDWNTRMRIAAGAAKGLEYLHDKANPPVIYR
DLKSSNILLDEGFHPKLSDFGLAKLGPVGDKTHVSTRVMGTYGYCAPEYAMTGQLTLKSDVYSFGVVFLELITGRK
AIDNTRAPGEQNLVAWARPLFRDRRKFPKMADPLLQGRYPIRGLYQALAVAAMCLQEQAATRPLIGDVVTALT
YLASQTYDPNAPSNQSNRVGPSTPRVKDDRRSMADGLDSPDARGRVGSPSTHRNSPDYRKKNHVREMSSGAE
LSRNEPGEGSGRKWGSLDESEQQESHTDSPMNSARARETSRNRDLDRERAVAEAKVWGENWREKKRANAM
GDSDGRND

>A513|A0A0D3EEB5

MSGCLPCFGSSAKDAASKDSVKKEASAKAKDASVTQSHHVSLDKSKSKGGSEQKKELTAPKEGPTAHIAAQTFTF
RELAAATKNFRPDCLLGEGGFGRVYKGRLETTGQIVAVKQLDRNGLQGNREFLVEVLMLSLLHHTNLVNLIGYCA
DGDQRLLVYEYMPLGSLEDHLHDLPPDKESLDWNTRMTIAAGAAKGLEYLHDKANPPVIYRDLKSSNILLGDGY
HPKLSDFGLAKLGPVGDKTHVSTRVMGTYGYCAPEYAMTGQLTLKSDVYSFGVVFLELITGRKAIDNARAHGEH
NLVAWARPLFKDRRKFPKMADPSLQGRYPMRGLYQALAVAAMCLQEQAATRPLIGDVVTALTYLASQTFDPN
AASSQNSRSGSGGGGPPFIRTRDERRSMGDGSSLDSPAETRSRLGSPATHKNSPDYRRRDMVREVNAGSEAGS
ENGGGSGRKWGLSDVEGTESQRGSPASVGRGTRGTPRNRDLDRERAVAEAKVWGENWRERKRGINGPGSFD
SSND

>A514|A0A0D9VGC3

MGCFSCFDSPAEEQLNPKVGGGSSSSHAAAAAYGGGGGGSGAGRHGDGRGYPDLHHHHQPMAAPRVEKLS
AGPEKARVKSNVIAREPSAPKDANGNVISAQTFTFRELATATRNFRPECFLGEGGFGRVYKGRLESTGQVVAIKQ
LNRDGLQGNREFLVEVLMLSLLHHQNLVNLIGYCADGDQRLLVYEYMHFGSLEDHLHDLPSDKEALDWNTRM
KIAAGAAKGLEYLHDKANPPVIYRDFKSSNILLDESFHPKLSDFGLAKLGPVGDKSHVSTRVMGTYGYCAPEYAM
TGQLTVKSDVYSFGVVLLELITGRRAIDSTRPHGEQNLVSWARPLFNDRRKLPKMADPRLEGRYPMRGLYQALA
VASMCIQSEAASRPLIADVVTALSYLASQSYDPNAAHASRKPGGDQRKVGENGRVVSRNDEASSSGHKSPNKD
REDSPKELPGILNKDFDRERMVAEAKMWGDRERMVAEAKMWGDRERMVAEAKMWGENWRDKRRAVEN
GQGSLDSPTENG

>A515|A0A3N6PNA0

MSGCLPCFGSSAKDAASKDSVKKEASAKAKDASVTQSHHVSLDKSKSKGGSEQKKELTAPKEGPTAHIAAQTFTF
RELAAATKNFRPDCLLGEGGFGRVYKGRLETTGQIVAVKQLDRNGLQGNREFLVEVLMLSLLHHTNLVNLIGYCA
DGDQRLLVYEYMPLGSLEDHLHDLPPDKEPLDWNTRMTIAAGAAKGLEYLHDKANPPVIYRDLKSSNILLGDGY
HPKLSDFGLAKLGPVGDKTHVSTRVMGTYGYCAPEYAMTGQLTLKSDVYSFGVVFLELITGRKAIDNARAHGEH
NLVAWARPLFKDRRKFPKMADPSLQGRYPMRGLYQALAVAAMCLQEQAATRPLIGDVVTALTYLASQTFDPN
AASSQNSRSGSGGGGPPFIRTRDERRSMGDGSSLDSPAETRSRLGSPATHKNSPDYRRRDMVREVNAGSEAGS
ENGGGSGRKWGLSDVEGTESQRGSPASVGRGTRGTPRNRDLDRERAVAEAKVWGENWRERKRGINGPGSFD
SSND

>A516|A0A498HM77

MGGCFPCFGSSKNKEGSGGGGGVKEVTKKDSSVKEGSAAQSHHVTRVSSDKKSRNGSDPKKEPPIPKDGPTAHI
AAQTFTFRELAAATKNFRPECLLGEGGFGRVYKGRLESTGQVVAVKQLDRNGLQGNREFLVEVLMLSLLHHTNL
VNLIGYCADGDQRLLVYEFMPLGSLEDHLHDLPSDKEPLDWNTRMKIAAGAAKGLEYLHDRANPPVIYRDLKSS
NILLDEGFHPKLSDFGLAKLGPVGDKTHVSTRVMGTYGYCAPEYAMTGQLTLKSDVYSFGVVFLELITGRKAIDN
TRGPGEHNLVAWARPLFKDRRKFPKMADPLLQGRYPMRGLYQALAVAAMCLQEQAATRPLIGDVVTALTYLA
SQTYDPNAASTQSNRVGPSTPRHRDERRNMGDGLDSPDDSLRGGRQGSPARHKNSPDFRRRDPNKDLNTAA
ELGRVETGTGSGRRGGLDGLDRQESQRDSPLSAGRARETPRNRDLDRERAVAEAKVWGENWREKKRANAMG
SFDGTNE

>A517|A0A540MVB6

MGGCFPCFGSSKNKEGSGGGGGVKEVTKKDSSVKEGSAAQSHRVTRVSSDKKSRNGSDPKKEPPIPKDGSTAHI
AAQTFTFRELAAATKNFRPECLLGEGGFGHVYKGRLESTGQVVAVKQLDRNGLQGNREFLVEVLMLSLLHHPNL
VNLIGYCADGDQRLLVYEFMPLGSLEDHLHDLPSDKEPLDWNTRMKIAAGAAKGLEYLHDKANPPVIYRDLKSS
NILLDEGFHPKLSDFGLAKLGPVGDKTHVSTRVMGTYGYCAPEYAMTGQLTLKSDVYSFGVVFLELITGRKAIDN
TRGPGEHNLVAWARPLFKDRRKFPKMADPLLQGRYPMRGLYQALAVAAMCLQEQAATRPLIGDVVTALTYLA
SQTYDPNAATTPSNRGGSSTPRHRDERRNMGDGLDSPDEYVRGGRHGSPATHKNSPDFRRRDPNRDLNTGVE
LGRIETGTGSGRRWGLDGLERQESQRDSPQSAGRARETPRNRDLDRERAVAEAKVWGENWREKKRANAMGS
FDGTNE

>A518|A0A5N5HB58

MGGCFPCFGSSKNKEGSGGGSGVKEVTKKDSSVKEGSAAQSHHVTRVSSDKKLRNGSDPKKEPPIPKDGPTAHI
AAQTFTFRELAAATKNFRPECLLGEGGFGRVYKGRLESTGQVVAVKQLDRNGLQGNREFLVEVLMLSLLHHTNL
VNLIGYCADGDQRLLVYEFMPLGSLEDHLHDLPSDKEPLDWNTRMKIAAGAAKGLEYLHDKANPPVIYRDLKSS
NILLDEGFHPKLSDFGLAKLGPVGDKTHVSTRVMGTYGYCAPEYAMTGQLTLKSDVYSFGVVFLELITGRKAIDN
TRGPGEHNLVAWARPLFKDRRKFPKMADPLLQGRYPMRGLYQALAVAAMCLQEQAATRPLIGDVVTALTYLA
SQTYDPNAASTPSNRVGPSTPRHRDERRNMGDGLDSPDDSERGGRQGSPARHKNSPDFRRRDPNKDLNTAAE
LGRFETGTGSGRRGGLDGLDRQESQRDSPLSAGRARETPRNRDLDRERAVAEAKVWGENWREKKRANAMGS
FDGTNE

>A519|LOC103403780

MGGCFPCFGSSKNKEGSGGGGGVKEVTKKDSSVKEGSAAQSHHVTRVSSDKKSRNGSDPKKEPPIPKDGPTAHI
AAQTFTFRELAAATKNFRPECLLGEGGFGRVYKGRLESTGQVVAVKQLDRNGLQGNREFLVEVLMLSLLHHTNL
VNLIGYCADGDQRLLVYEFMPLGSLEDHLHDLPSDKEPLDWNTRMKIAAGAAKGLEYLHDKANPPVIYRDLKSS
NILLDEGFHPKLSDFGLAKLGPVGDKTHVSTRVMGTYGYCAPEYAMTGQLTLKSDVYSFGVVFLELITGRKAIDN
TRGPGEHNLVAWARPLFKDRRKFPKMADPLLQGRYPMRGLYQALAVAAMCLQEQAATRPLIGDVVTALTYLA
SQTYDPNAASTQSNRVGPSTPRHRDERRNXGDGLDSPDDSLRGGRQGSPARHKNSPDFRRRDPNKDLNTAAE
LGRVETGTGSGRRGGLDGLDRQESQRDSPLSAGRARETPRNRDLDRERAVAEAKVWGENWREKKRANAMGS
FDGTNE

>A520|LOC103932033

MGGCFPCFGSSKNKEGSGGGSGVKEVTKKDSSVKEGSAAQSHHVTRVSSDKKLRNGSDPKKEPPIPKDGPTAHI
AAQTFTFRELAAATKNFRPECLLGEGGFGRVYKGRLESTGQVVAVKQLDRNGLQGNREFLVEVLMLSLLHHTNL

VNLIGYCADGDQRLLVYEFMPLGSLEDHLHDLPSDKEPLDWNTRMKIAAGAAKGLEYLHDKANPPVIYRDLKSS
NILLDEGFHPKLSDFGLAKLGPVGDKTHVSTRVMGTYGYCAPEYAMTGQLTLKSDVYSFGVVFLELITGRKAIDNS
RGPGEHNLVAWARPLFKDRRKFPKMADPLLQGRYPMRGLYQALAVAAMCLQEQAATRPLIGDVVTALTYLAS
QTYDPNAASTQSNRVGPSTPRHRDERRNMGDGLDSPDDSERGGRQGSPARHKNSPDFRRRDPNKDLNTAAE
LGRVETGTGSGRRGGLDGLDRQESQRDSPLSAGRARETPRNRDLDRERAVAEAKVWGENWREKKRANAMGS
FDGTNE

>A521|LOC103958564

MGGCFPCFGSSKNKEGSGGGSGVKEVTKKDSSVKEGSAAQSHHVTRVSSDKKLRNGSDPKKEPPIPKDGPTAHI
AAQTFTFRELAAATKNFRPECLLGEGGFGRVYKGRLESTGQVVAVKQLDRNGLQGNREFLVEVLMLSLLHHTNL
VNLIGYCADGDQRLLVYEFMPLGSLEDHLHDLPSDKEPLDWNTRMKIAAGAAKGLEYLHDKANPPVIYRDLKSS
NILLDEGFHPKLSDFGLAKLGPVGDKTHVSTRVMGTYGYCAPEYAMTGQLTLKSDVYSFGVVFLELITGRKAIDN
TRGPGEHNLVAWARPLFKDRRKFPKMADPLLQGRYPMRGLYQALAVAAMCLQEQAATRPLIGDVVTALTYLA
SQTYDPNAASTQSNRVGPSTPRHRDERRNMGDGLDSPDDSERGGRQGSPARHKNSPDFRRRDPNKDLNTAA
ELGRVETGTGSGRRGGLDGLDRQESQRDSPLSAGRARETPRNRDLDRERAVAEAKVWGENWREKKRANAMG
SFDGTNE

>A522|LOC111292669

MGGCFPCFGSSNKEGSNGGDTVKELSKKDSTKDGSVGQSHHVNRVNSDKSKSRSASDSKKEPAVPKDGPTANI
AAQTFTFRELAAATQNFRPECLLGEGGFGRVYKGRLESTGQVVAVKQLDRNGLQGTREFLVEVLMLSLLHHPNL
VNLIGYCAEGDQRLLVYEFMPLGSLEDHLHDLPPDKEPLDWNTRMKIAAGAAKGLEYLHDRANPPVIYRDLKSS
NILLDEVYHPKLSDFGLAKLGPVGDKTHVSTRVMGTYGYCAPEYAMTGQLTLKSDVYSFGVVFLELITGRKAIDNT
CAPGEHNLVAWARPLFKDRRKFPKMADPLLQGRYPMRGLYQALAVAAMCLQEQAATRPLIGDVVTALTYLAS
QTYEPNVPSNHSNRVGPSTPRLKDDRKSIADGLDSPDDRGQHGSPSTRRNSPDYRKRNHGRELSFGAELGRNE
AGGGSGRKYGLDDPERQESHRDSPLNPVRARETPRNRDLDRAHAVAEAKVWGENWREKKRAMGSFDGTND
WRIQTT

>A523|101297427

MGGCFPCFGSSNKEGSSGGGGVKEVSKKELGKEGSATQSHHVTRVSSDRSKSRTGSDPKKEPPIPKDGPTAHIA
AQTFTFRELAAATKNFRPECLLGEGGFGRVYKGHLESTGQVVAVKQLDRNGLQGNREFLVEVLMLSLLHHPNLV
NLIGYCADGDQRLLVYEFMPLGSLEDHLHDLPTEKEPLDWNTRMKIAAGAAKGLEYLHDKANPPVIYRDLKSSNI
LLDEGFHPKLSDFGLAKLGPVGDKTHVSTRVMGTYGYCAPEYAMTGQLTLKSDVYSFGVVFLELITGRKAIDNTR
GPGEHNLVAWARPLFKDRRKFPKMADPLLQGHYPMRGLYQALAVAAMCLQEQAATRPLIGDVVTALTYLASQ
TYDPNAASGQSNRVGPSTPRHKDDRRNMGDGLDSPDEPGRGGRHGSHSYRNSPDFRRKDPNRDLGAGVELG
RIETGSGSGRKWGLDGLERQESQRDSPVSAGRARETPRNRDLDRERAVAEAKVWGENWREKKRANVMGGSF
DGTNE

>A524|111382165

MGCFPCFGSAKKEQNNVNVVKEVEKQESFKEVSVAQSRNHVSRTSSDKSKSRSGNDPKKEAATPKEPLAHIAAQ
TFTFRELASATQNFRPECLLGEGGFGHVYKGRLESTGQVVAVKQLDRNGLQGNREFLVEVLMLSLLHHPNLVNLI
GYCADGDQRLLVYEYMPLGSLEDHLHDLPSEKKPLDWNTRMKIAAGAAKGLEYLHDKANPPVIYRDLKSSNILLD
EGYFPKLSDFGLAKLGPVGDKTHVSTRVMGTYGYCAPEYAMTGQLTLKSDVYSFGVVFLEIITGRKAIDNRRDPG
EQNLVAWARPLFRDRRKFPKMADPLLQGCYPMRGLYQALAVAAMCLQEQAATRPLIGDVVTALTYLASQTYD
PNDHSPPSNRMDASTPRHRDARRNMSVGTDSLDESGRSSRHSSPSVHKNSPDFRKRESFKELNNGELRRIETGG
GSGRKLGLDELERPDSQRDSPVIAGRGRETPRNRDLDRERAVAEAKVWGENWRGRKRANGGVSYDGTNDSLH

>A525|A0A1U8MBZ0

MGGGGCFPCFGSSNKEKSNNGGKSIKELNNKDSTKDGSVGQSHHVNRASLDKSKSRSGSDSKKEPAVSKNGST
ENIPAQTFTFRELAAATKNFRPECLLGEGGFGHVYKGRLESTGQVVAVKQLDRNGLQGNREFLVEVLMLSLLHH
PNLVNLIGYCADGDQRLLVHEFMPLGSLEDHLHDLPPDKEPLDWNTRMKIAAGAAKGLEYLHDKASPPVIYRDL
KSSNILLDEGYHPKLSDFGLAKLGPVGDKTHVSTRVMGTYGYCAPEYAMTGQLTLKSDVYSFGVVFLELITGRKAI
DNMRAPGEQNLVAWARPLFKDRRKFPKMADPLLQGRYPMRGLYQALAVAAMCLQEQAATRPLIGDVVTALT
YLASQTYDPNAPGNQSNRVGPSNPRLKDDRNMADGLDSPEGRGWHHGSPSTHRNSPDYRKRNQMRESSIGS
ELIRNDAGGGSGRKCGLDDSERQESNRGSPLNASRATETPHNRVLDRERAVAEAKVWGENWRERKRANAMG
SFDGTNE

>A526|A0A2P5WI51

MGGGGCFPCFGSSNKEKSNNGGKSIKELNNKDSIKDGSVGQSHHVNRASLDKSKSRSGSDSKKEPAVSKNGSTE
NIPAQTFTFRELAAATKNFRPECLLGEGGFGRVYKGRLESTGQVVAVKQLDRNGLQGNREFLVEVLMLSLLHHP
NLVNLIGYCADGDQRLLVYEFMPLGSLEDHLHDLPPDKEPLDWNTRMKIAAGAAKGLEYLHDEASPPVIYRDLK
SSNILLDEGYHPKLSDFGLAKLGPIGDKTHVSTRVRGTYGYCAPEYAMTGQLTLKSDVYSFGVVFLELITGRKAIDN
MRAPGEQNLVAWARPLFKDRRKFPKMADPLLQGRYPMRGLYQALAVAAMCLQEQAATRPLMRDVVTALTYL
ASQTYDPNAPGNQSNRVGPSNPRLKDDRNMADGLDSPEGRGWHHGSPSTHRNSPDYRKRNQVRESSTGSELI
RNDAGGGSGRKCGSDDSERQESNRGSPLNASRARETPHNRVLDRERAVAEAKVWGENWRERKRANAMGSF
DGTNE

>A527|A0A397ZT17

MSWCCLPCFGSSAKDADSKDSVKKEVSAKDGSVTQQSHRASLDKSKSRGGSEHKKELTAPKEGPTANIAAQTFT
FRELAAATKNFRPECLLGEGGFGRVYKGRLETTGQIVAVKQLDRNGLQGNREFLVEVLMLSLLHHTNLVNLIGYC
ADGDQRLLVYEYMALGSLEDHLHDLPPGKEPLDWNTRMTIAAGAAKGLEYLHDKANPPVIYRDLKSSNILLGDG
YHPKLSDFGLAKLGPVGDKTHVSTRVMGTYGYCAPEYAMTGQLTLKSDVYSFGVVFLELITGRKAIDNARAPGE
HNLVAWARPLFKDRRKFPKMADPSLQGRYPMRGLYQALAVAAMCLQEQAATRPLIGDVVTALTYLASQTFDP
YAPNGQNSRSSGGGGPPFIRTRDERRSLGDGSSLDSPAETRSRLGSPGTHKNSPDYRRRDMVREVNAGSEAGSE
AGGGSGRKWGLSDVEGTESQRESPASVGRGGRGTPRNRDLDRERAVAEAKVWGENWRERKRGANEPGSFDS
FND

>A528|A0A5B6VFD3

MGGGGCFPCFGSSNKEKSNNGGKSIKELNNKDSTKDGSVGQSHHVNRVSLDKSKSRSGSDSKKEPAVSKNGST
ENIPAQTFTFRELAAATKNFRPECLLGEGGFGRVYKGRLESTGQVVAVKQLDRNGLQGNREFLVEVLMLSLLHH
PNLVNLIGYCADGDQRLLVYEFLPLGSLEDHLHDLPPDKEPLDWNTRMKIAAGAAKGLEYLHDKANPPVIYRDLK
SSNILLDEGYHPKLSDFGLAKLGPVGDKTHVSTRVMGTYGYCAPEYAMTGQLTLKSDVYSFGVVFLELITGRKAID
NMRAPGEQNLVAWARPLFKDRRKFPKMADPLLQGRYPMRGLYQALAVAAMCLQEQAATRPLIGDVVTALTY

LASQTYDPNAPGNQSNRVGPSNPRLKDDRNMADGLDSPECRGWHHGSPSTHRNSPDYRKRNQVRESSTGSE
LVRNDAGGGSGRKCGSDDSERQESNRGSPLNASRARETPHNRVLDRERAVAEAKVWGENWRERKRANAMG
SFDGSNE

>A529|A0A5D2W2N2

MGGGGCFPCFGSSNKEKSNNGGKSIKELNNKDSTKDGSVGQSHHVNRASLDKSKSRSGSDSKKEPAVSKNGST
ENIPAQTFTFRELAAATKNFRPECLLGEGGFGHVYKGRLESTGQVVAVKQLDRNGLQGNREFLVEVLMLSLLHH
PNLVNLIGYCADGDQRLLVYEFMPLGSLEDHLHDLPPDKEPLDWNTRMKIAAGAAKGLEYLHDKASPPVIYRDL
KSSNILLDEGYHPKLSDFGLAKLGPVGDKTHVSTRVMGTYGYCAPEYAMTGQLTLKSDVYSFGVVFLELITGRKAI
DNMRAPGEQNLVAWARPLFKDRRKFPKMADPLLQGRYPMRGLYQALAVAAMCLQEQAATRPLIGDVVTALT
YLASQTYDPNAPGNQSNRVGPSNPRLKDDRNMADGLDSPEGRGWHHGSPSMHRNSPDYRKRNQMRESSTG
SELIRNDAGGGSGRKCGLDDSERQESNRGSPLNASRARETPHNRVLDRERAVAEAKVWGENWRERKRANAM
GSFDGTNE

>A530|LOC103856323

MSWCCLPCFGSSAKDADSKDSLKKEVSAKDGSVTQQSHRASLDKSKSRGGSEHKKELTAPKEGPTANIAAQTFT
FRELAAATKNFRPECLLGEGGFGRVYKGRLETTGQIVAVKQLDRNGLQGNREFLVEVLMLSLLHHTNLVNLIGYC
ADGDQRLLVYEYMALGSLEDHLHDLPPGKEPLDWNTRMTIAAGAAKGLEYLHDKANPPVIYRDLKSSNILLGDG
YHPKLSDFGLAKLGPVGDKTHVSTRVMGTYGYCAPEYAMTGQLTLKSDVYSFGVVFLELITGRKAIDNARAPGE
HNLVAWARPLFKDRRKFPKMADPSLQGRYPMRGLYQALAVAAMCLQEQAATRPLIGDVVTALTYLASQTFDP
NAPNGQNSRSSGGGGPPFIRTRDERRSLGDGSSLDSPAETRSRLGSPGAHKNSPDYRRRDMVREVNAGSEAGS
EAGGGSGRKWGLSDVEGTESQRESPASVGRGTRGTPRNRDLDRERAVAEAKVWGENWRERKRGANEPGSFD
SFND

>A531|A0A397XLD2

MSGCLPCFGSSAKDAASKDSVKKEASAKAKDASVTQSHHVSLDKSKSKGGSEQKKELTAPKEGPTAHIAAQTFTF
RELAAATKNFRPDCLLGEGGFGRVYKGRLETTGQIVAVKQLDRNGLQGNREFLVEVLMLSLLHHTNLVNLIGYCA
DGDQRLLVYEYMPLGSLEDHLHDLPPDKEPLDWNTRMTIAAGAAKGLEYLHDKANPPVIYRDLKSSNILLGDGY
HPKLSDFGLAKLGPVGDKTHVSTRVMGTYGYCAPEYAMTGQLTLKSDVYSFGVVFLELITGRKAIDNARAHGEH
NLVAWARPLFKDRRKFPKMADPSLQGRYPMRGLYQALAVAAMCLQEQAATRPLIGDVVTALTYLASQTFDPN
VASSQNSRSGGGGPPFIRTRDERRSMGDGSSLDSPAETRSRLGSPATHKNSPDYRRRDMVREVNAGSEAGSEN
GGGSGRKWGLSDVEGTESQRGSPASVGRGTRGTPRNRDLDRERAVAEAKVWGENWRERKRGINGPGSFDSS
ND

>A532|A0A4D9A0D2

MGGCVPCFGSSNKDGNGNEFDNGVKEMGKKESFKDGSAAHSNSQVNRVNSDKSKARNSHDSKKEPGIPKEPT
AHIAAQTFTFRDLAAATKNFRPECLLGEGGFGRVYKGKLESGQVVAVKQLDRNGLQGNREFLVEVLMLSLLHHQ
NLVNLIGYCADGDQRLLVYEYMPLGSLEDHLHDLPPDKQPLDWNTRMKIAAGAAKGLEYLHDKANPPVIYRDLK
SSNILLDEDYFPKLSDFGLAKLGPVGDKTHVSTRVMGTYGYCAPEYAMTGQLTLKSDVYSFGVVFLELITGRKAID
NNRGAGEHNLVAWARPLFKDRRKFPKMADPDLQGQYPIRGLYQALAVAAMCLQEQAATRPFIGDVVTALTYL
ASQTYDPNSPSAQSNKTGPSTPRHRHERSASNGTDGLDDAGRGGHHRSSSTHKNSPDLRKRESPREFSVGGELR
RIETSGGSGSKWGSDEPDRPDSRRDSPASAGRVRESSRNRDLERERAVALAKVWGENWRERKRTSAMGSFDG
NE

>A533|LOC103845820

MSGCLPCFGSSAKDAASKDSVKKEASAKAKDASLTQSHHVSLDKSKSKGGSEQKKELTAPKEGPTAHIAAQTFTF
RELAAATKNFRPDCLLGEGGFGRVYKGRLETTGQIVAVKQLDRNGLQGNREFLVEVLMLSLLHHTNLVNLIGYCA
DGDQRLLVYEYMPLGSLEDHLHDLPPDKEPLDWNTRMTIAAGAAKGLEYLHDKANPPVIYRDLKSSNILLGDGY
HPKLSDFGLAKLGPVGDKTHVSTRVMGTYGYCAPEYAMTGQLTLKSDVYSFGVVFLELITGRKAIDNARAHGEH
NLVAWARPLFKDRRKFPKMADPSLQGRYPMRGLYQALAVAAMCLQEQAATRPLIGDVVTALTYLASQTFDPN
AASSQNSRSGGGGPPFIRTRDERRSMGDGSSLDSPAETRSRLGSPATHKNSPDYRRRDMVREVNAGSEAGSEN
GGGSGRKWGLSDVEGTESQRGSPASVGRGTRGTPRNRDLDRERAVAEAKVWGENWRERKRGINGPGSFDSS
ND

>A534|LOC105117132

MGGCFPCFGSSNKEGSSGGGVVKEVNKKDSAKEGSVGQSQHVGRVNSDKSKSRSGSDQKKEPSIPKDGPTANI
AAQTFTFRELATATKNFKPECLLGEGGFGRVYKGRLESTGQAVAVKQLDRNGLQGNREFLVEVLMLSLLHHPNL
VNLIGYCADGDQRLLVYEFMPLGSLEDHLHDLPPDKEPLDWNTRMKIAAGAAKGLEYLHDTANPPVIYRDLKSS
NILLDEGFHPKLSDFGLAKLGPVGDKTHVSTRVMGTYGYCAPEYAMTGQLTLKSDVYSFGVVFLELITGRKAIDN
TRAPGEHNLVAWARPLFKDRRKFPKMADPLLQGRYPMRGLYQALAVAAMCLQEQAATRPLIGDVVTALTYLA
SQTYDPNAASQSNRVGPSTPRNRDERRGMADGLDSPDEHGLGGRRDSPSICKNSPDYRKRDRVREFSTDAELG
RSEAGSGSGRKWGLDDSERQDSQKDSLVNTSRARETPRNRDLDRERAVAAAKVWGENWREKKRANAMGSF
DGTNE

>A535|LOC110664245

MGGCFPCFGSSNKEGSGGGAAVKEVAKKDSVKEGSVAQSHHVSRVSSDKSKSQNGSDPKKEPAIPKDGPTANI
AAQTFTFKELAAVTKNFRPECLLGEGGFGRVYKGYLESTGQVVAVKQLDRNGLQGNREFLVEVLMLSLLHHTNL
VNLIGYCADGDQRLLVYEFMPLGSLEDHLHDLPPDKEPLDWNTRMKIAAGAAKGLEYLHDKASPPVIYRDLKSS
NILLDEGYHPKLSDFGLAKLGPVGDKTHVSTRVMGTYGYCAPEYAMTGQLTLKSDVYSFGVVFLELITGRKAIDN
TRAPGENNLVAWARPLFKDRRKFPKMADPLLQGRYPMRGLYQALAVAAMCLQEQAATRPLIGDVVTALTYLA
SQTYDPNAANQSNRIGPSTPRNRDDPRGMAGGLHSPDEHGHDGQHGSPSTHKNSPDYRRRDTVWELSNGAE
LRRSDASGGSSRKWGLDDSEQQDSQRDNPVNNCRARETSRNRDLDRERAVADAKVWGQNWREKKRANAM
GSFNGTNE

>A536|A0A1U8IM87

MGGCFPCFGSSNKAGSNGGGSVKELSKKDSTKDSSVGQPHHVNRVNSDKSKSRSVSDPKKEPAVPKDGPTANI
AAQTFTFRELAAATKNFRPECLLGEGGFGRVYKGRLESTGQVVAVKQLDRNGLQGNREFLVEVLMLSLLHHPNL
VNLIGYCADGDQRLLVYEFMPLGSLEDHLHDLPSDKEPLDWNTRMRIAAGAAKGLEYLHDKANPPVIYRDLKSS
NILLDEGFHPKLSDFGLAKLGPVGDKTHVSTRVMGTYGYCAPEYAMTGQLTLKSDVYSFGVVFIELITGRKAIDNT
RAPGEQNLVAWARPLFRDRRKFPKMADPLLQGRYPIRGLYQALAVAAMCLQEQAATRPLIGDVVTALTYLASQ
TYDPNAPNNQSNRVGPSTPRVKDDRRSMADGLDSPDARGRVGSPSTHRNSPDYRKKNHVREMSSGAELSRNE
PGEGSGRKWGSLDESEQQESHTDSPMNSARARETSRNRDLDRERAVAEAKVWGENWREKKRANAMGDSDG
RND

>A537|A0A1U8K5L2

MGGCFPCFGSSNKAGSNGGGSVKELSKKDSTKDSSVGQPHHVNRVNSDKSKSRSVSDPKKEPAVPKDGPTANI
AAQTFTFRELAAATKNFRPECLLGEGGFGRVYKGRLESTGQVVAVKQLDRNGLQGNREFLVEVLMLSLLHHPNL
VNLIGYCADGDQRLLVYEFMPLGSLEDHLHDLPSDKEPLDWNTRMRIAAGAAKGLEYLHDKANPPVIYRDLKSS
NILLDEGFHPKLSDFGLAKLGPVGDKTHVSTRVMGTYGYCAPEYAMTGQLTLKSDVYSFGVVFLELITGRKAIDN
TRAPGEQNLVAWARPLFRDRRKFPKMADPLLQGRYPIRGLYQALAVAAMCLQEQAATRPLIGDVVTALTYLAS
QTYDPNAPNNQSNRVGPSTPRVKDDRRSMADGLDSPDARGRVGSPSTHRNSPDYRKKNHVREMSSGAELSR
NEPGEGSGRKWGSLDESEQQEFHTDSPMNSARARETSRNRDLDRERAVAEAKVWGENWREKKRANAMGDS
NGRND

>A538|A0A3N7FK27

MGGCFPCFGSSNKEGSSGVGLVKELNNKDSLKEGSAGQSHHVGRVSSDKSKSRSGSDPKKEQSIPKDGPTANIA
AQIFTFRELAAATKNFRPECLLGEGGFGRVYKGRIESTGQVVAVKQLDRNGLQGNREFLVEVLMLSLLHHPNLVN
LIGYCADGDQRLLVYEFMPLGSLEDHLHDLPPGKEPLDWNTRMKIAAGAAKGLEYLHDKANPPVIYRDLKSSNIL
LDEGYHPKLSDFGLAKLGPVGDKTHVSTRVMGTYGYCAPEYAMTGQLTLKSDVYSFGVVFLELITGRKAIDNTRD
PGEHNLVAWARPLFKDRRKFPKMADPLLQGRYPMRGLYQALAVAAMCLQEQAATRPLIGDVVTALTYLASQT
YDPNAVNQSNRVGPSTPRNRDDRRGMADGLDSPDEHGRGGRNGSPSTYKNSPDYRKRDHVREFSTGAELGRS
ETGGSGRKWGLDDSDQQDSQRDSPVSTSRARETPRNRDLDRERAVAEAKVWGENWREKRRANAMGSFDGT
NE

>A539|A0A5D2TBK2

MGGCFPCFGSSNKAGSNGGGSVKELSKKDSTKDSSVGQPHHVNRVNSDKSKSRSVSDPKKEPAVPKDGPTANI
AAQTFTFRELAAATKNFRPECLLGEGGFGRVYKGRLESTGQVVAVKQLDRNGLQGNREFLVEVLMLSLLHHPNL
VNLIGYCADGDQRLLVYEFMPLGSLEDHLHDLPSDKEPLDWNTRMRIAAGAAKGLEYLHDKANPPVIYRDLKSS
NILLDEGFHPKLSDFGLAKLGPVGDKTHVSTRVMGTYGYCAPEYAMTGQLTLKSDVYSFGVVFLELITGRKAIDN
TRAPGEQNLVAWARPLFRDRRKFPKMADPLLQGRYPIRGLYQALAVAAMCLQEQAATRPLIGDVVTALTYLAS
QTYDPNAPGSQSNRVGPSTQRIKDDRRSMADGLDSPDARGRVGSPSTHRNSPDYRKKNHVREMSSGAELSRN
EPGEGSGRKWGSLDESEQQEFHTDSPMNSARARETSRNRDLDRERAVAEAKVWGENWREKKRANAMGDSN
GRND

>A540|A0A5D2XNL3

MGGCFPCFGSSNKAGSNGGGSVKELSKKDSTKDSSVGQPHHVNRVNSDKSKSRSVSDPKKEPAVPKDGPTANI
AAQTFTFRELAAATKNFRPECLLGEGGFGRVYKGRLESTGQVVAVKQLDRNGLQGNREFLVEVLMLSLLHHPNL
VNLIGYCADGDQRLLVYEFMPLGSLEDHLHDLPSDKEPLDWNTRMRIAAGAAKGLEYLHDKANPPVIYRDLKSS
NILLDEGFHPKLSDFGLAKLGPVGDKTHVSTRVMGTYGYCAPEYAMTGQLTLKSDVYSFGVVFLELITGRKAIDN
TRAPGEQNLVAWARPLFRDRRKFPKMADPLLQGRYPIRGLYQALAVAAMCLQEQAATRPLIGDVVTALTYLAS
QTYDPNAPNNQSNRVGPSTPRVKDDRRSMADGLDSPDARGRVGSPSTHRNSPDYRKKNHVREMSSGKELSR
NEPGEGSGRKWGSLDESEQHESHTDSPMNSARARETSRNRDLDRERAVAEAKVWGENWREKKGANAMGDS
DGRND

>A541|LOC105116025

MGGCFPCFGSPNKEGSSGVGLVKELNKKDSLKEGSAVQSHHVGRVSSDKSKSRSGSDPKKEQSIPKDGTTPNIA
AQIFTFRELAAATKNFRPECLLGEGGFGRVYKGRIESTDQVVAVKQLDRNGLQGNREFLVEVLMLSLLHHPNLVN
LIGYCADGDQRLLVYEFMPLGSLEDHLHDLAPDKEPLDWNTRMKIAAGAAKGLEYLHDTANPPVIYRDLKSSNIL
LDEGFHPKLSDFGLAKLGPVGDKTHVSTRVMGTYGYCAPEYAMTGQLTLKSDVYSFGVVFLELITGRKAIDNTRA

PGEHNLVAWARPLFKDRRKFPKMADPLLQGRYPMRGLYQALAVAAMCLQEQAATRPLIGDVVTALTYLASQT
YDPNAANQSNRVGPSTPRNRDDRRGMADGLDSSDEHGRGGRNGSPSTYKNSPDYRKRDHGREFSTGAELGR
SETGGSGRKWGLDDSDQQDSQRDSPVSTSRARETPRNRDLDRERAVAEAKVWGENWREKRRANAMGSFDG
TNE

>A542|LOC107426910

MGGCFPCFRSSKKEGSDVKEVTKDSVKEGSATQSHHVTRVSSDKSKSRSGSDSKKEPPIPKDGPTAHIAAQTFTF
RELAAATKNFRPESLLGEGGFGRVYKGRLESTGQVVAVKQLDRNGLQGNREFLVEVLMLSLLHHPNLVNLIGYC
ADGDQRLLVYEFMPLGSLEDHLHDLPPDREPLDWNTRMKIAAGAAKGLEYLHDKANPPVIYRDLKSSNILLDEG
YHPKLSDFGLAKLGPVGDKTHVSTRVMGTYGYCAPEYAMTGQLTLKSDVYSFGVVFLELITGRKAIDNNRAPGE
HNLVAWARPLFKDRRKFPKMADPLLQGRYPMRGLYQALAVAAMCLQEQAATRPLIGDVVTALTYLASQTYDP
NAVSSHSNRVGPSTPRNRDDRRNMADGLDSPDEHGRGGRHGSPATHRNSPDFRRRDPARELSTGAELGRIET
GGGSGRKWGVDESEQHESQRDSPLSTGRARETPRNRDLDRERAVAEAKVWGENWREKKRANAMGSFDGTN
EKHR

>A543|LOC110673955

MGGCFPCFGSSNKEGSGGGAVKELAKKDSVKEGSVAQSHHVGRVSSDKSKSRNGSDPKKEPTIPKDGPTANIA
AQTFTFKELAAATKNFRPECLLGEGGFGRVYKGRLESTGQVVAVKQLDRNGLQGNREFLVEVLMLSLLHHPNLV
NLIGYCADGDQRLLVYEFMPLGSLEDHLHDLPPDKEPLDWNTRMKIAAGAAKGLEYLHDKANPPVIYRDLKSSNI
LLDEGYHPKLSDFGLAKLGPVGDKTHVSTRVMGTYGYCAPEYAMTGQLTLKSDVYSFGVVFLELITGRKAIDNTR
APGENNLVAWARPLFKDRRKFPKMADPLLQGRYPMRGLYQALAVAAMCLQEQAATRPLIGDVVTALTYLASQ
THDPNAANQSNRVGPSTPRNRDDPRGMADGVDSPDEHGRGGRHGSPSTHKNSPDYRRRDTVRELGTGSELR
RSDTGGGSGRKWGLDDSERQDSQRDSPVNASRARETPRNRDLDRERAVAMAKVWGENWREKRRANAMGS
FDGTNE

>A544|A0A061EVX3

MGGCFPCFGSSNKEGSNGGGTVKELSKKDSTKEGSVGQSHHVNRVSSDKSKSRSVSDPKKEPTVPKDGATANIA
AQTFTFRELAAATKNFRPECLLGEGGFGRVYKGRLESTGQVVAVKQLDRNGLQGNREFLVEVLMLSLLHHPNLV
NLIGYCADGDQRLLVYEFMPLGSLEDHLHDLPPDKEPLDWNTRMKIAAGAAKGLEYLHDKANPPVIYRDLKSSNI
LLDEGHHPKLSDFGLAKLGPVGDKTHVSTRVMGTYGYCAPEYAMTGQLTLKSDVYSFGVVFLELITGRKAIDNTR
APGEHNLVAWARPLFKDRRKFPKMADPLLQGRYPMRGLYQALAVAAMCLQEQAATRPLIGDVVTALTYLASQ
TYDPNAPSNQSNRVGPSTPRLKDDRRSMGDGLDSPDERGQHGSPSTHRNSPDYRKRNHAREMSTGTELGRSE
AGGGSGRKWGLDDSERQESQRDSPLNAVRARETPRNRDLDRERAVAEAKVWGENWREKKRANAMGSFDGT
ND

>A545|A0A0A0L562

MGGCFPCFGSSDEDGSSNGVKEATKKDTAKDGSTAQSHHVTRVGSDKSKSRSVSDAKKEPTIQKDGTTAHIAA
QTFTFRELATATKNFRSECLLGEGGFGRVYKGRLESTGQVVAVKQLDRNGLQGNREFLVEVLMLSLLHHPNLVN
LIGYCADGDQRLLVYEFMPLGSLEDHLHDLPPDKEPLDWNTRMKIAAGAAKGLEYLHDKANPPVIYRDLKSSNIL
LDEGYHPKLSDFGLAKLGPVGDKTHVSTRVMGTYGYCAPEYAMTGQLTLKSDVYSFGVVFLELITGRKAIDNTRG
PGEHNLVAWARPLFKDRRKFPKMADPLLQGRYPMRGLYQALAVAAMCLQEQAATRPLIGDVVTALTYLASQT
YDPNAAASQSNRMGGSTPRARDERRSFPDGLDSPDERGRGRGSPSNYRNSPDYRKKDFHRELSCGGTELSKIDT
GGGSGRKWGLDELERQESLRDSPVYAGRARETPRNRDLNRERAVAEAKVWGENWRERKRANAQGSFDGSQE

>A546|A0A0D3B1U3

MSWCCLPCFGSSAKDVDSKDSLKKEVSAKDGSVTQQSHRASLDKSKSRGGSEHKELTAPKEGPTANIAAQTFTF
RELAAATKNFRPECLLGEGGFGRVYKGRLESTGQIVAVKQLDRNGLQGNREFLVEVLMLSLLHHTNLVNLIGYCA
DGDQRLLVYEYMALGSLEDHLHDLPPGKEPLDWNTRMTIAAGAAKGLEYLHDKANPPVIYRDLKSSNILLGDGY
HPKLSDFGLAKLGPVGDKTHVSTRVMGTYGYCAPEYAMTGQLTLKSDVYSFGVVFLELITGRKAIDNARAPGEH
NLVAWARPLFKDRRKFPKMADPSLQGRYPMRGLYQALAVAAMCLQEQAATRPLIGDVVTALTYLASQTFDPN
APNGQNSRSGVGPPFIRTRDERRSLGDGSSLDSPAETRSRLGSPGTHKNSPDYRRRDMVREVNAGSEAGSEAG
GGSGRKWGFSDVEGTESQRGSPASVGRGGRGTPRNRDLDRERAVAEAKVWGENWRERKRGTNGPGSFDSS
ND

>A547|A0A1S3VJM3

MGGCFPCFGSSNKEDSGGVRVKEVPNKDSSFKEAASLVPQSHHPSRANTDKSKSSRSGADAKKEAPVPKDGPT
AHIAAQTFTFRELAAATKNFRPECLLGEGGFGRVYKGRLESTGQVVAVKQLDRNGLQGNREFLVEVLMLSLLHH
PNLVNLIGYCADGDQRLLVYEFMPLGSLEDHLHDLPPDKEPLDWNTRMKIAAGAAKGLEYLHDKANPPVIYRDL
KSSNILLDEGYHPKLSDFGLAKLGPVGDKTHVSTRVMGTYGYCAPEYAMTGQLTLKSDVYSFGVVFLELITGRKAI
DNTRAHGEHNLVAWARPLFKDRRKFPKMADPLLQGRYPMRGLYQALAVAAMCLQEQAATRPLIGDVVTALTY
LASQTYDPNAANQSNRVGPSTPRMRDDRRSLADGVDSPDRRLGSPSTHRNSPDFRKRDSRDASMGTELGSRM
DTGGGSGRKWGLDDYERQESQRDSPVSTGRARETPWNRDLDRERAVAEAKVWGENWREKKKANAMGSFD
ATNE

>A548|A0A1U8HRL7

MGGCFPCFGSSNKESNNGGTTVKELQNKDSTKDGSVGQFHHVNRVNSDKSKSQSGSDSKKEPAVPKDGPTAN
IAAQTFTFRELATATKNFRPECLLGEGGFGRVYKGRLESTGQVVAVKQLDRNGLQGNREFLVEVLMLCLLHHPNL
VNLIGYCADGDQRLLVYEFMPLGSLEDHLHDLPPGKEPLDWNTRMKIAAGAAKGLEYLHDKANPPVIYRDLKSA
NILLGEGYQPKLSDFGLAKLGPVGDKTHVSTRVMGTYGYCAPEYAMTGQLTLKSDVYSFGVVFLELITGRKAIDN
TRSHGEHNLVAWARPLFKDRRKFPQMVDPLLQGRYPMRGLYQALAVAAMCLQEQAATRPLIGDVVTALTYLA
SQTYDPNVASNQSNRVGPSTPRRKNDRGGMVDGLDSPDEHGQRGSPSSHRNSPDYRMRNHARKLSTGAELG
RNETGGGSGRKWGFEDSERHESHRGSPLNTTRTRETSRNRDFDRERAVAEAKVWGENWREKKRANAIGSFDS
TNG

>A549|A0A1U8I7U5

MGGCFPCLGSSNKETNNGGTTVKELQNKDSTKDGSVGQFHHVSRVNSDKSKYQSGSDSKKEPAVPKDGPMA
NIAAQTFTFRELATATKNFRPECLLGEGGFGRVYKGRLESTGQVVAVKQLDRNGLQGNREFLVEVLMLSLLHHP
NLVNLIGYCADGDQRLLVYEFMPLGSLEDHLHDLPPGKEPLDWNTRMKIAAGAAKGLEYLHDKANPPVIYRDLK
SANILLGEGYHPKLSDFGLAKLGPVGDKTHVSTRVMGTYGYCAPEYAMTGQLTLKSDVYSFGVVFLELITGRKAID
NARSHGEHNLVAWARPLFKDRRKFPQMVDPLLQGHYPMRGLYQALAVAAMCLQEQAATRPLIGDVVTALTYL
ASQTYDPNAASNQSNRVGPSTPRRKNDRRGMLDGLDSPDEHGQRGSPSSHRNSPDYRMRNHARKLSTGAEL
GRNETGGGSGRKWGFEDSERHESHRGSPLNTTRTRETSRNRDFDRERAVAEAKVWGENWREKKRANAIGSFD
STNG

>A550|A0A2P5YM33

MGGCFPCFGSSNKESNNGGTTVKELQNKDSTKDGSVGQFHHVNRVNSDKSKSQSGSDSKKEPAVPKDGPTAN
IAAQTFTFRELATATKNFRPECLLGEGGFGRVYKGRLESTGQVVAVKQLDRNGLQGNREFLVEVLMLCLLHHPNL

VNLIGYCADGDQRLLVYEFMPLGSLEDHLHDLPPGKEPLDWNTRMKIAAGAAKGLEYLHDKANPPVIYRDLKSA
NILLGEGYQPKLSDFGLAKLGPVGDKTHVSTRVMGTYGYCAPEYAMTGQLTLKSDVYSFGVVFLELITGRKAIDN
TRSHGEHNLVAWARPLFKDRRKFPQMVDPLLQGRYPMRGLYQALAVAAMCLQEQAATRPLIGDVVTALTYLA
SQTYDPNVASNQSNRVGPSTPRRKNDRRGMVDGLDSPDEHGQRGSPSSHRNSPDYRMRNHARKLSTGAELG
RNETGGGSGRKWGFEDSERHESHRGSPLNTTRTRETSRNRDFDRERAVAEAKVWGENWREKKRANAIGSFDS
TNG

>A551|A0A5D2THS7

MGGCFPCLGSSNKETNNGGTTVKELQNKDSTKDGSVGQFHHVSRVNSDKSKYQSGSDSKKEPAVPKDGPMA
NIAAQTFTFRELATATKNFRPECLLGEGGFGRVYKGRLESTGQVVAVKQLDRNGLQGNREFLVEVLMLSLLHHP
NLVNLIGYCADGDQRLLVYEFMPLGSLEDHLHDLPPGKEPLDWNTRMKIAAGAAKGLEYLHDKANPPVIYRDLK
SANILLGEGYHPKLSDFGLAKLGPVGDKTHVSTRVMGTYGYCAPEYAMTGQLTLKSDVYSFGVVFLELITGRKAID
NARSHGEHNLVAWARPLFKDRRKFPQMVDPLLQGHYPMRGLYQALAVAAMCLQEQAATRPLIGDVVTALTYL
ASQTYDPNAASNQSNRVGPSTPRRKNDRRGMLDGLDSPDEHGQRGSPSSHRNSPDNRMRNHARKLSTGAEP
GRNETGGGSGRKWGIEDSERHESHRGSPLNTTRTRETSRNRDFDRERAVAEAKVWGENWREKKRANAIGSFD
STNG

>A552|A0A5D2XXJ6

MGVCFPCFGSSNKESNNGGTTVKELQNKDSTKDGSVGQFHHVNRVNSDRSKSQSGSDSKKEPAVPKDGPTAN
IAAQTFTFRELATATKNFRPECLLGEGGFGRVYKGRLESTGQVVAVKQLDRNGLQGNREFLVEVLMLCLLHHPNL
VNLIGYCADGDQRLLVYEFMPLGSLEDHLHDLPPGKEPLDWNTRMKIAAGAAKGLEYLHDKANPPVIYRDLKSA
NILLGEGYQPKLSDFGLAKLGPVGDKTHVSTRVMGTYGYCAPEYAMTGQLTLKSDVYSFGVVFLELITGRKAIDN
TRSHGEHNLVAWARPLFKDRRKFPQMVDPLLQGRYPMRGLYQALAVAAMCLQEQAATRPLIGDVVTALTYLA
SQTYDPNVASNQSNRVGPSTPRRKNDRGGMVDGLDSPDEHGQRGSPSSHRNSPDYRMRNHARKLSTGAELG
RNETGGGSGRKWGFEDSERHESHRGSPLNTTRTRETSRNRDFDRERAVAEAKVWGENWREKKRANAIGSFDS
TNG

>A553|LOC111279144

MGGCFPCLGSSDKEGKNGGGSVKELGKKDSTKEGSVGQSHHVNRVNSEKSKSWSASEPKKEPAVPKDGPTANI
AAQTFTFRELAAVTKNFRPECLLGEGGFGRVYKGHLESTGQVVAVKQLDRNGLQGNREFLVEVLMLSLLHHPNL
VNLIGYCADGDQRLLVYEFMPLGSLEDHLHDLPPDKEPLDWNTRMKIAAGAAKGLEYLHDRANPPVIYRDLKSS
NILLDEGYHPKLSDFGLAKLGPVGDKTHVSTRVMGTYGYCAPEYAMTGQLTLKSDVYSFGVVFLELITGRKAIDN
MRASGEHNLVAWARPLFKDRRKFPKMADPLLQGRYPMRGLYQALAVAAMCLQEQAATRPLIGDVVTALTYLA
SQIYDPNAPSNQSNRVGPSTPRPKDDRRSMADGLDIPDERGQHGSPSTHRNSPDYRKRNHARELSTGAELVRN
ETDGGSGRKWGLDDSERQESHRGSPLNAVRAREIPRNRDLDREHAVAEAKVWGEKWREKKRANPMGSFDG
MND

>A554|LOC111306820

MGGCFPCFESSKKGGSNGGGTVKELSKKDSTKDGSVGLSHHVNRANSDKSKSRGASDSKKEPTVPKDGPTANIA
AQTFTFRELAAATKYFRPECLLGEGGFGRVYKGHLESTGQVVAVKRLDRNGLQGNREFLVEVLMLSLLHHSNLV
NLIGYCADGDQRLLVYEFMPLGSLEDHLHDFPPDKEPLDWNTRMKIAAGAAKGLEYLHDKANPPVIYRDLKSSNI
LLDEGYHPKLSDFGLAKLGPVGDKTHVSTRVMGTYGYCAPEYAMTGQLTLKSDVYSFGVVFLELITGRKAIDNAR
PPGEHNLVAWARPLFKDRRKFPKMADPLLQGRYPMRGLYQALAVAAMCLQEQAATRPLIGDVVTALTYLASQ
AYDPNAPSSQSNRVGPSTPRLKDDRRSMADGLDSPDEHGQHGSPSTHRNSPDYRKRNHARELSTGAELGRNET

GGRSGRKWGLDDSDRQESHRDSPLNAVRARETLRNHDLDRERAVAAAKVWGENWREKKYANALGSFDGTN
D

>A555|LOC18603450

MGGCFPCFGSSNKEGSNGGGTVKELSKKDSTKEGSVGQSHHVNRVSSDKSKSRSVSDPKKEPTVPKDGATANIA
AQTFTFRELAAATKNFRPECLLGEGGFGRVYKGRLESTGQVVAVKQLDRNGLQGNREFLVEVLMLSLLHHPNLV
NLIGYCADGDQRLLVYEFMPLGSLEDHLHDLPPDKEPLDWNTRMKIAAGAAKGLEYLHDKANPPVIYRDLKSSNI
LLDEGHHPKLSDFGLAKLGPVGDKTHVSTRVMGTYGYCAPEYAMTGQLTLKSDVYSFGVVFLELITGRKAIDNTR
APGEHNLVAWARPLFKDRRKFPKMADPLLQGRYPMRGLYQALAVAAMCLQEQAATRPLIGDVVTALTYLASQ
TYDLNAPSNQSNRVGPSTPRLKDDRRSMGDGLDSPDERGQHGSPSTHRNSPDYRKRNHAREMSTGTELGRSE
AGGGSGRKWGLDDSERQESQRDSPLNAVRARETPRNRDLDRERAVAEAKVWGENWREKKRANAMGSFDGT
ND

>A556|NIOBTv3_g34290

MGGCFPCFRSSNKESGNGVKEVVKKESFKDGSAAQSIHLSKVNSDKSKSRGSHDPKKDPAIPKDGPTAHIAAQT
FTFRELAAATKNFRPECLLGEGGFGRVYKGRLESTGQVVAVKQLDRNGLQGNREFLVEVLMLSLLHHPNLVNLIG
YCADGDQRLLVYEFMPLGSLEDHLHDLPPDKEPLDWNTRMKIAAGAAKGLEYLHDKANPPVIYRDLKSSNILLDE
GYHPKLSDFGLAKLGPVGDKTHVSTRVMGTYGYCAPEYAMTGQLTLKSDVYSFGVVFLELITGRKAIDNARSHG
EHNLVAWARPLFKDRRKFPKMADPLLQGRYPMRGLYQALAVAAMCLQEQASTRPLIGDVVTALSYLASQTYDP
NAVGAQSSRVGSSTPRSREDRLPSADGVDSPDEHCSAHHGSPSIQRNSPDSRKRDSARDFNTGIELRKIATNGGS
GRKWGLDESERPDSQKNSPVSAGRTRETPRNRDLDRERAVAEAKVWGENWRDKKKTSARGSSFDGMNE

>A557|XP_019235940.1

MGGCFPCFRSSNKESGNGVKEVVKKDSFKDGSAAQSIHLSKVNSDKSKSRGSHDPKKDPAIPKDGPTGHIAAQT
FTFRELAAATKNFRPECLLGEGGFGRVYKGRLESTGQVVAVKQLDRNGLQGNREFLVEVLMLSLLHHSNLVNLIG
YCADGDQRLLVYEFMPLGSLEDHLHDLPPDKEPLDWNTRMKIAAGAAKGLEYLHDKANPPVIYRDLKSSNILLDE
GYHPKLSDFGLAKLGPVGDKTHVSTRVMGTYGYCAPEYAMTGQLTLKSDVYSFGVVFLELITGRKAIDNARSHG
EHNLVAWARPLFKDRRKFPKMADPLLQGRYPMRGLYQALAVAAMCLQEQASTRPLIGDVVTALSYLASQTYDP
NAVGAQSNRVGSSTPRSREDRLHSADGVDSPDEHSSAHHGSPSIQRNSPDSRKRDSARDFNTGIELRKIATSGG
SGRTWGLDESERPDSQKNSPVSAGRTRETPRNRDLDRERAVAEAKVWGENWRDKKKTSARGSSFDGMNE

>A558|PAN06183.1

MGCFSCFDSPADEQLNPKLGGAGGYGGASSAPKDANGGSGGGRHGDRGYPDLQQAPMAAPRVEKLSAAGE
KARVKSNALAREASAPKDANGNVISAQTFTFRELATATRNFRPECMLGKGGFGRVYRGRLESTGQVVAIKQLNR
DGLQGNREFLVEVFMLRVLHHQNLVNLIGYCADGDQRLLVYEYMPFGSLEDHLHDLPLDKEALDWNTRMKIAA
GAAKGLEYLHDKANPPVIYRDFKSSNILLDESFHPKLSDFGLAKLGPVGDKSHVSTRVMGTYGYCAPEYAMTGQL
TVKSDVYSFGVVLLELITGRRAIDSTRPHGEQNLVSWARPLFNDRRKLPKMADPRLEGRYPMRGLYQALAVASM
CIQSEAASRPLIADVVTALSYLASQSYDPNAALASRKPGGDQRCKPGDNGRLVSRNDETSSSGHKSPGKDREDSH
RDLPGILNKDFDRERMVVEAKMWGDRERMVAEAKMWGDRERMVAEAKMWGENWRDKRRAENGQGSLD
SRTGNS

>A559|A0A445I8H2

MGGCFPCFGSSNKEGSGGGVRVKEVPNKDSSFKEAASVVPQSHLPSRVNSDKSKSRNGADIKKDTPVPKDGPT
AHIAAQTFTFRELAAATKNFRPECLLGEGGFGRVYKGRLESTGQVVAVKQLDRNGLQGNREFLVEVLMLSLLHH

PNLVNLIGYCADGDQRLLVYEFMPLGSLEDHLHDLPPDKEPLDWNTRMKIAAGAAKGLEYLHDKANPPVIYRDL
KSSNILLDEGYHPKLSDFGLAKLGPVGDKTHVSTRVMGTYGYCAPEYAMTGQLTLKSDVYSFGVVFLELITGRKAI
DNTRAHGEHNLVAWARPLFKDRRKFPKMADPLLQGRYPMRGLYQALAVAAMCLQEQAATRPLIGDVVTALTY
LASQTYEPNAANQSNRVGPSTPRIRDDRRSMADGVDSPDRRLGSPSTHRNSPDFRKRDSRDPSAATELGRIDIG
GGSGRKWGLDDNERQESQRDSPVNTARTRETPWNRDLDRERAVAEAKVWGENLREKKKANAMGSFDATND

>A560|A0A5D3CXP5

MGGCFPCFGSSDEDGSNGVKEATKKDTAKDGSTAQSHHVTRVSSDKSKSRSVSDAKKEPTVQKDGTTAHIAAQ
TFTFRELATATKNFRSECLLGEGGFGRVYKGRLESTGQVVAVKQLDRNGLQGNREFLVEVLMLSLLHHPNLVNLI
GYCADGDQRLLVYEFMPLGSLEDHLHDLPPDKEPLDWNTRMKIAAGAAKGLEYLHDKANPPVIYRDLKSSNILL
DEGYHPKLSDFGLAKLGPVGDKTHVSTRVMGTYGYCAPEYAMTGQLTLKSDVYSFGVVFLELITGRKAIDNTRG
PGEHNLVAWARPLFKDRRKFPKMADPLLQGRYPMRGLYQALAVAAMCLQEQAATRPLIGDVVTALTYLASQT
YDPNAAASQSNRMGGSTPRARDERRGFPDGLDSPDERGRGRGSPSNYRNSPDYRKKDFHRELSCGGTELSKID
TGGGSGRKWGLDELERQESLRDSPVYAGRARETPRNRDLNRERAVAEAKVWGENWRERKRANAQGSFDGSQ
E

>A561|A0A5J5AC98

MGGCFPCFRSSNKEGNGVKEVAKKDSVKEGSVAQSHHGSRVSSDKSKSRSGSDPKKEPAVPKVGSTAHIAAQT
FTFRELAAATRNFRPECLLGEGGFGRVYKGRLESTGQVVAVKQLDRDGLQGNREFLVEVLMLSLLHHPNLVNLIG
YCADGDQRLLVYEFMPLGSLEDHLHDLPPDKEPLDWNTRMKIAAGAAKGLEYLHDRANPPVIYRDLKSSNILLD
DGYHPKLSDFGLAKLGPVGDKTHVSTRVMGTYGYCAPEYAMTGQLTLKSDVYSFGVVFLELITGRKAIDNTRAP
GEHNLVAWARPLFKDRRKFPKMADPLLQGHYPMRGLYQALAVAAMCLQEQAATRPLIGDVVTALTYLASQTY
DPNAATTQSSRVGPSTPRNKDDRRTMANGMDSPGEPGSSGRHGSPSTHKNSPDFRKRDSERKLSTGAELGRSE
TSGGSGRKWGVDGLECQESQRDSPVSTGRTRETPRNRDLDRERAVAEAKVWGENWRGKKRANATGSFDGTN
E

>A562|LOC104706173

MSGCLPCFGSSAKDAASKDSVKKEVSAKDGSVTQSHHVSLDKSKSRRGLDQKKELTAPKEGPTAHIAAQTFTFRE
LAAATKNFRPECLLGEGGFGRVYKGRLETTGQIVAVKQLDRNGLQGNREFLVEVLMLSLLHHPNLVNLIGYCAD
GDQRLLVYEYMPLGSLEDHLHDLPLDKEPLDWNTRMTIAAGAAKGLEYLHDKANPPVIYRDLKSSNILLGDGYH
PKLSDFGLAKLGPVGDKTHVSTRVMGTYGYCAPEYAMTGQLTLKSDVYSFGVVFLELITGRKAIDNARAPGEHN
LVAWARPLFKDRRKFPKMADPSLQGRYPMRGLYQALAVAAMCLQEQAATRPLIGDVVTALTYLASQTFDPNA
PSGQNSRSSGGPPFIRTRDDRRSLGDGSSLDSPAETRSRLGSPSTHKNSPDYRRRDMVREVNAGSEGGSETGGG
SGRKWGLSDLEGPESQRGSPASVGRTSRGTPRNRDLDRERAVAEAKVWGENWRERKRATNGPGSFDSTND

>A563|LOC104735924

MSGCLPCFGSSAKDAASKDSVKKEVSAKDGSVTQSHHVSLDKSKSRRVLEQKKELTAPKEGPTAHIAAQTFTFRE
LAAATKNFRPECLLGEGGFGRVYKGRLETTGQIVAVKQLDRNGLQGNREFLVEVLMLSLLHHPNLVNLIGYCAD
GDQRLLVYEYMPLGSLEDHLHDLPPDKEPLDWNTRMTIAAGAAKGLEYLHDKANPPVIYRDLKSSNILLGDGYH
PKLSDFGLAKLGPVGDKTHVSTRVMGTYGYCAPEYAMTGQLTLKSDVYSFGVVFLELITGRKAIDNARAPGEHN
LVAWARPLFKDRRKFPKMADPSLQGRYPMRGLYQALAVAAMCLQEQAATRPLIGDVVTALTYLASQTFDPNA
PSGQNSRSSGGPPFIRTRDDRRSLGDGSSLDSPAETRSRLGSPSTHKNSPDYRRRDMVREVNAGSEGGSETGGG
SGRKWGLSDLEGPESQRGSPASVGRTSRGTPRNRDLDRERAVAEAKVWGENWRERKRATNGPGSFDSTND

>A564|LOC104770191

MSGCLPCFGSSAKDAASKDSVKKEVSAKDGSVTQSHHASLDKSKSRRGLEQKKELTAPKEGPTAHIAAQTFTFRE
LAAATKNFRPECLLGEGGFGRVYKGRLETTGQIVAVKQLDRNGLQGNREFLVEVLMLSLLHHPNLVNLIGYCAD
GDQRLLVYEYMPLGSLEDHLHDLPPDKEPLDWNTRMTIAAGAAKGLEYLHDKANPPVIYRDLKSSNILLGDGYH
PKLSDFGLAKLGPVGDKTHVSTRVMGTYGYCAPEYAMTGQLTLKSDVYSFGVVFLELITGRKAIDNARAPGEHN
LVAWARPLFKDRRKFPKMADPSLQGRYPMRGLYQALAVAAMCLQEQAATRPLIGDVVTALTYLASQTFDPNA
PSGQNSRSSGGPPFIRTRDDRRSLEDGSSLDSPAETRSRLGSPSTHKNSPDYRRRDMVREVNAGSEGGSETGGG
SGRKWGLSDLEGSESQRGSPASVGRTSRGTPRNRDLDRERAVAEAKVWGENWRERKRATNGPGSFDSTND

>A565|LOC111009260

MGGCFPCCGSSDEDGNGVKESAKKKDSAKDGSTAQAHHVSRVSSDKSKSRSVSDTKKEPTVQKDGTTAHIAAQ
TFTFRELATATKNFRPECLLGEGGFGRVYKGRLESTGQVVAVKQLDRNGLQGNREFLVEVLMLSLLHHPNLVNLI
GYCADGDQRLLVYEFMPLGSLEDHLHDLPPDKEPLDWNTRMKIAAGAAKGLEYLHDKANPPVIYRDLKSSNILL
DEGYHPKLSDFGLAKLGPVGDKTHVSTRVMGTYGYCAPEYAMTGQLTLKSDVYGFGVVFLELITGRKAIDNTRG
PGEHNLVAWARPLFKDRRKFPKMADPLLQGRYPMRGLYQALAVAAMCLQEQAATRPLIGDVVTALTYLASQT
YDPNAAASQSNRMSGSTPRARDERRGFPDGLDSPDERGRGRGSPSNYKNSPDYRKKELHRELSRGGTELGKLD
TGGGSGRKWGLDELERQESLRDSPVYTGRARETPRNRDLNRERAVAEAKVWGENWRERKRANAQGSFDGSQ
E

>A566|A0A0D9VA01

MSCFMCFGSAQEGDAKKPGADAKDARKDGSADRGVSRVGSVLMLRGLHTTITDFELYNTFCLDQSKSHGGLDS
KKDVVIHRDGNNQNIAAQTFTFRELASATKNFRQDCLLGEGGFGRVYKGHLETGQAVAVKQLDRNGLQGNREF
LVEVLMLSLLHHTNLVNLIGYCANGDQRLLVYEFMPLGSLEDHLHDLPPDKEPLDWNTRMKVAAGAAKGLEYL
HDKASPPVIYRDFKSSNILLGEGFHPKLSDFGLAKLGPVGDKTHVSTRVMGTYGYCAPEYAMTGQLTVKSDVYSF
GVVFLELITGRKAIDNTKPPGEQNLVAWARPLFKDRRKFPKMADPMLQGRFPMRGLYQALAVAAMCLQEQAT
TRPHIGDVVTALSYLASQTYDPNAPVQHSRSNSSTPRARNIAGWNDDRRSVRSPNHHSPDLRESARSSRAEVSR
TSSTGDSGRRSGLDDLDMTGSQMGSPAQTGRKRETRRTADRQRAIAEAKMWRENSRERKQLNGHGSFDSTN
E

>A567|A0A314ZJB8

MGGCFPCFGSSNKEGSGVKEVTKKDSVKEGSAAQSHHVTRVSSDKKSRNGSDHKKEPAIPKDGPTAHIAAQTFT
FRELAAATKNFRAECLLGEGGFGRVYKGRLESTGQVVAVKQLDRNGLQGNREFLVEVLMLSLLHHPNLVNLIGY
CADGDQRLLVYEFMPLGSLEDHLHDLPSDKEPLDWNTRMKIAAGAAKGLEYLHDKANPPVIYRDLKSSNILLDE
GFHPKLSDFGLAKLGPVGDKTHVSTRVMGTYGYCAPEYAMTGQLTLKSDVYSFGVVFLELITGRKAIDNTRGHG
EHNLVAWARPLFKDRRKFPKMADPLLQGRYPMRGLYQALAVAAMCLQEQAATRPLIGDVVTALTYLASQTYD
PNSASGHSNRVGPSTPRHKDERRNMADGLDSPDEPGRGGRHGSPSTHKNSPDYRRRDPIRDLSTGVELGRIET
GTGSGRRWGLDGLERQESQRDSPVSAGRARETPRNRDLDRERAVAEAKVWGENWREKKRANAMGSFDGTN
E

>A568|A0A5E4ESQ1

MGGCFPCFGSSNKEGSGVKEVTKKDSVKEGSAAQSHHVTRVSSDKKSRNGSDHKKEPAIPKDGPTAHIAAQTFT
FRELAAATKNFRAECLLGEGGFGRVYKGRLESTGQVVAVKQLDRNGLQGNREFLVEVLMLSLLHHPNLVNLIGY
CADGDQRLLVYEFMPLGSLEDHLHDFPSDNEPLDWNTRMKIAAGAAKGLEYLHDKANPPVIYRDLKSSNILLDE

GFHPKLSDFGLAKLGPVGDKTHVSTRVMGTYGYCAPEYAMTGQLTLKSDVYSFGVVFLELITGRKAIDNTRGHG
EHNLVAWARPLFKDRRKFPKMADPLLQGRYPMRGLYQALAVAAMCLQEQAATRPLIGDVVTALTYLASQTYD
PNSASGHSNRVGPSTPRHKDERRNMADGLDSPDEPGRGGRHGSPSTHKNSPDYRRRESIRDLNTGVELGRIET
GTGSGRRWGLDGLERQESQRDSPVSAGRARETPRNRDLDRERAVAEAKVWGENWREKKRANAMGSFDGTN
E

>A569|A0A5N6NR58

MAQPDRQLGDDRRSSNKEGNAVKEVSKKDIVKEGSVGHQSRHVGRVSSGKSKSLVNLDHKKDKMVTKDGQT
ANIAAQTFTFRELAAATKNFRPESLLGEGGFGRVYKGRLDSTGQVVAVKQLDRNGLQGNREFLVEVLMLSLLHH
SNLVNLIGYCADGDQRLLVYEFMPLGSLEDHLHDLPPDKEPLDWNTRMKIAAGAAKGLEYLHDKANPPVIYRDL
KSSNILLNEGFHPKLSDFGLAKLGPVGDKTHVSTRVMGTYGYCAPEYAMTGQLTLKSDVYSFGVVFLELITGRKAI
DNTRAPGEHNLVAWARPLFKDRRKFPKMADPLLQGRYPVRGLYQALAVAAMCLQEQAATRPLIGDVVTALTYL
ASQPYDPEAVRAERAIRGSGSGTPRSRADRRNPSNGGLDSLDVSMRPHGSPSTGKNSPDYRKINTRSDLGDGES
SNGGDGSGRRWGLVDDPDSQRDSPANTIRARNRDLDRERAVAEAKVWGENWRDRKRTNTRGGGSFDATNE

>A570|A0A5N6RCT3

MGGCFPCFGSSNKEESGVKEVAKKDSVKEGSAGQSHHVIRVSSDKSKSRSGSDPKKEPAIPKDGPTAHIAAQTFT
FRELATATKNFRPECLLGEGGFGHVYKGRLESTGQVVAVKQLDRNGLQGNREFLVEVLMLSLLHHPNLVNLIGY
CADGDQRLLVYEFMPLGSLEDHLHDLPQDKKPLDWNTRMKIAAGAAKGLEYLHDKANPPVIYRDLKSSNILLDE
VYHPKLSDFGLAKLGPVGDKTHVSTRVMGTYGYCAPEYAMTGQLTLKSDVYSFGVVFLELITGRKAIDNTRAPGE
HNLVAWARPLFKDRRKFPKMADPLLQGRYPMRGLYQALAVAAMCLQEQAATRPLIGDVVTALTYLASQTYDP
NSAASQNNRVGPSTPRSRDERRSLGDGLDSPDQPGRGGWNGSPSAYKNSPDFRKRDARESSSSAALARIETGG
GSGRKLGVDELERHESQRDSPVSTGRARETPRNRDLDRERAVAEAKVWGENWREKKRANVMGSFDATNE

>A571|LOC111997227

MGGCFPCFGSSNSEGSDVKEVTKKDSIKDGSTQSHHVSRVSSVISTEKSKSRSGSDPKKEPAIPKDGPTAHIAAQT
FTFRELAAATKNFRPESLLGEGGFGHVYKGRLESTGQVVAVKQLDRNGLQGNREFLVEVLMLSLLHHPNLVNLIG
YCADGDQRLLVYEFMPLGSLEDHLHDLPPDKEPLDWNTRMKIAAGAAKGLEYLHDKANPPVIYRDLKSSNILLDE
GFHPKLSDFGLAKLGPVGDKTHVSTRVMGTYGYCAPEYAMTGQLTLKSDVYSFGVVFLELITGRKAIDNTRAPGE
HNLVAWARPLFKDRRKFPKMADPLLKGRYPMRGLYQALAVAAMCLQEQAATRPLIGDVVTALTYLASQTYDPI
AAAAANNRVGPSTPRSRDERRGMADGLDSPDERGRLGSPSTHKNSPDFRKRDARESSTGTALARIETGGGSGR
KLGLDELERHESQRDSPVGTGRARETPRNRDLDRERAVAEAKVWGENWREKKRANAMGSFDGTYE

>A572|LOC102599236

MGGCFPCFGSSNKETGKDEVKKESFKDASSAAQSIHLTKVNSDKSKSRGSHDPKKDPTIPKDGPTAHIAAQTFTF
RELAAATKNFRPESLLGEGGFGRVYKGRLESTGQVVAVKQLDRNGLQGNREFLVEVLMLSLLHHPNLVNLIGYC
ADGDQRLLVYEFMPLGSLEDHLHDLPPNKEPLDWNTRMKIAAGAAKGLEYLHDKANPPVIYRDLKSSNILLDEG
YHPKLSDFGLAKLGPVGDKTHVSTRVMGTYGYCAPEYAMTGQLTLKSDVYSFGVVFLELITGRKAIDNTRSHGEH
NLVAWARPLFKDRRKFPKMADPLLQGRYPMRGLYQALAVAAMCLQEQASTRPLIGDVVTALSYLASQTYDPN
AVGAQSNRVGSSTPRSREDRLHSVDGVDSPEYSSAHHGSPSIQRNSPDSRKRDSARDFNTGIELRKIATSGGSGR
KWGLDESERPDSQRNSPVSAGRTRETPRNRDLDRERAVAEAKVWGENWRDKKKTNARGSSFDGIND

>A573|A0A2G2XQZ3

MGGCFPCFGSSNKETGKQVVKKESFKDASAPQSIHLSKVNSDKSKSRGSHDPKKDPAIPKDGPTAHIAAQTFTFR
ELAAATKNFRPECLLGEGGFGRVYKGRLESTGQVVAVKQLDRNGLQGNREFLVEVLMLSLLHHPNLVNLIGYCA
DGDQRLLVYEFMPLGSLEDHLHDLPPDKGPLDWNTRMKIAAGAAKGLEYLHDKANPPVIYRDLKSSNILLDEGY
HPKLSDFGLAKLGPVGDKTHVSTRVMGTYGYCAPEYAMTGQLTLKSDVYSFGVVFLELITGRKAIDNTRSHGEH
NLVAWARPLFKDRRKFPKMADPLLQGRYPMRGLYQALAVAAMCLQEQASTRPLIGDVVTALTYLASQTYDPN
AAGAQSNRVGSSTPRSREDRLHSVDGVDSPEHCSAHHGSPSIQRNSPDSRKRDYARDFNTGIELRKIATSGGSSR
KWGLDESERPDSQRNSPVSAGRTRETPRNRDLDRERAVAEAKVWGENWRDKKKTNARGSSFDGIND

>A574|A0A2T7F6Y3

MGCFSCFDSPADEQLNPKLGGAGGYGGASSAAAAYGGGRHGDRGYPDLQQAPMAAPRVEKLSAAGEKARVK
SNALAREASVPKDANGNVISAQTFTFRELATATRNFRPECFLGEGGFGRVYRGRLESTGQVVAIKQLNRDGLQG
NREFLVEVLMLSLLHHQNLVNLIGYCADGDQRLLVYEYMPFGSLEDHLHDLPLDKEALDWNTRMKIAAGAAKG
LEYLHDKANPPVIYRDFKSSNILLDESFHPKLSDFGLAKLGPVGDKSHVSTRVMGTYGYCAPEYAMTGQLTVKSD
VYSFGVVLLELITGRRAIDSTRPHGEQNLVSWARPLFNDRRKLPKMADPRLEGRYPMRGLYQALAVASMCIQSE
AASRPLIADVVTALSYLASQSYDPNAALASRKPGGDQRSKPGENGRLVCRNDETSSSGHKSPGKDREDSPRDLP
GILNKDFDRERMVAEAKMWGDRERMVAEAKMWGDRERMVAEAKMWGENWRDKRRAENGQGSLDSRTG
NS

>A575|A0A4P1RMD0

MGGCFPCFGSSKNEDNNNGVKEVVAKKESFKDASIPQSQYPTRVSSDKSKSRSGSDPKKEIPVVKDGPTAHIAA
QTFTFRELAVATKNFRPECLLGEGGFGRVYKGRLESTGQVVAVKQLDRNGLQGNREFLVEVLMLSLLHHPNLVN
LIGYCADGDQRLLVYEFMPLGSLEDHLHDLPPDKEPLDWNTRMKIAAGAAKGLEYLHDKANPPVIYRDLKSSNIL
LDEGYHPKLSDFGLAKLGPVGDKTHVSTRVMGTYGYCAPEYAMTGQLTLKSDVYSFGVVFLELITGRKAIDNTRS
HGEHNLVAWARPLFKDRRKFPKMADPLLQGHYPMRGLYQALAVAAMCLQEQAATRPLIGDVVTALTYLASQT
YDPNAPNLSNRLGGPSTPRSKDDRRNMGDGVDSPDHGRLGSPSTHRNSPDYRKRDGRDPSSGTELGRSVSNG
GSGRKWGLDDVERQESQRDSPVNTGRARETPRNRDLDRERAVAEARVWGENWREKKRANAMGSFDATN

>A576|A0A2R6RVA8

MGGCFPCFGSSNKDGNGVKEVAKKEFVKDPLAAQSVNRVNSDKSKSRSGSDAKKDLKKELSVQKDGSTAHIAA
QTFAFRELATATKNFKPECLLGEGGFGRVYKGRLESTGQVVAVKQLDRNGLQGNREFLVEVLMLSLLHHPNLVN
LIGYCADGDQRLLVYEFMSLGSLEDHLHDLPPNKEPLDWNTRMKIAAGAAKGLEYLHDKANPPVIYRDLKSSNIL
LGKGYYPKLSDFGLAKLGPVGDKTHVSTRVMGTYGYCAPEYAMTGQLTLKSDVYSFGVVFLEIITGRKAIDNTRA
AGEHNLVAWARPLFKDRRKFPKMADPLLQGRYPMRGLYQALAVAAMCLQEQAATRPLIGDVVTALTYLASQT
YDPNAPTAQNRAGPSTPRHKDDRRNMAEGQNGPDNSWRGGSYGSPSNHRNSPDFRKRDAMRELSELGRIET
GGGSGRKWGVGEVERQDSPRDSPVSAGRTRETPRNQDLDRESAVAEAKVWGENWRDRKRAMGSFDGTNE

>A577|A0A4D9BL07

MGCFSCFGSSKTEVNSGSNGVKEVSKKDSVKDGSAAQSNSNVNRVNSDKPGLRTGNDSKKKMTIPKEPNIAAQ
IFTFRELAAATKNFRPESLLGEGGFGRVYKGRLESTGQAVAVKQLDRNGLQGNREFLVEVLMLSLLHHPNFVNLI
GYCADGDQRLLVYEYMPLGSLEDHLHDVPPDKEPLDWNTRMKIAAGAAKGLEYLHDKANPPVIYRDLKSSNILL
DEGYFPKLSDFGLAKLGPVGDKTHVSTRVMGTYGYCAPEYAMTGQLTLKSDVYSFGVVFLEIITGRKAIDNMRSA
GEQNLVAWARPLFKDQRKFPKMADPLLQGRYPMRGLYQALAVAAMCLQEQAATRPLIGDIVMALTYLASQSY
DPNAPGSQSRGGSGTPRHRDARRNISDADSLEDVGRGGHYGSPSAHKNSPNFRMKDSVGDVNAGAELRRIDT
GGGSGRRWGVDEPDRSDSQWGSPASGGRTRETPKNRDLDRERAVAAAKVWGENWREKKRTNGPGDDAV

>A578|LOC110738028

MGGCFPCWGSSNQAGKTDKEAANKDGVKDNTSIIQSNQLGKVNSDKSKNRNGSEPKKEPAVSKDGSTAHIAA
QTFTFRELAAATKNFRPESLLGEGGFGRVYKGRLESTGQVVAVKQLDRNGLQGNREFLVEVLMLSLLHHPNLVN
LIGYCADGDQRLLVYEYMPLGSLEDHLHDIPPNKEPLDWNIRMKIAAGAAKGLEYLHDKANPPVIYRDLKSSNILL
DEGYHPKLSDFGLAKLGPVGDKTHVSTRVMGTYGYCAPEYAMTGQLTLKSDVYSFGVVFLELITGRKAIDNTRAP
GEHNLVAWARPLFKDRRKFPKMADPLLQGRYPMRGLYQALAVAAMCLQEQAATRPLIGDVVTALTYLASQTY
DPNAPNNQSGRVGPSTPRSRDDRRSYGDSLDSPDRRAARLSSPSTVRNSPDFRKRDHGRELSTGAELGRSEVGG
GSGRRWGLDDLERQGSQRGSPASAGRTPRNRDIDRERAVAEAKVWGENYREKKQANAMGSFDSSNE

>A579|LOC110739358

MGGCFPCWGSSNQAGKTDKEAANKDGVKENTSIIQSNQLGKVNSDKSKNRNGSDPKKEPAVSKDGSTAHIAA
QTFTFRELAAATKNFRPESLLGEGGFGRVYKGRLESTGQVVAVKQLDRNGLQGNREFLVEVLMLSLLHHPNLVN
LIGYCADGDQRLLVYEYMPLGSLEDHLHDIPPGKEPLDWNIRMKIAAGAAKGLEYLHDKANPPVIYRDLKSSNILL
DEGYHPKLSDFGLAKLGPVGDKTHVSTRVMGTYGYCAPEYAMTGQLTLKSDVYSFGVVFLELITGRKAIDNTRAP
GEHNLVAWARPLFKDRRKFPKMADPLLQGRYPMRGLYQALAVAAMCLQEQAATRPLIGDVVTALTYLASQTY
DPNAPNNQSGRVGPSTPRSRDDRRSYGDSLDSPDRRAARLSSPSTVRNSPDFRKRDHGRELSTGAELGRSEVGG
GSGRRWGLDDMERQESQRGSPASSGRTPRNRDIDRERAVAEAKVWGENYREKKKANAMGSFDSSNE

>A580|LOC110021573

MACLPCFGSLEKVEEKNREVKPGGRDFSKEAPAAPSSNQMTRVSSDKSKSRSFSESKKETSVHKEGNTRHIAAQT
FSFRELATATKNFRAECLLGEGGFGRVYKGRLESGQVVAVKQLDWNGLQGNREFLVEVLMLSLLHHPNLVNLIG
YCADGDQRLLVYEYMPLGSLEDHLHDLPSDKQPLDWNTRMKIAAGAAKGLEYLHDKANPPVIYRDFKSSNILLS
DEYHPKLSDFGLAKLGPVGDKTHVSTRVMGTYGYCAPEYAMTGQLTLKSDVYSFGVVFLELITGRKAIDNTRPTG
EQNLVAWARPLFKDRRKFPKMADPLLQGHYPMRGLYQALAVAAMCLQEQAATRPLIGDVVTALSYLASQTYD
PNAPPMQSSRVGPSTPRSREERRNHGGSSDSKSATDSPHRDSPKLRLRDSIKGMNLGADVGRGDGDGVSGRK
WGLEEFERQDSHRDSPNHVIRPRDSPKGLNRDVDRERAIAEAKVWGENWRERKRGNAPKCVDSTNV

>A581|LOC104101148

MGGCFPCFRSSNKESGNVVKEVVKKESFKDGSAAQSIHLSKVNSDKSKSCGSHDPKKDPAIPKDGPTAHIAAQTF
TFRELAAATKNFRPECLLGEGGFGRVYKGRLESTGQVVAVKQLDRNGLQGNREFLVEVLMLSLLHHPNLVNLIGY
CADGDQRLLVYEFMPLGSLEDHLHDLPPDKEPLDWNTRMKIAAGAAKGLEYLHDKANPPVIYRDLKSSNILLDE
GYHPKLSDFGLAKLGPVGDKTHVSTRVMGTYGYCAPEYAMTGQLTLKSDVYSFGVVFLELITGRKAIDNARSHG
EHNLVAWARPLFKDRRKFPKMADPLLQGRYPMRGLYQALAVAAMCLQEQASTRPLIGDVVTALSYLASQTYDP
NAVGAQSNRVGSSTPRSREDRLHSADGVDSPDEHSSAHHGSPSIQRNSPDSRKRDSARDFNTGIELRKIATSGG
SGRKWGLDESERPDSQKNSPVSAGRTRETPRNRDLDRERAVAEAKVWGENWRDKKKTKCKG

>A582|LOC105045239

MGCFPCFGSPDQEKEGKSEVKGGGDFKQEAPAAPSSHHVARVSSDKSKSMSGSESKKEASIPKEGNGAHIAAQ
TFTFRELAAVTQNFRADCFLGEGGFGRVYKGRLENGQVVAVKQLDRNGLQGNREFLVEVLMLSLLHHPNLVNLI
GYCADGDQRLLVYEFMPLGSLEDHLHDLPSDKEPLDWNTRMKIAAGAAKGLEYLHDKANPPVIYRDFKSSNILL
GEGYHPKLSDFGLAKLGPVGDKTHVSTRVMGTYGYCAPEYAMTGQLTVKSDVYSFGVVFLELITGRKAIDNTRP
AGEQNLVAWARPLFKDRRKFPKMADPLLQGHYPVRGLYQALAVAAMCLQEQAATRPLIGDVVTALSYLASQG

YDPNAPAQNNKVGPSTPRARDDRKSLGGGSHQHGVQSPHRNSPDFRQRDLIRGASYGADIGRGGAGGGSGR
TWGLDELETEVSQKDSPVDIGKERETPKNVHRDFNRERAVAEAKVWGENWRERKRTYAQGSFDSTNE

>A583|LOC109338885

MGGCFPCFGSSNNSDSNGVKEVVAKIESFKDASISQSQYPDSKSRSGSDPKKEIPIPKDGQTTHIAAQTFTFRELA
AATKNFRPECLLGEGGFGRVYKGRLENTGQVVAVKQLDRNGLQGNREFLVEVLMLSLLHHSNLVNLIGYCADG
DQRLLVYEFMPLGSLEDHLHDLPPDKEPLDWNTRMKIAVGAAKGLEYLHDKANPPVIYRDLKSSNILLDEGYHPK
LSDFGLAKLGPVGDKTHVSTRVMGTYGYCAPEYAMTGQLTLKSDVYSFGVVFLELITGRKAIDNTRSHGEHNLV
AWARPLFKDRRKFPKMADPLLQGRYPIRGLYQALAVAAMCLQEQAATRPLIGDVVTALTYLASQTYDPNASNLS
NRLGSSSTPRSRDGRRNMGDSVDSPDRGRLGSPSSYRNSPDYRKSHSRDPSSGTELGRSVSNGGSGRKWGLDD
VERQESHRDSPVNTGRARETPRNRDLDRERAVAEARVWGENWREKKRANAMGSFDATNE

>A584|LOC110109502

MGCFPCFGSLEEEEEEKKREVKTGGADFSKEASAAPSSNQMTRVSSDKSKSRSVSDSKKETLVPKEGNATHIAAQ
TFTFRELATATKNFKPECLLGEGGFGRVYKGRLESGQVVAVKQLDRNGLQGNREFLVEVLMLSLLHHPNLVNLIG
YCADGDQRLLVYEYMPLGSLEDHLHDLPSDKEPLDWNTRMKIAAGAAKGLEYLHDKANPPVIYRDFKSSNILLGE
EYHPKLSDFGLAKLGPVGDKTHVSTRVMGTYGYCAPEYAMTGQLTLKSDVYSFGVVFLELITGRKAIDNTRPAGE
QNLVAWARPLFKDRRKFPKMADPLLQGHYPMRGLYQALAVAAMCLQEQAATRPLIGDVVTALSYLASQTYDP
NAPATQSSRVGPSTPRSRDERKNHGSGSDSKSATDSPHRDSPKLRLRDSIKGMNLGTDSDGTPGRKWGLEELER
QESHRDSPNHLAKPRDSPKALNRDADRERAIAEAKVWGENWRERKRATMPKSFDSTNE

>A585|A0A1D6N201

MSCFVCFGSAAQDEEPRKPAAAGAGKDAAPDRAVARVGSDKSRSQGGPDSKKDLIIHKDGNSQNIAAQTFTFR
ELAAATKNFRQDCLLGEGGFGRVYKGRLENGQSFTSQAVAVKQLDRNGLQGNREFLVEVLMLSLLHHTNLVNLI
GYCADGDQRLLVYEFMPLGSLEDHLHVFICIDLPPDKEPLDWNTRMKIAAGAAKGLEHLHDKASPPVIYRDFKSS
NILLGEGFHPKLSDFGLAKLGPVGDKTHVSTRVMGTYGYCAPEYAMTGQLTVKSDVYSFGVVFLELITGRKAIDN
TKTQGEQNLVAWARPLFKDRRKFPKMADPMLQGRFPMRGLYQALAVAAMCLQEQAATRPHIGDVVTALSYL
ASQAYDPNAPVQHVRSNSSTPRARNPAGWNGDQRSVRSPNHHSPDPRRRDAAGASKYGAEVSRTSSTSDSG
RRSGLDDMDLTGSQVGSPAQTGRRREAPRGTDRQRAVAEARTWGENSRERTNGHGSFDSTHE

>A586|A0A1U8LZY5

MGGCFPCFGSSNKEANNNNNNGGTTVKELSKKDSTKDSSVPQSQHVNRDKSKSRNGSGSKRELPVVPKDGPT
ANIAAQTFTFRELAAATKNFRPECLLGEGGFGRVYKGRLESTGQVVAVKQLDRNGLQGNREFLVEVLMLSLLHH
PNLVNLIGYCTDGDQRLLVYEFMPLGSLEDHLHDLPLDKEPLDWNTRMKIAAGAAKGLEYLHDKANPPVIYRDL
KSSNILLHEGFHPKLSDFGLAKLGPVGDKTHVSTRVMGTYGYCAPEYAMTGQLTLKSDVYSFGVVFLELITGRKAI
DNTRAHGEHNLVAWARPLFKDRRKFPKMADPLLQGHYPMRGLYQALAVAAMCLQEQAATRPLIGDVVTALT
YLASQTYDPNAPGNQSNRVGPSIPRPKEDGLDSPKEHSHRNSPDYRKRNHARESSPGAELGRNETSGGSGRKW
GLDDSERHESHIDSPLNTSRPRERNRDLDREHAVAEAKVWGENLREKKRANAMGSFDGTND

>A587|A0A1U8MC84

MGGCFPCFGSSNKEANNNNNNGGTTVKELSKKDSTKDSSVPQSQHVNRDKSKSRNGSDSKRELPVVPKDGPM
ANIAAQTFTFRELAAATKNFRPECLLGEGGFGRVYKGRLESTGQVVAVKQLDRNGLQGNREFLVEVLMLSLLHH
PNLVNLIGYCADGDQRLLVYEFMPLGSLEDHLHDLPPDKEPLDWNTRMKIAAGAAKGLEYLHDKANPPVIYRDL
KSSNILLHEGFHPKLSDFGLAKLGPVGDKTHVSTRVMGTYGYCAPEYAMTGQLTLKSDVYSFSVVFLELITGRKAI

DNTRAHGEHNLVAWARPLFKDRRKFPKMADPLLQGHYPMRGLYQALAVAAMCLQEQAATRPLIGDVVTALT
YLASQTYDPNAPGNQSNRVGPSIPRPKEDGLDSPKEHSHRNSPDYRKRNHARESSTGEELGRNETSGGSGRKW
GLDDSERHESHRDNPLNTSRPREHNRDLDRERAVAEAKVWGENLREKKRANAMGSFDGTND

>A588|A0A5B6UGZ3

MGGCFPCFGSSNKEANNNNNNGGTTVKELSKKDSTKDSSVPQSEHVNRDKSKSRNGSDSKRELPVVPKDGPTA
NIAAQTFTFRELAAATKNFRPECLLGEGGFGRVYKGRLESTGQVVAVKQLDRNGLQGNREFLVEVLMLSLLHHP
NLVNLIGYCADGDQRLLVYEFMPLGSLEDHLHDLPPDKEPLDWNTRMKIAAGAAKGLEYLHDKANPPVIYRDLK
SSNILLHEGFHPKLSDFGLAKLGPVGDKTHVSTRVMGTYGYCAPEYAMTGQLTLKSDVYSFGVVFLELITGRKAID
NTRAHGEHNLVAWARPLFKDRRKFPKMADPLLQGQYPMRGLYQALAVAAMCLQEQAATRPLIGDVVTALTYL
ASQTYDPNAPGNQSNRVGPSIPRPKEDGLDSPKEHSHRNSPDYRKRNHARESSTGAELGRNETSGGSGRKWGL
DDSERHESHRDSPLNTSRPRERNRDLDRERAVAEAKVWGENLREKKRANAMGSFDGTND

>A589|A0A5D2TSE3

MGGCFPCFGSSNKEANNNNNNGGTTVKELSKKDSTKDSSVPQSQHVNRDKSKSRNGSDSKRELPVVPKDGPT
ANIAAQTFTFRELAAATKNFRPECLLGEGGFGRVYKGRLESTGQVVAVKQLDRNGLQGNREFLVEVLMLSLLHH
PNLVNLIGYCADGDQRLLVYEFMPLGSLEDHLHDLPLDKEPLDWNTRMKIAASAAKGLEYLHDKANPPVIYRDL
KSSNILLHEGFHPKLSDFGLAKLGPVGDKTHVSTRVMGTYGYCAPEYAMTGQLTLKSDVYSFGVVFLELITGRKAI
DNTRAHGEHNLVAWARPLFKDRRKFPKMADPLLQGHYPMRGLYQALAVAAMCLQEQAATRPLIGDVVTALT
YLASQTYDPNAPGNQSNRVGPSIPRPKEDGLDSPKEHSHRNSPDYRKRNHARESSPGAELGRNETSGGSGRKW
GLDDSERHESHIDSPLNTSRPRERNHDLDRERAVAEAKVWGENLREKKRANAMGSFDGTND

>A590|A0A5D2Y2R8

MGGCFPCFGSSNKEANNNNNNGGTTVKELSKKDSTKDSSVPQSQHVNRDKSKSRNGSDSKRELPVVPKDGPM
ANIAAQTFTFRELAAATKNFRPECLLGEGGFGCVYKGRLESTGQVVAVKQLDRNGLQGNREFLVEVLMLSLLHH
PNLVNLIGYCADGEQRLLVYEFMPLGSLEDHLHDLPPDKEPLDWNTRMKIAAGAAKGLEYLHDKANPPVIYRDL
KSSNILLHEGFHPKLSDFGLAKLGPVGDKTHVSTRVMGTYGYCAPEYAMTGQLTLKSDVYSFGVVFLELITGRKAI
DNTRAHGEHNLVAWARPLFKDRRKFPKMADPLLQGHYPMRGLYQALAVAAMCLQEQAATRPLIGDVVTALT
YLASQTYDPNAPGNQSNRVGPSIPRPKEDGLDSPKEHSHRNSPDYRKRNHARESSTGEELGRNETSGGSGRKW
GLDDSERHESHRDNPLNTSRPRERNRDLDRERAVAEAKVWGENLREKKRANAMGSFDGTND

>A591|LOC108456723

MGGCFPCFGSSNKEANNNNNNGGTTVKELSKKDSTKDSSVPQSQHVNRDKSKSRNGSDSKRELPVVPKDGPM
ANIAAQTFTFRELAAATKNFRPECLLGEGGFGRVYKGRLESTGQVVAVKQLDRNGLQGNREFLVEVLMLSLLHH
PNLVNLIGYCADGDQRLLVYKFMPLGSLEDHLHDLPPDKEPLDWNTRMKIAAGAAKGLEYLHDKANPPVIYRDL
KSSNILLHEGFHPKLSDFGLAKLGPVGDKTHVSTRVMGTYGYCAPEYAMTGQLTLKSDVYSFGVVFLELITGRKAI
DNTRAHGEHNLVAWARPLFKDRRKFPKMADPLLQGHYPMRGLYQALAVAAMCLQEQAATRPLIGDVVTALT
YLASQTYDPNAPGNQSNRVGPSIPRPKEDGLDSPKEHSHRNSPDYRKRNHARESSTGEELGRNETSGGSGRKW
GLDDSERHESHRDNPLNTSRPRERNRDLDRERAVAEAKVWGENLREKKRANAMGSFDGTND

>A592|LOC110690497

MGCFSCFESKEEERLNPTSGEKDDRKDASPSSNSNISKLSSGADRLKARNNVGPRRESSVPNDGPMANISSSAHT
FTFRELAVATKNFRDECFLGEGGFGRVYKGQLETGQVVAVKQLDRNGLQGNREFLVEVLMLSLLHNDNLVNLIG
YCADGDQRLLVYEYMALGSLEDHLFDLPPDKEPLDWNTRMKIAAGAAKGLEYLHDKANPPVIYRDFKSSNILLD

DGFHPKLSDFGLAKLGPTGDKSHVSTRVMGTYGYCAPEYAMTGQLTVKSDVYSFGVVFLELITGRRAIDSDRPH
GEQNLVAWARPLFNDRRKFLKLADPQLQSRFPTRGLYQALAVASMCIQENAAARPLIGDVVTALSYLANQAYD
PTMKDRSRDENGGRIARSEDGAGSGRKWDLEGSEKDDSPRETVRMLNKDSDRERAVAEAKMWGENWREKR
RQTLDLEGGSEKDDSPKETARMLNRDSDRERAVAEAKMWGENWREKRRQSAQGTNDNMEG

>A593|LOC110711532

MGCFSCFESKEEERLNPTSGEKDDRKDASPSSNSNISKLSSGADRLKARNNVGPRRESSVPNDGPMANISSSAHT
FTFRELAVATKNFRDECFLGEGGFGRVYKGQLETGQVVAVKQLDRNGLQGNREFLVEVLMLSLLHNDNLVNLIG
YCADGDQRLLVYEYMALGSLEDHLFDLPPDKEPLDWNTRMKIAAGAAKGLEYLHDKANPPVIYRDFKSSNILLDE
GFHPKLSDFGLAKLGPTGDKSHVSTRVMGTYGYCAPEYAMTGQLTVKSDVYSFGVVFLELITGRRAIDSDRPHGE
QNLVAWARPLFNDRRKFLKLADPQLQSRFPTRGLYQALAVASMCIQENAAARPLIGDVVTALSYLANQAYDPT
MKDRSRDENGGRIARGEDGASSGRKWDLEGSEKDDSPRETVRMLNKDSDRERAVAEAKMWGENWREKRRQ
TLDLEGGSEKDDSPKETARMLNRDSDRERAVAEAKMWGENWREKRRQSAQGTNDNIEG

>A594|103977711

MGCFTCFGSPSQREEEEEVKKKRREGKGGGDRKREPSSRRGSSDKLTARTGSDSKKEASASKEGNAGHIAAQTF
TFRELAAATKNFRQDCLLGEGGFGRVYRGRLENGQVVAVKQLDRNGLQGNREFLVEVLMLSLLHHPNLVNLIGY
CADGDQRLLVYEFMRLGSLEDHLHDIPADKEPLDWNTRMKIAAGAAKGLEYLHDKANPPVIYRDFKSSNILLSEG
YHPKLSDFGLAKLGPVGDKTHVSTRVMGTYGYCAPEYAMTGQLTVKSDVYSFGVVFLELITGRKAIDNTRPAGE
QNLVAWARPLFKERRKFPKMADPLLQGHYPMRGLYQALAVAAMCLQEQAATRPLIGDVVTALSYLASQTYDQ
NAATAQNTRVGPSTPRSRSGFDNQQAVHSPHRNSPDLRQRDLVKGVSKGAKHGRGGSGGGSGRKWGLDELE
TQESQMDSPVHVGRARDSPKNIDRDVVREHAVAEAKVWGENERERQQKNALGIFNGTHE

>A595|103994206

MGCFPCFGSASQREEEEVKKKNEGKAGGAYHKDSSAATASNHGSIDKSKSRMGSDTSKKASTPKECNADHIAA
KTFTFRELAAATQNFRQDCLLGEGGFGRVYKGRLENGQVVAVKQLDRNGLQGNREFLVEVLMLSLLHHPHLVN
LIGYCADGDQRLLVYEFMPLGSLEDHLHDIPADKEPLDWNTRMKIAAGAAKGLEYLHDKANPPVIYRDFKSSNIL
LGEGYHPKLSDFGLAKLGPVGDKTHVSTRVMGTYGYCAPEYAMTGQLTVKSDVYSFGVVFLELITGRKAIDNTRP
AGEQNLVAWARPLFKDRRKFPQMADPLLQGHYPARGLYQALAVAAMCLQEQAATRPLIGDVVTALSYLASQT
YDPNSATGQSTRFGPSTPRSRSGKDHQHAVHSPQRNSPDLRQRDPVKGLSKGAKVGRGGSGCGSGQKWGLEE
SETQESQMDSPVHIDGARDSPKNRDLVREHAVAEAKLWGEKWRERQRRNAPDSFESTHE

>A596|A0A1D6G4S8

MSCFVCFGSAAHDGESRKPPAAGAGKDVPPDRAVVRVGSDKSRSQGGPESKKDLIIHRDGNSQNIAAHTFTFR
ELAAATKNFRQDCLLGEGGFGRVYKGRLENGQSFTSQAVAVKQLDRNGLQGNREFLVEVLMLSLLHHTNLVNLI
GYCADGDQRLLVYEFMPLGSLEDHLHDLPPDKEPLDWNTRMKIAAGAAKGLEYLHDKTSPPVIYRDFKSSNILLG
EGFHPKLSDFGLAKLGPVGDKTHVSTRVMGTYGYCAPEYAMTGQLTVKSDVYSFGVVFLELITGRKAIDNTKTQ
GEQNLVAWARPLFKDRRKFPKMADPMLQGRFPMRGLYQALAVAAMCLQEQAATRPHIGDVVTALSYLASQA
YDPNAPVQHSRSNASTPRSRNPAAWNGDQRSVRSPNHHHSPDPRRRDPARASKYGAEVSRTSSASDSGRRSG
LDDMDPTGSQVGSPAQTGRRREAPRATDRQRAVGEARMWGENSRERTNGHGSFDSTHE

>A597|A0A1D6N1Z9

MSCFVCFGSAAQDEEPRKPAAAGAGKDAAPDRAVARVGSDKSRSQGGPDSKKDLIIHKDGNSQNIAAQTFTFR
ELAAATKNFRQDCLLGEGGFGRVYKGRLENGQSFTSQAVAVKQLDRNGLQGNREFLVEVLMLSLLHHTNLVNLI

GYCADGDQRLLVYEFMPLGSLEDHLHDLPPDKEPLDWNTRMKIAAGAAKGLEHLHDKASPPVIYRDFKSSNILL
GEGFHPKLSDFGLAKLGPVGDKTHVSTRVMGTYGYCAPEYAMTGQLTVKSDVYSFGVVFLELITGRKAIDNTKT
QGEQNLVAWARPLFKDRRKFPKMADPMLQGRFPMRGLYQALAVAAMCLQEQAATRPHIGDVVTALSYLASQ
AYDPNAPVQHVRSNSSTPRARNPAGWNGDQRSVRSPNHHSPDPRRRDAAGASKYGAEVSRTSSTSDSGRRSG
LDDMDLTGSQVGSPAQTGRRREAPRGTDRQRAVAEARTWGENSRERTNGHGSFDSTHE

>A598|A0A4Y7L911

MVGCFSCLGPSDKGENKGVKESSKKDGSGPQSRNTSKVSSEQPESQGGGVAKKEKLLPVDGLTPNIAAHTFKYQ
ELAAATNNFKEECVLGEGGFGRVYKGYLESTDQVVAVKQLDRDGLQGNREFLVEVLMLSLLDHSNLVKLIGYCA
DGDQRLLVYELMPLGSLDDHLYDLPPNKEPLNWITRMKIAEGAAKGLEYLHDKANPSVIYRDLKASNILLGEGFY
PKLSDFGLAKLGPTGDNTHVSTRVMGTYGYCAPEYAMTGQLTLKSDVYSFGVVLLELITGRKAIDNNRAVKERNL
VAWARPLFRDRRMFPEMADPLLQGRYPRRGLYQALAVAAMCLQEQAVSRPQIAEVVTALSYLTSQTNAAWD
KNKAGPSSNPSTMHIDKESGNEVGNGRENNVFESPRQNSPNLRHNREAGYEQGMKLRLNKSEEQSSHQDSSS
NLPKARETVKSPNNRDLDREHAVAEAKLWGENLRGRKRENSVGNSFDSTNGTIM

>A599|A0A1D6G4T1

MSCFVCFGSAAHDGESRKPPAAGAGKDVPPDRAVVRVGSDKSRSQGGPESKKDLIIHRDGNSQNIAAHTFTFR
ELAAATKNFRQDCLLGEGGFGRVYKGRLENGQAVAVKQLDRNGLQGNREFLVEVLMLSLLHHTNLVNLIGYCA
DGDQRLLVYEFMPLGSLEDHLHGLYLPPDKEPLDWNTRMKIAAGAAKGLEYLHDKTSPPVIYRDFKSSNILLGEG
FHPKLSDFGLAKLGPVGDKTHVSTRVMGTYGYCAPEYAMTGQLTVKSDVYSFGVVFLELITGRKAIDNTKTQGE
QNLVAWARPLFKDRRKFPKMADPMLQGRFPMRGLYQALAVAAMCLQEQAATRPHIGDVVTALSYLASQAYD
PNAPVQHSRSNASTPRSRNPAAWNGDQRSVRSPNHHHSPDPRRRDPARASKYGAEVSRTSSASDSGRRSGLD
DMDPTGSQVGSPAQTGRRREAPRATDRQRAVGEARMWGENSRERTNGHGSFDSTHE

>A600|A0A394DBS9

MASPSLHIFPIFSNMFFFFPQPYLSLVIHVDSKSRSGSDPKKEIPIPKDGQTTHIAAQTFTFRELAAATKNFRPECLL
GEGGFGRVYKGRLENTGQVVAVKQLDRNGLQGNREFLVEVLMLSLLHHSNLVNLIGYCADGDQRLLVYEFMPL
GSLEDHLHDLPPDKEPLDWNTRMKIAVGAAKGLEYLHDKANPPVIYRDLKSSNILLDEGYHPKLSDFGLAKLGPV
GDKTHVSTRVMGTYGYCAPEYAMTGQLTLKSDVYSFGVVFLELITGRKAIDNTRSHGEHNLVAWARPLFKDRRK
FPKMADPLLQGRYPIRGLYQALAVAAMCLQEQAATRPLIGDVVTALTYLASQTYDPNASNLSNRLGSSSTPRSRD
GRRNMGDSVDSPDRGRLGSPSSYRNSPDYRKSHSRDPSSGTELGRSVSNGGSGRKWGLDDVERQESHRDSPV
NTGRARETPRNRDLDRERAVAEARVWGENWREKKRANAMGSFDATNE

>A601|LOC104897507

MGCFSCFESKEEERLNPTSGDKDYNKDAPSNSNISKLSSGADRLKARNNVGPRQESSISKDGPDTNISSSAQTFTF
RELAIATKNFRDECFLGEGGFGRVYKGRLETGQVVAVKQLDRNGLQGNREFLVEVLMLSLLHQPNLVNLIGYCA
DGDQRLLVYEYMALGSLEDHLHDLPPDKEPLDWNTRMKIAAGAAKGLEYLHDKANPPVIYRDFKSSNILLDEGF
QPKLSDFGLAKLGPTGDKSHVSTRVMGTYGYCAPEYAMTGQLTVKSDVYSFGVVFLELITGRRAIDSDRPHGEQ
NLVAWARPLFNDRRKFLKLADPRLQGKFPTRGLYQALAVASMCIQENAAARPLIGDVVTALSYLANQAYDKDRS
RDENGGKIVRGEDGATSGRKWDLEGGSDDSPRETARMLNKDSERERAVAEAKMWGENLRDKRRQTLDLEGG
SEKDDSPKETARMLNRDSDRERAVAEAKMWGENLREKRRQSAQGTDDNLDG

>A602|A0A0D9ZYC0

MLGCRGLHPWLPKSKLEGGKAFLAVGTSSVAAEFVINGTSLGLGEGGLDSRKDAFIPRDANGQPIAAHTFTFREL
AAATKNFRQDCLLGEGGFGRVYKGHLENGQAVAVKQLDRNGLQGNREFLVEVLMLSLLHHDNLVNLIGYCAD
GDQRLLVYEFMPLGSLEDHLHDIPPDKEPLDWNTRMKIAAGAAKGLEFLHDKANPPVIYRDFKSSNILLGEGYHP
KLSDFGLAKLGPVGDKTHVSTRVMGTYGYCAPEYAMTGQLTVKSDVYSFGVVFLELITGRKAIDNTKPLGEQNLV
AWARPLFKDRRKFPKMADPLLAGRFPMRGLYQALAVAAMCLQEQAATRPFIGDVVTALSYLASQTYDPNAPV
QHSRSNASTPRARNRVGANFDQRRLHSPNHQQSPDLRKEGTTTSKYEAEVSRTNSGSGSGRRAGLDSMDVTG
SQMGSPAHAGRKRESSRSTDRQRAVAEAKTWGENSRERKWPNARGSFDSTNE

>A603|A0A199VU07

MGCFPCFGSSHGGEEGKGGAKGSGDVKKDASAAPSSRHVSRISSDKLKSRNGLDSKKEGSVQKEESTAHIAAQT
FTFRELAAATKNFRQDCLLGEGGFGRVYKGRLEGGQVVAVKQLDRNGLQGNREFLVEVLMLSLLHHANLVNLIG
YCADGDQRLLVYEFMPLGSLEDHLHDLPPDKEPLDWNTRMKIAAGAAKGLEHLHDKANPPVIYRDFKSSNILLG
EGFHPKLSDFGLAKLGPVGDKTHVSTRVMGTYGYCAPEYAMTGQLTVKSDVYSFGVVFLELITGRKAIDNTRPA
GEQNLVAWARPLFKDRRKFPKMADPLLQNRFPSRGLYQALAIAAMCLQEQAATRPLIGDVVTALSYLASQTHN
PNAASVQSTRSGPSIPRVREDRRSIRLPDAQNSVQSPNPNSPDLRRKESLKGANSGADSGRKSGLDESEMQEFQ
YGSPVHARKNRETQRNSDRQRAIAEAKLWGENWRERKQANTRGSFDSTNE

>A604|A0A3L6SHZ7

MSCFVCFGSAQDGEAKKPAAADAKDARKDGPPDRGMTRVGSDKSRSQGGSDSKKDIIIHRDGNSQNIAAQTF
TFRELAAATKNFRQDCLLGEGGFGRVYKGRLENGQAVAVKQLDRNGLQGNREFLVEVLMLSLLHHTNLVNLIGY
CADGDQRLLVYEFMPLGSLEDHLHDLPPDKGALDWNTRMKIAAGAAKGLEYLHDKASPPVIYRDFKSSNILLGE
GFHPKLSDFGLAKLGPVGDKTHVSTRVMGTYGYCAPEYAMTGQLTVKSDVYSFGVVFLELITGRKAIDNTRPQG
EQNLVAWARPLFKDRRKFPKMADPMLQGRFPMRGLYQALAVAAMCLQEQATTRPHIGDVVTALSYLASQAY
DPNAPVQHSRSNSSTPRARNPAGWNDDQRSMRSPNHHSPDLRRREAARASKYGAEVSRTSSTGDSGRRSGLD
DLDMTGSQVGSPAQTGRRRETPRAADRQRAIAEAKMWGEYSRERSNGHGSFDSTNE

>A605|LOC109724221

MGCFPCFGSSHEGEEGKGGAKGSGDVKKDASAAPSSRHMSRISSDKLKSRNGSDSKKEGSVQKEESTAHIAAQT
FTFRELAAATKNFRQDCLLGEGGFGRVYKGRLEGGQVVAVKQLDRNGLQGNREFLVEVLMLSLLHHANLVNLIG
YCADGDQRLLVYEFMPLGSLEDHLHDLPPDKEPLDWNTRMKIAAGAAKGLEHLHDKANPPVIYRDFKSSNILLG
EGFHPKLSDFGLAKLGPVGDKTHVSTRVMGTYGYCAPEYAMTGQLTVKSDVYSFGVVFLELITGRKAIDNTRPA
GEQNLVAWARPLFKDRRKFPKMADPLLQNRFPSRGLYQALAIAAMCLQEQAATRPLIGDVVTALSYLASQTHN
PNAASVQSTRSGPSIPRVREDRRSIRLPDAQNSVQSPNPNSPDLRRKESLKGANSGADSGRKSGLDESEMQEFQ
YGSPVHARKNRETQRNSDRQRAIAEAKLWGENWRERKQANTRGSFDSTNE

>A606|A0A078IUE7

MVDKEKKKTFLLRMESQFPSFLSGYSQSFWLFRSCSKGLCKFLITILHPLCQTLSGRRWVVSRVFIRVKESKPQKQS
QTIVSNNFSTLPSGGEKKLSSKSNVRSKRELLLPRDGLEQIAAHTFAFRELVAATMNFHPDTFLGEGGFGCVYKGR
LESTGQVVAVKQLDRNGLQGNREFLVEVLMLSLLHHPNLVNLIGYCADGDQRLLVYEFMPLGSLEDHLHDLPPD
KEALDWNTRMKIAAGAAKGLEFLHDKANPPVIYRDFKSSNILLGEGFHPKLSDFGLAKLGPTGDKSHVSTRVMG
TYGYCAPEYAMTGQLTVKSDVYSFGVVFLELITGRKAIDSEMPHGEQNLVAWARPMFNDRRKFIKLADPKLKGR
FPTRALYQALSVASMCIQEEAATRPLIADVVTALSYLANQAYDPNKNERGGGKLITRDDEGGGSEKEDSPRETAR
ILNRDIDRERAVAEAKMWGESLREKRRQSEQGTSESNSTGSK

>A607|A0A0E0DQR4

MGCFPCFGSGGKGEAKKGGGGGRKDGGSADRRVARVGSDKSKSQGGLESRKDAFIPRDANGQPIAAHTFTFR
ELAAATKNFRQDCLLGEGGFGRVYKGHLENGQAVAVKQLDRNGLQGNREFLVEVLMLSLLHHDNLVNLIGYCA
DGDQRLLVYEFMPLGSLEDHLHDIPPDKEPLDWNTRMKIAAGAAKGLEFLHDKANPPVIYRDFKSSNILLGEGYH
PKLSDFGLAKLGPVGDKTHVSTRVMGTYGYCAPEYAMTGQLTVKSDVYSFGVVFLELITGRKAIDNTKPLGEQNL
VAWARPLFKDRRKFPKMADPLLAGRFPMRGLYQALAVAAMCLQEQAATRPFIGDVVTALSYLASQTYDPNAP
VQHSRSNASTPRARNRVGGNFDQRRLHSPTHQQSPDLRKEGTTTSKYEAEVSRTNSGSGSGRRSGLDSMDVTG
SQMGSPAHAGRKKESSRSTDRQRAVAEAKTWGENSRERKWPNARGSFDSTNE

>A608|A0A0E0L1S5

MGCFPCFGSGGKGEAKKGGGGGLRKDGSADRRVARVGSDKSKSQGGLDSRKDAFIPRDANGQPIAAHTFTFR
ELAAATKNFRQDCLLGEGGFGRVYKGRLENGQVVAVKQLDRNGLQGNREFLVEVLMLSLLHHDNLVNLIGYCA
DGDQRLLVYEFMPLGSLEDHLHDIPPDKEPLDWNTRMKIAAGAAKGLEFLHDKANPPVIYRDFKSSNILLGEEYH
PKLSDFGLAKLGPVGDKTHVSTRVMGTYGYCAPEYAMTGQLTVKSDVYSFGVVFLELITGRKAIDNTKPLGEQNL
VAWARPLFKDRRKFPKMADPLLAGRFPMRGLYQALAVAAMCLQEQAATRPFIGDVVTALSYLASQTYDPNAP
VQHSRSNASTPRARNRGGGNFDQRRLHSPNHQKSPDLRREGTTTSRYEAEVSRTNSGSGSGRRSGLDSVDVTG
SQMGSPAYAGRKRESSRSTDRQRAVAEAKTWGENSRERKWPNARGSFDSTNE

>A609|A0A0E0L1S6

MGCFPCFGSGGKGEAKKGGGGGLRKDGSADRRVARVGSGAVGAKGGLDSRKDAFIPRDANGQPIAAHTFTFR
ELAAATKNFRQDCLLGEGGFGRVYKGRLENGQVVAVKQLDRNGLQGNREFLVEVLMLSLLHHDNLVNLIGYCA
DGDQRLLVYEFMPLGSLEDHLHDIPPDKEPLDWNTRMKIAAGAAKGLEFLHDKANPPVIYRDFKSSNILLGEEYH
PKLSDFGLAKLGPVGDKTHVSTRVMGTYGYCAPEYAMTGQLTVKSDVYSFGVVFLELITGRKAIDNTKPLGEQNL
VAWARPLFKDRRKFPKMADPLLAGRFPMRGLYQALAVAAMCLQEQAATRPFIGDVVTALSYLASQTYDPNAP
VQHSRSNASTPRARNRGGGNFDQRRLHSPNHQKSPDLRREGTTTSRYEAEVSRTNSGSGSGRRSGLDSVDVTG
SQMGSPAYAGRKRESSRSTDRQRAVAEAKTWGENSRERKWPNARGSFDSTNE

>A610|A0A0D3EYE6

MSCFLCFGSAQEGEAKKPGADSKDARKDGSADRGVSRVGSDKSRSHGGLDSKKDVVIQRDGNNQNIAAQTFT
FRELAAATKNFRQDCLLGEGGFGRVYKGRLETGQAVAVKQLDRNGLQGNREFLVEVLMLSLLHHTNLVNLIGYC
ADGDQRLLVYEFMPLGSLEDHLHDLPPDKEPLDWNTRMKIAAGAAKGLEYLHDKASPPVIYRDFKSSNILLGEGF
HPKLSDFGLAKLGPVGDKTHVSTRVMGTYGYCAPEYAMTGQLTVKSDVYSFGVVFLELITGRKAIDNTKPQGEQ
NLVAWARPLFKDRRKFPKMADPMLQGRFPMRGLYQALAVAAMCLQEQATTRPHIGDVVTALSYLASQTYDP
NAPVQHSRSNSSTPRARNLAGWNEDRRSVRSPNHHSPDLRREAARSSRAEVSRTSSTGDSGRRSGLDDLDMT
GSQMGSTAQTGRKRETPRTADRQRAIAEAKTWGENSRERKHPNGHGSFDSTNE

>A611|A0A0E0CCQ4

MSCFLCFGSAQEGEAKKPGADSKDARKDGSADRGVSRVGSDKSRSHGGLDSKKDVVIQRDGNNQNIAAQTFT
FRELAAATKNFRQDCLLGEGGFGRVYKGRLETGQAVAVKQLDRNGLQGNREFLVEVLMLSLLHHTNLVNLIGYC
ADGDQRLLVYEFMPLGSLEDHLHDLPPDKEPLDWNIRMKIAAGAAKGLEYLHDKASPPVIYRDFKSSNILLGEGF
HPKLSDFGLAKLGPVGDKTHVSTRVMGTYGYCAPEYAMTGQLTVKSDVYSFGVVFLELITGRKAIDNTKPQGEQ
NLVAWARPLFKDRRKFPKMADPMLQGRFPMRGLYQALAVAAMCLQEQATTRPHIGDVVTALSYLASQTYDP

NAPVQHSRSNSSTPRARNLAGWNEDRRSVRSPNHHSPDLRREAARSSRAEVSRTSSTGDSGRRSGLDDLDMT
GSQMGSPAQTGRKRETPSTADRQRAIAEAKTWGENSRERKHPNGHGSFDSTNE

>A612|A0A0E0JTB7

MSCFLCFGSAQEGEAKKTGADAKDARKDGSADRGVSRVGSDKSRSHGGLDSKKDVVIHRDGNNQNIAAQTFT
FRELAAATKNFRQDCLLGEGGFGRVYKGRLETGQAVAVKQLDRNGLQGNREFLVEVLMLSLLHHTNLVNLIGYC
ADGDQRLLVYEFMPLGSLEDHLHDLPPDKEPLDWNTRMKIAAGAAKGLEYLHDKASPPVIYRDFKSSNILLGEGF
HPKLSDFGLAKLGPVGDKTHVSTRVMGTYGYCAPEYAMTGQLTVKSDVYSFGVVFLELITGRKAIDNTKPQGEQ
NLVAWARPLFKDRRKFPKMADPMLQGRFPMRGLYQALAVAAMCLQEQATTRPHIGDVVTALSYLASQTYDP
NAPVQHSRSNSSTPRARNLAGWNDDRRSVRSPNHHSPDLRREAARSSRAEVSRTSSTGDSGRRSGLDDLDMT
GLQMGSPAQTGRKRETPRTADRQRAIAEAKTWGENSRERKQPNGHCSFDSTNE

>A613|A0A0D9VA02

MSCFMCFGSAQEGDAKKPGADAKDARKDGSADRGVSRVGSDQSKSHGGLDSKKDVVIHRDGNNQNIAAQTF
TFRELASATKNFRQDCLLGEGGFGRVYKGHLETGQAVAVKQLDRNGLQGNREFLVEVLMLSLLHHTNLVNLIGY
CANGDQRLLVYEFMPLGSLEDHLHDLPPDKEPLDWNTRMKVAAGAAKGLEYLHDKASPPVIYRDFKSSNILLGE
GFHPKLSDFGLAKLGPVGDKTHVSTRVMGTYGYCAPEYAMTGQLTVKSDVYSFGVVFLELITGRKAIDNTKPPG
EQNLVAWARPLFKDRRKFPKMADPMLQGRFPMRGLYQALAVAAMCLQEQATTRPHIGDVVTALSYLASQTY
DPNAPVQHSRSNSSTPRARNIAGWNDDRRSVRSPNHHSPDLRESARSSRAEVSRTSSTGDSGRRSGLDDLDMT
GSQMGSPAQTGRKRETRRTADRQRAIAEAKMWRENSRERKQLNGHGSFDSTNE

>A614|F2E500

MGCLPCFGSSSKGEPVKKGTAQKDVPSDRRATGVGSDKSKPQGLLESKKDTVIPREGNNQHIAAHTFTFRELAA
ATKNFRQDCLLGEGGFGRVYRGRLDNGQAVAVKQLDRNGLQGNREFLVEVLMLSLLHHDNLVNLIGYCADGD
QRLLVYEYMPLGSLEDHLHDIPPEKEPLDWNTRMKIAAGAAKGLEYLHDKASPPVIYRDFKSSNILLGEGFHPKLS
DFGLAKLGPVGDNTHVSTRVMGTYGYCAPEYAMTGQLTVKSDVYSFGVVFLELITGRKAIDNTKPHGEQNLVA
WARPLFKDRRKFPKMADPSLQGRFPMRGLYQALAVAAMCLQEQAATRPFIGDVVTALSYLASQAYDPNAPTQ
HSRSNASTPRTRDRGSVNGDQRRIRSPNHHSPDLRRKEATTSSKYEAEVSRNSSGGGSGRRSGLDDRDVTGSQ
QGSPAQTGKRRETSRTSERQRAIAEAKTWGENSRERKWPNARGSFDSTNE

>A615|A0A2H3YB92

MRRVLAPIVMIMNFSKLGVQHLNDKLKSRSGSESKKEASMPKEGNSAHIAAQIFTFRELAAATKNFRADCLVGE
GGFGRVYKGRLENGQVVAVKQLDRNGLQGNREFLVEVLMLSLLHHPNLVNLIGYCADGDQRLLVYEFMPLGSL
EDHLHDIPPDKEPLDWNTRMKIAAGAAKGLEYLHDKANPPVIYRDFKSSNILLGEGYHPKLSDFGLAKLGPVGDK
THVSTRVMGTYGYCAPEYAMTGQLTVKSDVYSFGVVFLELITGRKAIDNTRPDGEQNLVAWARPLFRDRRKFPK
MADPLLQGRYPMRGLYQALAVAAMCLQEQAATRPLIGDVVTALSYLASQTYDPNAPAAQSNKVGPSTPRNRG
DRKSLGCGTINQHALQSPQRNSPDFRQRDLIRGASYGADVGRGGVGGGSGRKWSLEELETQESQRDSPVHVG
KARESPKNVNRDLDRERAVAEAKVWGENWRERKQSYVQGGCDSTNE

>A616|A0A446QD39

MSCFSCFGPALEAEAGKPGPDAKDPRAKDGAAPDRAGSDKLRLQGGSDPKNNHLTIPRDGSSQNIAAQIFTFRE
LAAATKNFRQDCMLGEGGFGRVYKGRLESGQAVAVKQLDRNGLQGNREFLVEVLMLSLLHHTNLVNLIGYCAD
GDQRLLVYEFMPLGSLEDHLHDVPPEKEPLDWNTRMKIAAGAAKGLEHLHDKASPPVIYRDFKSSNILLGEGFHP
KLSDFGLAKLGPVGDNTHVSTRVMGTYGYCAPEYAMTGQLTVKSDVYSFGVVFLELITGRKAIDNTKPQGEQNL

VAWARPLFKDRRKFPKMADPMLQGRFPMRGLYQALAVAAMCLQEQATTRPHIGDVVTALSYLASQTYDPNA
PTQHTRSNSSTPRARNVGGRNSEQRNGRSPNHHSPRTSKHGGEVSRTSSTGGDSGRRSGLDEMDMAGSQAG
SPAQTGRKRETPRTADRQRAIADAKMWGENSRERKRPNGSFDSTNE

>A617|LOC102720044

MGCFPCFDSRLEEEEEVCYRKGAGGGGNGAAASSSGVGGGGGGGXXXXAPRVERISAAAGASGKGNATVKEL
SALKDANGNVISAQTFTFRQLAAATRNFREECFIGEGGFGRVYKGRLDGTGQVVAIKQLNRDGTQGNKEFLVEV
LMLSLLHHQNLVNLVGYCADGDQRLLVYEYMPLGSLEDHLHDLPPDKVPLDWNTRMKIAAGAAKGLEYLHDK
AQPPVIYRDFKSSNILLGEGFHPKLSDFGLAKLGPVGDKSHVSTRVMGTYGYCAPEYAMTGQLTVKSDVYSFGV
VLLELITGRKAIDSTRPNVEQNLVSWARPLFNDRRKLPKMADPGLEGRYPMRGLYQALAVASMCIQSEAASRPLI
ADVVTALSYLASQKYDPNATPSSKKVGGGGGGCEGGRAALSRNDEAGSSGHKSPSSKDSPRGPGPGPGPLPGIL
NDRERMVAEARMWGENWREKRRAATTGNSSSAQGSLDSPTETG

>A618|A0A0D9W4B4

MGCFPCFDSRQEEEELYYGKGGGGGGGGGNGAALSAAASSSGVGGGSGGGGGWREGTSTAAPRIEKISAGAR
GKGNASVKKELSALKDANGNVISAQTFTFRQLASATRNFREECFIGEGGFGRVYKGRLDGTGQVVAIKQLNRDG
TQGNKEFLVEVLMLSLLHHQNLVNLVGYCADGDQRLLVYEYMPLGSLEDHLHDLPPDKVPLDWNTRMKIAAG
AAKGLEYLHDKAQPPVIYRDFKSSNILLGEDFHPKLSDFGLAKLGPVGDKSHVSTRVMGTYGYCAPEYAMTGQLT
VKSDVYSFGVVLLELITGRKAIDGTRPHVEQNLVSWARPLFNDRRKLPKMADPGLEGRYPMRGLYQALAVASM
CIQSEAASRPLIADVVTALSYLASQKYDPNATPSSRKVGGGEGGRALSRNDEAGSSGHKSPSSKDSPREQLPGILN
DRERMVAEAKMWGENWREKRRAATSTSSNAQGSLDSPTETG

>A619|A0A0E0L1S7

MGCFPCFGSGGKGEAKKGGGGGLRKDGSADRRVARGGLDSRKDAFIPRDANGQPIAAHTFTFRELAAATKNFR
QDCLLGEGGFGRVYKGRLENGQVVAVKQLDRNGLQGNREFLVEVLMLSLLHHDNLVNLIGYCADGDQRLLVYE
FMPLGSLEDHLHDIPPDKEPLDWNTRMKIAAGAAKGLEFLHDKANPPVIYRDFKSSNILLGEEYHPKLSDFGLAKL
GPVGDKTHVSTRVMGTYGYCAPEYAMTGQLTVKSDVYSFGVVFLELITGRKAIDNTKPLGEQNLVAWARPLFK
DRRKFPKMADPLLAGRFPMRGLYQALAVAAMCLQEQAATRPFIGDVVTALSYLASQTYDPNAPVQHSRSNAST
PRARNRGGGNFDQRRLHSPNHQKSPDLRREGTTTSRYEAEVSRTNSGSGSGRRSGLDSVDVTGSQMGSPAYA
GRKRESSRSTDRQRAVAEAKTWGENSRERKWPNARGSFDSTNE

>A620|A0A3L6QCM3

MGCFPCFGPGREEELDYYGAKGGNGGAAGWAAASSSAAATAAGGSGAEAAVAVPPRAERIPAGADKLRAKG
NAGSKKELAVLKDANGNVISAQTFTFRQLASATKNFRDECFIGEGGFGRVYKGRLDGTGQVVAIKQLNRDGNQ
GNKEFLVEVLMLSLLHHQNLVSLVGYCADGDQRLLVYEYMPLGSLEDHLHDLPPGKEPLDWNTRMKIAAGAAK
GLEYLHDKAQPPVIYRDFKSSNILLGEGFHAKLSDFGLAKLGPVGDKSHVSTRVMGTYGYCAPEYAMTGQLTVKS
DVYSFGVVLLELITGRKAIDSTRPASEQNLVSWARPLFNDRRKLPRMADPGLEGRFPTRGLYQALAVASMCIQSE
AASRPLIADVVTALSYLANQIYDPSPANTTKKGGGDQRSRVCDSERAVSRNDDTGSSGHRSPSKDRDDSPREHA
TGTVNKGLERERMVAEAKMWGENWREKQRAAQGSLDSPKEGG

>A621|A0A2T7CU66

MGCFPCFGPGREEELEYYGGKGGGNGGAAGWAAASSSAAAAGGGGAEAAVEVPPRAERIPAGVDKSRAKGN
AGSKKELAVLKDANGNVISAQTFTFRQLAAATKNFRDECFIGEGGFGRVYKGRLDTTGQVVAIKQLNRDGNQG
NKEFLVEVLMLSLLHHQNLVSLVGYCADGDQRLLVYEYMPLGSLEDHLHDLPPGKEPLDWNTRMKIAAGAAKG

LEYLHDKAQPPVIYRDFKSSNILLGEGFHAKLSDFGLAKLGPVGDKSHVSTRVMGTYGYCAPEYAMTGQLTVKSD
VYSFGVVLLELITGRKAIDSTRPASEQNLVSWARPLFNDRRKLPKMADPGLEGRFPMRGLYQALAVASMCIQSE
AASRPLIADVVTALSYLANQIYDPSPVNITKKGGGDQRSRVGDSERAVSRNDGTGSSGHRSPSKDRDDSPREHA
TGTANKGLERERMVAEAKMWGENWREKQRAAQGSLDSPKGGG

>A622|A0A3L6PZQ8

MGCFPCFGPGREEELEYYGGKGGGNGGAAGWAAASSSAAAAGGGGAEAAVAVPPRAERIPAGADKSRTKGN
ASSKKELAVLKDANGNVISAQTFTFRQLAAATKNFRDECFIGEGGFGRVYKGRLDATGQVVAIKQLNRDGNQG
NKEFLVEVLMLSLLHHQNLVSLVGYCADGDQRLLVYEYMPLGSLEDHLHDLPPGKEPLDWNTRMKIAAGAAKG
LEYLHDKAQPPVIYRDFKSSNILLGEGFHAKLSDFGLAKLGPVGDKSHVSTRVMGTYGYCAPEYAMTGQLTVKSD
VYSFGVVLLELITGRKAIDSTRPASEQNLVSWARPLFNDRRKLPKMADPGLEGRFPTRGLYQALAVASMCIQSEA
ASRPLIADVVTALSYLANQIYDPSPANITKKGGADQRSRIGDSERAVSRNDDTGSSGHRSPSKDRDDSPREHATG
TANKGLERERMVAEAKMWGENWREKQRAAQGSLDSPKGGG

>A623|A0A0E0KPX7

MGCFPCFDSRMEEEEVYYGKRGAGGGGNGGGALSAAAAASSSSGVSAGGGGGGWEGTSTAAPRVEKISAGA
RGRGNATVKKELSALKDANGNVISAQTFTFRQLAAATRNFREECFIGEGGFGRVYKGRLDGNGQIVAIKQLNRD
GTQGNKEFLVEVLMLSLLHHQNLVNLVGYCADGDQRLLVYEYMPLGSLEDHLHDLPPDKVPLDWNTRMKIAA
GAAKGLEYLHDKAQPPVIYRDFKSSNILLGEDFHPKLSDFGLAKLGPVGDKSHVSTRVMGTYGYCAPEYAMTGQ
LTVKSDVYSFGVVLLELITGRKAIDSTIPHVEQNLVSWARPLFNDRRKLPKMADPGLEGRYPMRGLYQALAVAS
MCIQSEAASRPLIADVVTALSYLASQKYDPNATPSSKKAGGGEAGRVLSRNDEAGSSGHKSPSSKDSPREQLPGIL
NDRERMVAEAKMWGENWREKRRAAASSNAQGSLDSPTETG

>A624|A0A540KSI6

MGLCVWTVGKKVDKKSRNGSDPKKEPPIPKDGSTAHIAAQTFTFRELAAATKNFRPECLLGEGGFGHVYKGRLE
STGQVVAVKQLDRNGLQGNREFLVEVLMLSLLHHPNLVNLIGYCADGDQRLLVYEFMPLGSLEDHLHDLPSDKE
PLDWNTRMKIAAGAAKGLEYLHDKANPPVIYRDLKSSNILLDEGFHPKLSDFGLAKLGPVGDKTHVSTRVMGTY
GYCAPEYAMTGQLTLKSDVYSFGVVFLELITGRKAIDNTRGPGEHNLVAWARPLFKDRRKFPKMADPLLQGRYP
MRGLYQALAVAAMCLQEQAATRPLIGDVVTALTYLASQTYDPNAGTTPSNRGGSSTPRLRDERRNMGDGLDS
PDEYVRGGRHGSPATHKNSPDFRRRGPNRDLNTGVELGRIETGTGSGRRWGLDGLERQESQRDSPQSAGRAR
ETPRNRDLDRERAVAEAKVWGENWREKKRANAMGSFDGTNE

>A625|A0A5J9TXY7

MGCFSCFGSGREEGRHGAKGGGNGAAASSSAVGGGGRRQEEAAAVTAAPRAERIPAGADKARAKGNASSRK
ELSVLKDANGNTISAQTFTFRQLAAATRNFREECFIGEGGFGRVYKGRLDGSGQVVAIKQLNRDGTQGNKEFLV
EVLMLSLLHHQNLVNLVGYCADGDQRLLVYEYMPLGSLEDHLHDLPPDKEPLDWNTRMKIAAGAAKGLEYLHD
KAQPPVIYRDFKSSNILLGNGFHPKLSDFGLAKLGPVGDKSHVSTRVMGTYGYCAPEYAMTGQLTVKSDVYSFG
VVLLELITGRKAIDSTRPPGEQNLVSWARPLFNDRRKLPKMADPGLAGRYPTRGLYQALAVASMCIQSEAASRPL
IADVVTALSYLANQTYDPNAANTSKKAGGGDQLSRVVDSGRALSKNDDSGSSGHKSPNKDREDSPRERLRIANK
DLDRERMVAEAKMWGENWREKRRAAASTQGSQDSPTSGG

>A626|A0A2T7EEM9

MGCLPCFGSSGEGAAKKGGARKDGSSDRRVTRVGSASALDKSKPQGGSDSKKDAVILRDGNNQHIAAHTFTFR
ELAAATKNFRQDCLLGEGGFGRVYKGRLENGQVVAVKQLDRNGLQGNREFLVEVLMLSLLHHDNLVNLIGYCA

DGDQRLLVYEFMPLGSLEDHLHDIPPDKEPLDWNTRMKIAAGAAKGLEYLHDKASPPVIYRDFKSSNILLGEGFH
PKLSDFGLAKLGPVGDKTHVSTRVMGTYGYCAPEYAMTGQLTVKSDVYSFGVVFLELITGRKAIDNTKPHGEQN
LVAWARPLFKDRRKFPKMADPLLQGHFPMRGLYQALAVAAMCLQEQAATRPFIGDVVTALSYLASQTYDPNA
PVQHSRSNTSTPRASRGGGSNDQRRLRSPNHHSPDLRRREATTGSKYEAEVSRTNSGSGSGRRSGLDDVDTAG
LQLGSPGHAGRKRESPRTTERQLAVAAAKTWGENSRDRN

>A627|A0A3B6FWJ0

MSCFSCFGPALEAEAGKPGPDAKDGAAPDRAGSDKLRLQGGSDPKNNHLTIPRDGSSQNIAAQIFTFRELAAAT
KNFRQDCMLGEGGFGRVYKGRLESGQAVAVKQLDRNGLQGNREFLVEVLMLSLLHHTNLVNLIGYCADGDQR
LLVYEFMPLGSLEDHLHDVPPEKEPLDWNTRMKIAAGAAKGLEHLHDKASPPVIYRDFKSSNILLGEGFHPKLSD
FGLAKLGPVGDNTHVSTRVMGTYGYCAPEYAMTGQLTVKSDVYSFGVVFLELITGRKAIDNTKPQGEQNLVAW
ARPLFKDRRKFPKMADPMLQGRFPMRGLYQALAVAAMCLQEQATTRPHIGDVVTALSYLASQTYDPNAPTQH
TRSNSSTPRARNVGGRNSEQRNGRSPNHHSPRTSKHGGEVSRTSSTGGDSGRRSGLDEMDMAGSQAGSPAQ
TGRKRETPRTADRQRAIADAKMWGENSRERKRPNGSFDSTNE

>A628|F2EHA9

MGCFPCFDSSSDGELLYPKQGGGGGGGGGNGTGGRTAAAASSSGVGAREERPMVPPRVEKLPAGAEKARAK
GNAGMKELSDLRDANGNVLSAQTFTFRQLTAATRNFREECFIGEGGFGRVYKGRLDGGQVVAIKQLNRDGNQ
GNKEFLVEVLMLSLLHHQNLVNLVGYCADGEQRLLVYEYMPLGSLEDHLHDLPPDKEPLDWNTRMKIAAGAAK
GLEYLHDKAQPPVIYRDFKSSNILLGDDFHPKLSDFGLAKLGPVGDKSHVSTRVMGTYGYCAPEYAMTGQLTVKS
DVYSFGVVLLELITGRKAIDSTRPHGEQNLVSWARPLFNDRRKLPKMADPGLQGRYPMRGLYQALAVASMCIQ
SEAASRPLIADVVTALSYLASQIYDPNAIHASKKAGGDQRSRVSDSGRALLKNDEAGSSGHKSDRDDSPREPPPGI
LNDRERMVAEAKMWGANLREKTRAAANAQGSLDSPTETG

>A629|PAN19905.1

MGCLPCFGSSGEGAAKKGGARKDGSSDRRVTRVGSASALDKSKPQGGSDSKKDAVILRDGNNQHIAAHTFTFR
ELAAATKNFRQDCLLGEGGFGRVYKGRLENGQVVAVKQLDRNGLQGNREFLVEVLMLSLLHHDNLVNLIGYCA
DGDQRLLVYEFMPLGSLEDHLHDIPPDKEPLDWNTRMKIAAGAAKGLEYLHDKASPPVIYRDFKSSNILLGEGFH
PKLSDFGLAKLGPVGDKTHVSTRVMGTYGYCAPEYAMTGQLTVKSDVYSFGVVFLELITGRKAIDNTKPHGEQN
LVAWARPLFKDRRKFPKMADPLLQGHFPMRGLYQALAVAAMCLQEQAATRPFIGDVVTALSYLASQTYDPNA
PVQHSRSNTSTPRASRGGGSNDQRRLRSPNHHSPDLRRREATTGSKYEAEVSRTNSGSGSGRRSGLDDVDTAG
LQLGSPGHAGRKRESPRTTERQLAVATAKTWGENSRDRN

>A630|102701840

MSCFPCSGSSGKAGADAQCVAALSPSPRPAASAAPDRSNSRGAGIKKDDSVRRGGSSANGGPAQIFTFRELAVA
TKNFRKDCLLGEGGFGRVYKGQMENGQVIAVKQLDRNGLQGNREFLVEVLMLSLLHHPNLVRLIGYCADGDQ
RLLVYEYMLLGSLENHLHDRPPGKKPLDWNTRMKIAVGAAKGLEYLHDKANPPVIYRDFKPSNILLGEDYYPKLS
DFGLAKLGPVGDKTHVSTRVMGTYGYCAPEYAMTGQLTVKSDVYSFGVVFLELITGRKAIDHTQPAGEQNLVA
WARPLFRDRRKFCQMADPSLHGGYPKRGLYQALAVASMCLQEQATSRPLVADIVTALLYLASNHYDPNAPSTK
SSRTCPSTPKAKAHRRTTSVPDAQHAADSLNWNFPDLGRKETTRGEFEQDHSEGYGSGSSSGRNDGIDVPELLA
LHNGQNNSEADIYHKSTVKLDAHEKQRSRSGKGSRQF

>A631|A0A2K1ZKR9

MLAELIQTNQSPGVVLIKRRNHQFPIPKDGPTANIAAQTFTFRELATATKNFKPECLLGEGGFGRVYKGRLESTG
QAVAVKQLDRNGLQGNREFLVEVLMLSLLHHPNLVNLIGYCADGDQRLLVYEFMPLGSLEDHLHDLPPDKEPLD
WNTRMKIAAGAAKGLEYLHDKANPPVIYRDLKSSNILLDEGFHPKLSDFGLAKLGPVGDKTHVSTRVMGTYGYC
APEYAMTGQLTLKSDVYSFGVVFLELITGRKAIDNTRAPGEHNLVAWARPLFKDRRKFPKMADPLLQGCYPMR
GLYQALAVAAMCLQEQAATRPLIGDVVTALTYLASQTYDPNAASQSNRVGPSTPRNRDERRGMADGLDSPDE
HGLGGRRDSPSTYKNSPDYRKRDRVREFSTGAELGRSEAGSGSGRKWGLDDSERQDSQKDSLVTTSRARETPR
NRDLDRERAVAAAKVWGENWREKKRANAMGSFDGTNE

>A632|A0A1S3TTF7

MGCFSCFDSSSSSRENHNLRSHHHPQPNSNSNSNPNPNINLSLPSQISKLPSGADKLRSRSNGGSKRELPASNTK
EGHGPAVQIAAQTFTFRELAAATKNFRPESFVGEGGFGRVYKGRLETTGQIVAVKQLDKNGLQGNREFLVEVLM
LSLLHHPNLVNLIGYCADGEQRLLVYEFLPLGSLEDHLHDVPPDKEPLDWNTRMKIAAGAAKGLEYLHDKANPP
VIYRDFKSSNILLDETYHPKLSDFGLAKLGPVGDKSHVSTRVMGTYGYCAPEYAMTGQLTVKSDVYSFGVVFLELI
TGRKAIDSTQPHGEQNLVTWARPLFNDRRKFSKLADPRLQGRFPMRGLYQALAVASMCIQESAATRPLIGDVV
TALSYLANQGYDPNNAGHGYRGSSDDKRNRDDKGGRILKNDEAGGSGRRWDLEGSEKDDSPRETARMLNRD
LDRERAVAEAKMWGENLRQKRQQSLQEGSYGSN

>A633|A0A3B6AZ51

MGCFPCFDSGSDGELLYPKQGGGGGGNGTGGRTVSAASSSGVGAREERPMVPPRVEKLPAGAEKARAKGNA
GMKELSDLRDANGNVLSAQTFTFRQLTAATRNFREECFIGEGGFGRVYKGRLDGGQVVAIKQLNRDGNQGNK
EFLVEVLMLSLLHHQNLVNLVGYCADGEQRLLVYEYMPLGSLEDHLHDLPPDKEPLDWNTRMKIAAGAAKGLE
YLHDKAQPPVIYRDFKSSNILLGDDFHPKLSDFGLAKLGPVGDKSHVSTRVMGTYGYCAPEYAMTGQLTVKSDV
YSFGVVLLELITGRKAIDSTRPHGEQNLVSWARPLFNDRRKLPKMADPGLQGRYPMRGLYQALAVASMCIQSEA
ASRPLIADVVTALSYLASQIYDPNAIHASKKAGGDQRSRVSDSGRTLLKNDEAGSSGHKSDRDDSPREPPPGILND
RERMVAEARMWGANLREKTRAAANAQGSLDSPTEIG

>A634|Aco016040.1

MGCFPCFDSREEEQLNPKSGGCGVGGVEEEAKSGERPMIPPQIEKLSSGTDILKIRSNLGLKREPSGQKDGSGVN
ISAQTFTFRELTAATKNFRQECFLGEGGFGRVYKGRLESTGQIVAIKQLDRNGLQGNREFLVEVLMLSLLHHPNLV
NLIGYCADGDQRLLVYEYMPLGSLEDHLHDLPLDKEPLDWNTRMKIAAGAARGLEYLHDKANPPVIYRDFKSSNI
LLDEGFHPKLSDFGLAKLGPVGDKSHVSTRVMGTYGYCAPEYAMTGQLTVKSDVYSFGVVLLELITGRKAIDSTR
PHGEQNLVSWARPLFNDRRKLPKMADPRLDGRYPMRGLYQALAVASMCIQSEAASRPLIADVVTALSYLASQT
YDPSTAHHSSSNRSSGERRGGRFLNDDKVGKLPSRTDSPKDSNGDREDSPKEAPGILNKDFDRERAVAEAKMW
GENWREKRRAIANNEKQGANAADSPTKSG

>A635|A0A1S5VH72

MGCFPCFDSGSDGELLYPKQGGGGGGNGTGGRTVSAASSSGVGAREERPMVPPRVEKLPAGAEKARAKGNA
GMKELSDLRDANGNVLSAQTFTFRQLTAATRNFREECFIGEGGFGRVYKGRLDGGQVVAIKQLNRDGNQGNK
EFLVEVLMLSLLHHQNLVNLVGYCADGEQRLLVYEYMPLGSLEDHLHDLPPDKEPLDWNTRMKIAAGAAKGLE
YLHDKAQPPVIYRDFKSSNILLGDDFHPKLSDFGLAKLGPVGDKSHVSTRVMGTYGYCAPEYAMTGQLTVKSDV
YSFGVVLLELITGRKAIDSTRPHGEQNLVSWARPLFNDRRKLPKMADPGLQGRYPMRGLYQALAVASMCIQSEA
ASRPLIADVVTALSYLASQIYDPNAIHASKKAGGDQRSRVSDSGRTLLKNDEAGSSGHKSDRDDSPREPPPGILND
RERMVAEAKMWGANLREKTRAAANAQGSLDSPTETG

>A636|A0A0D2VNY0

MLTELIQVKSKSRSVSDPKKEPAVPKDGPTANIAAQTFTFRELAAATKNFRPECLLGEGGFGRVYKGHLESTGQV
VAVKQLDRNGLQGNREFLVEVLMLSLLHHPNLVNLIGYCADGDQRLLVYEFMPLGSLEDHLHDLPSDKEPLDW
NTRMRIAAGAAKGLEYLHDKANPPVIYRDLKSSNILLDEGFHPKLSDFGLAKLGPVGDKTHVSTRVMGTYGYCAP
EYAMTGQLTLKSDVYSFGVVFLELITGRKAIDNTRAPGEQNLVAWARPLFRDRRKFPKMADPLLQGRYPIRGLY
QALAVAAMCLQEQAATRPLIGDVVTALTYLASQTYDPNAPGNQSNRVGPSTPRVKDDRRSMADGLDSPDARG
RVGSPSTHRNSPDYRKKNHVREMSSGAELSRNEPGEGSGRKWGSLDESEQQESHTDSPMNSARARETSRNRD
LDRERAVAEAKVWGETWREKKRANAMGGSNGRND

>A637|A0A2T7EEM8

MGCLPCFGSSGEGAAKKGGARKDGSSDRRVTRVGSDKSKPQGGSDSKKDAVILRDGNNQHIAAHTFTFRELAA
ATKNFRQDCLLGEGGFGRVYKGRLENGQVVAVKQLDRNGLQGNREFLVEVLMLSLLHHDNLVNLIGYCADGD
QRLLVYEFMPLGSLEDHLHDIPPDKEPLDWNTRMKIAAGAAKGLEYLHDKASPPVIYRDFKSSNILLGEGFHPKLS
DFGLAKLGPVGDKTHVSTRVMGTYGYCAPEYAMTGQLTVKSDVYSFGVVFLELITGRKAIDNTKPHGEQNLVA
WARPLFKDRRKFPKMADPLLQGHFPMRGLYQALAVAAMCLQEQAATRPFIGDVVTALSYLASQTYDPNAPVQ
HSRSNTSTPRASRGGGSNDQRRLRSPNHHSPDLRRREATTGSKYEAEVSRTNSGSGSGRRSGLDDVDTAGLQLG
SPGHAGRKRESPRTTERQLAVAAAKTWGENSRDRN

>A638|A0A3L6REN8

MGCLPCFGSSGEGAAKKGGARKDGSSDRRVTRVGSDKSKPQGGSDSKKDAVILRDGNNQHIAAHTFTFRELAA
ATKNFRQDCLLGEGGFGRVYKGRLENGQVVAVKQLDRNGLQGNREFLVEVLMLSLLHHDNLVNLIGYCADGD
QRLLVYEFMPLGSLEDHLHDIPPDKEPLDWNTRMKIAAGAAKGLEYLHDKASPPVIYRDFKSSNILLGEGFHPKLS
DFGLAKLGPVGDKTHVSTRVMGTYGYCAPEYAMTGQLTVKSDVYSFGVVFLELITGRKAIDNTKPHGEQNLVA
WARPLFKDRRKFPKMADPLLQGHFPMRGLYQALAVAAMCLQEQAATRPFIGDVVTALSYLASQTYDPNAPVQ
HSRSNTSTPRASRGGGSNDQRRLRSPNHHSSDLRRREATTGSKYEAEVSRTNSGSGSGRRSGLDDVDTTGLQLG
SPGHAGRKRESPRTTERQLAVAEAKTWGENSRDRN

>A639|A0A3L6T2D3

MGCLPCFGSSGEGAAKKGGARKDGSSDRRVTRVGSDKSKPQGGSDSKKDAVILRDGNNQHIAAHTFTFRELAA
ATKNFRQDCLLGEGGFGRVYKGRLENGQVVAVKQLDRNGLQGNREFLVEVLMLSLLHHDNLVNLIGYCADGD
QRLLVYEFMPLGSLEDHLHDIPPDKEPLDWNTRMKIAAGAAKGLEYLHDKASPPVIYRDFKSSNILLGEGFHPKLS
DFGLAKLGPVGDKTHVSTRVMGTYGYCAPEYAMTGQLTVKSDVYSFGVVFLELITGRKAIDNTKPHGEQNLVA
WARPLFKDRRKFPKMADPLLQGRFPMRGLYQALAVAAMCLQEQAATRPFIGDVVTALSYLASQTYDPNAPVQ
HSRSNSSTPRVSRGGGSNDQRRLRSPNHHSPDLRRREATTGSKYEAEVSRTNSGSGSGRRSGLDDMDTTGLQL
GSPGHAGRKRESPRTTERQLAVAEAKTWGENSRDRN

>A640|A0A4U6VDW3

MGCLPCFGSAGEGAAKKGGARKDGSSDRRVTRVGSDKSKPQGGSDSKKDAVILREGNNQHIAAHTFTFRELAA
ATKNFRQDCLLGEGGFGRVYKGRLENGQVVAVKQLDRNGLQGNREFLVEVLMLSLLHHDNLVNLIGYCADGD
QRLLVYEFMPLGSLEDHLHDIPPDKEPLDWNTRMKIAAGAAKGLEYLHDKASPPVIYRDFKSSNILLGEGFHPKLS
DFGLAKLGPVGDKTHVSTRVMGTYGYCAPEYAMTGQLTVKSDVYSFGVVFLELITGRKAIDNGKPHGEQNLVA
WARPLFKDRRKFPKMADPLLQGRFPMRGLYQALAVAAMCLQEQAATRPFIGDVVTALSYLASQTYDPNAPVQ

HNRSNSSTPRASRGGGSNDQRRLRSPNHHSPDLRRREATTGPKYEAEVSRTNSGSGSGRQSGLDDVDMTVSQL
GSPGHGGRKRESPRTAERQGAIAEAKTWGENSRGRN

>A641|A0A5J9U690

MGCLPCFGSGGEGAAKKGGARKDASSDRRVSRDGSDKSKPQAGSDSKKDAVILREGNNQHIAAHTFTFRELAA
ATKNFRQDCLLGEGGFGRVYKGRLETGQAVAVKQLDRNGLQGNREFLVEVLMLSLLHHDNLVNLIGYCADGD
QRLLVYEFMPLGSLEDHLHDIPPEKEPLDWNTRMKIAAGAAKGLEYLHDKASPPVIYRDFKSSNILLGEGYHPKLS
DFGLAKLGPVGDKTHVSTRVMGTYGYCAPEYAMTGQLTVKSDVYSFGVVFLELITGRKAIDNTKPHGEQNLVA
WARPLFKDRRKFPKMADPLLQGRFPMRGLYQALAVAAMCLQEQAATRPFIGDVVTALSYLASQTYDPNAPVQ
HSRSNSSTPRASRGGGSSDQRRLRSPNHHSPDLRRREATTTSKYEAEVSRTNSGSGSGRRSGLDDVDMASLQVG
SPAHTGRKRESPRTSDRQRAVAEAKTWGENSRERK

>A642|Brast09G061900.1

MGCFSCFDSSSNKELGYPKHGGGSGNSTGGRAAAAESSGVGAREERPMVARRVDKLPAGVEKARTKGNAGM
KELSVLRDANGNALSAQTFTFRQLTAATRNFREECFIGEGGFGRVYKGRLDGSQVVAIKQLNRDGNQGNKEFLV
EVLMLSLLHHQNLVNLVGYCADGEQRLLVYEYMALGSLEDHLHDLPPDKESLDWNTRMKIAAGAAKGLEYLHD
KAQPPVIYRDFKSSNILLGDDFHPKLSDFGLAKLGPVGDKSHVSTRVMGTYGYCAPEYAMTGQLTVKSDVYSFG
VVLLELITGRKAIDSTRPHGEQNLVSWARPLFSDRRKLPKMADPGLQGRYPSRGLYQALAVASMCIQSEAASRPL
IADVVTALSYLAAQTYDPNAIHASKKAGSDQRSRVGDSGRVLLKNDEAGSSGDKSDRDDSPRERPQGILNDRER
MVAEAKMWGTNLREKTRAAANAQGNLDSPTETG

>A643|A0A4Y7JJR9

MGCFPCFDSKEEEKLNPGNKEESSDNVRKEVHHNHNNQPVVNPNVAKLSSGADRLRSRSTLGGSRRESSGPKE
GPYDHIAAQTFTFRELAAATKNFRPESLLGEGGFGRVYRGFLESTGQAVAVKQLDKDGLQGNREFLVEVLMLSLL
HHQNLVSLIGYCADGDQRLLVYEFMCLGSLEDHLHDLPPDKEALDWNTRMKIAAGAAKGLEYLHDKANPPVIY
RDFKSSNILLGEGFHPKLSDFGLAKLGPTGDKSHVSTRVMGTYGYCAPEYAMTGQLTVKSDVYSFGVVFLELITG
RKAIDSTQGHGEQNLVQWARPMFNDRRKFPKLVDPRLEGRFPMRGLYQALAVASMCIQESAATRPLIGDVVT
ALSYLATQSYDPNAPPSNKANGDKEDRRTSNERGGRIGKNEEGGGSGRKWDLEGSEKDDSPRETQRMLNRDL
DRERAVAEAKMWGENWREKKRQSAQGSFDGVNG

>A644|E3UPB0

MGCFPCFDSGSDGELLYPKQGGGNGTSGRTAPAASSSGVGAREERPMVPPRVEKLPAGAEKARAKGNAGMK
ELSDLRDANGNVLSAQTFTFRQLTAATRNFREECFIGEGGFGRVYKGRLDGGQVVAIKQLNRDGNQGNKEFLVE
VLMLSLLHHQNLVNLVGYCADGEQRLLVYEYMPLGSLEDHLHDLPPDKEPLDWNTRMKIAAGAAKGLEYLHDK
AQPPVIYRDFKSSNILLGDDFHPKLSDFGLAKLGPVGDKSHVSTRVMGTYGYCAPEYAMTGQLTVKSDVYSFGV
VLLELITGRKAIDSTRPHGEQNLVSWARPLFNDRRKLPKMADPGLQGRYPMRGLYQALAVASMCIQSEAASRPL
IADVVTALSYLASQIYDPNAIHASKKAGGDQRSRVSDSGRTLLKNDEAGSSGHKSDRDDSPREPPPGILNDRERM
VAEAKMWGANLREKTRAAANAQGSLDSPTETG

>A645|A0A1S3V6Z3

MGCFSCFDSREDEKLNPNPHQENHHHLHHHHDHNLNPPIPSRISRLPSASAAADKLRSSSNANGNNGDSKREL
AALKDGPAVQIAAQTFTFRELAAATKNFRPQSFLGEGGFGRVYKGRLETTGQAVAVKQLDRNGLQGNREFLVE
VLMLSLLHHPNLVNLIGYCADGDQRLLVYEFMPLGSLEDHLHDLPPEKEPLDWNTRMKIAAGAAKGLEYLHDKA
NPPVIYRDFKSSNILLDEDYHPKLSDFGLAKLGPVGDKSHVSTRVMGTYGYCAPEYAMTGQLTVKSDVYSFGVVF

LELITGRKAIDSTRPHGEQNLVTWARPLFNDRRKFPKLADPELHGRYPMRGLYQALAVASMCIQEQAAARPLIG
DVVTALSFLANQAYDHKGGGDDKRNRDDQGGRILKNDQGGGSGRRWDLEGSEKDESPRETARMLNNRDLDR
ERAVAEAKMWGENWREKRRQSAQGSFDGSNA

>A646|V7BAH9

MGCFSCFDSREDEKLNPNPHQENHQHHLHHQHHHHDHDLNPPIPSRISRLPSAAGADKLLSSTNGNANNGDS
KRELAILKDGNIAAQTFTFRELAAATKNFRPQSFLGEGGFGRVYKGRLETTGQAVAVKQLDRNGLQGNREFLVEV
LMLSLLHHPNLVSLIGYCADGDQRLLVYEFMPLGSLEDHLHDLPPDKEPLDWNTRMKIAAGAAKGLEYLHDKAN
PPVIYRDFKSSNILLDEGYHPKLSDFGLAKLGPVGDKSHVSTRVMGTYGYCAPEYAMTGQLTVKSDVYSFGVVFL
ELITGRKAIDSTRPHGEQNLVTWARPLFNDRRKFPKLADPELHGRYPMRGLYQALAVASMCIQEQAAARPLIGD
VVTALSFLANQAYEHKGGGDDKRNRDDQGGRILKNEQGGGSGRRWDLEGSEKDESPRETARILNNRDLDRER
AVAEAKMWGENWREKRRQSAQGSFDASNA

>A647|A0A0A0KFA2

MGCFPCFDSREEEKLNPEKESDDGKQDHPMVPPNIAKLPSGIDKLRSKSNGGSKREQQIPTPLVNISAQTFTFRE
LATATKNFRPECFIGEGGFGRVYKGRLESTSQIVAVKQLDRNGLQGNREFLVEVLMLSLLHHPNLVNLIGYCADG
DQRLLVYEFMPLGSLEDHLHEIPPEKEPLDWNTRMKIAAGAARGLEYLHDKANPPVIYRDFKSSNILLDEGYHPKL
SDFGLAKLGPVGDKSHVSTRVMGTYGYCAPEYAMTGQLTVKSDVYSFGVVFLELITGRRAIDSTRPQGEQNLVT
WARPFFNDRRRFSKLADPQLQGRYPMRGLYQALAVASMCTQEQAAARPLIGDVVTALSYLANQSYDPSSPSGT
AAAGTAAQRGSGDRDERRNRDEKGGGGMVKNEEGGASGRRWPLDGPEKDDSPRETARILNRDLDRERAVAE
AKMWGENWREKRRQSTQGSFDGSNG

>A648|LOC108480397

MGCFSCFDSKEEEKLNTVNETNDPKRPQPIVSSNISRLSSGGDRLRSRSNGGSKREIPSLRDGPGVQIAAQTFSFR
ELAAATKNFRPESFLGEGGFGRVYKGRLESTGQVITGQVVAVKQLDRNGLQGNREFLVEVLMLSLLHHPNLVNLI
GYCADGDQRLLVYEFMPLGSLEDHLHDLPPGKEPLDWNTRMKIAAGAAKGLEYLHDKANPPVIYRDFKSSNILL
EEGFHPKLSDFGLAKLGPVGDKSHVSTRVMGTYGYCAPEYAMTGQLTVKSDVYSFGVVFLELITGRKAIDSSQPH
GEQNLIAWARPLFNNRRKLSKLADPLLQGRYPMRGLYQALAVASMCIQEEAATRPLIGDVVTALSYLANQAYDP
NTTGNGHRGSGETDEKRYRDDRGGRVSKNDEGGVSGRKGDLEGSEKEDSPRETARMLNRDLDRERAVAEAK
MWGENWREKRRQSAQGSSDGSNG

>A649|LOC109357403

MGCFSCFESLEDEKLNTHSEQQYQHQQQQQQQQQQTNHNLRKHQPPTPPHISRMPSDKSQSRTNRDSKREE
PAPNAIHIPGVQIAAQTFTYRELAAATKNFRPESFIGEGGFGCVYKGWLESTNQIVAVKQLDRNGLQGNREFLVE
VLMLSLLHHTNLVNLIGYCADGDQRLLVYEFMPLGSLEDHLHDLPPDKEPLDWNTRMKIAAGAAKGLEYLHDKA
NPPVIYRDLKSANILLDEGFHPKLSDFGLAKLGPVGDKSHVSTRVMGTYGYCAPEYAMTGQLTVKSDVYSFGVVL
LELITGRKAIDSNQPHGEQNLVSWACPLINDRRKLSKLADPKLHGRLPMRGLYQALAVASMCIQESAAARPLIGD
VVTALSYLASQAYDPKGSSGDDKRNKDDKGGRILKNDEAGGSGLKWDLEGSEKDDSPRETARMIDRERAVAEA
KLWGENLREKRRQSAPPGNFNDSNT

>A650|AdPBS1XP_015962894.1

MGCFSCFDSREDQKLNPHGKHDHDHDHLRKQHQQQQQHPHPSNQALPSHISRLPSGADKLRSRSNGGSKREL
PAPKDGPGVQIAAQTFTFRELAAATKNFRPESFIGEGGFGRVYKGKLESTGQVVAVKQLDRNGLQGNREFLVEV
LMLSLLHHPNLVNLIGYCADGEQRLLVYEFMPLGSLEDHLHDLPPDKEPLDWNTRMKIAAGAAKGLEYLHDKAN

PPVIYRDFKSSNILLDEGYHPKLSDFGLAKLGPVGDKSHVSTRVMGTYGYCAPEYAMTGQLTVKSDVYSFGVVFL
ELITGRKAIDSTQPHGEQNLVTWARPLFNDRRKFSKLADPRLQGRFPMRGLYQALAVASMCIQESAATRPLIGD
VVTALSYLANQAYDPNNTGHGNRGSGDDKRNRDDKGARLLKNDEAGGSGHRWDLEGSEKDESPRETARMLN
RDLDRERAVAEAKMWGENWREKRRQS

>A651|LOC105051034

MGCFPCFDSKEAAQLNPGEGRDDRREEQPMVPPRVEKLCSGADRLKTRSHLDSKKESLGTKEGPGFNISAHTFT
FRELAVATKNFRPECFLGEGGFGRVYKGRLESTGQVVAVKQLDRNGLQGNREFLVEVLMLSLLHHPNLVNLIGY
CADGDQRLLVYEYMPLGSLEDHLHDLPPEKEPLDWNTRMKIAAGAAKGLEYLHDKANPPVIYRDFKSSNILLDE
GFHPKLSDFGLAKLGPVGDKSHVSTRVMGTYGYCAPEYAMTGQLTVKSDVYSFGVVLLELITGRKAIDSTRAHAE
QNLVSWARPMFNDRRKLPKLADPKLQGRYPMRGLYQALAVASMCIQEEAASRPLIADVVTALAYLASQAFDPN
AVPNSNSRSGGERRSRNSDEKGGRMLAKNDDGGSGRKWELDVDKEDSPRETIGILNRDFDRERAVAEAKMW
GENWREKRRVNANAQGAFDAASGNG

>A652|**LOC111488023**

MGCFPCFDSREEEKLNPERESDDGQQDHPMVPPNIAKLPSGTDKLGSKSNGGSKREQQIPKDAPLVNISAQTFT
FRELAIATKNFRPECFIGEGGFGRVYKGRLESTGQIVAVKQLDRNGLQGNREFLVEVLMLSLLHHPNLVNLIGYCA
DGDQRLLVYEFMPLGSLEDHLHEIPPDKEPLDWNTRMKIAAGAARGLEYLHDKANPPVIYRDFKSSNILLEEGYH
PKLSDFGLAKLGPVGDKSHVSTRVMGTYGYCAPEYAMTGQLTVKSDVYSFGVVFLELITGRRAIDSTRPQGEQN
LVTWARPFFNDRRRFSKLADPQLQGRYPMRGLYQALAVASMCTQEQAAARPLIGDVVTALSYLANQSYDPSSP
SGTAAAQRGSGDRDERQNSGEKGGGVVKNEEGGASGRRWTLDGPEKDDSPRETARILNRDLDRERAVAEAK
MWGENWREKRRQSTQGSFDGSNG

>A653|LOC111497667

MGCFPCFDSREEEKLNPEKESDDGKQHHPMIPPNIAKLPSGIDKLRSKGNGGSKREQQNPKDGPLVNISAQTFT
FRELAIATKNFRPECFIGEGGFGRVYKGRLESSGQIVAVKQLDRNGLQGNREFLVEVLMLSLLHHPNLVNLIGYCA
DGDQRLLVYEFMPLGSLEDHLHEIPPDKEPLDWNTRMKIAAGAARGLEYLHDKANPPVIYRDFKSSNILLEEGYH
PKLSDFGLAKLGPVGDKSHVSTRVMGTYGYCAPEYAMTGQLTIKSDVYSFGVVFLELITGRRTIDSTRPQGEQNL
VTWARPFFNDRRRFSKLADPQLQGRYPMRGLYQALAVASMCTQEQAAARPLIGDVVTALSYLANQSYDPSSPS
GTAAAAAQKGSGDRDERRNRDEKVAVKNEEGGACERRWTLDGPGKDESPRETGRILNKDSERERAVAEAKM
WGENWREKRRQSAQGSFDGSNG

>A654|I1NJF4

MGCFSCFDSREDEKLNPNPQQENHQHEHEHEHDLKPPVPSRISRLPPSASASASASVGADKLRSTTSNGNGEST
AVQIAAQTFSFRELAAATKNFRPQSFLGEGGFGRVYKGRLETTGQVVAVKQLDRNGLQGNREFLVEVLMLSLLH
HPNLVNLIGYCADGDQRLLVYEFMPFGSLEDHLHDLPPDKEPLDWNTRMKIAAGAAKGLEYLHDKANPPVIYR
DFKSSNILLDEGYHPKLSDFGLAKLGPVGDKSHVSTRVMGTYGYCAPEYAMTGQLTVKSDVYSFGVVFLELITGR
KAIDSTRPHGEQNLVTWARPLFSDRRKFPKLADPQLQGRYPMRGLYQALAVASMCIQEQAAARPLIGDVVTAL
SFLANQAYDHRGAGDDKKNRDDKGGRILKNDVGGGSGRRWDLEGSEKDDSPRETARMLNNRDLDRERAVAE
AKIWGENWREKRRQSAQGSFDGSNA

>A655|LOC110659892

MGCFPCFDSREEETLNPQKENDNRKQSLSTESSNISKLSSGADRLRSRSNGRSKRELPVPKDGLSGGNIAAQTFTF
RELAAATKNFRPESFIGEGGFGCVYKGRLESTGQVVAVKQLDRNGLQGNREFLVEVLMLSLLHHPNLVNLIGYCA

DGDQRLLVYEFMPLGSLEDHLHDLPPDKEPLDWNTRMRIAAGAAKGLEYLHDKANPPVIYRDFKSSNILLDEGFF
PKLSDFGLAKLGPTGDKSHVSTRVMGTYGYCAPEYAMTGQLTVKSDVYSFGVVFLELITGRKAIDSTRPHGEQNL
VTWARPLFNDRRKFSKLADPQLQGRYPMRGLYQALAVASMCIQEQAAARPLIGDVVTALSYLANQAYEPNSAG
HGYRGSGDKDEKRNRDERGGQLSKSEEGGGSSRRWDLDGSEKEDSPRETARMLNRDLDRERAVAEAKMWGE
NWREKRRQSAQGSSDGSNG

>A656|100279284

MSCLPCSGSSGKEDKSLAALSPSPRPAAKAAPVRSNSRASSSRKEDSVPVRRGGNAAHGPAQIFSFRELAVATKN
FRRDCLLGEGGFGRVYKGHMENGQVIAVKQLDRNGFQGNREFLVEVLMLSLLHHPNLVRLIGYCADGDQRLLV
YEYMLLGSLENRLFGPAGKEPLDWNTRMKIAAGAAKGLEYLHDKANPPVIYRDFKSSNILLGEDYYPKLSDFGLA
KLGPVGDKTHVSTRVMGTYGYCAPEYAMTGQLTLKSDVYSFGVVFLELITGRKAIDHTQPSGEQNLVAWARPLF
RDRRKFCQLADPLLHGGYPKRGLYQALAVAAMCLQEQAASRPLIGDVVTALSYLAAHPYDPNAPSTKDSKTCPS
TPRAKTHRRTTSVPDAQHAAESLILNFPDMRKETVRGGEFEKDRTEGSGSSSSSGRNDCLDVPRLLSVPNGKVC
GEGNNIQKSTVKVGAREN

>A657|A0A061E5W6

MGCFPCFDSSEEEKLTPVKETDDRKQGQPTVSSNISRLSSGGDRLRSRSNGGSKRELPSPRDGPGVQIAAQIFTFR
ELAAATKNFRPESFLGEGGFGRVYKGRLESTGQVVAVKQLDRNGLQGNREFLVEVLMLSLLHHPNLVNLIGYCA
DGDQRLLVYEFMPLGSLEDHLHDLPPEKEPLDWNTRMKIAAGAAKGLEYLHDKANPPVIYRDFKSSNILLDEGFH
PKLSDFGLAKLGPVGDKSHVSTRVMGTYGYCAPEYAMTGQLTVKSDVYSFGVVFLELITGRKAIDSTRPHGEQNL
VTWARPLFNDRRKFSKLADPRLQGRYPMRGLYQALAVASMCIQEQAATRPLIGDVVTALSYLANQAYDPNAVG
HGHRGPGDKDDKRYRDDRGGRVSRNDEGGASGRRWDLEGSEKEDSPRETARMLNRDLDRERAVAEAKMW
GENWREKRRQSAQGSFDGSNG

>A658|A0A1U8JAI1

MGCFSCFDSKEEEKLNTVNESNDPKQAQPTVSSNISRLSSGGDRLRSRSNGGSKRELPCPRDGPGVQIAAHIFSF
RELAAATKNFRPESFLGEGGFGHVYKGQLESTGQVVAVKQLDRNGLQGNREFLVEVLMLSLLHHHNLVNLIGYC
ADGDQRLLVYEFMPLGSLEDHLHDLPPGKEPLDWNTRMKIAAGAAKGLEYLHDKANPPVIYRDFKSSNILLDEG
FHPKLSDFGLAKLGPVGDKSHVSTRVMGTYGYCAPEYAMTGQLTVKSDVYSFGVVFLELITGRKAIDSTRPHGEQ
NLITWARPLFTNRRKLSKLADPLLQGRFPMRGLYQALAVASMCIQEEAATRPHIGDVVTALSYLANQAYEPNAS
GHGHSGSGETDEKRYRDDRGGRVSKNDEGGVSGCRWDLEGSEKDDSPKESARMLNRDLDRERAVAEAKMW
GENWREKRRQSAQGSSDGSNG

>A659|A0A1U8JAK4

MGCFSCFDSKEEEKLNTVNETNDPKRPQPIVSSNISRLSSGGDRLRSRSNGGSKREIPSLRDGPGVQIAAQTFSFR
ELAAATKNFRPESFLGEGGFGRVYKGRLESTGQVVAVKQLDRNGLQGNREFLVEVLMLSLLHHPNLVNLIGYCA
DGDQRLLVYEFMPLGSLEDHLHDLPPGKEPLDWNMRMKIAAGAAKGLEYLHDKANPPVIYRDFKSSNILLEEGF
HPKLSDFGLAKLGPVGDKSHVSTRVMGTYGYCAPEYAMTGQLTVKSDVYSFGVVFLELITGRKAIDSSQPHGEQ
NLIAWARPLFNNRRKLSKLADPLLQGRYPMRGLYQALAVASMCIQEEAATRPLIGDVVTALSYLANQAYDPNTT
GNGHRGSGETDEKRYRDDRGGRVSKNDEGGVSGRKGDLEGSEKENSPRETARMLNRDLDRERAVAEAKMW
GENWREKRRQSAQGSSDGSNG

>A660|A0A2P5XSN4

MGCFSCFDSKEEEKLNAVNETNDPKRPQPIVSSNISRLSSGGDRLRSRSNGGSKREIPSLRDGPGVQIAAQTFSFR
ELAAATKNFRPESFLGEGGFGRVYKGRLESTGQVVAVKQLDRNGLQGNREFLVEVLMLSLLHHPNLVNLIGYCA
DGDQRLLVYEFMPLGSLEDHLHDLPPGKEPLDWNTRMKIAAGAAKGLEYLHDKANPPVIYRDFKSSNILLEEGFH
PKLSDFGLAKLGPVGDKSHVSTRVMGTYGYCAPEYAMTGQLTVKSDVYSFGVVFLELITGRKAIDSSQPHGEQN
LIAWARPLFNNRRKLSKLADPLLQGRYPMRGLYQALAVASMCIQEEAATRPLIGDVVTALSYLANQAYDPNTTG
NGHRGSGETDEKRYRDDRGGRVSKNDEGGVSGRKGDLEGSEKEDSPRETARMLNRDLDRERAVAEAKMWGE
NWREKRRQSAQGSSDGSNG

>A661|A0A540NDR2

MGCFPCFDSKEEEKLNNPAAEIDDRKQGQPTVSNNISRLPSGVDRLRSRSNGGSRRELPKLPDPKDVVPGGQIA
AQTFTFRELATATKNFRPESFIGEGGFGRVYKGRLESTGQVVAVKQLDRNGLQGNREFLVEVLMLSLLHHPNLV
NLIGYCADGDQRLLVYEFMPLGSLEDHLHDLPSDKEPLDWNTRMKIASGAAKGLEYLHDKANPPVIYRDFKSSNI
LLEEGFHPKLSDFGLAKLGPTGDKSHVSTRVMGTYGYCAPEYAMTGQLTVKSDVYSFGVVFLELITGRKAIDSDR
PHGEQNLITWARPLFNDRRKFAKLADPRLQGRYPMRGLYQALAVASMCIQEQAATRPLIGDVVTALSYLANQS
YDPNLASGHGHRGSGEKDEKRHRDGGRILKNEEGGGSGRRWDLDGSEKDDSPKETVRMLDRERAVAEAKMW
GENWREKRRLSAQGSFDGTNL

>A662|A0A5D3ADH4

MGCFSCFDSKEEEKLNTVNESNDPKQAQPTVSSNISRLSSGGDRLRSRSNGGSKRELPCPRDGPGVQIAAHIFSF
RELAAATKNFRPESFLGEGGFGHVYKGQLESTGQVVAVKQLDRNGLQGNREFLVEVLMLSLLHHHNLVNLIGYC
ADGDQRLLVYEFMPLGSLEDHLHDLPPGKEPLDWNTRMKIAAGAAKGLEYLHDKANPPVIYRDFKSSNILLDEG
FHPKLSDFGLAKLGPVGDKSHVSTRVMGTYGYCAPEYAMTGQLTVKSDVYSFGVVFLELITGRKAIDSTRPHGEQ
NLITWARPLFTNRRKLSKLADPLLQGRFPMRGLYQALAVASMCIQEEAAARPHIGDVVTALSYLANQAYEPNAS
GHGHSGSGETDEKRYRDDRGGRVSKNDEGGVSGCRWDLEGSEKDDSPKESARMLNRDLDRERAVAEAKMW
GENWREKRRQSAQGSSDGSNG

>A663|A0A5N5FD54

MGCFPCFDSKEEEKLNNPAAEIDDRKQGQPTVSNNISRLPSGVDRLRSRSNGGSRRELPKLPDPKDVVPGGQIA
AQTFTFRELATATKNFRPESFIGEGGFGRVYKGRLESTGQVVAVKQLDRNGLQGNREFLVEVLMLSLLHHPNLV
NLIGYCADGDQRLLVYEFMPLGSLEDHLHDLPPDKEPLDWNTRMKIASGAANGLEYLHDKANPPVIYRDFKSSNI
LLEEGFHPKLSDFGLAKLGPTGDKSHVSTRVMGTYGYCAPEYAMTGQLTVKSDVYSFGVVFLELITGRKAIDSDR
PHGEQNLITWARPLFNDRRKFAKLADPRLQGRYPMRGLYQALAVASMCIQEQAATRPLIGDVVTALSYLANQS
YDPNLASGHGHRGSGEKEEKRHRDGGRILKNEEGGGSGRRWDLDGSEKDDSPKETARMLDRERAVAEAKMW
GENWREKRRLSAQGSFDGTNL

>A664|A0A5N5P0T5

MGCFPCFDSREEEKLNREKQSDDLKHTLPAVPSNISKLSSGSDRLRPRSDGGQSKRQLPSPKDAPGVNIAAQIFA
FRELAAATKNFRPECFIGEGGFGRVYKGCLESTGQVVAVKQLDRNGLQGNREFLVEVLMLSLLHHSNLVNLIGYC
ADGDQRLLVYEFMPLGSLEDHLHDLPPEKEPLEWNTRMKIAAGAARGLEYLHDKASPPVIYRDFKSSNILLEEGF
HPKLSDFGLAKLGPTGDKSHVSTRVMGTYGYCAPEYAMTGQLTVKSDVYSFGVVFLELITGRKAIDSTQPHGEQ
NLVAWARPLFNDRRRFSKLADPLLQGRYPMRGLYQALAVASMCIQEQAAARPLIGDVVTALSYLANQAYEPNG
GHGHRGSGDRDEKRQRCDRGGQLSRNEEGGGSGRRWDLDGSEKEESPRETAKMSNRDLDRERAVAEAKMW
GENWREKQRQSAQESFDGSNG

>A665|LOC103929032

MGCFPCFDSKEEEKLNNPAAEIDDRKQGQPTVSNNISRLPSGVDRLRSRSNGGSRRELPKLPDPKDVVPGGQIA
AQTFTFRELATATKNFRPESFIGEGGFGRVYKGRLESTGQVVAVKQLDRNGLQGNREFLVEVLMLSLLHHPNLV
NLIGYCADGDQRLLVYEFMPLGSLEDHLHDLPPDKEPLDWNTRMKIASGAANGLEYLHDKANPPVIYRDFKSSNI
LLEEGFHPKLSDFGLAKLGPTGDKSHVSTRVMGTYGYCAPEYAMTGQLTVKSDVYSFGVVFLELITGRKAIDGDR
PHGEQNLITWARPLFNDRRKFAKLADPRLQGRYPMRGLYQALAVASMCIQEQAATRPLIGDVVTALSYLANQS
YDPNLASGHGHRGSGEKDEKRHRDGGRILKNEEGGGSGRRWDLDGSEKDDSPKETARMLDRERAVAEAKMW
GENWREKRRLSAQGSFDGTNL

>A666|LOC110810800

MGCFSCFDSKEEEKLNTQKETEDRKESQATVPNNISKLSSGGERLRSRSNGGSKRELPGPRDGLGVNIAAQTFTF
RELAAATKNFRPESFLGEGGFGRVYKGNLDSTGQVVAIKQLDRNGLQGNREFLVEVLMLSLLHHPNLVNLIGYC
ADGDQRLLVYEFMPFGSLEDHLHDLPPDKEPLDWNTRMKIAAGAARGLEYLHDKANPPVIYRDFKSSNILLDEG
FHPKLSDFGLAKLGPVGDKSHVSTRVMGTYGYCAPEYAMTGQLTVKSDVYSFGVVFLELITGRKAIDSTLPHGEQ
NLVTWARPLFNDRRKFSKLADPKLQGRYPMRGLYQALAVASMCIQEQAATRPLIGDVVTALSYLANQAYDPNA
AGYGYRGSGDKDDRRTRDEKGGRISKNEEGTGSGRRWDLEGSEKEDSPRETARMLNRDLDRKRAVEEARMW
GENLREKRRQNAQGSFDGNTE

>A667|LOC18608901

MGCFPCFDSSEEEKLTPVKETDDRKQGQPTVSSNISRLSSGGDRLRSRSNGGSKRELPSPRDGPGVQIAAQIFTFR
ELAAATKNFRPESFLGEGGFGRVYKGRLESTGQVVAVKQLDRNGLQGNREFLVEVLMLSLLHHPNLVNLIGYCA
DGDQRLLVYEFMPLGSLEDHLHDLPPEKEPLDWNTRMKIAAGAAKGLEYLHDKANPPVIYRDFKSSNILLDEGFH
PKLSDFGLAKLGPVGDKSHVSTRVMGTYGYCAPEYAMTGQLTVKSDVYSFGVVFLELITGRKAIDSTRPHGEQNL
VTWARPLFNDRRKFSKLADPRLQGRYPMRGLYQALAVASMCIQEQAATRPLIGDVVTALSYLANQAYDRNAVG
HGYRGPGDKDDKRYRDDRGGRVSRNDEGGASGRRWDLEGSEKEDSPRETARMLNRDLDRERAVAEAKMWG
ENWREKRRQSAQGSFDGSNG

>A668|XP_008222220.1

MGCFPCFDSKEEEKLNNPVNDIDDRKQGQPTVSSNISRLPSGADRMRSRSNGGSKRDLGSKLPDLKDVPGVQIA
AQIFTFRELVTATKNFRPESFIGEGGFGRVYKGRLESSGQVVAVKQLDRNGLQGNREFLVEVLMLSLLHHPNLVN
LIGYCADGDQRLLVYEFMPLGSLDDHLHDLPPDREPLDWNTRMKIASGAAKGLEYLHDKANPPVIYRDFKSSNIL
LEEGFHPKLSDFGLAKLGPTEDKSHVSTRVMGTYGYCAPEYAMTGQLTVKSDVYSFGVVFLELITGRKSIDSNRP
HGEQNLITWARPLFNDRRKFSKLADPRLQGRYPMRGLYQALAVASMCIQEQAATRPLIGDVVTALSYLANQSY
DPNMASGHGHRGSGEKDERRHRDERGGRILKNEEGGGSGRRWDLDGSEKDDSPKETARMLNRDLDRERAVA
EAKMWGENWREKRRQSAQGS

>A669|XP_030960494.1

MGCFSCFDSKEEEKLNTQKPSDDRKQALPMVSSNISRMPSGADKLVSRANGGTKKELPSPKDGPVPGVHIAAQ
TFTFRELAAATKNFRPECFLGEGGFGRVYKGRLESTGQIVAVKQLDRNGLQGNREFLVEVLMLSLLHHPNLVNLI
GYCADGDQRLLVYDFMPLGSLEDHLHDLPPDKEPLDWNTRMKIAAGAAKGLEYLHDKANPPVIYRDFKSSNILL
DEGFHPMLSDFGLAKLGPVGDKSHVSTRVMGTYGYCAPEYAMTGQLTVKSDVYSFGVVFLELITGRKAIDSTRP
HGEQNLVTWARPLFNDRRKFSKLADPQLQGRYPMRGLYQALAVASMCIQEQAATRPLIGDVVTALSYLANQAY

DPNTGHGHRGSGDKDEKRNRDERVGRILKNEEGGGSGRRWDFEGSEKDDSPRETARMLNRDLDRERAVAEA
KMWGENWREKRRQNAQGSFDGNN

>A670|108199807

MGCFPCFDSKEEESLNPQKHSNHHKETHTHPALSNNISRLASGADRFKSRSNVGGKREQPGLKELADAQIAAQT
FTFRELAAATNNFRPESFIGEGGFGRVYKGRLQTTGQVVAVKQLDRDGLQGNREFLVEVLMLSLLHHSNLVNLIG
YCADGDQRLLVYEFMPLGSLEDHLHDLPPDKEPLDWNTRMKIAAGAAKGLEFLHDKASPPVIYRDFKSSNILLEE
EFHPKLSDFGLAKLGPTGDKSHVSTRVMGTYGYCAPEYAMTGQLTVKSDVYSFGVVFLELITGRKAIDSTQPQG
QQNLVTWARPLFNDRRRFAKLADPRLQGQYPMRGLYQALAVASMCIQEQAAARPLIGDVVTALSYLANQAYD
SSSVPGHGYRLNGDRDEKNNRGSGRFLRNDMGGGSGLKWDLEGSEKDDSPKETARTLNRDLERERAVAEAKL
WGENLREKRRQEDNFDTNNE

>A671|A0A498HQE1

MGCFPCFDSKEEEKLNNPAAEIDNRKQVQPTVSNNISRLPSGVDRLRSRSNGGSRGELPKLPEPKDVVPGGQIA
AQTFTFRELATATKNFRPESFIGEGGFGRVYKGQLESTGQVVAVKQLDRNGLQGNREFLVEVLMLSLLHHPNLV
NLIGYCADGDQRLLVYEFMPLGSLEDHLHDLPPDKEPLDWNTRMKIASGAAKGLEYLHDEANPPVIYRDFKSSNI
LLEEEFHPKLSDFGLAKLGPTGDKSHVSTRVMGTYGYCAPEYAMTGQLTVKSDVYSFGVVFLELITGRKAIDSDRP
HGEQNLITWARPLFNDRRKFSKLADPRLQGRYPMRGLYQALAVASMCIQEQAATRPLIGDVVTALSYLANHSYD
PNSASGNGHRGSGEKNEKRHRDGGRILKNEEGGGSGRSWGLDGSEKDDSPKETARMLDRERAVAEAKMWGE
NWREKRRQSAQGSFDGTS

>A672|A0A540N1X6

MGCFPCFDSKEEEKLNNPAAEIDDRKQVQPTVSNNISRLPSGVDRLRSRSNGGSKGELPKLPEPKDVVPGGQIA
AQTFTFRELATATKNFRPESFIGEGGFGRVYKGQLESTGQVVAVKQLDRNGLQGNREFLVEVLMLSLLHHPNLV
NLIGYCADGDQRLLVYEFMPLGSLEDHLHDLPPDKEPLDWNTRMKIASGAAKGLEYLHDKANPPVIYRDFKSSNI
LLEEEFHPKLSDFGLAKLGPTGDKSHVSTRVMGTYGYCAPEYAMTGQLTVKSDVYSFGVVFLELITGRKAIDSDRP
HGEQNLITWARPLFNDRRKFSKLADPRLQGRYPMRGLYQALAVASMCIQEQAATRPLIGDVVTALSYLANHSYD
PNSASGNGHRGSGEKNEKRHRDGGRILKNEEGGGSGRSWGLDGSEKDDSPKETARMLDRERAVAEAKMWGE
NWREKRRQSAQGSFDGTS

>A673|B9GMP8

MGCFPCFDSREEEDLNREKQSDDLKQTLPTVPSNISKLSSGSDRLRPRSNGGQSKRQFPSPKDAPGVNIAAQIFA
FRELAAATKNFMPECFLGEGGFGRVYKGCLESTGQVVAVKQLDRNGLQGNREFLVEVLMLSLLHHPNLVNLIGY
CADGDQRLLVYEFMPLGSLEDHLHDLPPEKEPLDWNTRMKIAAGAAKGLEYLHDKASPPVIYRDFKSSNILLEEG
FNPKLSDFGLAKLGPTGDKSHVSTRVMGTYGYCAPEYAMTGQLTVKSDVYSFGVVFLELITGRKAIDSTQPHGQ
QNLVAWARPLFNDRRKFSKLADPRLQGRYPMRGLYQALAVASMCIQEQAAARPLIGDVVTALSYLANQAYEPN
GHGHRGSGDRDEKRQRDERGGQLSRNEEGGGSGRRWDLDGSEKEDSPRETAKMLNRDLDRERAVAEAKMW
GENWREKQRQNAQGSFDGSNG

>A674|KAB2043520.1

MGCFSCFDSKEEEKLNAVNETNDPKRPQPIVSSNISRLSSGGDRLRSRSNGGSKREIPSLRDGPGVQIAAQTFSFR
ELAAATKNFRPESFLGEGGFGRVYKGRLESTGQVVAVKQLDRNGLQGNREFLVEVLMLSLLHHPNLVNLIGYCA
DGDQRLLVYEFMPLGSLEDHLHDLPPSKEPLDWNTRMKIAAGAAKGLEYLHDKANPPVIYRDFKSSNILLEEGFH
PKLSDFGLAKLGPVGDKSHVSTRVMGTYGYCAPEYAMTGQLTVKSDVYSFGVVFLELITGRKAIDSSQPHGEQN

LITWARPLFNNRRKLSKLADPLLQGRFPMRGLYQALAVASMCIQEEAATRPLIGDVVTALSYLANQAYDPNTTG
NGHRGSGETDEKRYRDDRGGRVSKNDEGGVSGRKWDLEGSEKEDSPRETARMLNRDLDRERAVAEAKMWG
ENWREKRRQSAQGSSDGSN

>A675|TYH86183.1

MGCFPCFDSKEEEKLNTVNESNDPKQAQPTVSSNISSLSSGGDRLRSRSNGGSKRELPCPRDGPGVQIAAHIFSF
RELAAATKNFRPESFLGEGGFGHVYKGQLESTGQVVAVKQLDRNGLQGNREFLVEVLMLSLLHHPNLVNLIGYC
ADGDQRLLVYEFMPLGSLEDHLHDLPPGKEPLDWNTRMKIAAGAAKGLEYLHDKANPPVIYRDFKSSNILLEEGF
HPKLSDFGLAKLGPVGDKSHVSTRVMGTYGYCAPEYAMTGQLTVKSDVYSFGVVFLELITGRKAIDSTRPHGEQ
NLITWARPLFTNRRKLSKLADPLLQGRFPMRGLYQALAVASMCIQEEAAARPHIGDVVTALSYLANQAYDPNAS
GHGHSGSGETDEKRYRDERGGRVSKNDEGGVSGCRWDLEGSEKDDSPKETARMLNRDLDRERAVAEAKMW
GENWREKRRQSAQGSSDGSN

>A676|KAE8686889.1

MGCFPCFDSKEEEKLNPVKETVDRKQGHPTVSNNISKLSSGGDRLRARSNGGSKREIPSPKDGPGGQIAAQIFTF
RELAAATKNFRPESFLGEGGFGHVYKGRLESTGQVVAVKQLDRNGLQGNREFLVEVLMLSLLHHPNLVNLIGYC
ADGDQRLLVYEFMPLGSLEDHLLDLPLEKEPLDWNTRMKIAAGAAKGLEYLHDKANPPVIYRDFKSSNILLDEGF
QPKLSDFGLAKLGPTGDKSHVSTRVMGTYGYCAPEYAMTGQLTVKSDVYSFGVVFLELITGRRAIDSTRPHGEQ
NLVTWARPLFNDRRKFSKLADPRLQGRYPMRGLYQALAVASMCIQEEAATRPLIGDVVTALSYLANQAYDPNS
AGHGYRGSGDKDDKRYKDDRGGRVSKNDEGASSGRRCDLEGSEKEDSPRETARMLNRDLDRERAVAEAKMW
GENWREKRRQNAQGSSDGSN

>A677|K7UWE8

MGCLPCFGSAGEGAAKEGGARKDDKSKPQGGLDSKKDAVILREGNNQHIAAHTFTFRELAAATKNFRQDCLLG
EGGFGRVYKGRLENGQVVAVKQLDRNGLQGNREFLVEVLMLSLLHHDNLVNLIGYCADGDQRLLVYEFMPLGS
LEDHLHDIPPDKEPLDWNTRMKIAAGAAKGLEYLHDKASPPVIYRDFKSSNILLGEGFHPKLSDFGLAKLGPVGD
KTHVSTRVMGTYGYCAPEYAMTGQLTAKSDVYSFGVVFLELITGRKAIDNTKPHGEQNLVAWARPLFKDRRKFP
KMADPSLQGCFPMRGLYQALAVAAMCLQEQAATRPFIGDVVTALSYLASHTYDPNAPAQHNRSNSSTPRVSR
GGGSNDQRRLRSSNHHSPDLRREVTTASRYDDEVSRANSGTGSGRRSGLDDADMSGSQLGSPAHTGRKRGSP
RTSESQHAIAEAKTYGENSRGRK

>A678|LOC103403202

MGCFPCFDSKEEEKLNNPAAEIDNRKQVQPTVSNNISRLPSGVDRLRSRSNGGSRGELPKLPEPKDVVPGGQIA
AQTFTFRELATATKNFRPESFIGEGGFGRVYKGQLESTGQVVAVKQLDRNGLQGNREFLVEVLMLSLLHHPNLV
NLIGYCADGDQRLLVYEFMPLGSLEDHLHDLPPDKEPLDWNTRMKIASGAAKGLEYLHDZANPPVIYRDFKSSNI
LLEEEFHPKLSDFGLAKLGPTGDKSHVSTRVMGTYGYCAPEYAMTGQLTVKSDVYSFGVVFLELITGRKAIDSDRP
HGEQNLITWARPLFNDRRKFSKLADPRLQGRYPMRGLYQALAVASMCIQEQAATRPLIGDVVTALSYLANHSYD
PNSASGNGHRGSGEKNEKRHRDGGRILKNEEGGGSGRSWGLDGSEKDESPKETARMLDRERAVAEAKMWGE
NWREKRRQSAQGSFDGTS

>A679|LOC103941019

MGCFPCFDSKEEEKLNNPAAEIDDRKQVQPTVSNNISRLPSGVDRLRSRSNGGSRGELPKLPEPKDVVPGGQIA
AQTFTFRELATATKNFRPESFIGEGGFGRVYKGRLESTGQVVAVKQLDRNGLQGNREFLVEVLMLSLLHHPNLV
NLIGYCADGDQRLLVYEFMPLGSLEDHLHDLPPDKEPLDWNTRMKIASGAAKGLEYLHDKANPPVIYRDFKSSNI

LLEEEFHPKLSDFGLAKLGPTGDKSHVSTRVMGTYGYCAPEYAMTGQLTVKSDVYSFGVVFLELITGRKAIDSDRP
HGEQNLITWARPLFNDRRKFSKLADPRLQGRYPMRGLYQALAVASMCIQEQAATRPLIGDVVTALSYLANHSYD
PNSASGNGHRGSGEKNEKRHRDGGRILKNEEGGGSGRSWGLDGSEKDDSPKETARMLDRERAVAEAKMWGE
NWREKRRQSAQGSFDGTN

>A680|LOC105123056

MGCFSCFDSREEEKLNQEKKSVDLKPTQPPVSSNISKLSSGADRLRSRSNDGQSKRELTSPKDAPGVNIAAHIFTF
RELAAATKNFRPECFLGEGGFGRVYKGRLESTGQVVAVKQLDRNGLQGNREFLVEVLMLSLLHHPNLVNLIGYC
ADGDQRLLVYEFMPLGSLEDHLHDLPPEKEPLDWNTRMKIAAGAAKGLEYLHDEASPPVIYRDFKSSNILLEEGF
HPKLSDFGLAKLGPTGDKSHVSTRVMGTYGYCAPEYAMTGQLTVKSDVYSFGVVFLELITGRKAIDSTRPHGEQ
NLVTWTRPLFNDRRKLSKLADPRLQGRYPMRGLYQALAVASMCIQEQAAARPLIGDVVTALSYLANQAYEPNG
HGYRGLGDRDEKRRREERGGQLSRNEEGGGSGRKWDLDGSEKEDSPRETARMLNRDLDRERAVAEAKMWGE
NWREKRRQSAQGSFDGSNE

>A681|PraPBS1XP_028803729.1

MGCFSCFDSKEDEQLNPRQEIDDLKQGHPAASSHISRLPSGADKLRSRSSNGGSKRELPAPKDGPGVQIAAQTFT
FRELAAATKNFRPESFIGEGGFGRVYKGRLESTGQVVAVKQLDRNGLQGNREFLVEVLMLSLLHHPNLVNLIGYC
ADGDQRLLVYEFMPLGSLEDHLHDLPPDKEPLDWNTRMKIAAGAAKGLEYLHDKANPPVIYRDFKSSNILLDEG
FHPKLSDFGLAKLGPVGDKSHVSTRVMGTYGYCAPEYAMTGQLTVKSDVYSFGVVFLELITGRKAIDSTRPHGEQ
NLVTWARPLFNDRRKFSKLADPRLQGRYPMRGLYQALAVASMCIQEQAATRPLIGDVVTALSYLANQAYDPNA
SGHGHRGLGDDKRSRDERSGRISRNEEGGGSGRRWDLEGSEKEDSPRETARMLNRDLDRERAVAEAKMWGE
NWREKRRQNGQANFDASNA

>A682|SapurV1A.0453s0170.1

MGCFPCFDSREEEKLNREKQSDDLKHTLPAVPSNISKLSSGSDRLRPRSDGGQSKRQLPSPKDAPGVNIAAQIFA
FRELAAATKNFRPECFIGEGGFGRVYKGCLESTGQVVAVKQLDRNGLQGNREFLVEVLMLSLLHHSNLVNLIGYC
ADGDQRLLVYEFMPLGSLEDHLHDLPPEKEPLDWNTRMKIAAGAARGLEYLHDKASPPVIYRDFKSSNILLEEGF
HPKLSDFGLAKLGPTGDKSHVSTRVMGTYGYCAPEYAMTGQLTVKSDVYSFGVVFLELITGRKAIDSTQPHGEQ
NLVAWARPLFNDRRRFSKLADPLLQGRYPMRGLYQALAVASMCIQEQAAARPLIGDVVTALSYLANQAYEPNG
HGHRGSGDRDEKRQRDDRGGQLSRNEEGGGSGRRWDLDGSEKEESPRETAKMSNRDLDRERAVAEAKMWG
ENWREKQRQSAQESFDGSNG

>A683|LOC111017471

MGCFPCFDSREEEKLNPEKESDDGKQDHPMVPPNISKFPSGIEKLRSKSNGGSKREQQSPKDTPLVNISAQTFTF
RELAIATKNFKPECFIGEGGFGRVYKGRLESTGQIVAVKQLDRNGLQGNREFLVEVLMLSLLHHSNLVNLIGYCAD
GDQRLLVYEFMPLGSLEDHLHEIPLDKEPLDWNTRMKIAAGAARGLEYLHDKANPPVIYRDFKSSNILLDEGYHP
KLSDFGLAKLGPVGDKSHVSTRVMGTYGYCAPEYAMTGQLTVKSDVYSFGVVFLELITGRRAIDSTRPQGEQNL
VTWARPFFNDRRRFSKLADPQLQGRYPMRGLYQALAVASMCTQEQAAARPLIGDVVTALSYLANQSYDPSSPS
GPSAQRGSGDRDERRNRDEKGGGEEGGASGRRWALDGPDKDDSPRETVRILNRDLDRERAVAEAKMWGEN
WREKRRQSAQGSFDGSNG

>A684|A0A059B7A3

MGCFPCFDSREEETLNPGKGSDDPKHGVPNVSSKISSLPSGVERLRSRSNLGLRRDLIGPKDVPGVHIAAQTFTFR
ELAAATKNFKPECFLGEGGFGGVYKGKLETTGQVVAVKQLDRNGLQGNREFLVEVLMLSLLHHPNLMNLIGYCA

DGDQRLLVYEFMAFGSLEDHLFDLPLGKEPLDWNTRMTIAAGAAKGLEYLHDKANPPVIYRDFKSSNILLDEKFH
PKLSDFGLAKLGPVGDKSHVSTRVMGTYGYCAPEYAMTGQLTVKSDVYSFGVVFLELITGRKAIDSSRPHGEQNL
VTWARPLFNERRKFSKLVDPSLQGRYPMRGLYQALAVASMCIQEQAAARPLIGDVVTALSYLANQAYDPNVAS
NFGNKGSSVDERGSKILRNEDAGGSGRSRWEFEGSEKDNSPKDGKLLNRDLNRERAVAEAKMWGENWREKR
RQSAQGSFDGSNGRLC

>A685|A0A445IUY2

MGCFSCFDSREDEMLNPNPQQENHHHEHEHDHDLKPPVPSRISRLPPSASAGDKLRSTTSNGESKRELAAAVQI
AAQIFTFRELAAATKNFMPQSFLGEGGFGRVYKGLLETTGQVVAVKQLDRDGLQGNREFLVEVLMLSLLHHPNL
VNLIGYCADGDQRLLVYEFMPLGSLEDHLHDLPPDKEPLDWNTRMKIAAGAAKGLEYLHDKANPPVIYRDFKSS
NILLDEGYHPKLSDFGLAKLGPVGDKSHVSTRVMGTYGYCAPEYAMTGQLTVKSDVYSFGVVFLELITGRKAIDST
RPHGEQNLVTWARPLFNDRRKFPKLADPQLQGRYPMRGLYQALAVASMCIQEQAAARPLIGDVVTALSFLAN
QAYDHRGGAGDDKRNRVLKNGEGGGGGSGGRWDLEGSEKDDSPRETARMLNSNNRDLDRERAVAEAKMW
GENWREKRRQSAQGSFDGSNA

>A686|LOC109808033

MGCFSCFDSREDEKLNPNPQQETNHHDHELKPPVPSRISRLPSASGAEKLRSTSNGDSNGELAALKDGPAVQIA
AQTFTFRELAAATKNFRPQSFLGEGGFGRVYKGRLETTGQVVAVKQLDRNGLQGNREFLVEVLMLSLLHHPNLV
NLIGYCADGDQRLLVYEFMPMGSLEDHLHDLPPDKEPLDWNTRMKIAAGAAKGLEYLHDKANPPVIYRDFKSS
NILLDEGYHPKLSDFGLAKLGPVGDKSHVSTRVMGTYGYCAPEYAMTGQLTVKSDVYSFGVVFLELITGRKAIDST
RPHGEQNLVTWARPLFNDRRKFPKLADPQLQGRYPMRGLYQALAVASMCIQEQAAARPLIGDVVTALSFLAN
QAFDHRGAGEDKRNRDDKGGRILKNDEGGGSGRRWDLEGSEKDESPRETARMLNNMHLDRERAVAEAKM
WGENWREKRRQSAQGSFDGSNA

>A687|LOC8074484

MAFLACLCRCPQDEDDDDKEGEQFRINRQVASVDYSKSPESCPLKTERAVHMEGAQLIGRHDEATIFTLRQLAE
ATKNFSEDYLLGRGGFGCVYKATLSNGQVVAVKQLDLNGFQGNREFLVEVLMLNLLHHPNLVNLHGYCVDGD
QRLLVYEYMPLGSLEDHLHDLAPDQEPLDWRTRMKIAAGAAAGLEYLHDKANPPVIYRDIKPSNILLGEGYHAKL
SDFGLAKLGPVGDKTHVTTRVMGTHGYCAPEYALTGQLTVKSDIYSFGVVFLELITGRRPQDSDRPPEEQDLVA
WARPLFKDQKKFPKMADPLLQGHFPRRGLYQALAIAAMCLQEKAKNRPPIREVAAALSYLASQTYDRNNTAPR
RNRAGASTSRVLDDQIGQDTTLANQQGAQMSMHGQTNHVMPEVKETSWSGNGSHRGGRGRVTPNGTDRE
RALADANVWAEAWRRQEKASKMR

>A688|LOC8078517

MSCLPCSGSSGKEAKSLAALSPTPRPAAKAAPVRSNSRASGSKKEDSVPVRRGGNIPHGPAQIFTFRELAIATKNF
RKDCLLGEGGFGRVYKGRMENGQVIAVKQLDRNGFQGNREFLVEVLMLSLLHHPNLVRLIGYCADGDQRLLVY
EYMLLGSLENHLFGPPDKEPLDWNTRMKIAAGAAKGLEYLHDKANPPVIYRDFKSSNILLGEDYYPKLSDFGLAKL
GPVGDKTHVSTRVMGTYGYCAPEYAMTGQLTLKSDVYSFGVVFLELITGRKAIDHTQPSGEQNLVAWARPLFR
DRRKFCQLADPSLQGRYPKRGLYQALAVAAMCLQEQAASRPLIGDVVTALSYLAAHPYDPNVPSTKDSRTCPST
PRAKTHRRTTSVPDAQHAAESLMLNFPDLRKETVRGGKFEKDGTEGSGSSSSSGRNDGLDVPQLLAGKACSEG
DNVQKSTIKVGAREN

>A689|A0A2U1NQY5

MGCFSCFDSPGDEKPISQKVGADRPEVHPTSAPSNISRLPSGVDRLKSRNNVSLRRESSGPKDNNNPYSQIAAHT
FTLRELAAATNNFSPDCFLGEGGFGPVYRGRLPGTGQLVAVKQLDRNGLQGNREFLVEVLMLSLLHHPNLVNLE
GYCADGEQRLLVYEFMPLGSLEDHLHDLPPESEALDWNTRMKIAAGAARGLEFLHDKANPPVIYRDFKSSNILLG
EGFQPKLSDFGLAKLGPTGDKSHVSTRVMGTYGYCAPEYAMTGQLTVKSDVYSFGVVFLELITGRKAIDSTAPQG
QQNLVTWARPLFNDRRKFASLADPRLEGHYPIRGLYQALAVASMCIQEQAAARPLIGDVVTALSYLANHAYDPN
AAAGQNSRHINGDKNGRILKNEEGGGSGRSRWDLEGSDKGDSPRENQTPRILNRDLDRERAVAEAKMWVEKR
RQSAQGSFDGNNNG

>A690|A0A3L6RP49

MGCFSCFDSPADEQLNPKLGGAGGYGGASSAAAAYGGGSGGGRHGDRGYPDLQQAPMAAPRVEKLSAAAEK
ARVKSNALAREASAPKDANGNVITAQTFTFRELATATRNFRPECFLGEGGFGRVYRGRLESTGQVVAIKQLNRD
GLQGNREFLVEVLMLSLLHHQNLVNLIGYCADGDQRLLVYEYMPFGSLEDHLLDLPLDKEALDWNTRMKIAAG
AAKGLEYLHDKANPPVIYRDFKSSNILLDESFHPKLSDFGLAKLGPVGDKSHVSTRVMGTYGYCAPEYAMTGQLT
VKSDVYSFGVVLLELITGRRAIDSTRPHGEQNLVSWARPLFNDRRKLPKMADPRLEGRYPMRGLYQALAVASMC
IQSEAASRPLIADVVTALSYLASQSYDPNAALASRKPSGEQRSKPGENGRVVSRNDETSSSGHKSPGKDREDSPR
DLPENFVRMKGHFEFQL

>A691|A0A5N6NSE8

MGCFCCFDSREEQKSNHLKVRADRPEVHPSAPSNISRLPSGEDRVKSRNNVNVSLRKESSGSKDGSQGQIAAHT
FTFRELAAATNNFSPDCFLGEGGFGHVYRGRLQSSGQIVAVKQLDRNGLQGNREFLVEVLMLSLLHHPNLVNLI
GYCADGDQRLLVYEFMPLGSLEDHLHDLPPEKEALDWNTRMKIAAGAARGLEFLHDKANPPVIYRDFKSSNILL
GEGFQPKLSDFGLAKLGPTGDKSHVSTRVMGTYGYCAPEYAMTGQLTVKSDVYSFGVVFLELITGRKAIDSTAPQ
GQQNLVTWARPLFNDRRKFATLADPRLEGRYPMRGLYQALAVASMCIQEQAAARPLIGDVVTALSYLANHNY
DPNVAPNQSTRYNNGDRNNRISKNEEGGGSGRSRWVDLEGSDKGDSPRETQTPKMLNRDLDRERAVAEAKM
WVEKRRQSAQGSFDGNTNG

>A692|XP_027353340.1

MGCFSCFSSSREDHNLNPRHRQPNPPIPSQISRLPSGAEKLRSRSNGGSKRELPAPKDGPAVQIAAQTFTFRELA
AATKNFRPDSFVGEGGFGRVYKGRLESTGQIVAVKQLDKNGLQGNREFLVEVLMLSLLHHPNLVNLIGYCADGD
QRLLVYEFMPLGSLEDHLHYLPPDKEPLDWNTRMKIAAGAAKGLEYLHDKANPPVIYRDFKSSNILLDEGYHPKL
SDFGLAKLGPVGDKSHVSTRVMGTYGYCAPEYAMTGQLTVKSDVYSFGVVFLELITGRKAIDSSQPQGEQNLVT
WARPLFNDRKKFSKLADPNMQGRFPMRGLYQALAVASMCIQESAATRPLIGDVVTALSYLANQAYDPSNAGH
GHRGSSDDKRNRDDKGGRILKNDEAGGSGRRWDLEGSEKDDSPRETARILNRDLDRERAVAEAKMWGENLR
QKRKQQSVQQANLDDSST

>A693|C6ZRR7

MGCFSCFDSREDEMLNPNPQQENHHHEHEHDHDLKPPVPSRISRLPPSASGDKLRSTTSNGESKRELAAAVQIA
AQIFTFRELAAATKNFMPQSFLGEGGFGRVYKGLLETTGQVVAVKQLDRDGLQGNREFLVEVLMLSLLHHPNLV
NLIGYCADGDQRLLVYEFMPLGSLEDHLHDLPPDKEPLDWNTRMKIAAGAAKGLEYLHDKANPPVIYRDFKSSNI
LLDEGYHPKLSDFGLAKLGPVGDKSHVSTRVMGTYGYCAPEYAMTGQLTVKSDVYSFGVVFLELITGRKAIDSTR
PHGEQNLVTWARPLFNDRRKFPKLADPQLQGRYPMRGLYQALAVASMCIQEQAAARPLIGDVVTALSFLANQ
AYDHRGGTGDDKRNRVLKNGEGGGGGSGGRWDLEGSEKDDSPRETARMLNSNNRDLDRERAVAEAKMWG
ENWREKRRQSAQGSFDGSNA

>A694|AE8660883.1

MGCFPCFDSKEEEKLNPVNETVDIKQGHPTVSSNISKLSSGGDRLRARSNGWSKREIPSPKDGPGVQIAAQIFTF
RELAAATKNFRPESFLGEGGFGHVYKGQLESTGQVVAVKQLDRNGLQGNREFLVEVLMLSLLHHPNLVNLIGYC
ADGDQRLLVYEFMPLGSLEDHLHDLPPEKEPLDWNTRMKIAAGAAKGLEYLHDKANPPVIYRDFKSSNILLEEGF
QPKLSDFGLAKLGPTGDKSHVSTRVMGTYGYCAPEYAMTGQLTVKSDVYSFGVVFLELITGRKAIDSARPHGEQ
NLVTWARPLFNDRRKFAKLADPRLQGRYPMRGLYQALAVASMCIQEEAATRPLIGDVVTALSYLANQAYDPNA
AGHGYRGSGDKDDKRQRDERGGRVSKNDEGASSGRRWDLEGSEKEDSPRETARMLNRDLERERAVAEAKM
WGENLREKRRQNARGS

>A695|XP_021291854.1

MGCFPCFDSREEEKLNPVKETDDRKQGQPTVSSNISRLASGGDRLRSRSNGGSKRELPSPRDGPGVQIAAQIFTF
RELAAATKNFRPESFLGEGGFGRVYKGQLESTGQVVAVKQLDRNGLQGNREFLVEVLMLSLLHHPNLVNLIGYC
ADGDQRLLVYEFMPLGSLEDHLHDLPPEKEPLDWNTRMKIAAGAAKGLEYLHDKANPPVIYRDFKSSNILLDEGF
HPKLSDFGLAKLGPVGDKSHVSTRVMGTYGYCAPEYAMTGQLTVKSDVYSFGVVFLELITGRKAIDSTQPHGEQ
NLVTWARPLFNDRRKFSKLADPRLQGRYPMRGLYQALAVASMCIQEQAATRPLIGDVVTALSYLANQAYDPNA
AGHGYRGPGDKDDKRYRDDRGGRVSRNDEGGASGRRWDLEGSEKEDSPRETARMLNRDLNRERAVAEAKM
WGENWREKRRQSAQGS

>A696|KAF3432345.1

MGCFSCFDSKEEEKLNPDKESDREQSQPTVPSSLSRLPSGADRFRSRTNGASKRELPVPKDVPAVGANIAAQTFT
FRELVAATKNFRPESFIGEGGFGRVYKGRLESTGQVVAVKQLDRNGLQGNREFLVEVLMLSLLHHPNLVNLIGYC
ADGDQRLLVYEFMPLGSLEDHLHDLPPDKEPLDWNTRMKIAAGAARGLEYLHDKANPPVIYRDFKSSNILLEEGF
HPKLSDFGLAKLGPTGDKSHVSTRVMGTYGYCAPEYAMTGQLTVKSDVYSFGVVFLELITGRKAIDSTQPHGEQ
NLVTWARPLFNDRRKFSKLADPQLQGRYPMRGLYQALAVASMCIQEQAATRPLIGDVVTALSYLANQAYDPNT
SHGYRGSGDKDDKRNRDERGGRMSRNEEGGGSGRRWDLDGSEKDDSPKETARMLNRDLERERAVAEAKMW
GENWREKRRQNAQGS

>A697|ABR46086.1

MGCFSCFDSSDDDEKLNPVVESNGQKKQPQPTVSNNISGLPSGGEKLSSKTNGGSKRELLLPRDGLGQIAAHTF
AFRELAAATMNFHPDTFLGEGGFGRVYKGRLDSTGQVVAVKQLDRNGLQGNREFLVEVLMLSLLHHPNLVNLI
GYCADGDQRLLVYEFMPLGSLEDHLHDLPPDKEALDWNMRMKIAAGAAKGLEFLHDKANPPVIYRDFKSSNILL
DEGFHPKLSDFGLAKLGPTGDKSHVSTRVMGTYGYCAPEYAMTGQLTVKSDVYSFGVVFLELITGRKAIDSEMP
HGEQNLVAWARPLFNDRRKFIKLADPRLKGRFPTRALYQALAVASMCIQEQAATRPLIADVVTALSYLANQGYD
PSKDDSKRNRDERGARLITRNDDGGGSGSKFDLEGSEKEDSPRETARILNRDINRERAVAEAKMWGESLREKRR
QSEQGTSESNSTG

>A698|ABR46067.1

MGCFSCFDSSDDEKLNPVDESNHVQKKQSQPTVSNNISGLPSGGEKLSSKTNGGSKRELLLPRDGLGQIAAHTF
AFRELAAATMNFHPDTFLGEGGFGRVYKGRLDSTGQVVAVKQLDRNGLQGNREFLVEVLMLSLLHHPNLVNLI
GYCADGDQRLLVYEFMPLGSLEDHLHDLPPDKEALDWNMRMKIAAGAAKGLEFLHDKANPPVIYRDFKSSNILL
DEGFHPKLSDFGLAKLGPTGDKSHVSTRVMGTYGYCAPEYAMTGQLTVKSDVYSFGVVFLELITGRKAIDSEMP
HGEQNLVAWARPLFNDRRKFIKLADPRLKGRFPTRALYQALAVASMCIQEQAATRPLIADVVTALSYLANQAYD

PSKDDSRRNRDERGARLITRNDDGGGSGSKFDLEGSEKEDSPRETARILNRDINRERAVAEAKMWGESLREKRR
QSEQGTSESNSTG

>A699|CAA7039895.1

MGCFSCFDSSDDEKLNPVEESKGQKQFQPTVSNNISGLPSGGEKLNLKSNGGSKRELLLPRDGLSQIAAHTFAFR
ELAAATMNFHPDTFLGEGGFGRVYKGRLDSTGQVVAVKQLDRNGLQGNREFLVEVLMLSLLHHDNLVNLIGYC
ADGDQRLLVYEFMPLGSLEDHLHDLPPDKEALDWNMRMKIAAGAAKGLEFLHDKANPPVIYRDFKSSNILLDEG
FHPKLSDFGLAKLGPTGDKSHVSTRVMGTYGYCAPEYAMTGQLTVKSDVYSFGVVFLELITGRKAIDSEMPHGE
QNLVAWARPLFNDRRKFIKLADPRLKGRFPTRALYQALAVASMCIQEQAATRPLIADVVTALSYLANQAYDPSK
DESRRNRDERGGRLITAKNDEGSGGSGSKFDLDGSEKEDSPRETARMLNRDINRERAVAEAKMWGESLREKRR
QSEQGTSESNSTG

>A700|LOC104769531

MGCFSCFDSSDDEKLNPVEESNGQKKKQLQPTVSNSLSGLPSGGEKLSSKSNGGSKRELLLPRDGLNQIAAHTFA
FRELAAATMNFHPDTFLGEGGFGRVYKGRLDSTGQVVAVKQLDRNGLQGNREFLVEVLMLSLLHHPNLVNLIG
YCADGDQRLLVYEFMPLGSLEDHLHDLPPDKEALDWNMRMKIAAGAAKGLEFLHDKANPPVIYRDFKSSNILLD
EGFHPKLSDFGLAKLGPTGDKSHVSTRVMGTYGYCAPEYAMTGQLTVKSDVYSFGVVFLELITGRKAIDSEMPH
GEQNLVAWARPLFNDRRKFIKLADPKLKGRFPTRALYQALAVASMCIQEQAATRPLIADVVTALSYLANQAYDP
SKDESRRNRDERGGKLITRNDEGGGSGSKFDLEGSEKEDSPRETARILNRDINRERAVAEAKMWGESLREKRRLS
EQGTSENNSTG

>A701|XP_018446992.1

MGCFSCFDSSDDDEKLNPVDESKSQKTTQPTLSNNNISGLPSGGEKLSSKSNGGSRRDLLLPRDGLSQISAHTFAF
REVAAATMNFHPDTFLGEGGFGRVYKGRLDSTGQVVAVKQLDRNGLQGNREFLVEVLMLSLLHHPNLVNLIGY
CADGDQRLLVYEFMPLGSLEDHLHDLPPDKEALDWNMRMKIAAGAAKGLEFLHDKANPPVIYRDFKSSNILLDE
GFHPKLSDFGLAKLGPTGDKSHVSTRVMGTYGYCAPEYAMTGQLTVKSDVYSFGVVFLELITGRKAIDTDMPHG
EQNLVAWARPLFNDRRKFIKLADPRLKGRFPTRALYQALAVASMCIQEQAATRPLIADVVTALSYLANQAYDPN
KDESKRSRDERGGRLITRNDEGGGSGSKFDLEGSEKEDSPRETTRILNRDINRERAVAEAKMWGESLREKRRQSE
QGTSESNSTG

>A702|A0A0D3F5L0

MFWSTWAGAEKTRVKSNAILREPSAPKDANGNVISAQTFTFRELATATRNFRPECFLGEGGFGRVYKGRLESTG
QVVAIKQLNRDGLQGNREFLVEVLMLSLLHHQNLVNLIGYCADGDQRLLVYEYMHFGSLEDHLHDLPPDKEALD
WNTRMKIAAGAAKGLEYLHDKANPPVIYRDFKSSNILLDESFHPKLSDFGLAKLGPVGDKSHVSTRVMGTYGYC
APEYAMTGQLTVKSDVYSFGVVLLELITGRRAIDSTRPHGEQNLVSWARPLFNDRRKLPKMADPRLEGRYPMR
GLYQALAVASMCIQSEAASRPLIADVVTALSYLASQSYDPNAAHASRKPGGDQRSKVGENGRVISRNDEASSSG
HKSPNKDREDSPKEPPGILNKDFDRERMVAEAKMWGDRERMVAEAKMWGDRERMVAEAKMWGENWRD
KRRAIENGQGSLDSPTENG

>A703|A0A3N6Q066

MGCFSCFDSSDDEKLNPVDESKSQKQSQPTLSNNNISGLPSGGEKLSSKSNGGSRRELLLPRDGLSQISAHTFSFH
EVAAATMNFHPDTFLGEGGFGRVYKGRLDSTGQVVAVKQLDRNGLQGNREFLVEVLMLSLLHHPNLVNLIGYC
ADGDQRLLVYEFMPLGSLEDHLHDLPPDMEALDWNMRMKIAAGAAKGLEFLHDKANPPVIYRDFKSSNILLDE
GFHPKLSDFGLAKLGPTGDKSHVSTRVMGTYGYCAPEYAMTGQLTVKSDVYSFGVVFLELITGRKAIDTDMPHG

EQNLVAWARPLFNDRRKFIKLADPRLKGRFPTRALYQALAVASMCIQEQAATRPLIADVVTALSYLANQAYDPN
KDDSRRNRDEKGGRLITRNDEGGGSGSKFDLEGSEKEDSPRETTRILNRDINRERAVAEAKMWGESLREKRRQS
EQGTSESNSTG

>A705|Bostr.2902s0050.1

MGCFSCFDSSDDEKLNPVEESNGQKKQLQPTVSNSISGLPTGGEKLSSKSNGGSKRELLLPRDGLGQIAAHTFAF
RELAAATMNFHPDTFLGEGGFGRVYKGRLDSTGQVVAVKQLDRNGLQGNREFLVEVLMLSLLHHPNLVNLIGY
CADGDQRLLVYEFMPLGSLEDHLHDLPPDKEALDWNMRMKIAAGAAKGLEFLHDKANPPVIYRDFKSSNILLDE
GFHPKLSDFGLAKLGPTGDKSHVSTRVMGTYGYCAPEYAMTGQLTVKSDVYSFGVVFLELITGRKAIDSEMPHG
EQNLVAWARPLFNDRRKFIKLADPKLKGRFPTRALYQALAVASMCIQEQAATRPLIADVVTALSYLANQAYDPSK
DESRRNRDERGARLITRNDEGGGSGSKFDLEGSEKEDSPRETARILNRDINRERAVAEAKMWGESLREKRRQSE
QGTSESNSTG

>A706|XP_030496845.1

MGCFSCFDSKEEETLNPEKHSDDQKQAQSTPISNLESLPSGVDRLRSRSNGASKREFPVPKDGPGVPIAAQTFTF
RELAAATKNFRPECFLGEGGFGRVYKGRLESTGQVVAVKQLDRNGLQGNREFLVEVLMLSLLHHPNLVSLIGYCA
DGDQRLLVYEFMPFGSLEDHLLDLPPDKEPLDWNTRMKIAAGAAKGLEYLHDKANPPVIYRDFKSSNILLDEGFH
PKLSDFGLAKLGPTGDKSHVSTRVMGTYGYCAPEYAMTGQLTVKSDVYSFGVVFLELITGRKAIDSTRPHGEQNL
VTWARPLFNDRRKFSKLADPQLQGRYPMRGLYQALAVASMCIQEQAATRPLIGDVVTALSYLANQAYDPSTAS
GHRGSGEKEDRRNRDGGRILKNEEGGGSGRRWDLDGSEKDDSPRETARLMVNKDLDRERAVAEAKMWGEN
WREKRRQSAQGS

>A707|LOC104750036

MGCFGRTVKSNKRSETKTTKKNDVTSPKKLTNDPNIVHKNINRDNQTQPSSDCLKVSLCGDVNKEVDTKEEDQL
ALDAKDTLVEDEVSGKKAQTFTFGQLAVATGNFKSDCFLGEGGFGKVYRGFIEKINQVVAIKQLDRTGSQGIREF
VVEVLTLSLADHPNLVKLIGFCAEGVQRLLVYEYMPLGSLDNHLHDLPHSRKPLAWNTRMKIAAGAARGLEYLH
DTIKPPVIYRDLKCSNILLDKEYHAKLSDFGLAKVGPRGSETHVSTRVMGTYGYCAPDYALTGQLTFKSDVYSFGV
VLLELITGRKAIDNTRPRNKQSLVEWARPLFKNRNNFKKMVDPLMEGEYPIRGVYQALAIAAMCVQEQPSMRP
VIADVVMALDHLASSKYDHGHHHRRNHSNVTETRGDDEEKKTLPESNVTEKRGDEEEKIVVPESNVCVEEEKQE
NKICSDQAT

>A708|LOC104786849

MGCFGRTVKSNKRSEPKTTKKNDITSPKKLTNDPNIVHKNINRDNQTQPTSDCLKVSLCGDVNKEVDTKEEDQL
ALDAKDTLVEDEVSGKKAQTFTFGQLAVATGNFKSDCFLGEGGFGKVYRGFIEKINQVVAIKQLDRTGSQGIREF
VVEVLTLSLADHPNLVKLIGFCAEGVQRLLVYEYMPLGSLDNHLHDLPHSRKPLAWNTRMKIAADAARGLEYLH
DTIKPPVIYRDLKCSNILLDEEYHAKLSDFGLAKVGPRGSETHVSTRVMGTYGYCAPDYALTGQLTFKSDVYSFGV
VLLELITGRKAIDNTRPRNQQSLVEWARPLFKNRNHFKKMVDPLMEGEYPIRGVYQALAIAAMCVQEQPSMRP
VIADVVMALDHLASSKYDHGHHHRRKDSNVPETRGDDEEKKTLPESIVIEKRGDEEEQIVVPESNVCVEEEKQEIK
ICSDQAT

>A709|111396104

MGCFPCFDTREEEKLNPQKQRDGHKEVQPNIPSNIAKLSSGADRAKLRSNVGKRKEPSGPVDAPVVQIAAQTFT
FRELAAATNNFRPECFLGEGGFGRVYKGQLPNGQVVAVKQLDRNGLQGNREFLVEVLMLSLLHHPNLMNLIGY
CADGDQRLLVYEFMPLGSLEDHLLDLPPDKEPLDWNTRMKIAAGAAKGLEYLHDKANPPVIYRDFKSSNILLGEE

YSPKLSDFGLAKLGPTGDKSHVSTRVMGTYGYCAPEYAMTGQLTVKSDVYSFGVVLLELITGRKAIDGTQPHGE
QNLVAWARRLFNDRRKFTKLADPRLQGKYPMRGLSQALAVASMCTQEQAAGRPLIGDVVTALSYLANQAHDT
STAPVNSYRFSGDKDDNRNKDERGGKILRNEEGGGSGLKWDLEGSEKDDSPRETTKILNRDLDRERAVAEAKM
WGENWREKRRQSA

>A710|A0A0P0W9R3

MGVALCRLLLRRRRRPPASAAVGGRGRLRRRRASRRSPHGARGRGNATVKKELSALKDANGNVISAQTFTFRQL
AAATRNFREECFIGEGGFGRVYKGRLDGTGQIVAIKQLNRDGTQGNKEFLVEVLMLSLLHHQNLVNLVGYCADG
DQRLLVYEYMPLGSLEDHLHDLPPDKVPLDWNTRMKIAAGAAKGLEYLHDKAQPPVIYRDFKSSNILLGEDFHP
KLSDFGLAKLGPVGDKSHVSTRVMGTYGYCAPEYAMTGQLTVKSDVYSFGVVLLELITGRKAIDSTRPHVEQNLV
SWARPLFNDRRKLPKMADPGLEGRYPMRGLYQALAVASMCIQSEAASRPLIADVVTALSYLASQKYDPNTTPSS
KKAGGGEAGRALSRNDEAGSSGHKSPSSKDSPREQQLPGILNDRERMVAEAKMWGENWREKRRAAATTSSN
AQVSLDSPTETG

>A711|VVB14322.1

MGCFSCFDSSDDEKLNLVEESKGQKQSQPTISNNISGLPSGGEKPSSKNNGGSKRELLLPRDGLSQIAAHTFAFRE
LAAATMNFHPDTFLGEGGFGRVYKGRLDSTGQVVAVKQLDRNGLQGNREFLVEVLMLSLLHHPNLVNLIGYCA
DGDQRLLVYEFMPLGSLEDHLHDLPPDKEALDWNMRMKIAAGAAKGLEFLHDKANPPVIYRDFKSSNILLDEGF
HPKLSDFGLAKLGPTGDKSHVSTRVMGTYGYCAPEYAMTGQLTVKSDVYSFGVVFLELITGRKAIDSEMPHGEQ
NLVAWARPLFNDRRKFIKLADPRLKGRFPTRALYQALAVASMCIQEQAATRPLIADVVTALSYLANQGYDPNKD
DSRRNRDERGGKLITRNDEGGGSGSKFDLEGSEKEDSPRETARILNRDINRERAVAEAKMWGESLREKRRQSEQ
GTSESNSTG

>A712|A0A5D2W2B6

MGCFSCFDSKEEEKLNTVNETNDPKRPQPIVSSNISRLSSGGDRLRSRSNGGSKREIPSLRDGPGVQIAAQTFSFR
ELAAATKNFRPESFLGEGGFGRVYKGRLESTGQGNREFLVEVLMLSLLHHPNLVNLIGYCADGDQRLLVYEFMPL
GSLEDHLHDLPPSKEPLDWNTRMKIAAGAAKGLEYLHDKANPPVIYRDFKSSNILLEEGFHPKLSDFGLAKLGPV
GDKSHVSTRVMGTYGYCAPEYAMTGQLTVKSDVYSFGVVFLELITGRKAIDSSQPHGEQNLITWARPLFNNRRK
LSKLADPLLQGRFPMRGLYQALAVASMCIQEEAATRPLIGDVVTALSYLANQAYDPNTTGNGHRGSGETDEKRY
RDDRGGRVSKNDEGGVSGRKWDLEGSEKEDSPRETARMLNRDLDRERAVAEAKMWGENWREKRRQSAQGS
SDGSNG

>A713|XP_009763258.1

MGCFSCFDSKEEEKLNPQKDRVDRKDQVLLTAPSNISRLSSGADRLKTRSTNGSKREFLGLKDAPDVQIAAHTFT
FRELAAATNNFRPESFIGEGGFGRVYRGRLPSGQGVAVKQLDRNGLQGNREFLVEVLMLSLLHHPNLVNLIGYC
ADGDQRLLVYEFMPLGSLEDHLHDLPPDKEPLDWNTRMKIAAGAAKGLEYLHDKANPPVIYRDFKSSNILLEEEF
FPKLSDFGLAKLGPTGDKSHVSTRVMGTYGYCAPEYAMTGQLTVKSDVYSFGVVFLELITGRKAIDSTKPQGEQN
LVAWARPLFNDRRKFAKLADPRLQGQFPMRALYQALAVASMCIQEQAAARPLIGDVVTALSYLANQGYDDKR
NKDDRGGRIFRNDDGPGGGSGRKWDLDGGSEKDDSPKETARMLNRDLDRERAVAEAKMWGENWRDKRRQ
NGQGSFDGTN

>A714|NIOBTv3_g34227

MGCFSCFDSKEEEKLNPQRDDRKEVHLTAPSNISRLSSGADRLKTRSINGSKREFLGLKDAPDVQIAAHTFTFREL
AAATSNFRPESFIGEGGFGRVYKGRLPSGQVVAVKQLDRNGLQGNREFLVEVLMLSLLHHPNLVNLIGYCADGD

QRLLVYEFMPLGSLEDHLHDLPPDKEPLDWNTRMKIAAGAAKGLEYLHDKANPPVIYRDFKSSNILLEENFFPKLS
DFGLAKLGPTGDKSHVSTRVMGTYGYCAPEYAMTGQLTVKSDVYSFGVVFLELITGRKAIDSTMPQGEQNLVA
WARPLFNDRRKFAKLADPRLQGQFPMRGLYQALAVASMCIQEQAAARPLIGDVVTALSYLANQVDDKRNKDD
RGGRICRNEDGAGGGGSGRKWPDLDGGSEKEDSPRETARMLNRDLDRERAVAEAKMWGENWREKRRQNA
QGSFDGTNG

>A715|Kalax.0401s0019.1

MGCFPCFDSKEEEQLKPEPVKYGRRDNHLSVPSNISRLASGADRKPRNNTGSGRDLISPRDGPEGQISAHVFTFR
ELAAATKNFRPESFLGEGGFGRVYKGQLETTGQVVAVKQLDRNGLQGNREFLVEVLMLSLLHHPNLVNLIGYCA
DGDQRLLVYEFMPLGSLEDHLFDLPPEKEPLDWNTRMKIAAGAAKGLEHLHDNAKPPVIYRDFKSSNILLDEGFT
PKLSDFGLAKLGPTGDKSHVSTRVMGTYGYCAPEYAMTGQLTVKSDVYSFGVVFLELITGRRAIDGTRPHGEQN
LVTWARPIFNDRRKFGKLADPQLEGRYPVRGLYQALAVASMCIQEQAATRPLIAEVVTALSYLANQAYDPASTS
GQDRRFKEPQASKVSPRGDSRWDLDGSEKDDSPKETARMLNKDLERERAVAEAKMWGENWREKRRQHGQG
SFDANG

>A716|Niben101Scf02996g03008.1

MGCFSCFDSKEEEKLNPQRDDRKEVHLTAPSNISRLSSGADRLKTRSINGSKREFLGLKDAPDVQIAAHTFTFREL
AAATSNFRPESFIGEGGFGRVYKGRLPSGQVVAVKQLDRNGLQGNREFLVEVLMLSLLHHPNLVNLIGYCADGD
QRLLVYEFMPLGSLEDHLHDLPPDKEPLDWNTRMKIAAGAAKGLEYLHDKANPPVIYRDFKSSNILLEENFFPKLS
DFGLAKLGPTGDKSHVSTRVMGTYGYCAPEYAMTGQLTVKSDVYSFGVVFLELITGRKAIDSTMPQGEQNLVA
WARPLFNDRRKFAKLADPRLQGQFPMRGLYQALAVASMCIQEQAAARPLIGDVVTALSYLANQVDDKRNKDD
RGGRICRNEDGAGGGSGRKWPDLDGGSEKEDSPRETARMLNRDLDRERAVAEAKMWGENWREKRRQNAQ
GSFDGTNG

>A717|Niben101Scf04343g00001.1

MGCFSCFDSKEEEKLNPQRDDRKEVHLTAPSNISRLSSGADRLKTRSINGSKREFLGLKDAPDVQIAAHTFTFREL
AAATSNFRPESFIGEGGFGRVYKGRLPSGQVVAVKQLDRNGLQGNREFLVEVLMLSLLHHPNLVNLIGYCADGD
QRLLVYEFMPLGSLEDHLHDLPPDKEPVDWNTRMKIAAGAAKGLEYLHDKANPPVIYRDFKSSNILLEENFFPKL
SDFGLAKLGPTGDKSHVSTRVMGTYGYCAPEYAMTGQLTVKSDVYSFGVVFLELITGRKAIDSTMPQGEQNLVA
WARPLFNDRRKFAKLADPRLQGQFPMRGLYQALAVASMCIQEQAAARPLIGDVVTALSYLANQVDDKRNKDD
RGGRICRNEDGAGGGSGRKWPDLDGGSEKEDSPRETARMLNRDLDRERAVAEAKMWGENWREKRRQNAQ
GSFDGTNG

>A718|PAN31545.1

MGFLACLCRCPQEEDDEEKEGEQFKINHQVASESCPLNTGSIVDIEGTQSISRHDEAIIFPLHQLADATKNFREDC
LLGRGGFGCVYKATLSDGQVVAVKKLDLNGLQGNREFLVEVLMLNLLHHPNLVNLFGYCVDGDQRLLVYEYMP
LGSLEDHLHDLAPDQEPLDWKTRMKIAAGAAAGLEYLHDKAHPPVIYRDIKPSNILLGEGYHAKLSDFGLAKLGP
VGDKTHVTTRVMGTHGYCAPEYASTGQLTIKSDIYSFGVVFLELITGRRPHDSNRPPEEQDLVPWACPLFKDQRK
FPRMADPLLHGRYPRRGLYQALAIAAMCLQKKAKNRPPIREVAAALSYLASQTYDRNNTAARRNRAGPSTSRVL
DDQMNQDTTLPSQQPVHCRMNHIIPEVKETSWSGSYRAGRGRVAPNGIDRERALADANVWAEAWRRQEKA
SKMR

>A719|PAN15404.1

MSCLPCSGSSGKDAKSLEALSPSPRPAAKSAPVRSNSRASGSRKEDSVPVRRGGNTSHGPAQIFTFRELAIATKNF
RKDCLLGEGGFGRVYKGRMENGQVIAVKQLDRNGFQGNREFLVEVLMLSLLHHPNLVRLIGYCADGDQRLLVY
EYMALGSLENHLYDKEPLDWNTRMKIAAGAAKGLEYLHDKANPPVIYRDFKSSNILLGEDYYPKLSDFGLAKLGP
VGDKTHVSTRVMGTYGYCAPEYAMTGQLTVKSDVYSFGVVFLELITGRKAIDHTQPSGEQNLVAWARPLFRDR
RKFCQLADPLLQGRYPKRGLYQALAVAAMCLQEQAASRPLIGDVVTALSYLASHPYDPNAPSTKDSRTCPSTPRA
KTHRRTTSVPDAQHAAESLMLNFPDLRKDTIRGGEFEQDRTEGSGSSSSSGRNDGLDVPQLLAAVKVDAREK

>A720|M0S5S6

MGCFPCFESPEEEDKKKRSEVGGGGDAKAETSAGVRFGKKFDKSKPRNVSDSKKEASAPKEENAGLIASRTFTFH
ELAAATRNFRQDCLLGEGGFGRVYKGMLDDGQVVAVKQLDLNGLQGNREFLVEVLMLCLLHHSNLVNLVGYC
ADGDQRLLVYEFMPLGSLQDHLHDIPPYKEPLDWNTRMKIAAGAAKGLEYLHDKADPPVIYRDFKSSNILLGEGY
HPKLSDFGLAKLGPVGDKTHVSTRVMGTYGYCAPEYAMTGQLTVKSDVYSFGVVFLELITGRKAIDVTRPAGEQ
NVVLWARPLFKDRRKFPKMVDPLLQGCYPIRGLYQALAVAAMCLQEQAATRPLIGEVVTALSYLASQSYDPNAT
AVQNAGICSSTQRVREDKRLLGGRFEVCTRVDVGRARGSPKNLSNGLVREGVVTEAKVWGKNLTEKEDRSAWY

>A721|A0A397ZS65

MGCFSCFNSSEDEKLNPVEESSKPQKQSQPIVSNNFSTLPSGGEKLSSKSNVRSKRELLLPRDELEQIAAHTFAFRD
LVAATMDFHPDTFLGEGGFGCVYKGRLESTGQVVAVKQLDRNGLQGNREFLVEVLMLSLLHHPNLVNLIGYCA
DGDQRLLVYEFMPLGSLEDHLHDLPPDKEALDWNMRMKIAAGAAKGLEFLHDKANPPVIYRDFKSSNILLAEGF
HPKLSDFGLAKLRPTGDKSHVSTRVMGTYGYCAPEYAMTGQLTVKSDVYSFGVVFLELITGRKAIDSEMPHGEQ
NLVAWARPMFNDRRRFIKLADPKLKGRFPTRALYQALSVASMCIQEEAATRPPIADVVTAISYLANQAYDPNKN
DRGGRNDEGGGKFDLAGSEKEDSPRDTARILNRDIDRKRAVAEAKMWGESSREKRRQSEKGTSESNSTTG

>A722|VDC78705.1

MGCFSCFNSSEDEKLNPVEESSKPQKQSQTIVSNNFSTLPSGGEKKLSSKSNVRSKRELLLPRDELEQIAAHTFAFH
ELVAATMDFHPDTFLGEGGFGCVYKGRLETTGQVVAVKQLDRNGLQGNREFLVEVLMLSLLHHPNLVNLIGYC
ADGDQRLLVYEFMPLGSLEDHLHDLPPDKEALDWNTRMKIAAGAAKGLEFLHDKANPPVIDRDFKSSNILLGEG
FHPKLSDFGLAKLGPTGDKSHVSTRVMGTYGYCAPEYAMTGQLTVKSDVYSFGVVFLELITGRKAIDSEMPHGE
QNLVAWARPMFNDRRRFIKLADPKLKGRFPTRALYQALSVASMCIQEEAATRPPIADVVTAISYLANQAYDPNK
NDRGGRNDEGGGKFDLAGSEKEDSPRETARILNRDIDRKRAVAEVKMWGESSREKRRQSEKGTSESNST

>A723|Bol034221

MGCFSCFHSSEDEKLNPVEESKPQKQSQPIVSNNFSTLPSGGEKKLSSKSNVRSKRELLLPRDGLEQIAAHTFAFRE
LVAATMNFHPDTFLGEGGFGCVYKGRLESTGQVVAVKQLDRNGLQGNREFLVEVLMLSLLHHPNLVNLIGYFA
DGDQRLLVYEFMPLGSLEDHLHDLPPDKEALDWNMRMKIAAGAAKGLEFLHDKANPPVIYRDFKSSNILLGEGF
HPKLSDFGLAKLGPTGDKSHVSTRVMGTYGYCAPEYAMTGQLTVKSDVYSFGVVFLELITGRKAIDSEMPHGEQ
NLLAWARPMFNDRRKFIKLADPKLKGRFPTRALYQALSVASMCIQEEAATRPLIADVVTALSYLASQAYDPNKNE
RGGGKLITRDDEGGGSEKEDSPRETARILNRDIDRERAVAEAKMWGGSLREKRRQSEQGTSESNSTG

>A724|M0U4I0.

MGCFPCFESKEGAQLGRGNESDRKREEQPMVAPRVEKLSLGNDDRKRTPNSLNTQKESLGEKRGAEFSISAQTF
TFRELAVATGNFRSECFLGEGGFGRVYRGRLESTGQVVAVKQLDRDGLQGNREFLVEVLMLSLLHHPNLVNLIG
YCADGEQRLLVYEFMPLGSLEDHLHDLPPDKSPLDWNTRMKIAAGAAKGLEYLHDKANPPVIYRDLKASNILLDK
GFHPKLSDFGLAKLGPVGDKSHVSTRVMGTYGYCAPEYALTGQLTVKSDVYSFGVVLLELITGRKAIDSTKTHAE

QNLVSWVRPMFKERRKLPGLADPKLQGRFPMRGLYQALAVASMCIQEEAASRPVIADVVTALSYLASQAYDPG
AASAVNTRPSGERTSRSGGEEGAKLTVRRALAEAKMWGQNWREKTQAKANVEGSFDVANAIG

>A725|LOC103858287

MGCFGRTGKSSKRSETKKKNNDFAKHNKLEIDANCVRKSADRDSQTHPSSEGSPRGDDVNKEVPSEEDQFSLD
AKDSNVEDEVSGKKAMTFAFEELSVSTGNFRSDCLLGEGGFGKVYKGLIERINQQVVAIKQLDRNGAQGIREFVV
EVMTLSLADHPNLVKLIGFCAQGVQRLLVYEYMPLGSLEDHLHDLPNGKTPLSWNNRMKIAAGAARGLEYLHD
TMKPPVIYRDLKSSNILLGEDYHPKLSDFGLAKVGPSGDATHVSTRVMGTYGYCAPEYAMTGQLTFKSDIFSFGV
VLLELITGMKAIYKTKARKDQTLVGWARPLFKDRKNFKKMVDPLMEGDYPVRGLYQALAIAAMCVQDQPGMR
PVISDVVMALDHLASCSYDATHKDKRTNVIENRVDDEEKKVTESNVCMEEEKQEIKICSAQAN

>A726|A0A0N7KKN3

IAAHTFTFRELAAATKNFRQDCLLGEGGFGRVYKGHLENGQAVAVKQLDRNGLQGNREFLVEVLMLSLLHHDN
LVNLIGYCADGDQRLLVYEFMPLGSLEDHLHDIPPDKEPLDWNTRMKIAAGAAKGLEFLHDKANPPVIYRDFKSS
NILLGEGYHPKLSDFGLAKLGPVGDKTHVSTRVMGTYGYCAPEYAMTGQLTVKSDVYSFGVVFLELITGRKAIDN
TKPLGEQNLVAWARPLFKDRRKFPKMADPLLAGRFPMRGLYQALAVAAMCLQEQAATRPFIGDVVTALSYLAS
QTYDPNTPVQHSRSNASTPRARNRVGANFDQRRLHSPNHQQSPDLRKEGTTTSKYEAEVSRTNSGSGSGRRAG
LDSMDVTGSQMGSPAHAGRKRESSRSTDRQRAVAEAKTWGENSRERKWPNARGSFDSTNE

>A727|A0A3B6AZH9

MGLREKTGIKCGAEKARAKGNAGMKELSDLRDANGNVLSAQTFTFRQLTAATRNFREECFIGEGGFGRVYKGR
LDGGQVVAIKQLNRDGNQGNKEFLVEVLMLSLLHHQNLVNLVGYCADGEQRLLVYEYMPLGSLEDHLHDLPPD
KEPLDWNTRMKIAAGAAKGLEYLHDKAQPPVIYRDFKSSNILLGDDFHPKLSDFGLAKLGPVGDKSHVSTRVMG
TYGYCAPEYAMTGQLTVKSDVYSFGVVLLELITGRKAIDSTRPHGEQNLVSWARPLFNDRRKLPKMADPGLQGR
YPMRGLYQALAVASMCIQSEAASRPLIADVVTALSYLASQIYDPNAIHASKKAGGDQRSRVSDSGRTLLKNDEAG
SSGHKSDRDDSPREPPPGILNDRERMVAEARMWGANLREKTRAAANAQGSLDSPTEIG

>A728|A0A446L2P2

MGLREKTGIKCGAEKARAKGNAGMKELSDLRDANGNVLSAQTFTFRQLTAATRNFREECFIGEGGFGRVYKGR
LDGGQVVAIKQLNRDGNQGNKEFLVEVLMLSLLHHQNLVNLVGYCADGEQRLLVYEYMPLGSLEDHLHDLPPD
KEPLDWNTRMKIAAGAAKGLEYLHDKAQPPVIYRDFKSSNILLGDDFHPKLSDFGLAKLGPVGDKSHVSTRVMG
TYGYCAPEYAMTGQLTVKSDVYSFGVVLLELITGRKAIDSTRPHGEQNLVSWARPLFNDRRKLPKMADPGLQGR
YPMRGLYQALAVASMCIQSEAASRPLIADVVTALSYLASQIYDPNAIHASKKAGGDQRSRVSDSGRTLLKNDEAG
SSGHKSDRDDSPREPPPGILNDRERMVAEAKMWGANLREKTRAAANAQGSLDSPTEIG

>A729|M0T1H7

MGCFPCFESRRGGVLNPGNGRDDSREEHPMVPPHIDRSSSVADSVNHRSNSGSKREAQSEKDVSSVNISAHTF
TFRELATATRNFRDECFLGEGGFGRVYKGRLESTGQIVAVKQLDREGLQGNREFLVEVLMLSLLHHPNLVNLIGY
CADGDQRLLVYEFLPLGSLEDHLHDLPPDKEPLDWNTRMKIAAGAAKGLEYLHDKASPPVIYRDFKSSNILLGEGF
HPKLSDFGLAKLGPTGDKSHVSTRVMGTYGYCAPEYAMTGQLTVKSDVYSFGVVLLELITGRKAIDSALPHGEQN
LISWARPMFNDRKKLPKLADPRLQGRFPMRGLYQALAVASMCIQEQAASRPGIADVVTALSYLANKAYDPNAS
PASRSEKEDSPREMAGIFKKDFDRERAVAEAKMWGENWRGKTRASSRNASSANS

>A730|3702_0:00207a

MGCFGRTPKSNKRSDTKTTKNNDFTPKKLTVNANRDKLTQPSSDCLKVSICGDVSKEIVTKKDQLALDAKDTNVE
DEVIVKKAQTFTFEELSVSTGNFKSDCFLGEGGFGKVYKGFIEKINQVVAIKQLDRNGAQGIREFVVEVLTLSLADH
PNLVKLIGFCAEGVQRLLVYEYMPLGSLDNHLHDLPSGKNPLAWNTRMKIAAGAARGLEYLHDTMKPPVIYRDL
KCSNILIDEGYHAKLSDFGLAKVGPRGSETHVSTRVMGTYGYCAPDYALTGQLTFKSDVYSFGVVLLELITGRKAY
DNTRTRNHQSLVEWANPLFKDRKNFKKMVDPLLEGDYPVRGLYQALAIAAMCVQEQPSMRPVIADVVMALD
HLASSKYDRSHRQKQDNVTETKVDEEKTLTTESNVCVEEKQEIKICSDQAT

>A731|PAN15785.1

MLSWLRRFPHDVVHRKENAGRRTSSTTTWRNRSSSFTARIIRCASSVVDATAKHDDDDDCPLPSSPPPPAPPPP
PEDDRVTVVSAQAFSFRELAEAAGNFRQDKLIGEGGFGRVYKGRLPAGEQEEQQRVVAVKQLDRNGRQGNSE
FVVEVLMLSMLHHPNLVRLVGYCAEGEQRLLVYEYMALGSLEDHLLLPMRRDDALLPWRTRMRVAHGAARGL
EYLHDRAVIYRDLKSSNILLADDYSPRLSDFGLARLLPLPSSSSSSSSSSSSSGTGRSRVMGTYGYCAPEYLRTGKLS
AKSDVYSFGVLLLELITGRRAIDASQPDGEQSLVGWAATMFGDPRRFQELVDPRLVMAMQGPPASELKQAVG
VAAMCLQENHALRPVMTDVVIALSFLAASTTHDHHHHHPPPLRPSPDPSYAS

>A732|LOC8072900

MLSWLRRFPNDVIHRKGGNGTTGRRTSSTTSWRNKSNSFTARIIRCASSVVDTAGRRHDDDDDDDDDCRLPSS
PPPPPPPPPAAPPNDNNRNTSVVLAAKAFSFRELADAAGNFRQANFLGEGGFGRVYKGRLHNNIAGEDLPVAIK
QLDRNGFQGNNEFMVEVLMLSMLHHPNLVSLVGYCAEGDQRLLVYEYMALGSLEDHLLLLPADADDGSHQR
QPLPWRTRMKIALGAAQGLEYLHENTVIYRDLKSSNILLDQDYSPKLSDFGLAKLLPAPRTDYSSSSSRSSSSSSSSKV
MGTYGYCAPEYLRTGKLSVKSDVYSFGVLLLELITGRRAIDASRPDGEQSLVGWAARIFGDPKRFHELVDPRLVM
AMRVPTTSELKQAVGVASMCLQEHYALRPVMTDVVVALSFLATDSPPC

>A733|A0A5A7RBL2

MGWFLCSGKSRKKANKGQDKKCDDQIPSNIEKLKANPSFDLQKEASKDGGSRHIAAHTFTFRELAMATKNFRA
ECLLGEGGFGRVYKGRLESTNQVVAIKQLDRNGLQGNREFLVEVLMLSLLHHPNLVNLIGYCADGDQRLLVYEY
MPLGSLEDHLHDLPSDKKRLDWNTRMKIAAGAAKGLEYLHDKANPPVIYRDLKCSNILLDEGYHPKLSDFGLAKL
GPVGDKTHVSTRVMGTYGYCAPEYAMTGQLTLKSDVYSFGVVLLEIITGRKAIDNSRAAGEHNLVAWARPLFKD
RRKFSQMADPTLQGQYPSRGLYQALAVAAMCVQEQPNMRPLIADVVTALTYLASQKFDPEAQQPAKKPSSGP
STPTMRWELRDSMSLATVVKICLKGRLGLLSPILRYYHLFLLM

>A734|LOC111258252

MLSWLRRFPHDVLHRKDNGGAGRRAASSTTWRNKNNNSFTARIIRCASSAARRRFDDDDDDRQLPSSPPPPG
PPPAEEEEDDDDDDYSRSKDRNNKVGVVSARAFSFRELAEAAGNFRQEHLIGEGGFGRVYKARIKEQQEQVVV
AVKQLDRNGPQGNGEFVVEVLMLSMLHHPNLVSLLGYCAEGEQRLLVYEYMALGSLEDHLLLISNHPLLPWRTR
MRIACGAGRGLEYLHERGVIFRDLKPSNILLDDHHNPRLSDFGLARLLPPSNSSSSSNSSSSSTGSSRVMGTYGYC
APEYLRTGKLSAKSDVYSFGVLLLELITGRRALDASRPDGEQSLVGWAAPMFGDPTRIHELVDPRLVMAMQGPP
APELKQAVGLAAMCLQEHHALRPVMTDVVFALDFLSTDRP

>A735|I1IXA6

MKELSVLRDANGNALSAQTFTFRQLTAATRNFREECFIGEGGFGRVYKGRLDGSQVVAIKQLNRDGNQGNKEF
LVEVLMLSLLHHQNLVNLVGYCADGEQRLLVYEYMALGSLEDHLHDLPPDKESLDWNTRMKIAAGAAKGLEYL
HDKAQPPVIYRDFKSSNILLGDDFHPKLSDFGLAKLGPVGDKSHVSTRVMGTYGYCAPEYAMTGQLTVKSDVYS
FGVVLLELITGRKAIDSTRPHGEQNLVSWARPLFSDRRKLPKMADPGLQGRYPSRGLYQALAVASMCIQSEAAS

RPLIADVVTALSYLAAQTYDPNAIHASKKAGSDQRSRVGDSGRVLLKNDEAGSFGDKSDRDDSPRERPLGILNDR
ERMVAEAKMWGTNLREKTRAAANAQGNLDSPT

>A736|A0A453GIB0

MLGEGGFGRVYKGRLESGQAVAVKQLDRNGLQGNREFLVEVLMLSLLHHTNLVNLIGYCADGDQRLLVYEFMP
LGSLEDHLHDVPPEKEPLDWNTRMKIAAGAAKGLEHLHDKASPPVIYRDFKSSNILLGEGFHPKLSDFGLAKLGP
VGDNTHVSTRVMGTYGYCAPEYAMTGQLTVKSDVYSFGVVFLELITGRKAIDNTKPQGEQNLVAWARPLFKDR
RKFPKMADPMLQGRFPMRGLYQALAVAAMCLQEQATTRPHIGDVVTALSYLASQTYDPNAPTQHTRSNSSTP
RARNVGGRNSEQRNGRSPNHHSPRTSKHGGEVSRTSSTGGDSGRRSGLDEMDMAGSQAGSPAQTGRKRETP
RTADRQRAIADAKMWGENSRERKRPNDSFDSTNE

>A737|A0A1U8A1Q9

MGWFPCSGKSNKKSKKKKPDDQQIQSTSEKLKVNPSLDVKKESSKDGSDRIAAQTFTFRELAAATKNFRADCLL
GEGGFGRVYKGRLESINQIVAIKQLDRNGLQGNREFLVEVLMLSLLHHPNLVNLIGYCADGDQRLLVYEYMPLGS
LEDHLHDLPPDKKRLDWNTRMKIAAGAAKGLEYLHDKANPPVIYRDLKCSNILLGEGYHPKLSDFGLAKLGPVGD
KTHVSTRVMGTYGYCAPEYAMTGQLTLKSDVYSFGVVLLEIITGRKAIDNSKAAGEHNLVAWARPLFKDRRKFS
QMADPMLQGQYPVRGLYQALAVAAMCVQEQPTMRPLIADVVTALTYLASQTYDPDTQPVQSSRMYPSTPPR
TKRDGDKRLNGGNGYERDQGRGLK

>A738|EMS50039.1

MDEERKPRPGRSATFRKKHCPDAAPSRKRFIRSGTSLTASSSARASFGRHSVDVPNYNHSIVSARSFTFPELAAAT
DCFSHANLIGEGGFGRVYRGLIGSSAVAVKQLDRTGFQGDHEFLVEVLVLSSLLTHPNLVGLLGYCADGNQRLLV
YQLMPLGSLENHLFLPRVPADGEEKPPPPVLPWRTRMRIAHDAAQGLEFLHETANPPVIYRDLKSSNILLDEGYN
AKLSDFGLAKLATPITRNGKGGEAEEEKDGPSRVMGTYGYCAPEYVRTGHLTVKSDVYSFGVVLLELITGRRVIDD
SRPSGEQNLVAWAAPMFSEQRRMHELVDPLLGEGPSGREVKQAVAVAAMCLQEEDTVRPIMSDVVMALSFA
ADDDLPSAPRYTSL

>A739|XP_021816394.1

MGCFPCFDSKEEEKLNNPVNEIDDRKQGQPTVSSNISRLPSGADRMRSRSNGGSRRDLGSKLPDLKDVPGVQIA
AQIFTFRELVTATKNFRPESFIGEGGFGRVYKGRLESSSQVVAVKQLDRNGLQGNREFLVEVLMLSLLHHPNLVN
LIGYCADGDQRLLVYEFMPLGSLEDHLHDLPPDREPLDWNTRMKIASGAAKGLEYLHDKANPPVIYRDFKSSNIL
LEEGFHPKLSDFGLAKLGPTEDKSHVSTRVMGTYGYCAPEYAMTGQLTVKSDVYSFGVVFLELITGRKSIDSNRP
HGEQNLITWARPLFNDRRKFSKLADPRLQGRYPMRGLYQALAVASMCIQEQAATRPLIGDVVTALSYLANQSY
DPNTASGHGHRGSGEKDERRHRDERGGRILKNEEGGGSGRRWDLDGSEKDDSLKETARMLNNRDLDRERAV
AEAKMWGENWREKRRQSAQGS

>A740|A0A061FN09

MGWFPCGGKSSKNAKKKLPNNNNSSSNRNSDDQIPSTSEKLKVNSVPDGKEEATKDGNSDHIAAHTFTFRELA
AATKNFRADCLLGEGGFGRVYKGRLESTNQVVAIKQLDRNGLQGNREFLVEVLMLSLLHHPNLVNLIGYCADGD
QRLLVYEYMPLGSLEDHLHDLPPDKRQLDWNTRMKIAAGAAKGLEYLHDKASPPVIYRDLKCSNILLGEGYHPKL
SDFGLAKLGPVGDKTHVSTRVMGTYGYCAPEYAMTGQLTLKSDVYSFGVVLLEIITGRKAIDNSRAGGEQNLVA
WARPLFKDRRKFAQMADPLLQGQYPVRGLYQALAVAAMCVQEQPNMRPLIADVVTALTYLASQKFDPETQSV
QGSRTGSSTPRMRRE

>A741|A0A0B0PQS7

MGWFICGGNSNQNEKKKLPINNNTTTTNNFDHQIPSTSEKLKVNSAPNTKKEATKDGGSDHIAAHTFTFRELAA
STKNFRPDCLLGEGGFGRVYKGRLESSNQVVAIKQLDPNGLQGNREFLVEVLMLSLLHHPNLVNLIGYCADGDQ
RLLVYEYMPLGSLEDHLHDLPPDRRRLDWNTRMKIAAGAAKGLEYLHDKASPPVIYRDLKCSNILLGEGYHPKLS
DFGLAKLGPVGDKTHVSTRVMGTYGYCAPEYAMTGQLTLKSDVYSFGVVLLEIITGRKAIDNSRAGGEQNLVAW
ARPLFKDRRKFAQMADPLLQGQYPVRGLYQALAVAAMCVQEQPNMRPLIADVVTALTYLASQRYDPETQSVQ
GARTASSTPRMRRE

>A742|A0A0D2QRJ0

MGWFPCGGKPNKKGKKKLPNHNNTTTINSSDDQIPSTSEKLKVNSVPDAKKEANKDGGSDHIAAHTFTFRELA
AATKNFRADYLLGEGGFGRVYKGRLESTNQVVAIKQLDRNGLQGNREFLVEVLMLSLLHHPNLVNLIGYCADGD
QRLLVYEYMPLGSLEDHLHELPPDKRQLDWNTRMKIAAGAAKGLEYLHDKASPPVIYRDLKCSNILLGEGYHPKL
SDFGLAKLGPVGDKTHVSTRVMGTYGYCAPEYAMTGQLTLKSDVYSFGVVLLEIITGRKAIDNSRAGGEQNLVA
WARPLFKDRRKFAQMADPLLQGQYPVRGLYQALAVAAMCVQEQPNMRPLIADVVTALSYLASQKYDPGTQT
VQGSRTGSSTPRMRRE

>A743|A0A0D2UXK7

MGWFLCGGNSNQDEKKKLPINNNTTTTNNFDHQIPSTSEKLKVNSAPNTKKEATKDGGSDHIAAHTFTFRELAA
ATKNFRADCLLGEGGFGRVYKGRLESTNQVVAIKQLDPNGLQGNREFLVEVLMLSLLHHPNLVNLIGYCADGDQ
RLLVYEYMPLGSLEDHLHDLPPDRRRLDWNTRMKIAAGAAKGLEYLHDKASPPVIYRDLKCSNILLGEGYHPKLS
DFGLAKLGPVGDKTHVSTRVMGTYGYCAPEYAMTGQLTLKSDVYSFGVVFLEIITGRKAIDNSRAGGEQNLVA
WARPLFKDRRKFAQMADPLLQGQYPVRGLYQALAVAAMCVQEQPNMRPLIADVVTALTYLASQRYDPETQSV
QGPRTGSSTPRMRRE

>A744|A0A1U8LTK1

MGWFLCGGNSNQDEKKKLPINNNTTTNNNFDHQIPSTSEKLKVNSAPNTKKEATKDGGSGHIAAHTFTFRELA
AATKNFRADCLLGEGGFGRVYKGRLESTNQVVAIKQLDPNGLQGNREFLVEVLMLSLLHHPNLVNLIGYCADGD
QRLLVYEYMPLGSLEDHLHDLPPDRRRLDWNTRMKIAAGAAKGLEYLHDKASPPVIYRDLKCSNILLGEGYHPKL
SDFGLAKLGPVGDKTHVSTRVMGTYGYCAPEYAMTGQLTLKSDVYSFGVVFLEIITGRKAIDNSRAGGEQNLVA
WARPLFKDRRKFAQMADPLLQGQYPVRGLYQALAVAAMCVQEQPNMRPLIADVVTALTYLASQRYDPETQSV
QGPRTGSSTPRMRRE

>A745|A0A1U8M5M5

MGWFICGGNSNQDEKKKLPINNNTTTNNNFDHQIPSTSEKLKVNSAPNTKKEATKDGGSDHIAAHTFTFRELAA
STKNFRADCLLGEGGFGRVYKGRLESSNQVVAIKQLDPNGLQGNREFLVEVLMLSLLHHPNLVNLIGYCADGDQ
RLLVYEYMPLGSLEDHLHDLPPDRRRLDWNTRMKIAAGAAKGLEFLHDKASPPVIYRDLKCSNILLGEGYHPKLS
DFGLAKLGPVGDKTHVSTRVMGTYGYCAPEYAMTGQLTLKSDVYSFGVVLLEIITGRKAIDNSRAGGEQNLVAW
ARPLFKDRRKFAQMADPLLQGQYPVRGLYQALAVAAMCVQEQPNMRPLIADVVTALTYLASQRYDPETQSVQ
GARTASSTPRMRRE

>A746|A0A1U8PAV5

MGWFPCGGKPNKKGKKKLPNHNNTATINSSDDQIPSTSEKLKVSSVPDAKKEANKDGGSDHIAAHTFTFRELAA
ATKNFRADYLLGEGGFGRVYKGRLESTNQVVAIKQLDRNGLQGNREFLVEVLMLSLLHHPNLVNLIGYCADGDQ

RLLVYEYMPLGSLEDHLHDLPPDKRKLDWNTRMKIAAGAAKGLEYLHDKASPPVIYRDLKCSNILLGEGYHPKLS
DFGLAKLGPVGDKTHVSTRVMGTYGYCAPEYAMTGQLTLKSDVYSFGVVLLEIITGRKAIDNSRAGGEQNLVAW
ARPLFKDRRKFAQMADPLLQGQYPVRGLYQALAVAAMCVQEQPNMRPLIADVVTALTYLASQKYDPGTQTVQ
GSRTGSSTPRMRRE

>A747|A0A5D2XQG8

MGWFICGGNSNQDEKKKLPINNNSTTNNNFDHQIPSTSEKLKVNSAPNTKKEATKDGGSDHIAAHTFTFRELAA
STKNFRADCLLGEGGFGRVYKGRLESSNQVVAIKQLDPNGLQGNREFLVEVLMLSLLHHPNLVNLIGYCADGDQ
RLLVYEYMPLGSLEDHLHDLPPDRRRLDWNTRMKIAAGAAKGLEYLHDKASPPVIYRDLKCSNILLGEGYHPKLS
DFGLAKLGPVGDKTHVSTRVMGTYGYCAPEYAMTGQLTLKSDVYSFGVVLLEIITGRKAIDNSRAGGEQNLVAW
ACPLFKDRRKFAQMADPLLQGQYPVRGLYQALAVAAMCVQEQPNMRPLIADVVTALTYLASQRYDPETQSVQ
GARTASSTPRMRRE

>A748|XP_031282179.1

MGCFPCFDSREEEKLNPVKESDDQKQGQPTVSNNISRLPSGRERLRSRSNGGSKRELPVPKELPGGANIAAQTFT
FRELAAATRNFRPECFIGEGGFGRVYKGRLESTGQVVAVKQLDRNGLQGNREFLVEVLMLSLLHHPNLVNLIGYC
ADGDQRLLVYEFMPMGSLEDHLHDLPPDKEPLDWNTRMKIAAGAAKGLEYLHDKANPPVIYRDFKSSNILLDE
GFHPKLSDFGLAKLGPVGDKSHVSTRVMGTYGYCAPEYAMTGQLTVKSDVYSFGVVFLELITGRKAIDSTRPHGE
QNLVTWARPLFNDRRKFAKLADPQLQGRYPMRGLYQALAVASMCIQEQAATRPLIGDVVTALSYLANQAYDP
NTASHGYRGSGDKEDRRSKDDRGGRILRNEEGGGSGRRWDLEGSEKEDSPRETGRMLNRDLDRERAVAEAKM
WGENWREKRRQSAQGSFDGNN

>A749|A0A446J1G7

MGCLPCFGSSGKGEPAKKGGARKDVPSDRRATGVGSDKPKPQGLLDSKDAVIPREGNNQHIAAHTFTFRELAA
ATKNFRQDCLLGEGGFGRVYRGRLDNGQAVAVKQLDRNGLQGNREFLVEVLMLSLLHHDNLVNLIGYCADGD
QRLLVYEYMPLGSLEDHLHDIPPEKEPLDWNTRMKIAAGAAKGLEYLHDKASPPVIYRDFKSSNILLGEEFHPKLS
DFGLAKLGPVGDKTHVSTRVMGTYGYCAPEYAMTGQLTVKSDVYSFGVVFLELITGRKAIDNTKPHGEQNLVA
WARPLFKDRRKFPKMADPALQGRFPMRGLYQALAVAAMCLQEQAATRPFIGDVVTALSYLASQAYDPNAPTQ
HSRSNASTPRARDRSS

>A750|A0A5B6WKV1

MGWFLCGGNSNQDGKKKLPINNNTTTNNFDHQIPSTSEKLKVHSAPNTKKDATKDGGSDHIAAHTFTFRELAV
ATKNFRADCLLGEGGFGRVYKGRLESTNQVVAIKQLDPNGPQGNREFLVEVLMLSLLHHPNLVNLIGYCADGD
QRLLVYEYMPLGSLEDHLHDLPPDRRRLDWNTRMKIAAGAAKGLEYLHDKASPPVIYRDLKCSNILLGEGYHPKL
SDFGLAKLGPVGDKTHVSTRVMGTYGYCAPEYAMTGQLTLKSDVYSFGVVLLEIITGRKAIDNSRAGGEQNLVA
WARPLFKDRRKFAQMADPLLQGQYPVRGLYQALAVAAMCVQEQPNMRPLIADVVTALTYLASQRYDPETQSV
QGARTGSSTPRMRRE

>A751|LOC109785675

MGCCTSSQKKGAAAGDRRKEPAEKPSQIAPAASPSPTPLPSAEISRLEASQGAVRTKFISSPGPHTNRLSFTYEEL
NAATLGFPRDHFLGEGGFGKVYKGVLDGNEVAIKILNPNGLQGNREFCTEVMVLSRMHHPNLVKLVGFCADDD
QRLLVYEYMPLGSLETHIFDLPPDKKPIDWNTRIKILVGAAQGLKHLHVNCNPPIINRDVKCANILLGEEYHPKLAD
FGLAKLGPTGDDTHVSTRVMGTPGYCAPEYLESGQLTIKSDVYSFGVVILEVITGRKALDQSRIKAERSLAEWATP

LINRRDFAMLADPALGNQYSMTSLYQVLSVARMCLNKTASQRPQITDVAAALAHISKSRRTRRLAHQQSAAQV
HQPGEDI

>A752|A0A3N7FDT6

MGCFSCAGKSSKNDSKKKPDDQIPSSSDGVNFISDKTKSNGAPDVKEASKDGGSEHIAAHTFTFRELANATKNFR
ADCLLGEGGFGRVYKGRLESTNQVVAIKQLDRNGLQGNREFLVEVLMLSLLHHPNLVNLIGYCADGDQRLLVYE
YMPLGSLEDHLYDLPPDKKRLDWNTRMKIAAGAAKGLEHLHDKANPPVIYRDLKCSNILLGEGYHPKLSDFGLAK
LGPVGDKTHVSTRVMGTYGYCAPEYAMTGQLTLKSDVYSFGVVLLEIITGRKAIDNSRAAGEHNLVAWARPLFK
DRRKFAQMADPLLHGQYPARGLYQALAVAAMCVQEQPNMRPLIADVVTALSYLALQKYDPETHPVQGSRTGP
STPRTRREQ

>A753|A0A251R1C9

MKLMTGSRVNQRSLLTFPDCLLVVAVKQLDRNGLQGNREFLVEVLMLSLLHHPNLVNLIGYCADGDQRLLVYEF
MPLGSLEDHLHDLPLDREPLDWNTRMKIASGAAKGLEYLHDKANPPVIYRDFKSSNILLEEGFHPKLSDFGLAKL
GPTEDKSHVSTRVMGTYGYCAPEYAMTGQLTVKSDVYSFGVVFLELITGRKSIDSNRPHGEQNLITWARPLFND
RRKFSKLADPRLQGRYPMRGLYQALAVASMCIQEQAATRPLIGDVVTALSYLANQSYDPNMASGHGHRGSGEK
DERRHRDERGGRILKNEEGGGSGRRWDLDGSEKDDSPKETARMLNRDLDRERAVAEAKMWGENWREKRRQ
SAQGSFDGTNL

>A754|A0A5J5ATP3

MGWFLCSGKSKKNAKKKHNKKPDDQIPSTSEKLKEKSSFDLKKEASKDGGSNHIAAHTFTFRELAAATKNFKADC
LLGEGGFGRVYKGCLESTNQIVAIKQLDRNGLQGNREFLVEVLMLSLLHHTNLVNLTGYCADGDQRLLVYEYMP
LGSLEDHLHDLPPDKKRLDWNIRMKIAAGAAKGLEYLHDKVNPPVIYRDLKCSNILLDEGYHPKLSDFGLAKLGPV
GDKTHVSTRVMGTYGYCAPEYAMTGQLTLKSDVYSFGVVLLEIITGRKAIDNSKAAGEHNLVAWARPLFKDRRK
FSQMADPLLQGQYPVRGLYQALAVAAMCVQEQPNMRPLIADVVTALTYLASQKYDPETQPVQSIRSGSSTPRT
RREQ

>A755|W9RNM0

MFWFLCSGKSNKNPNKDKHSQKPEDQISSASEKSKVNSVDGKKESSRDGGSDHIAAHTFTFRELAAATKNFRAD
CLLGEGGFGRVYKGRLESTSQVVAIKQLDRNGLQGNREFLVEVLMLSLLHHPNLVNLIGYCADGDQRLLVYEYM
PLGSLEDHLHDLPPDKKRLDWTTRMKIAAGAAKGLENLHDKANPPVIYRDLKCSNILLGEGYYPKLSDFGLAKLG
PVGDKTHVSTRVMGTYGYCAPEYAMTGQLTLKSDVYSFGVVLLEIITGRKAIDNSRSAGEHNLVAWARPLFKDR
RKFSQMADPLLQGQYPVRGLYQALAVAAMCVQEQPTMRPLIADVVTALSYLANQKYEPETASVQGFHTSSSTP
RSRREF

>A756|A0A4D8YXQ6

MGWFLCSGKSKKRVKKEHDKKCDDQIPSEKLKANPLFDVKKEPSKDGGSGRIAAHTFTFRELATAAKNFRPDCLL
GEGGFGRVYKGRLESTNQVVAIKQLDRNGLQGNREFLVEVLMLSLLHHPNLVNLIGYCADGDQRLLVYEFMPLG
SLEDHLHDLPPDKKRLDWNTRMKIAAGAAKGLEYLHDKANPPVIYRDLKCSNILLDEDYHPKLSDFGLAKLGPVG
DKTHVSTRVMGTYGYCAPEYAMTGQLTLKSDVYSFGVVLLEIITGRKAIDNSRAAGEHNLVAWARPLFKDRRKF
SQMADPTLHGQYPARGLYQALAVAAMCVQEQPNMRPLMADVVTALTYLASQKYDPETQPVQRPSSSSSTPR
MRRESR

>A757|A0A4D9A6R7

MGWFLCSGKSKKRVKKEHDKKCDDQISSEKLKANPLFDVKKEPSKDGGSGHIAAHTFTFRELATAAKNFRPDCLL
GEGGFGRVYKGRLESTNQVVAIKQLDRNGLQGNREFLVEVLMLSLLHHPNLVNLIGYCADGDQRLLVYEFMPLG
SLEDHLHDLPPDKKRLDWNTRMKIAAGAAKGLEYLHDKANPPVIYRDLKCSNILLDEDYHPKLSDFGLAKLGPVG
DKTHVSTRVMGTYGYCAPEYAMTGQLTLKSDVYSFGVVLLEIITGRKAIDNSRAAGEHNLVAWARPLFKDRRKF
SQMADPTLQGQYPARGLYQALAVAAMCVQEQPNMRPLMADVVTALTYLASQKYDPETQPVQRPSSSSSTPR
MRRESR

>A758|S8BXP4

MGWFLCYGKSKKKTRKAEEKASDDQIPSNREKIKVNSPFDLKEGSKDGGSGHIAAHTFAFRELATATKNFRADCL
LGEGGFGRVYKGRLESTNQVVAIKQLDRNGLQGNREFLVEVLMLSLLHHPNLVNLIGYCADGDQRLLVYEYMPL
GSLEDHLHDLPPDKKRLDWNTRMKIAAGAAKGLEYLHDKANPPVIYRDLKCSNILLGEGYHPKLSDFGLAKLGPV
GDKTHVSTRVMGTYGYCAPEYAMTGQLTLKSDVYSFGVVLLEIITGRKAIDNSRAAGEHNLVAWARPLFKDRRK
FYQMADPVLQGHYPARGLYQALAVAAMCVQEQPNLRPLIADVVTALTYLASQKFDHEAQPPPAQLPGSGSSTP
RMRR

>A759|A0A2G9HWA9

MGWFLCSGKSRKKTKKEQEKNSDDQIPSNIEKLKVNPSFDVKKEGSKDGGSGHIAAHTFTFRELATATKNFRAD
CLLGEGGFGRVYKGRLESTNQIVAIKQLDRNGLQGNREFLVEVLMLSLLHHPNLVNLIGYCADGDQRLLVYEYMP
LGSLEDHLHDLPPDKKRLDWNTRMKIAAGAAKGLEYLHDKANPPVIYRDLKCSNILLDEGYHPKLSDFGLAKLGP
VGDKTHVSTRVMGTYGYCAPEYAMTGQLTLKSDVYSFGVVLLEIITGRKAIDNSRSAGEHNLVAWARPLFKDRR
KFSQMADPVLQGQYPSRGLYQALAVAAMCVQEQPNMRPLIADVVTALTYLASQKYDPETQPAQRPSSGSSTP
RMRR

>A760|A0A068U7R9

MGCFLCTGKSKKKIESQKSHKSDDQIPSSIEKLKVNPSFSVKSEASKDGGSGHIAAHTFTFRELAAATKNFRADCLL
GEGGFGKVYKGRLEGTNQVVAIKQLDRNGLQGNREFLVEVLMLSLLHHPNLVNLIGYCADGDQRLLVYEYMPL
GSLEDHLHDLPPDKDPLDWNTRMKIAAGAAKGLEYLHDKASPPVIYRDLKCSNILLDEGYYPKLSDFGLAKLGPV
GDKTHVSTRVMGTYGYCAPEYAMTGQLTLKSDVYSFGVVLLEIITGRKAIDNSRGAGEHNLVAWARPLFKDRRK
FSQMADPLLQGQYPSRGLYQALAVAAMCVQEQPNMRPLMADVVTALSYLASQRYDPETQPVQRQRSGASTP
RTR

>A761|A9PFC3

MGCFSCAGKSSKNDSKKKPDDQIPSSSDKTKSNGAPDVKEASKDGGSEHIAAHTFTFRELANATKNFRADCLLG
EGGFGRVYKGRLESTNQVVAIKQLDRNGLQGNREFLVEVLMLSLLHHPNLVNLIGYCADGDQRLLVYEYMPLGS
LEDHLYDLPPDKKRLDWNTRMKIAAGAAKGLEHLHDKANPPVIYRDLKCSNILLGEGYHPKLSDFGLAKLGPVGD
KTHVSTRVMGTYGYCAPEYAMTGQLTLKSDVYSFGVVLLEIITGRKAIDNSRAAGEHNLVAWARPLFKDRRKFA
QMADPLLHGQYPARGLYQALAVAAMCVQEQPNMRPLIADVVTALSYLALQKYDPETHPVQGSRTGPSTPRTR
REQ

>A762|A0A452YYY3

LCIRYRNKPKPQGLLDSKKDTVIPREGNNQHIAAHTFTFRELAAATKNFRQDCLLGEGGFGRVYRGRLDNGQAV
AVKQLDRNGLQGNREFLVEVLMLSLLHHDNLVNLIGYCADGDQRLLVYEYMPLGSLEDHLHDIPPEKEPLDWNT
RMKIAAGAAKGLEYLHDKASPPVIYRDFKSSNILLGEEFHPKLSDFGLAKLGPVGDKTHVSTRVMGTYGYCAPEY

AMTGQLTVKSDVYSFGVVFLELITGRKAIDNTKPHGEQNLVAWARPLFKDRRKFPKMADPALQGRFPMRGLYQ
ALAVAAMCLQEQAATRPFIGDVVTALSYLASQAYDPNAPTQHSRSNASTPRARDRGSVNGDQRRIRSP

>A763|A0A287MGW0

LAAATKNFRQDCMLGEGGFGRVYKGRLENGQAVAVKQLDRNGLQGNREFLVEVLMLSLLHHTNLVNLIGYCA
DGDQRLLVYEFMPLGSLEDHLHDVPPEKEPLDWNTRMKIAAGAAKGLEHLHDKASPPVIYRDFKSSNILLGEGF
HPKLSDFGLAKLGPVGDKTHVSTRVMGTYGYCAPEYAMTGQLTVKSDVYSFGVVFLELITGRKAIDNTKPQGEQ
NLVAWARPLFKDRRKFPKMADPMLQGRFPMRGLYQALAVAAMCLQEQATTRPHIGDVVTALSYLASQTYDP
NAPTQHTRSNSRTSSNGGDSGRRSGLDDMDMAGSQAGSPAQTGRKRDTPRTADRHRAVVDAKMWGE

>A764|A0A453GIE1

TKNFRQDCMLGEGGFGRVYKGRLESGQAVAVKQLDRNGLQGNREFLVEVLMLSLLHHTNLVNLIGYCADGDQ
RLLVYEFMPLGSLEDHLHDVPPEKEPLDWNTRMKIAAGAAKGLEHLHDKASPPVIYRDFKSSNILLGEGFHPKLS
DFGLAKLGPVGDNTHVSTRVMGTYGYCAPEYAMTGQLTVKSDVYSFGVVFLELITGRKAIDNTKPQGEQNLVA
WARPLFKDRRKFPKMADPMLQGRFPMRGLYQALAVAAMCLQEQATTRPHIGDVVTALSYLASQTYDPNAPT
QHTRSNSSTPRARNVGGRNSEQLDMAGSQAGSPAQTGRKRETPRTADRQRAIADAKMWGE

>A765|A0A067GU08

MLSLLHHSNLVNLIGYCADGDQRLLVYEFMPLGSLEDHLHDLPPDKEALDWNTRMKIAAGAAKGLEYLHDKAN
PPVIYRDFKSSNILLEEGFHPKLSDFGLAKLGPVGDKSHVSTRVMGTYGYCAPEYAMTGQLTVKSDVYSFGVVFLE
LITGRKAIDSTRPHGEQNLVTWARPLFNDRRKFSKLADPRLEGRYPIRGLYQALAVASMCIQEQAATRPLIGDVV
TALSYLANQTYDPNSHRGAGDKDDRRNRDGARIFKNDEGGGSGRRWDLEGSEKEDSPRETARILNRDLERERA
VAEAKMWGENLREKRRQSAQGSFDGTNG

>A766|Cre16.g659400.t1.1

MSSGRKRTKADVCEAFVARLSERGDVELDSSLLEGIRQHFDRLPTRYALDVNVDGLDVLSHKRLLDEARADPTTV
SFAVRPVEIVGPRADGVSSPHELTRANSKKLGQLCRPAFGSSPNLQALALEVGERAEGHEAEASSGPGPREEHPV
FYEITIASVDQPKLLSRLSEALGDLGLNIREAHAFNTNDSFSLDVFVVDQWQPQPGQNLEELLGQRLLQMPPPPP
KGGAAQQQQAVAAGPAPALRLPADPPPNLSMLAGPRPDSPAVDDWEIDITQLHIEAKIASGAFSNLYKGTYCG
QEVAVKILKDVHDDSSQYQEFLQEVAIMRKVRHKNVVQFIGACTRKPNLCIVFEYMSGGSVYDYIRRQEGPLKLS
AILKLAADVARGMDYLHQRKIIHRDLKAANLLMDDNAIVKIADFGVARVIETTGHMTAETGTYRWMAPEVIEHK
PYDEKADVFSFGIVLWELLTCKVPYADMTPLQAAVGVVQKGLRPGVPANCPPLLGELMEACWTGNPASRPSFR
ELTPRLQHLNAMALEEEKRQLEAKPASKQGLLSKLRGK

>A767|Cre03.g166750.t1.2

MTLEQLEDRNSSHGGTCAGSNSCLTRGRRSSATETLALEELPSVGHLAEAGWTRALADNIKSVAAVTGVMGGG
SMPNSPHGSVLGARGSLTGVKYGTALGVGAAPNAVMAGFRPSGSGLKSSSGAGGLPGGPVGSATLVTELEEFD
GGDDVEAALNVVPDALMQQAVKAGVNLQVCMDTDVTLSSEQLGRGVSGTVVKGTYRGQPAAIKMLPPDLLF
GNRSLELHTFVQEMVVLCGVRHPNIVNLLGGSLQPPNVFIVEELCVGSLEARIHGGPGKNAAAPKALSAYEQLRI
AVDVATGLQYLHERTPAIVHRDLKPANILIDPNGTAKISDFGLARVKTHAVINTKAPDVGSIGYMAPECFTNEDG
QLTDKCDTWSLGVTIWEMVTRKRPWASCNMAEYYREVVIRKSRLPIPQDDNVCPMALRRLISSCWCDDPEDR
PSCGHIVEELSRLLKYAPRPLVEENL

>A768|Cre03.g194100.t1.2

MNYQQGPQSYPQQYAPPQPTSEEQAISIALAQSVVDHEAWRHRERELQAAQAHRVVKSSAAEAASQKFWRE
GSLGYGEVVADGFYDIYGDFPEVCEGPNQFPALADLRKVRTGSGDVREVVLIDHEEDQGLLAVEEQLSEAMEEA
KPADVAARIQVVARVVCERFGGAYDTEAALDHFWHASSAAEKRRTRSAVVPLCRLDVGSARHRALLFKVLADA
AKLPCRLVRGAALCGSEAGVVVLVAVPAATGAGGGSRSGGEWVVDLVYEPGRLYGPQEYCALVKSKRTRSDSW
HRNGSASTLAAAVETSAVSSSSASSVATVSTPAAVTGAAGSGEALGPASGNLLTAPPHSVSTASLQSAAAAAAGS
TGSQSTPALSSPRTALPRPLVAVPPVPGPGAFSSSLPNSGGGSSMHGHGQHGGGVWGSGLSVHPQGQHGGG
SGSGGAGTGAGAGGAGPAIAGSRVRFTITDLPKSGSGTPHVSTSITTASSSSPHGHLSTLGAGGHAAPGALHSSG
GSAGATVGSAPHASSHVPHVRIGPVAAGPTGGAGALQHCTAIAPPPGTAAGPGPGSAAAAGSSGVGGPASRH
ANLPHSKARSMLDLHGNSVGGDLIRLDSEPLPPPEVCPTPTGAGHHHPPHVTAAQVPHKQQASLDANGWVKF
SGSFGRAPTPHKDEATGHTPSRLGQQGAPPPSVAPGQLGASLLAAAGANGMPTHPHPQQQPRQPHQPQLPT
HMESSGSLTGMGNPPSPADSAGNGNGNGGSGSGSGAHCNGISSASSGLAAQPTLSAMPPTGLPVVTTGATGL
SSASPFAHFMLPGGMSLSLPGAAVAAGVGAAAGQQGGGGGGISTEATVSSAGSTQGGAAATAAAAAPAQGP
ASGKPPTGAPGAGAGGLPHATVSAASPFQAAQLGLGTGPQQRSSLPQHLASSFPTLHPQHQPLPQLTSQQQQ
QQPNLASPPLAPGQRQQATPTGLPAGFMPHAATTITTGAGGNALYTPPPKDAGTSSKAPSPALSTPLALTASSA
QSTPGNAAPVESIPFADLSPWYNGTNANPAAAGATGGGAAGGRAGAGVGAGRPGQGGLLPPAQASTADPAE
DRASKARSATFFADLSPFNNGVGGPGGGAGGRDNTPPGGHARGAGRQSPIVEMPSSDNSRDTTPRMSFTNV
PDNGHMLLPGGGAGGPSSWSESTVDMMGRLRIGTASGTGMGSRADASVGRLGSHGHHASAGSGGGQLVLH
GQSMQQMHLATTTGQGQTFAPMGQAMGAGTGLGQAAAAAAVAEQQRQREMAITLYQQGMQGGPNSLQ
LISQAMALAQAAGMINLNPTPGAGAMTNLAHMQQMHVMQSPFGAPAGWPHGLGHGHGPQQGQGFGGA
QQPLLLAPQQMGLLGMGPGAMGGGAGAVQGPGTALHPWAAQAWVAANQHQQHQQQQLQQQVPTFQP
HFPPPQPGGGAQSLSQQQHQQQQQRQQSAFMMHSAFMQQQQGMAQQQQLHQHQQQQQQAHMERN
SSFATTTTTTTTTVVRQTTQVVETSAAAVSSAQQQGGVASAAAGAIGLAHEMTIPSASRLAALQAAGAAAAAGG
VSAVSDGGAAGVSSPLGLQPTLSVTSTMPLPPHYKDLEIDPKELTLGQRIGIGSYGEVYKGSWRGTEVAVKRFLE
QNLSPPTIRDFRDEVLIMSKLRHPNIVLFMGAVTQSNQLAIVTQFVARGSLFRLLHRTKEVLDPRRRLNMSLDIAK
GMEYLHNCKPVLVHRDLKSPNLLVDRDWTVKVCDFGLSKVKMDTFLTAKTQGGSPAWMAPEILRSERCDEKS
DVFSFGVILYELVTGREPWEELNPMQVVGVVGFNGQRMDLPPDLDPGVTALITACWADKPADRPSFSQILATL
TTWSELRPTAEVMERQAAAARARQARQQGGGGGGGGGGGA

>A769|Dusal.0459s00011.1

MKKLFGGKHRRNHSCEGAFAGGSGTNLDIVSELPPLETSSAARHRGPANLQPAHLAHLQQASPRGRHSLQHPQ
LQQQQQGQHQQQQQQQQQQQQQQQYHHPVGISPPMMLQQHPHYPPGVPAPVHLNDPAAIEDAHVEEA
KRASLQVFQERLYREHEEEMLIQEREQKWQRAQRAEQEAKLAAYRLSHKYYESGSLEYSEPLVDGFYMLYGDFP
ELEEVVDANTKFPSLDELRRVPLYEGDVREVVFVDADQDSGLVAIQEKASVATCEAAVCANSDIERAFRRITSLAQ
VVVDVLGGSYDSEEMLQQMWQAESSKEKTRSKCIVIHLAHLTAGTCRHRALLFKVLADSLNLMCQLQRSRHSKT
EEERAVNVVQIDKKDYVVDLVYQPGSLEPLNKFVAENPPFSHPGKATRTQSATLGSAVVGAALAPSAAAGGSQ
QPSSAIQSRPSSGTTAPVTPSPSHPSTPAAGQPAAASAAAAAAATVAAASPDAITAAVQDPAQPQQLQPLQQQ
QQQQLQYEQQGQHHLRHGRAESWDELTASRAMNQITTDLISLDEDDAGGAERGSGSGAQGAGWVTFGNNS
FKGGQHLGALLAAAHNRHGSEEQQQQQQQQQQQQQQEGQAKQMPGLHSYQTHMQGGSPCAIPGSSGVA
SSSGGASDSPFANYLAMMSAGMQDNLSQPSNTGSPRAQNSTGLQGQGHRDSMEGSPVHRAHDPGGQGLP
PKLPHQSSSTSSQDARGSGTPTPLSAAGLAQAASASSAFLYHPHHAHHQSITDMSLSGLSLGADASLRPGSLPLQ
RQQLLGGPGLGGSISGSAQSAGADPNARPRSNSWNQQQLCSSAVHHNKRGDEVFADLSPFPIRPPPPVPRLAN
STSMDRLSAARAHPPGATSPGANNSSQQQQRQHLQQQQQQQQQQMNGPFGSNLQPPGGVAGATEGVQA
TASTAAAAAAAAAANSQVIGGASRIPTFMPSSPFSGQPWTSRYPQIASESGVGLQAQASQAQSQSARPCSTALPL
PTHSQSLLVGHPMLIGTSLPPPSFLHPLPMVSSPLQPPLSTSPFVLPTSSQPQLPPLYNPSTQLNTRPFLQSAPAGL

SNNSTNTLPPIQYTPPHSAQPRAGGAGLGPPLPTAPLVNPESAGEQPTANGVAGGAAGAGAQASQDPKRLTV
QPSLQMAGYAWEIDPEDLDYGSGQRLGVGSYGEVYKGMWQGTEVAIKRFLDQNLSDAIRRDFKHEVQLMSRL
RHPNIVLFLGAVIQPTQLAIVSEFVPRGSLFRLLHRSRAHIEPKRLLLMAMDIARGMNYLHSCRPMVVHRDLKSP
NLLVDRDWTIKITDFGLSRVANATVSQTPRSCAGTPEWMAPEILRNEQVDEKSDVFSFGVILWELVTRKEPWEG
LNAMQVVGAVGFNKKRLLIPDDLAPELAQLIKACWEEQPSLRPSFKSILGYKKCSHAKGRDVFLKDIEPFIKWLEE
AKEDADDDDK

>A770|62964

MSTLAIDVERTAQNGVTSSNEPSPVGSMTHSPTGRKSPFTEDRIASIVGKKTLRRESSRGSAGSLAGLGLAGDAS
AATSPYGSAPPSRRESIGDLSAADDNGGWRDDVDASAGDDAASVGGASVSSGMDGGRRRSLGRASGASSVM
SFASTVSAGGGGSRAKPADTNLPGAPGRFLSWLPREPTLEGTLEKQKSEGYFGSISRMFGMTKRGWKMRHFVL
YDNHLFWGRGFSRMYGYGTVLSAKPAPEEGETAFSLELVTHPKFSLRRGGYDSLDYMQRLYGLCCSTHGYSVRV
MRAGTVLDRDRWISSLQRGLPPPDGTTRPNGTNTPTSPIDAIDSQEQSVFGLSTPKMSPRSSPEPPEVVAGGLA
RSPGSSENIAEKVEESGGFSPRVGIKKSPRAKAPATLEEAREQSGLWTPRDTTGAEAEAAAAWEAAAHAAMLSE
AAAAEEELAREAEEEIKAKADAAKRVANRADSRGGGSVSFADALDSVLEDEARAESKTPKATPVPGGGGKPPRP
GKGFPPKKHDSAQSINRSSSSSGSDEDVKDSARGSPEDSTSPADEPDSPEPDYDTADEALSDAGSARTVLGTDDK
TSGGGGAGSTGRRGDSVPAEWLAANTGGVRRNRLKSQVPPVSVLRKTSSIEQKSRHSKGGSLHIADAIGLLGGG
SGSNPEPTDGPGSLRRKVSFREEGEIESTKLYAPTPTKRNPQGVGQTSAKYAPFAARGDGGGVPGTINELDDPSF
DEALAGQFGSFSQQRALQQAAGRWVIPPQELKLGRRIGSGSFGVVYTADWNGTEVALKQMHDKSLSASNVQE
FSGEIRMMQGMRHPNIVLFLGAVIQAPRLSIVCELMPLGSLHALLHGKTQNGVELATNGRLRRQMAQDCARG
MSYLHSRSPPVVHHDLKPANLLVDSHWTLKVSDFGMSRLKHNTYLSSKSPGGTPEWMAPEVLRNDPTDERSD
VYSFAVILWELITLKYPWEELSSPVQIVVQVAFLHRRPKLPTWLPAEAVALLQQCWHKDPDERPAFSAILGALKAE
MPEAWVDQPTESPKAAAFAELARKEQPFSGPISPQRPRRRDRAPSPSGSVNSVNSLASSVNDSLGGSLRSASPSI
EGNFVTLTGLKPIKTPKPAKKKGSSALGPNHMSENGAAAAEEDDDDDDDEDVFKGFPGMGRVKVPGTARSAG
SASANDGLRTPPMSPSRSAPGSSSSGGVKSPDMAAAAGVLPKLSPLKIVRKLPTK

>A771|66339

MSTDIDLAIALSLSEGRTSNFVDTRRDSSIARSIQDEDIAQWQSTLGPGPLPEMHADDRQLRLERTLSERLAESGA
LTQHIPRTNSIEEGPGIADVGEFGAERLRMQERLGRYSLCEREVKGDGNCQFRALSDQLYRAPGYYDQLRRVAV
DELRSHADRYSPYVAEDWGDYLRQMAKSGTWGDHLTLQAIADHFGVKMYIITSYREGEIIQINPIGRLRSERVLY
LSFWAEVHYNSVFPRAEPPPVLPKDKTLGSRKLSQQVLPVGCKYFTYRELEEATGDWAQSNVIGDGGFGRVYKG
RLRNGLLIAAKRLDRHGLQGDKEFNVEVSILSRLHHPGIIKLLGVCVDGDQRIAVFELLSRGSLSSALGNSNDSESA
RSAGGRDPSKQLSHVLTWQQRMQIALGLAQGLSYMHQERLVHRDVTSGNVLLTEGAHARIADLGLAQRLTMP
DSIVIAAPVEADSILMGTYGYVAPEYAMSGELSQKTDVYAFGVVLLEILTAKPAVDGSRPPGCQMLSEWLLPSLSS
VDRIWEHLDPALDIGTVAAPQLATLADVALASLSKNPSDRPRMTDVVRVLETAAAFKPDPSAASPSAANRPALN
LIDAAAAPVGQLSAFGDPFASNPFAVGAPPESNLHAPLCTGMPGATDPGRGHAGGSSAAAAVTPQRGSPVSP
VRHLGHLSSSRAAHALPKAQPEDFASPLQSGLRFGVMSQVSPDVGSAFGRSSATDQAAGSGRSNDREVSSVAS
SPLFDAMASRQSSPAASSGAPQQRDEASQSPSQLGSNTISLLNYPADQFWPHRGGSTAAPEEPSSSDTGPGPP
VGSSFAAAVRRKEWRVAGPPSAPSFGDEPAGFSGGSGTQNRQRQRAGGGGLGGPLADSVASLTMSADRPPG
RQTYSHAPGEIFGALQPIVDEEAAGPPEAAKKGVEGAGLSPSVARRRSSGNPFKDEECSTPPNRSSAERSGSGNP
FAVAGGGDVQGRPGPSDGAQLHLDNSREAEVHWPCAQLDPAFLQLSPKPLAA

>A772|63499

MDRGPGVPPPPRHQLVGGSRGSSSGSLGAHIPGADGSSEDLHALGKREKVKDKVKRYIAKLSFVEMSVMKATT
KIDGPPKEKHTRRLVLESWSHPDTAPAEMVHALSRINMLNSPVVTLKALSTVHELMQRGSPAVLPAVGGWLDH
LSLVQSHWDRNGNNADYGLSEVCSPLVVAYARLLAAKARFHSEHRAFENNYSFDEEAEGRGGGGIRGGGPHPI
SAPALRGMLSVGRTCREALEHCSKILPANPPLITIQWQRLVAHVGKLMAVESHLLHGAAVYVAAMLASSGAGGL
GTSGLSVEDAAALEGEHSALRGAFERARARPAIVSAFVEEGGDPALPSGWSGEDGTGGRGGFRFLELPATLPSFD
TEEGRAAMSSMIASSFGPPSSHLPPLPHEEEDDDDYTCSPFDDYPSDDDVGGESPAPPLKIVDVPDLIDLGDFDD
VAQPGLGPPAAHFVQEWPSPTRLAPAPPRPPLFAGYGGVSNTPAAPAPHGASLTPVSISGKGPARGAWKEHSR
NNSGDKVLDINASLAAFELTATRPQESPKMKLNVREPSVFADIAPAGINSTRPAPPPPVSMNAQRVARPLVNQF
APSPQPQPSPQPQQQQQQPPHVMHQPAMTVPQAPWPVPMQAPQQPKANPQPALQKWNSPPRDWVQW
DDAIADAPSVQTPADTPIVHPLNDALVQPDVAGLLGDLTQGSFDEIPIAAIRFGKRVGTGAFGEVLKATYQGTDV
AVKRLRLDPNQPQAADDFRRELRVLCGLRHRHVVQFLGACTTGPDLCLVMDFCGVGSLYGVLHNRRQSITAAH
VMRWMADTARGMVYLHSRNIIHRDIKSGNLLLDDSGVIKVADFGLARAHGPTSNLLTLVGTYPYMAPELLDSQ
PYNSSVDVYSFGVVMWECLTRDEPFRGFSPMQIVATLLRGERPKLPAQPALPASYVSLLTQCWATEPERRPTFE
VALERLLEIAHAMKAAESRG

>A773|Cre12.g532050.t1.2

MGNSLHKACSEKDLQKVRVLVLQHPGQINQQEKEMDWTPLHIAAFKGSDPIVRELMARGARHDITDKEGRTA
LHLAALNGFSAVVTDLLRRGASPAARDKNGKAPYDYAVQKHPNVAALLGGPPPPATQPAAAAADAGDWGGT
VASPRSAGGAAAAAPQPQPSGRLASGPGGSQHDLFAGAHAPPAAQPGLGYPAAPAFPVSPTAAATGVPPYGY
GYGAGAYGTAPPPNTSVAPPAAGAYAYPQRPQPYQAPTGAAAPPPQPQQQPYYQYPSAPAPGNAGGAAAAA
SHPPPAPPTAPSVPVAPAHSGPPPHGREGGGGGNSSVAPPPAPASDLDWGPPAPSDWGDEPDTAADAPPPR
RPGFNPIMSARFQLEKLLGAGLAQLGIHSGGGAGNGQWGHGGGGGGSAHGSTSASQPLSQQGSGSTLPPTP
GRTAPAYHPPGQPHPHLHPHQAGPSPLGPMAGHGGLLSEGSTTSVSSISMAGRTYSYDDLRAATGGFSPINKL
GEGGYGPVYRGTLDGIPVAVKVMDCTEGAMQGRNEFEAEVRILSGLHHPHVVLLIGSCPDKGILVYELMPNGSL
ETHLFGWEGGRSANAGGARGPVPLGWRHRVRIAAEVASALLFLHSAPTPIVHMDLKPANILLDEHLTAKLGDV
GLARLAPTLGAPSGPAAAAAAAAGGVKSTIKDSRLVGTFEYMDPEYMRTGEYSARSDVYALGMVLLQLLTGREG
AQVVSVVESARRQPLGFGPCIDPRAGDWPAAEAMAFADLALRCVEYRRQDRPDLRTVVLPTLMQLKQRTQLY
EQQQPTAASSPSPLGGDAVPPMFLCPITQDVMEDPVVAADGYTYERLAITEWVSRSPTSPLTNMRLEHTQVVP
NLTLRSAIKEWRQQQQHPRRSGPGAVVGAVVGTGAGAPEGAPSAPPMTSAPPVRARADAGGW

>A774|64721

MGPSTGTPYRCYVNPMTCQHKPFLSNNGQVSAAWDACMDPGMNTTIDAEKTINYCSCMSSYNYTTVNGTVF
KSDGGCIQDDVNIPPWCLVVEKSCSQPPPKKPNGQAYDICRGTEYDIRQASATLPTGPALPRPETPTGCQCLAN
WNYTTPGSNQTSYFNGTCGTPGIKSAGPWCYVDKNTCSHQPIADQSGLYYDNCANVTSNHTMDTSKTVNYCN
CQPVYNYTAADGVAYSVTNGSCIRTTPSELPWCYVVEDTCITPVLHREGLGRNDSAWDTCLTTGESLSYIPPPGV
GSASSASAGVNRKLALAIGIPCAVAFVLLHIPLCWGLYILLQRRRTKKKEEDKKAEGLLERPWGQDVLQAYMDKQ
YGDGSKDDEHGSGGKIVAGMPPANGSTAHSNGTNGTNGSSETVGLISGRKASRQLSAASSLPEVVPSSSWEIKP
SEIVICKHPDGSDWEIGSGGFGKVYKAQWNDYQQVAVKQLKQHDARNDIRFLREIAILKECRSTNVVQFLGACV
APGSTMLVCELMEGGSVSDLIRSGQLVWSEGGLDIGLDVARGLSYLHNNRIAHLDIKSGNVLLTREGKAKIADVG
LAQMVQNTHISNLNGMGTFAYAAPELLTGGKCNEHADIFSFGVLLWELVTGEIPRRGKLRELRVGEECTQEVAD
LLDICMRTNVEERPSAKELVAVLQALPPQPNNRPKRAASAQSFVSPLDRAAHSDDSSSTLGHSTNSSKSASGFNF
YQGNTAGYSDSMPSSGTLPLIREQDTSPFVNGGTASHVTEVHTDELPVSSDPSAPVEKNPATDALEAAKALEAA
EQEEAARQEEAAPATEPKA

>A775|OUS43630.1

MGRTKQRGKTLTLGEFFAETSTQRTSGLLGARLARAVDGATDGGDDGDDDSYAEFTVEIGNASVRCAEQEGSY
DVRAFAREGRNAGGKVIKSVTFVFGGKMGERRVTEAPFEVQYRCETSVDVEVAVQFHRALNARPVRELHAIELS
SEEREFSRSFAVEVKRRALMKVLGKDKGDAEVDVRRAESRSSLLEESQSRGVDAWTVQDVSEWLRSIELEELVER
FAKAKINGYELLRLTEKDLRESLHLERNLERVRAIRAINVLRASAGAADAENDDKSATPPLSAPLGAPRGGLSPLEL
ELDVSWIEFVSEKARASVLIGWFMHVLDEVKAKEFDEPGPQLSVYCEATLQASKSCEDALEERVLDILESTPGWD
PRTKMFPSTCDLTKLNDQLMELYLEVKAFEEFAALNMDDDRGEHERSRSTPPKMPGESSPAIPRLNRAESLTTPS
VSGSPTFTSPRNDMGPSPSPSGLNVGARSFSPTPLSDLTRSSLPELQEDEVADGDRVMELNTEWEIDYNDIEFEG
GVPSSKNRIGHGGFGEVFLGRYHGSLVAVKKLFNQDMMGKGLQDFRREVRILSRLRHPSIVLWLGACTQAPNL
TIVLEYMEKGSLHQFLHRTTNPYTTLTLTRWAITIVQGMVYLHSAKPFPIVHCDLNTNNVLVNRDGMVKITDFGL
SKVKHSSRLSRQTGMTGTVNYAAPEVIRGGKFSEASDVFSFGVVMWELLTRRIPWEDLNEYQIVFQMTSELDAP
LAATAKNLELPSSSPEGFRKIIHGCWATQPERRSGFKDVLVHLREEYRVLVEKEKALRASRQGSSSSLSGAPPQ

>A776|58573

MPDLEEMGAEGSPAMTSHAGMETTFAEGSGGTASPSMLAMASLEVNDRLQHIDELVEKGVDHVQSPLKRRG
TPPRSFWVEEPDAHSGGGLGLGGLERQGSLRRQGSGGAGPSSNRSARSHGSDDIVLPGNAGAARGGGELWRP
DGLASPPGFASKFAKKPREEAVSADGKGVQDPNHVDAQMDAWLRQTVERMPIKPDHVSGVRGPLMNVTAV
DDKTVDVVMTTSFRAHFSQPISQLGAGAGGGGNRLELPAPGSVGDGAGAGAGRIREIGDGGSRESTRSAVPTT
PLGERAQLSLRAGAYPGSTGRFAIASPDDYVQSGGSGGSGGGSPKSYEQNRALVAGGDVPLLNTMREWEIAPA
DVQLHERVAVGGFAEVFRGTWNGTIVAVKQLLERGQDVVTRLREEAVVLSRLRHPNLLLFMGWCADPPFIATE
FMRRGSLHNILRRNGAPLGGPRTHHVALSVARGMQYLHSRSPPILHLDLKSPNILVDDKWRVKIADFGLSRVRR
NTLLSGRSNIHGTFEWMAPEMLRAENFDEKADVYSYGVVLWELLSAPLTPWNELINVQVVAVVGYDRQRLVLG
LAAEEAAREDAATRTIGELFWACAGNDPRGRPTFQKVLERLEAALTLMLPGPDGTPGAAGTTPKGTTTLEVTAG
LGRVAGEGAAGPPANRGAWRGASQIETRADEDKNIAKGPTGLVIEEIAGERGEGAAKDEDESGVPYVVDEDGH

>A777|56536

MPDLEELDDSPPPPEPRFVERAPDGPDALPSGGDGASSGNVVGVASSPHGLDMGKRLTLSDELRRLGSRRDGD
RSPASGHDRDRDGVDWWVDEPSTSRSADARSGAGGGDPSSGKRYSGSVDEDDDGMIVIHGGGGRRDGDGD
AWTPHETTKKPRPPREGRTGTGIAGVVGEDDVGADALDAQMDAWLRRTVDAMPVKPDHISGVRRPLMNVS
QVDDTTVDVTMTTSFRAHFSQPISSFANANDARRGTRALPPAEGDGGGVLGEGAVVAVGGSNGMVPGSSGR
FGIASPDDYVGGKGGGESGGGARGGYMELPDIEPTVSLGAAKEEEAEANALVSAKDVALDNAVREWEVRPSEL
RLRERLAVGGFAEVFRGTWNGTTVAVKQLLQRGPDVVARLREEVHVLSRLRHPNLLLFMGWCPEPPLIATEFM
KRGSLHNILRKNKGPLDGPRMHHCALSVARGMHYLHSRSPPILHLDLKSPNILVDDKWRVKIADFGLARVRSNTL
LSGNSAFHGTPEWMAPEMLRAENYDEKADVYSYGVVLWELLAAQTPWNELHPMQVVAVVGYSERRLALTPD
AEATARSDPATAVIGDLFHACASKLATERPLFAEVLDRLERVLTLMLPGPSATGTDATGAGAGAGAGATRAAGD
AARDLAKAKAAAVRAALEAAADSPTAMKAEDAELGGGELGGAKPAYVVTEHGDE

>A778|OUS47961.1

MSPVVDGFDADASANARDASTVPVVADAASPTFADFLSPEAQAFSSEHAGESVPVVRVRGTARSGSKLWGIAR
AAVREDGHASRDAAAAEGSRRRRRASAEYESFLRPILDRMPVKPERVSGVDGPLMNVTRIDDKTLDVTMTTSFR
AHFGQSIQAIGRRSPALALPEADGARADVQREGKWGDAGGDGVQISEIDSGEFVPGRGGRYSVMTPEDSWNV
AVGDRRAPSPAIGWHDEEGSSSDALHTEFLINPDDVRLQERIAVGGFAEVFRGTWQGTVVAVKQLLERTSEVKE
KLEQEVQVLAKLRHPNLLLFMGYCVDPPLICTEFMRRGSLHTILKAGKPLEPARNHAIALAVARGMSYLHSRSPPI
LHLDLKSPNILVDEKWRVKIADFGLARMRQTTQMSAKSQFHGTPEWMAPEMLRAEDYDEHADSYSYGVVLW

ELITAHKPWEDLHPMQIVAVVGYSGRSLELPSEGFPESSHPLTALLADIFTRCARRDPSARPLFPAILTDLERARDL ARARAEAAGADKTAARIPPRPDTESVDRRALGVQLEYISLADAAESTDASS

>A779|OUS47966.1

MMRQRETKEAVARRTLAEFETQIEETARRNGDATTRARVERMLKRLPKQYAMDVNFIEDVLAHAELLGRVEQE LQAMGVSSVYCSVREVEVGRAAFGSHEDMMDTDGGIEQLNVTADNGIPIRADSPAGKPRGPTFGSSLQMTSSL GGDPSRGSAGMYEVAVAAGNKPRLLSRVSAVLFDVGLNIAEAHVFCTDDGLALDIFVVTGWKRGEEAAVGHAV QTALDAADFSDIVPASRNASAATPSADEGRMSAGSHGRSTSNDSVSIDGGEWELKESQLVFNEKIASGAFGLLY RGSYCGQEVAIKVLKSNAQEGNAGNETMREFAQELSILRRVHHKHIIQLIGALTKQKTMCLVTEFMHGGNVLQF VQEHALKLHEIIRFSLGVAMGLDYLHKINIIHRDIKTANLLLDENSVVKIADFGVARLQPTDGSTMTAETGTYRW MAPEVIAHGFYNEKADVYSYGIMVWELESGGEVPYPGYTPLQAAVGVVQRGLRPAISTSCNPKLAQVMQSCW LADATQRPGFEQIISLLKSIDTQKAETDGKHGFFDRLRSVSFKSKKNAPARSS

>A780|RMZ52337.1

MVQVTGTMNSGSSNSDEQLIDRRLTIRWGPTEEELIERQDTFNGSVPDETAAEIASLHGNSAAQRRAITELLFFA CVGDLKRCQRIVRLWNLKVAAPDCCDYDKRTPLHLAASEGAYSVTEWLLAEGVNVNALDRFNRTPLEDAVRGD FNLVASLLMKAGGKVHASGKLIPLEDSELVNGLGFRAPQQKASGFALEWELDPSTIHIREKLGEGEFGVVHRAK WFGTVVAAKVLKASSEIAVNDFRAEIEILQRVHHPNCVQFLGACTAKEPYILVTELMSGGSLADAFRMPQAFPM RRALEIALDAARGLAYLHNRKPTPVIHRDLKPGNLMLSGSQYQDRAQVVFNTGVVKLADFGLSKTLPTNRHANF HLDERFKLTGETGSYRYMAPEVFRHEPYNSRVDVYSFSMIVYQLFEYQPPYADMDPVEAARLAALENARPNFITL AQPGPHKKELRELIERCWAPNADERPSFPEICRIIETLLAQIPRQEYQSSGAVGGDAGCCVVA

>A781|26422

MEEMQIQKQIGEGSFGKVYLAKWKETTVAVKILTSTSGSSDDDFPTRLPNPLLQSLEKEAGMMAAMRHPNVVL YLGVCLDPPCVVTEYCARGSLNDVLKRALYNSKYAEQLDWRVRLSMALDAAKGMNYLHTSDPPVIHRDLKSPNL LVDKHWRVKVCDFNLSRVMEESSILSSMAATNPRWLAPEILAGRGYTFSSDIYSFGIILWEFMTWRVPWHEYGP WQVRERKGSSLHKMLVHAVHDPGYLEGYCWCVQNATERPSFAEIIQVLRRLLADEARRVPNKSPGDAAAARS QQASTSTSSADTARPDQGSYREGANGAQGSGDLAVGGLRFHMAHSSSGDLRNGAQPHVPGSPGPSGRRDEP QHARSESLGSDTHLQALAGQLGSGSQFVPDGLRVGESPRSRATYSNEQYGVPGYRDGGGGAAGGPLMRREGA DGSGQLSSNGDAQWDLARIHALLKEAHSDTSSNP

>A782|108286

MRLLERSAVEKPGGVLCESRGIRVSTVLGDMSNSTENMEVALSPGATKRLRAPTFGSSFNLHTLEEHLGGEAGAS RAVYEIAVSGLNRPRMLSRVSTALFDIGLNISEAHVFCTDDGYALDVFVVTGWRADDEAAMNEKLQRRLDQVN WDEGVKPGTSDGATMGEGEKALAGASDSEWEIQEVQLNFMEKIASGAFGVLYRGSYCGQEVAIKVLKTGGKSS QEEVYREFAQELSILRKVRHKNIVQLIGAMTKPPRLCLVTEFMKGGSALQYLHQRAPLKLNQLLKLSSGVALGMD YLHKVNVIHRDLKTANLLMDENEVVKVADFGVARVKATDGKAMTAETGTYRWMAPEVISHQKYDHKCDVFSF GILMWELVSGGDIPYPGYTPLQAAVGVVQRGLRPTVPPLCHPVLSQVMQYCWQPDPWARPEFEQIVELLKHT DSQTETVPNKGFFSKLRKSMTTSSKG

>A783|Dusal.0140s00004.1

MPGAAAVQAFKRAGSRAAGLEVGPCNIPSSEWELNGMHVQYGKRIAVGGFAEVFAGKYLGTLVAIKKLLATDP DAVRHFAHEVRVLARLRHPNLILFIGYCLRPEPAILYEFMPRGSLFNIMRQVCFHCSGFQGFRVQGCVARGMAYL HSRNPPILHLDLKSPNILVDAQWRIKVCDFGLSAVRRQAFLSSACAGGTPEWMAPEMLRCEDYDEKADVWSFG

VVLWELLTGEVPFVDLSPMQVVGMVGFKRQSLSPPGQGDTTLQQLCVMCMTQDPAERPSFTAILDCMDKAY
HHGSSSGIGSMPNACQTNGDGPQHQAQDGHAARQAPRLKLIEAGAASSAGGGQQCGGGVEKARVQEAVAA
CAQGAPHAASGSSVQDPAQQQPHAVHGTLRPASSSKTPHQNPGESSQPSKSRRLDKGHRSPAGRRLDEGAAL
RHAGTAVNHGQTDQDYLSPGKHEKDAAIL

>A784|35653

MTDASSEQLRRKTKQQVCEAFLQKLREKKSIDLDAPGVVEGIRQHFQTLPTRYALDVNINSYDIINHQRLLNSARA
DPSAVSFQVRTVDVSLPRPAFGSSPNLQVRALDATFYEITIASVDQPRLLCRLSESLGDLNLNICEAHAFNTTDRFS
LDVFVVNGWSGEPDDWELDPTDIVFEEKIASGAFGDLYKGTYCGQEVAIKILRNVHTDSQQYQEFLQEVAIMRK
VRHKNVVQFIGACTRKPNLCIVFEFMSGGSIYDYMRKAGQLKLSLVLKIGTEVCRGMDYLHKRKIVHRDLKAANL
LMDETGTVKIADFGVARVINTTGVMTAETGTYRWMAPEVIEHNPYREKADVFSYAITMWELLTGRVPYEEMTP
LQAAVGVVQKGLRPVIPPNCPEGLASVMRDCWQRDSKQRPSFELLKVRLS

>A785|45682

MYEIAVSAGNKPRLLSRVSAVLFDVGLNIAEAHVFCTDDGYALDIFIVTGWRQGDAASVQSAVQTALDAADFSD
LPASSKGTNAITSSQGSEGRMSNPSGDRSNSDSISIDGGEWELTEKQLVFNEKIASGAFGLLYRGSYCGQEVAIKV
LKSNAAEGSGAETLREFAQELNILRRVHHKNIIQLIGALTKQKTMCLVTEFMHGGNLLQYVQEHALKLPELIRYSL
GVAMGLDYLHKINIIHRDIKTANLLLDENNAVKIADFGVARIQPTDGSTMTAETGTYRWMAPEVIAHQFYNEKA
DVYSYGIMVWELVSGGEVPYPGYTPLQAAVGVVQRGLRPTIAPSCHAVIAQVMQYCWLVDPNARPGFEQIISL
LKHVDVPREQEGKHGFFDRLRSVSFKSKKKEAQTRSG

>A786|Dusal.0308s00019.1

MKPAASAAGDLRVFSAADLADATSGFSPLFLIGEGGFGKVFRAMVHLTPVAIKVLDHEGLQGLREFQNEMTILA
GLQHPHIVRLLGYTAEGPGESSVPSKDGEGIQALVYELMARGSLDEHLASKNTATSLGWFTRIKIAAQTACALAYL
HNNGIIHRDIKPANVFLDADFNAKLGDIGLAAMDRLYGGPGAHRWQNLDTTGQGSSVGQEAIGTWQYLAPEY
RTQSHSSVQTDTYALGLTLLQLVTGASGPKDLVHLAQAALEQATLKSKFLDASAGDWDLQAGERMVKLALWCC
MHNPSQRPSCATVFTSLKALLERLCSMQP

>A787|14113

MRGGGSAGADSGAAARASSSGGGGSSAVSAAEDAAEWEIDASEIELGPRIGIGSYGEVFRGSWRHTDVAVKRF
LEQDLSPQLMAEFRAEVALMQRLKHPNVVLFMGACTQPPNLSIVTSFMPRGSLFRILHRTPNFVLDDRRRINIAL
DVARGMNYLHSCRPPIVHRDLKSPNLLVDKDYTTKVCDFGLSRVRRSTWLSSKSQAGTPEWTAPEQSYNEKSDV
YSYGVVLWELFTGQVPWHDMSAMQVVGAVGWGNMRLELPEAMHSTIASLIRRTWADPAERPNFSEIIDTLKP
LQHAMAVSGGSTSLPVVRDPQTAAAP

>A788|50798

MELNTEWEIDYKDIEFEGGVPSSQNRIGHGGFGEVFLGRYHGSLVAVKKLFNQDMMGKGLSDFRREVQILSRLR
HPSIVLWLGACTQAPNLTIVLEYMDKGSLHQFLHRTTTPYTTLTLTRWAMTIAQGMVYLHSAKPFPIVHCDLNT
NNVLVNRDGMVKITDFGLSKVKHSSRLSRQTGMTGTVNYASPEVIRGGKFSEASDVFAYGVILWELLTRRIPWE
DLNEYQIVFQMTSDLDASLAATAKNLELPASAPEGYRKIIHGAWATQPERRSAFKDVLGDLREVYREQVDIEKAL
RAARKGSTSSLSATSAPDK

>A789|Vocar.0006s0358.1

MRTMAQSGDFPEYGVAELYAATGGFHKLCLIGEGGFGKVYRAMINYTPVAIKVLDPQGLQGIAEYKNEIQLARSI
HHPHIVRLLGFTGAVEAAAGGGGGGSDGGTQCLVYELLTNGNLEDRLLRRTAPTPALLWPVRVRIAAQISDALA
YLHSLGIIHRDIKPANMFLDANMDAKLGDIGLAALDGWRAGASRARDDSAVGTWAYLAPEYKTAGVISPATDT
WAMGLCLLQLVLGKDPRDIVRQVQDVLDKCTLPEVVDSTAGSWDMKVAERLVKLGLWCCMHDARQRPAVS
AVAQELVRMVVALQKQGLLEPTAT

>A790|Cre12.g517600.t1.2

MAGRFQEQAEYGVAELYAATGGFHKLCLIGEGGFGKVYRAMINYTPVAIKVLDKQGLQGMAEFLNEVRLARSI
QHPHVVRLLGFTGDAAKGASRGSEGNGTQCLVYELLTNGNLEDRLLRRMASTPPLLWPTRVKVAAQIADALQY
LHSLGIVHRDIKPANMFLDCNMDAKLGDIGLASLDGWRAGASRAADENAVGTWAYLAPEYKTEGRSSPSTDA
WALGLCLLQLVLGRDPKDIIRAVQQALEECKLPQVVDASAGAWDMKVAERMLKLGLWCCMHDARQRPAVAT
VHQELARLAGTLQSQGLLE

>A791|6189

FEEIPLDHLEFGRQIGRGAFGEVFRGKYRGTDVAIKRLCVLSDVSDERGLAEFKRELSFLTRLRHRHIVQFIGASTAP
PNLCIIMDYCDKGSLYAYLHNPNKTLSAFKVLKWMSEAAKGLVYLHASDIIHRDVKSGNLFIDDGGSIKLGDFGLS
KFHTGASTSGGMMSLVGTYQFMAPELLEGQPRYTTAVDVYSFAVVMWECLTREDPFSGLSPMQIVAALLRGE
RPSLDDASKFAVRLPEEYIAVIARCWRADALERPTMEDIAPELERM

>A792|PNW79200.1

MTRGEQSEEQDLVPVAPEVEHRLAPLVAGPELASAMIKDTVKYLGIQPTGVSGLRGPYLQVRPAGEGASDIFVM
GSWRTHHKPGAGPSGAVLDSMQRADVARPPQLVQLHAAASGAGEDASMQQAERGAAAGVAVAAGGGGA
TGGGRPDAPNGAGAGALPHPPAMSNGGVRGMHAAGPDASFVPRVVEHHDGEDYMQHDHGHAARVRIME
HTGPTSPMATEAGRGAAAAGSVHGNGHGGYGAGGQRRDGPLIEECLSDTEPPASPSGRKATASQHHDGAAA
TAGVGAGLLGHGGGGLTPMDSSPRPPATAARQRPQPIVQLADSLPDPDRMMEDSWPQQHQAVEQPVSYPD
ERLGPGQQPHHVEEPRSTDAQEERQAASGHVVELAGPALPAAHALATAAAAAAAGTSRHDAGASPLTHMAPP
ASVKAAAPPPTANGMVGASGAGPLVLSPRPRSEWELDPSKIIIGRRLAVGGFGEVFVAKYEGTLVAVKRLLATDS
DTTQRFIDEVHMLARLRHPNLLLFMGYTLTPEPSIVTEFMSRGSLFHILRQAGDKVPEARMQRVVAVSVARGM
AYLHSRSPPILHLDLKSPNVLVDDRWRVKIADFGLSRVRQRTYVSSGAAAGSPEWMAPEVLRCDHYAEAADVYS
YGVVLWELLTGKAPWADLNAMQVVGAVGFARRSLPDPTEGDPLLLHLCKACRAYEPSQRPSFSQIVEAMDSHY
GPHALANGASGGPQAGGAVAGHNPAALPPQRQEPPALMPPPPPPPLETEAAAVPIVQETHSPSPAPLQSTVPP
PAASPAPVPGQAQAQAGGASASLRITRRHSLAASPVPAAAAAVASSAAAPATAPDADGAAGPARQDAARFTS
VHDSPRSRRRYRSPHGEDAMMGPPPVTARLNDDRKTPSPRTALGAAGAAGRLWAAHGLASQSFADNDGGM
HRPQAAAAAAAAAATAGAVAPRHNAALTAASPFANASASGAASQAASPSPFAAANTAAVAVAAPAAEPPARL
PASASPFAAAAAAADAHDDGAKAQASPPVSVVDVRQLAAPPAVAVEAGAGARAGPEAAGPAVAVAVDAAAP
AEACALEGPDSMDLGSPRPRLVTSPREGWGAETPKEAAPHPAQGPELAAAAAATAPEAAEVQAKEEVQEQQP
AQQAQLQQQEERRPLVVQPRPSSGATAHSLRPPQPPVGSGAQGNPSVLRQVLAGRYGRPGHRRVASDEVGQ
LQRLNAALAMAAAGGAGVTGLGGSGPGSAAPPHGAAAAAAAAAAAAIMQVHQQHRLHAAAAAAAAAAAT
PGSGQASGGGIGSGSGGGVGARRSRGLARNSAPMRYTYGGAGSVGAPTAAGAMQPPSALPQPRAHHSHGH
LLHQQMRAAAAAAAAAAAAAAAAAASSSAPSTPAANAGAGMSGGGVGVSAPVGLSPMFDVTSAAAAAAAAAA
AAAGGGSAASSLDLHDLAGLAAAAAAAGDRWAAGSADTQSELSFAFILGDRGDGGSICSLDTADDYQDYMAS
ASLDQLPLLEEDERMDSQEPIEEEGEISLDEDDEAAEPGAGSKSGSGGGAQSEGGGSAAEGERPHLPHLQLPHL
QQQLQQQTDVDLDADFELPKPPPEAEGPAVELIPRPPSPFVAPSAQVPWGSSAAGNAGAAAPEGAIAASAAPH
QQPQQPQQPQQQEPEADLWAALQRARSSRQDSAAAASELRPVAIAHASSHVPDHHQQQQHPHQSSSGAVA

PPSPFNTYSNQQQQQSAPHGGQQFAFAGDASQHQHLHTQVSHQQQPQSLLQHGTNKGHGHAHVHFAPLP
GAHSHAELHGSQQQQAGHSYSNNSFQQQQQHVHVHVHSAAGRPAEGSAAAATATAGPDATAAAGQPSRTL
SRNASGGGSGAAGASATTYRSGRFKVTIEPTAGDAPSHAEHAASHAHATSPAAHSHPHQYPMVGARAQPHH
QHAQPGTGPSAAQSPATPLSSMIVGMQGPPSPGSEAASPTGMAAAQPRPDVDWIFPSAFALAPTAAGAGAG
AGASPASGQPPQSRTASGEVAHMAGSHAGHSHSQHHLQQQQHQQPPPLSPAGSTGAGPLPHALSVEGAGD
ATAAAAAAAVARSRSTPQHHTAVAAAGSLGTASAGSAGGSPLHLGDQHHHTSSAAAAAAAALSGGGPPVALN
SVTMYRKGRFFVSSHSAAGTHSLSVTNPFASSAATSSHAGGAGHGGGGEYGGAHDDGSEEHDGASGPASPRL
GGGLAAPAAAAGGGAALFRRVSSHAGSDGSSPGAAGGDGGAAAAAVAAGGGKMSWAAVAALPPSPGAAGS
SPSQQAPPIIAAGRQYSRGRFTVAESIVAAPPLPEGRRPSMSFPVATASAAGELPSAPSGGLGASATAGAGADLA
SAPPSMNGVGNGVGPAPAQPVPVPGRAAGAGAAGVVVGSAPLWAPMAMSVSPPSAFAVLAAAPASGPGSA
LPSRTGSGIMGPPPPPILASSVGGMGMGSGSGLGLGPPSPQQPQVTVVRKMGRFTVREMDSTSNASSRGPSIS
GAVNDAAPGGSLFVPPPPSLPPGSSPAGAPTAGAPAGRSGPSTAATPSAHGAAVASGAAAGAGAGGLMMLG
SPPDHEAMLLSSHHSSCSFASASGCSRTSSASASQALEVDMGAAAGAAPGGAAAGGAPHQQHHRQLERRPSG
GSPAAHGASAGTLSQPPLAGSPTPGGAAGALPAASPPSLDATAPRNRSSEGGTPPGSAAGMDMGKVLRVKSK
GRFTVIEHEVAPPPALTQQQQQQQQPQQPQQPQEQQQHSSLEQELHERQSHLTQAQHDQLMMLPPRQQS
VGPAHGAAPAATAHPAQDEQQQQQQHGIGAAAAAGLVEAGRDHVAEGPGGHGDPFPAVLETVSLPLPPVAR
AGSLPRAPSGSAGAGASATDLPGAQPHQQPQRQRLASPPPPARQAPTMAAAARGAATGAAAPPPATWGLSG
PQAPETAAQLHADMAQMVRERMQRNASMGHLQQEQAAAAVQAVQREREEQARARVAGGAGGAARDED
EEEEQGRGRGQRPEQQPQPQQQGPGRRRPGRRGQVGPGGEEDRNREDEDDEDGAPGGSGGGRRARRGLR
AGGAGSGAGGAGAERCTYEVSFGDGLTIQISHELPAGAGAGDDDGDDAVMLDVGIVHGGGDEEEDDEAVEQ
EEEGTDSSSEGEDAEDGGEQGPLFPPGTPVHEGTVVHGWDDGTAGDAFSPRRRGWRGLYGARSSDETGDGA
GAGGGTGAGEGDDADSMEM

>A793|0044s0024

MHEGRHVILQLYAATIGFYLVTGAAAQFQGSVSCPQASGFTFLRLHGSLERNLGFVTRPANTSASSWISYLAFVC
GGLDSCTGFSTLGWLKAEVGTFGTLGQWYALGSWDLKEDPTGMCAGFYIRADKPRDGCPQVSGYFLYENTDV
SGGELVLRNRINAPILEVPTADIVAKYGEECDTYPNCIAVTSFGVLLGSLPTYKNLTYLPQTVSELYGQCVGLYIRIP
QPMDNVGGFRSLVRANSSMELPVIDSSALRQLQPDFKIPSDWNSAVNYIELSFEGVYLPIGSYGTEVGTMELAQ
WSRNNRNDVKLWLGGPDGSRVARVTWYLTNVELEAGGVARFRIQTARCGWSALRYRIVLTYRMNALPQLPGP
VKPPPDTPPVPGAPPPWPRSTLSPPQLPYSHLSSTTGNIPITLPPKDGDRNTTLISLTLPVGWSFTGARIVIEYTGAY
VALPNASLGRSYGSGFGPMGQFVLIEARKVPPSLTPAMLRPPRALPPSMPRLPSPPSPPPSPPSPPGPPPSPPAP
PRAPQPPTSPPAFERQGSPPTRPESPEPPGSPWPEDVPDSYPGWRRRLLQAPWASPLSLTAAQQIDSIIETSYTL
PIESDRNGFDAAPRTSLYFIGLPLAAGSQVTLAVRTVNGGWNQLQASLLVEWQQPSSMTVTLPDMPSKPLLPLP
PSPRPPSPRPPHPPLPPSSPTLPPHPPSTPPRPPFLPSPPRPPPWPPSIPQPPAAPVLPPWPPGITGRPDRAGPYL
GPPPPVAPSLGASGSASGISKALVIGVPVAISGVGLCMFAAAMIFVVAARRRVMPRSLRLSELYNYHWRNKRSSS
NSAAQLGGVHGGGGSNSSPCGDETAAEATTAERNGGADKTGGLAQNGGRKPPSLVCPVSPSSPPQPNRGSAS
GFSPQGNLLRGTWQGDLLPLTSFFPAAAPQVNASFAARTPQPGGAGALTGRATRGSESGSSPASLLPHARSRSS
PSVPHDAPHGLHMPSPFEKNAIALADAPEGKDGSNQLATTVSCSEALHGENHTSGTYDKAGWGSSPAVLAKEE
QRRRSWTPKQPEAKGTSYTDSNSTFARGQPDCCRQLPLQQPTKQHQQRQYQSCIDSEGAQRQATPKHPVMT
RPNLSVNISYVSPTVITDISSASTDPRRAGMTSSVGSLPAAAMADNFTPALASSSLSLPANTDTEGARGALTAAGT
NLDPVLISCLRSHGTRAGQQRARTSTRRSLNGTSSADGAGCLSGHDSGQQPDVESNDEDSPHVQDTASSGGRG
DVIRSAAAANASQWPPASALPPPYPHSQPRDLQLPVQSPLRHHARTRRSSWCGYPPPSRLVHRGASVELGLELQ
PASRKGHPSVGLALPPIDLSSLEGVYRLIRAGAAALPIMPSAGGSSAAELPQRLSRTLSDSYMFTASGQAVAVAAV
AAAAAAAVATTAARKRRSRGGIGRVSGRRSNELDPALLETPLKSRKQPVPYSSPPPQRRLVGAIPGFQSAAEAAT

AAAATNGGPYAHAFSAAAFAASTGACQMPQDYTLNRSRSCMLPGPESKSLKSLEDQMLPVLPSAHLLLPPHPH
RGRRRRASAPLLPSFPSVPHNSPEDNDTRAEVVERSTPCVKHSMPKRISFDEKAPASRSMNLDQSRGGCEALLRS
AALRLEAHGSATDVVVSVAPAASVPLNAVAVAGAKMPVNASVSYTRPGPPPPSNASPRSPSVLDDGGPFAAAA
KFAEVYGSSSPTAALAPSHQLASPYPSIPAASSNASPAGGAPDRPQGPQSRSPPQPLAISTPSLPYRRPRYATELQ
TILSGALDDCSGGPPQPQAARRLDPSLKAASLPVPAVPGVALQHRQPSDNVLTTATASTLGPLEPLPNSSSIRSVR
GSVSAGGAAPAATSATAVIARLAGTTSTTNPSSGSVSPQASGTCSGQSLPPAGIERSHEGNESLPTQWLPSFMA
RSQSYSARQATRMGMSTSLARNDSGVNPARSTASQLQRASGPFAIPAAAAVASGGIGNGSPSLIGSSSTSSMPG
SRPGSGSLGFQLARRNQPMASMLSRAASFALRRLQASGQAQLSLQAVAPSPSGSEPLGNSPSQVGAAVSPAVS
PVAAGMAHYTSATGSPLNASTALSSAVATVTASDQSGPDHNTSWATSTMHSMTDKSATTMPTYSGMGSLLV
NTTLDMPYGQPSNSKAQPQPQQQLQQLQEQQQQALQQQQLGGEHPVSAVAGADGDQFKRTSPTETHPQV
PQRQQQLQKQVTPKWQPPSLPLPQEDSPTHGGSNSTLNGPGRDFATASPIVRGTPRKAEAPNTHSIGKVLPEP
ALAVSQARTAADVSGSGPMSPFNDDTAAAAAWAAAAAVAMGLDGTPVKDSAESSSAEKPHQVLSPRDGVDS
WSGSGGSRTPWARSRSNLQRSTGPSPRPMRSGQQLSQQQQPQARLQLPLRQKTADLDQQQQAAQQMHPQ
VVQQGQQQQAVQQPSVAQEQMLLVPARSVDMDLDPNEIHIFRDGLLGQGAYGAVYRGVYRQDMVAVKLIN
GSIVDGRALQRDMESFHAELSILSRLRHKNIVRVYGGCLRPPCIFLVMQLMQQSLDSVIHHGQRHLTLRRALQLA
RDVAAGLSYLHPTIVHRDLKPANILIDESGTAKISDFGLARYKYKAYLSTRTPDQGSVAYMAPECFNTDIGGLGPKT
DIYSFGVLLWELLSGEYPWMGESNVQIIYKVAIKGERLPLPPADGSPFIATSLTGAPSYRSTASADSTQALIVPAAIR
DLMNACFSHQPNERPDTHTAVCILDTIMMELDSGRLVVDQPVGGSQGDYGRDSPGSSDSPVSGSGGARDGV
AGENSGVEGSVRMAGGGGSGGSDQGPAAAGSSLISSKDGSARRPSNNIHSSRVGDSGAVGDGGYASNGEGG
RVVNQGAGQASATESGKVPGVAPCIPEPRGSSVAEGECLMAPGNQQQLATPFSNLDFLFD

>A794|PRW32999.1

MRRKLLEELAGPASAAQLAAEAQSLSYWQSDALGYTDLVLDGFYEVHGDFPEVCGHSEFPRLAELLRVVPRTDA
DPGLQRREVVLVDRRYDKQLAAMRKEAIEAVARARVTSSRSSDGGGAGPLGSPGSMLGSPGGVGSPSRSRRSD
SPDTAALAGGFDTLPCFQALALVVAGHMGGSLDSQPTAAEQWCAMINRLKVQTASVVVPIGLLAMGAARHRA
LLFKILADELRMPCRILKGRRLPGNESDNAGVVVTCSNRELYIDLLARPGEATPHALHLAAEAAMGGDSLLVGGG
SAIPAAPSSSSGGGSSARERGGAGSSGGPPSSAGPGSRASTSRQASVVRWDSQMLHIEAGLARAGSSALSARSR
HSSGHLPEPSLADGVPHAERLLEVAEEGWQEAEPFQAEAAEAAAAAAALASGDELASPFGSAAVTYGAGPSPF
GAAAAVYDDAAASPFGAASAAAYEEASPFGAAAGALPFDDDEEEEAAASAGGDADQGITLPFANSAAPANDA
VRRSGTSDPGAASTSRRCGSAALEALAELRAQQQEDGPPGQDWQFSGVTLKPAASPFEAQAAAAAAMAAQP
PSQQAAMQAAAQAAALQAAAQQAGMQAGAAVVAPGGIDATTPFLAMQGPLLRQTSPQGLPGNHSPVTRH
LSGGGLSLELGPAHLEVDEKTGQAFLVQPVRAASDMSGTGGLRSQVSGSSSGGPLSNEGSLMELVDALRQSGLV
DGGSLPLRPNGHSPLYRSAAASGAGSHASHLRFVGMRAKSEPPPPHAELYAEEAEGGLLQQAAGGSCNIFSSSG
GLGGALPSLGPTLSAAGPSLSFTGLAADAFNPRHAAQGAEAGAAGEPQMQATASGSTFNACLGAVGGLGLPIS
PYENRRSVEFFPAPSPGPTRLSVPIEWERLQAIRQQVAASANGGSGGGTSTPPSPDLTMVPARRSLSSGDPVAIP
FGGSIPEGGPFTPTVVPASSLPSPFANSLQAVSAGGMLSLGAMQQQQAAAAAAAGLYPGVYPQAGRQSDNSS
LTGSTADATSSMHSTTSDGQHAAAMAGVMLEGLEEWEIQPDEIVLGPRIGIGSFGEVYRGIWRQTDVAVKRLL
DQEVSQQMLAEFRQEISIMKRLRHPHIVQFLGAVTQPPHLCIVTQFVPRGSLFKLLHRTPAFNPDERRRLQMAL
DIARGMNFLHTCKPPIIHRDLKSPNLLVDKDLTVKVCDFGLSRARRSTMLSTKSQAGTPEWTAPEVLRSQPYNEK
CDVYSYGVILWELMSNEEPWHDRSAMQVVGAVGWNKERLPIPSDAPQGMQDLIAACFGEPQLRPSFSDIIPM
LKQMIKALGPPPGHERLGVCAGWRGAYLYGINDDGTIQEYNSINKTDVTVLATGCVTVGGSTAQSNAFAFDKD
RDVMFWLYRSNRLQDPKAGLWYWDRQAGVFGVFAPPCDVLLNFVADNPAYTNCAGRDPPAISTELPADADF
YDNAFWYIPTRTPGQSFLNGYPRCSLVKVPIKYNAMQRAIGIGTPVVYDVSGGGCPAISTGLVFGDIATNRKTGI
MYGSTATPPRQFFSINMKTLQPRPAVNNWNQIAAPSPAGTLGFQISWDAEYSTLYGVTAGGGSAGGLWYTLN

LATGAPTLIPGFVTRPGPRDLGGAACDPLPPPIPTLTKVTDPADGNLTVGDLFTYILTIQNNGTSDMFDTVLTDT
MPPGIVAKAASCTPQGSCDVSPDGASVTCQLGTVSQQGLPVICGIVAMAADVDPPGPRVNVAVLSVSNPGVA
DVTDNATVTVKPAPEPPRPVIAKTPSQKEMFEGESFTYTIVVSNAGGTTLLDPTLTDKTPAGISCTAVQPDSCGIR
DATVFCQLTDLEPGESFQAVIYCQGTKEGKWTNVATAESSNANTVATAAIVTVLPKPLGPVPTIVKSAWPVVMT
VDDEFEYTIVASNAGDTVLRDATLKDTLPSGVKATGVSSKPSRPCRIFAGVLACNLGDMAPGDQVQITVSATATK
DGVWENVATEAGNNYDGEVTDNAFILVKPPPPTPQPVVTKTADPSTVVVGQEVTYTVIVSNVGEADLLDTRLV
DTVPAGITALSVEPADKCQISAGGQLVTCALGTLGPDVEVTITANATQNGVWNNVATVTAGNYVGEVEDDALV
VVNPRPEKPQPVISKTVEPDTVTVGQQLTYTIEVSNVGKADLLDTTVTDKLPVGIIALAADPADACEVALGGGSVT
CNLGTLTPGGSTTVSITAKAFECCGVFSNVASVTGNNYDGSVEDDASVEVVRPPPIPQPNITKSADPNTVTVGEL
LTFTLTVSNAGNGTMTGTTLTDDIPAGLTALSVEPADLCTLNADGSKLTCALGSLAPEASAVIVVTAKAEQAGTFK
NTAVVTVTNPGIPPVSDDDTVVVKPPPPVPVPNVVKSVYPPVVAVGEQFVYTITVSNAGNATMTGTTLTDDLP
NGIAGLAVEPADLCQLAPGGGSLSCALGNLAPAASVEITVTAVATRPATFYNTAVVSVTNPGIPPVEDDARVIVTS
PLIGPKPNVTKSVDPETVTVGDQFTYTMVVSNAGSAAMTGTVLTDTLPAGVTGLVVEPADKCQLAPGSSSLSCA
LGTVQPAGSVVVTVTAVATKPGTFSNTAVVSVTNPGVPPVEDDAEIVATPPGVGPKPNLTKLADPNNVTVGKQ
FKYNLAVSNAGDAPLEGTTLTDTLPDGIVALSVEPATLCALAPGGTAIACQLGTLAPGDSVVVVVTAVATRVGTF
KNTAAVAVTNPGIPPVTDDDTVVVTPAVCVCEPQPVEPCYATIKSCRRCAPITVYLAVTAPATLSPQQLLDLEARV
HAALLAAQPQLALYPEFSVVAQPAAGGAVVAVTLPPGQVGRGAAASLAAIHASCEANPLAVPEEGICQGPIQILC
GKCKPIGRPPPPGGYGGWHHHKRPPPAHHKGEGKGKHSKPGRKPNKGKPKRTQQKP

>A795|PRW60877.1

MGRWALLVLLAALLGAASSAAQPAGAAGPPPPPAQELLAPVPAPAADASAGLFDMALEAAAAGDAAGGIDLL
TPAPPPRPALGAAAQAAALGSPPRSLGSPPPPGFSIQAAASPAPPKSPSPPALLAAAAEAVEAQPPGAAPGPSPV
PSPSPTPSPAPGAVNAQAVSLLAFKNALTSSDSLATWTNVTDNPCGPPAWQGVSCDAGNKVVQQLDLWGLGL
QGPISDRLGDLVGLTRIELAQNQLAGRVPELQLPFLQELSLHTNNLSGPLPSLSKLPSLLTANLYNNQLTGSLPAA
WSELPDNTTVRVLPNNLCGSVPSEPYPQLCFVAETDSCYPVVNTLGSCFNATCNRETSLAGTNVFNAALDNGAT
VWDTLAVSNLSGLGAGSSWSTGPDGIGIGNATAAGNTSTAAPSKVALPCYLPGSPVGDYLGGDQAHMMMA
WGDAQDPSAPAFVVSEVPGNTPSCSSQGALWAVDLRYTTQLALVVIEAGANGISNAAIQVSDSPALPATGRYM
TVTADQPMDLCRVSVYPAAADAAWNQPYRATSDLGDAVNGTYGEGGNGTVVVKASRGVQAFPGTTPTFVLD
LGANAGVAAVTLGTEQFTVTAVKVSTTSPELSMFAAGVRRLRQLGVGGGGVNCQLGTTFSGIDSVYACNGGQ
GRYVVVSGYPDGELVVRSLAVYLTQPRNRQPPSPSSPAPPPPPPAMSESQYVGTLLLGGNISQFSNQDADAKQL
LAVYNRAWGQALAASGFTLDPKATSLQSAQPNVSGLAGPPIYYSATPPPGGNLLTNTSISFLGSSWLTRDDLYDIL
GTSEVAGSISQGLAALDYGQVTVWMLPPSEPGSTSSLSAGAVAGISIGIVVVLVAAGSGLFVWRRRRRTQQEGA
ALSKAQVAEEGLGVAAVANGSGSGSGSGSPPTPAKHGSPPGSPAAAAPLAYSHALQHTQAHAQAGKKDSGRP
DAALLWGSAQQYGNGLLPSLSARSTAAASAMSFGTSVAPSSRLSRGASANESQLAGPTSSDLQRLHDDPLLEWI
LKSQSPPTAAESASAAAVAATAAAAAEAHTRASRDGNGAASDGSSSTSAADAAAGTSPASPAASEMQQQRRG
SSANPLMDVRVWQFCFRELEIQRQIGAGSFGRVYLAKWRETLVACKILMDTGVDIEDEDDAERALTLSNPVLESL
EREASMMAALRHPNVVAFLGVCASPPCVATEYCSRGSLTDVLRGGKGSATKAKQLDWARRLNIALDAAKGMH
YLHEHSPPIIHRDLKSPNLLIDRHWRAKVSDFNLSKIMGDNAVMSSVAATNPRWLSPEILDGKKASYQSDVYAFG
VVLWELLTWELPWGATNPWQVVNIVAEGGRLEFPPREKLPGPDSQTWTELDCYCQLVQHCWAQDPADRPLF
KEIIIQLRDMLERTLAAKASLAGSSVAGGVPYGSHAPGGSSAGEPTGGTPSAPPTPARSAPGSPALHVFVPPGVP
MSPAASQRYGRLSRGISGLSGSSSGALLVGLQPADSSRRVLTATGAVPAIVGGRNAQKNRFPYYTSLREAVSSYH
FCGSTLIAPRFLLTAAHCITEWATPQAVEWSAPHRAFPRARVGAYQTYPVPAVPGVNSYEHRQVIAVAVHRLFD
SALQNDIALLLLDQPATRTPITLPMGPAQLQRRAPVGSRMLSVGFGMSEFQDVVDDLPAQAYEPTLQQLWML
RQPDTLGTFVMAGNPPRFLSDTWKGDSGGALLVPNATDPSRDMQFGIVSNGMYGAPGYYTDVSNFTAWIAR

GQQLLLDANGQGPDYHIKRIPSCASTTNNYCVYCNSQGERLTCVDKTPCHIAYQGQTQLLLRCNDEEFDAGPRA
SSGCFRAIQYTDRTGYGQGPGILGNQTFTLQAALGARLPNNTSPSCSAAAPFVLKACPGASPAPSCVPGARALTG
LESGCCACRRVRAATPYALVYPATPVTNTWYETVPNCKRAVRALPGGALVHYTGALVTGNPWLACQQKKYAKV
VLEVLSSSGSGVKYEAFYVEADNVRMADSAPMEVEQAQPVEEKKGKTKASGKRFEIKKWNAVAMWSWAICTD
TCAICRNNLYEPSIEYQANPTGDADHPGLSIAWGTCGHVFHLDCIQRWLKTRSACPLCNREWEFSKIERIVGGSA
NAE

>A796|KXZ49686.1

MQEQEKHVEPRRLHGNGAAVVGGRELATAVIQDTVKYLGIQATGISHLNGPYLSVRNVGPDASDILLVSSWRA
HYEPGGNGSSPILDGLPPAITQKAGAAQAGQNGLALLTQSSSQAAPACEAMAGSFADEREYAHRHAAGHAEA
NGDMMHQDNHHGHPEGPARDAPGMASGGGKGPGAGGPFAAEALRAFPSVASERAGPIIEEYTSDAEPPSPG
GHRRRGRQQKYGEEHSVENGRMVSHAMPIVQLADSMADPDRMMEDVPVQGGPNGQGPAQPVTTVAAVA
QQPQVGGQPSLGGGLPAPGHVVKLAGPGLGALSAFASTAAAEAAAVGGVAHASTAPTPTSGPLVLSPRPRSE
WELDPSKIVLGRRLAVGGFGEVFVAKYEGTLVAVKRLLASDSDTTQRFIDEVHMLARLRHPNLLLFMGYTLTPEP
SIVTEFMARGSLFHILRQAGNRPPDPRMQRAVAVAVARGMAYLHSRSPPILHLDLKSPNVLVDDRWRVKIADF
GLSRVRQRTYVSSGAAAGSPEWMAPEVLRCDHYAEAADVYSFGVVLWELLTGQAPWADLNAMQVVGAVGF
ARRSLPDPSEGDQLLVHLCKACRAYEPTQRPSFTQIIEAMDSHFGPPPWPETAAVAANNPAVAAAEGAAGAAA
DGGAGVAQRALPPPEGASEPAAAAEVVATQIGPPPRLVRSHSLASAPDKDAGGADRPDGAVPPTASINVPPPR
LDARFMSVHESPRSRRRYRSPRGEDMLLGPPAITARLQDGRKTPSPRTASGRAWRIGTVDSCEDYEAPPQSEQQ
QPKARSPAPKAGGAASGELTVGAGRVPAARGSPFADLAAAEFAPGSVSDVRSLRVEIPSEAAVAEGDEGGPFDK
PESMELGSPVLQQQGLGMRLQQYYLQHAGAAAASGGGGAEPSTPGAAGGHAGGSLMPPIQLGPFQPDPPG
GFGPDDLGQRLDAAVAAAAAAEAGGGDPWDGTQSELSFAFILADDNASICSDDFLQSASLDRIPDVAEEEEMA
EVGDAFVRQEAPGSDGGAPDSNDISFKLPRPLLINPSQPHPAQPDQADQGPPASPFLAAAAAAEAAAAVAAQP
QAPGDVAVAEPPSADLWAALQHHSAGQHSPKQHMATAAWLQDAGLDEGGADAAGGAAAWSQQAVRPQ
VPAQPELGSQPEQQPAEQQLLQNNKASAHAQGGDCSTVLQQPRQFSQALPEPAGRVEVARFSSSGGGSGVTT
YRTGRFKVTIEPAVSDAQQPVQTPATIPAVQQQQQQQAHTAHINPVDQAPPQEQQQQQQPSLLMHAPAPAP
AAPAASVADAAAAAAVATAAAAGNTPMSPTGSAGGAHPAGSSPGGAPSTCGRGRPSHHGACHSELSGGAPV
ALNCVTMYRKGRFFVSHAAAGTHSLTVTNPFAGAGTSSMAAGGGGGSGGGASSAESPHDESEDHDAASGPAS
PRRAAAAVGAAAAAAVGAAAAAAAGGGSALASRQNSVEGSPQEASRPMTWAAVAALPPPAKPQEAAAAGS
VVGVGRQYSTGRFTVAESIVVAPVPEPRRPSCGSATGERDAAGGAPGAAMAPADGSLPLITVHSAVVGESTAA
AAAAAAAAHLQPHRVQSAPCIAPDASQWGQFGPPAAAHGQSTGPAGQPPHHAPGHPGAAATAPASPRNEH
VVVRKVGRFTVREVEAGSNTSSRGPSSSGTPVEGTRPEALPAAGALPAAAAGILLSVAGMGCSVPGSPPEDAHL
LSSHHSSCSFASASGGCSRSSSQGIEMDMGAPPAPTQLAQHLDMDLALQPGPSQVQGQRDQQPQPEGAGPA
AASVAAAAGEPAGALPAAGVPPIAAAAGAVEFAAPPQVLRVKSKGRFTVIEHGAPLVGFLPAELAPPAPAPRTG
PGAAAAPLPPQPGSAPWGLSGPQAPEQAAQLHADMARMVQERMARNEREAWEQQQPQRRGPGQPWD
PARARLGPGGGAAQGGGDEEEEENAGDDNAAAAAGGLGCLYEVTIGDGLTIQISHGIQGGGGGNVGAEEEED
GDAMMVDVNLLHGEDEEGDDSASEGGGDGGGEEQLPQGGGGGEPNGNGGDWEHAPLEPPGTPTHGAAH
ERDWLLGGDVGIEWRRRRAGRGRDGDDSSSSGDFDSMDDS

>A797|GBF93972.1

MRPHQPDAGALQPVDGAPSLPAGASSTQAPPQVTFEDSGDSTTIRVAPWRAWGVEELTPVFDSLGVEVLSCSS
PSSSGPGDADITIVVAHARSSKPLDARIKSSLACSLASALSTGGSMLQQQQQQQQQQQQQQQQQQQQQER
EQRVASSSGRGSSREEQLSSGGVLGQALRQQQAFAQRAAHAQHAALVRAGGGDGTAWEDGGGTDSPGGRG
LRLHVPDAAGTPPVGWLSSSSPPAVQPADTSRLLRSPLATVGPTPTETPRSPASGSRRLSGGEAPAVPGPSVAAR

LVPLLLSPGRDGAGAGAGGAAEPRRGIARRPSRIILSPVDGSGEARLFVAIPKSPSERSAASSADTRRLPSRRASAL
TPPPSTRRMSAMSPSSEAPGPDADAPEKRCSSADQLPTVPNSVAVAAAQLLAGRQRRQGGEGGGAAAKVGA
GDAAAGGSGAAARPRPALPLLVLGEGGRADDSEADVGHAAAVDEPEPPRQQQQEEGEEGGEDGQRRRQEAA
RGAADPSSTPDPADAGADAASSCSEEWWCGPPTGLAVREVMASPPHWIDADADAGLARTLMAAHAATLLLV
DLGPGLEPGLIEKRDLFKLPSIKRRRGGKRRVAARDLMHHPVVVVGAGDEIEAAAQALQEADMRRAVVLDEAA
AADGGGGGDGRARWVGTISDVAIYRCMGFHGGGLSDEEDDGDAAGGHARRPGAEALGVLTEGDATPGASR
VATPTGARRVSTSGGEGASGDGGGGGAAPGKPASPGGSAGAAAAPTPPRPGSFGAVLTLPEMLVLRERTAGA
SLSSHSSAGSAAGASGASTAPSMGTGTGSLTGSMWGDAPDALTRYKTAASLWELDFDEIEITRKIGEGSFGEVLL
GNFRGTKVAVKRLHALDGDSSSGCGSGGAVQGASQAAFKQFFEREIAILASIRHPNVVNFIGACHRASTRCLVTE
YCARGSLDKLLHKSGLALDLSKRIEFALDIARGMACLHAQQPIIIHRDLKTANLLVSARFEVKVADFGLSRIKDATHL
QNSRAGLEGTIEYCAPEVLRGEPFTEKCDVWSFGVVLWELLTRARPYSDLDVPVYILMVNVGNGSLRLPPVPER
DASPRLCALVERCLSAAPAERPAFIEVLPVLEAEHRAARARAGASSGGGAGTPRRGEASGAGIAGSGAGGGGGS
VGLRDSIDLPRGGFDTPRGGGSGSGGGGSGGGGGVFYGGPGGREAASPSPFMMQQQQQLQVQALGGSLSA
GGYGGYSSFGVAAAAAVAASPFLQLADGAGGPSPPLQQQFSNPQQPRSRLAWQEAPAPSPADGHRSGGASG
GGGSGGAGHHGRRRTMSRARSMPMPRHSSSWLEEELDAAISGGGFGPGPDSGPIGGGGGGGGSNAPAHGG
WGGYAESAAGSDATSGHETPGAGGWASPRAGGHWPPQHQWPPQQQHHQQPQGHQPPCTPGAVSPFAV
GSPSQGGTMMAMAMIGAAIAAQAAASHRLPSLAHASSGGSADREPAGAGPAAAGPPVAVATSAPLPLLPLAQ
PRHGSPVRHLTPRGLGHGRSTGGGAGSGSATVASGAGGRGELPPLQLLQIGDGGGSACLDGLSPRSAQPPAAP
NSAAVLLQRHDSAGRSRSPTASPRAGAGVSPFQQYSRLPSPDCGGSGGGSCSGGGGGGGGGAALGSFHRHAS
SRLSVQGSYELPPWHGCGAGDGGAAAAPAPGAPPPSGSCTPPRQHVAPKRHSLLRHESGRETPVPAAPAART
QSPFALVADNAPGSARSSAFGSPTPGSPVSQQARFRQLQLHAQPPPPSQQPQPQPQPQPQPQPQPQPQPQP
QPQPQPQPQEPPPSGRASRTGPLPSPFALMQQ

>A798|Vocar.0035s0068.1

MSQALRWALTVLLCGRTVREESSNRSAEELSVAGAAPLPAVPLSRPSAVMLSNHQPTPASYPPSEDHALSIALAQ
SVADQAAWQAARASGSQTHQLVLKGSAGEALPKKFWQDGSLAYSDVVTDGFYEIYGDFPEVCEGPHEFPSLEV
LRKVRTGSGDVREVVLVHHEQDQGLLQAEEMLSEAMAEANPQDVMARIRVVAHVVCDRFGGTYDREESLERF
WQVSSANVKRRNRSAVLLLCQLDVGSLRHRALLFKVLADAVKLPCKVIRGQALCGADSAANAIVMVDGREHIV
DLVFDPGKLISQDQFAAMVQERRLHAGLDAWHHPAHDTTSAAAGNASAVEASAASASSATGAPPAVGTVGS
MKSTGVSSSSSSAQLGPVSSGVACSGSGGQSAVCGTNNLGASLPAFVSAAADAAPVPHSSPRGAPSRTQMLVP
PVPMPAPAVYSSSLPAPGMGLHVQSGAWVPGPAHAHGSGPGPAIAGSRVRFTITDLPKTENPQPQGGRGDSR
QAPLAVPPFPAPPHGAAAAAGGAQIAAPHSGRHSLSSLPGKAKSMLDLAVTVQRGGDCMPSGSGAGCDLIRLD
SEPLPDSLHDSVPPPERSGTPTGTSAPGPRGPPVVPGRRQNSFDQNGWVKFSGSFTRSKDDSRTHTGGPGLSPL
SQSSGGCAAGAPAAQPGSFSGSQTAGGLQPLEMVSSFPILGPAEPSPDGTKLDDSVSSSNAARASSAGSLGQM
GSTSVPPVAQGASRYSTATNASAFANFQLPSDLSATACSDTGCSAGGSGTAGSAGASGSHLSARIPHGSSEGSPS
KTIIPPQQRSGPQALASQQQQLSIQSALPQPPQQQARSSLALASPFHAMQMGFASHMPPASLVSAFGAVPPTP
QPGTITHVQQPTQQQQQQQAAQRPPQVLAGAGNAGTSTTGANLPLPRMGSLPHAGQPQAPSQQQPAHQQ
QPAAGSAVRQYVGVSHDSAHRMYGLGNGAAATIGGTAYYATPPKSADGIGNAGSKAPSPALSTPFTAPTASSA
QSTPPGSAPGPSVPFADLSPFNVGEATSGCGSDRKAEKTERERDQREREQRDWERGRERDRERELERDRERDR
AQSFFADLSPFRVSLGPGGETRDATPARQSAPGPSSQPIVETPSSDNSRETTPRVSITNITDTTGGVGSGCGGAD
GVALDLSRIQLGGQGGGRPDPSGGRPGTAQQQQIQIQQGFGSMPMMGPAAVGGHLGQQLPHHVAMSYYA
AAAMQGNPYMYMQPSAAMAAAAAAMMPQAAGFMHGPAPAGAPGMQGWRHVFNPAWESSWTAAQQ
SSQYMLQAAYLHHQQRQHQQAILAAAAGMAAQPQPGGALALSGIGMPVGGSSTVAAANLLESGTALATQTG
GPGAIAARPPIASGSPQLALPPSIQPQLPQTQPPRMRQQQQQQKPPQGSQGALDLAHEMTVPSARRMERSRV

SGAGDSDATAEAGLPSPLGQQPSLSASMPLPPSHRDLEINPEDLTFGQRIGMGSFGEVYRGTWRGTNVAIKHF
HDQNLSPVTIREFRDEVLIMSKLRHPNIVLFLGAVTQKNQLAIVTQYMTRGSLFRMLHRNKEVVLDPRRRLNMA
LDIAKGMEYLHNCKPVLVHRDLKSPNLLVDKDWTVKVCDFGLSRFKNNTYLTAATQNGSPAWMAPETLKGEPC
DEKSDVFSFGVILYELVTGKEPWEELNPMQVVGVVGFSGRRMDLPTDLDPAVTNLIQSCWATNPKERPSFTQIL
ATMNTWSELRPTAAVLEAQRAKAPQRNAAQ

>A799|PSC72028.1

MLRRLLAQPHAAAALLQSACAAAGPSARPFSLVQLAALSASAAAASGKLLGAVVFERLPVVVPEDPEWEREYFE
WQDGLAGKYRKELPREFTDLKDSEMKEDAGASYEPAPRVTAADHANDRRSLKRRLDQRLFMLVRQKGGWTLP
QQENAEGETMRQTAERALADAVELEAVRPYFVGNAPAGHAPLGDAGTLFFHRCQLIQGVPALRGGSGYSEHV
WVTKEELGEYLQQPDVLAVMQKMLHANEQDLIVSHPENGTDLRWGLNDAGRQQAAAAGAQLAAALASEPG
YDAASQLLVLTSPFSRTVQTAELAAAPLGVQPGDARFQVEAALRERCFGAFEMTSCSNYEVVWAEDAASTANR
SPEATLDGLRLATTNWPEFQSANNLSGWGADAAVPPCLYTGITCDADGRIQTISLQCQTCPVKLEGTLPAALAQ
ATSLNTLNLQSNALRGTLPPEWGAPGALPSLLNLLVLNNQLTGSIPDEWADPEAFPRLTLLGVSSNRLNGSLPSGL
VLPSLLILRANDNQLTGPLPPEWGTNASLPSLRLVSLHRNQLSGELPAEWGAAGAAHNLEELYLQENQLTGGLPP
EWGGAGLPQLRVLNISTNPLGPYTLPAEWSRERSFPALTTLVLSHTGLEGSLPPSWDCVDPERCGLPQLQTLWL
DGNNVTGPVPPSWGLLRNLDAVFVWPGNEGLCMPLLADFRFKLCDASKGAACSDEVRLPTCPLPATAPSEEAV
AAATGAPDQAGGADSGGGGAGGSADSGGGSSTGAIVGGVVGGVAAVAVAAAALLLLARRRRRRQRQERELP
FYAPDDEVDKSLHPRGRFFLAGLLGDNRTPRGCSPLGGSPGTPSAPRVQQLWHAPGAEGALASPFAAQEPFPA
HEGDSMFSPFAGAAAPAGEGLHSGGGAAQRAAQQQGQQDVGQQESSMDGASESSNAILHRMALGRGGSG
GGGGGGAPALAVAAEADAAAAGQAAAVDEAALDAEMERHLSAEMAALPRSASARQLAAGSSPSTSAAGGAR
RPSPCSIIPSSAGSSFGEGLLPGLSDWEIRPDEIEIQKRPDGTDWELGSGGFGKVYKALRHGAQPVAVKVLMLVG
GESRYMAVEDFRKEIAILRACRDPNLLGFLGACLQPGRTMLVTEYCEGGSLTRNLMADRVTWYKRGKKISLDVA
KGLTYLHSRRIIHFDLKSPNILLARDGTAKIADVGMAKIMAREYSGVTGNMGTLAWAAPEMLLGARCTEKSDIYS
YGVVLWEICTGQTPIRGQLRDVSVPDECPPEVRELMLDCLATNPRKRPSAVEIVERLRGWPDTPAPPGLAAGPH
AAAAAAGRSPAAQRPAAAAPVWQQIEMPPPQPQQPQQPQAPSQSTAGVSTPSTLLESLGGEPPPQLPSAYAS
PLFGSSAAAVAAANRLAALARPSGASGAAPLRRAGSMQVAAPPPPLAIPPRQLHEMMMQRASSSPPESHLFGP
GLSPFAAASPFAAAGFNGAGGPQSARRRPPWPMQRRRSLDDTLQQFTEVQRVVHLLGGALQAGGGEQAGQ
QQPQEQTREQQQEQQQQQQPSSVQHRLVRQQHADGLPRASSACELGQQQQQVGVPRASSACELGQQQQ
QPLRASQTDSSLTRVSGSTRSSNEQLSDRHSLEQKALAVQQQGQANSLASEHRGQLRRLVARQHQEQQDLDG
QQQTERDEEVQMWLDLEEQVEEAEEAQHALQAQQQQQQQEVPPVQQQQQQQQQTGSAGKQSRQPSLGS
ISEAAEAPAQ

>A800|PSC70242.1

MTRWLLAALLAALLLGSAAAAPGGGGDGGPGRRNALAPSPAEAPQQQDQQKQEGASPPLGLEPLSPEPRGGG
ESPSPAPPPTTDGSPALPPPTDGSPAPAPVASSPAPAPADSGPAPVPAAAPISSSSPAPVPAAAPPSSTSGPAPAP
RVLAASSNANRDAVSPTLASAPAPPPSDAPNSDNGDVYVAAASAVSPPAQAPLARPPSKQGGSNDYGASPLPQ
QEPSPSPIVEAPAPPPAGQGGSNALGSPSPSLEPQSPLPSPDPLPPSPAPLSPSPVPAPPPGDFTVTDEDGGKAPS
AAVDSPPPFSPSPSPLVSPPPDPSPDPPPSPVLPPPPDANPQAAALLDFKAGLSDSSQDLLEGWNSSTANPCSG
WKGVVCDGANENVQGLQLSGLDLTGTISPSLADLPKLQQVDLSDNQLAGVIPKPWQTGDGNAPELQSLLLHN
NWMTGPLPALTLRTLGNATLHGNMLTGSIPISWATAPSGASISILPQQAEKQGGGLCGDVPSAPKLTLYAPPGA
WYPVVNTLGSCLSSCNNPVTSLTNTNIYQAALDSNATVWDTAAINGWAGQEASAESVTIPCYQQGSPVGGYLG
GDQAFMMVAWSNGGSTAASAVVAAKPGDDATTTCGMQGTFWSVDLRYNLTVTQVVIQAGSNGIDDFAVYL
DNELLAPGSGTPCGTGISLTSAAATVSCAGITGRYLTLVSGAGTGMSLCSVAVYPEVANAAINRYYYFTTAGGVV

QAATNAAGSSHLVEAPDGQTPTFVLNMGSKALVTAVSLALAENLATISVSNSNPLVPAAAPAAPAPAAGRRRLA
RHAQRALLQGEPSAGVVSCTPGPQPQPNFSYNCAGGTTGQYVIVTGTPGTTLRLDNLAAFLGQPPAPGPSPSPS
PAPAAPSPPPVRTFQTMWIGNLLVGGNLDRFQNTSPYSDEVLETYNAAWSNALIQRGYAMDTNASAFTTVEK
NVANPTGSPYYYGGTETGPNVFTNTSVSVDSRLSYQRLTELLDTPEVAGAIETNLSGSWGLFDAWMLPYATPAP
PPSPSSGGLSTGAIIGIAAGGAAAVVAAAVALFCCVCRGDRRRARQDTGTSKGSLEAGDAAQSMELGGLRQQY
PPPALPGGKGGSASTQQLADARKLYAEQQRQEGDPAVPYGSAYANGGGSGGAAQHSHHPSADSSGMLGGG
GALSSSEGAGASGSGGPIADDPLLEWILRTQSNTPQHAGRSAAMAAAAATAAATAAAAGDATVLVEAPRDGG
AAPGGSGSARGTPKHSRSGSKALMDVRVWQFDFRDLEIQKQIGEGGFGRVYLAKWRETLVAVKVLLNTGVDIE
DVDDAERALTLSNPVLEDLQKEASMMAALRHPNVVAFLGVCVNPPSVATEYCARGSLTDVLRGGRANAAKAK
QLDWARRINMALDAAKGMLYLHEHSPPIIHRDLKSPNLLVDKHWRVKVSDFNLSKIMEENSVMSSLAATNPR
WLAPEILSGGKATLASDVWAFGIVMWEMLTWDLPWGNTNPWQVVQIVGEGGRLEIPPRERLPGPDSATWQ
ELDAYLALLDRCCAQTPAARPKFQEIIVVLRDMLERTLAAKGVIAGSGGAGVSAGASPGGTPFTDSLLRGAASAS
TAATARGGRGGGGLAPATSNASSLAAARGVSTPASPVAGGDLTLGALSAHDSEADTPMSAAAYGSTLAELGSV
GTLSPLGSQSGGGNGGRNA

>A801|Vocar.0035s0070.1

MLASRNVRAAAGPRVAAAPGQRVILSARAGRRALTAAKAQKKEIMMWEALREAIDEEMERDPTVCVMGEDV
GHYGGSYKCTYGLYKKYGDMRVLDTPICENGFMGMGVGGAMTWLRPIVEGMNMGFLLLAFNQISNNCGML
HYTSGGQFKVPMVIRGPGGVGRQLGAEHSQRLESYFQSIPGVQLVACSTVRNSKALLKAERITSDVHRKKALEN
HENLEITSAGHAGLDAWHHPAHDTTSAAAGNASAVEASAASASSATGAPPAVGTVGSMKSTGVSSSSSSAQLG
PVSSGVACSGSGGQSAVCGTNNLGASLPAFVSAAADAAPVPHSSPRGAPSRTQMLVPPVPMPAPAVYSSSLPA
PGMGLHVQSGAWVPGPAHAHGSGPGPAIAGSRVRFTITDLPKTENPQPQGGRGDSRQAPLAVPPFPAPPHG
AAAAAGGAQIAAPHSGRHSLSSLPGKAKSMLDLAVTVQRGGDCMPSGSGAGCDLIRLDSEPLPDSLHDSVPPPE
RSGTPTGTSAPGPRGPPVVPGRRQNSFDQNGWVKFSGSFTRSKDDSRTHTGGPGLSPLSQSSGGCAAGAPAA
QPGSFSGSQTAGGLQPLEMVSSFPILGPAEPSPDGTKLDDSVSSSNAARASSAGSLGQMGSTSVPPVAQGASRY
STATNASAFANFQLPSDLSATACSDTGCSAGGSGTAGSAGASGSHLSARIPHGSSEGSPSKTIIPPQQRSGPQAL
ASQQQQLSIQSALPQPPQQQARSSLALASPFHAMQMGFASHMPPASLVSAFGAVPPTPQPGTITHVQQPTQ
QQQQQQAAQRPPQQPAAGSAVRQYVGVSHDSAHRMYGLGNGAAATIGGTAYYATPPKSADGIGNAGSKAP
SPALSTPFTAPTASSAQSTPPGSAPGPSVPFADLSPFNVGEATSGCGSDRKAEKTERERDQREREQRDWERGRE
RDRERELERDRERDRAQSFFADLSPFRASLGPGGETRDATPARQSAPGPSSQPIVETPSSDNSRETTPRVSITNIT
DTTGGVGSGCGGADGVALDLSRIQLGGQGGGRPDPSGGRPGTAQQQQIQIQQGFGSMPMMGPAAVGGHL
GQQLPHHVAMSYYAAAAMQGNPYMYMQPSAAMAAAAAAMMPQAAGFMHGPAPAGAPGMQGWRHVF
NPAWESSWTAAQQSSQYMLQAAYLHHQQRQHQQAILAAAAGMAAQPQPGGALALSGIGMPVGGSSTVAA
ANLLESGTALATQTGGPGAIAARPPIASGSPQLALPPSIQPQLPQTQPPRMRQQQQQQKPPQGSQGALDLAHE
MTVPSARRMERSRVSGAGDSDATAEAGLPSPLGQQPSLSASMPLPPSHRDLEINPEDLTFGQRIGMGSFGEVY
RGTWRGTNVAIKHFHDQNLSPVTIREFRDEVLIMSKLRHPNIVLFLGAVTQKNQLAIVTQYMTRGSLFRMLHRN
KEVVLDPRRRLNMALDIAKGMEYLHNCKPVLVHRDLKSPNLLVDKDWTVKVCDFGLSRFKNNTYLTAATQNGS
PAWMAPETLKGEPCDEKSDVFSFGVILYELVTGKEPWEELNPMQVVGVVGFSGRRMDLPTDLDPAVTNLIQSC
WATNPKERPSFTQILATMNTWSELRPTAAVLEAQRAKAPQRNAAQ

>A802|59547

MPGGSLLLLTAAAAVLLLSCPPGPVEAWGHVEMGQPGPLQGCCGGRGIARKVLLQVEPQTAESPGQQEGSLT
AGPPPPVTSGVAQSPPAEPPPEPQPPEVAASPSQNPDSPAAAPEAPVASPEQQQQPEASPPVTPSAETSPSPNI
PQQQPDSSPRSPSPVPAADSNPYYTLAKKRMFPLRPSTPVLENGTAYTREPVDTSSAGRSGEASPAAQGLPSPP

AGGEGDQMSLLAFRMQFIVAGPGLIPWAYDTPFLAGMNDYLGPNSGVGNLTIRNWAEVNLANFPEVPALLSL
PKDGLGSPVWIIMTGTTTALRGYNRLISRIVPLTAASRSADLIPYMVADGLNVTRVFLYNLDYGPFTQLPPAVPSA
PPAVQSRQVPPPDRRPPSGGVPSAAAPAGAAAAAGASAAAQSDAKANVGMIGGVVAAGVVGIVAAAAFLFLY
ARTRRRKAEHLLALHSSGPSEGHRRNRSFDWAGRLWGPPSPGPPGTPNLRGKASLAQARSSALSARRSDPWAI
DPAEIHVMQQADGQPWVLGEGASGSVYKATWRVQTVAVKMLTHTTEKQMEAFRREAFILEDLKDANIVQFM
GACFDEGSTMLVTEFMAGGNLFDAIGNDRSGKLGWYQRGRKIAMDVARGLDKMHSRHIIHLDLKSPNVLLTA
GGTAKIADVGLSKILVNESTMMTNFQGTFEWAPPELINGGECSEKADIYSFGVILWEIVTAERPFRKQSPEECPAE
IANLIRDCLSPEPESRPSSTVIFHILQQQAALAPHLSTPEATPRDSADVSAHTREDASTSARAADAAAPVLPAVPPA
VLARGPGAVMEKVALPPRPSTPIGERMPPGLPKSPSRPPPTSDTTSVGTPSGNGLPRRPSATTTATSSSQETEGL
VGRQSGLSEPTSSWLPSGGTSTSAPGQELPSSSGREKRLRLSGQPNANGFAGAASSLMSAASGGFTQGVSERPG
GPIGSTSRGLGFAPDEDVRWAGSDHDMDSGPPVRHIAWHKYDFIIQTSCLLTAFVLSLRYIIRSASGSVEAQSLIL
AVLCSVFVALTGYVVAKMAYSIHRRSRALAHLPGPKYPWLLGDMAFLARKDPHRAATELAERYGPIVKIRVMCF
HCVIITDPVLATQILRSKVVDKMRFAYSFLDPFLGGENLLTGHTDEHWKAVRKAVAPAFSAGNMRDALEHVVDR
TMALVDYLKEGGPTKVHNVDNLLLRESMDVIGRFGFQKEMNALHSLRTGNAEETEAVASLLGSTHEIERRIQEV
YRWWRLWLPDVRAGWGMLGRFQAIIQGLLEHIKAGQPKPGSFAQLLLKAKDPKTGQRLSDEQMFPEIVALFFA
GIDTTGHTGTFVLYTISQHPEVEAKIMEELESLELTITPDRPTPRPMVYADLSKMVYLNAVIKEVLRMYPPVGIGQI
RVSHSHDITLAGRLHIPAGTQIWVPHHAIQNVSFNWDDADKFKPERWLTPGTEYAVPEKLPLPREWYSDWDR
DGAADTNGGALKSANSGPAGGTDVDESSNSKRPKRYFPFAEGPRNCVGQNLAKVSLLATAATLLQHFSFKLAKE
MGGPEGVRASEHYTLVIGLSNGMSMHATPRPGVM

>A803|PNW71720.1

MSFICKVLRSALLLLLLAGSLLDAAWAQPAASPPPGGGWPLSAPPPQSVSDVAKTPETIMQLRGLTGIRSCAELQ
PMFLELLDSGNIFSRNQLCYVGREVLTDGSEDLSDPGLQQLSPPSLPLSLTAPSPPSLSSFVLSAVLPSLEMAQRAI
ASLGTDTGADVSTFMQVAGMPCGGALLATTLTEDQDTATVYTFSCDGTLSVPGSVASLVLPAMCCATNVPRPP
KRHTPPRPPPVLPPWRALPDPPSPEPPSPQPHPQLPTRPPRPPTRRYPPPAAVPGAPQLTLFVMADPQRLLCAK
LLAACETLAQPYGLLNGKYGCYASNELSSSGSTTPMPRYGSAAPLPSAYTASNESLAVNGTGANSTDLVLAVPYM
IVTATWPARLYSRSFRRMQAALPSSVEVFASLGAVPCNTEVRAVLLSTTGMVHREEAYGCSNALPAVYTALLEQ
DGLKVVPRVLLCCRPPPAPVPPNAPPPGGPALPPPVHPPPGPFTPPSPPGAPPPGLIIELAPPPQPPSPPRSPSPP
PPPPPPPPPPPPFPGYEDHVSTHPRPPPGRVQNGQEWFPPPPPSISYTSANLSTGAIVVIAALASAAFVSTLVVVF
VLYRRRSLGGAGANKSGPAPGPGPAPAAPASSEGYQAQHESGAAADTAWVGISLTDGDSTYHQLAHPLQQQD
KQHVEQLLQQAQKKQLSTKRADGGLLAYQSSLESQVSVAGTGVALGGTTSIGTEAAASGGVASPQWVVLYPGG
GEAGLDPHRVSVLLPEGDSVAVIRASPHRALPAPVWQQQEAAAVEGGVAGDTLAAAAAALEGCGGDRAPGAS
SFGVMDVFREAAQNICLSNATSAAPSAAGLLAANGLSAVPSSLPPLSPGALHGAAASASRQPTGVAATPFQRTP
RVGGVSAGSGCPQPAALVGPVQPMASRAFSSGSPGTPGSVPPATSAFAPSPARHPASSSMRRREAPCELTLQD
LFMQSPLTGAYLATPPTSSGGAPPSPPRHAASEPQTPQRPVPAAAAPTATLERLSVQLAMNIARLSRATPSGDLG
MRSSALSGLPTVAHGGSQASSVARLQRWRTYTEGLGRGVAAALSGLACGSAALHDVVSGGDGAPEQREDGLA
NVNHSSAGRRNYSSGSQPVSDSASGAPSGLESGSISGNGTTLSMRSSQGTAGAVAVLSDAEGAAGHPPAKTGG
VAGLSPTHDSALPPLPPTRSRGIPAAADTSASAATATTAGAAGSSGQAVLLPAPADPHLDLDISPRDLKLQADGLL
GAGAFGSVYRGRFRDQPVAIKVLHHLHLQQPMPGGSPGGPGGALPYKDKEVESFRQEIAILASLRHQNIVRVLG
GCAHAGRPFLVMELLPRCLHNVIHGANSRLPLSEVLRIATDVARGLRYLHPAIVHRDLKPANILLDATGTAKISDF
GLARYHLKPYISTQQPDAGSVAYTAPEGFDPAIGRLSSKCDVYSFGVLLWEMITQEHPWSGDSNVAIIYRVAVHR
MRLPVPADLAVCPPRLATLLEACMAYRPADRPDMRHVLGELEAMSTVAVRH

>A804|XP_007509473.1

MSSSRNNAAPPQNRRRGSHHHPSSSTAGGGDGGGGANTKTTNRDDQRDEETDANLYARLSEPLSDLQRLRLS
SIEKLNELVLNNANADGENASLGSDGRETTSGGGGMNENASLGGGRNFFRSTSSDDSADTGTYDDDDDVQNG
SCDSSPGSDEYDEANEERMLRQRLRLEAANNQSKKEQLKEKLESLLSLDQAVEDDTMTELDENGNVIAKSRGSS
DLLSNTDTNSSNESSLGSSGLNAVENNQQETPNTPTTTAEGTSLDSIPTQMPKFMIWLPTVPSKEGTLEKYTEIRG
NWFSNLMRGFGSSGRNDASWKLRHFVLYDNHLFWGKGFSRMYGYGTVLAARDAFEYGQTAIAIDLLLYPKKSS
RHGSSSTSTPQNLVEALTGACCRPEGYSHKIVRCETLKDKALWLEVLNRTAAMHRERAEKEQQIQEQREHEQQK
QRENGSNPSNQRKAITNENSAETTGYAIQPPSPRISSDDYQYGSKESKRFGKNKRESRDYFARTDGDRHQRHQL
SPTVGSPSITTDEESEESELESDEYDDDDVFNDNDDDSEYVENGRNRMHGGASSSGLKTHASMLKHNDSEDSLS
LDPQNGIPPSTDDRSTGSPASRNQPSSRGLVRTGSGSVRFRNRSLSPAPNQRSPGSTPRSARAAQKQKNGVFTS
SPRSELVRTSSSPSIGSKKPPPVSSPSGATKYSAAKPPFDPNARPKQSSMKRSAIHSAFSAAQQKVLDDANKITEQ
QQKEGESGMSTPPPHNKNLQNVNSPRTTGGSKKSVTFQNDALLEEVKKYDATPTNGKKKKKSHMFQNNISAS
AWAEQLERRSSRALIKAAGSWSISINDLIFGKKIGIGSFGKVYKAKWHGTNVAVKKTLDVATHNTIKEFAAEIRLM
RDLRHPNIVLFLGAVVDAPSMCIVTELMKRGNLHSILHDYDNVVRETVADNGRLRLQMATDCARGMSYLHSRS
PPIVHHDLKPANLLVDSKWNLKISDFGMSRIKYRAYLQKSNPELETAGGTPEWMSPEALRNDNVDELSDVYSFGI
ILWELITLNYPWHELKDPVQIVGKVAFLHHRPKIPSWVETEMEELLLDCWSRESCDRPEFVRILELLQTVTTPGA
WSLGKGDNALEVKRSKFSQYLQDEHLEAAVNAQDEPERNSFFGFGGTPSRSKESGSLTSVASSQYNEADDPYIP
GENERDSLYGDNDSDLSGKGQSKSSSTGGIPGHKRRESHENILAFRPHSLTGLTIKTPAPKPDGKEHDRPDDKGR
GLGALKIRIENKRTSMTSGTASGMTSTTTAARSATAGEDNNTPKNISLEELVKLDDDLDDDLDVDEDEDEDDEDL
ESEVSGFSQLSEAQQQKMNKFKEQQIKLKNTKNKGMSKLSEEAPSGKEEEEEEQPKTPRKMSSLFSDDDDDTKG
AEERVGEEGLEARETAFPSRSAPDAYGSGTGGKKKKKKKKSDEKPKEPSEGEERSKPMFASQL

>A805|PSC71778.1

MVLCGLLAASGAQAASAAPCAATQHRRLLQSPASPANAASGPIIETVEVDVVSQECIMLMNSYYDAAALGVVEL
AAPSANQADSARDCCALCHDSRTSCNAFQWCPEAQGCNLGSVSFPYRGCQLLDLSGYLNASVNTGAIKQDGP
GVPFTAGSPLFFSVPKLSGYDVDIGRNFGGKFNYTCAGSMVEKSCVIAGSAQEVSVACNADPLCLAFTWYPSGA
ASRPQNPLGVLKGGGNVQLSSNDTELNPYAAIYIKLQPYGASASGDGGGGPTTVVIAVVASVVGVAAVAMAVL
AVFDVRYRRLKASVADGRAKPAADGDSPGDDGEGSGDTPGLDLSSAGGASSLAAAGGHSPAGSYSSQCEDEAA
LGRSGSPPLAMAASSGSGSGCGVPPGYTPFMLLSPHHMAPHTGANVEVAVVDVSRSSSGGSRAAPAADGWA
AAPNGSWGAAAAGDGWEASRASPAAGAAALPLVVEVARTVGNPTSCGRGELPPSLQQAGTARELLEVFARM
YSQRPAVDYAVVAQMLDQDADPGDVSAAERADEEALRRADAAAAQRRAASRTPSAQLSGSSGPPSGPRSQLH
AATLHAALQQQQQALQQQQQQGSYALPPAGAEAGAVQGLAALPGSAQLEQEQSLLPGMAGSEAASSMLPPS
EWSLQPEEVEICKRPDGTYWQLGTGAFGTCYKGLYHGSMLVAVKVLHRVESRRRGEEFEREVGLLKQLHDRNIV
QFIGACLDGPAPMLVTEFLEFGDLWRALPLTNPGGQRIFAWYKRGRRLLFDVAKGLHYLHQRRIVHLDLKSANIL
LSRHGTAKICDIGMARVLGNKNYLSMLSGMGTFAWSAPEVLAGKRCTEKVDLYSFGVVIWEVCTGDVPVRGE
MRALCAPADCPPEVVALYERCISEDPEQRPTAAELLQQYAAPAVQQREPARSAPRGVRGVRTMAHHVSSAVA
ALLTLIMLQGAAAAGQGTAETRPCGALPAAVRHRHLLHDALGPSPPPATAAVNDPSSQASNGTFINQLVDQKC
SMMHRALVSGTVLQPGDGNLQPDEVSCCQSCLAGEGCTAWVYCPEPLGCSAEGSRAGTAQNGTAAAAPAGS
ATPPGSADQRRLLQLPFQGCRLLSIPAFKLQRDSPQILVKGPEVQFVSGTPVSFDLPILPGYSVRTGKETVFGLGYQ
CEESLLLHSCLLKGLAEELAAICDADPLCKAFVYLPGGVDSLSEPVGIFKGGAGVESIDISSLLPNPSTATYIKDGAVS
LSGGSSSGGPLGASGGGGGSGTNALWIVLPTVLAVALAAAAAVAVYASLAMRRQARRLTEAEAALAKAQSRAP
RGGGDKQAPATGGSGMLTVHLLKPSPPSSGSSTGGSADGSTPRAGATSCGSADGGVPCPQCGATLGVSACRQ
PRQRHRHAQQQQQQHHHQQTGQQQPQPQQVVLAATSAPPNGAGSALAPP

>A806|GAX75524.1

MDQETEPYLFVAINNEEGDGEDDATNLTFTTSGRRGVRLKDVTPIFDSLDIEVLSYGLNKRLTNQSHYTVTSSEGG
QLEPELLTKLYKAVEAELGIKVVRGHPREQQILHKQNSHTAPSTGTRNSSDMSDGSFTLNQRKLSKTLSWDANTA
EACSPSSNATEFTPKPLLALQSINELSAGIQVIEDSAPSSPFSTVAWQDADSNSPSIPAWQESTLESESLESRPALG
NPCECEDESDRPFWCGGITPDDVVERIMSRPVRWISFEADLGQAKALMSMWGISALMVDTDSVNPGFITRQN
LLMASISSNLRRTKVKDIMKQPVVYVSPRIPIKRALQVMREKQVRRVAVFEAGIVKDDDPETWRAAWTGMVSD
TDIFKYLGQPCGPPEGIETQQEGFNQSGSTPQEESTAGVNCMAIPNNTQHAASGAVSHGYSMSSTSLTASFSSIS
SLANSINVGGSRPVRMVSMEERYRAAAALWELDFNELEIIRKIGEGSFGEVVLANFRGTKVAAKRLRGFDSSDEA
MPSPTVQPVLAQFFEREIEILATIRHPNVVNFIGACHTPPNVCLVTEYCARGSLDHLLHKSGLHLDTVKKTEFSLDI
ARGMSCLHAQHPPVIHRDLKPANLLVSARFEVKVADFGLSRIKDHAQLTNSRAGLEGTVEYCAPEVLRGEPYTEK
CDIWSFGVVLWELLTRQRPYADADVPVFLLMMSLGNGSLRLPHLREEVIGSDAMGLSLLCKRCMQEDPQDRPS
FREVLHVLEQEYKVIRGKAAAVPRSDSATSLRGCGVSQTSATPQLSVSPRVPRHLPRAISHNQSPIASRFSPLTPPV
MEVSAAEERGSGDSAQRQHSGPALASLEGKASPNGEMRKKRSMMNRSSSTPTPDQLWFDDEGELIMMPVIA
EGRSANGSERSSAHIDDAGSSSQHSEDRTLQQQQPLSRAPSIPRSSAPAQFPSSSPFAAMSSLQDAITRPTLGLSP
FAAAPSVDVFMMQTTASAFSPFAALNASYEVAQPGTTLDAAAAAAAATSLASAPFPSAEEVSLFASATSSAAPG
ATPFAAASSQTTADASATLPAAASSQERTANKADDALSPSVALNMISNKRCSNSGLQSISSDRNSHPSSSSAAAL
PQDKSRLSRPLKSDIAANCTDRSNIMPPFSVAGDSEYTIPERAASFGSPFSVQPSPALRDVTGPRRRSQIKSDLSD
GNLVKEISSALSPFSNFSCRSPFSQTCDADAVEDILDIQLCSITYDEVADEKHSSSINTPRTTKPVSDDSCALQNFQG
LDTLLPSRESSVCCWELGNTGIAGTVISPMMKAMADERQRSNAPSSAHFSDSTTAPVHAPSRLYADGASRVDSV
DQHICWLTIDGKQLPAEDGLFPVLSPRRSVQILNSVAVDPQKLQRPCVDPEVSSAVQLTLHSNAWVTI

>A807|GAX75441.1

MSRAPNPTWDGYIPPDGTLQAIGVPSNQSFPVSHVPLSVGTAGPYLAPSMPYQAYPGYSDQTTYDQGLLNYA
MAVSYSEHAARAVSQSQSVQTNRQSSREIGPKRDVSLKSQAEALSYKYWSTGSLGYTDPVIDGFYMTHGDFPEI
CDKDKFPSLEELKRVQTEEEDIREVVYIDLDQDSKLRDLKDRAAEAVAAQESEGASAKIEALAKVVSVAFGGSFDS
EDALNLFWRDSSREEKKVTKYVSILLGRLRYGTARHRSLLFKMLADPLNLRCKLLQGRYLSASEDTAVCFVNVNGR
EELVDLVYEPGRMLPPDQLAGCSSRSSSSPGVTGGTAAGYEASGSGSARSSFPPHRRSSSGSARLSSGSSRPSAV
VPLIPGPSYTRQEGQGSSDGTHAKLSIPEAREGSAFSVSGTRNPSAQRSVRSSLVPIVMPPSGHHAPSQPSTLTSN
LSGASNGSSTGGGSGAAPASPQLPPSQQQPAGLVQGPLPLHSAPLFKTQSDTTLKVSPQGPGRDMLPSPAGPP
PDLIRLDSGPISKAAAAAALEAAAAAAALQHQTLGASSASGNTNIDTSWAMFPAPNDSTASSDQAIHRHSKSFD
MTMLSSYDLTIQPDQPRNAMPPFARASESIGSGVPQLPSRWNAPLVTAGFQAGEGETTVTQASSGLSPIQGVH
LHSPSNTQDGTQLSSSYQSSSTLQFYPYARPPPPPLPQPQISPQGSLSNWTQGRALDTSTNNPSPSYVPGHLHTP
LQMQQVPSLTPKGSSTLMPPSPHQQSPISPPSVSPAVSHGGSLIASPAAWMQQQQQQEQQRLARSGIVVSST
AAASAGAPLSSSLTQISNHEAFADLSPFTSQPPPPRKTLSQQQQEKSTASVVSSSTSLSSVFNLSTNANMTGAKQS
GALGSEDFPVSISSMNSNDALKPGLSSRSSTQEGDATTSKPISQSSLTTAPSLSLQRQQIATQVGQQGAWAQPSR
TPTSAFEFQAASVLGGSAWGGANVPSLASAYQDAASASGVIAVSPWVAQPLHNHSGAIYQQQQQQGGGAAG
NAMAAPAVRASPFAQGMPYMTPLQQQPGHLDGNGELHEDEGLEPVLSRMPSLHLGAHNDWEINHRELTFG
PRIGIGSYGEVYKGTWRGTEVAIKRLLEQNLSASTVREFRDETSIMARLRHPNVVLFMGAVVQPNHLAIVTQFCP
RGPLYDILHRRNFQIDSKRRLSMAADIARGMLYLHSCSPPIVHRDLKSPNLLVDKDWTIKVCDFGLSRVKNETFLS
SKSQAGTWEWAAPESLRNEQYDEKCDVFSFGVILYELVACEKPWAHLRNRLAVNSLVATQHKRLDIPQDIQPEV
KQLMTECWEERPERRPSFAQVLQRLLKLKSLEPSLSQQSQVAERERAG

>A808|KXZ54209.1

MDLDKEAEVNGLLSAIGRQALAEAVSAVVGAGSTELQARTSAAAGVWPTAYVTAPAPAQTLRPLARIDAASTG
KDRGVPAKQPGALQAEQRTDRQRPPQLQVRPLEQETSLAFHISEVVPASPDGLRPSAVALVAGATGSTIGGSGV

GGLAGGGGGGNGYTPALTTSPASTIAGGDAGPRMTVSSSLPGEGSGSSFSAVEEARAAAQGQGSRYLAERAVRR
HGAPTPTRLHAESSFEAFGPHSGGHESGQLPDVEDNDDSRSDTTASSSIDATALRTFEALAQNAASMSAVPPHP
RSPTRGQPAGREGSRNGARRQLWSSPHLGPQEQLQQPLAPAPPSAEAPHVGSRGGTPVPELPQDGSQTLDKV
YRFLIARAGAPGAQPLTGMRLSRTQSDPFQLAVASYMAAQAAAAAAGATALASADVAQARAGRGRRRRASDD
ASAAEWLADQWGSTPVSPTVDRLTTSAGGAGAGTGFRGLGRGDRTVSDAQVLAGLLSSLPPTLPRRRRRLSVPL
PPELPAQSALLRAAAAVANERRGRIRRGRLPRDGPDGRDGESGADLGVYGALLLSAARMLAPPVASDAGLATSA
GGAAAAGPSAATASSAGAASPFTQQASAGHRGLRASPRLGDNGTSVGPFTQSAATAAAAASSGAAPTSPPSSS
PTAGAAAATSQGRSGPTGTMPPHHVPVAPSSLLQRRPRFTTDLQTILSGTHEEYSADVEPVMRPLPQPLLNHAA
AAAAPAAPVPLYGPTSALVASAHGSGPHVQPIRVPPHHPSHGEGYGGHELQQQQPQQQPQQLFSTHVGSWS
APGGSLGAPLPSSAFAAAVGSLTGSVAFSPPAAVLRPIRTTQPGDSQLRDAASGSASGPGSGQFAGSAIALIARLA
GTTGGSPSGSGSGSPATSRTVSERCLVSAGAALRGVSDRSLEVSGGGASLHRSRTAGSVMGRIGSGGGLSEQLL
MPAESVNLDLDPNDVLIYRDGLLGQGAFGAVYRGVYGRDKAVPVAVKVLNGSVVDGRAMQRDMASFHAEM
SILSRLRHKNIVHLFGGCMRPPHIFFVMQLMRQSLDSVIHHMHPPLSLRRALELARDVAAGLLYLHPTIVHRDLKP
ANILIDEAGVAKISDFGLARYKFKAYLSTRSTDQGSIPYMAPECFNPDIGGLGPKTDIYSFGVMLWELMSGEYPW
MGESNVTIIYRVAVSRERLPLPPADGTPWTLASSGSARSSGGGALSGRFSNGEPLVVPPPIRQLLEACFQHAPAD
RPDTHTVVEVLDSVLLSLDSGASSSDGGPGVSGTRGSTAVGASGAAVARGGDGAVASSAVEASDGGAQAATEP
GGGVQEGAGAACSAAEGAAASSAAGQQLATPFSNMAFLFE

>A809|XP_007511367.1

MREDPGEEEEEEEGESEYGTPEARVNNDAGVLVHSPPSSSSLESYPSSSVKKKKFASSFVTNNNDEEKKKKTSRTR
EDPTPKSPEYSTPQMRVNASNNNSVRVRGDGSNRAASPSMIASPANNSKVSSSPSSFGVTPTKRTNNNMNKSS
STRTSNIDVSSVQSLMLANTHAVNDLHKRVLRATSHREKCITLLSNAHALQLALVEANQTHEMLDAQSLTQLKKI
MISCVGLLRLCVARVDEVGNLSILEKAFTRYGILSSSSNKKFISLQSDLEIIETQLWNLTGASGGGIASKTQSIASLTR
HSRLLRGGFTTAMNKIIFGSGVTEMIACGVDCSELWVANETRFPRRSGILQVTDLFLNERRELENGGRLDASSSG
SSGSISSRNKSSSDAYSKSRGIGGCFRSLEEGDEYNDDGNTNAIRNGMSSLSMRVSEKPRGAITVMTKGSGACAA
LLFVGTSAGDVCVWDVDLGKGNENAVMKVRKGVSATAMSIVDDDEEESNDDEEDDNDNDLVRGEEENGTNE
VRASAFQKNKFQICTLWTGFSDGSILEVMAKVKIKSRRQSSSLGNSQNTSPGDSWQEKFDNEVDRIAGADFEVY
LCLGRLIRGEADGFGVSKRAVKFIVYLNGHVYVPYTNATATKYANENLEVWSTKSGKRIASNEVHNDLGLVEECV
AYKDAECVVTLHQAEGTFGSALGTVQLWGGRGIAPEARSAKINEIPFGSLACRVDIVEFASLLIIGHVSGHITIWKL
PSTDELDAYVTKRVDERSLNLGVPGKILAHRSGMVSMCGVHAVSGSHSGVATAGTFGSVLLWPMSELESAADR
AIKENGGKMRRKKRRQPSQEKLSAGLTSSRTVSQDLSPMLSKDRNGGSSGDIGKKFTHTPSEKRIASQLNTTLISQ
SEVALKQKIGEGSFGRVHVAVWNHVQVAVKFIGTEGMEESSDLRMAMDELEKEVSIMTNLRHPNIVALFGIMR
YPPAIVEEYCARGSLFSVLQRHAKPGVPSLQWRVRLRLALGAACGMCYLHNCTPPVIHRDLKSANLMVDASFRV
KVGDFNLSRVTAANRATGNSVSTSVNLHSPRWSAPEVLDTGDYSKASDVYSFGIVLWEILTLQLPWAEWSHWQ
VLHAVIELEERPEIPADVSPRFHALDKFIQLMRLCWSQKSVDRPTFETIIQTVQKMIESTEKFENEERLKFKNESGT
VGGAIAPRKSGSLPKSIKIFSANTNEGEKAPAPSSSRSNVVDTNKDVKSSRDDDVNNNNNEDDDDDAVVIIKDKR
KTSGVAPSNSITSATKNTNNDSLTSPPSQKSSSILAGIESRKSFNTRQSRVKSPYKSPSKILRGLKSLSKSADL

>A810|GBF95629.1

MKLKLTDKIRKYFGSAKERPGSAGDGGSGATRFAAALGGGAPADAKAAALAGPGLVSAKADLASNPRTTGNGC
GDAAAALSAEVCFPQLLETLAAAGPEQRAVAAEALFNFTAESDAARQRATAAGVVAHLTELLGSGTDHARMYA
AYTLSSLTSIDEAIAQMRERRAPAALVALLAHSPLLVCKKGAMRALGRLARHDDTAAEIVAAGGLRPIVGLLAHG
DASLVRRCLVALYFIAADKPDLQAALGAVPGAVPQLVSLLRSDSADVRAEAADVVKVISRSAACGAQIADAGGLE
PLAALAGGGGGGGGGGAGGGASARARASAARALQRLGDHPELRPALAARLGPAAAGGAAATAASRSVTSSAL

PASPGAHGVPVPVSPAALEEVAAASEGEDVRRLVEAVSRGSSWMRAKAAAAIEQVAADDPTASKDLAALGVID
PLLDLLERGTEAGRKGAARALGRMAEDPPVAARMCAPAVLPRVLRAAAAAPPPAQRVAAALVERLLLSERGLR
ALPAAQVVPPLLRLLEGESDAATHERVARLLVAVTGTDESGLGAVVADQPGFAALLQRLQQGPAEVRKPLLHLV
LSLTARRQDQQALLRSGVVQALLSLFKGGHGPAVTEVVSKALENLSQPATSILERTLSERRGSAGGARAAAAAAA
AAAAAEAAAAAEAARAAQAGAGPSSARAAQQETIMQRARSLSSAGPLAALAAAAAAARAGKVSEFEQQQQQ
QQQQQPQQQLQQPLAEQRQPLLVRKASGCAVAAAAAAVIAAAEASSVVERPLTPTRQLKTGTVAAVVAAVES
ASRHGAAPPAAASGATPRGDRAPSPGCGGAPSPLPPQSRGRSPSGRSSLQLPAAPPAAAPAPLSPGVPALPPRP
ISGSTSGNRKQQQQQQQQQQQSVQAQGQQHNALAAPQQQPAQSSAQQQPQGLQPAEQQQQQQQQA
QRQQQPQAGPGPGSSAAPPARGPSPARGPSPARGPSPARGPSPGRAPDGRDGAEPGPSGSSPAAAADSDDLL
SSTYDNLHGLLLRKTDIRICRAPDGRKLRLGEGGFGVVYKAVMNSVDEVAVKLVKTEKPSRQDLELFLKEVQTLAK
LHHRNIVQFYGACLETGSMFFVTELMKGGDLYTALRHHSETMRWERLGRKVALDVALGLNYLHSQRPPMMHR
DLKSPNVLLSEEGVAKIADVGMVRSQVKDLVTAQPVMTPLWAAPEVVRHERASIKADIWSYGIILWELISGEDIT
TYQPFSISRQMGAGASGRALSLPARCPAIARRIFTACTQMDPEKRPTAHQLVEWLRADVAGGPAAARASAGGK

>A811|KAA6424050.1

MVRLDWPWHRRRDEAHVPPAFEIASEEHELQVALALSQSANDATATRTRSEDEDLAAAKRESLHIVSHSSRAEA
LSYQYWDSNCLNYDDLVCNNFYDMWGEFQEATDNEQACPTLSALQRLGADTQDNREVLVVHHDDDAGLLAL
DEAAVEAVGNASARGPSAGIQALAKVVSHHMGGHGKSDAELMRQWQRVSRQLKRQHRSVVIPISALTAGLAR
HRALLFKVLADFCQIPCRLLRGQFYTGGGDDKAVVMVMCNRQEWLVDLVREPGKLLPLKPDTAPHQAPFLNR
MSGHASGLEWEEIESSEGPTLLSLQPSAPNGQASASPTQQLPMPVPVASTSLTNVHLRPQLDQYEDMARFLGY
SSSSKSQQDRSNHSQASSQSGLPSTTSSQLPAAHTNSLTPPSSHTGLYPNDHAVDLPANKPFRAASAAPASVPAS
APASAAAHAANQAGPSVSDYVNPWEGLQQRAASNSSTSSSSSSSHHSHHRTYPSPYQSTHHTHQQPQQSHQP
LQQDQRQHSQQPDRQFATPTARFAPSPFMANAEHSFVPVTFAPSPFMASAEHSFIPVAFAPSPFMADAEHSFV
PTPVEDPPQNAWEPFGSPTFPQAEVHSNGNHSSAQSQQAAASYTGNNASNSLSPPTDYFSYLDLLGTGRPTPQ
TVSQQQSLLHHWVGSGQFVDPQTDRQQHRQDRREPSQNHEQHPDERNQHQAHAQQRARGGPRQQPSQQ
PTGTSSDPLPVLGLNYHSAFDSFDDLDAPSEGESGYRSHSSPDGQLASRPRLGTSPRPLSPLRNAPPRQMEAHHY
LSADPTPESSDGSRRRAQRAHDQAQQSHVGLAKPLESVSLIPEHGAGSSESIRHVELTGAMEPDEWEIEADELEL
GPRIGIGSFGEVYRGTWRHTDVAVKKFLEQDLSPQLMQEFKAEVSIMKRLKHPNVVLFMGACTQPPNLSIVTQF
VPRGSLFRLLHRAGQHGALQLNDQRRIRIALDVARGMNYLHSCRPPIVHRDLKSPNLLVDKDLTVKVCDFGLSRV
RRSTWLSSKSQAGTPEWTAPEVLRSQAYNEKSDVYSFGVILWELCTGQEPWSDKNAMQVVGAVGWGNAQLP
LTDDMQPVLRGLIKSCWGQPQDRPGFGDIIAILKPMVQNTPIPPPPPMANAELVNIQRIQPMQSPMLSSANAG
ASQRHPSGMDERMTSAQSLPGRSSSHTMYGDMSMAAQHSHDPANAAGDKFAEVVRKVMHLANNAKNAVE
QHVGEQPGGLHSASSRMEPHAM

>A812|PRW05816.1

MVVTSGAALTLSKSSGELAGSFGGLGALSGAGNQLHSPAMNAGGALPPVGGPLVSHGSLRRRSKTEIAHQFLA
NLRARGSVEVDDTLEQEIVQHFESLPSRYALDVNISSLDVLNHKRLLDSARADPSAVSFQLRPVDVVSGSDLAKRP
SFGSLDTLQLQAAEALSNFSPHKLGGRALPRPAFGSSPNLQALVLEAEEKLEAQGAGDRASLAAAATTFYEVTIAS
IDQPKLLSRLSESLGDLGLNICEAHAFNTKDRFSLDVFVVNGWAGGGTEELEEVLSRRLQELPPPVVRGASGSPPS
SAREQELRVPQEELDVLARQATTGAADNDWELDPNEIIFHEKIASGAFGDLFRGSYCGQDVAIKILRNVHEDSQQ
FQEFLQEVAIMRKVRHKNIVQFIGACTQKPNLCIVFEFMSGGSVYDYIRKSGPLRVSTVLKIAVEVCRGMDYLHKR
KIVHRDLKAANLLLDETGTVKIADFGVARVMDHTGIMTAETGTYRWMAPEVIEHNPYKEKADVFSFGIVLWELL
TGRIPYSDMTPLQAAVGVVQKGLRPPIPPNCPPPLSDIMRLCWQRDPNVRPSFEQLKVKTEELLEVYRQQDGA
GGVRKVPHATAGAAAAPVASGSSGGLLARLRGSSGGSHSGGGSSSGRPNFERKRLGGRAEEVVIEGVTVELGAS

MIWNKNYLMADLAQAAGLDREDLAEEGLLAVYDGQTLVFEESSWSLLTLIRLILRYWFSFWEFRTAPAAAFAKF
QGIYQLLDSGTTFDRPDDLLKATGLYEYTQASLFEWVRETYGVGMDQLRAELMAGVVKCDYNQHTLDVNALA
GMVALLPATDPGVWRIRGGNGRLAPAVLRAANATVRCPAAVSAVRRLEDGRFQLELGPQQDQGPEHCGADQ
EQQLAGQLGAEGPFDAVILATPLESSGLSLEGLPDKPLIPARKYQKVVTTIVKGSVRPSYFGLAEMVYSTVLVTEDA
RLPWNSLSLVGRTTPGNATLWKLFSLDGMPHAWLELMFERGFEVVAERVWDAPGAYPAFDPPEDYAPFKLAN
GLYYTAALENAASAMEIAAIEGRMTALLAAQHLKSRAAAAAGAAGQQRAQGDGAVLRPGSEGVEQPAVAAAA
A

>A813|PRW57005.1

MARRRAWAAQRLALSLLTLAAWLTPSLAAQGSTQTDQTRLLAQSYAITNWDAYKAANNISGWVDDNSVPVCL
WTGVTCSPEGRIEGLALQCPNCAVKALGTIAPNTSEIQTMKTLNLQQQLLSGRLPPQFGLPTAFQNLFALILQQN
QLTGTIPKEWNVAGTFPRMSLLAMHYNNLTGDLPEPELWPQLGVLRLQHNQLSGTLPASWPTSLPNLQILSLH
QNQLRGPMPFDWYKPGTFQKIEEIYIQNNQLTGEMPANKDVLMSFPRLRIMDMSFNKFTGGLPPGWGMKNT
VPNMELMYIRGMGLNGTLPPDWGSDGGLQKMRQLWIDGNNLSGTIPAEWTKLPSLEYVYVKPGNLKLCGPLP
SGMNFKLCDASGNSECNIPAALDGPDCPPWVPPEDPTASTNTGAIVGGVVGGVVGGVLLAIAGVAAARWNRR
RKQRMSPFIAEAHGAVKPAKDDLSNYNTVLEALEGGADPDLDAEHRSWLGRQIDRCFTCGSTVRLTSDPAESAG
GTGSSSLPPLPPSGGGGALLGPQSKKASASTEAGGAGGMLFTNDSMLLSQEMSATSQLVLQQNPRLSNNSTVE
EGGQSGGGEEARDHFATDGLATLSDWEIRPEEIEYMKRPDGSDWELGTGGFGKVYKALRNGAQPVAVKVLMS
GPETRYMALNEFRREVAILKACRDPNIVAFLGACMRDDCAMLVTEYCEGGNLARNIVASKVTWYRRGRRIALDI
AKGLVFLHSRRIVHLDLKSPNILLSRDGTAKIADVGLAKIIAQEYSAVTGAVGTLAWSSPEMLLGARCTERSDIYSY
GVVLWEIATGQTPIRGQLRDVKVPEECPDELRKLMLDCLEQNPRRRPTAVQIIERLRKIPVSPREHAPALPQGSTP
TRGMMVPRAVTGAAKPRVPLGAGAAAAKTAPARAPPGVPPVVAEGEGISPFAMAAAGAEAAAADMSRLASN
AGDSIAGKPSGTVLGSQEDSSLALAPSSAMLAPAGSLPTPFKVVQQQQQQQQQQQERQAQSELGTQLPSSE
GSSDLVSSSRDPGSTAMSRAGSGAVLSPSRKGSLPVRPASRQPSLPGGGLPPGMPRPPPSPFAAAGGSSLSPGQ
QPSSPQNGNRETPR

>A814|Vocar.0017s0004.1

MGTFLGGFRNLFGYFTSLHKAAEKQDLAELRIQVQKHPSDVDVQEANMGWTPLHVAADKGFDPGVRELLARR
AKPDTQDKDGRTALHLAADRGYRKVVEDLVRGGASVSIRDKLGRTPYDNAVQRHPTIAALLGGPSLAPPMQQQ
QQPQLAQMAATQQRPPPPPPPSIRASPSSSGSLPSVHRGQLPAEPLLPSPPAAAAAAATVAADPSHLTAPSPAG
PSPPDMYDTGVMAAPQPYRQPYVQGTPTHQAPTALLSYPVVPGVWQPPGGGSGSGGVGGTCTQAQRGEPP
QVPLHPPPPLHPPQQSQYPLPPEPPKPESYAPQVWPRPPATGAAMESGSADRGVIRGGGGGGSGGGGGSGG
GGGSGSGGGSGGGGGSGGGGGSGGGGGSGGGGSGGGGSGGGGSGISVGVEGTRGSVSAAGPEDLDWGGP
SAPSGWGGEPDTDLAPPPSAAFNRPNFNPIASARVQLERLRDGGLALLGIAPPRPGPQPATAPAPGSLPASSDLR
DHSASAAWTVSSVSSGASTTLIGCRTYSYEELQAATGNFNPLNKLGEGGYGPVYRGLLDGIPVAVKVLDTSAGCL
QGRSEFESEVRILSSLHHPHLVLLIGSCPDRAVLVYELMRNGSLETHLFSAGDGSGGGGGTAASLPAPATASSSAA
AAAAAAGGAALRAGPHTVGLTAGLSWQDRVRIASEVATALLFLHTSLPPVVHRDLKPANILLDAHMTAKLGDV
GLASLVPTLARPAGHSAGAVQGISCGGGGGGGGGESTVLDSRLVGTFEYMDPEYLHTAQFSPRSDVYSLGMVML
QMLTGKRGKQVFSQVEAERRDPLGFGPCIDPRAGTWPVAEAAAFADLALRCASPSRQDRPDLRSVILPTLMQL
KQRTRLYDQQPPPPQQLQQEEQQQDVNVPPMFLCPITQDIMDEPVVAADGYTYEKLAIAEWMRRSSSSPLTN
LPMKNTNLVENRTLRSAIREWRERQQHQQHQHQQHQQHQQHQHQQHQQHQQHQQHQQHQQHQQQQQQ
HQHQQQQQQQQQQQAKEESAVAQAAAQAPPPPPPPPLPPPPPPPPRPVARADSGGW

>A815|QDZ22108.1

MTMRTRGEWKRLAAVVVLLASALPLHATEAAAASEDLQTAQPLSTTRGGNGTATNDEPASAKPVEGGIISDDW
VMSVVDANLSSLVWPGEESGYPTAPANPEAPVLVLEAQRFGYLNMRLKRKEKPVSGFTHVCLDVMVAENTDQ
PAPPQTILTLTGYNQRPQASASVSAHTVLDGNPEFAANAWSQVRIPLPDALDFSWYEVRIEEKSVQGSIVFVQKI
EFCGGEEGLDSSGYSQCLWRTEGEIDVPAPASATMPFVEAVYLSGFGLAPRWTDRSYIGFYDFSYILPSGEAAVK
ALVGPNGALSFATDASFEFWEALHFQILASSNEVDVCLEVSRENESRKEAQCVESLWNQSQLTGTWVEVSAPLS
DFGFPRPWNRFDLVDGSDQGIFFIVTNILFNSKPNSSDSGELWVLDLSEMSELEASLLDLDYSQGSGNSHVLGW
SLGIVIGVVTALILVPILICIIFPKQPGKRLTSVPLCCVALHESFQEAIDDVSAYRHRFRIPATVKMKAANKKGWVES
VTYREAADPDDGGGPLESTNIDNILPVYGVSKALSHSPSKASTRTASRQTLPCDTARVSPLPSERVEELMYHTHCG
GSIVEEDVPQTVRMERQPLYKVSSISEGEPTSRGLFKHDTEIMPSPIVVPTNRSEPMSYRKFSNLQHVDDALQAS
CKALLKSSNDLKKLLDFSSPQGKARGKHLRKSASEVIDMSVASDYHELIEKQIQSLQTAQKLVAEEWRVMKEVVD
SARSEQTPKRHAVEKIPSRTDVESMNVDIFKDVQITHLIGSGGSGSVYEGTYKGHRVAIKLLHNNNELNADQVDS
LKTEVALLQNLRHPNIVNFLGCCLNPESLCVILEYAEGGSLHSMLHTNQKQPEYGTLLQLAEDVASAMDYCHSLE
PPIIHRDLKPQNILLHRDGRAQVADFGIARIRTNTFVETKHLNAGTVAYMSPEIMQGRNVDEKCDVYSFGIILWE
CLTGQKPWADKLLPMQVVVAVGVEGDRLPLPSGCPISLKRLIRDCWRHDPRLRPTFREIKLRLAYLRNKHGSAH

>A816|59318

MDDVRPPPRHNPGNSALSRVNSGSLGNFHMNREIAAPDPPVKKERKREVVKRLIANLSYVEMSVVKATAPTDG
PPKSKHTRRLVDESWRSPAAAEEAILALSRCPLLNSPIITLKVLSTVHELMQKGSPSVLPATCQWLDHFMLVEAH
WGPGQMGGEYGLSQVCGRLIVAYAKMLSAKARFHKDFRAFENNYSFDAERSTNARPDPVSAQSLSAMLSLGS
CCREALDVAADILPRDPPLAALQWHRLLAHVGKLVAVEAHLLHGAAVYVAATLAEYGAGGLKNAGPGGRGEG
GLPREEARRLEMEHSALRATFVEARARPAMMLAFVEEGGDPALPPGWLGEGEGGERFGFLELPASLPNFDSKE
GRAAMASMIASSFGPPSSHTPPPASAREVSPFDAYPSDDEYASASEGGNERERGARDDGGASAEDFGWPVVFS
PGGVNERNAPTAGAPLIDFDDGAGWVSAAAATTAATPRPPIFGGYSGSTSIAGGTVPGGGAGRGGAGMRSGV
ATGGHNRTPSQEKKALDMAASLAAFELTAVRPTTQQEAVFDAKAPTMFKDIAPAGINSQRPPPTAGPPMSMN
AASRTNGGGGVAAAMGGWGGNGGGWGGQQQQQLQQQPPPQPQQSQLQQSQQFVSARQQQPPAPPPP
PQPQRTQAPMEGWANFDDAPTAPPPPIPMKNRPQNLNIIPPNFAPAVPTPRAGHQRTPSGGSPVPEASFDEIP
IEAIVFGKRVGTGAFGEVLKANYQGTDVAVKRLRLDPSQPQAAEDFRRELRVLCGLRHKHVVQFLGACTTGPDL
CLVMDFCSNGSLYGVLHNRRQNITAAHVLRWMADTARGMVYLHSRSIIHRDVKSGNLLLDESGCIKVADFGLA
RAHGPTSNLLTLVGTYPYMAPELLDNQAYNNSVDVYSFGIVMWECLTRDEPFRGHSPMQIVATLLRGERPKLPA
SPALPSSYVRLLMECWATQPERRPTFSAALDRLVGIAQAMRAAEENRHRR

>A817|XP_007509474.1

MSLQEFFKQTPMPVRASGILHGRDGGGQEFDSSFDGGFTANATTPGSGKKKTNTVSPFRGNDSASFSSSVSHLI
NGTIDFRVGFKVLQLNEKSTSNVGEMFVHLEDTELDKLYANKIIKRVDVLFGNEALGSMEMSSAPFVATFQTELK
GVDVLIKITFRSSLHRPPVKFTKSFEFFECAEGDARETIERVDFKPELIKKIIVVKGEESGEDEDGEDEGRRTSRVAK
MLSSNSSGSKKKDAAVAKRRFTYPNNNATATSNMFRNLDLESNNDDDDDDDDDEKSDREEEEKKSSSGSRKN
NKQTPADPNVWTVKDVGNWLESNMLGFLVSRFANSAVDGYVLLRLTDQDVLKDLRVTSHVERLRLFRNIDALK
ASRVPFEEKTRSLEKETRDKLNRTTASTNSFNTPVDLLEADVSWLQFVLPMRRLAVLSGWLSHVVDEVIERFNVD
VGSKRETIAAQISVYCVSAIKAELDIQESNADRSKQSTPTTARKSRIRESLETVKGWSKELELFDDPKTLDERQLQK
NLIALHLLLKEEEEEEEDVENHANNWMRSSGGASTPPPLLNAKAQSFIGSGTSMASLGELEPSSRSASPSNAHHL
RHVSSVMELAEECEIRYDDIEFSTGEASSSNRIGRGGFGEVFLGRYNGSLVAVKKLFESPVGKGLDEFKREVSVLST
LRHPSIVLWLGACTVSPNTAIILEYMDRGSLHDVLHRTEAVLTLSTRIRWSISIAKAMAYLHTHKPHAIIHCDLNCN
NVLINRDGAVKVTDFGLSKVKQHSKATRQTGVTGTVSYAAPEVIIGNQFTESSDVFSYGVTIWEIVARKIPWDGL

TEYQIVYRMSSALDNSDTEYAACEQHLKMNARSDEEQDQESSTTTTPASSIASTPFRPPLQSILRDCWFKLPERRP
KMGDILVRMIEVHKEACKEERTKRREKAKEEEEVGGATTKTI

>A818|KAA6427613.1

MDCFLCGGRRRTKRVTIPPGTNTLSAGPAAHVTQQQQALGPYAAVVPTSRQHPVQCHIYQYRELQAATADFSE
RNIIGEGGFGRVYKGRLPNGTLVAVKRLDRHGLQGDLEFNVEVSIMAGLHHAHVVPLLGSCCQADQRVAVFEY
MPHGNLRDLLDGKIAAARHFNWPARLNVLLGVAKGLAYLHEGTNPAVVHRDLKSSNILIDGAGQARICDFGLAR
HKMLLYSQPPPGPPPGGIMTGTFGYMAPEYATSGQTTDKSDVYAFGVVVLEMMTGMQAVDARRPTGCEALP
QLLQPALQAVQLMQGNLDPALNRHASLPAVQLAALCEVAASCLEPQPTDRPTAARLVTYLQKLLKSTDSANTPV
SRHGRTISSPPAGAPSPTHHPSASDTPRDYHQQPTSGSTSSSSPHQQQPQHRQQTHQQHRHRPAQSMPTMG
FTASPDWTADTAASTHAGALPRGHDLRRSAGYGNELGIPGPSRHLTPLALGLHAASLCSQPAASAPAASPAPEN
LATPGASSAALLPLAQEGPSRLPPMLPLQASPTRPDMRACRGEGSLLDSTKLAFGGGQAPRRAGLMEAGRDLR
DSAWGPSFSSQPPSAPLFPIRSSAQPANHAGSAQAVLRPSNSHRPTQSALPGQGKSHTQAGHLHTASSPYDQS
LHQLVQSRSMHPPIQSQDAPGPRSGQTAAQASLVSHGLGYDQERQLSRRPTRHQSGNPFASDSKADSTASGD
GMVSSASALLVSGNPFRSSVDRDALSIGQGRSGEGQQVVVDQQASDSVALGQGGSPTASCDLLKNAEMSPLQ
NGLIRAWPSPCKLQPPEVMARSWHHFLSASTTPHQAVSHTTKQQPSLSQPASQRASARVGVCLVCLDASRDW
QMVPCGHAAACKTCAEQLQQHRLPCPVCRAAVTGLCYRQFQQTYVGSAVPVNGHAVQQEQVV

>A819|KAA6426630.1

MRKVHAGVPAPAPSGSVAAPAPGTVIAANGTVLVPAPAVGTPPAGAPTPSLLTTSNASAQSPDAGSIAGIESSN
TTAGVPGLAPSPSTAEASAVPTPAEAAPAVAPSTTTSTGAIVPALAPVSSTTSQYQNVADAPASWNSSAYSTATV
QITVTNTSVTGFDTPTQTALLNALNSYLQAQGYSNFYMGLNGISGAVAVVQASPGAAAAAPAEGLPPGVSVTV
VVTPVDNSTAATPLTAQASPAPARRLLADTVTSSGTGVDVNITITAPASDLNNITALVSEAVQSGQVQQQLQAA
GVNISLTRVNVEVVIAGLPGVITVPAVSSPSPVAVLPPSPPAATSKSAGVPIGAVAGGIAGGAVVIVGGGLLTCLLC
KRRRRRRQWTKDLETAKAGYSEDPRGSSDSGSSANSGRPGGLGRKVAAYMPFGTNSNESVDATPHAKHATM
DDSFAGISRFKPDGTLNVHAPRLQLISSAAPAHQMGTIPERKSYSGDAAGVHLHDTANLLDSHSNSVQTAADHA
LLSPVSLFSFGTPVSRSGQASIVDPFRPAGMVSRPSEGKLSSDGSTNLMRGGSGDQLLGAHASGDYSDRFQELA
VEPIASRKGSNASGRQYSGNVDANLWSVAWKDLEIQKQIGEGSFGKVFLAKWRETTVAVKLLTYTGVSGSLEDN
DEFVNQGMNVLLKGLEQEAGMMASMRHPNVVMYLGVCLQPPCVVTEYCARGSLNDVFKRARATPALAAQL
DWGRRLNMVLDAAKGMMYLHSSEPPVIHRDLKSPNLLVDKHWRIKVCDFNLSRVMEESAILSSMAASNPRWL
APEILSGRGYTFSSDVYSFGVIMWEFLTWQIPWHECGPWQVVALVTEGAQRPELPAPEDLPGTGAFAGYSEYI
MLMEQCWDQDPAARPTFAQVITVLRKMLAAEMRAKSSNNAELNPQKSASPSV

>A820|QDZ24812.1

MKRFMRWAGGGQGDSERRDSSSGRNTNPNTPSPNSNQYSYNAEGDAFAGHYSALAESQQDAQSSLPPDPYH
QHYSQAQIFHHHRRTASRDSYDSFTKYGGTPNQSDNFLSDEEEYQTQLALAMSVSEQEAKQRDSMRDGLQPES
LTDGTMSGKRATNAQELVEKYWYTRRVHCDESILDGFYDCVGNFDSVRGGELSPRGGRDPADRAGSRPSSRQS
GSNLHKTTSKELPNLQRIQSDMVYLCASVEGMDERELLLVDRRVDEKIVALAKQAEAISSSGGSPKEKAIALGDLV
VANMGGPTNGTDGVGYMRQRNKWLGSSHQAKDNAKSYVVKLGDLSCGMSRHRALLYKVLSEEVQGLGCQL
VKGCFYYTDNDSDSLTGAPLLAEDEAKPAEGEGEPQLNLQATFAGLQLQAPTPPSAPEVDTSPYSVSVGKDCDN
GSVVVQCDGDHYVVDLMSQPVELIQLPSSDKFDTLGCTRSSSRGALRSTLESGTSSLSDRSWVQQELEATSQLVS
TIESLMRTNSMKDSGRDPGGKGTADPKGVQKSQSNRQHDALDELLKRNNRTSSSGEGSSNNSGTLSSGGGHV
DMGTSTDTSKSDATQGGLPKNQGQAEGKEKDSAQTQLSIDIIKESVGVSDGDSQWNIKVEDVTFGDRVGIGSF
GEVYRGMWRGTEVAVKKLIDQDITEESKQEFLGEVSIMRRLRHPNIVLFMGVITSRDNLAIVTEFLPRGSLFKLLH

RSGIQLDLRRRLRMAEDVAKGMHYLHTCNPMIVHRDLKSPNLLVDRNWCVKVTDFGLSRMKHSTFLSTKSNAG
TPEWMAPEVLRSEPANEKSDIYSYGIILYELITGKIPWEGLNAMQVVGAVAFQDKRLNVPEGIDEEVKELMQNC
WRGDPSKRPSFEEILKALRVVIKRTPVPTRKVTPT

>A821|GBG00361.1

MGNNLSALHSAAMEGDVHKLRVAVQEQPQRINEAEPSQGWTPAHIVAAKGYDVMLRELAARGAELNAPDKD
GRTPLHLAAGAGHLQVVQDLLARRADAGLRDREGHLALDLAVAGQHGAVAAVLQRATPAAALAAGPPPAAA
AAAGGGGFGYPSVPPPGGAAGPVSLNLPSAPPAPPGGGGASAPGAPPGYLDYLHGGAGISSPGMYPAPAYDA
GYAPAGAAAPGAPGAPAPGAGGGGAGYPAFSAPGAAGGGYAGPAQGALPTWPPRQQQQQQGGGGGGPP
PPPPPAAPQGPPPVLSRVAEADEDDDWGQPSISTDWGSSRPADGAGGAPVGPAVASGLPSYGTGPPPPPPPG
FGQPPPGGQLDMDAQAAAAEAARARLDKLTIVSKAAGAIGGAIGGAAVAGAKHLLGGLLNGARGGGGGGGG
GGGGGGGPQAPQIMRPGGGGSASSGSSGPVLSGSSGAARHYTYEELFKATQGFSEANKLGAGGYGPVYRGVL
DGVPVAVKCLDTSEGAMQGEREFLQEASILGRLHHPHIVLLIGLCPSCCMLVYELLDHGSLEEHVLGGGRGDLQ
WQDRVRITAEIASALLFLHSAPEPIIHLDLKPANVLLSRNLTSKIGDVGLSRLAPSLAPGAGASTVMDTRLVGTPAF
MDPEYLRTGRFGPKSDVFSLGVVMMQLLTGQDARSVITTVEAALRRAQSDPASFAAVVDPRAGAWPLVEAAS
FAHLAARCAALRRSDRPELRSEVLPGLLQLADRARQYATTPAALAAAPTFRRLASLGAGGSAALDEPPSMFVCPI
TQEVMEDPVFASDGFTYERDAIAGWIANHNTSPMTNLPLAHAGLTPNNALRSSVREWLDQHPKYAASAGLR

>A822|GBF98702.1

MKKWFKRRGKDGKQPPEPGAPPRRTSAPRSESGSDYTDGDDEPVHDFELAQEDYLVSVALATSANEYQRNGG
GAGGGFPLAMGAAGALELSRKYWATCRLSSRDAVVDGFYDVVGDFPEIVERDEFPTLAALRHVQLFEGDPREVL
LVNRLVDEGLCRVEVEAEEAAAQVSPFGEGARVQALASLVADRLGGPYASPERLARAHRDASAAVKAGGRSVIV
PLGQLRVGTSRHRALLFKVLAESLGIGCVLLRGQQYVGGDDEAAVVVRLEGQEWQIDLVGEPGRLLPTAVQQGS
TLGSAALGQLGQQQQHYGTAGGRAGVTVPMPASTSAPGGRAPRPPAGGGRQRAPGASSGDLISLNEPGEGSS
ALSRHASSSVLSQVSGMPSLTLSDDGALGQSPGAGGGGVPAPAPQRPRQQQQQQAGGAGPSRNGGGAAQA
RGGYIESPQPQPNMTFDVNHAGAWEIDPAEITLGPRIGIGSYGEVYKGSWRGTEVAVKRFLEQNLSPQLVQEFK
DEVDIMARLRHPNVVLFMGAVTRPNQLAIVTQFIPRGSLYRLLHRARADLDPRRRLQMALDIARGMNYLHSSRP
AIVHRDLKSPNLLVDRDWTVKVCDFGLSRVKSTTFLTSRSHGGTPEWMAPEILRNEPSDEKCDVYSYGVVLYELV
TGLEPWHSLNPMQVVGAVGFAGQRLQLPADLEPAVARIILACWKTNSRDRPSFGEVLDMLRPLKELPICGPAA
DAAVAAAGAGVGAPAADADGVGDAEVEPATEPVTDVGTSSGPGAAAEGGAAGRAAAAQADATTAAAAAAA
AAAAALPPSAVAGLPNVAPPAPPAVVAAGGGGGGGGGQQWQ

>A823|Dusal.0160s00006.1

MGSPTTEERRCKPAVVWTQLLDGTLFNASVSLAHTAIHMRLGPLVSAAEDLLPGICIISCGTERPRQGEQPPEDE
QMAFRWRRFPSSLLTLMHFNAITVQKESVDGRGTTFEPLDEDTCYLKPPCPSPVLRLGLQARGVSREDRFRAAA
ALWELDMSEIHFHHKLGEGSFGEVLLGSFRGTKVAIKRMHEGQVHKAGDEGPGTAAQSFEREIETLACIRHPNV
VNFFGACHAPPHLCLVTEHCARGSLDQLLHKSGLQINLARAIEFAIDIARGMNCLHEQRPAIIHRDLKTANLLVSA
RFEIKVADLGLSCVKDQARNTSSEAWLEGTVEYSAPEVLRGEPCSEKCDVWSYGVVLHELLTRERPYSDIDCSVYL
MMIKIGDGSLRLPPVPEKLATPGLVRLVSRCLSMQPGDRPSFRDILFHLEPEYKVAKGRAPAVPRSGSATSMLES
CKLQQQQLLRRGNAARTQRQQQGQPDQGQRHQQQSKLPTCSAFDPLPQPEGGPCCPPAEQHAAAMEARCA
SNELQQLHQATVTSQVSSMPPTRAQAPTPAYNRSPSVDLVHSEPVVKMKHKESEVQECMQQQQQRQQEQQ
QRQEQQQQEQQQEDMLMSKQQVLRDHQRVYFGLSSRFHAGLPRISEESTARSSMDTKRESGSSFDLPVLRPA
PSGVAQPSAAVPSPPASPVCSKQPRGDAHSPLNLATHTGFLHASVPQPCGPSNNASGIPAPSYPAGASSPFANN

ASPAEASSPFANNALPAGASSPFANNASPSPPHSPSPCPFSITPDPSQPDEPPVVSSSFADASPALSSHRAATLLR
NPSPNAVK

>A824|KXZ51279.1

MGNAGSTALHKAANDKDLARLRWEVQQHPSEVNVQEREHGWTALHVAAARGSDPIVRELLARGARHDILDK
DGRTALHLAAQSGYVEVVRDLLRRGASPAVRDKSGAAPYDLAVQRHPTVAALLGPPPPPPPAASPAAAAPPPLP
LQYGGMPPVDVASAASASHLLPAAPAPGPPHIHSQPPAAPPNPADLDWGPAAPTDWGGEPDVPGPSPPRRP
GFNPIMSARFQLEKLLGAGLSQLGLVGPAAGPGSSAGGGGAARADPRVGGGSGGGGGASGASAASGLTAASS
GALSSPGGRLYSYRQLYDATGGFSAVNKLGEGGYGPVYRGSLDGIPVAVKVMDCSEGALQGRAEFEAEVAILSSL
HHPHVVLLIGSCPEKVVAGGGRGAGRGPGAGHCEELDLIRVSPLCPPRPAIAMHASYCVRQGMLVYELMTNSS
LEVHLFGNGYGREAAAGAAPPVTAGGRPPLPLPWRDRVRIAAEVASALLFLHSAPTPIVHMDLKPANILLDEHLT
AKLGDVGLARLAPALAQPAGPAGAAAAAPGGPRSTVKDSRLVGTFEYMDPEYLRSGEYSARSDVYALGMVLLQ
LLTGRGGAQVVATVETARKQPLGFAPIIDPRAGPWPAAEAVAFADLALRCVEYRRQDRPDLRTVVLPTLAQLKQ
RTMLYEPQPAPAPSQPPPGATDGDVMDEPVVAADGYTYERLAIQEWVSRSSTSPLTNMPLPHSHLVENRTLRS
AIVEWREQQQGQQQGTSAGGGQQGQAGAAAAAARPAYDNSAGW

>A825|PNW75630.1

MWRTVLGFLDGGSPLPSHYRQTHRLAARRDLPDLEGQARDDAESDEEESMATNEDGGPLARRRSYSGSAGGA
SLEGHTAPLFPDSQQAQQAQQPQPQQQQQESIVVDVDGPSGEGGAGHAQPVWTGRASACGGAVDHSPIGV
VGTSGAHALAAGPGPPVQAAPASPINMGTTVLGPSAPLAPGAGMPGAVVVAVGRGGGPNPNNHDYNNNGA
VQNLNKGPTKAPGAPAPYLSDYATMSPDQGAGTAAGDATAELRATGAGADANKAAAAAGRANGGNGRRPS
HHQHQQNIPPNGALGGLYGPGLFRHDSYPPPLDSSKADMAALGGGARGPPIAKFSDLRFVQQIGEGGFGRVYY
GYWQGHRVAIKLAHPPSGSASAADLEHLVREFRREVEAMSALPPHKNVLQLLAACTEPPQLALVTDYCAAGSLY
QLLHGARVPGHSHLHPPWPQLLAICLGVAQGMSWLHRHSILHRDLKSANILLDNAGNARIADFGLAKIAAGSG
RQVMTGGLGTYQWAAPEVLAHQRYSEKADVYSFGMVLYECLTRKLPYEGMTAVQAAVGVVNHGLRPEIPRGT
PPAVAELIRACWAAIPEQRPSFTQIELQMMLLLDQARTAAAPPPMPQAAGAGGGSSGGGGTGAVVYQPPQP
QPQPHLQQLQAPHPHMQQQQQQQQALPPPMGPLGAGALPGQPGGGGLVIGIGPMPYGGGGGPGGGLGG
GLGGGGLGGGGGGAGGGRPHMPMRPSPPAPPMAAAGGMYV

>A826|RMZ52838.1

MNASTLLAALEMLESADDSSHADGAPTQVVFLPNGSISIPTPGGTSTTAPQDASPVVVQDLDQAAGSGDGDGD
FDDQELSRLSPEHGADLGATSFQSPGFLNGEEGTPATQPSVPVMTGASLVVHAPPAPPLPPARALPPAPAPGEP
SPAPEPSPESVGSATETVTVLSPETNPSREGPLPLPYPKSSVDGSGKSQTIAIAVSASVGGFVGLLLLASIPLVIWLV
RRRRAWLREQSRLSSGSDLEAGLGSGRRSSSPLAKPAPNDRGDGDGSLVTRPSTGMLGLLPLLGLGAREAPSRM
QFMVSGGGGTGRDPSRRGIGSSDGGARPGGGGPSGSASAQTPRSATARGGGGGGSSSLTDSVAPPSPSTGAA
PPPPAMVEDLLEDELAPQRSAASSQLWPGGSGALPWTDGRVSPEEITIALRPDGSPWLLGAGAYGRVYRALRD
GVQPVAVKVLTGMENSRRRDEFIREVTLLRSCRDHNIVQFIGACIQGEEAMLVTEFMEFGDLWRAATLRSPRGD
RIFGWWGRGRKVALDIARGLHFLHSRSIVHLDLKSANVLLMRDGTAKIADVGFARVMSKSYMLSSTGGLGTFA
WSAPEMLFGDRCSAKSDIYSFGIVLWEIVTGETPLRGDMRPMRVPEECSQGVVDLFDACLAQDPSARPDTRSL
VTRIQELLDEPVPGAAQGVELDAAGDRGVGKPAAAAAQGPGGSAAPAQVPDAARARADAPPAADA

>A827|0368s00006

MGGCFSSTPPQAPPHHAAPPEVKAAAAYAAPPQQSHVKETAELNKVYQQQGMSQPVPVQASKSECAPGIEN
AMPMENNRGFPSRQTSKKGERSSGTSQNTPRRGVSFAGNSDFPGDSSPDARMSRSRRASAPKRSPSFVGADL

HNIMDEQGMLHEGVSRQLSSMSHYSVRSESGNVIIEPEQPKAPFELLGLIGRGGFGNVYLGEWEGKKVAIKVM
QGNCAEMLQPEEQRWEARKERMAQMEAVLMSAIHHPNIVNTWKDGVPSFEWHIIMEFCDKGSLSRELASFK
FHRPLDDRNVGWDAWASLEVLKEVVHALKFLHEHKIIHGDLKAANVLLSSSDQDRRGWIGKLSDFGLSRVMSQ
DKGMITTRTFGTVTHMPSELLARGHLLPSSDVYAFGIVMWEVFTGEKVFKQLSDSAVVLAVVTKKARPTFPSDC
PSRYRFLAESFLCKQASSCPCALPLPGQDSPLIPCKVYPSRQNMLGQYIQYLASKEEAAQKAKEKAQAQAQAANS
SDPNLPARRSALKSGPSQKSLRINEARFGDSNSNLINGTSTREGVKRGVQDYGVQEQPLTSHKSMHARIAGPPS
DKEEEEEEEEQAPTGPRSLQRRAKSMVARRQHSFHTANVPKAERTQDEESESREGGKEESGPSRPNLGGMRPK
SQSFATLHSKGSTDTEPESRANATNDLPPPLDNNYIHDAQRVQAMSSQSGLPGQPPGSEQSLH

>A828|Vocar.0034s0092.1

MWRSVLSLLEPQTSPLPTHYKPARRPLQDLEAPPRPQLHDDDDDDDYPDFGHVEQHPLSLRPRRSVSAGGAP
DATSNFHQLTLQSEQPRDDGTSIVLDVDPGSTTTQPPLPPPQQQLLQQNDSPLPGAAASATGWQQQHRISGG
SGGPSEPSPTGTPTAGLVQLAQPGPSNASPKGLRLSDYATSPPDQGADATAELRALAGSNSDPQKAGPQQHGA
ASTAAVAAAAAGHAAGGGAAGTAVGGGRRGNQHIPPNGALGGLYGPGLFRHDSYPPPQDAARGVMEGPSR
TSSSRGPPVARFEDLRFVQQIGEGGFGRVYYGYWQGHRVAIKLAHPTTAAGPDQEHVVREFGREVAAMSALPP
HKNVLQLLAACTEPPHLALVTDYCASGSLYQLLHGPRPGGGCPPWTQLVSICLGVAQGMAWLHRHSILHRDLK
SANILLDNGGNARIADFGLAKIYNGNRQVLTGGLGTYQWAAPEVLAHQRYSEKADVYSFGIVLWECLTRKLPYE
GMTAVQAALGVVTHGLRPDIPRVTPHDVADLVRACWAAVPEQRPSFAQIEVQLGVMLQHARAAAASAAAAA
TTAAAAAGGGGGGGGGLSSASGTPRRGAVYGGGGGGGGGGGGGQMGLLPPPPPLPISPLEAAAAAAAAA
GGAAMALPLPLQHLRPVVAPPPAGAVV

>A829|XP_007514203.1

MSGSKSRPQKGSSVETSPMTLSSPQIPMAGSVQHQEKLRVCQKVIEQLCEDWPELNEDGERKAKVHDLILRLPR
QYGLDAHHLEDIETHVRLLERASTKDAPVVHVREVLVGGEEEEEEGSEGESDTDIGGKEEEEDANREDVSMSSA
NEESGQSARYKSQAKAILQQNHQQQQLQQTRGLRRPIFGSSLTLSSFQPGSPSSRGGSMSGRSIDNNTNNRNS
VEGTYEIAVCGGNKPRTLSRISTTLFDVGLNIAEAHVFCTSDGFVLDVFVAQGWRENDAKGLEAMLQSTFDQFN
WGDAVSSRKLQQQKKSGGAVDSAPAHPKRKSEDVSQKKNNGRDRRALIDDRSVSPMPSEWEIDEKLLTYSEKI
AQGAFGVLYLGQYCGQEVAVKVLKTPKNESHDDLKREFQQELSTLRKVHHKNVIQLIGAITKGPMLCLVTEFMH
GGSMLSFLHKNAPLKLSQIVKYSTGVTLGLDYLHKINIVHRDVKTANLLMDENDVVKIADFGVARVMAKDGVM
TAETGTYRWMAPEVIAHQVYNHKCDVYSFAITLWELVTGGDIPYSGYTPLQAAVGVVQRGMRPTIPQSCHPVL
AHTIQYSWQADMNTRPEFEQIVEMLRDINVTDDGKKDENNGLMSRFRSMGFAGSKKKQNKELKQ

>A830|XP_011396869.1

MLESADDSSHADGAPTQVVFLPNGSISIPTPGGTSTTAPQDASPVVVQDLDQAAGSGDGDGDFDDQELSRLSP
EHGADLGATSFQSPGFLNGEEGTPATQPSVPVMTGASLVVHAPPAPPLPPARALPPAPAPGEPSPAPEPSPESV
GSATETVTVLSPETNPSREGPLPLPYPKSSVDGSGKSQTIAIAVSASVGGFVGLLLLASIPLVIWLVRRRRAWLREQ
SRLSSGSDLEAGLGSGRRSSSPLAKPAPNDRGDGDGSLVTRPSTGMLGLLPLLGLGAREAPSRMQFMDLLEDEL
APQRSAASSQLWPGGSGALPWTDGRVSPEEITIALRPDGSPWLLGAGAYGRVYRALRDGVQPVAVKVLTGME
NSRRRDEFIREVTLLRSCRDHNIVQFIGACIQGEEAMLVTEFMEFGDLWRAATLRSPRGDRIFGWWGRGRKVAL
DIARGLHFLHSRSIVHLDLKSANVLLMRDGTAKIADVGFARVMSKSYMLSSTGGLGTFAWSAPEMLFGDRCSAK
SDIYSFGIVLWEIVTGETPLRGDMRPMRVPEECSQGVVDLFDACLAQDPSARPDTRSLVTRIQELLDEPVPGAAQ
GVELDAAGDRGVGKPAAAAAQGPGGSAAPAQVPDAARARADAPPAADA

>A831|GBF88923.1

MGDAPSSSAFGAAAGRPQQRRSKSDVAQAFLQRLQERGDIDLAVPGFVEGVCQHFERLPTRYALDVNIDSLDV
LSHKRLLEEARSDPATVSFAVRSVEVLHARHSNEPLPSPAFPAEPSPRRGQPVPPSSRSRPLPRPAFGSSPNLQAL
AVDLGERLDSTDGGDPGTPAPDSMFYEITIATVDQPKLLSRLSDAMGDLGLNIREAHVFNTTDGFALDVFVVDG
WSIEAGESLEEQLGQRLLRMPPPVVPARGRGPASGPNIDPAAAAAGGEGPASLSGWSSALPAEPPPPGFVSGG
GGSGGGGGGVGPGAWANRVPGGSSAPDDWEIDISQLHIDSKVAAGSFSNLYKGFYCGQEVAVKILKDLGDDV
AQYNEFLQEVSIMRKVRHKNVVQFIGACTRKPNLCIVFEYMSNGSVYDWVRREGPLRLSVVLKVALEVSRGMD
YLHQRKIIHRDLKAANLLLDDNGTVKIADFGVARTIEASGHMTAETGTYRWMAPEVIEHKPYDEKADVFSFGVV
VWELLTCKIPYSDMTPLQAAVGVVQKGLRPALPPNCPPGLAETMVACWDASPAHRPTFKELTPRLQQLLDAAR
EEEARGGGNGKGGPRGGLLSKLNLRGAGGGRGERS

>A832|QDZ23488.1

MGTRVVERLRESGIEVSSENERLVCDHFRSLPKRYGVAVKDALSEILMHISLLEDSRNRGGQIAHCLRAVVANLPT
KDPGNEVTCSSEAARGELKPSAYIPIETPGTTHRIVSATPTGKDSYLESPQRYGTSLSSLGSQDDLTGLAAAKESRTS
SNNGIAMQDCYELTVAMGSSPASLGTVYGTIATTGLLVAETHGFTTQDGYHLYWFVLQDQGRVTSQHLTALDN
LFHARFSLDSRGLFPVDAASVGKGAPAPSGGPGGKASAARGAGGAQRRASDSSDEGPEAVAAAAASAAQKGH
GGMRRGSSFLNLWKAKLDVQQTSVADWEVDPDKLLLSKKVGQGGFGAIHKGTFNGDVVAIKYIKSDFNKEFEYI
KEFAQEISIISELKHENIVEFIGACTKGERVCIIYEFMGNGNLKSFLQKHPKLPTQEKLKMALDICKGMVFLASKSIV
HRDLKAANVLMSESGVLKIGDFGVARLLPRNDEVMTAETGTYRWMAPEVIEHKPYNSKADVYSFGVLLWEIMA
DGKSPYELLSPIQAAIGVAKHGVRPKIPQSCCSQMARLMEQCWESDPDKRPTFEEIQRSLEDAMENVSKSEAAK
VVGKGNGTSSPKNSFTGLLSRILK

>A833|PSC75761.1

MAELPPLSHAGSLRRQSKQQIADLFLQNLRQRGNLDVDAPGSAEDLRAHFESLPSRYALDVNISSLDVLNHKRLL
DSARADPSAVSFQLRPVDVVSGSDLAKRPSFGSLDALQMHHESLTSSFSPHKLAGGRPLPRPAFGSSPNLQALVL
EAEEKLEAQSGGGDRQALAAAATTFYEVTIASIDQPKLLSRLSESLGDLGLNICEAHAFNTKDRFSLDVFVVNGW
AGGGTEELEDVLSRRLQELPPPVVRGASASPPASSREPEQRVPQDELAVMAKQASASTSDNDWELDPHEIVFHE
KIASGAFGDLFRGTYCGQDVAIKILRNVHEDTQQFQEFLQEVAIMRKVRHKNIVQFIGACTQKPNLCIVFEFMSN
GSVYDYIRKAGPLRVGTVLKVAVEVCRGMDYLHKRKIVHRDLKAANLLLDETGTVKIADFGVARVMDHTGIMTA
ETGTYRWMAPEVIEHNPYKEKADVFSFGIVLWELLTGRIPYSDMTPLQAAVGVVQKGLRPPIPPNCPPPLSDIM
RLCWQRDPNVRPSFEQLKVKMQELLEVYRQQDGGVAAGGIGGTAVRKVPQAGAAAAPPSSGSSGGLLARLRA
SGSGSSARK

>A834|GAX84415.1

MGSATSSNSSTWAQSNSLRRTQDNASLRSAASAPPVVQVAPRNADNAPSGHRMPVVTASSRNAVSSTMQGP
SGRNPASESDDGVRACCKFRLVDLSEATSGFSAANVIGEGGFGKVYRGTLLDGTDVAVKRLDPLSLQGDREFYAE
VGMLTRLRHPNLVCIMGMCNENGQKLGVFEYMPRGSLRELLDKKILSWRDRMTVAVGSARGLAFLHEISQPSV
IHCDFKSTNVLIDAYGQAHVADFGLARHIGGYRPPVKGLGSMPFASPDITTVTSIRGTYGYLSPEYLEKGHASVRA
DVFAFGVVMLELMTGLQPVDTSRGKGWEVLADWLRPKLRQPDEMFKLLDPSVSEQASAVQVAVMCEIAEACL
RANPRERPSMLEVIKTLSAVSRLNEASTLSLSVHDSMTDQASASLIPSPVGQVTAQPRKLTIPPARSSAGTKQSSN
SAGSFTAQPNNGIRGHHDATPHGSLVTDWSSQEPASGGLTKSKPPLAPLIGIVSGAEVQVSSNGISRPHTGLAID
KALPLDLDKPTGFGVDKAPSTAFAELHQRSTGNQDQKPSRSASRSASPGVTDGGGRLLTPSYQQPRGLPQVNIF
GMPGL

>A835|GAX76505.1

MTLLIRQHPSYFEPSTSELPQGCAPTFKMSVASTMLARKKTKLEVFQAYSKKLSERGDIDMSAPGFLESLRDHFN
RLPTRYALDVNIDSLDVLSHKRLLEEARSDPTAVSFAIRPVEVVVSRHRDSSQSPTISQSRHKEKTYLPKPAFGSSPN
LQALTIEAGEKIADEDMASPSGHNHDGEHMTFYEITVASADQPKLLSRLSEALSDLGLNIREAHAFNTNDSFSLDV
FVVDQTERQTNSNLEELLGDRLAQMPPPSGAASGTPQLKLPAEDPIASALSAMQQIPARPGSPAADDWEIDIA
QLQIDAKVASGAFSNLYRGSYCGQEVAVKILKDVQDDTSQYQEFLQEVSIMRKVRHKNVVQFIGACTRKPNLCIV
FEFMSGGSVYDYMRREGMLSLSQVLKIAADVSRGMDYLHQRKIIHRDLKAANLLMDEHNIVKIADFGVARIIETT
GHMTAETGTYRWMAPEVIEHRPYDEKADVFSFAVVLWELLTCKVPYSEMTPLQAAVGVVQKGLRPGVPASAP
PALGQLMEACWAQEPGKRPAFRDLTPRLQLMLQAAKDEESRRAAAAALAASKPVPSGGLLSKLRGKG

>A836|KAA6428177.1

MVTLSLSPTGPWSAGTFLSPPLTGLYGPAMSGTAHQSLRKRTKAEVSEAFIQRLRDRRSIDLEVPGVIESIQKHFQ
LLPTRYALDVNLGSLDVLNHKRLLDSARADPSAVSFQVRPVDVAVRQSSMARRPSFGNLDGLAGEATPYGSAKQ
GHPRSLPKPAFGSSPNLQALVLEAEEGAKDSVLSDTNFYEVTIASVDQPKLLSRLSEALGDLNLNICEAHAFNTLDK
FSLDVFVVNGWKGEGTDDLEEAMSERLQELPPPLGRHNSTSKQDGDGGFSFPQDMDTEGVGPSQVMSELQR
PSPPVDDWELEPSEIAFQEKIASGAFGDLYKGAYCGQDVAIKIVRNVQDNTQQFQEFVQEVTIMRKVRHKNVV
QFIGACTRKPNLCIVFEFMSGGSVYDYMRKGGQLKLSTVLKIGTEVCRGMDYLHKRKIVHRDLKAANLLMDETG
TVKIADFGVARVMDSTGVMTAETGTYRWMAPEVIEHNPYGQKADIFSFAIVLWELLTCKVPYSEMTPLQAAVG
VVQKGLRPPMPPSAPPALAELMQQCWQRKPDDRPSFEEAKVRMEEIWRVHRQDDAKRSQQTGLLSKFRKSK
G

>A837|XP_005850216.1

MSQASLPPVPPLASQASLRRRSKAEIAEQLLANLRARGMDVDSPGFAADLRAHFEGLPSRYALDVNISSLDVLNH
KRLLDSARADPSAVSFQLRPVDVVSGSDLAKRPSFGSLDTLQYQHELLQAVSSNYSPHKLAGGRPLPRPAFGSSP
NLQALVLEAEEKLEAQAGGERSSLAAQATTFYEVTIASIDQPKLLSRLSESLGDLGLNICEAHAFNTKDRFSLDVFV
VNGWAGGGTEELEDVLSRRLQELPPPVVRGASASPPASAQEVELRVPQDELDALAKQASTSASDNDWELDPNE
IIFHEKIASGAFGDLFRGSYCGQDVAIKILRNEVAIMRKVRHKNIVQFIGACTQKPNLCIVFEFMSGGSVYDYIRKA
GPLRVGAVLKIAVEVCRGMDYLHKRKIVHRDLKAANLLLDETGTVKIADFGVARVMDHTGIMTAETGTYRWMA
PEVIEHNPYKEKADVFSFGIVLWELLTARIPYSDMTPLQAAVGVVQKGLRPPIPPNCPPPLSDIMRLCWQRDPNV
RPSFEQLKVKTEELLEVYRQQDGAGGVRKASTTGGGGGGAGGGLLARLRSGGGSSSMRK

>A838|XP_002949123.1

MGGEDQNNFSTHDSGPMQDAPPLGSSTMREVQQLRPVQLSEQPHDLTPSITNDQVSSSSLESGPTVALIEECA
VEYGVPGWERLLTAQIAACSGMSTSRSRRAAPYCNATVPGGGDGGASLPFSSTETRLPSSAQAIFPFGDATPTPP
PSANADSNTQTFFVDNSTVPGSFSRPARTPCSMAAAAATTGSIPAMPPLSNLVKPPPSLPAAHCRSRRSSFRQV
VLPKELVSKAAEQGTDLRLDLRRDVAVDCSRTLGWGQFGAVYPGTFRGEPVAIKSVRHLLRDGCTIDDLEVFVQE
ITVLSSLHHDNVVRLLGGCLQPPDICLVEELCVTSLDLVLHSGAKSPLPLFRILEIALNVALGLEYLHSRTPAVVHRDL
KPANILLDKNGRAKISDFGLARCKYSAYLDTNRPETGSMAYMAPECWDPHLDGGLSDKMDIFSFGVVLWELVV
GERPWAGCKMSEFVHKVVTQGARLEVPTDDNVCPYALRRLISSCTERHPSERPTIRHITNELPAKERCSGTRSVSY
RVPSDDESDSEPEVTSAAHADTLSKPDAVEEQRQLLQQQLLASLEASAWLP

>A839|46664

MATARGDDDARAATTRREDDDDDDGTRARTRDARDAAMREELAKRFPTCDWREARAMDDACLVLVRAAVA
AERHGARALSCGARRVAGEDEDDASQRMARGGSNSSSASAGTSFSREAYELEQLTAAVRGAGLKTPEETEYKLV
FCGADVGGMLTRTMQVVDRIGLEVKDAQAHKSVDGMALNFFTVVGYEGEDERALEEAVEAALVNEESEEKKP

PVPLIINAAPPKSGVKRRRDARARGGEAFSPSTSAEHHGKSQRAAASFGGRASSRRSHSRSGSELSMNDSFNNT
ASATDTPGSEEATEEPMSEDNSDGALSVGSDIDSKKLQIGRKIGEGTFGTLYHGIYPSRTREGRVVHIEVALKYVTL
KRDDVKSARLDFFQEVKMLRTLKHANLVGYVGSVVEGSELCLVTEFMAKGPLLEYLRENGPMRKVEAIRVAVGI
TRGMTYLHEVGVIHRDLRAANVLLSGSFDAKISDFGLARRVPRNRSRMTAETGTYRWMAPEVITHGEYDVKAD
VFSFAITLWEIVTGGANPYGELNPLQAELLENRAAVAASF

>A840|KXZ52483.1

MASHGGRKRTKADVCEAFLARLAERGDVELDSALVDGIRQHFDRLPTRYALDVNVDGLDVLSHKRLLDEARADP
TTVSFAVRPVEIIAPRVEVSSPREALALEVGERAEAHDAEPSGSGAGRSAEEHPVFYEITIASVDQPKLLSRLSEALG
DLGLNIREAHAFNTNDSFSLDVFVVDQWTPQPGQNLEELLGQRLLQMPPPPPKGATPPPAAASTSYTPALRIPA
EPTPTFAQDPRLQALLQAAGPRPDSPAADDWEIDITQLHIEAKIASGAFSNLYKGTYCGQEVAVKILKDVHDDSS
QYQEFLQEVAIMRKVRHKNVVQFIGACTRKPNLCIVFEFMSGGSVYDYIRREGPLKLSAILKLAADVARGMDYLH
QRKIIHRDLKAANLLMDDNAIVKIADFGVARVIETTGHMTAETGTYRWMAPEVIEHKPYDEKADVFSFGVVLWE
LLTCKVPYSDMTPLQAAVGVVQKGLRPGIPPNCPMALAEVMEACWTGNPAQRPSFRELAPRLQQLHVIALEEE
KRQQESKPVAAKQGLLSKLRGK

>A841|XP_002954493.1

MASGRKRTKGDVCEAFLGRLAERGDVELDAVLLEGIRQHFDSLPTRYALDVNVDGLDVLSHKRLLDEARADPTT
VSFAVRPVEIVAQRAESALLHELPRANSRKLGLCRPAFGSSPNLQALALEVGERAEGQEAEATAVGSSRSADDHA
VFYEITIASVDQPKLLSRLSEALGDLGLNIREAHAFNTNDGFSLDVFVVDQWQPQQLARLRFVCLSSKHQPSCRC
APQQRHARVCSGGGAAGPRPESPAVDDWEIDITQLHIEAKIASGAFSNLYKGTYCGQEVAVKILKDVHDDSSQY
QEFLQEVSIMRKVRHKNVVQFIGACTRKPNLCIVFEYMSGGSVYDYIRREGPLKLSAILKLAADVARGMDYLHQR
KIIHRDLKAANLLMDENAIVKIADFGVARVIESSGCMTAETGTYRWMAPEVIEHKPYDEKADVFSFGIILWELLTC
KAGGAVPYSDMTPLQAAVGVVQKGLRPGIPLNCPLPLAELMEACWAGNPVQRPSFRELAPRLQALFTMALEEE
KRQETKPVATKQGLLSKLRGK

>A842|PRW61238.1

MGGCISTSSTQLDIRTVGDLRLLARELSRRPGIASRPRDGHGDTLLHLAALNGNPSSIRMLLEAGGQIDARDSLGR
TALHIAALSAHKACLLALLENQAAVNAQDGGGSTALAAAAFQGNEACLRALLTAKAAALDDSALAPEQPGSSGD
HDGEPHNGGSSSSSSPEAEPAATPAQMPPRQSNGAAEQLKSWELRFSDLTLERAIGQGSYGRVFLARLHETPCA
VKLLLSWQEGGGNGGGGNGNTGAFGGGNNAGGSAAVNGGGDGSSAALAGEQRHTLTLSSPLLADLRREAGV
MAALHHPNCVQLMGICADPAAIVTEYCPRGSLYDVLSTARRDPVAARQLTWTRRLHLAMGAAQGMLHLHTRT
PPVLHRDIKSPNILVNAAWKAKVCDLNLAKAAAGLSSQLSSAGALNPRWLAPEVLAGGGASLASDVFSFGVVLW
ELLTWQAPWSEVGGNPWQLAAGVISGKRPAVPPREALPGPDTAQFGGLDAYIALMQRCWATDPAERPGFAE
VVRQLRALLSAASRAGAASGSRATAAGST

>A843|XP_001697921.1

MSSGRKRTKADVCEAFVARLSERGDVELDSSLLEGIRQHFDRLPTRYALDVNVDGLDVLSHKRLLDEARADPTTV
SFAVRPVEIVGPRADGVSSPHEVCVGSTSESEKLGQLCRPAFGSSPNLQALALEVGERAEGHEAEASSGPGPREE
HPVFYEITIASVDQPKLLSRLSEALGDLGLNIREAHAFNTNDSFSLDVFVVDQWQPQHNVASNPILQRPHHAAR
NALTYRSPSPRPRRSRHDVQLAGPRPDSPAVDDWEIDITQLHIEAKIASGAFSNLYKGTYCGQEVAVKILKDVHD
DSSQYQEFLQEVAIMRKVRHKNVVQFIGACTRKPNLCIVFEYMSGGSVYDYIRRQEGPLKLSAILKLAADVARGM

DYLHQRKIIHRDLKAANLLMDDNAIVKIADFGVARVIETTGHMTAETGTYRWMAPEVIEHKPYDEKADVFSFGI
VLWELLTCKVPYADMTPLQAAVGVVQKGLRPGVPANCPPLLGELMEACWTGNPASRPSFRELTPRLQHL

>A844|147696

KRSLRERFLGAAISNRYSQSCSLNFTEAVPDGFYAPWGDYPEVWDEDADGDGGDGASGAGERERRLPPLALLES
IEPDDRTERASAIARAVANAMGGDVESDDAIASRWAETSYDLRSERGKLTIPIGRLLGRCGLRRHRALLFKVIADC
FDVPSRLVRGRYYCGADDAAANVVVVNGGEVFVDVMSAPGRMFAPDDSPASSLSFNKQRAAAAGVTGPGRG
GGGSGSGSGSIDPDDPIAALSIAVDLSIDAETIQLGERIGIGSYGEVHRGLWRGCEVAVKRFLDQDFSSALMQEFT
AEVDLMRRLRHPNVVLLMGAVTTTPNLSIVTEYLHRGSLYKLLHKPQPPAIKAALSEQRRMRMALDVAKGMHY
LHSCTPIIVHRDLKSPNLLVDKHWSVKVCDFGLSRMKNQTFLSSKSNAGTPEWMAPEVLRNEPSDEKSDVFSFG
VIFWELCTLQEPWNGLNPMQVVGAVGFCGNRLAIPEAESEEARGICEDCWRGKARERPSFLEIQKRLR

>A845|XP_002957782.1

YSYEELQAATGNFNPLNKLGEGGYGPVYRGLLDGIPVAVKVLDTSAGCLQGRSEFESEVRILSSLHHPHLVLLIGSC
PDRAVLVYELMRNGSLETHLFSAGAALRAGPHTVGLTAGLSWQDRVRIASEVATALLFLHTSLPPVVHRDLKPA
NILLDAHMTAKLGDVGLASLVPTLARPAGHSAGADSRLVGTFEYMDPEYLHTAQFSPRSDVYSLGMVMLQMLT
GKRGKQVFSQVEAERRDPLGFGPCIDPRAGTWPVAEAAAFADLALRCASPSRQDRPDLRSVILPTLMQLKQRTR
LYDQQPPPPQQLQQEEQQQDVNVPPMFLCPITQDIMDEPVVAADGYTYEKLAIAEWMRRSSSSPLTNLPMKN
TNLVENRTLRSAIREWRERQQHQQHQHQQHQQHQQHQHQQHQQHQQHQQHQQHQQQQQQQHQHQ
QQQQQQQQQQAKEESAVAQAAAQAPPPPPPPPLPPPPPPPPRPVARADSGGW

>A846|XP_001691431.1

MCMSVGAGRLALALSLPGADVTMEGTQGSGHAQCHDSMHTAYGTGHAPPQRLLQDRLGGGQESSLHSAAG
RANTLTTSIQNVDNNRKHLANKAASLVHRLQLSIRRRNPEDVSCQSTLEGEISTQTSVESYDRALVLGEAVSPEFG
TPGWERALTQQIAACTGLSTTASSRRASESAAQQQAPLPGFGQVVLPQQLLHKAAEHGANLHIDLRRDVTVDG
NRALGSGQFGTVFAGTYRGAPVAVKSLRPLMQGCTIDDLEVFVQEVTVLCTLRHPSIVQLLGACLQPPDICLVEEL
CATSLDAVLHRRDTIALDVALGMQYLHSRAPAVVHRDLKPSNILLDAEGRAKIGDFGLARLAYNAYIDTARPETG
SMAYMAPECWDPVLGGGLTDKMDIFSYGVVLWELCTGERPWAHCRTTDFVSKVVSRGARLPVPTNDNACPY
ALRCLISSCTEERPSERPAVAHIVAELQRMLKYTRRTADC

>A847|KAA6426300.1

MSRSRSQTHESRAGSRAGSLPGRTASQAARPLVTRLEEAETNLVAKQEANALLNSQVDLLTKQLKELQAGKATY
EKHFKDLSALLAKERRQKAADTTEERQNGEHVTDPQVASPDELNAATAERDEASWVCEERWEELRKLGQAGG
WLVDHQHVKLGKVLGEGTFGTTYMGRWRGGDVAVKCVRIQQRDEAESFLREVHVLACVRHPNVMPFYGACL
QPPQHCMLLCEYLPGGNLRDWLYGQGRSPPKRPLAERLQMALGVARGMAALEENDPPIVHRDLKPTNVFIDA
GGHARVADMGLARWLTPESMGVLTGETGTYLYMSPEMIRHELYNSRTDVYSWGVLLVELITQKIPYEDQHLTP
LQVALAVGDDQLRPALSSTTDQSLVDLAHACFEADPLSRPSFSHLVTELSTICSKAGKQPAQQQVQAGVLSRFM
KARPFS

>A848|108063

MDTGGATDKGDEREGKGGKESDEWRYYGRGEDMGASFGVLPGHEAKVDGGGFSQMPAPPGESPPRASPAG
TIAGRTSPAERQDSTESPEPSSSSSPASLLSIAVDLSIPAEEIQLGERIGIGSYGEVHRGLWRGTEVAVKRFLDQDLS
QHLMREFETEVDLMRRLRHPNVILLMGAVTKTPNLSIVTEFLHRGSLYKLLHRPQPPQVTAALSEARRMRMALD
VAKGMHYLHSCDPIIVHRDLKSPNLLVDKHWMVKVCDFGLSRMKNHTFLSSKSNAGTPEWMAPEVLRNEPSD

EKSDIWSYGVIFWELLTLKEPWNGLNPMQVVGAVGFSGNSLAIPEDARPEAKSLCEDCFRGNAKDRPSFLEIQK
RLRPMQAMITRPGSGNGGAALGGSPAQFSDSKPPASPSEPEKPPRAPPPKDEYPILP

>A849|KAF6255068.1

MPGATFGGPAGRVYSYDELARATQGFSAANRLGAGGYGPVYRGRLDGVPVAVKLLDTSGCSSQGPKEFAAEV
AILSRLHHPHIVLLMGACPEACGLVYELLEHGSLEQHLFSSSMPDLAWQDRIRIACETVSALVFLHSAPEPIIHMDL
KPGNLLLNRSLLCKVGDVGLSRLMPGAPGRAQLEGAPGGPRQPPQSTMLDSRLVGTPSYMDPEYLRTGRFGPK
SDTYSLGVILLQMLTGKDAGRVVSVVEAALARCQGDPASFATIIDPRSGNWPITEAASFAHLAMRCVELSRGNR
PDLRGEVLPALLQLAERASLYDSGRAAKRSVSLSISGQAPSMFICPITQDVMDDPVFAADGYTYEREAIAGWIAH
HNTSPMTNLPLAHTGLTANLGLRSAIREWQDKQGGRRG

>A850|XP_001692915.1

MAGRTYSYDDLRAATGGFSPINKLGEGGYGPVYRGTLDGIPVAVKVMDCTEGAMQGRNEFEAEVRILSGLHHP
HVVLLIGSCPDKGILVYELMPNGSLETHLANAGGARGPVPLGWRHRVRIAAEVASALLFLHSAPTPIVHMDLKPA
NILLDEHLTAKLGDVGLARLAPTLGAPSGPAAAAAAAAGGDSRLVGTFEYMDPEYMRTGEYSARSDVYALGMV
LLQLLTGREGAQVVSVVESARRQPLGFGPCIDPRAGDWPAAEAMAFADLALRCVEYRRQDRPDLRTVVLPTLM
QLKQRTQLYEQQQPTAASSPSPLGGDAVPPMFLCPITQDVMEDPVVAADGYTYERLAITEWVSRSPTSPLTNM
RLEHTQVVPNLTLRSAIKEWRQQQQ

>A851|KAF6253092.1

MQQQQQQQQQQQRRDQQHYQQQQQQYGHRQQQHHQQHPALSLVAAHAAWEIDPSEITLGQRIGIGSYG
EVYKAMWRGTEVAVKRFLEQNLSPQLVQEFKDEVDIMARLRHPNVVLFMGAVMQAHQLAIVTQFIPRGSLFR
LLHRSKADLEPRRRLQMALDIARGMNYLHTSTPAIVHRDLKSPNLLVDRDWTVKVCDFGLSRVKSATFLTSKSHG
GTPEWMAPEILRNEPSDEKADVYSFGVVLYELVTNQEPWTSLNPMQVVGAVGFAGQRLALPPNLEPHVAAVI
NHCWASKPSDRPSFAQVLDKLRSFKELPACPHPPATAAAADDGSGSFAAAASVRASSGGGAAAAAAGSGGGA
AVTSRQGSELAGAGSSSVQQQEQALIML

>A852|KAF6251010.1

MAVPIVQPCEVALATGGFHSLAGIGEGGFGKVYRAMVHNRPVAIKVMAEGGLQGQAEFDTEQRLLARLSHGH
LVRLLGVVRSAQPCLRCLLYELMPGGNVEEQLASEGGRRVLPWWLRLRVAAQVSAAVAYLHGLPASAGGALVH
RDIKPANWFLDGRLNAKLGDMGLALPLSEAGAAGSVRGGAGGREQAMVGTWQYMPPEYRSSGLIGLKGDVY
ALGVSLLQLATGQLERLAELLPRCREAVAGGQGCSLLDHKAGAWDEAAGERLLCLGLWCCQDTPEGRPSSQTV
ADQLAKLWLATDETLKAAATAGDGPVASAAAWAGSRTLGGQQQQLSAGAHHHHHQQQQQHLPPQRQQH
VPVVASSGGSFWRFLTGGAQ

>A853|KXZ51336.1

MDPAKAGMAREAAARGPPAARFEELRFVQQIGEGGFGRVYYGYWQGQRVAIKLAHPASGPDPGALVREFTRE
VAAMSALPPHKNVLTLLAACTEPPQLALVTDYCAAGSLYSLLHAPRPAGGGPPLPQLLSVCLGVATGMAWLHR
HSILHRDLKSANILLDNSGNAKIADFGLAKIYNGHHRQVMTGGLGTYQWAAPEVLAHQRYSEKADVYSFGVVL
WECLTGRLPYEGMTAVQAAVGVVNHGLRPEIPRATPPAMADLIRACWAAVPEQRPSFTQIELQVAVLLQQAR
EAAAASLGRGSVSSIGSSSGGGGVGSGGGGVPAGAMSPIRLPQPPQPQPGLGPLAAGAMGVMAGGGGGLGP
AGPMARAGAAGLW

>A854|20149

MDASVTSERFGGGVGGRGGGIEDWELLTTDVTVNELIGEGAFGEIRSGHWRGCPVAIKTLKTAVVTDQIAIKEF
NREMAIWSKLVHPFIVQFLGVGYKAGQPPIMCCELMSGGSLQRRLHDLKLEGKNMNFDEGFRIAQNIASALTY
MHSRRPFAVLHRDLKPANVLLTAEGVAKLADFGLSKMLSLYDHQFLMTGETGAYKYMAPEVFRHDFYGLKCDL
YSFAIVAFELFEGLLTLRDPVSWAHRATGEEALRPGWAFMAAYGTRRCQMMTQLVEQCWHPDPNERPTCAV
VSKIMRNIGRLSKYDKAEKSPGKAGGKRAGGPKGAKESGGAAPSKAEEEPAPSCGCVVM

>A855|20161

MVSGREKGVDDALSSDETLLLPALDDDFGTERGGEKRLQALRLAAGRWVIPPHELKLGRRIGEGSFGEVFTADW
NGTEVALKQTHDKVLSKDTAEELSGEIRMMQGMRHPNIVLFLGAVIESPRVSIVCELMPRGSLHSLLHGKARGG
VELSHNGRLRLQMAQDCARGMSYLHSRAPAVVHHDLKPANLLVDAHWTLKVSDFGMSRLKYNSRLKSARRSG
DASGDASDKAPGGTPEWMAPEGLRNEHSDERSDVYSFAVILWELMTLEYPWEELSSPVQIVVQVAFLHRRPRL
PTWLPTEAVALLQRCWNKDPNKRPAFTEILEKLK

>A856|KXZ52895.1

MARLPDFPAYGVAELYQATGGFNKLCLIGEGGFGKVYRAMINYTPVAIKVLDPEGLQGMAEFLNEVRLARSIQH
PYVVRLLGFTGDAGPGASSGGAGGGAGGGAGGTQCLVYELLTNGNLEDRLLRRGPSTPALTWPVRVKVGCQIA
DALSYLHSLGIIHRDIKPANMFLDCNMDAKLGDIGLASLDGWRAGKSVGGASRASDANAVGTWAYLAPEYKTE
GRSSPATDAWALGLCLLQLVLGRDPKDIIRTVQQALEECRLPQVVDSSAGTWDMKVAERVLKLGLWCCMHDA
RQRPPVSAVHQELARLVSLLQAQGLLEPGA

>A857|GBF97015.1

MDEALAASAGIRHYSPDDIASVTGGFNELALIGEGGFGRVFRGMLSSTPVAIKVMDGSGLQGRAEFANELQLLS
RLRHPHLVRLLGFCSGGAGSHGGGGNAGACAAALVYELMPGGGIDAHLVAKGGRASLPWCCRIRCAAQAGAA
LAHLHAQDPPVVHRDVKPANILVDGALNAKLGDVGLAAAARDGALTGAAREAGRDASSIAGEWPYLAPELRAD
GRHSLKTDVYAWGVSMLQLATGATDAVATLPARAREAVASGRGRQLLDPGAGHWDAFAGEQLLVLGLWCCS
DAPEERPVMSVVAARLARLAAAAA

>A858|KAA6419679.1

MLCWVSGSKICKLWLAELIVPQNPDGKVHMDLITQQGIQPLDVNICKNPDGTDWLLGVGGYSEVYKAQRPDG
QEVAVKVLHNADKVRLQMFAMEVKVLMAVISHPNVVQFHGACLRDNLAMIVLEYMEGGDLHEALMSVRCG
AQGAGLLSWNRQGAKLALDIAKGLEFLHARKIVHCDIKTKNVLLNKGCSIAKLADCGVSRLLDSHAFSSTGTSTFR
GTLAYAAPELLLGSKCTDKVDMYSFGVLLWEIITHEKPFRGQLRDFKVPEECSQSIAEMHEACLQLGPEKRPSAEN
AVLQIQQAIEEHEARQHC

>A859|Dusal.0080s00012.1

MSLNSSLKNLQLNSCGSTDVDKLLQENQELKRQISGNACQIPYEELDIQDKIGGGGFSIVYRGFWRGTPVAIKKW
FDPSMSDELMQEFREEVMTLQSLRHPNVLQFLGVCMKPPNLCMVTEHMPFNLHSVLYTSKTQLDKPKIIEMAK
DIARALSYLHSRKPAVVHRDIKPANFLVDRAFKVKLCDMGLASNSKSQVGAGTPQYMAPELLENKPYNEKVDIY
AFGVVLNELVSRMQPWGFMTPAEIKSNVLAGGRPELPLSCPRNIQEVITKCWHQDPAQRPSCDTLLDMLSKVA
KQL

>A860|XP_002957185.1

MPLPPSHRDLEINPEDLTFGQRIGMGSFGEVYRGTWRGTNVAIKHFHDQNLSPVTIREFRDEVLIMSKLRHPNIV
LFLGAVTQKNQLAIVTQYMTRGSLFRMLHRNKEVVLDPRRRLNMALDIAKGMEYLHNCKPVLVHRDLKSPNLL
VDKDWTVKVCDFGLSRFKNNTYLTAATQNGSPAWMAPETLKGEPCDEKSDVFSFGVILYELVTGKEPWEELNP
MQVVGVVGFSGRRMDLPTDLDPAVTNLIQSCWATNPKERPSFTQILATMNTWSELRPTAAVLEAQRAKAPQR
NAAQ

>A861|GAX76050.1

MNSAYQTLKVFSASELHQATGGFHKICLIGEGGFGKVYHAMINLTPVAIKVLDHEGLQGMREFHNEMRLLASIR
HPHVVQLLGYAAEGHTQCLVYELMARGNLEDILAGKGGNRETLTWPMRVRIAAQMAHALAHLHTKGIIHRDV
KPANMFLDCDFNAKLGDIGLASLDSGISNAEEAIGTWSYLAPEYKHSGRSSSKTDIYAFGLSILQLMTAASQTQDL
VHTCQIALEACNLPQVLDKRAGEWDLLAAERMVKLSLWCSMHDPEQRPSMAMIFTDLQRILRQLQQLGLEPL

>A862|KAF6257891.1

MPHQQQHQQQLVQQGGGAAVAQHAPPVVRGFVPRCEWELDPRKVLVGRRLAVGGFAEVFLGKYEGTVVAI
KRLLTNDAATIERFVSEVRMLARLRHPNLILFMGYCTTPELCIISEYMSKGSLYSQRQRRLEVLDPKIQRLVAVAVA
RGMAYLHTRTPPILHLDLKSPNILVDERWRVKITDFGLSRARQQTYVSASAQGGTPEWMAPEVLRCESVAEPAD
VYSYGVVLWELITGRAPWENYNPMQVVAMVGFRELQLPLPTAAAEPFLLDLCRRCVHRDPAARPSFTQI

>A863|5873

EIELDDIELGALLGRGAYGTVHRGSWQAEAEVVAVKTLHAVAGASERELRTFAREVAVLSRLSHPCVVRLLGACT
RPPRVCIVEELMRGGSLYDRIHGHGGGGGGGGSDGRRLTFVETMRVASDVAAAMSYLAREKVVHRDLKSHNR
GDGPERGVWAKVADFGIARAKGHTMLQTTRGATANGKGDAGTPAYMAPELFRGDKCDEKCDVYSFGVVLW
ECVTGRAPWAWLSNQMQIIFAVAVEGRRLPMREGECLASELTSLMFECWREEPRERPAFSHIEERVAAM

>A864|XP_005651898.1

MRSDLHYQVSLGQLLGEGMFGRTFLGMWRGGDVAVKTVRVGKESEASSFLREVASLAAIRHPNVMQFFGACL
QPPEQCWLLCEYLPGGNLTQWLHGDRKQGQVRRSLEERVRMALGVAQGMQALEAAEPPILHRDLKPSNVFLD
VSGRPCVADMGLARRLTPASAACLTGETGTYVYMAPEMIRHELYTSKADVFSWGVLLAEVLSQRPPYEGLYMT
PVQVALAVGDNELRPTLPSDTPEPLLNVALACYNPEPENRPSFALIVHHMRKVSSAGLPYVTISSF

>A865|XP_005844457.1

MLEGLEEWEIQPDEIVLGPRIGIGSFGEVYRGIWRQTDVAVKRLLDQEVSPQMLEEFRQEISIMKRLRHPHIVQFL
GAVTQPPHLCIVTQFVPRGSLFKLLHRTPAFNPDERRRLQMALDIARGMNFLHTCKPPIIHRDLKSPNLLVDKDLT
VKVCDFGLSRARRSTMLSTKSQAGTPEWTAPEVLRSQPYNEKCDVYSYGVILWELMTNEEPWHDKSAMQVVG
AVGWNDERLGTPEEGPPAIRELIDACFGEPAGRQSFSEIIPMLKGMIKAMGPPAGYEHH

>A866|GFH22806.1

MWCGPVGVQMAGQPWEIDPGELVFGQRLGMGSFGEVFRGEWRGTEVAIKRLLEHTLSDSSMRDFKAEVSILS
RVRHPNVVLFMGAVVQPPELAIVTEFVPRGSLFRLLHHSKAVLDPSRRLTMAIDVTKGLTYLHRCKPTIVHRDLKS

PNLLVDRDWTVKVCDFGLSQFLSSTFLTSRSTSGTPEWMAPEILRNQQSDEKSDVFSLGVVLCAQHSMGVVGA
VGYGGQRLEIPSDLAPGVQQLIRDCWKEVPAERPSSSQVLARLSEMKELAPSQIMKPANRG

>A867|XP_001697608.1

MVGASGAGPLVLSPRPRSEWELDPSKIIIGRRLAVGGFGEVFVAKYEGTLVAVKRLLATDSDTTQRFIDEVHMLA
RLRHPNLLLFMGYTLTPEPSIVTEFMSRGSLFHILRQAGDKVPEARMQRVVAVSVARGMAYLHSRSPPILHLDLK
SPNVLVDDRWRVKIADFGLSRVRQRTYVSSGAAAGSPEWMAPEVLRCDHYAEAADVYSYGVVLWELLTGKAP
WADLNAMQVVGAVGFARRSLPDPTEGDPLLLHLCKACRAYEPSQRPSFSQIVEAM

>A868|XP_003062382.1

DLSIDAETIQLGERIGIGSYGEVHRGLWRGCEVAVKRFLDQDFSSALMQEFTAEVDLMRRLRHPNVVLLMGAVT
TTPNLSIVTEYLHRGSLYKLLHKPQPPAIKAALSEQRRMRMALDVAKGMHYLHSCTPIIVHRDLKSPNLLVDKHW
SVKVCDFGLSRMKNQTFLSSKSNAGTPEWMAPEVLRNEPSDEKSDVFSFGVIFWELCTLQEPWNGLNPMQVV
GAVGFCGNRLAIPEAESEEARGICEDCWRGKARERPSFLEIQKRLRPLQGPIGGA

>A869|XP_001693263.1

MPLPPHYKDLEIDPKELTLGQRIGIGSYGEVYKGSWRGTEVAVKRFLEQNLSPPTIRDFRDEVLIMSKLRHPNIVLF
MGAVTQSNQLAIVTQFVARGSLFRLLHRTKEVLDPRRRLNMSLDIAKGMEYLHNCKPVLVHRDLKSPNLLVDRD
WTVKVCDFGLSKVKMDTFLTAKTQGGSPAWMAPEILRSERCDEKSDVFSFGVILYELVTGREPWEELNPMQVV
GVVGFNGQRMDLPPDLDPGVTALITACWADKPADRPSFSQILATL

>A870|KAF6254693.1

FEREIAILASIRHPNVVNFIGACHKPGQRCLVTEYCARGSLDQVLHKSGLVLDLLKRVEFAMDVARGMACLHAQR
PVIIHRDLKTANLLVSARFEVKVADFGLSRIKDASHLQVSRAGLEGTIEYCAPEVLRGEPYTERCDVYSFGVVLHELL
TRQRPYADQDVPVFLLMVNIGNGSLSLPDLPAEAATPGLIQLTGRCLAFSAADRPDFREVLSQLEGEYRSLRAAQ
QKQPRQQAAASAQQQQARLEQQQQQQLGQQAHPGQRE

>A871|GFH25143.1

MESLQMAGQPWEIDPGELVFGQRLGMGSFGEVFRGEWRGTEVAIKRLLEHTLSDSSMRDFKAEVSILSRVRHP
NVVLFMGAVVQPPELAIVTEFVPRGSLFRLLHHSKAVLDPSRRLTMAIDVTKGLTYLHRCKPTIVHRDLKSPNLLV
DRDWTVKVCDFGLSQFLSSTFLTSRSTSGTPEWMAPEILRNQQSDEKSDVFSLGVVLCAQHSMGVVGAVGYG
GQRLEIPSDLAPGVQQLIRDCWKEVPAERPSSSQ

>A872|XP_005848611.1

MLSRMRHPNIVSFMGLCTLPPCILTEYCERGSLYDVLQAAAKRPERAAALTWRLRLKMALDAATGLMYLHRRSP
PIIHRDVKSPNLLVDHSWCVKVADFNLSKIMGPQQPSVLSTSGGASNPVWLAPEVLEGGRATAASDTYSFGLVL
WELLTWRLPWAGMAPLQIMRLATSGQRPECPERGSLPGPGSEEFGGLEEYCQLIRDCWAQRPEERPLFIDVVP
RLRGLLESAAEQ

>A873|XP_002958672.1

PRSEWELDPTKIAIGRRLAVGGFGEVFLAKYEGTLVAVKRLLATDSDTAQRFVDEVHMLARLRHPNLLLFMGYTL
TPEPSIVTEFMARGSLFHILRHAGNRPPDPRMQRAVAMSVARGMAYLHSRAPPILHLDLKSPNVLVDDRWRVK

IADFGLSRVRQRTYVSSGAAAGSPEWMAPEVLRCDHYAEAADVYSYGVILWELLTGQAPWADLNAMQVSKTPCSNGCKFWKTSF

>A874|XP_013892099.1

MRGALQAAAAAAGFSDSNKLGSGGYGPVYKGLLDGLPVAVKCLDTSEGAMQGEVEFLQEAQILGRLHHPHIVLLIGVCPACCMLVYELLDNGNLEEHILGSRGDDLLWQDRVRITAEVASALLFLHSAPEPIIHLDLKPANILLSRNLTSKIGDVGLSRLAPSLVPGGGQSTVMDTRLVGTPSFMDPEYLRTGRFGPKSDIFSLGAPDTPARQGSLLAAAPQKRSWQPTTP

>A875|7730

EVEVLAKLRHPNLLLFMGYCVEPPLICTEFMRRGSLHTILKSGNVLEPARNHAVAIAVARGMSYLHSRSPPILHLDLKSPNILVDEKWRVKIADFGLARMRQTTQVSAKSEFHGTPEWMAPEMLRAEDFDERADSYSFGVVLWELLTARKPWMDLHPMQIVAVVGYSERKLELPPEGVPAADHDFTILLSDLFRACAQKDP

>A876|PRW33012.1

MESEQELITQDSAFCIQTAAQACSTADLQAQRPRPSLPATTSLASSMAAAGEEDALVVAADGSGGVRLASDIARDVTQLLEHDGLLEAGEVTGAGAPRLAIHRVTDGDDDDVVEIEVVHTIRAHLRHKPPSLAASAGASEQGSLAEELAAAATQSQGSSRSSLHDVKPGSGCSSLAAEAAAALAAAAAADPTAAPTVLHGGSHATLAARAGSGGSGSGSGTASTPSLRDRSMRHLSAVGSSLSLLAGSPAPQSLQPLAGGAGYDTLPAEQQQQQQQQQQQQQPQQAQQGAALPQSPFKAAAPGGTLAAVEAWEQQQAEELHREDRRRAAAADAALAPYEIDPRDILVGERLAVGGFAEVFVGRYQGTLVAIKLLLSVDERGQERFRAEVETLAALRHPNIVLFMGCCTQPYLAIVSEFMHRGSLFKLLRRGGNKPLEPKLQRSVAISVARGMAYLHTRSPPFLHLDLKSANILLDDRFRVKIADFGLSKVRSHTLVSGTGAGTPEWMAPEVLRSEGYDEHADVYSYGVVLWEVLTGQQPWAGMHAMQVVGAVGFQNKSLPPPTEGDPFLIGLCLRCLAPTPRSRPLFPQIVQELEHHYSRGPSFASLGSHTLSQGTIQLDTSDATAAARAGGARELPTMPAPPPPSLHGPPGLRLPARAVSRNGNKAAPGLGPVARRAYGGHEASPFQAESSAAPGSPDRVSPFASAQLPFDQEGKAPAATAQQAQQQQQLAQQQWPQQQQQQQQQQQPLQQAPAAVQLHPAASMTNDSPFASLAPFGDSDEEDGAAAPPPRLDGIPEAGSGSLSGAPTASSAGGGTEGGERSEASLSDRLRLCGLDGSEMGSSLEEPMQSQYLGASTLARATTLQQHLEETAAARPAPPAARLARQRSARELSPSAAQRRDSAAKAGGGGGSGPASVAAVGPADAKAALLHGVSSPRVPPSFPSVTEAAAWCSLDDAGL

>A877|Cre16.g659400.t2.1 g15929.t2

MSSGRKRTKADVCEAFVARLSERGDVELDSSLLEGIRQHFDRLPTRYALDVNVDGLDVLSHKRLLDEARADPTTVSFAVRPVEIVGPRADGVSSPHELTRANSKKLGQLCRPAFGSSPNLQALALEVGERAEGHEAEASSGPGPREEHPVFYEITIASVDQPKLLSRLSEALGDLGLNIREAHAFNTNDSFSLDVFVVDQWQPQPGQNLEELLGQRLLQMPPPPPKGGAAQQQQAVAAGPAPALRLPADPPPNLSMLAGPRPDSPAVDDWEIDITQLHIEAKIASGAFSNLYKGTYCGQEVAVKILKDVHDDSSQYQEFLQEVAIMRKVRHKNVVQFIGACTRKPNLCIVFEYMSGGSVYDYIRREGPLKLSAILKLAADVARGMDYLHQRKIIHRDLKAANLLMDDNAIVKIADFGVARVIETTGHMTAETGTYRWMAPEVIEHKPYDEKADVFSFGIVLWELLTCKVPYADMTPLQAAVGVVQKGLRPGVPANCPPLLGELMEACWTGNPASRPSFRELTPRLQHLNAMALEEEKRQLEAKPASKQGLLSKLRGK

>A878|XP_022839923.1

MGRTKQRGKTLTLGEFFAETSTQRTSGLLGARLARAVDGATDGGDDGDDDSYAEFTVEIGNASVRCAEQEGSYDVRAFAREGRNAGGKVIKSVTFVFGGKMGERRVTEAPFEVQYRCETSVDVEVAVQFHRALNARPVRELHAIELSSEEREFSRSFAVEVKRRALMKVLGKDKGDAEVDVRRAESRSSLLEESQSRGVDAWTVQDVSEWLRSIELEELVER

FAKAKINGYELLRLTEKDLRESLHLERNLERVRAIRAINVLRASAGAADAENDDKSATPPLSAPLGAPRGGLSPLEL
ELDVSWIEFVSEKARASVLIGWFMHVLDEVKAKEFDEPGPQLSVYCEATLQASKSCEDALEERVLDILESTPGWD
PRTKMFPSTCDLTKLNDQLMELYLEVKAFEEFAALNMDDDRGEHERSRSTPPKMPGESSPAIPRLNRAESLTTPS
VSGSPTFTSPRNDMGPSPSPSGLNVGARSFSPTPLSDLTRSSLPELQEDEVADGDRVMELNTEWEIDYNDIEFEG
GVPSSKNRIGHGGFGEVFLGRYHGSLVAVKKLFNQDMMGKGLQDFRREVRILSRLRHPSIVLWLGACTQAPNL
TIVLEYMEKGSLHQFLHRTTNPYTTLTLTRWGITIVQGMVYLHSAKPFPIVHCDLNTNNVLVNRDGMVKITDFGL
SKVKHSSRLSRQTGMTGTVNYAAPEVIRGGKFSEASDVFSFGVVMWELLTRRIPWEDLNEYQIVFQMTSELDAP
LAATAKNLELPSSSPEGFRKIIHGCWATQPERRSGFKDVLVHLREEYRVLVEKEKALRASRQGSSSSLSGAPPQ

>A879|XP_022839011.1

MSPVVDAFDADASANARDASTVPVVADAASPTFADFLSPEAQAFSSEHAGESVPVVRVRGTARSGSKLWGIAR
AAVREDGHASRDAAAAEGSRRRRRASAEYESFLRPILDRMPVKPERVSGVDGPLMNVTRIDDKTLDVTMTTSFR
AHFGQSIQAIGRRSPALALPEADGGRADVQREGKWGDAGGDGVQISEIDSGEFVPGRGGRYSVMTPEDSWNV
AVGDRRAPSPAIGWHDEEGSSSDALHTEFLINPDDVRLQERIAVGGFAEVFRGTWQGTVVAVKQLLERTSEVKE
KLEQEVQVLAKLRHPNLLLFMGYCVDPPLICTEFMRRGSLHTILKAGKPLEPARNHAIALAVARGMSYLHSRSPPI
LHLDLKSPNILVDEKWRVKIADFGLARMRQTTQMSAKSQFHGTPEWMAPEMLRAEDYDEHADSYSYGVVLW
ELITAHKPWEDLHPMQIVAVVGYSGRSLELPSEGFPESSHPLTALLADIFTRCARRDPSARPLFPAILTDLERARDL
ARARAEAAGADKTAARIPPRPDTESVDRRALGVQLEYISLADAVASTDASS

>A880|XP_022839010.1

MRQRETKEAVARRTLAEFETQIEETARRTGDATTRARVERMLKRLPKQYAMDVNFIEDVLAHAELLGRVEQELQ
AMGVSSVYCSVREVEVGRAAFGSHEDMMDTDGGIEQLNVTADNGIPIRADSPAGKPRGPTFGSSLQMTSSLG
GDPSRGSAGMYEVAVAAGNKPRLLSRVSAVLFDVGLNIAEAHVFCTDDGLALDIFVVTGWKRGEEAAVGHAV
QTALDAADFSDIVPASRNASAATPSADEGRMSAGSHGRSTSNDSVSIDGGEWELKESQLVFNEKIASGAFGLLY
RGSYCGQEVAIKVLKSNAQEGNAGNETMREFAQELSILRRVHHKHIIQLIGALTKQKTMCLVTEFMHGGNVLQF
VQEHALKLHEIIRFSLGVAMGLDYLHKINIIHRDIKTANLLLDENSVVKIADFGVARLQPTDGSTMTAETGTYRW
MAPEVIAHGFYNEKADVYSYGIMVWELESGGEVPYPGYTPLQAAVGVVQRGLRPAISTSCNPKLAQVMQSCW
LADATQRPGFEQIISLLKSIDTQKAETDGKHGFFDRLRSVSFKSKKNAPARSS

>A881|XP_001703525.1

MTLEQLEDRNSSHGGTCAGSNSCLTRGRRSSATETLALEELPSVGHLAEAGWTRALADNIKSVAAVTGVMGGG
SMPNSPHGSVLGARGSLTGVKYGTALGVGAAPNAVMAGFRPSGSGLKSSSGAGGLPGGPVGSATLVTELEEFD
GGDDVEAALNVVPDALMQQAVKAGVNLQVCMDTDVTLSSEQLGRGVSGTVVKGTYRGQPAAIKMLPPDLLF
GNRSLELHTFVQEMVVLCGVRHPNIVNLLGGSLQPPNVFIVEELCVGSLEARIHGGPGKNAAAPKALSAYEQLRI
AVDVATGLQYLHERTPAIVHRDLKPANILIDPNGTAKISDFGLARVKTHAVINTKAPDVGSIGYMAPECFTNEDG
QLTDKCDTWSLGVTIWEMVTRKRPWASCNMAEYYREVVIRKSRLPIPQVCP
