# Supplementary material for: An Ancestry Perspective of the Evolution of PBS1 Proteins in Plants
Source: Int J Mol Sci. 2021 Jun 25;22(13):6819. doi: 10.3390/ijms22136819 (PMC8269361; doi:10.3390/ijms22136819)
Supplement: Supplementary file 1 [file ijms-22-06819-s001.zip › Supplementary Legends.pdf]

Supplementary Figure 1. **Analysis of conserved kinase subdomains and important motifs in PBS1 orthologs in terrestrial plants.** Sequence logo representation of multiple sequence alignment of 765 sequences from monocots, eudicots, bryophytes charophyte, gymnosperms, lycophytes, liverwort and pteridophyte with high similarity to Arabidopsis PBS1 were identified by MEME analysis. The domains are ordered from lowest to the highest expectation (E) values. The conserved kinase subdomains are labeled with Roman numerals. Subdomain I: GxGxxG, II: K, III: E, VIb: DxxxxN, VII: DFG, VIII: APE, IX:DxxxxG, XI: R. The AvrPphB cleavage site GDK is labeled with a red star and the recognition motif required for RPS5 mediated plant resistance is labeled with a yellow star.

Supplementary Figure 2. Phylogenetic analysis of PBS1 orthologs in terrestrial plants. A total of 881 amino acid sequences from Arabidopsis PBS1 orthologs were searched for in different databases and aligned using ClustalW. Maximum likelihood phylogenetic tree was constructed based on the deduced amino acid sequences of PBS1 from terrestrial plants. Red indicates eudicots; blue, monocots; purple, pteridophytes; light blue, bryophytes; pink, charophytes; orange, gymnosperms; green, lycophytes and brown, liverwort.

Supplementary Figure 3. Ancestral state reconstruction in PBS1 orthologs of the motif GDKSHV. The reconstruction of ancestors was used the maximum Parsimony algorithm using as character ancestral the amino acid of PBS1 protein. Reconstruction of the ancestral state at amino acid positions: **A)** 245 (glycine); **B)** 246 (aspartic acid); and **C)** 247 (lysine) of PBS1. Font color in branches indicate the evolutionary divergence of the ancestral amino acid. The color in the names indicates terrestrial plants species. Transitions and reversion are indicated with asterisks above nodes.

Supplementary Figure 4. Ancestral state reconstruction in PBS1 orthologs of the motif GDKSHV. The reconstruction of ancestors was used the maximum Parsimony algorithm using as character ancestral the amino acid of PBS1 protein. Reconstruction of the ancestral state at amino acid positions **A)** 249 (histidine) **B)** 250 (valine) of PBS1. The colors in the branches indicate the evolutionary

divergence of the ancestral amino acid. The color in the names indicates terrestrial plants species. Transitions and reversion are indicated with asterisks above nodes.

Supplementary Figure 5. Ancestral state reconstruction in PBS1 orthologs of the motif SEMPH. The reconstruction of ancestors was used the maximum Parsimony algorithm using as character ancestral the amino acid of PBS1 protein. Reconstruction of the ancestral state at amino acid positions **A)** 296 (serine) **B)** 298 (methionine) of PBS1. The colors in the branches indicate the evolutionary divergence of the ancestral amino acid. The color in the names indicates terrestrial plants species. Transitions and reversion are indicated with asterisks above nodes.

Supplementary Figure 6. Ancestral state reconstruction in PBS1 orthologs of the motif SEMPH. The reconstruction of ancestors was used the maximum Parsimony algorithm using as character ancestral the amino acid of PBS1 protein. Reconstruction of the ancestral state at amino acid positions **A)** 299 (proline) **B)** 300 (histidine) of PBS1. The colors in the branches indicate the evolutionary divergence of the ancestral amino acid. Font color indicates terrestrial plants species. Transitions and reversion are indicated with asterisks above nodes.
