# Supplementary material for: An Ancestry Perspective of the Evolution of PBS1 Proteins in Plants
Source: Int J Mol Sci. 2021 Jun 25;22(13):6819. doi: 10.3390/ijms22136819 (PMC8269361; doi:10.3390/ijms22136819)
Supplement: Supplementary file 1 [file ijms-22-06819-s001.zip › Supplementary Table S1.pdf]

Table 1. Presence of PBS1 motifs in plant species

ID	PROTEIN	ORGANISM	Linage	Motif Putative Cleavage Site Motif	Putative Plant Resistence Recognition Motif
**A002|A0A2P5XSL9**	Protein kinase domain-containing protein	***Gossypium barbadense***	Angiosperms	GDKSHV	STRPH[b]
**A003|Q9FE20**	Serine/threonine-protein kinase PBS1	***Arabidopsis thaliana***	Angiosperms	GDKSHV[a]	SEMPH[b]
**A004|A0A0E0DQR2**	Protein kinase domain-containing protein	***Oryza meridionalis***	Angiosperms	GDKTHV	NTKPL
**A008|A0A446L2W9**	Protein kinase domain-containing protein	***Triticum turgidum***	Angiosperms	GDKSHV	STRPH
**A009|A0A0E0NFF1**	Protein kinase domain-containing protein	***Oryza rufipogon***	Angiosperms	GDKSHV	STRPH
**A011|101294724**	Serine/threonine-protein kinase PBS1	***Fragaria vesca***	Angiosperms	GDKSHV	STRSH
**A012|102595931**	Protein kinase domain-containing protein	***Solanum tuberosum***	Angiosperms	GDKSHV	STKPQ
**A013|Peaxi162Scf00386g00811.1**	Protein kinase domain-containing protein	***Petunia axillaris***	Angiosperms	GDKTHV	NARAH
**A015|A0A287MGV2**	Protein kinase domain-containing protein	***Hordeum vulgare***	Angiosperms	GDKTHV	NTKPQ
**A016|A0A0B0PYC3**	Serine/threonine-protein kinase PBS1	***Gossypium arboretum***	Angiosperms	GDKTHV	NTRAH
**A020|A0A4V6D778**	Protein kinase domain-containing protein	***Setaria viridis***	Angiosperms	GDKSHV	STRPA
**A021|A0A1S3CCG0**	Serine/threonine-protein kinase PBS1	***Cucumis melo***	Angiosperms	GDKSHV	STRPQ
**A022|A0A0L9TCZ6**	Protein kinase domain-containing protein	***Phaseolus angularis***	Angiosperms	GDKSHV	STQPH
**A023|A0A124SD39**	Protein kinase domain-containing protein	***Cynara cardunculus***	Angiosperms	GDKSHV	STAPQ
**A024|LOC111479363**	Serine/threonine-protein kinase PBS1-like	***Cucurbita maxima***	Angiosperms	GDKTHV	NTRGP
**A025|A0A5N6MQM2**	Protein kinase domain-containing protein	***Mikania micrantha***	Angiosperms	GDKTHV	NTRAP
**A028|LOC112282602**	Serine/threonine-protein kinase PBS1-like7	***Physcomitrella patens***	Moss	GDKTHV	NSRSA
**A029|D8RRM2**	Protein kinase domain-containing protein	***Selaginella moellendorffii***	Lycophyte	GDKTHV	NARPA
**A031|LOC110785742**	Serine/threonine-protein kinase PBS1	***Spinacia oleracea***	Angiosperms	GDKSHV	SNRPH
**A035|108196266**	Serine/threonine-protein kinase PBS1-like	***Daucus carota***	Angiosperms	GDKSHV	STQPQ
**A038|XP_018811839.1**	Serine/threonine-protein kinase PBS1	***Juglans regia***	Angiosperms	GDKSHV	GTRPH
**A042|A0A0Q3I194**	Serine/threonine-protein kinase PBS1	***Brachypodium distachyon***	Angiosperms	GDKSHV	STRPH
**A043|A0A2H5N6Y7**	Protein kinase domain-containing protein	***Citrus unshiu***	Angiosperms	GDKTHV	NTRPP
**A046|A0A4S8IPK9**	Protein kinase domain-containing protein	***Musa balbisiana***	Angiosperms	GDKSHV	TTKSH
**A048|LOC105168454**	Serine/threonine-protein kinase PBS1	***Sesamum indicum***	Angiosperms	GDKSHV	NTLPQ
**A053|ANC96959.1**	phototropin	***Equisetum hyemale***	Pteridophyta	VEREIL	SEEAS
**A054|EMS52724.1**	Serine/threonine-protein kinase PBS1	***Triticum urartu***	Angiosperms	GDKSHV	STRPH
**A056|A0A368RUW5**	Protein kinase domain-containing protein	***Setaria italica***	Angiosperms	GDKSHV	STRPA
**A057|LOC17884091**	serine/threonine-protein kinase PBS1	***Capsella rubella***	Angiosperms	GDKSHV	SEMPH
**A058|A0A2R6W6N6**	Protein kinase domain-containing protein	***Marchantia polymorpha***	Angiosperms	GDKTHV	NARAP
**A059|F6HDG9**	Protein kinase domain-containing protein	***Vitis vinifera***	Angiosperms	GDKTHV	NNRAA
**A062|A0A2G5DLW4**	Protein kinase domain-containing protein	***Aquilegia coerulea***	Angiosperms	GDKTHV	NTRGP
**A063|A0A4D8Z9L6**	serine/threonine-protein kinase PBS1	***Salvia splendens***	Angiosperms	GDKTHV	NNRGA
**A064|A0A2K1KFP5**	Protein kinase domain-containing protein	***Physcomitrella patens***	Moss	GDKTHV	NARAV
**A066|A0A2R6PK59**	serine/threonine-protein kinase	***Actinidia chinensis***	Angiosperms	GDKTHV	NTRVA
**A068|A0A1Q3BYQ7**	Pkinase domain-containing protein	***Cephalotus follicularis***	Angiosperms	GDKTHV	NARTH

**A070|A0A2I0B394**	serine/threonine-protein kinase PBS1	***Apostasia shenzhenica***	Angiosperms	GDKTHV	NTRPT
**A071|D8SMJ2**	Protein kinase domain-containing protein	***Selaginella moellendorffii***	Lycophyte	GDKTHV	NSRPA
**A075|A0A4Y7IXE0**	Protein kinase domain-containing protein	***Papaver somniferum***	Angiosperms	GDNTHV	NSRAV
**A077|A0A2I0XBR2**	serine/threonine-protein kinase PBS1	***Dendrobium catenatum***	Angiosperms	GDNTHV	NTRAA
**A079|A0A1E5VW28**	serine/threonine-protein kinase PBS1	***Dichanthelium oligosanthes***	Angiosperms	GDKSHV	STRPA
**A080|A0A200Q1K2**	Protein kinase domain-containing protein	***Macleaya cordata***	Angiosperms	GDNTHV	NSRPH
**A087|LOC104415649**	*serine/threonine-protein kinase PBS1*	***Eucalyptus grandis***	Angiosperms	GDKSHV	SSRPH
**A090|A0A1U7ZAQ8**	serine/threonine-protein kinase PBS1	***Nelumbo nucifera***	Angiosperms	GDKSHV	STRAH
**A096|A0A199W2Y9**	serine/threonine-protein kinase PBS1	***Ananas comosus***	Angiosperms	GDKTHV	NTRPT
**A099|111375917**	Serine/threonine-protein kinase PBS1-like	***Olea europaea***	Angiosperms	GDKSHV	GTRPH
**A101|103986028**	serine/threonine-protein kinase PBS1	***Musa acuminata***	Angiosperms	GDKSHV	SALPH
**A103|A0A2H3WYF2**	serine/threonine-protein kinase PBS1	***Phoenix dactylifera***	Angiosperms	GDKSHV	STRAH
**A108|A0A3L6QHY7**	Protein kinase domain	***Panicum miliaceum***	Angiosperms	GDKSHV	STRPH
**A110|LOC18434449**	Serine/threonine-protein kinase PBS1-like-27	***Amborella trichopoda***	Angiosperms	GDKSHV	STRAP
**A112|A0A0K9PTD6**	serine/threonine-protein kinase PBS1	***Zostera marina***	Angiosperms	GDKSHV	STKPH
**A118|Kaladp0040s0137.1**	serine/threonine-protein kinase PBS1	***Ziziphus jujuba***	Angiosperms	GDKSHV	GARPH
**A126|LOC8067569**	Serine/threonine-protein kinase PBS1-like-7	***Sorghum bicolor***	Angiosperms	SSSSSK	ASRPD
**A128|LOC109774779**	Serine/threonine-protein kinase PBS1-like	***Aegilops tauschii***	Angiosperms	EEEKDG	ESRPS
**A130|A0A5E4EWW2**	serine/threonine-kinase	***Prunus dulcis***	Angiosperms	EDKSHV	SNRPH
**A133|GBG73041.1**	HYPOTHETICAL PROTEIN	***Chara braunii***	Charophyta	GGETHV	ATRPH
**A134|ADX68910.1**	brassinosteroid-like receptor protein	***Picea abies***	Gymnosperms	-	-KEEF
**A135|ATP67107.1**	HYPOTHETICAL PROTEIN	***Pinus pinaster***	Gymnosperms	GDKTHV	NNRAA
**A136|AcvPHY3**	Phytocrome 3 PHY3	***Adiantum capillus-veneris***	Pteridophyta	AEREIL	SERTA
**A137|ACY92450.1**	neochrome	***Lindsaea austrosinica***	Pteridophyta	VEREIL	SEEAA
**A138|AML79240.1**	putative LOV domain-containing protein	***Taxus baccata***	Gymnosperms	TEREIL	REDAV
**A140|AHZ63854.1**	phototropin	***Gnetum montanum***	Gymnosperms	AERDIL	NEEAA
**A141|GAQ85442.1**	Protein kinase domain	***Klebsormidium nitens***	Charophyta	GDKTHV	SSRPR
**A142|BAF79980.1**	receptor-like kinase	***Nitella axillaris***	Charophyta	QRFSIS	LTQPE
**A144|A0A443P8A0**	Serine/threonine-protein kinase PBS1	***Cinnamomum micranthum***	Angiosperms	GDKSHV	SSRPH
**A146|AML78026.1**	putative LOV domain-containing protein	***Lindsaea linearis***	Pteridophyta	AEREIL	SERAA
**A147|Pavir.Aa02161.1**	Serine/threonine-protein kinase PBS1	***Panicum virgatum***	Angiosperms	GDKSHV	STRPH
**A148|APU94870.1**	leucine-rich repeat receptor-like protein kinase	***Pohlia nutans***	Moss	GSEREV	SSRPR
**A149|AML79064.1**	putative LOV domain-containing protein	***Lonchitis hirsuta***	Pteridophyta	AEREIL	SEETA
**A152|LOC110877396**	Serine/threonine-protein kinase PBS1-like	***Helianthus annuus***	Angiosperms	QSIPYV	-MHNG
**A160|A0A5J9V222**	Protein kinase domain	***Eragrostis curvula***	Angiosperms	GDKSHV	STRPH
**A161|A0A0D9YJR9**	Protein kinase domain	***Oryza glumipatula***	Angiosperms	GDKTHV	NTKPQ
**A162|Sphfalx0021s0054.1**	Protein kinase domain	***Sphagnum fallax***	Moss	GDKTHV	NARAA
**A167|LOC112283883**	Serine/threonine-protein kinase PBS1-like7	***Physcomitrella patens***	Moss	GDKTHV	ESRGP

**A168|A0A2U1NTM5**	Protein kinase domain	***Artemisia annua***	Angiosperms	GDKTHV	NTRAP
**A170|8AT94**	Protein kinase domain	***Oryza sativa subsp. Indica***	Angiosperms	GDKSHV	STRPH
**A175|A0A068TRM7**	Protein kinase domain	***Coffea canephora***	Angiosperms	GDKTHV	NTRGP
**A180|A0A4S4DNN2**	Protein kinase domain	***Camellia sinensis***	Angiosperms	GDKTHV	NTRAA
**A185|Tp57577**	Serine/threonine-protein kinase PBS1	***Trifolium pratense***	Angiosperms	GDKSHV	STRPH
**A186|ALR83541.1**	leucine-rich repeat receptor-like kinase	***Adiantum aethiopicum***	Pteridophyta	ES----	-AEYG
**A189|LOC109175864**	Serine/threonine-protein kinase PBS1	***Ipomoea nil***	Angiosperms	GDKSHV	STQPP
**A199|A0A5A7RGI9**	Protein kinase domain	***Striga asiatica***	Angiosperms	GDKTHV	NMRSA
**A215|LALR83540.1**	*Leucine-rich repeat receptor-like kinase*	***Ginkgo biloba***	Ginkgophyta	ES----	-SEFG
**A221|LOC105034278**	Serine/threonine-protein kinase PBS1-like	***Asparagus officinalis***	Angiosperms	GDKSHV	STRAH
**A222|LOC105048968**	Serine/threonine-protein kinase PBS1	***Elaeis guineensis***	Angiosperms	GDKSHV	NSRPQ
**A224|A0A5D3ADM4**	Protein kinase domain	***Gossypium mustelinum***	Angiosperms	GDKTHV	NMRAP
**A229|ADE76472.1**	unknown	***Picea sitchensis***	Gymnosperms	GDKTHV	NSRSA
**A231|LOC110027867**	Serine/threonine-protein kinase PBS1-like	***Phalaenopsis equestris***	Angiosperms	GDQSHV	SSRAH
**A233|LOC108844003**	Serine/threonine-protein kinase PBS1	***Raphanus sativus***	Angiosperms	GDKSHV	TELPH
**A236|A0A328CZG9**	Protein kinase domain	***Cuscuta australis***	Angiosperms	GDKSHV	STQPP
**A240|LOC105958008**	Serine/threonine-protein kinase PBS1	***Erythranthe guttata***	Angiosperms	GDKSHV	STRPQ
**A248|XP_024388663.1**	receptor-like cytoplasmic kinase 176	***Physcomitrella patens***	Moss	GDKTHV	KNRPS
**A271|Oropetium_20150105_05286A**	Serine/threonine-protein kinase PBS1	***Oropetium thomaeum***	Angiosperms	GDKTHV	NTKPH
**A277|LOC111905833**	Serine/threonine-protein kinase PBS1-like18	***Lactuca sativa***	Angiosperms	GDWSHV	YKRVA
**A279|KFK32284.1**	Protein kinase domain	***Arabis alpine***	Angiosperms	GDETHV	HTKAR
**A288|A0A1R3KLX9**	Protein kinase domain	***Corchorus olitorius***	Angiosperms	GDKTHV	NSKTG
**A290|PAN50235.1**	Serine/threonine-protein kinase PBS1-like7	***Panicum hallii***	Angiosperms	GDNTHV	ESLPE
**A292|102712431**	Serine/threonine-protein kinase PBS1	***Oryza brachyantha***	Angiosperms	GDDTHI	DSRAK
**A295|SELMODRAFT_110362**	protein STRUBBELIG-RECEPTOR FAMILY 8	***Selaginella moellendorffii***	Lycophyte	SLERQI	SSRPR
**A302|SELMODRAFT_80744**	hypothetical protein	***Selaginella moellendorffii***	Lycophyte	MGNTHV	MNRPR
**A306|LOC8285769**	Serine/threonine-protein kinase PBS1-like10	***Ricinus communis***	Angiosperms	DDKTHV	PHLPI
**A313|D8S619**	Protein kinase domain-containing protein	***Selaginella moellendorffii***	Lycophyte	GDKTHV	NSRPA
**A316|SELMODRAFT_112070**	STRUBBELIG-RECEPTOR FAMILY 8	***Selaginella moellendorffii***	Lycophyte	GAERQV	SSRSR
**A343|AFG44754.1**	hypothetical protein	***Pinus taeda***	Gymnosperms	EKSYVT	-----
**A344|ACH59260.1**	LRR receptor-like protein kinase, partial	***Pseudotsuga menziesii***	Gymnosperms	-	-----
**A349|A0A1U8E6G8**	Serine/threonine-protein kinase PBS1	***Capsicum annuum***	Angiosperms	GDKTHV	NTRSH
**A350|A0A0D2Q6X1**	Protein kinase domain	***Gossypium raimondii***	Angiosperms	GDKSHV	STRPH
**A351|A0A397XMK6**	Protein kinase domain	***Brassica campestris***	Angiosperms	GDKSHV	TDMPH
**A352|LOC112282882**	Serine/threonine-protein kinase PBS1-like7	***Physcomitrella patens***	Moss	GDKTHV	NSRAA
**A358|A0A0D3G6V3**	Protein kinase domain	***Oryza barthii***	Angiosperms	GDKTHV	NTKPL
**A364|A0A2P6R064**	Protein kinase domain	***Rosa chinensis***	Angiosperms	GDKSHV	STRPH
**A365|LOC18788970**	Serine/threonine-protein kinase PBS1	***Prunus persica***	Angiosperms	EDKSHV	SNRPH
**A367|101256183**	serine/threonine-protein kinase PBS1	***Solanum lycopersicum***	Angiosperms	GDKSHV	STKPQ

**A375|A0A067JSB9**	Protein kinase domain	***Jatropha curcas***	Angiosperms	GDKSHV	STRPH
**A376|XP_002871549.1**	serine/threonine-protein kinase PBS1	***Arabidopsis lyrata***	Angiosperms	GDKSHV	SEMPH
**A379|Thhalv10013511m**	serine/threonine-protein kinase PBS1	***Eutrema salsugineum***	Angiosperms	GDKSHV	SEMPH
**A381|XP_009125889.1**	serine/threonine-protein kinase PBS1	***Brassica rapa***	Angiosperms	GDKSHV	TDMPH
**A383|A0A0E0G784**	Protein kinase domain	***Oryza nivara***	Angiosperms	GDKSHV	STRPH
**A384|LOC4329463**	serine/threonine-protein kinase PBS1	***Oryza sativa Japonica***	Angiosperms	GDKSHV	STRPH
**A385|LOC102630702**	Serine/threonine-protein kinase PBS1-like27	***Citrus sinensis***	Angiosperms	GDKTHV	NTRPP
**A397|A0A1J6KHY8**	serine/threonine-protein kinase PBS1	***Nicotiana attenuate***	Angiosperms	GDKTHV	NARSH
**A411|A0A2P5E436**	Mitogen-activated protein kinase kinase kinase	***Parasponia andersonii***	Angiosperms	GDKTHV	NTRAQ
**A412|A0A2P5ERU2**	Serine/threonine protein kinase	***Trema orientale***	Angiosperms	GDKTHV	NTRAQ
**A418|103627200**	serine/threonine-protein kinase PBS1	***Zea mays***	Angiosperms	GDKSHV	STRPH
**A421|11442947**	Serine/threonine-protein kinase PBS1-like	***Medicago truncatula***	Angiosperms	GDKTHV	NTRGH
**A428|A0A0D9WFT6**	Protein kinase domain	***Leersia perrieri***	Angiosperms	GDKTHV	NTKPL
**A444|D8RXH9**	Protein kinase domain	***Selaginella moellendorffii***	Lycophyte	GDKTHV	NARPA
**A446|A0A445D9T9**	Protein kinase domain	***Arachis hypogaea***	Angiosperms	GDKSHV	STQPH
**A452|A0A0S3SKZ1**	Protein kinase domain	***Vigna angularis***	Angiosperms	GDKSHV	STRPH
**A454|A0A314UFY7**	Serine/threonine-protein kinase PBS1 isoform X1	***Prunus yedoensis***	Angiosperms	EDKSHV	SNRPH
**A461|LOC110639117**	Serine/threonine-protein kinase PBS1-like	***Hevea brasiliensis***	Angiosperms	GDKSHV	STRPH
**A462|LOC112033137**	Serine/threonine-protein kinase PBS1	***Quercus suber***	Angiosperms	GDKSHV	STRPH
**A464|A0A1U8M516**	Serine/threonine-protein kinase PBS1-like	***Gossypium hirsutum***	Angiosperms	GDKSHV	SSQPH
**A467|LOC111318441**	Serine/threonine-protein kinase PBS1	***Durio zibethinus***	Angiosperms	GDKSHV	STRPH
**A468|A0A2C9UNZ8**	Protein kinase domain	***Manihot esculenta***	Angiosperms	GDKSHV	SSQPH
**A469|A0A5N5NAY9**	Protein kinase domain	***Salix brachista***	Angiosperms	GDKSHV	STRPH
**A470|B9GZC6**	Protein kinase domain	***Populus trichocarpa***	Angiosperms	GDKSHV	SSRPH
**A471|LOC105133734**	Serine/threonine-protein kinase PBS1-like	***Populus euphratica***	Angiosperms	GDKSHV	SAQPH
**A475|A0A1S2Y0L1**	Serine/threonine-protein kinase PBS1 isoform X1	***Cicer arietinum***	Angiosperms	GDKSHV	GTRPH
**A477|XP_010419881.2**	Serine/threonine-protein kinase PBS1	***Camelina sativa***	Angiosperms	GDKSHV	SEMPH
**A481|A0A151TDB0**	Serine/threonine-protein kinase PBS1	***Cajanus cajan***	Angiosperms	GDKSHV	STQPQ
**A484|LOC104810211**	Serine/threonine-protein kinase PBS1	***Tarenaya hassleriana***	Angiosperms	GDQSHV	SEKPH
**A487|A0A2G9HHZ2**	Serine/threonine protein kinase	***Handroanthus impetiginosus***	Angiosperms	GDKSHV	STRPQ
**A497|AHZ63816.1**	neochrome	***Dryopteris expansa***	Pteridophyta	AEREIL	SEEAA
**A498|AHZ63819.1**	neochrome	***Plagiogyria japonica***	Pteridophyta	AEREIL	SEEXA
**A501|A0A0E0K0A0**	Protein kinase domain	***Oryza punctata***	Angiosperms	GDKSHV	STRPH
**A510|A0A2K1KK32**	Protein kinase domain	***Physcomitrella patens***	Moss	GDKTHV	NSRSA
**A512|A0A5B6WLN4**	Serine/threonine-protein kinase CDL1-like	***Gossypium australe***	Angiosperms	GDKTHV	NTRAP
**A517|A0A540MVB6**	Protein kinase domain	***Malus baccata***	Angiosperms	GDKTHV	NTRGP
**A518|A0A5N5HB58**	Serine/threonine-protein kinase PBS1-like	***Pyrus ussuriensis***	Angiosperms	GDKTHV	NTRGP

**A544|A0A061EVX3**	Serine/threonine-protein kinase PBS1	***Theobroma cacao***	Angiosperms	GDKTHV	NTRAP
**A545|A0A0A0L562**	Protein kinase domain	***Cucumis sativus***	Angiosperms	GDKTHV	NTRGP
**A570|A0A5N6RCT3**	Protein kinase domain	***Carpinus fangiana***	Angiosperms	GDKTHV	NTRAP
**A573|A0A2G2XQZ3**	Serine/threonine-protein kinase PBS1	***Capsicum baccatum***	Angiosperms	GDKTHV	NTRSH
**A581|LOC104101148**	Serine/threonine-protein kinase PBS1-like7	***Nicotiana tomentosiformis***	Angiosperms	GDKTHV	NARSH
**A593|LOC110711532**	Serine/threonine-protein kinase PBS1-like	***Chenopodium quinoa***	Angiosperms	GDKSHV	SDRPH
**A601|LOC104897507**	Serine/threonine-protein kinase PBS1	***Beta vulgaris***	Angiosperms	GDKSHV	SDRPH
**A632|A0A1S3TTF7**	Serine/threonine-protein kinase PBS1	***Vigna radiata***	Angiosperms	GDKSHV	STQPH
**A635|A0A1S5VH72**	Serine/threonine-protein kinase PBS1	***Triticum aestivum***	Angiosperms	GDKSHV	STRPH
**A642|Brast09G061900.1**	Serine/threonine-protein kinase PBS1-like	***Brachypodium stacei***	Angiosperms	GDKSHV	STRPH
**A649|LOC109357403**	Serine/threonine-protein kinase PBS1-like	***Lupinus angustifolius***	Angiosperms	GDKSHV	SNQPH
**A650|XP_015962894.1**	Serine/threonine-protein kinase PBS1	***Arachis duranensis***	Angiosperms	GDKSHV	STQPH
**A654|I1NJF4**	Serine/threonine-protein kinase PBS1-3	***Glycine max***	Angiosperms	GDKSHV	STRPH
**A665|LOC103929032**	Serine/threonine-protein kinase PBS1-like	***Pyrus x bretschneideri***	Angiosperms	GDKSHV	GDRPH
**A666|LOC110810800**	Serine/threonine-protein kinase PBS1	***Carica papaya***	Angiosperms	GDKSHV	STLPH
**A668|XP_008222220.1**	Serine/threonine-protein kinase PBS1	***Prunus mume***	Angiosperms	EDKSHV	SNRPH
**A669|XP_030960494.1**	Serine/threonine-protein kinase PBS1	***Quercus lobata***	Angiosperms	GDKSHV	STRPH
**A675|TYH86183.1**	Protein kinase domain	***Gossypium tomentosum***	Angiosperms	GDKSHV	STRPH
**A676|KAE8686889.1**	Serine/threonine-protein kinase PBS1	***Hibiscus syriacus***	Angiosperms	GDKSHV	STRPH
**A678|LOC103403202**	Serine/threonine-protein kinase PBS1-like	***Malus domestica***	Angiosperms	GDKSHV	SDRPH
**A681|XP_028803729.1**	Serine/threonine-protein kinase PBS1	***Prosopis alba***	Angiosperms	GDKSHV	STRPH
**A682|SapurV1A.0453s0170.1**	Serine/threonine-protein kinase PBS1	***Salix purpurea***	Angiosperms	GDKSHV	STQPH
**A683|LOC111017471**	Serine/threonine-protein kinase PBS1	***Momordica charantia***	Angiosperms	GDKSHV	STRPQ
**A692|XP_027353340.1**	Serine/threonine-protein kinase PBS1	***Abrus precatorius***	Angiosperms	GDKSHV	SSQPQ
**A695|XP_021291854.1**	Serine/threonine-protein kinase PBS1	***Herrania umbrática***	Angiosperms	GDKSHV	STQPH
**A699|CAA7039895.1**	Protein kinase domain	***Microthlaspi erraticum***	Angiosperms	GDKSHV	SEMPH
**A704|XP_013667958.1**	Serine/threonine-protein kinase PBS1	***Brassica napus***	Angiosperms	GDKSHV	TDMPH
**A706|XP_030496845.1**	Serine/threonine-protein kinase PBS1	***Cannabis sativa***	Angiosperms	GDKSHV	STRPH
**A709|111396104**	Serine/threonine-protein kinase PBS1-like	***Olea europaea var. Sylvestris***	Angiosperms	GDKSHV	GTQPH
**A711|VVB14322.1**	Protein kinase domain	***Arabis nemorensis***	Angiosperms	GDKSHV	SEMPH
**A713|Peaxi162Scf00128g01633.1**	Serine/threonine-protein kinase PBS1	***Nicotiana sylvestris***	Angiosperms	GDKSHV	STKPQ
**A714|NIOBTv3_g34227**	serine/threonine-protein kinase PBS1-like	***Nicotiana tabacum***	Angiosperms	GDKSHV	STMPQ
**A715|Kalax.0401s0019.1**	Serine/threonine-protein kinase PBS1	***Kalanchoe laxiflora***	Angiosperms	GDKSHV	GTRPH
**A716|Niben101Scf02996g03008.1**	Serine/threonine-protein kinase PBS1a	***Nicotiana benthamiana***	Angiosperms	GDKSHV	STMPQ
**A722|VDC78705.1**	Protein kinase domain	***Brassica campestris***	Angiosperms	GDKSHV	SEMPH
**A723|Bol034221**	Serine/threonine-protein kinase PBS1	***Brassica oleracea capitata***	Angiosperms	GDKSHV	SEMPH
**A734|LOC111258252**	Serine/threonine-protein kinase PBS1-like25	***Setaria italica***	Angiosperms	SSSSTG	ASRPD
**A739|XP_021816394.1**	Serine/threonine-protein kinase PBS1	***Prunus avium***	Angiosperms	EDKSHV	SNRPH

**A755|W9RNM0**	Serine/threonine-protein kinase PBS1	***Morus notabilis***	Angiosperms	GDKTHV	NSRSA
**A758|S8BXP4**	Protein kinase domain	***Genlisea aurea***	Angiosperms	GDKTHV	NSRAA

[a] In PBS1 from *Arabidopsis thaliana* the cleavage site motif GDK has been studied. [b] The SEMPH and STRPH motifs of PBS1 have been studied in *Arabidopsis thaliana* (Qi et al., 2014) and *Triticum aestivum cv.* (Sun et al., 2017) respectively.

The different motifs found in PBS1 from various species were obtained according to the alignment using the SEMPH and GDK motif from *Arabidopsis thaliana* as reference.
