# Supplementary material for: An Ancestry Perspective of the Evolution of PBS1 Proteins in Plants
Source: Int J Mol Sci. 2021 Jun 25;22(13):6819. doi: 10.3390/ijms22136819 (PMC8269361; doi:10.3390/ijms22136819)

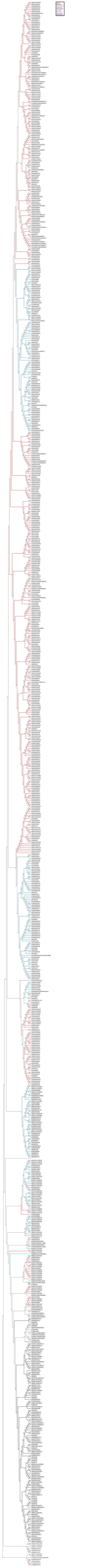

Supplement: Supplementary file 1 [file supplementary figure 2.pdf › supplementary figure 2.pdf]
